# *PLOS ONE* 2016 Reviewer and Editorial Board Thank You

**DOI:** 10.1371/journal.pone.0174259

**Published:** 2017-03-20

PLOS and the *PLOS ONE* team would like to express our sincere gratitude to all of you who lent us your valuable time and expertise as an academic editor, as a guest editor, or as a peer reviewer in 2016. We are fully dependent on your voluntary efforts to publish *PLOS ONE* for the larger academic community, and we could not carry forward our mission without you. In 2016, *PLOS ONE* depended upon over 5,000 academic and guest editors to manage the nearly 50,000 articles submitted, which were assessed by nearly 70,000 reviewers. Together, your efforts enabled the publication of more than 22,000 rigorously reviewed Open Access papers.

The names of our editors that handled submitted manuscripts in 2016 appear in the Supporting Information as S1 Editor List. Our reviewers appear in the Supporting Information as S1–S5 Reviewer Lists. Thank you all! We are exceedingly grateful for your dedication, generosity, and support of Open Access science.

## Supporting information

S1 Editor List Alphabetical list of 2016 *PLOS ONE* Active Editorial Board Members. (PDF)

S1 Reviewer List Alphabetical list of 2016 *PLOS ONE* peer reviewers (A–D). (PDF)

S2 Reviewer List Alphabetical list of 2016 *PLOS ONE* peer reviewers (E–J). (PDF)

S3 Reviewer List Alphabetical list of 2016 *PLOS ONE* peer reviewers (K–O). (PDF)

S4 Reviewer List Alphabetical list of 2016 *PLOS ONE* peer reviewers (P–T). (PDF)

S5 Reviewer List Alphabetical list of 2016 *PLOS ONE* peer reviewers (U–Z). (PDF)

## References

[1] (2016) *PLOS ONE* 2015 Reviewer Thank You. PLoS ONE 11(2): e0150341 doi: 10.1371/journal.pone.0150341

[2] (2015) *PLOS ONE* 2014 Reviewer Thank You. PLoS ONE 10(2): e0121093 doi: 10.1371/journal.pone.0121093.

