# Supplementary material for: PLOS ONE 2016 Reviewer and Editorial Board Thank You
Source: PLoS One. 2017 Mar 20;12(3):e0174259. doi: 10.1371/journal.pone.0174259 (PMC5358840; doi:10.1371/journal.pone.0174259)
Supplement: S1 Editor List — (PDF) [file pone.0174259.s001.pdf]

PLOS | ONE

PLOS ONE would like to thank all those who served on the journal's Editorial Board or as Guest Academic Editors in 2016:

Katriina Aalto-Setala
María Abad-Grau
Ashraf Abdel-Naim
Amar Abderrahmani
Zaid Abdo
Amir Abdollahi
Hideharu Abe
Keiko Abe
Takeru Abe
Toshiaki Abe
Pasquale Abete
P.C. Abhilash
Alash'le Abimiku
Zsolt Ablonczy
Abdelilah Aboussekhra
Thomas Abraham
Sigal Abramovich
William Abrams
Yael Abreu-Villaça
Yousef Abu Kwaik
Osama Abulseoud
Serena Aceto
Varenyam Achal
Krishnendu Acharya
Alessandro Achilli
Ted Acott
Rodney Adam
Andrew Adamatzky
Jean Adams
Michelle Adams
Amy Adamson
Elsa Addessi
Christina Addison
Zach Adelman
Paul Adlard
Christof Aegerter
Kamyar Afarinkia
Gijs Afink
Farhat Afrin
Pradeep Agarwal
Pratul Agarwal
Rajesh Agarwal
Ritesh Agarwal
Sudha Agarwal
Gabriel Agbor
Ashutosh Aggarwal
Pierfrancesco Agostoni
Irina Agoulnik
Christian Agrillo
Francisco Aguayo
Marta Agudo-Barriuso
Marcia Aguila
Ruben Aguilar
Abelardo Aguilera
Helmut Ahammer
Golo Ahlenstiel
Aamir Ahmad
Faiz Ahmad
Muzamil Ahmad
Rasheed Ahmad
Shama Ahmad
Aamir Ahmed
Niyaz Ahmed
S. Ashraf Ahmed
Shawn Ahmed
Byeong-Cheol Ahn
Sang Hoon Ahn
Ingo Ahrens
Sunil Ahuja
Jyrki Ahveninen
Jing Ai
Jinglu Ai
Tomohiko Ai
Xun Ai
Eugene Aidman
Elena Aikawa
Masanori Aikawa
Tadayuki Akagi
Fadi Akar
Yoshiki Akatsuka
Suminori Akiba
Taishin Akiyama
Orhan Aktas
Elkan Akyürek
A. Al-Ahmad
Claude Alain
Nehad Alajez
William Alazawi
Angel Alberich-Bayarri
Jose Alberola-Ila
Emidio Albertini
Urs Albrecht
Benedicte Albrectsen
Rafael Aldabe
Alexander Alekseyenko
Branko Aleksic
André Aleman
Marià Alemany

Riccardo Alessandro
Silvia Alessi-Severini
Sheila Alexander
Lena Alexopoulou
Kristin Al-Ghoul
Jauhar Ali
Mohammad Ali
Raghib Ali
Evren Alici
Mani Alikhani
Alberto Aliseda
Anna Alisi
Benjamin Allen
Irving Allen
Philip Allen
Rachel Allen
Bernadette Allinquant
David Allison
Silvana Allodi
Alejandro Almarza
Adelaide Almeida
Graca Almeida-Porada
Ana Almodóvar
Marta Alonso
Gianfranco Alpini
Thierry Alquier
Mohammed Alsharifi
Berta Alsina
Christian Altmann
Eduardo Altmann
M. Lucrecia Alvarez
Inés Álvarez
Diego Alvarez de la Rosa
Gualtiero Alvisi
Stephen Alway
Sara Amancio
Salomon Amar
Luís Amaral
Frederic Amblard
Zandrea Ambrose
Roberto Ambrosini
Carlos Ambrósio
Thierry Amédée
Roberto Amendola
Janaki Amin
Sygal Amitay
Nicola Amodio
Lingling An
Madhur Anand
Shrikant Anant
Andrew Anderson
Kurt Anderson
Matthew Anderson
Michael Anderson
Rozalyn Anderson
David Andes
Claudia Andl
Usha Andley
Giuseppe Andò
Shaida Andrabi
Paula Andrade
Miguel Andrade-Navarro
Isabelle Andre
Frédéric André
Graciela Andrei
Zane Andrews
Ioannis Androulakis
Juan Añel
Darwin Ang
Peter Angeletti
Adriano Angelucci
Philip Anglewicz
Arga Anil
Maria Anisimova
Muna Anjum
Alexander Annala
Lucio Annunziato
Aftab Ansari
Daniel Ansari
Kausar Ansari
Andrea Antal
Ruby Anto
Alessandro Antonietti
Christophe Antoniewski
Antony Antoniou
Maxim Antopolsky
Ichiro Aoki
Cristian Apetrei
Marco Apollonio
Vasu Appanna
Silke Appel
Muhammad Aqeel Ashraf
Ehsan Arabzadeh
Hossam Arafa
Ken Arai
Gururaj Arakeri
Wagner Araujo
Ronaldo Araújo
Filippos Aravanopoulos
Natarajan Aravindan
Jack Arbiser
Philippe Archambault
Luca Ardigò
Thomas Arendt
Benjamin Arenkiel
Ramon Arens
Ana Paula Arez
Oscar Arias-Carrion
Kevin Ariën
Hiroyoshi Ariga

Mehrdad Arjomandi
Dan Arking
Robert Arkowitz
Raphaël Arlettaz
Ines Armando
Cristina Armas
Anna Armitage
Cherie Armour
Dawn Arnold
Derek Arnold
Ricardo Aroca
Raffi Aroian
Mark Aronoff
Pankaj Arora
Rajesh Arora
Jorge Arreola
Valder Arruda
Ruben Artero
Wolfgang Arthofer
Thiruma Arumugam
Atsushi Asakura
Masato Asanuma
Hitoshi Ashida
Ali Ashkar
Hassan Ashktorab
Hossam Ashour
Nick Ashton
Candice Askwith
Yan Asmann
Monika Asnani
Pontus Aspenstrom
Patricia Aspichueta
Shervin Assassi
Eric Asselin
Piia Astikainen
Dhammika Atapattu
Timothy Atherton
Stephen Atkin
Bernard Attali
Houssam Attoui
Paul Atzberger
Harald Auge
Tim Aumann
Holger Auner
Ferdinando Auricchio
Alessio Avenanti
Matias Avila
Jesus M. Avilés
Massimo Avoli
Ram Avtar
Hani Awad
Belay Ayele
Nukhet Aykin-Burns
Eduard Ayuso
Samy Azer
Luciano Azevedo
Jan Baak
Horacio Bach
Markus Bachschmid
Ryan Bachtell
Peter Backx
Tudor Badea
Joel Bader
Michael Bader
Jonathan Badger
Christopher Bae
Kwang-Hyun Baek
Maria Baer
Andrew Baggaley
Carolyn Baglole
Serena Bagnasco
Pantelis Bagos
Yong-Sun Bahn
Chunxue Bai
Guihua Bai
Xue-feng Bai
Yidong Bai
David Bailey
Paul Baird
Niranjan Baisakh
Vladimir Bajic
Vivek K. Bajpai
Jennifer Baker
Julien Baker
Michelle Baker
Kithiganahalli Balaji
Ramesh Balasubramaniam
Muthuswamy Balasubramanyam
Jose Luis Balcazar
Fausto Baldanti
Robert Baldwin
Troy Baldwin
Raffaella Balestrini
Mitchell Balish
Grant Ballard
Esteban Ballestar
Daniel Ballhorn
Ramesh Balusu
Giorgos Bamias
Karin Bammann
Syuhei Ban
Obul Bandapalli
Christianne Bandeira de Melo
Thomas Bandholm
Bruce Banfield
Krystof Bankiewicz
Lawrence Banks
Sam Banks
Geetha Bansal
Vipul Bansal

Pedro Baptista
Stefan Baral
Danny Barash
Olivier Barbier
Tiago Barbosa
Andrea Barbuti
Joseph J Barchi
Kim Bard
Barbara Bardoni
William Barendse
Noel Barengo
Carolina Barillas-Mury
Jacob Barkley
Ruanne Barnabas
Steven Barnes
Jean-Claude Baron
Marco Baroni
Muhammad Barozai
Alain Barrat
Pasqual Barretti
Gregory Barsh
Paul Bartell
Alessandro Bartolomucci
Jason Barton
Richard Barton
Birke Bartosch
Jason Bartz
Mikael Bask
Ilia Baskakov
Tobias Baskin
Diane Bassham
Martine Bassilana
Hormoz BassiriRad
Brock Bastian
Joyoti Basu
Nandita Basu
Sujit Basu
Warren Batchelor
Tone Bathen
Surinder Batra
Da-Tian Bau
Chris Bauch
Olivier Baud
Michael Baudis
Michel Baudry
Joseph Bauer
Wolfgang Bauer
Mathias Baumert
Heiner Baur
Ivan Baxter
Antony Bayer
Matthew Baylis
Jagadeesh Bayry
Maxim Bazhenov
Bernard Beall
Carrie Bearden
Shawn Bearden
Elaine Bearer
Brian Beatty
Cristina Becchio
Eduard Beck
Karsten Becker
Nigel Beebe
Jeffrey Beekman
Jeff Beeler
Martin Beer
Simon Beggs
Christopher Beh
Maik Behrens
Thomas Behrens
Andrea Belgrano
Scarlett Bellamy
Mark Bellgrove
Saverio Bellusci
Christophe Beloin
Robert Belshaw
Antonio Paolo Beltrami
Suliann Ben Hamed
Charaf Benarafa
Sompop Bencharit
Mohammed Bendahmane
Linda Bendall
Umberto Benedetto
José Bengoechea
Claudia Benjamim
Lbachir BenMohamed
Joshua Benoit
Panayiotis Benos
Sliman Bensmaia
Guy A.M. Berbers
Rebecca Berdeaux
Iris Berent
Gabriele Berg
Ivan Berg
Frank Berger
Thomas Berger
Lars Berglund
Andreas Bergmann
Sven Bergström
Anne Berit Skiftesvik
Olivia Bermingham-McDonogh
Daniel J. Bernard
Giacomo Bernardi
Gilbert Bernier
Harold Bernstein
Jean-Guy Berrin
Louis-Felix Bersier
Antonio Bertoletti
Francesco Bertolini
Cristiano Bertolucci

Luc Bertrand
Stéphanie Bertrand
Robert Berwick
Annamaria Bevivino
Suresh Bhargava
Surya Bhatt
Surajit Bhattacharjya
Samir Bhattacharya
Sanjoy Bhattacharya
Siladitya Bhattacharya
Sukesh Bhaumik
Neil Bhowmick
Anirban Bhunia
Sujit Bhutia
Zulfiqar Bhutta
Giuseppe Biagini
Cesario Bianchi
Luigi Bianchi
Faysal Bibi
Nuno Bicho
Hemant Bid
Zhilong Bie
Peter Biehl
Anja-Katrin Bielinsky
Brian Bigger
Patrick Biggs
Gary Bilotta
Verner Bingman
Alexander Binshtok
Franco Biondi
Anna Birukova
Nanette Bishopric
Sylvie Bisser
Animesh Biswas
Indranil Biswas
Kaushik Biswas
Peyman Björklund
Niklas Björkström
Petter Bjornstad
François Blachier
Keith Black
Peter Black
Jason Blackard
Ira Blader
Jeffrey Blanchard
Paul Blanchon
Miguel Blazquez
Wolfgang Blenau
Michelle Block
Bertrand Blondeau
Gareth Bloomfield
David Blum
Robert Blum
Miroslav Blumenberg
Norma Bobadilla
Pierre Bobé
Tomasz Bochenek
Jerzy Bodurka
Simon Body
Harm Bogaard
Titus Boggon
Kath Bogie
Steven Bograd
Matthew Bogyo
Gil Bohrer
Alexandre Boissonnas
Christian Bökel
Bazartseren Boldgiv
Chiara Bolego
Johan Bolhuis
Johan Bollen
Johannes Boltze
Subbarao Bondada
Vladimir Bondarenko
Luca Bondioli
Ben Bond-Lamberty
Ivo Boneca
Raffaella Bonecchi
Josh Bongard
Maciej Boni
Halvard Bönig
Ferruccio Bonino
Gianluca Bontempi
David Bonter
Adrianus Boon
David Boone
Craig Boote
David Booth
Thomas Boraud
Iman Borazjani
David Borchelt
Justin Borevitz
Renee Borges
Claudio Borghi
Kerstin Borgmann
Katherine Borkovich
Cesar Borlongan
Lutz Bornmann
Consuelo Borras
Francesca Borrelli
Steffen Borrmann
Ray Borrow
Santanu Bose
C Boswell
Michel Botbol
Donald Bottaro
Yvonne Böttcher
Abderrezak Bouchama
Michael Bouchard
Marie-Josée Boucher

Pierre Boudinot
Dmitri Boudko
Habib Boukerche
Thierry Boulinier
Michael Boulton
Gerrit Bouma
Jérémie Bourdon
Kostas Bourtzis
Vassiliki Boussiotis
Daniel Bouvard
Nicole Bouvier
Mark Bowden
Mary Bowen
Prosper Boyaka
Maxim Boyanov
Mark Boyce
Laura Boykin
Justin Boyles
Patricia Bozza
Monika Bradl
Robert Bradley
Sean Brady
Christian Braendle
Érika Braga
Paula Braitstein
Pablo Brañas-Garza
Igor Branchi
David Brand
Thomas Brand
Johanna Brandner
Elvira Brattico
Shawn Bratton
Lidia Braunstein
Kelly Brayton
Björn Brembs
Caroline Brennan
Jerome Breslin
Casper Breuker
Diego Breviario
Joanna Bridger
Matthias Briel
R. Mark Brigham
Volker Briken
Vera Bril
Hassan Brim
Catherine Brissette
Robert Britton
Guy Brock
Gudrun Brockmann
Jeffrey Brodsky
James Brody
Basil Brooke
James Brophy
Jürgen Brosius
Sarah Brosnan
Susan Broughton
David Brown
Jon Brown
Keith Brown
Kevin Brown
Sam Brown
Stephanie Brown
Glenn Browning
Stephanie Broyles
Amanda Bruce
Sonia Brucki
Holger Brüggemann
Helge Bruns
Lorenzo Brusetti
Klaus Brusgaard
Mary Bryk
Shilpa Buch
Maciej Buchowski
Jeffrey Buckel
Gavin Buckingham
Hikmet Budak
Laszlo Buday
Nediljko Budisa
Valquiria Bueno
Sven Buerki
Raffaele Bugiardini
Bang Bui
Catalin Buiu
Joseph Bump
Jacob Bundy
Kevin Bunting
Emanuele Buratti
Irina Burd
Emmanuel Burdmann
Harold Burgess
Gordon Burghardt
David Burgner
Robert Burk
Robert Burne
Thomas Burne
Richard Burney
Jonathan Burns
Jorge Burns
Kevin Burrage
Christina Bursill
Rainer Bussmann
Benedetta Bussolati
Pierre Busson
Patrick Butaye
Peter Butko
Michael Butterworth
Raffaella Buzzetti
Kimberly Byrnes
Huaibin Cai
Qiliang Cai

Tao Cai
Xiaodong Cai
Yang Cai
Laura Calabresi
Francesc Calafell
Jose Calbet
Adriana Calderaro
Christine Caldwell
Joseph Califano
George Calin
Raffaele Calogero
John Calvert
Diego Calvisi
Niels Câmara
D Cameron
Elissa Cameron
Sharon Cameron
Douglas Campbell
Moray Campbell
Kevin Camphausen
Patrizia Campolongo
Giovanni Camussi
Rouwen Canal-Bruland
Hector Candela
Peter Canoll
Dario Cantu
Bing-Yang Cao
Cong Cao
Heping Cao
Jian Cao
Ya Cao
Yongchang Cao
Cristian Capelli
Steve Caplan
Andrea Caporali
Francesco Cappello
Massimo Caputi
David Caramelli
Christopher Carcaillet
Pere-Joan Cardona
Marly Cardoso
Claudio Carere
Paolo Carloni
Clotilde Carlow
Morgan Carlson
Yohay Carmel
Tom Carney
David Carpenter
Luis Carretero
David Carrier
David Carter
Dee Carter
Kim Carter
Calogero Caruso
Denise Carvalho
Leonardo Carvalho
Luzia Carvalho
Dulce Casarini
Eric Cascales
Giovanni Casella
Maurizio Casiraghi
Philippe Castagnone-Sereno
Miguel Castanho
Sergi Castellví-Bel
Riccardo Castiglia
Filippo Castiglione
Martin Castonguay
Javier Castresana
Maria Castro
Alberico Catapano
Luigi Cattaneo
Byron Caughey
Andrea Cavalli
Giacomo Cavalli
Luigi Cavallo
Elena Cavarretta
Joan Caylà
Marek Cebecauer
Just Cebrian
Francesca Ceccherini-Silberstein
Nico Cellinese
Shan Cen
Valentin Ceña
Mara Cercignani
Cristina Cereda
Nicolas Cermakian
Vincenzo Cerullo
Jose Chabalgoity
Maurice Chacron
Christos Chadjichristos
Brian Chadwick
Karl Chai
Lisa Chakrabarti
Debasis Chakrabarty
Shukti Chakravarti
Dipshikha Chakravortty
Nicolas Chaline
Etienne Challet
James Chalmers
Jeffrey Chalmers
Martin Chalumeau
Mathias Chamaillard
Jamshidkhan Chamani
Alanna Chamberlain
Roger Chammas
Chris Chan
Christina Chan
Daniel Chan
David Chan
Franky Chan

Kelvin Chan
Michael CW Chan
Renee Chan
Sherine Chan
Zhulong Chan
Dhyan Chandra
Alice Y. W. Chang
Chin-Kuo Chang
Ing-Feng Chang
Jeffrey Chang
Yu-Jia Chang
Yung-Fu Chang
Tailoi Chan-Ling
Linda Chao
Maura (Gee) Chapman
Georges Chapouthier
Alain Charbit
Thierry Chardot
Charlotte Charpentier
Stéphane Charpier
Isabelle Charrier
Lucienne Chatenoud
Bandana Chatterjee
Delphi Chatterjee
Dipankar Chatterji
Rupesh Chaturvedi
Vishnu Chaturvedi
Christos Chatziantoniou
Gyaneshwer Chaubey
Gautam Chaudhuri
Ashok Chauhan
Neeraj Chauhan
Edward Chaum
Michael Chaussee
Franck Chauvat
Triantafyllos Chavakis
Pascale Chavatte-Palmer
Esteban Chaves-Olarte
Alain Chédotal
Farid Chehab
Leonardo Chelazzi
Srikumar Chellappan
Isabelle Chemin
Aimin Chen
Cathy Chen
Chaolun Chen
Chien-Sheng Chen
Chih-Jung Chen
Chi-Ling Chen
Chin-Tu Chen
Chunxian Chen
Clark Chen
Fan Chen
Guo-Qiang Chen
Han Chen
Han-Chiao Chen
Huei-Wen Chen
Jeremy Chen
Jing Chen
Jin-Gui Chen
Jin-Ran Chen
Jonathan Chen
Kewei Chen
Li Chen
Li-Mei Chen
Lin Chen
Li-Tzong Chen
Maohua Chen
Mike Chen
Peter Chen
Robert Chen
Shilin Chen
Suzie Chen
Tian-wu Chen
Tzong-Yueh Chen
Xianfeng Chen
Xiaoli Chen
Xinbin Chen
Xinguang Chen
Xiongwen Chen
Yan Chen
Yang-Ching Chen
Yanguang Chen
Yuan-Jia Chen
Zhiwei Chen
Zhong-Hua Chen
Zhongxue Chen
Jin Q. Cheng
Juei-Tang Cheng
Xianwu Cheng
Xiaodong Cheng
Zhihui Cheng
Zhukuan Cheng
Venugopalan Cheriyath
Stacey Cherny
Annie NY Cheung
Jonathan Chevrier
Jay Chhablani
Dante Chialvo
Keng-Hwee Chiam
Tzen-Yuh Chiang
Chuan-Chin Chiao
André Chiaradia
Lorenzo Chiariotti
Ornit Chiba-Falek
Glyn Chidlow
Cheng-Ting Chien
Roberto Chiesa
Joseph Chilcot
Wei-Chun Chin

Yick-Pang Ching
John Chiorini
Giuseppe Chirico
Nakul Chitnis
Chung-Jung Chiu
Kin-Sang Cho
Doo-Sup Choi
Je-Min Choi
Jonghoon Choi
Man-Yeon Choi
Sangdun Choi
Alexander Chong Shu-Chien
Kim-Kwang Raymond Choo
Imti Choonara
Arvind Chopra
Sanjay Chotirmall
Anuradha Chowdhary
Gerardo Chowell
Julie Chowen
Emanuel Christ
Hanna Christiansen
Brian Christie
Magdalena Chrzanowska-Wodnicka
Charleen Chu
Pei-Yi Chu
Eric Chuang
Jen-Hsiang Chuang
Michael Chung
Yeonseok Chung
Andrew Churg
Chih-Pin Chuu
Massimo Ciccozzi
Andrea Cignarella
Daniela Cimini
Roberta Cimmaruta
Caterina Cinti
Pietro Cipresso
Patrick Cirino
Vitaly Citovsky
Leon Claessens
Bruno Clair
Marc Claret
David Clark
Jesse Clark
Simon Clark
Timothy Clark
Stephen Clarke
Thomas Claudepierre
Paul Cleary
James Clelland
Julian Clifton
Axel Cloeckaert
Paul Cobine
Amanda Cockshutt
Luca Cocolin
Andrea Cocucci
Claudia Codeço
Laurent Coen
Loren Coen
Tom Coenye
Jorn Coers
Lark Coffey
Frederick Cohan
Irun Cohen
Noam Cohen
Malka Cohen-Armon
Ronald Cohn
Dilek Colak
Marco Colasanti
Raymond Colello
Melissa Coleman
Ross Coleman
William Coleman
Jonathan Coles
Donald Colgan
Gary Collins
James Collins
Giorgio Colombo
Gualtiero Colombo
Valery Combes
Fabio Cominelli
J. Alberto Conejero
John Conly
Karen Conneely
Philippe Connes
Che Connon
James Connor
Marcia Consolaro
Sofia Consuegra
M Consuelo del Cañizo
Mark Cookson
Austin Cooney
Brenton Cooper
Christine Cooper
David Copland
Domenico Coppola
Vincenzo Coppola
Richard Cordaux
Mario D. Cordero
Nils Cordes
Alex Córdoba-Aguilar
Estelle Cormet-Boyaka
Stephania Cormier
Stephen Cornell
Ignacio Correa-Velez
Aldo Corriero
Ilaria Corsi
Bernardo Cortese
Cristina Costa
Manuel Costa

Max Costa
Claudio Costa-Neto
Marcello Costantini
Alix Coste
Michael Costigan
Daniela Cota
Fenton Cotterill
Sue Cotterill
Roger Coulombe
Elizabeth Coulson
Franck Courchamp
Delphine Courvoisier
Michael Cousin
Francisco Couto
Mihai Covasa
Ashley Cowart
Benjamin Cowling
Dermot Cox
James Coyne
Mario Cozzolino
John Craft
Alister Craig
Robert Cramer
Corentin Cras-Méneur
Paolo Cravedi
Dana Crawford
Gary Crawford
James Cray Jr.
Chad Creighton
J. David Creswell
François Criscuolo
Fatima Crispi
Stefania Crispi
Jose Crispin
Marco Cristani
Hilary Critchley
Kevin Croce
Daniel Crocker
Simon Cropper
Elliott Crouser
Damian Crowther
Mathew Crowther
Anne Croy
Wim Crusio
Rogelio Cruz-Martinez
Peter Csermely
Laszlo Csernoch
Attila Csikász-Nagy
Ranji Cui
Zongbin Cui
Edna Cukierman
Aedín Culhane
Zoran Culig
Daniel Cullen
Richard Culleton
Mónica Cunha
Thiago Cunha
Edecio Cunha-Neto
Giuseppe Curcio
Walter Curioso
Kevin Currie
Bruce Cushing
Salvatore Cuzzocrea
Gennady Cymbalyuk
Lucette Cysique
Paula da Costa Martins
Monica da Silva Nunes
Marcel Daadi
Krystyna Dabrowska
Fulvio d'Acquisto
Clifford Dacso
Francesca D'Addio
Soheil Dadras
Daniele Daffonchio
Etienne Dague
Rajvir Dahiya
Helen Dahlke
Shaojun Dai
Gavin Daker-White
Felipe Dal Pizzol
Koustuv Dalal
Yamini Dalal
Andrew Dalby
Doralyn Dalisay
Hans Dam
Margot Damaser
Fábio DaMatta
Erik Danen
Christopher Danforth
Yong-hui Dang
Robert Dante
Ming Dao
Li Daqing
Andrea Dardis
Jean-Luc Darlix
Anindita Das
Gobardhan Das
Gokul Das
Surajit Das
Kaberi Dasgupta
Suryasarathi Dasgupta
Chandravanu Dash
Saeed Dastgiri
Prasun Datta
Sibnarayan Datta
Alessandro Datti
Jean Daunizeau
Sabato D'Auria
Alessandro D'Ausilio
Christopher Davey

Andrew Davies
Wayne Davies
C. Todd Davis
Dana Davis
Ian Davis
Keith Davis
Sarah Davis
Roberta Davoli
Thomas Dawson
Abhijit De
Alexandre de Brevern
Robertus de Bruin
Fernando de Castro
Gabriel de Erausquin
Jan de Fockert
Vittorio de Franciscis
Anne De Groot
Wouter de Herder
Alberto de la Fuente
Juan C. de la Torre
Floris de Lange
Herminia de Lencastre
Marc de Lussanet
Paulo De Marco Júnior
Ramon de Mello
Josué de Moraes
Gonzalo de Polavieja
Valli De Re
Salvatore De Rosa
Jerome de Seze
Bert De Smedt
Ive De Smet
Giuseppe De Socio
Russell de Souza
Harriet de Wit
Deborah Dean
Wendy Dean
Margaret DeAngelis
Peter Dearden
Sumitra Deb
Waldemar Debinski
Chitrita DebRoy
Maite deCastro
Stéphane Declerck
Yuriy Dedkov
Gagan Deep
Vadim Degtyar
Faramarz Dehghani
Sharon Dekel
Olaf Dekkers
Juan del Alamo
Filippo Del Bene
Maurizio Del Poeta
Charles Dela Cruz
Mária Deli
Odir Dellagostin
Persio Dello Sbarba
Dominique Delmas
Giovanni Delogu
Anna Delprato
Neal DeLuca
Vincenzo DeLuca
Sylvain Delzon
Anneke den Hollander
Dajun Deng
Hao Deng
Yong Deng
Z. Daniel Deng
Zhaohong Deng
Patricia Denning
Vida Dennis
Alok Deoraj
Cynthia Derdeyn
Tobias Derfuss
Kebede Deribe
Kathryn DeRiemer
Bart Dermaut
Gemma Derrick
Sébastien Descamps
Robert Deschenes
Abhishek Deshpande
Nicolas Desneux
Maurizio D'Esposito
Jean-Luc Desseyn
Mickaël Desvaux
Robert Dettman
Patrizia d'Ettorre
Eric Deutsch
Joseph Devaney
Timothy Devarenne
Shamala Devi Sekaran
Joanne Devlin
Andrew DeWan
Saikat Dewanjee
Sharon DeWitte
Charlene Dezzutti
Sangeeta Dhaubhadel
Keertan Dheda
Marc Dhenain
Navneet Dhillon
Amit Dhingra
Yuanpu Peter Di
Ferdinando Di Cunto
Simone Di Giovanni
Javier Di Noia
Giovanni Di Pasquale
Giuseppe di Pellegrino
Francesco Di Russo
João Miguel Dias
Emmanuel Dias-Neto

Bruno Diaz
Guillermo Diaz-Pulido
Gabriel Dichter
Frederic Dick
Joseph Dickens
Clayton Dickson
Francesco Dieli
David Diemert
Binh Diep
Paul Digard
Kottarappat Dileepan
Patrizio Dimitri
Dimiter Dimitrov
George Dimopoulos
Maya Dimova Lambreva
Aijun Ding
Lei Ding
Qiang Ding
Wenjun Ding
Tzvetanka Dinkova
Joseph DiStefano III
Thomas Dittmar
Kimon Divaris
Oleg Dmitriev
Kim Do
Ulrich Dobrindt
Renwick Dobson
Aristides Docoslis
Peter Dodson
Christopher Doering
Sam Doesburg
Junsang Doh
Hideyuki Doi
Riccardo Dolcetti
Juan Dominguez
Katharina Domschke
Bruce Donald
Julie Donaldson
Tobias Donath
Qunfeng Dong
Yufeng Dong
Maureen Donlin
Joaquin Dopazo
Alex M. Dopico
Christopher Doran
Robin Dore
Thomas Dorner
Pablo Dorta-González
Daniel Doucet
Michael Douglas
Constantine Dovrolis
David Dowdy
Bogdan Draganski
Shashank Dravid
Joël Drevet
Liam Drew
Steven Drews
Adam Driks
Petros Drineas
Don Driscoll
Paul Driscoll
David Dryden
Stuart Dryer
Chenyu Du
Lanying Du
Quansheng Du
Shao Du
Wen-Bo Du
Tao (Tony) Duan
Wenjie Duan
Zhenfeng Duan
Marie-Pierre Dubé
Ludwig Dubois
James Duce
John Duda
Anette Duensing
N.S. Duesbery
Jeremy Duffield
Jozef Dulak
J. Stephen Dumler
Jacques Dumont
Anna Dunaevsky
Gary Dunbar
Melinda Duncan
Steven Duncan
Sébastien Duperron
Daniel Duplisea
Denis Dupuy
William Durante
Caroline Durif
Daniel Durstewitz
Jean-Claude Dussaule
Bas Dutilh
Amit Dutt
Adrian Dyer
Simon Dymond
Petras Dzeja
Valsamma Eapen
Conrad Earnest
Connie Eaves
Ivano Eberini
Leo Eberl
Matthias Eberl
Kristie Ebi
Klaus Ebmeier
Mansour Ebrahimi
Esmaeil Ebrahimie
Miriam Echevarría
Juergen Eckel
Richard Eckert

Tobias Eckle
David Eddington
Mariola Edelmann
Andreas Eder
Luis Eduardo Quintas
Claire Edwards
Owain Edwards
Philip Efron
Christophe Egles
Oliver Eickelberg
Henrik Einwächter
Leonard Eisenberg
Peter Eklöv
Joseph El Khoury
Sam Eldabe
James Elder
Wael El-Deredy
Mounya Elhilali
M Carolina Elias
Kathrin Eller
Nathan Ellis
Osman El-Maarri
Stefan Elmer
Mohammed Elsalanty
Hany El-Shemy
Holger Eltzschig
Costanza Emanueli
Frank Emmert-Streib
Nicole Endlich
Antje Engelhardt
Jacob Engelmann
Adam Engler
Christoph Englert
Omolola Eniola-Adefeso
Eng Eong Ooi
Fikret Er
Danilo Ercolini
Isil Ergin
Kimmo Eriksson
Bard Ermentrout
Ingemar Ernberg
Alejandro Escobar-Gutiérrez
Hector Escriva
MariaPaz Espinosa
Alejandro Espinoza Orías
L. Michel Espinoza-Fonseca
Alessandro Esposito
Susanna Esposito
Francisco J. Esteban
William Etges
Nima Etminan
Eliseo Eugenin
Alistair Evans
Nir Eynon
Hiroshi Ezura
M. Faadiel Essop
Antonio Facchiano
Andreas Fahlman
Yiu Fai Tsang
Cecile Fairhead
Syed Faisal
Catherine Faivre-Sarrailh
Patrizia Falabella
Marco Falasca
Barbara Fam
Guo-Chang Fan
Xiaobing Fan
Yong Fan
Daniele Fanelli
David Fang
Deyu Fang
Shenying Fang
Eric Faragher
David Fardo
Andrew Farke
Alfonso Fasano
S. Hossein Fatemi
Dimitris Fatouros
Guido Favia
Herman Favoreel
Stefano Federici
Maurizio Federico
Talitha Feenstra
Carol Feghali-Bostwick
Michael Fehlings
Heinz Fehrenbach
Peiwen Fei
Kim Felmingham
Frank Feltus
Wenke Feng
Ying-Mei Feng
Youjun Feng
Yue Feng
Brock Fenton
Robert Fenton
Eric Feraille
Jorge Fernandes
Miguel Fernandez
José Fernández Robledo
Delmiro Fernandez-Reyes
Martin Fernandez-Zapico
Rashida Ferrand
Nicola Ferri
Raffaele Ferri
Sebastian Ferse
Marco Festa-Bianchet
Joerg Fettke
Maria Fiammetta Romano
Matthew Fidelibus
Edda Fiebiger

Ruth Filik
Lionel Filion
Scott Filler
Stephanie Filleur
Helen Fillmore
Michael Fine
Paul Fine
Bernhard Fink
David Finkelstein
Anthony Fiorillo
Paolo Fiorina
Gabriele Fischer
Uwe Fischer
Gordon Fisher
Matthew Fisher
Marco Fisichella
Gilberto Fisone
Patricia Fitzgerald-Bocarsly
David Flaspohler
Johannes Fleckenstein
Suzanne Fleiszig
Sheila Fleming
Erica Fletcher
Andres Floto
Guglielmo Foffani
Franco Folli
Daniel Foltz
Peter Fong
Stephen Fong
Diego Fontaneto
Nathan Ford
Christiane Forestier
Gianluigi Forloni
Albert Fornace Jr.
Naomi Forrester
Robert Forster
Thomas Forsthuber
John Forsythe
Patrice Fort
Anny Fortin
Angel Foster
Dimitrios Fotiadis
Ron Fouchier
Nicholas Foulkes
Hayley Fowler
Matthew Fox
Michael Fox
Leonardo Fraceto
Diego Fraidenraich
Pier Francesco Ferrari
Heather Francis
Renato Franco
Rodrigo Franco
Nikolaos Frangogiannis
Ingmar Franken
Alexander Franz
Andrea Franzetti
Martin Frasch
Pina Fratamico
Franca Fraternali
Giacomo Frati
David Frayer
David Fredricks
Jonathan Freedman
James Freeman
Alexander Freiberg
Michael Freitag
Nancy Freitag
Antonio Freitas
Kathleen Freson
Brigitte Frey
Oliver Frey
Esteban Fridman
Iddo Friedberg
Tim Friede
Doron Friedman
Matt Friedman
Peter Friedman
Alex Friedrich
Teresa Frisan
Friedrich Frischknecht
Laura Frishman
Jörg Fritz
Holger Fröhlich
Binying Fu
Jian Fu
Kai Fu
Flávio Fuchs
Sebastien Fuchs
Juan Fuentes
Mariana Fuentes
Sebastian Fugmann
Jong-Ling Fuh
Barbara Fuhrman
Hodaka Fujii
Robert Fujinami
Tohru Fukai
Tadashi Fukami
Yoshihiro Fukumoto
Christopher Fulton
Jennifer Fung
Roberto Furlan
Clemens Fürnsinn
Ivan Fuss
Bernard Futscher
Sandra Gabelli
Nandor Gabor Than
Attila Gacser
Sudhindra Gadagkar
Alain-Pierre Gadeau

Silvana Gaetani
Carlo Gaetano
Amit Gaggar
Joel Gagnier
Jean-Luc Gaiarsa
Georgios Gakis
Paul Galardy
Massimiliano Galdiero
Philippe Gallay
Alvaro Galli
Imed Gallouzi
Andrew Gallup
Ferenc Gallyas Jr.
Sanjiv Gambhir
Karen Gamble
Siew Gan
Yang Gan
Thumballi Ganapathi
Esteban Gándara
A Ganesan
Xu Gang Xia
Giuseppe Gangarossa
Nupur Gangopadhyay
Roman Ganta
Soren Gantt
Allen Gao
Chang-Qing Gao
Feng Gao
Hongwei Gao
Jian-Xin Gao
Lei Gao
Qian Gao
Shou-Jiang Gao
Xin Gao
Yulin Gao
Zhong-Ke Gao
Stavros Garantziotis
Nuria Garatachea
Silvio Garattini
Oscar García
Jose Manuel Garcia Aznar
Pablo Garcia de Frutos
Carlos Garcia de Leaniz
Aurora García-Gallego
José-María García-García
J. Gerardo García-Lerma
Rafael Garcia-Mata
Jordi Garcia-Ojalvo
Steven Gard
David Gardner
Pradeep Garg
Pere Garriga
Andrei Gartel
Stephane Gasman
Mauro Gasparini
Maria Gasset
Michelle Gatton
Nick Gay
Stefan Gebhardt
Thomas Gebhardt
Andrew Geers
Fabrizio Gelain
Mathias Gelderblom
Juri Gelovani
Marco Gemma
Kristina Gemzell-Danielsson
Daniele Generali
Damian Genetos
David Gent
Valérie Geoffroy
Irene Georgakoudi
Nikolaos Georgantzis
Anthony George
Joseph George
Sarah George
André Gerber
Armin Gerger
Roman Gerlach
Stephane Germain
Matthew Germino
Hernâni Gerós
Edward Gershburg
Murad Ghanim
Saeid Ghavami
Pietro Ghezzi
Anuja Ghorpade
Debashis Ghosh
Jagadananda Ghosh
Kalpana Ghoshal
Cynthia Gibas
Yann Gibert
Spencer Gibson
Mark Gijzen
Sam Gilbert
Giorgio Gilestro
Kenneth Gilhuijs
Andrew Gill
Matthew Gill
David Gillikin
Thomas Gillingwater
Fiona Gillison
Stephen Gilman
Edward Giniger
Stephen Ginsberg
Francesco Giorgino
Olivier Gires
Alessandro Giuffrè
Michele Giugliano
Amy Gladfelter
Wolfgang Glanzel

Stefan Glasauer
Norbert Gleicher
John Glendinning
Robert Glinwood
John Glod
Joseph Glorioso
Christian Gluud
M. Glymour
Sharon Glynn
Christopher Gobler
Ajay Goel
Stefano Goffredo
Anna Goldberg
Aaron Golden
Shira Goldenberg
Ellen Goldman
Gustavo Goldman
Bob Goldstein
Edward Goldstein
Delia Goletti
Aldrin Gomes
Newton Gomes
Hector Gomez
Sergio Gómez
Diego Gomez-Casati
Jesus Gomez-Gardenes
Moncho Gomez-Gesteira
Lourdes Gómez-Gómez
Dusan Gomory
Raquel Goncalves
Cheng-Xin Gong
Gaolang Gong
Qiyong Gong
Yan Gong
Zhiyuan Gong
Pedro Gonzalez
Germán González
Pedro Gonzalez-Alegre
Jose Gonzalez-Andujar
Concepción Gonzalez-Bello
Antonio Gonzalez-Bulnes
José González-Méijome
John Goodrich
Subash Gopinath
Olga Gorlova
Elena Gorokhova
Nandu Goswami
Yoav Gothilf
Sven Gould
Sebastien Gourbiere
Ajit Govind
Alessandro Gozzi
Alena Grabowski
Luis Graca
Jordi Gracia-Sancho
Paul Graham
Susan Graham
Jessica Grahn
Sander Granneman
Giovanni Grasso
Brian Gratwicke
Clive Gray
Ronald Gray
Maria Grazia Giansanti
Magdalena Grce
Jason Grebely
Andy Green
Colin Green
John Green
Stefan Green
Mark Greenlee
Elisa Greggio
Luisa Gregori
Aric Gregson
Pierre Gressens
Paul Gribble
Andrew Grierson
Ulla Griffiths
Michael Grigg
Ramon Grima
Anatoly Grishin
Sergei Grivennikov
Laurent Groc
A.B. Johan Groeneveld
Vince Grolmusz
Wulfila Gronenberg
Stan Gronthos
Rita Grosch
Michela Grosso
Tudor Groza
Jean Gruenberg
Tilman Grune
Niklaus Grunwald
Salvatore Gruttadauria
Alexei Gruverman
Oreste Gualillo
Camillo Gualtieri
Le Guan
Xin-Yuan Guan
Teja Guda
Agustín Guerrero-Hernandez
Fernando Guerrero-Romero
Dominique Guerrot
Nuri Gueven
Gaël Guilhem
Gilles Guillemin
Hervé Guillou
Laszlo Gulacsi
Maria Gulinello
Donald Gullberg

Anthony Guo
Haitao Guo
Jun-Tao Guo
Kun Guo
Nancy Guo
Wenge Guo
Xuejiang Guo
Yiru Guo
Dinesh Gupta
Ravindra Gupta
Sudeep Gupta
Sudhiranjan Gupta
Vijai Gupta
Vineet Gupta
Robert Guralnick
Roi Gurka
Attila Gursoy
José Gutiérrez
Julie Gutman
Nikolas Haass
Georg Häcker
Lilach Hadany
Jeffrey Haddad
Nouchine Hadjikhani
Leontios Hadjileontiadis
Christoph Hagemeyer
Christian Hagen
Bumsuk Hahm
Cecil Hahn
Sinuhe Hahn
Benjamin Haibe-Kains
Neena Haider
Mehrdad Hajibabaei
Ramin Hakami
Mohamed Hakimi
Leona Hakkaart
Siân Halcrow
William Halford
Rebecca Hall
Ruth Hall
Kay Hamacher
Björn Hamberger
Michael Hamblin
Frederick Hamel
Michelle Hampson
Arum Han
Gang Han
Guoqi Han
Kap-Hoon Han
Renzhi Han
Weiqing Han
Xiaonan Han
Yiping Han
Yuepeng Han
Zhaozhong Han
John Hancock
Lynn Hancock
Ronald Hancock
David Handelsman
Marc Hanewinkel
bo hang
Immo Hansen
Peter Hansen
Jürgen Harder
Tilmann Harder
Karen Hardy
Joshua Hare
Jaroslaw Harezlak
Edward Harhaj
Leila Harhaus
Pirkko Härkönen
Frank Harmon
Ilan Harpaz-Rotem
Diane Harper
David Harrich
Paul Harrigan
Fiona Harris
Jeffrey Harrison
Kevin Harrod
Anne Hart
John Hart
Dominik Hartl
Lisa Hartling
M. Elizabeth Hartnett
James Hartsfield
Kevan Hartshorn
Rudy Hartskeerl
Brandon Harvey
Eric Harvill
Kim Hasenkrug
Kenji Hashimoto
Ryota Hashimoto
Takanori Hashimoto
Seyed Hasnain
Imtaiyaz Hassan
Quazi Hassan
Mark Hatherill
Christos Hatzis
Martine Hausberger
Richard Haverkamp
Shannon Hawkins
John Hawks
Dror Hawlena
Phillipa J. Hay
Satoru Hayasaka
Naoyuki Hayashi
Johannes Haybaeck
Finbarr Hayes
Shawn Hayley
Graeme Hays

Elliott Hazen
Alain Haziot
Bin He
Chunyan He
Guangyuan He
Huiguang He
Johnny He
Na He
Ruihua He
Weijing He
Xiaoming He
Ya-Wen He
Yuxian He
Zhili He
Joshua Heazlewood
Andrew Hector
Man Hee Rhee
Christopher Heeschen
Nagendra Hegde
Berthold Heinze
Andreas Hejnol
James Hejtmancik
Samuli Helle
Stephane Helleringer
Manuela Helmer-Citterich
Fred Helmstetter
Charlotte Hemelrijk
Jan Hemmi
Susanne Hempel
Linda Hendershot
Michael Hendricks
Peter Hendricks
Jeroen Hendrikse
Petr Heneberg
Michael Hensel
David Henshall
Yann Herault
Christian Herder
Karl Herholz
Christophe Herman
Carlos Hermenegildo
Marcelo Hermes-Lima
Pilar Hernandez
Alejandro Raul Hernandez Montoya
Tina Hernandez-Boussard
Enrique Hernandez-Lemus
Martin Herold
Alfredo Herrera-Estrella
Jose Ignacio Herrero
Jean Louis Herrmann
Michael Herzog
Wolfgang Hess
Michal Hetman
Judi Hewitt
Dominique Heymann
César Hidalgo
Jan Hiddink
Dennis Higgs
Kristiina Hildén
Elisabeth Hildt
Andrew Hill
Philip Hill
Edna Hillmann
Peter Hills
Robert Hills
Karen Hind
Dariush Hinderberger
Kensuke Hirasawa
Emilio Hirsch
Peter Hitchcock
Anita Hjelmeland
Ja-an Ho
Jacqueline Ho
Mitchell Ho
Paulo Ho
Simon Ho
Wenzhe Ho
Yuan-Soon Ho
Sung Ho Ha
Mojgan Hodaie
Matt Hodgkinson
Robert Hoffman
Andreas-Claudius Hoffmann
Heather Hoffmann
Andreas Hofmann
Thomas Hofmann
Michael Hofreiter
Simon Hogan
Philip Hogarth
Peter Hohenstein
Petter Holme
David Holowka
Christian Holscher
Judith Homberg
Saw-See Hong
Xiao-Yue Hong
Yiguo Hong
Jane Hoppin
Mohammad Hoque
Elvira Hörandl
Andrew Horne
Stacy Horner
Arie Horowitz
Malcolm Horsburgh
Marc Horwitz
Yoko Hoshi
Yujin Hoshida
Yoshihiko Hoshino
Toru Hosoda
Khaled Hossain

Neil Hotchin
Bing Hou
Chenping Hou
Ling Hou
Andreas Houben
Nicolas Houlie
Eugene Houseman
Daniel Houser
Jon Houtman
Thomas Howdieshell
Piers Howe
Peter Howell
Daniela Hozbor
Marta Hribal
Chuhsing Kate Hsiao
Janet Hsiao
Chih-hao Hsieh
Fushing Hsieh
Hsi-Lung Hsieh
Patrick Hsieh
Yi-Hsien Hsieh
Ying-Hen Hsieh
Ann Hsing
Cheng Hu
Chin-Kun Hu
Dewen Hu
Jiani Hu
Shuijin Hu
Valerie Hu
Wenhui Hu
Xiaosong Hu
Yi Hu
Chun-Hsi Huang
Hao Huang
Jee-Fu Huang
Jinhai Huang
Li-Min Huang
Qingyang Huang
Shuang-Quan Huang
Wendong Huang
Xuhui Huang
Yhu-Chering Huang
Yi-Hsiang Huang
Yu Huang
Leng Huat Foo
Timothy Hubbard
Victor Huber
Dorothee Huchon
Barry Hudson
Matthew Hudson
Michael Huen
Rodrigo Huerta-Quintanilla
François Hug
William Hughes
Dafeng Hui
J. Hull
Maj Hulten
Jean-François Humbert
Sandrine Humbert
Stuart Humphries
Thomas Hund
Kwok Hung Chan
Enamul Huq
Peter Hurlin
Robert Hurst
Salik Hussain
Nguyen Huy
Snehalata Huzurbazar
Jiang-Shiou Hwang
Shang-Jyh Hwang
Sheng-Ping Hwang
David Hyrenbach
Marco Iacoboni
Adrianna Ianora
A. Ibekwe
Andrea Icks
Marco Idzko
Koichi Iijima
Kazutaka Ikeda
Yasuhiro Ikeda
Tetsuro Ikegami
Hee-Jeong Im Sampen
Ryozo Imai
Axel Imhof
Carmen Infante-Duarte
Hanne Ingmer
Thomas Ioerger
Nigel Irwin
Jennifer Isaacs
Richard Isaacson
Petros Isaakidis
Yoshitaka Isaka
Mark Isalan
Carlos Isales
Akira Ishihama
Ryouhei Ishii
Yoshiro Ishimaru
Fakir Islam
Saeed Islam
Hiroyuki Itabe
Etsuro Ito
Kazuhiro Ito
Miren Iturriza-Gómara
Yury Ivanenko
Zoran Ivanovic
Juraj Ivanyi
Kazuya Iwamoto
Andrew Iwaniuk
Jose Izarzugaza
Angelo Izzo

Monica Jablonski
Catherine Jackson
Daniel Jackson
Mark Jackson
Jon Jacobs
Ilse Jacobsen
Steven Jacobson
Lutz Jaencke
Ganesh Jagetia
Mukesh Jain
Pankaj Jaiswal
Leighton James
Eric Jan
Yih-Kuen Jan
Nihar Jana
Karin Jandeleit-Dahm
Andreas Janecke
Damir Janigro
Axel Janke
Paul Janssen
John Jefferies
Nathan Jeffery
Mika Jekabsons
Wolfgang Jelkmann
Albert Jeltsch
Clinton Jenkins
Barbara Jennings
Jong-Seong Jeon
Jae-Wook Jeong
Ruth Jepson
Kandiah Jeyaseelan
Samithamby Jeyaseelan
Vivekanand Jha
Vishal Jhanji
Ravi Jhaveri
Thomas Jhou
Hong-Long (James) Ji
Rongrong Ji
Zhanjun Jia
Jian Jian Li
Bin Jiang
Bing-Hua Jiang
Luo-Luo Jiang
Mingxi Jiang
Quan Jiang
Shibo Jiang
Yongshuai Jiang
Zhigang Jiang
Susana Jiménez-Murcia
Dong-Yan Jin
Xia Jin
Zhen Jin
Jian Jing
Dong-Gyu Jo
Ulrich Joger
Ludger Johannes
Darcy Johannsen
Neil Johannsen
Blake Johnson
Christopher Johnson
Colin Johnson
Erik Johnson
Norman Johnson
Stephen Johnson
Welkin Johnson
Jaap Joles
Alice Jones
D. Dafydd Jones
John Jones
Julia Jones
Kelvin Jones
Therésa Jones
Henrik Jönsson
Choun-Ki Joo
Fiona Jordan
I. King Jordan
Marie Jose Goumans
Shijo Joseph
David Jourd'heuil
Xian Jun Loh
Rex Jung
Juan Luis Jurat-Fuentes
Lars Kaderali
Deborah Kado
Philipp Kahle
Chikara Kaito
Poonam Kakkar
Panagiotis Kalaitzis
Ruslan Kalendar
Vipin Kalia
Tanya Kalin
Vladimir Kalinichenko
Hooman Kamel
Jason Kamilar
Juliane Kaminski
Harm Kampinga
Jaap Kamps
Akio Kanai
Tatsuo Kanda
Osamu Kaneko
Jean Kanellopoulos
Colette Kanellopoulos-Langevin
Hojeong Kang
Rui Kang
Sang-Moo Kang
Madhuri Kango-Singh
Natarajan Kannan
Chryso Kanthou
Jyotshna Kanungo
Makoto Kanzaki

Chung-Lan Kao
Katy Kao
Maria Kaparakis-Liaskos
Amit Kapoor
Zoi Kapoula
Claudia Kappen
Sophia Karagiannis
Petros Karakousis
Nikos Karamanos
Dimitrios Karamichos
Vardan Karamyan
Goran Karapetrov
Efstathios Karathanasis
Katrin Karbstein
Jörn Karhausen
Petr Karlovsky
Morris Karmazyn
Sadashiva Karnik
Susanne Kaser
Fatah Kashanchi
Khalil Kashkush
Jan Kassubek
Rajesh Katare
Tadafumi Kato
Takuma Kato
Masaru Katoh
Dan Kaufman
Jordy Kaufman
Gunnar Kaufmann
Rupert Kaul
Deepak Kaushal
Alexandra Kavushansky
Namik Kaya
Manfred Kayser
Lawrence Kazembe
Damien Keating
Chun Kee Chung
Kylene Kehn-Hall
Andreas Keil
Jennifer Keiser
Ian Kellar
Miklos Kellermayer
Lisa Kellman
Christina Kellogg
Ben Kelly
Gregory Kelly
John Kelly
Rachel Kendal
Debra Kendall
Kok Keng Tee
Stefan Kepinski
Jake Kerby
Irina Kerkis
Chandrasekharan Kesavachandran
Ozlem Keskin
Hans Kestler
Alexandra Key
Brian Key
Sung Key Jang
Salomeh Keyhani
Andre Khalil
Mohammad Khamseh
Asad Khan
Gulfaraz Khan
Wasif Khan
Raya Khanin
Hemant Khanna
Rohit Khanna
Mohamed Khayyal
Reza Khodarahmi
Yury Khudyakov
Muhammad Khurram Khan
Zoha Kibar
Stefan Kiechl
Steffen Kiel
Alexandre Kihara
Arianna Kim
Beom Seok Kim
Chang Kim
Do Young Kim
Hoguen Kim
Hongkyun Kim
Jayoung Kim
Jeong-Ho Kim
Jonghan Kim
Jung-Eun Kim
Jung-Woong Kim
Kyoung Heon Kim
Samuel Kim
Seung Up Kim
Yoon Ki Kim
Tjeerd Kimman
Akinori Kimura
Anthony Kincaid
Sonja Kinner
John Kirby
Rudolf Kirchmair
Martyn Kirk
Karla Kirkegaard
Apar Kishor Ganti
Patricia Kissinger
Marian Kjellevold
Robyn Klein
Christoph Kleinschnitz
Dimitris Kletsas
Athol Klieve
A. Peter Klimley
Ned Klopfenstein
Anita Kloss-Brandstätter
Jan Kluytmans

Michael Klymkowsky
Michael Knapp
Olaf Kniemeyer
Matty Knight
Brian Knoll
Laura Knoll
Jason Knott
Erik Knudsen
Dennis Ko
Bostjan Kobe
Firas Kobeissy
Karl-Wilhelm Koch
Kersten Koelsch
Deanna Koepp
Devin Koestler
Tsuyoshi Koide
Kin-Hang Kok
Yoshihiro Kokubo
Frank Kolligs
Tobias Kollmann
Sergios-Orestis Kolokotronis
Motohiro Komaki
Natalia Komarova
Yulia Komarova
Masaaki Komatsu
Melanie Königshoff
Marina Konopleva
Dimitris Kontoyiannis
John Koomen
Murray Korc
Michael Kormann
Sergey Korolev
Maya Koronyo-Hamaoui
Wolfgang Köster
Konstantinos Kostikas
Erika Kothe
Sonja Kotz
Hari Koul
Katerina Kourentzi
Michael Koval
Susan Kovats
Christine Kozak
Gerald Kozlowski
Roberto Kraenkel
Ralf Krahe
Florian Krammer
Anna Kramvis
Holger Krapp
Susanne Krauss-Etschmann
Eric Kremer
Laurent Kremer
Laurent Kreplak
Mariska Kret
Jens Kreth
Doris Kretzschmar
Jeffrey Krichmar
Andreas Krieg
Mohana Krishna Mudiam
Koyamangalath Krishnan
Viswanathan Krishnan
Darren Kriticos
Evelyn Kroesbergen
Andrew Kroll
Florian Kronenberg
Juergen Kropp
Rebecca Krukowski
Claude Krummenacher
M. Fabiana Kubke
Wilfried Kues
Thomas Kufer
Jens Kuhn
Oscar Kuipers
Helena Kuivaniemi
Rakesh Kukreja
Rohit Kulkarni
Pawan Kulwal
Anil Kumar
Ashok Kumar
Lalit Kumar
Nirbhay Kumar
Sanjai Kumar
Sanjay Kumar
Saravana Kumar
Shashi Kumar
Sunil Kumar Manna
Navnith Kumaran
Chandan Kumar-Sinha
Tsutomu Kume
Matjaž Kuntner
Gotthard Kunze
Chih-Horng Kuo
Ho-Chang Kuo
Kornelius Kupczik
Gary Kupfer
Eiko Kuramae
Inari Kursula
Petri Kursula
Masataka Kuwana
Michael Kyba
Natasha Kyprianou
Minna-Maarit Kytöviita
Jeum Kyu Hong
Massimo Labra
Nathalie Labrecque
Jean-Marc Lacape
Ezio Laconi
Shannon LaDeau
Denis Lafontaine
Robert Lafrenie
Tim Lahm

Martina Lahmann
Garet Lahvis
Erh-Min Lai
Hsin-Chih Lai
Jui-Yang Lai
Liangxue Lai
Katariina Laine
Majlinda Lako
Jerson Laks
Madepalli Lakshmana
Hon-Ming Lam
Wilbur Lam
Javier Lama
Christophe Lamaze
Eric Lamb
Cornelis Lambalk
Sergio Lambertucci
Renaud Lambiotte
Claus Lamm
Mikko Lammi
Severine Lamon
Ke Lan
Ruth Landau
Alan Landay
Giovanni Landoni
Nicoletta Landsberger
Julia Lane
Roland Lang
Scott Langevin
Berthold Langguth
Thomas Langmann
Jörg Langowski
Gordon Langsley
Lucia Languino
Humberto Lanz-Mendoza
Markus Lappe
Natalia Lapteva
Alexander Larcombe
Banafshe Larijani
Vincent Larivière
Clark Larsen
Peter Larsen
Charles Larson
Corinne Lasmezas
Joerg Latus
Philipp Latzin
Robert Latzman
Andy Lau
Wan Yee Lau
Vincent Laudet
Jörn Lausen
Derek Laver
Pascal Lavoie
Matthew Law
Keith Laws
Victoria Lawson
Craig Layman
Claudio Lazzari
Chiara Lazzeri
Weidong Le
Bernard Le Foll
Roger Le Grand
Herve Le Stunff
Walter Leal
Mikhail Lebedev
Nikolai Lebedev
Irina Lebedeva
Binnaz Leblebicioglu
Mark LeDoux
Bok-Luel Lee
Chon-Lin Lee
Hyun-Sung Lee
Ji-Hyun Lee
Joohyung Lee
Jung Eun Lee
Jung Ryeol Lee
Jung Weon Lee
Ju-Seog Lee
Kyung Lee
Leo Lee
Myon-Hee Lee
Sean Lee
Seok-Geun Lee
Seon-Woo Lee
Seungbok Lee
Shing Lee
Terence Lee
Yin-Won Lee
Young-Min Lee
Yungling Lee
Alexander Leemans
Jason Lees
Markos Leggas
R. Lehman
Sune Lehmann
Hans-Joachim Lehmler
Benfang Lei
Saobo Lei
David Leib
Nic Leipzig
Luciana Leite
Maria Leite-de-Moraes
José Lemos
Megan Lenardon
Fenfei Leng
Christophe Lenglet
Laurel Lenz
Tiziana Leone
Zoya Leonenko
Livia Leoni

Christopher Lepczyk
Christophe Leroyer
Andres Lescano
Gregory Lesinski
Edward Lesnefsky
Adam Lesner
Olivier Lespinet
Frederick Leung
Yuk Fai Leung
Sue Levkoff
David N Levy
Yaakov Levy
F. Markus Leweke
Alfred Lewin
Patrick Lewis
Joel Lexchin
Bo Li
Chengdao Li
Cheng–Sen Li
Dongmei Li
Jin-Tian Li
Jinyan Li
Jun Li
Kui Li
Lei Li
Mai-He Li
Maoteng Li
Mei Li
Qianjun Li
Qizhai Li
Shengxu Li
Songhai Li
Suxia Li
Tiansen Li
Tingting Li
Wan-Ju Li
Wei Li
Xia Li
Xiangzhen Li
Xiao-Jiang Li
Xiu-Qing Li
Yan Li
Yi Li
Yun Li
Yuqing Li
Zheng Li
Zhenyu Li
Ziyin Li
Zongjin Li
Giovanni Li Volti
Chen Liang
Cheng-Guang Liang
Eryuan Liang
Feng Liang
Peipeng Liang
Wenju Liang
Mingzhi Liao
Mathias Lichterfeld
Karen Lidzba
Stefan Liebner
Michael Lierz
Marc Liesa
Jonathan Lifshitz
David Lightfoot
Hyunjung Lim
Kah-Leong Lim
Viviane Lima
Federica Limana
Baochuan Lin
Ching-Po Lin
Fa-Hsuan Lin
Hai-Yan Lin
Han-Chieh Lin
Haotian Lin
Jinxing Lin
Senjie Lin
Wen-Xiong Lin
Wenyu Lin
Ying-Ju Lin
Zhicheng Carl Lin
Rafael Linden
Erjun Ling
Ralf Linker
Igor Linkov
Vincenzo Lionetti
Michael Lipinski
Frederique Lisacek
Sefer Lisesivdin
Damon Little
Bin Liu
Chen-Hua Liu
Chunming Liu
Guei-Sheung Liu
Hanjun Liu
Huafeng Liu
Jian Liu
Jian-Feng Liu
Ji-Hong Liu
Jinny Liu
Shan-Lu Liu
Xiaoan Liu
Xiaohua Liu
Xuefeng Liu
Yong Liu
Zheng Liu
Zhihua Liu
Zhong-Jian Liu
Alexander Ljubimov
Anthony Lo
Kwok-Wai Lo

Jean-Marc Lobaccaro
Alessio Lodola
David Loeb
Marie Lof
Gunnar Loh
Yuin-Han Loh
Steven Loiselle
Bruno Lomonte
Cheryl London
David Long
Travis Longcore
Matthew Longo
Russell Lonser
Juan Loor
Miguel López
Guillermo López Lluch
Miguel Lopez-Ferber
Cecilio López-Galíndez
Giancarlo López-Martínez
Carlos López-Vaamonde
Marcelo Lorenzo
Christopher Lortie
Lise Lotte Gluud
Stefan Lötters
Edward Louis
Matthieu Louis
Stelios Loukides
Denis Loustau
Jason Love
Connie Lovejoy
Christian Lovis
Sergi Lozano
Hua Lu
Lin Lu
Qing Lu
Rui Lu
Shan Lu
Sheng-Nan Lu
Tao Lu
Wang-jin Lu
Xin-Yun Lu
Zhong-Lin Lu
Gert Lubec
Yoel Lubell
Edralin Lucas
Paul Lucas
Paul Lucassen
Alejandro Lucía
Fabio Lucidi
Su Lui
John Luk
Lewis Lukens
Ken Lukowiak
Helge Lumbsch
Peter Lundberg
Oleg Lunov
Hong Luo
Jia Luo
Nan Luo
Wenbo Luo
Xi Luo
Yi Luo
Zhao-Qing Luo
Zhong-Cheng Luo
Riitta Luoto
Raul Luque
Arthur Lustig
Heike Lutermann
Esther Lutgens
Dawn Luthe
Aernout Luttun
Adrian Luty
Andrea Luvisi
Zhihan Lv
John Lynch
Francis Lynn
Lisa Lyons
Rick Lytel
Grant Lythe
William Lytton
Daqing Ma
Jianjie Ma
Jiyan Ma
Jun Ma
Wujun Ma
Xiaolei Ma
Xin-Liang Ma
Yina Ma
Zheng-Liang Ma
Stefan Maas
Andrea Macaluso
Ben MacArthur
Roberto Macchiarelli
Guy MacGowan
Ricardo Machado
Miguel Machuqueiro
Brian MacKenzie
Una Macleod
Paolo Madeddu
Michele Madigan
Norikazu Maeda
Shiro Maeda
Kathrin Maedler
Walter Maetzler
Giovanni Maga
Vanesa Magar
Frédérique Magdinier
Sanjay Maggirwar
Ramamurthy Mahalingam
Brion Maher

Toby Maher
Andrew Mahon
Eugene Major
Carl Maki
Makoto Makishima
Lucia Malaguarnera
Luc Malaval
Jesus Maldonado
Luis Maldonado Manjarrez
Rayaz Malik
Krishna Mallela
Bibekanand Mallick
Moises Mallo
Manuel Malmierca
Jesus Malo
Fabrizio Mammano
Kwan Man
Ichiro Manabe
Yukari Manabe
Emmanuel Manalo
Andrea Manca
Michael Mancini
Shekhar Mande
Mercedes Susan Mandell
Riccardo Manganelli
Balaji Manicassamy
Arto Mannermaa
Ben Mans
Ed Manser
Nicholas Mantis
Lorenzo Mantovani
Roberto Mantovani
Lamberto Manzoli
Jian-Hua Mao
Jingdong Mao
Tapio Mappes
Antonella Marangoni
Miguel Maravall
Christos Maravelias
Alessandro Marcello
Adriano Marchese
Franck Marchis
Alessandra Marengoni
Antoni Margalida
Rogerio Margis
Leonid Margolis
Daniel Margulies
Bernard Mari
Stefano Mariani
Christophe Mariat
Pierre Marie
Daniele Marinazzo
Claudio Marinho
Leonardo Mariño-Ramírez
Martin Marinus
Frederic Marion-Poll
Wanda Markotter
Gilles Marodon
Ernesto Marques
Andrew Marr
Vicki Marsh
Christopher Marshall
James Marshall
Lorna Marson
Fabio Martelli
Lennart Martens
Sally Martin
Stephen Martin
Federico Martinelli
Luis Martinez
Jose Angel Martinez Climent
Francisco Martinez-Abarca
Susana Martinez-Conde
Silvia Martínez-Llorens
Ligia Martins
Andrea Martinuzzi
Michael Massiah
Ramin Massoumi
Maria Masucci
Naoki Masuda
Juan Mata
Vikram Mathews
Timothy Matisziw
Jonathan Matsui
Minami Matsui
Takashi Matsui
Takuya Matsumoto
Hiroaki Matsunami
Keitaro Matsuo
Yutaka Matsuoka
Michiya Matsusaki
Sachiko Matsuzaki
Atsushi Matsuzawa
Joseph Mattapallil
Fabrizio Mattei
Mikhail Matz
Pallab Maulik
Natasha Maurits
Ulrich Maus
Clarissa Maya-Monteiro
Olga Mayans
Claudine Mayer
Alfredo Mayor
Sylvie Mazoyer
Rachid Mazroui
Marianna Mazza
Silvia Mazzuca
Grainne McAlonan
Brian McCabe
Joseph McCarty

James McCaw
Kevin McCluskey
Karen McComb
Cheryl McCormick
David McCormick
Jean McCrory
Scott McCue
James McCutcheon
Scott McDonald
Andrew McDowell
Sheena McGowan
Alistair McGregor
Melvin McInnis
David McKemy
Peter McKenna
Christopher McKindsey
Margaret McLaughlin-Drubin
Ian McLoughlin
Laoise McNamara
Paul McNeil
Jodie McVernon
James Meador
Andrea Mechelli
Warren H Meck
Stephan Meckel
Miguel Medina
Mónica Medina
Anand Mehta
Jodhbir Mehta
Kapil Mehta
Lin Mei
Benjamin Meier
Onno Meijer
Michael Meijler
Edgar Meinl
Ulrich Melcher
Stefano Meletti
Jaymie Meliker
Rossana Melo
Jose Melo-Cristino
Marco Meloni
Maeli Melotto
Frank Melzner
John Mendelson
Pablo Menendez
Liset Menendez de la Prida
Luis Menéndez-Arias
Gustavo Menezes
Xiang-Jin Meng
Zhefeng Meng
Guadalupe Mengod
Adina Merenlender
Peter Mergaert
William Merigan Jr.
Bruno Merk
Roeland Merks
Stefano Merler
Daniel Merrifield
Tesfaye Mersha
Frédéric Mertens
Marc Merx
Alex Mesoudi
Ilhem Messaoudi
John Metcalfe
Claudia Mettke-Hofmann
Konradin Metze
Dennis Metzger
Bernhard Metzler
Eliane Meurs
Sven Meuth
Craig Meyers
David Meyre
Eva Mezey
James Mezhir
Dengshun Miao
Pawel Michalak
Kristin Michel
Stephen Michnick
Claudia Miele
Antimo Migliaccio
Cathy Mihalopoulos
Matthew Mihlbachler
Takeshi Miki
Jeremy Miles
Peter Milgrom
Frederick Miller
Patrick Miller
Todd Miller
Francis Miller Jr.
P.J.M. Milligan
Ken Mills
David Milstone
Kyung-Jin Min
Xiang Min
Zhi Min Yang
Masabumi Minami
Tohru Minamino
Federico Mingozzi
John Minna
Jens Minnerup
Timothy Minogue
Eric Mintz
Barbara Mintzes
Monica Miozzo
Vincenzo Miragliotta
Harald Mischak
Dan Mishmar
Ramesh Mishra
Yogendra Mishra
Rajeev Misra

Fanis Missirlis
Nerges Mistry
Alex Mitchell
Caroline Mitchell
Rachel Mitchell
Nandita Mitra
Neena Mitter
Eliane Miyaji
Sayuri Miyamoto
Toshiyuki Miyata
Norikatsu Miyoshi
Emiko Mizoguchi
Nancy Mock
Mirjam Moerbeek
Mohammad Mofrad
Masaki Mogi
Jacqueline Mohan
Rajiv Mohan
Rajesh Mohanraj
Subhra Mohapatra
Pierre Moine
Christine Moissl-Eichinger
Igor Mokrousov
Juan Molinero
Bertrand Mollereau
Sassy Molyneux
Tapan Mondal
Mario Mondelli
Alexander Mongin
Valérie Mongrain
Daniel Monleon
Ali Montazeri
Courtney Montgomery
Dhayendre Moodley
Mari Moora
Anne Moore
Darren Moore
Spencer Moore
Ana Mora
Paula Morais
Maria Moran
Corrie Moreau
Francisco Moreira
Luciano Moreira
Nei Moreira
Edgardo Moreno
Eduardo Moreno
Silvia Moreno
Yamir Moreno
Gabriel Moreno-Hagelsieb
Gregorio Moreno-Rueda
Rafael Moreno-Sanchez
Claudio Moretti
José A. Morgado-Díaz
Keisuke Mori
Lisa Morici
Takaya Moriguchi
Ryuichi Morishita
Cedric Moro
Andrea Morrione
Alessio Mortelliti
Kevin Mortimer
J Bruce Morton
Rory Morty
Antonio Moschetta
George Mosialos
Csaba Moskát
R. Mosley
M.D. Motaleb
Michael Motes
Jean-Pierre Mothet
Justin Mott
Andrea Motta
Sophie Mouillet-Richard
Vincent Mouly
Catherine Mounier
André Mouraux
Laurent Mourot
Tim Mousseau
Jordi Moya-Larano
Rosa Moysés
Riccardo Mozzachiodi
Jorge Mpodozis
Tarek Msadek
Michael Muders
Daniel Mueller
Scott Mueller
Elliot Mufson
Ken-ichi Mukaisho
Amitava Mukherjee
Partha Mukhopadhyay
Mark Muldoon
Jennifer Mulle
Marc Muller
Ferenc Müller
Rolf Müller
Bertram Müller-Myhsok
Gabriele Multhoff
Srinivas Mummidi
Ulrike Munderloh
Rachata Muneepeerakul
Gnanasekar Munirathinam
Vincent Munster
Craig Murdoch
Eric Murillo-Rodriguez
William Murphy
Ashlesh Murthy
Antonio Musaro
Gokhan Mutlu
Ivan Nabi

Angel Nadal
Hossein Naderi-Manesh
Sathyamangla Naga Prasad
Yoshitaka Nagai
Ram Nagaraj
Takeshi Nagasaka
Michael Nagler
Julian Naglik
Brahim Nait-Oumesmar
Behzad Najafian
Joseph Najbauer
Takahisa Nakamura
Hiroyasu Nakano
Jin Nam
Sang-Chul Nam
Mahesh Narayan
Raja Narayanan
Raul Narciso Guedes
Marko Nardini
Rajesh Narendran
Fabio Nascimento
Marcelle Nascimento
Ramona Natacha Pena i Subirà
Serge Nataf
Kalimuthusamy Natarajaseenivasan
Urs Nater
Dhruba Naug
Alfons Navarro
Carlos Navas
Tim Nawrot
Daniel Naya
Ara Nazarian
Aamir Nazir
Lishomwa Ndhlovu
Dan Nebert
Brett Neilan
Robert Nerenberg
Pratibha Nerurkar
Luis Netto
Josef Neu
Stephan Neuhauss
Michael Nevels
Richard Newcomb
Peter Newman
William Newman
David Newth
Robert Newton
Olivier Neyrolles
Carl Ng
Lai Guan Ng
Lisa Ng
Quan Sing Ng
Hang Nguyen
Henry Nguyen
M. Hong Nguyen
Tuan Nguyen
Xijun Ni
Raymond Niaura
Joseph Nickels
Mark Nicol
Daotai Nie
Feiping Nie
Thomas Niederkrotenthaler
Conrad Nieduszynski
Randall Niedz
James Nieh
Kirsten Nielsen
Morten Nielsen
Heiner Niemann
William Nierman
Jérôme Nigou
Carlo Nike Bianchi
Dragana Nikitovic-Tzanakaki
Nikolas Nikolaidis
Georgios Nikolopoulos
Yi Ning
Wei Ning Chen
Daisuke Nishi
Noriyuki Nishida
Hisao Nishijo
Hiroyoshi Nishikawa
Stephen Nishimura
Wataru Nishimura
Hiroshi Nishiura
Aleksandra Nita-Lazar
Douglas Nixon
Gisela Nogales-Gadea
Antal Nógrádi
Abdisalan Noor
Mohd Noor Norhayati
Giuseppe Norata
Melissa Norberg
Antoine Nordez
Patrice Nordmann
Saima Noreen
David Norman
Christopher Norris
Francois Nosten
Giuseppe Novelli
Ariel Novoplansky
Paulina Nowicka
Minou Nowrousian
Ulrich Nübel
Rachel Nugent
Syam Nukavarapu
Olga Nunes
Dmitry Nurminsky
Michael Nurmohamed
Howard Nusbaum
Annunziata Nusca

Chukwumere Nwogu
George-John Nychas
Assad Oberai
Monika Oberer
Tatiana Oberyszyn
Alexander Obukhov
Bonnie O'Connor
Agricola Odoi
Patrick O'Grady
Olorunseun Ogunwobi
Andreas Ohlmann
Masuo Ohno
Yoshikazu Ohya
Naoki Oiso
David Ojcius
Toshiyuki Ojima
Mariko Okada (Hatakeyama)
Jason Okulicz
Taras Oleksyk
Baldo Oliva
Pedro Oliveira
Sergio Oliveira
Jake Olivier
Jeff Ollerton
Kenneth Olsen
Donald Olson
Michael Olson
I. Anna Olsson
Michal Olszewski
Abdelwahab Omri
Ali Önder Yildirim
Tolu Oni
N. Charlotte Onland-Moret
Don Operario
László Orbán
Covadonga Orejas
Matej Orešic
Joseph Orgel
Ludovic Orlando
Dennis O'Rourke
Sandra Orsulic
Pavel Ortinski
Robert Oshima
Daniel Osorio
Sonia Osorio-Algar
Henrik Oster
Henrik Österblom
Jan Ostermann
Oksana Ostroverkhova
Mario Ostrowski
Marisa Otegui
Motoyuki Otsuka
Geir Ottersen
Michael Otto
Toru Ouchi
Cees Oudejans
Michel Ouellette
Tim Oury
Christos Ouzounis
Ofer Ovadia
Cristina Óvilo
Gozde Ozakinci
Antonio Pacheco
Emanuele Paci
Eugenio Paci
Davide Pacini
Svetlana Pack
Jaya Padmanabhan
Parasuraman Padmanabhan
Johnny Padulo
Joseph Pagano
Madhukar Pai
Arnab Pain
Coro Paisan-Ruiz
Samuel Paiva
Soumitro Pal
Utpal Pal
Elisabetta Palagi
Senthilnathan Palaniyandi
Nades Palaniyar
Francesc Palau
Alexander Palazzo
Swati Palit Deb
Pierlorenzo Pallante
Komaraiah Palle
Subba Palli
Johan Pallud
Nicholette Palmer
Enzo Palombo
Arnar Palsson
An Pan
Chen-Wei Pan
Chongle Pan
Xiaoping Pan
Deeksha Pandey
Girdhar Pandey
Gunjan Pandey
Siyaram Pandey
Udai Pandey
Maharaj Pandit
John Panepinto
Alexander Panfilov
Xiaoming Pang
Aditya Pant
Kostas Pantopoulos
Akos Pap
Roberto Papa
Salvatore Papa
Gianpaolo Papaccio
Christos Papadelis

Nikos Papadopoulos
Elena Papaleo
Thalia Papayannopoulou
Tamás Papp
Francesco Pappalardo
Hanu Pappu
Dimitrios Paraskevis
P. Pardha-Saradhi
Daniel Paredes-Sabja
Swarup Parida
Nehal Parikh
Samir Parikh
Tanya Parish
Deric Park
Jong-In Park
Man-Seong Park
Paul Park
Emily Parker
Helena Parkington
John Parkinson
Aristeidis Parmakelis
Marie-Laure Parmentier
Alessandro Parolari
Maddy Parsons
Janet Partridge
Louis Pasquale
Alberto Passi
Claudio Passino
Geraldo Passos
Annalisa Pastore
Kevin Paterson
Santosh Patnaik
Jayadeep Patra
Heather Patterson
Tommy Pattij
Hemant K. Paudel
Annika Paukner
Friedemann Paul
Richard Paul
Ramasamy Paulmurugan
Patrick Paulus
Gianni Pavan
Martin Pavelka
Marina Pavlova
Jodi Pawluski
George Paxinos
William Paxton
Jorge Paz-Ferreiro
Valerio Pazienza
Cameron Peace
Eve-Isabelle Pecheur
Robert Pechnick
Myron Peck
Shyamal Peddada
Andrew Pekosz
Beatriz Pelacho
Matteo Pellegrini
Sergio Pellis
Leandro Peña
Elise Pendall
Gurudutt Pendyala
Zuogang Peng
Trevor Penney
Marzio Pennisi
Thomas Penzel
José Perales
Matjaz Perc
Manuel Perea
Inês Pereira
Luísa Pereira
Lygia Pereira
Marco Peresani
M.A. Pérez
Antonio Perez-Martinez
Eugene Permyakov
Carlo Federico Perno
Dragan Perovic
Mark Pershouse
Matthew Perugini
Lorenzo Peruzzi
Maurizio Pesce
Mathias Pessiglione
Hans-Ulrich Peter
Michael Peters
Jonathan Peterson
Karin Peterson
Odile Petit
Kathy Petoumenos
Michael Petraglia
Emanuel Petricoin
Pier Giorgio Petronini
Sarah Pett
Salvatore Petta
Sebastien Pfeffer
Brian Pfleger
Dzung Pham
Thanh Phan
Robert Phillips
Wayne Phillips
William Phillips
Andrew Philp
Maria Francesca Piacentini
Shilong Piao
Didier Picard
Mauro Picardo
Ciriaco Piccirillo
Raymond Pickles
Marcio Pie
Russell Pieper
Massimo Pietropaolo

Jakob Pietschnig
Giuseppe Pignataro
Jay Pillai
Ron Pinhasi
João Pinto
Gianfranco Pintus
Matteo Pirro
Alexander Pisarchik
Dario Pisignano
Juan Pizarro
Carmine Pizzi
Salvatore Pizzo
Josep Planas
Emmanuel Planel
Paul Planet
Michelina Plateroti
Michael Platten
Alan Pockley
Boris Podobnik
Stefanie Pöggeler
Stefan Pöhlmann
Irina Polejaeva
Guido Poli
Stephen Polyak
Michael Polymenis
Jean-François Pombert
Daniela Ponce
Giovanni Ponti
Mikhail Pooggin
Venuprasad Poojary
Art Poon
Michel Popoff
Alexey Porollo
Enzo Porrello
James Porter
Manuel Portero-Otin
Manuel Portolés
Maarten Postma
James Potash
Marie-Claude Potier
Benjamin Poulter
Nader Pouratian
Pedro Póvoa
Juan Poyatos
Gabriele Pradel
Roger Pradel
Sriharsa Pradhan
Gustavo Pradilla
Manoj Prasad
Antje Prasse
Stephen Pratt
Tobias Preis
Thomas Preiss
Louis Premkumar
Peter Prentis
Garrett Prestage
Matt Price
Nicholas Price
Theodore Price
Claude Prigent
Josef Priller
Shankar Prinja
Kathleen Pritchett-Corning
Ludmila Prokunina-Olsson
Vasilis Promponas
Roberto Pronzato
Paul Proost
Michael Proulx
Stephen Proulx
Nicholas Provart
Paolo Provero
Patrick Prunet
Reeta Prusty Rao
Jude Przyborski
Maurice Ptito
Read Pukkila-Worley
Hemant Purohit
Louise Purton
Nicholas Pyenson
Yong Pyo Lim
Krzysztof Pyrc
Qian Qian
Gangjian Qin
Gao-Feng Qiu
Jiang Qiu
Jianming Qiu
Xinghui Qiu
Fan Qu
Hui-Qi Qu
Caroline Quach
Federico Quaini
Zhe-Xue Quan
Antonietta Quigg
Petra Quillfeldt
Janet Quinn
Terence Quinn
Marcel Quint
Alexander Rabchevsky
Thierry Rabilloud
Craig Radford
Sima Rafati
Gajendra Raghava
Yvan Rahbé
Abidur Rahman
Mahfuzar Rahman
Pasquale Raia
Nigel Raine
Sari Räisänen
Gireesh Rajashekara
Johnson Rajasingh

Istvan Rajcan
Raghavan Raju
Zoltán Rakonczay Jr.
Stuart Raleigh
Shree Ram Singh
Sreeram Ramagopalan
Harsh Raman
Jose Javier Ramasco
Ramani Ramchandran
Pranela Rameshwar
Joe Ramos
Lennart Randau
Paul Randazzo
Shoba Ranganathan
A.L.N. Rao
Christopher Rao
Hengyi Rao
Zhonghao Rao
Cedric Raoul
Fabio Rapallo
Arash Rashed
David Rasko
Randall Rasmusson
Maria Rastaldi
John Ratcliffe
Adam Ratner
Magnus Rattray
Nadine Ravel
Ann Rawkins
John Rawls
Ranjit Ray
Ratna Ray
Imran Razzak
Francisco Real
Hugo Rebelo
Gianpaolo Reboldi
David Reby
Gadi Reddy
Hemachandra Reddy
Jay Reddy
Sakamuri V. Reddy
Aaron Reed
Judy Rees
R. Keith Reeves
Thomas Reh
Nicholas Reich
Andreas Reichert
Sean Reid
Andreas Reif
Manuel Reigosa
Gwendolen Reilly
Markus Reindl
David Reiner
Kurt Reinhart
Rui Reis
Johannes Reisert
Gernot Reishofer
Giuseppe Remuzzi
Lei Ren
Xuefeng Ren
Alvaro Rendon
Jyothi Rengarajan
Giuseppe Rengo
Laurent Rénia
Michel Renou
Gourapura Renukaradhya
Andre Renzaho
Stephen Resch
Sylvie Rétaux
Constantino Reyes-Aldasoro
Hamid Reza Baradaran
Human Rezaei
Jong Rho
Francesco Ria
Domenico Ribatti
Miria Ricchetti
Zaccaria Ricci
Andrew Rice
Yolande Richard
Kristy Richards
Bryce Richardson
Thomas Richie
William Ridgway
Dean Riechers
Christian Riedel
Frederic Rieux-Laucat
Howard Riezman
Paul Riggs
Bruce Riley
Riikka Rinnan
Arun Rishi
Praveen Rishi
Marco Rito-Palomares
Susan Rittling
Daniel Rittschof
Christian Rixen
Stacey Rizza
Seungil Ro
Stanley Robboy
David Roberts
Tony Robillard
Donald A. Robin
D. Ashley Robinson
Marc Robinson-Rechavi
Craig Robson
Alfred Roca
Danilo Roccatano
Duccio Rocchini
Flavio Rocha
Luis Rocha

Sonia Rocha
Daniel Rockey
Karl Rockne
Luigi Rodella
Marcio Rodrigues
Fernando Rodrigues-Lima
B. Rodríguez
Antoni Rodriguez-Fornells
Carlos Rodriguez-Ortigosa
Cesar Rodriguez-Saona
Francisco Rodriguez-Valera
Ryan Roeder
Henry Roehl
Bernard Roelen
Klaus Roemer
Lesley Rogers
Lynette Rogers
Igor Rogozin
Myung-Il Roh
Tae-Young Roh
Holger Rohde
Sergio Roiloa
Mauricio Rojas
Antonis Rokas
Louise Rollins-Smith
Peter Roma
Gregg Roman
Stephanie Romanach
Andrej Romanovsky
Floyd Romesberg
Andrea Romigi
Junkang Rong
Suzan Rooijakkers
Ilse Rooman
Roy Roop II
Yan Ropert-Coudert
Pierre Roques
Rafael Rosell
James Rosenbaum
Eli Rosenberg
Karen Rosenberg
Paul Rosenberg
Peter Rosenberger
Joshua Rosenbloom
Cheryl Rosenfeld
Pauline Ross
John Rossi
Sergio Rossi
Marcello Rota
Rossella Rota
Randi Rotjan
Martin Rottenberg
Martin Rottman
Klemens Rottner
Hatem Rouached
Jean-Pierre Rouault
Tracey Rouault
Philippe Rouet
Anna Roujeinikova
Alexandre Roulin
Kasper Rouschop
Jagat Roy
Sudipto Roy
Daniel Rozen
Celine Rozenblat
Elena Rozhkova
Yu Ru Kou
Jianhua Ruan
Craig Ruaux
Craig Rubens
Dustin Rubenstein
Daniel Rubin
Corrado Rubino
Tiziana Rubino
Boris Rubinsky
John Rudan
Thomas Rudel
Uwe Rudolph
Olav Rueppell
Florence Ruggiero
Christiana Ruhrberg
A.R.M. Ruhul Amin
Jodie Rummer
Mark Runco
Klemens Ruprecht
Bayden Russell
Bruce Russell
Charles Russell
Michael Russello
Danilo Russo
Emilio Russo
Suzannah Rutherford
Gerard Rutteman
Benjamin Ruttenberg
Andrey Ryabinin
Sadie Ryan
Valentin Rybenkov
Bernhard Ryffel
Bart Rypma
Choong-Min Ryu
Hoon Ryu
Andrey Rzhetsky
Jamil Saad
Hatem Sabaawy
Kanaga Sabapathy
Massimo Sacchetti
Geetanjali Sachdeva
Todd Sacktor
Sakthivel Sadayappan
Roya Sadeghi

Scheheraz ade Sadegh-Nasseri
Junichi Sadoshima
Maria Saez
Bhaskar Saha
Vikrant Sahasrabuddhe
Upendra Sainju
Nicola Saino
Leonor Saiz
Manabu Sakakibara
Kentaro Sakamoto
Naoya Sakamoto
Dennis Salahub
Mohammad Saleem
Marco Salemi
Christopher Salice
Sara Salinas
Fadi Salloum
Jorge Salluh
Henri Salmon
Freddie Salsbury Jr.
Mauro Salvi
Walter Salzburger
Siba Samal
Rajeev Samant
Dino Samartzis
Suryaprakash Sambhara
Dorit Samocha-Bonet
Karen Samonds
Jaime Sampaio
Maurilio Sampaolesi
Anthony Sampson
Michel Samson
James Samuel
David Samuels
R. Samulski
Carmen San Martin
Javier Sanchez
Angel Sánchez
Víctor Sánchez-Margalet
Jose M. Sanchez-Ruiz
Øyvind Sandbakk
Johan Sandberg
Devinder Sandhu
Jeff Sands
Paul Sandstrom
Qing-Xiang Sang
Miguel A.F. Sanjuán
Osman Sankoh
Anderson Sant'Ana
Fabio Santanelli, di Pompeo d'Illasi
Mario Santiago
Miguel Santin
Daniele Santini
Hélder A. Santos
Janine Santos
Subhabrata Sanyal
Yolanda Sanz
Anna Sapino
Ligia Saraiva
Gabriele Saretzki
Devanand Sarkar
Sabrina Sarrocco
Güher Saruhan-Direskeneli
Marinko Sarunic
Daimei Sasayama
Hidenori Sassa
Juan Sastre
Krish Sathian
Makoto Sato
Abhay Satoskar
Frédéric Saudou
Markus Sauer
Candida Savage
Sevtap Savas
Hideyuki Sawada
Robert Sawers
Nancy Sawtell
Enrico Scalas
Monica Scali
Giuseppe Scapigliati
Maria Rosaria Scarfi
Vinod Scaria
Aldo Scarpa
Cristoforo Scavone
Eliana Scemes
Matthew Schabath
Joseph Schacherer
Martin Schädler
Andreas Schaefer
Andreas Schäfer
Gerwin Schalk
Henk Schallig
Luis Schang
Dena Schanzer
Peter Schausberger
Dirk-Jan Scheffers
Haline Schendan
Andre Scherag
Roberta Scherer
Michael Scheurer
Jay Schieber
Bernd Schierwater
Raphael Schiffmann
Oliver Schildgen
Giuseppe Schillaci
Niels Schiller
Michael Schindler
Stephan Schirmer
Felix Schlachetzki
Stefan Schlatt

Tamar Schlick
Patrick Schlievert
Jens Schlossmann
Christian Schmahl
Benedikt Schmidt
Edward Schmidt
Harald Schmidt
Jennifer Schmidt
Robert Schmidt
Ulrike Schmidt
Axel Schmitt
Fernando Schmitt
Francois Schmitt
Christina Schmitz
Jürgen Schmitz
Mirco Schmolke
Monika Schmoll
Renate Schnabel
Lynn Schnapp
Daniel Schneditz
Bradley Schneider
Marlon Schneider
Regine Schneider-Stock
Matthias Schnell
Joel Schnur
Andrew Scholey
Christian Schönbach
C. Schooling
Jon Schoorlemmer
Veronika Schöpf
Francisco Schopfer
Gideon Schreiber
Katrin Schröder
Michael Schubert
Raymond Schuch
Peter Schuck
Markus Schuelke
Christian Schulz
David Schulz
Guy Schumann
Wolf-Hagen Schunck
Simone Schütz-Bosbach
Adam Schwarz
Oliver Schweiger
François Schweisguth
Luitgard Schwendenmann
Friedhelm Schwenker
Christian Schwentner
Luca Scorrano
James Scott
Jamie Scott
Kristin Scott
Maxwell Scott
Rodney Scott
Hazel Screen
Thomas Scriba
Angelo Scuteri
Tiffany Seagroves
Matthew Seaman
Rebecca Sear
Michael Sears
Leonardo Sechi
Timothy Secomb
Soraya Seedat
Janet Seeley
Antonio Seguro
Roland Seifert
Luis Seijo
Mohamed Seleem
Gernot Sellge
Vimal Selvaraj
Linda Selvey
Szabolcs Semsey
Utpal Sen
Mauricio Sendeski
Irene Sendiña-Nadal
Chaminda Seneviratne
Shantanu Sengupta
John Senko
Masaharu Seno
Luc Sensebé
Balasubramanian Senthilkumaran
Jeong-Sun Seo
Jong Seong Khim
Giuseppe Sergi
Guido Serini
Andrea Serino
Rosa Serra
Emmanuel Serrano Ferron
Karol Sestak
Gautam Sethi
Jaswinder Sethi
Sanjay Sethi
Peter Setlow
Véronique Sgambato-Faure
Sergey Shabala
Vishal Shah
Lion Shahab
Mohammad Shahid
Jeffrey Shaman
Mohammed Shamji
Simon Shamoun
Homayoun Shams
Bin Shan
Hong Shang
Yilun Shang
Bhavani Shankar
Esaki M. Shankar
Kartik Shankar
Rishi Shanker

Feng Shao
Renfu Shao
Mark Shapiro
Igor Sharakhov
Jyotika Sharma
Manu Sharma
Dror Sharon
V. Prasad Shastri
Hagit Shatkay
Peter Shaw
Matthew Shawkey
Gregory Shearer
Chandra Shekhar Bakshi
Eric Shelden
Yiqun Shellman
Han-Ming Shen
Rulong Shen
Sanbing Shen
Wei Shen
Jonathan Sherman
Michael Sherman
Salah Sheweita
Haitao Shi
Honglian Shi
Lei Shi
Qinghua Shi
Wei Shi
Xianglin Shi
Xing-Ming Shi
Xuan-Zheng Shi
Yongtang Shi
Paul Shiels
Clive Shiff
Chiaho Shih
Yen-Yu Ian Shih
Hiroshi Shiku
Eiji Shimizu
Tatsuo Shimosawa
Dong Hoon Shin
Eui-Cheol Shin
Jae-Ho Shin
Toshi Shioda
Shin-Han Shiu
Ralph Shohet
Noam Shomron
Neal Shore
Yogesh Shouche
Naglaa Shoukry
Anil Shrestha
Viji Shridhar
Arun Shukla
Deepak Shukla
Rosely Sichieri
Mark Siedner
Allan Siegel
Britta Siegmund
Mariano Sigman
Israel Silman
Lucas Silva
Olivier Silvie
Robert Sim
J. Pedro Simas
Umberto Simeoni
Michel Simon
Sidney Simon
George Simos
Matthew Simpson
Anthony Sinai
Alison Sinclair
Aran Singanayagam
Andrew Singer
Florian Singer
Ajay Singh
Amit Singh
Brij Singh
Jaswinder Singh
Kamaleshwar Singh
Keshav Singh
Lalit Singh
Pankaj Singh
Ravindra Singh
Seema Singh
Tiratha Singh
Udai Singh
Sunil Singhal
Dinender Singla
Anna-Leena Sirén
Angela Sirigu
Giovanni Sitia
J. Sivaraman
Efthimios Skoulakis
Mikael Skurnik
Kristel Sleegers
Katie Slocombe
Andrzej Slominski
Rob Slotow
Nicolas Sluis-Cremer
Laura Sly
Guy Smagghe
Neil Smalheiser
Richard Smeyne
Hauke Smidt
Alastair Smith
Brenda Smith
Kenny Smith
Lachlan Smith
Matthew Smith
Tara Smith
Thierry Smith
Thomas Smith

Victoria Smith
Wanli Smith
Wm. Leo Smith
Michael Smotherman
Georges Snounou
Joel Snyder
Claudio Soares
Paula Soares
Mario Soberón
Robert Sobol
Irene Söderhäll
Kenneth Söderhäll
Donald Sodora
José Soengas
M. Sohel Rahman
Shay Soker
Alexander Sokolov
Igor Sokolov
Bernd Sokolowski
Christophe Sola
Maria Sola
Aldo Solari
Thierry Soldati
Ricard Solé
Samuel Solomon
Ilia Solov'yov
Ken Solt
Christopher Somers
David Somers
Claudia Sommer
Peter Sommer
Monica Soncini
Chunhua Song
Houbing Song
Linsheng Song
Ping Song
Qing Song
Yiqing Song
Young-Hwa Song
Silvia Sookoian
Torben Sørensen
Carles Soriano-Mas
Khalid Sossey-Alaoui
Giovanni Sotgiu
Erik Sotka
John Souglakos
Mohammed Soutto
Vanessa Souza-Mello
Ali Sovari
Graça Soveral
Ramanathan Sowdhamini
H. Peter Soyer
J. David Spafford
Pieter Spanoghe
Paul Spearman
Roberto Speck
Matthaios Speletas
Juliet Spencer
Markus Sperandio
Niko Speybroeck
Tobias Spielmann
Maria Spies
Mark Spigelman
Charalampos Spilianakis
Donatella Spinelli
Cassandra Spracklen
Rosanna Squitti
Srinand Sreevatsan
Narayanaswamy Srinivasan
Srinivasa Srinivasula
Robert Srygley
Francesco Staffieri
Simona Stäger
Harald Staiger
Jason Stajich
Emmanuel Stamatakis
John Stambas
Martin Stangel
Jo-Ann Stanton
Roscoe Stanyon
Daniel Starczynowski
Peter Starkel
Randi Starrfelt
Roland Steck
Robert Steele
Gary Stein
Dieter Steinhilber
Jena Steinle
Dov Stekel
Salomon Stemmer
Andreas Stengel
Ian Stephen
Stanislaw Stepkowski
Nigel Stepto
Konstantinos Stergiou
Michele Sterling
Annette Sterr
Rick Stevens
Brian Stevenson
David Stewart
James Stewart
Robert Stewart
Ewout Steyerberg
Heinrich Sticht
Knut Stieger
Ymkje Stienstra
Jonathan Stiles
Alan Stitt
Matthias Stöck
Cheryl Stoddart

Georg Stoecklin
Tobias Stoeger
Keith Stokes
Gustavo Stolovitzky
Michael Stout
Cordula Stover
Adam Stow
Yves St-Pierre
Olaf Strauß
Pavel Strnad
Martina Stromvik
Paul Struik
Collin Stultz
Roger Sturmey
Joy Sturtevant
Chien-Wei Su
Yunchao Su
Oscar Suarez
Suresh kumar Subbiah
Selvakumar Subbian
Senthil Subramanian
Rajagopal Subramanyam
Agathe Subtil
Prasanta Subudhi
Jan Suchodolski
Omar Sued
Garret Suen
Cédric Sueur
Izumi Sugihara
Reiko Sugiura
Richard Sugrue
Yousin Suh
Hussein Suleman
David Sullivan, Jr.
Paul Sumby
Masahiko Sumitani
Beicheng Sun
Genlou Sun
Gui-Quan Sun
H. Sunny Sun
Hao Sun
Hong-jin Sun
Jianzhong Sun
Jie Sun
Jun Sun
Kang Sun
Lu-Zhe Sun
Meng-xiang Sun
Qinghua Sun
Qing-Yuan Sun
Shao-Chen Sun
Durai Sundar
Shian-Ying Sung
Philip Supply
Mark Sussman
Catherine Suter
Robert Sutherland
Andrew Sutherland-Smith
Susmit Suvas
Hiromu Suzuki
Per Svenningsson
James Swain
Anand Swaroop
Sharon Swartz
David Swerdlow
William Switzer
Ehsan Syed
Wing-Kin Syn
Gergely Szakacs
Pal Szecsi
Josué Sznitman
Peter Szodoray
Edgardo Szyld
Yvette Tache
Gilda Tachedjian
Michael Taffe
Elda Tagliabue
Shahrad Taheri
Ludovic Tailleux
Heidar-Ali Tajmir-Riahi
Tetsuo Takehara
Nori Takei
Toru Takimoto
Patricia Talamas-Rohana
Alena Talkachova
Marco Tamietto
Ming Tan
Min-Han Tan
Mihai Tanase
Ivan Tancevski
Reshma Taneja
Veena Taneja
Chih-Hsin Tang
Dalin Tang
Daolin Tang
Haixu Tang
Jianming Tang
Julian Tang
Patrick Tang
Shao-Jun Tang
Tieqiao Tang
Yaoliang Tang
Michael Tangrea
Robert Tanguay
Masako Taniike
Hiromu Tanimoto
Yoshiaki Taniyama
Herbert Tanowitz
Malú Tansey
Qian Tao

Yi Tao
Giovanni Targher
Kjetil Tasken
Ming Tat Ling
Satyanarayana Tatineni
Jörg Tatzelt
Christian Taube
Pedro Tauler
Nektarios Tavernarakis
Andrew Taylor
Bradley Taylor
Cormac T. Taylor
Simon Taylor
William Taylor
Bamidele Tayo
Marinus te Pas
Muy-Teck Teh
Ines Teichert
Cristina Teixeira
Francisco Tejedor
Fabien Tell
Dennis Templeton
Piero Andrea Temussi
Hugo ten Cate
Olle Terenius
Jefferson Terry
Dawit Tesfaye
Luca Testa
Kevin Tetteh
Kyaw Tha Paw U
Farook Thameem
Douglas Thamm
Kumarasamy Thangaraj
Binu Tharakan
Thomas Thatcher
Mukund Thattai
Steven Theg
Franziska Theilig
Hugo Theoret
Volker Thiel
Nathan Thielman
Benjamin Thierry
Thimmasettappa Thippeswamy
Bobby Thomas
Diana Thomas
Florian Thomas
Jean-Léon Thomas
Tim Thomas
Torsten Thomas
Brett Thombs
Benjamin Thompson
Cristiane Thompson
Dylan Thompson
Fabiano Thompson
Richard Thompson
Jo Thompson Coon
Sarah Thomsen
Claire Thorne
Simon Thrush
Erik Thuesen
Ryan Thummel
Ronald Thune
Bin Tian
Jie Tian
Xiuchun Tian
Zhixi Tian
Hans Tillmann
Antje Timmer
Nicholas Timpson
Angela Ting
Yin Tintut
Tan To Cheung
Michal Toborek
Takashi Toda PhD
Matthew Todd
Peter Todd
Stephen Todryk
Sergio Tofanelli
Philip Tofilon
Mathias Toft
Amanda Toland
Stephen Tompkins
Hung Ton-That
Laszlo Tora
Karen Tordjman
Ali Torkamani
Maria Lina Tornesello
Christopher Torrens
Eduard Torrents
Néstor Torres
Silvio Tosatto
Gianluca Tosini
Jorg Tost
Jonathan Touboul
Kazushige Touhara
Martin Tovée
Kazunori Toyoda
Philip Trackman
Rochelle Tractenberg
Vladimir Trajkovic
Anna Tramontano
Dat Tran
Lam-Son Tran
Ulrich Tran
Gregory Tranah
Yara Traub-Csekö
Alexander Travis
Mohamed Trebak
Jason Tregellas
John Tregoning

François Tremblay
Jose Trevino
Elizabeth Triche
Susheela Tridandapani
Srikanth Tripathy
Claudio Tripodo
Ralph Tripp
Prabodh Trivedi
Giancarlo Troncone
Francois Trottein
Caroline Trotter
Martha Trujillo
Ramon Trullas
Randy Trumbower
Alexander Tsai
Robert Tsai
Herman Tse
Ching-Ping Tseng
Scheffer Tseng
George Tserpes
Athanassios Tsikliras
Effie Tsilibary
Lev Tsimring
Stella Tsirka
Sophia Tsoka
Maria Tsokos
Panagiotis Tsonis
Takafumi Tsuboi
Hiroyuki Tsuchiya
Kenji Tsuchiya
Moriya Tsuji
Yoshiaki Tsuji
Hirokazu Tsukaya
Yu-Kang Tu
Jan Tuckermann
Tamir Tuller
Michael Tunney
Stephen Turner
Juha Tuukkanen
Anil Tyagi
Radouil Tzekov
A. Aria Tzika
Monica Uddin
Venkatachalam Udhayakumar
Matsuo Uemura
Victor Ugaz
Satish Ukkusuri
Ilya Ulasov
Michael Ullman
Henning Ulrich
Govindhaswamy Umapathy
Shahid Umar
James Umen
Richard Unsworth
Derya Unutmaz
Turgay Unver
Cosimo Urgesi
Masuko Ushio-Fukai
Bob Uttl
Jaydutt Vadgama
Jamunarani Vadivelu
Krishna Vadrevu
Chandan Vaidya
Pedro Valdes-Sosa
Giovanna Valenti
John Valentine
Nicole Valenzuela
Therese van Amelsvoort
Pieter van Baal
Robert van Beers
Willem van Berkel
Michiel van Boven
Els van Damme
Peter van den Besselaar
Ruud van den Bos
Eva Van den Bussche
Christina van der Feltz-Cornelis
F. Gisou van der Goot
Johan van der Vlag
Patrick van der Wel
Lucas van der Woude
Paul van Diest
Michiel van Elk
Martijn van Griensven
Leo van Grunsven
Thorbald van Hall
Jack van Honk
Gilles van Luijtelaar
Peter van Ooijen
Jim van Os
Leonard van Overbeek
Mark van Raaij
Hedderik van Rijn
Frank van Rijnsoever
Willem van Schaik
Maurice van Steensel
Hendrik W. van Veen
Andre van Wijnen
Ruud van Winkel
Jacobus van Wouwe
Nico W. Van Yperen
Ger van Zandbergen
Menno van Zelm
Richard van Zyl-Smit
Jean-Marc Vanacker
Klaas Vandepoele
Deborah Vanderveen
Luca Vanella
Seralynne Vann
Sven Vanneste

Steven Varga
Jean-Pierre Vartanian
Deepak Vashishth
Eleni Vasilaki
Nikos Vasilakis
Athanasios Vasilakos
Cristina Vassalle
Manu Vatish
Hubert Vaudry
Lloyd Vaughan
Demetrios Vavvas
Rafael Vazquez-Duhalt
Gayatri Vedantam
Rakesh N. Veedu
Guillermo Velasco
Binu Velayudhan
Digna Velez Edwards
Adrian Vella
Giovanni Vendramin
Thiyagarajan Vengatesen
Salvador Ventura
Vittorio Venturi
Julio Vera
Antonio Verdejo-García
Tom Verguts
Bruno Verhasselt
Esther Verheyen
Chandra Verma
Suresh Verma
Geerat Vermeij
Eric Vermetten
Sten Vermund
David Vernon
Akos Vertes
Beata Vertessy
Aristidis Veves
Laura Via
Cecile Viboud
David Vicario
Mark Vickers
Giuseppe Viglietto
Neeraj Vij
Jose Vilar
Erica Villa
Pablo Villoslada
Andreas Villunger
Jose Vina
Boris Vinatzer
Maria Cristina Vinci
Manlio Vinciguerra
Joao Viola
Amarjit Virdi
Gianni Virgili
Laurent Viriot
Marie-Joelle Virolle
Virginia Vitzthum
Antonia Vlahou
Sergei Volis
Michael Volkert
David Volle
Steve Vollmer
Ute Vollmer-Conna
Arndt von Haeseler
Ulrich von Hecker
Matthias von Herrath
Frank Voncken
John Vontas
Marc Vooijs
Christian Voolstra
Kay Vopel
Martin Voracek
Ina Vorberg
Michiel Voskuil
Daniel Voth
Kent Vrana
Nalini Vudattu
Edward Vul
Vladyslav Vyazovskiy
Claire Wade
Gebhard Wagener
Bridget Wagner
Daniel-Christoph Wagner
Wolfgang Wagner
Tuck Wah Soong
Tom Waigh
Mark Wainberg
Ari Waisman
Piotr Walczak
Jonas Waldenström
Lourens Waldorp
Graham Wallace
John Wallace
Joseph Wallace
Ross Waller
Consuelo Walss-Bass
Martin Walter
Xiaoang Wan
Chuan-Chao Wang
De-Hua Wang
Dong Wang
Fei Wang
Guoying Wang
Haibin Wang
Hanping Wang
Hong Wang
Hongyan Wang
James Wang
Jieru Wang
Junming Wang
Junwen Wang

Kai Wang
Kunbo Wang
Li Wang
Long Wang
Meijing Wang
Pei Wang
Ping Wang
Qiang Wang
Qiming Wang
Rui-Wu Wang
Shiping Wang
Shixia Wang
Tai Wang
Tian Wang
Ting Wang
Tony Wang
Wei Wang
Xiaochen Wang
Xiao-Dong Wang
Xiaofeng Wang
Xiao-Wei Wang
Xiaoying Wang
Xiujun Wang
Yanchang Wang
Yan-Ling Wang
Yeng-Tseng Wang
Yi Wang
Yibin Wang
Ying-Jan Wang
Yuanquan Wang
Yue Wang
Yun Wang
Zhen Wang
Zhengfeng Wang
Zhengqi Wang
Zonghua Wang
Mohan Wani
Hong Wanjin
Meni Wanunu
David Warburton
Darren Ward
Lawrence Ward
W. Steven Ward
Gulam Waris
Digby Warner
Eric Warrant
Kirk Warren Brown
Kishore Wary
Katsumi Watanabe
Koichi Watashi
Grant Waterer
Dan Weary
Eric Weaver
Edward Webb
Andrew Webber
Christopher Weber
Martin Weber
Nicole Webster
Heiner Wedemeyer
Andreas Wedrich
Christian Wegener
Barbara Wegiel
Xander Wehrens
Chongyi Wei
Huafeng Wei
Qingyi Wei
Zhi Wei
Hong Wei Chu
Norbert Weidner
Joel Weiner
Aviv Weinstein
Tiffany Weir
Robert Weiss
Stefan Weiss
Alessandro Weisz
Jörn-Hendrik Weitkamp
Jonathan Weitzman
Jeffrey Welker
Raymund Wellinger
Rong Wen
Z. Wen
Xuchu Weng
Thomas Wennekers
Peter Wenner
Heiman Wertheim
Anne Wertheimer
James West
Christopher Wheeler
Bryan White
Edward White
Helen White-Cooper
Marlène Wiart
Gerhard Wiche
Marieke Wichers
Jelte Wicherts
Claude Wicker-Thomas
Anja Widdig
Eva Widerström-Noga
David Wieczorek
Hans-Joachim Wieden
Heinz Wiendl
Frank Wieringa
Harriet Wikman
Andrew Wilber
Willy Wilbur
Andrea Wiley
Peter Wilf
Katalin Wilkinson
Jennifer Wilkinson-Berka
Bart Williams

Cecilia Williams
Jeffrey Williams
John Williams
Mark Williams
James Wilsdon
Brenda Wilson
Emma Wilson
Megan Wilson
Richard Wilson
Carol Wilusz
Sabine Windmann
Wendy Wing Tak Lam
Shannon Wing-Ngor Au
Christoph Winkler
Robert Winn
Gary Winslow
Alan Winston
Carlos Winter
Thomas Wishart
Stephan Witt
Paul Witten
Alexander Wlodawer
Christiane Wobus
Dominik Wodarz
Collynn Woeller
Andrew Wolfe
Stefan Wölfl
Gayle E. Woloschak
Chun-Ming Wong
Frank Wong
G. Wong
Kwong-Kwok Wong
Nai Sum Wong
Sek-Man Wong
Thian Sze Wong
Vincent Wong
William Wong
Patrick Woo
Chris Wood
Gavin Woodhall
Charles Woodrow
Kerry Woolfall
R. Wooten
Stefan Worgall
Floyd Wormley Jr.
K. Brad Wray
Christine Wrenzycki
James Wright
Neil Wright
Han-Chung Wu
Keqiang Wu
Min Wu
Min-Hsien Wu
Ping-Hsun Wu
Qiang Wu
Qing Wu
Qiong Wu
Rongling Wu
Shu-Biao Wu
Song Wu
Wen-Chih Wu
Xifeng Wu
Ye Wu
Yuntao Wu
Zhi-Ying Wu
Gerhard Wunderlich
Anton Wutz
Doug Wylie
Glenn Wylie
Cheng-Yi Xia
Houhui Xia
Yinglin Xia
Gaoxi Xiao
Jingfa Xiao
Qingzhong Xiao
Jingwu Xie
Keping Xie
Shang-Ping Xie
Xing Xie
Zhi Xie
Zhongcong Xie
Yi Xing
Zheng Xing
Momiao Xiong
Benjamin Xu
Bing Xu
Changjie Xu
Eric Xu
Haiyan Xu
Hui-Xiong Xu
Jianhua Xu
Jianqing Xu
Jun Xu
Mingliang Xu
Mingqing Xu
Peng Xu
Ping Xu
Shang-Zhong Xu
Shuang-yong Xu
Wei Xu
Wenqing Xu
Xiaolei Xu
Yan Xu
Ying Xu
Yingjie Xu
Zongli Xu
Bin Xue
Yu Xue
Rattan Yadav
Nagendra Yadava

Soroku Yagihashi
Judy Yam
W.C. Yam
Masaya Yamamoto
Yoshihiro Yamanishi
Yoshio Yamaoka
Taro Yamashita
Hidenori Yamasue
Shin Yamazaki
Chunhong Yan
Jun Yan
Riqiang Yan
Wei Yan
Yong-Bin Yan
Zhen-guang Yan
Hiromi Yanagisawa
Burton Yang
Chengfeng Yang
Chuen-Mao Yang
Daichang Yang
Fan Yang
G Yang
Guangxiao Yang
Haibing Yang
Hongyuan Yang
Isaac Yang
Jian Yang
JianJun Yang
Jinn-Moon Yang
Li Yang
Ming Yang
Pingfang Yang
Rongge Yang
Shaolin Yang
Shihui Yang
Wan-Xi Yang
Xi Yang
Xiao-Feng Yang
Xiaoming Yang
Xiaoyan Yang
Yang Yang
Yanmin Yang
You Yang
Zeng-Ming Yang
Krassimir Yankulov
Kentaro Yano
Cedric Yansouni
Dezhong Yao
Lixia Yao
Maosheng Yao
Yong-Gang Yao
Pew-Thian Yap
Vinod Yaragudri
Andrew Yates
Jianping Ye
Sheng Ye
Xiaoqin Ye
Zhiping Ye
Peh Yean Cheah
Eldad Yechiam
Hui-Ling Yen
Sai Yendamuri
Sachin Yende
Suresh Yenugu
Andrew Yeudall
Huso Yi
Özlem Yilmaz
Hang Yin
Tongming Yin
Zhan Yin
Ken Yoda
Junji Yodoi
Utako Yokoyama
Jae Yong Han
Byung-Jun Yoon
Kyoung-Jin Yoon
Young-sup Yoon
Takeo Yoshikawa
Akihiko Yoshimura
Bridget Young
Kyle Young
Martin Young
Roger Young
Scott Young
Reza Yousefi
Mohammed Yousfi
Choongho Yu
Fu-Shin Yu
Herbert Yu
Hong-Guo Yu
Jae-Hyuk Yu
Jianhua Yu
Jr-Kai Yu
Jun Yu
Kefei Yu
LIQING YU
Lu-Gang Yu
Ming-Lung Yu
Rongjun Yu
Sidney Yu
Xiao-Fang Yu
Xiao-Qiang Yu
Xu Yu
Xue-Jie Yu
Yang Yu
Fan Yuan
Jiajin Yuan
Tifei Yuan
Wenping Yuan
Y. Adam Yuan

Bi-Song Yue
Gen Hua Yue
Junming Yue
Chiou-Hwa Yuh
Ruan Yuhua
Joshua Yukich
Sung-Hwan Yun
Wing-ho Yung
Vyacheslav Yurchenko
Andrey Yurkov
Katherine Yutzey
Mark Zabel
Olga Zabotina
Amir A. Zadpoor
René Zahedi
Tamas Zakar
Tiziana Zalla
Elias Zambidis
Dario Zamboni
Lorenzo Zane
Dirce Zanetta
Mengwei Zang
RunGuo Zang
Yu-Feng Zang
Gianluigi Zanusso
Carlos Zaragoza
Oscar Zaragoza
Dmitri Zaykin
Cecilia Zazueta
Hajo Zeeb
Mirjam Zegers
Jochen Zeil
Tanja Zeller
Ana Zenclussen
Fan-Gang Zeng
Li Zeng
Alma Zernecke
Kornelius Zeth
Dimitrios Zeugolis
Xiangming Zha
Aimin Zhang
Baohong Zhang
Chi Zhang
Chiyu Zhang
Daoqiang Zhang
Dingguo Zhang
Fuli Zhang
Ge Zhang
Guihong Zhang
Guoping Zhang
Harry Zhang
Heye Zhang
Hong Zhang
Hong-Liang Zhang
Huiping Zhang
Jian Zhang
Jianhua Zhang
Jianmin Zhang
Jinfa Zhang
Jinsong Zhang
Jin-Song Zhang
Lanjing Zhang
Li Zhang
Luo Zhang
Luwen Zhang
Meijia Zhang
Ming Zhang
Nanyin Zhang
Peng Zhang
Qijing Zhang
Qin Zhang
Qinghui Zhang
Quanguang Zhang
Shu-Dong Zhang
Shuguang Zhang
Tianzhen Zhang
Weili Zhang
Xiang Zhang
Xianlong Zhang
Xiaoliang Zhang
Xin Zhang
Yanbin Zhang
Yang Zhang
Yanqiao Zhang
Y-H Percival Zhang
Yingchun Zhang
Yingfeng Zhang
Yinping Zhang
Youjun Zhang
Yudong Zhang
Zhang Zhang
Zhao Zhang
Zhe Zhang
Zhengdong Zhang
Zhengguang Zhang
Zhiqian Zhang
Zhuo Zhang
Zhuoli Zhang
Zi-Ke Zhang
Chunfeng Zhao
Feng Zhao
Heng Zhao
Jianjun Zhao
Min Zhao
Richard Zhao
Shuhong Zhao
Xiuhai Zhao
You-Yang Zhao
Deyou Zheng
Jialin Zheng

Jie Zheng
Song Guo Zheng
Yingfeng Zheng
Yiwen Zheng
Yun Zheng
Zhi-Ming Zheng
Boris Zhivotovsky
Guangming Zhong
Changsong Zhou
Dongsheng Zhou
Fengfeng Zhou
Hua Zhou
Huaijun Zhou
Juan Zhou
Meixue Zhou
Renping Zhou
Shengtao Zhou
Weijun Zhou
Wei-Xing Zhou
Xi Zhou
Xiangtian Zhou
Xuhui Zhou
Xu-jie Zhou
Yanming Zhou
Yifeng Zhou
Yong Zhou
Yunli Zhou
Zhi Zhou
Zhongjun Zhou
Chao-Dong Zhu
Donghui Zhu
Dongxiao Zhu
Jiahua Zhu
Kun Yan Zhu
Liping Zhu
Shankuan Zhu
Wei-Guo Zhu
Xuewei Zhu
Yi Zhun Zhu
Zhiming Zhu
Xiaoxi Zhuang
Asim Zia
Kirk Ziegler
Elke Zimmermann
Andreas Zirlik
Gernot Zissel
Michal Zmijewski
Michal Zochowski
Erwin Zoetendal
Quan Zou
Xi-Nian Zuo
