# Supplementary material for: PLOS ONE 2016 Reviewer and Editorial Board Thank You
Source: PLoS One. 2017 Mar 20;12(3):e0174259. doi: 10.1371/journal.pone.0174259 (PMC5358840; doi:10.1371/journal.pone.0174259)
Supplement: S1 Reviewer List — (PDF) [file pone.0174259.s002.PDF]

PLOS ONE would like to thank all those who reviewed on behalf of the journal in 2016:

Tuomas Aakala
Rob Aalberse
Daniel Aalberts
Inge H. Aalders
Mikko Aalto
Håvard Aanes
James Aanstoos
Bizhan Aarabi
Jiska Aardoom
K. Aarnio
Erika Aaron
Gloster Aaron
Grant Aaron
Shawn Aaron
Stuart Aaronson
Hans Aarstad
Henk Aarts
Ulrika Aasa
Jan Aasly
Tor Aasmundstad
Mervi Aavikko
Safa Aazar
Nurul Hidayah Ab Rahman
Elena Abad
Eduardo Abade
Fernando Abad-Franch
Tabare Abadie
Tatjana Abaffy
Nicole Abaid
Georgios Abakoumkin
Jenny Abanto
Francisca Abanyie
Winston Abara
Samuel Abariga
Federico Abascal
Aniekan Abasiattai
Zaid Abassi
Davide Abate
Nicola Abate
John Abatzoglou
Theodore Abatzopoulos
Mohammed Abba
Yusuf Abba
Faisal Abbas
Haider Abbas
Hashim Abbas
James Abbas
Mohamad Abbas
Syed Abbas
Ali Abbasi
Amir Abbasi
Hussein Abbass
Maggie Abbassi
Jessica Abbate
Pable Abbate
Angela Abbatecola
Souheila Abbeddou
Olivier Abbo
Shahal Abbo
Attilio Abbondanza
Ciro Abbondanza
Amin Abbosh
David H. Abbott
Kristen Abbott
Louise Abbott
Lynette Abbott
Marcia Abbott
Ryan Abbott
William Abbott
Steven Abcouwer
Ekram Abd El Wahab
Ahmed Abd El Wahed
Taia Abd El-Mageed
Ekram Abd El-Wahab
Nadia Abdala
Chadi Abdallah
F. Abdallah
Florence Abdallah
Ayesha Abdeen
Mohamed Abdel Hakeem
Rania Abdel Hay
Arafat Abdel Latef
Ahmed Abdel Moneim
Mohamed Abdelbary
Ahmed Abdelfattah
Nizar Abdelfattah
Tamer Abdelkader
Ahmed Abdel-Latif
Mohamed Abdelmegeed
Kotb Abdelmohsen

Anas AbdelRahman
Hamada Abdelrahman
Yasser AbdelRahman
Amr Abd-Elrahman
Ali AbdElrazek
Essam Abdel-Sattar
Nourtan Abdeltawab
El-sayed Abdelwhab
Hamidreza Abdi
Reza Abdi
Edimansyah Abdin
Walied Abdo
Zaid Abdo
Jalal Abdolalizadeh
Asghar Abdoli
Hamid Abdollahi
Hamid Abdolmaleky
A. S. Abdoon
Abdelkarim Abdrabo
Kartini Abdul Jabar
Muhammad Khalis Abdul Karim
Dania Abdul Malak
Suraya Abdul Razak
Ethayathulla Abdul Samath
Amr Abdulazim
Noraishah Abdul-Aziz
Mohamed Faizal Abdul-Careem
Rashad Abdul-Ghani
Mehmet Abdulhayoglu
Hassan Abduljabbar
Regina Abdulkader
Abu Sayeed Abdullah
Afnizanfaizal Abdullah
Kalil Abdullah
Maha Abdullah
Jibril Abdulmalik
Raja-Elie Abdulnour
Muhydeen Abdulraheem
Samia Abdul-Rahman
Midhat Abdulreda
Ali Abdul-Sater
Amal Abdul-Sattar
Jaafar Abduo
Hirokazu Abe
Junichi Abe
Keietsu Abe
Kohtaro Abe
Takaaki Abe
Yoshifumi Abe
Kaleab Abebe
Solomon Abebe
Francisco Abecasis
Leticia Abecia
Ahmed Abed
Behnoush Abedi-Ardekani
Lily Abedipour
Alyson Abel
Gregory Abel
Kathryn Abel
Larry Abel
Willie Abel
Sailau Abeldenov
Monica Abella
James Abelson
Bart Aben
Ludovico Abenavoli
Michael Abend
Jan Abendroth
Daniel Aberdam
Kristoffer Aberg
Christoffer Åberg
Avraham Abergel
Charbel Abi Khalil
Md. Ruhul Abid
Elia Abi-Jaoude
Anders Abildgaard
Oscar Abilez
Ajibola Abioye
Laurent Abi-Rached
Joe Abisambra
Carolyn Abitbol
Marc Abitbol
Alfonso Abizaid
Adib Abla
Jacob Ablin
Susan Abmayr
Erin L. Abner
Drew Abney
Eric Aboagye
Mohamed Aboelmaged
Vicente Aboites
Gabriella Abolafio
Melanie Abonnenc
M. E. Abood
Ahmed Aboraia
Anne Abot
Rachid Abou
Hussein Abou Abbass
Mahmoud Abou Alaiwa
Hadi Abou El Hassan
Majdi Abou Najm
Mona Abou Zahra

Frances Aboud
A. Abou-Eisha
Ahmed Abouelnour
Mourad Aboul-Soud
Eliane Abou-Mansour
Anna Abou-Raya
Carla AbouZahr
Khaled Abouzid
Allistair Abraham
Analìa Abraham
Asha Abraham
Dennis Abraham
Eleni Abraham
Gad Abraham
Isaac Abraham
Ivo Abraham
John Abraham
Nader Abraham
Sheela Abraham
T. Abraham
Mark Abrahams
Naeemah Abrahams
Sharon Abrahams
Elger Abrahamse
Mariëlle Abrahamse
Paul Abram
Amitai Abramovitch
Steven Abramowitch
Matthew Abramowitz
Barbara Abrams
Harvey Abrams
Joseph Abrams
Julian Abrams
Marshall Abrams
Peter Abrams
Thomas Abrams
John Abramson
Myriam Abramson
Jacqueline Abranches
Isabel Abrantes
Mauricio Simões Abrão
Patrícia Antonia Estima Abreu
João Abreu Neto
Ana Lucia Abreu-Silva
Avital Abriel
Hugues Abriel
Jose Abril
Ravinder Abrol
Saeid Abroun
Sabrina Absalon
David Abson
Elliot Abt
Ruzilawati Abu Bakar
Asad Abu Bakar Md Ali
Redzal Abu Hanifah
Hatem Abu Hashim
Leen Abu Safieh
Ahmad Abu-Akel
Ado Abubakar
Abdulai Abubakari
Mario Abud
Aierken Abudu
Kuerbanjiang Abuduxikuer
Sara Abuelmaali
Mohamed Abu-Farha
Shada Abuhattoum
Muhanned Abu-Hijleh
Abde Abukhdeir
Salem Abukres
Nada Abumrad
Lisa Abuogi
Synan AbuQamar
Octavio Aburto-Oropeza
Ala' Fahed Abdelhameed Aburumman
Mahmoud Abu-Shakra
Loreto Abusleme
Adnan Abu-Surrah
Timothy Abuya
Maurizio Acampa
Ali Acar
Gabriele Accetta
Ettore Accolla
Salvatore Accomando
Filippo Acconcia
José Acebes
Carmen Aceijas
Arianna Aceti
Paola Aceto
Andres Acevedo
Flor Acevedo
Jorge Acevedo
Miguel Acevedo
Pelayo Acevedo
Alejandro Acevedo-Gutierrez
Karina Acevedo-Whitehouse
Thomas Ach
Palakorn Achananuparp
Animesh Acharjee
Bhoj Acharya
Chaitanya Acharya
Chirag Acharya
Kshitish Acharya

Pawan Acharya
Vishal Acharya
Amelia Acha-Sagredo
David Achat
Guillaume Achaz
Barry Ache
Francine Acher
Yvonne Achermann
Amit Achhra
Michael Achiam
Samuel Achilefu
Felice Achilli
Alexis Achim
Jacqueline Achkar
Anja Achtziger
Bhagelu Achyut
Seher Bahar Aciksöz Özden
Rebeca Acin-Perez
Rakefet Ackerman
William Ackerman
Paul Ackermann
Cheryl Ackert-Bicknell
Brian Ackley
Karen Ackroff
Hervé Acloque
Jaime Acosta
Jose Acosta
Oscar Acosta
Sandra Acosta
George Acquaah-Mensah
Kimberly Acquaviva
Claudia Acquisti
Luis Actis
Jeffrey Actor
Stefan Aczel
Hisashi Adachi
Katsuyuki Adachi
Souichi Adachi
Taiki Adachi
Takehiko Adachi
Yoshikazu Adachi
Yushii Adachi
Timothy Adair
Alejandro Adam
Barry Adam
Elhadi Adam
Iris Adam
Ishag Adam
Thomas Adam
Tom Adam
Vannay Adam
Agata Adamczyk
Igor Adameyko
Giovanni Adami
Guy Adami
Darryl Adamko
Sergio Adamo
Marios Adamou
Jan Adamowicz
Stephane Adamowicz
Wiktor Adamowicz
Amanda Adams
Christopher Adams
Crystal Adams
David Adams
Dean Adams
Henry Adams
John Adams
Jonathan Adams
Joseph Adams
Justin Adams
Kenneth Adams
Mark Adams
Meredith Adams
Michael Adams
Tim Adams
Maja Adamska
Piotr Adamski
Zbigniew Adamski
David Adamson
Justus Adamson
Robert Adamson
Samuel Adamson
Grazyna Adamus
Roger Adan
Ram Adapa
Nithin Adappa
Eli Adashi
K. Adb
Ian Adcock
D. M. Adcock Funk
Shaquria Adderley
Maria Filippa Addis
Anthony Addlagatta
Nii Addy
Ogun Adebali
Clement Adebamowo
Esther Adebayo
Ezekiel Adebiyi
Adeyemi Adedeji
Dennis Adeegbe
Olasunkanmi Adegoke

Victor Adekanmbi
Adesina Adekunle
Ehsan Adeli
Davies Adeloye
Karine Adel-Patient
Coen Adema
Abiodun Adeoye
Alex Ades
Adegbola Adesogan
Daniel Adesse
Rasheed Adeyemi
John Adgate
Atin Adhikari
Badri Adhikari
Bipin Adhikari
Neill Adhikari
Nirmal Adhikari
Rajan Adhikari
Ramesh Adhikari
Till Adhikary
Satish Adiga
Esther Adi-Japha
Bryon Adinoff
Karim Adiprasito
Vitria Adisetiyo
Andrew Adjei
Nicholas Adjei
DeAnna L. Adkins
Elizabeth Adkins-Regan
Milo Adkison
Wolfram Adlassnig
Heiko Adler
Peter Adler
Nagesh Adluru
Belayneh Admassu
Wilfried Admiraal
Arie Admon
Roee Admon
Mikael Adner
Frederic Adnet
Serge Adnot
Yaw Adomako-Ankomah
Tarso Adoni
Véronique Adoue
Teresa Adragao
Eric Adriaenssens
Lopes Adriana
Walter Adriani
Emily Adrion
Seth Adu-Afarwuah
Dennis Adu-Gyasi
Carolina Adura
Sahir Advani
Wichai Aekplakorn
Gemma Aellah
Hugo Aerts
Jean-Marie Aerts
Johannes Aerts
Peter Aerts
Daniel Aeschlimann
Isa Afan
Farrukh Afaq
Saima Afaq
Solomon Afelik
Susanne Affenzeller
Nicholas Affrunti
Enis Afgan
A. Rahim Afidah
Maha Afifi
Adeleye Afolayan
Ana Afonso
Nikolaos Afratis
Evans Afriyie-Gyawu
Dil Afroze
S. Afshar
Babak Afsharipour
Neda Afsharkhamseh
Rebecca Aft
Rizwan Afzal
Mohammad Afzal Khan
Mohamed Ag Bendech
Neda Agahi
Sultan Agalarov
Christos Agalianos
Dritan Agalliu
Ilir Agalliu
Agar Agar
Meera Agar
Ameeta Agarwal
Amitesh Agarwal
Anupam Agarwal
Beamon Agarwal
Gunjan Agarwal
Kshitij Agarwal
Mukesh Agarwal
Nisheeth Agarwal
Rajiv Agarwal
Rajkumar Agarwal
Seema Agarwal
Smisha Agarwal
Soumik Agarwal
Subhash Agarwal

Suresh Kumar Agarwal
Vibhu Agarwal
Vikas Agarwal
Vinayak Agarwal
Meghna Agarwala
Seema Agarwala
Luciano Agati
Kemal Agbaht
Jimoh Agbaje
Mavis Agbandje-McKenna
Yuksel Agca
Shinsuke Agehara
Alex Agelidis
Dau Aggarwal
Monika Aggarwal
P. Aggarwal
Rohit Aggarwal
Saurabh Aggarwal
Shikhar Aggarwal
Srijan Aggarwal
V. Aggarwal
George Aggelis
Samuel Aggrey
Mickey Agha
Naser Aghababaei
Mohmoud Aghaei
Mortaza Aghbashlo
Ali Aghdassi
Alessio Aghemo
Manish Aghi
Rokhsareh Aghili
Ahmad Agil
Hermann Agis
Paul Agius
Konstantin Agladze
Luca Agnelli
Francesco Agnello
Emily Agnew
Philippa Agnew
Sameer Agnihotri
Gabriella Agnoletti
Sergio Agnoli
Yukio Ago
Antonella Agodi
Simon Agolory
Federica Agosta
Donat Agosti
Angelo Agostinho
Aubert Agostini
Elizabeth Agostini
Marco Agostini
Tiziano Agostini
Denes Agoston
Piergiuseppe Agostoni
Kawango Agot
Alexander Agoulnik
William Agras
Alok Agrawal
Aneil Agrawal
Anurag Agrawal
Arpana Agrawal
Arun Agrawal
Devendra Agrawal
Gaurav Agrawal
Rupesh Agrawal
Sandeep Agrawal
Suraksha Agrawal
Ruben Agrelo
Susanna Ågren
Marco Agrifoglio
Alexander Agrios
Paul Agris
Chukwuma Agu
Begoña Aguado
José María Aguado
Marta Aguar
Antonio Agüera
Maria Agüera
Zaida Agüera
Umberto Aguglia
Aderbal Aguiar
Anna Caroline Aguiar
Cristina Aguiar
Wilson Aguiar
Pedro Aguiar Junior
Gianluca Aguiari
Hector Aguila
Alex Aguilar
Erwin Aguilar
Hector Aguilar
Patricia Aguilar
Sebastian Aguilar Pierlé
Hector Aguilar-Carreno
Carlos Aguilar-Salinas
Ximena Aguilera
Isidro Aguillo
Nacho Aguilo
Dianne Joy Aguilon
Andre Aguirre
Armando Aguirre
Carlos Aguirre
Jesus Aguirre

Urko Aguirre
Wael I. Agur
Trevor Agus
Tetsuro Agusa
Jordi Agustí
Rina Agustina
Christian Agyare
Irene Agyepong
Arunee Ahantarig
Seyedhossein Aharinejad
Efrat Aharonov-Majar
Gerard Ahern
Jeffrey Ahern
Dinesh Ahirwar
Merav Ahissar
Per Ahlberg
Christoph Ahlgrim
Mark Ahlman
Ola Ahlqvist
Jayne Ahlstrom
Aasim Ahmad
Abdullah Ahmad
Aftab Ahmad
Ali Ahmad
Anis Ahmad
Aqeel Ahmad
Arsalan Ahmad
Basir Ahmad
Faridahanim Ahmad
Imran Ahmad
Mahmood Ahmad
Nafees Ahmad
Nazni Ahmad
Riaz Ahmad
Saif Ahmad
Shaad Ahmad
Shandar Ahmad
Yasmin Ahmad
Zeeshan Ahmad
Aida Syarinaz Ahmad Adlan
Mohamed Ahmad Hassali
H. Ahmadi
Keivan Ahmadi
Alireza Ahmadian
Ali Reza Ahmadvand
Bilaal Ahmed
Magdy Ahmed
Merina Ahmed
Mohamed Ahmed
Mohamed Saveer Ahmed
Monjur Ahmed
Mukhtar Ahmed
Naseer Ahmed
Nova Ahmed
Rebekah Ahmed
Saad Ahmed
Saifuddin Ahmed
Samrah Ahmed
Tofael Ahmed
Umar Ahmed
Warish Ahmed
Zaghloul Ahmed
Noha Ahmed Nasef
Brian Ahmedani
Curie Ahn
Dong Ahn
Hanjong Ahn
Hyochol Ahn
Jaehong Ahn
Ji Hoon Ahn
Joong Kyong Ahn
Ki Hoon Ahn
Minkoo Ahn
Myung-Ju Ahn
Sang-Joon Ahn
Sun Joo (Grace) Ahn
Woo-Young Ahn
Yoon-Ok Ahn
Sebastian Ahnert
Collins Ahorlu
Oussama Ahrazem
Md. Rezwanul Ahsan
Syed Ahsan
Mehmet Ahsen
Divya Ahuja
Ishita Ahuja
Manuj Ahuja
Shama Ahuja
Jorge Ahumada
Bin Ai
Qinghui Ai
Bruno Aiazzi
Takeshi Aiba
Tomomi Aida
Yoko Aida
Allison Aiello
Brett Aiello
Ernesto Aiello
Franklin Aigbirhio
Michael Aigner
Michiko Aihara
Yoko Aihara

Tan Aik Kah
Takuya Aikawa
Abigail Aiken
Catherine E. Aiken
Moses Aikins
Laurie Ailles
Flavio Aimbire
Ashley Aimone
Alar Ainla
David Ainley
Jose Ainsa
Cameron Ainsworth
Elizabeth Ainsworth
Roberto Aiolfi
Manuel Aira
Anu Airaksinen
Mikko Airavaara
Marta Aires-de-Sousa
Valentina Airi
Edoardo Airo
Edoardo Airò
I.P. Aisiku
Ounissa Aït-Ahmed
Tahar Ait-Ali
Guruprasad Aithal
Elisabeth Aitken
Elizabeth Aitken
Samuel Aitken
Copper Aitken-Palmer
Timothy Aitman
Hafid Ait-Oufella
Ke Aiwu
Yoshifusa Aizawa
Mokrish Ajat
Divya Ajay
Ayo Ajayi
Sadia Ajaz
Vladeta Ajdacic-Gross
Dragana Ajdic
Marco Ajelli
Omotomilola Ajetunmobi
A. Bolu Ajiboye
Ghada Ajlani
Said Ajlouni
Paolo Ajmone Marsan
Kolapo Ajuwon
Dilek Ak
Peter Aka
Rinji Akada
Kazutaka Akagi
Ryota Akagi
Satoshi Akagi
Tadamichi Akagi
Tomohiko Akahoshi
Masami Akai
Alberto Akama
Nobuhisa Akamatsu
Tomonari Akamatsu
Maxwell Akanbi
Sajid Hamid Akash
Chihiro Akazawa
Hiroshi Akazawa
James Akazili
Michael Akbar
Noreen Akbar
Hamid Akbarali
Mohammad Akbari
Schahram Akbarian
M.K. Akboga
F. Akcha
Guray Akdogan
Sam Akech
Koji Akeda
Randall Akee
Dickens Akena
Katherine Akers
R. Akers
Tobias Akerstrom
Baki Akgül
Omid Akhavan
Pegah Akhavan
Haleh Akhavan-Niaki
Alireza Akhondi-Asl
Most Mauluda Akhtar
Muhammad Akhtar
Riaz Akhtar
Yasmin Akhtar
Murtaza Akhter
Nasima Akhter
Yusuf Akhter
Toshihiko Aki
Ryutaro Akiba
Altinbas Akif
Ainur Akilzhanova
Yoritaka Akimoto
Tatiana Akimova
Ibrahim Akin
Ayse Akincigil
Modupe Akinola
Robert Akins
Akanni Akinyemi
Rufus Akinyemi

Tomi Akinyemiju
Lisa Akison
Toshihiro Akisue
Hisanao Akiyama
Kazufumi Akiyama
Masashi Akiyama
Masato Akiyama
Yoshiyuki Akiyama
Anthony Akkari
Derya Akkaynak
Ramesh Akkina
Mustafa Akkiprik
Gokhan Akkoyunlu
Zeynettin Akkus
Melis Akman
Lara Aknin
Belinda Akpa
Patrick Akpaka
Ali Akpinar
Ani Akpinar
Michael Akresh
Nazan Aksan
Nadia Akseer
Michel Akselrod
Hulya Aksoy
Samil Aktas
Ebru Akten
Shamima Akter
Shaw Akula
Tatsuya Akustu
Sercan Akyalcin
Denise Al Alam
Noor Al Dahhan
Ayman Al Haj Zen
Elie Al Kazzi
Abeer Al Masri
Ala-Eddin Al Moustafa
Nashwan Al Naiemi
Julien Al Shakarchi
Olufemi Alabi
Igor Alabugin
Concepción Alados
Parham Alaei
Kaat Alaerts
Mahmoud Alagawany
Faisal Al-Agel
Abraham Al-Ahmad
Outi Ala-Honkola
Tommy Alain
Enateri Alakpa
Mazen Al-Alawi
Ziyad Al-Aly
Asim Alam
Feroz Alam
Khurshid Alam
Mohammad Alam
Nazmul Alam
Shah Alam
Tiina Alamäe
Luis Alameda
Arsham Alamian
Ahmed Al-Amiery
Hassen Al-Amin
Reem Alamoudi
Yassar Alamri
Alexandre Alanio
Gregorio Alanis Lobato
Sarah Alansari
Merve Alanyali
Satish Alapati
Balbino Alarcon
Pablo Alarcon
Francisco Alarcon-Chaidez
Luis Alarcón-Martínez
Maha Al-Asmakh
Imourana Alassane-Kpembi
Tero-Pekka Alastalo
Ana Alastruey-Izquierdo
Juan José Alava
Maryam Alavi
Ali Alawieh
Christina Alba
David Alba
Samir Al-badri
Cecilia Albala
Amaya Albalat
Ricard Albalat
Diego Albani
Maria Albani
Francis Albarede
Frédérique Albarel
Pedro Albarran
Sameer Al-Bataineh
Manal Al-Batanony
John Albeck
Natalia Albein-Urios
Birgit Alber
Angel Alberich
Federico Alberici
Luciane Alberici
Cristina Alberini
Andreas Albers

D. J. Albers
Kathryn Albers
Aurelie Albert
James Albert
Joerg Albert
Mark Albert
Steven Albert
Megan Albertelli
Warren Albertin
Kurt Albertine
David Albertini
Roger Alberto
Arthur Alberts
Simone Albisinni
Maria Albo
Julie Albon
Alessandro Alboresi
Genevieve Albouy
Philippe Albouy
Axel Albrecht
Christiane Albrecht
Daniel Albrecht
Helmut Albrecht
Philipp Albrecht
Randy Albrecht
Adam Albright
Silviu Albu
Rachel Albuquerque
Ulysses Albuquerque
Francisco Alburquerque-Sendín
Maria Alcaide
Francisco Javier Alcain
Myriam Alcalay
Roy Alcalay
Antonio Alcami
Luiz Alcantara
Maria Alcantar-Curiel
José Carlos Alcantud
Luis David Alcaraz
Maria Jose Alcaraz
Domingo Alcaraz-Segura
Lisa Alcock
Pedro Alcolea
Nasser Al-Daghri
Luis Aldámiz-Echevarría
Haider Al-Darraji
Anthony Aldave
Mihaela Aldea
Rodrigo Aldecoa
Kieran Alden
A. P. Aldenkamp
Janet Alder
James Alderman
Robyn Alders
Elisabetta Aldieri
Kimberly Aldinger
Ahmad Al-Dissi
David Aldous
Grace Aldrovandi
Jared Aldstadt
Zane Aldworth
Carlo Aleci
Lucian Alecu
Victor Alegana
Jorge Alegre-Cebollada
Joel Alejandre
Nicolas Alejandre Alba
Fares Al-Ejeh
A. V. Alekseenko
Ivana Aleksic
Farhang Alem
Andre Aleman
Dione Aleman
Julie Aleman
Tomas Aleman
Heliodoro Alemán Mateo
Francisco Alemany
Zewdie Alemu
Jose Ales
Olatunji Alese
Marianna Alesi
Cristiano Alessandri
Guido Alessandri
Francesca Alessandrini
Marie-Christine Alessi
Kirsi Alestalo
Alberto Aleta
Aldeida Aleti
John Aletta
María Alevizaki
Ilias Alevizos
Vasileia-Ismini Alexaki
Alana Alexander
Heather Alexander
James Alexander
Justine Alexander
Karen Alexander
Mallika Alexander
Melannie Alexander
Oettl Alexander
Sarah Alexander
Erik Alexandersson

Joachim Alexandre
Evan Alexandrou
Kirill Alexandrov
Rainer Alexandrowicz
Kharitonenkov Alexei
Oleg Alexeyev
Ulrike Alexiev
Christoph Alexiou
Alexandra Alexopoulou
Athina Alexopoulou
D. Alexopoulus
M. Alfa
Ayman Alfalou
Carlos Alfaro
Michael Alfaro
Abdullah Al-Farraj
R. F. Alfenas
A. Alfieri
Carlo Alfieri
Carolina Alfieri
Roberta Alfieri
Zarko Alfirevic
Yira Alfonso
Mercedes Alfonso-Prieto
Rosa Alfonso-Rosa
Lital Alfonta
Cecilia Algarin
Alicia Algeciras-Schimnich
Metab Al-Geffari
Medhat Al-Ghobashy
Holly Algood
Hadi Al-Hasani
Jacob Al-Hashemi
Albert Alhatem
Mohammed Al-Houqani
Abduladhem Ali
Akhtar Ali
Amjad Ali
Basharat Ali
Declan Ali
Disha Ali
Golestani Ali
Hamid Ali
Liaqat Ali
M. P. Ali
Maruf Ali
Meser Ali
Mir Ali
Muhammad Ali
Nahid Ali
Nasir Ali
Naushad Ali
Parveen Ali
Qurban Ali
Saleem Ali
Simak Ali
Syed Ali
Syeda Ali
Yasmin Ali
Topal Ali Osman
Ricardo Alia
Hadi AliAkbarpour
Stefano Aliani
Juan Carlos Alías Gallego
Lorenzo Alibardi
Hanene Ali-Boucetta
Jesus Alierta
Jesús Alierta
Rosa Aligue
Jonas Alin
Matthew Aliota
Babak Alipanahi
Fatima Ali-Rahmani
Reid Alisch
Malcolm Alison
Noorjahan Banu Alitheen
S. Alivernini
Alessandro Aliverti
Abubakar Aliyu
Gambo Aliyu
Azra Alizad
Patrick Alizai
Mohammed Aljearah
Dhiya Al-Jumeily
S. Aljunid
Karim Alkadhi
Noam Alkan
Nura Alkathiri
Nabil Alkayed
Rob Alkemade
Alisa Alker
Cristina Al-Khalili Szigyarto
Amir Al-Khami
Mohammad Alkhamis
Ahmed Alkhateeb
Souhaila Al-Khodor
Mahmoud Al-Khrasani
Huseyin Alkim
Abbey Alkon
Fowzan Alkuraya
Philippe Allain
Joel Allainguillaume

Jean Pierre Allam
Alison Allan
Andrea Allan
Charlotte Allan
Richard Allan
Sandra Allan
Shailaja Allani
Elias Allara
Karel Allegaert
Eugenia Allegra
Andrew Allegretti
Claudia Allemani
Andrew Allen
Bruce Allen
Bryan Allen
Craig Allen
Daniel C. Allen
David Allen
Edith Allen
Eric Allen
Harriet Allen
James Allen
Jessica Allen
Joanne Allen
John Allen
Joseph Allen
Kara Allen
Lee Allen
Lee-Ann Allen
Mark Allen
Matthew Allen
Melanie Allen
Randy Allen
Sam Allen
Stuart Allen
Will Allen
Charlotte Allender
Matthew Allender
Diane Allen-Gipson
Alberto Allepuz
Elena Aller
Kristina Allers
Sascha Alles
Karina Alleva
Arthur Allignol
Juri Allik
Ginger R. H. Allington
Nathalie Allioli
Andrew Allison
Beth Allison
Ed Allison
Gordon Allison
Jane Allison
Paul Allison
Thomas Allison
Timothy Allison
W. Ted Allison
Tamara Alliston
Jens Allmer
Vanessa Allom
Mouhamad Alloosh
Yves Allory
S. Allouche
Brady Allred
Carl Allwood
Thomas Ally
Almundher Al-Maawali
Juan Almagro
Amy Alman
Abdullah Almansour
Julio Cesar Almanza-Perez
Badar Almarri
Alex Almasan
Alexandru Almasan
Mohammad Hadi Almasi
Piero Almasio
Laura Almasy
Carlos Almeciga-Diaz
Sergio Almécija
Afonso Almeida
Ana Almeida
António Almeida
Carina Almeida
Daniela Almeida
Danilo Almeida
Eduardo Almeida
Erick Almeida
Fausto Almeida
Hugo Almeida
Karen Almeida
Marcio Almeida
Maria Almeida
Maria Ines Almeida
Mathieu Almeida
Quincy Almeida
Rafael Almeida
Raquel Almeida
Raul Almeida
Rodrigo Almeida
Sandra Almeida
Márcia Almeida-De-Macedo
Adiel T. De Almeida-Filho

Hesham Al-Mekhlafi
Conny Almekinders
Nicole Almenrader
Jordi Almirall
Nisreen Al-Moghrabi
Neil Almond
Antoine Almonte
Ibrahim Saker Al-Mssallem
Markus Almstrom
Kristian Almstrup
Hisham Al-Mubaid
Omar Alnachukati
Olutayo Aloba
Anna Maria Aloisi
A. Alomainy
Soha Alomar
Uri Alon
Maya Aloni
Covadonga Alonso
David Alonso
Javier Alonso
Juan Alonso
Juan Carlos Alonso
Leandro Alonso
Sergio Alonso
Todd Alonso
Wladimir Alonso
Flora Alonso Vega
Pablo Alonso-Coello
Marta Alonso-Hearn
Paloma Alonso-Magdalena
Francis Alonzo III
Michael Alosco
Nedal M. Alouran
Haoues Alout
Tamer Alpagot
Benjamin Alper
Hal Alper
Marianna Alperin
Charles Alpers
Luke Alphey
Arno Alpi
Ahmed Al-Qaissi
Ali Alqarni
Thierry Alquier
Chadi Alraies
Abdulla Al-Rawabdeh
Ghada Al-Rawahi
Waddah Alrefai
Ramzi Alsallaq
Mohammed Alsaweed
Eben Alsberg
Mohamed Al-Shabrawey
Mahmoud Al-Sha'er
Rustam Al-Shahi Salman
Ahmad Alshannaq
J. Alspaugh
Marjan Alssema
Mohammed Alsultan
Jane Alsweiler
David Alt
Karen Alt
Kurt Alt
Mary Alt
Silke Alt
May Elfar Altamimi
Jalal-eddin Al-Tamimi
Claudia Altamura
Louis Altamura
Nihal Altan-Bonnet
Raffaele Altara
Chiara Altare
William Altemeier
Ammar Altemimi
Adrian Altenhoff
Adam Alter
David Alter
Orly Alter
Ronnie Alterman
Eric Altermann
Mike Althaus
David Althoff
Benjamin Althouse
Omar Althuwaynee
Craig Altier
Andrew Altieri
Nicholas Altieri
Ronn Altig
Sefa Altikat
Can Altinelataman
Eyup Altinoz
Ibrahim Altiparmak
John Altman
Andre Altmann
Daniel Altmann
Michael Altmann
Claudio Altomare
D. F. Altomare
Eric Alton
Stephen Altschul
Richard Altschuler
Umar Al-Turki

Kishore Alugupalli
Joshi Alumkal
Luigi Alvarado
Patricia Alvarenga
Veronica Alvarenga
Belén Alvarez
Carlos Alvarez
Carmen Paula Alvarez
Elvira Alvarez
Eva Alvarez
F. Javier Alvarez
Fernando Alvarez
Javier Alvarez
Julio Alvarez
Mario Alvarez
Nadir Alvarez
Susana Alvarez
Xavier Alvarez
Mónica Álvarez
Esteban Álvarez Fernández
Jose Alvarez Ramirez
Esther Alvarez-Ayuso
José Álvarez-Castillo
Damiana Álvarez-Errico
Daniel Alvarez-Fischer
Juan Raúl Alvarez-Idaboy
Carmen Alvarez-Lorenzo
Juan Carlos Alvarez-Pizarro
Gloria Alvarez-Sola
Gerardo Alvarez-Uria
Alejandro Alvaro-Meca
Jonathan Alvarsson
Andrew Alverson
Cresio Alves
Davi Alves
Estefania Alves
Joao Alves
Nuno Alves
Paula Alves
Rômulo Alves
Sandra Alves
Soniza Alves Leon
Maria Virgínia Alves Martins
Ayesha Alvi
Amjad Alwaal
Abdelrahman Aly
Hussein Aly Ibrahim
Mohammad Alyami
Akram Alyass
Irina Alymova
Salah S. Al-Zaiti
Naweed Alzaman
Munir Al-Zeer
Christian Alzheimer
Marialaura Amadio
Peter Amadio
Cristian Amador
Omar Amador-Muñoz
Stefano Amaducci
Marcos Amaku
Mary Amalaradjou
René Amalberti
S. Amalfitano
Placidus Amama
M. Javad Aman
Malin Åman
Derek Amanatullah
Praveen K. Amancha
Diego Amancio
Rodrigo Amancio
M. Javad Amanlou
Hirofumi Amano
Mariane Amano
Frederic Amant
Robert Amant
Claudio Amanti
Amanullah Amanullah
Ahmad Amanzada
Andre Amaral
Fernanda Amaral
Flavio Amaral
Karina Amaral
Leonard Amaral
Lucas Amaral
Sandra Amaral
David Amarantini
Alberto Amato
Katherine Amato
Patricia Amavet
Peter Amaya
Padma Ambalam
Bala Ambati
Atul Ambekar
Alemayehu Amberbir
Sandeep Amberkar
Satyajit Ambike
David Amblas
Marianna Amboni
Elisa Ambrosi
Concetta Ambrosino
Giuseppe Ambrosio
Santiago Ambrosio

Christian Ambrosius
Christine Ambrosone
Slawomir Ambroziak
Indu Ambudkar
Evans Ameade
Shazhan Amed
Oluwatoyin Ameh
Daniela Amelio
Agustina Amelong
Kei Amemiya
Sarah Amend
Peter Amenta
Amal Amer
Hatem Amer
Kurosh Ameri
Mahmoud Ameri
Pietro Ameri
Gregory Ames
Nancy Ames
Adam Ameur
Eran Amichai
Patrizia Amico
Rivet Amico
Manuel Amieva
Marcel Amills
Amr Amin
Muhammed Amin
Raid Amin
Rajesh Amin
Ruhul Amin
Keyvan Amini
N. Aminisani
Mahmoud Aminlari
Mamuda Aminu
Saeed Aminzadeh
Lisa Amir
Shimon Amir
Israel Amirav
Alireza Amirbaigloo
Steve Amireault
E. Amiri
Esmaeil Amiri
E. Amirian
Ilda Amirian
George Amiridis
Mohammadtaghi Amiri-Khorasani
Yuri Amirkhanian
Evžen Amler
David Ammar
El-Desouky Ammar
Anne Ammerdorrfer
Enrico Ammirati
Sylwia Ammoun
Luisa Amo
Linda Amoah
Fiifi Amoako Johnson
Winfried Amoaku
Alongkorn Amonsin
M.A. Amoozegar
Rumelo Amor
Alessandro Amore
M. Clara Amorim
Vittoria Amorosi
Antonio Amoroso
Mariano Amoroso
Ricardo Amoroso
Mohammad Javad Amoshahy
P. Amouroux
Agbessi Amouzou
Yiannis Ampatzidis
Neil M. Ampel
Charles Ampomah-Dwamena
Ruchanee Ampornaramveth
Javier Ampuero
Julia Ampuero
Sandra Ampuero
Abdelaziz Amrani
Fouad Amraoui
Karin Amrein
Hakima Amri
Bharadwaj Amrutur
Charles Amsler
Kurt Amsler
Adam Amsterdam
Franklin Amthor
Helge Amthor
Sylvie Amu
John Amuasi
Laufey Amundadottir
Mario Amzel
Baiguo An
Bang An
Cheng-Bang An
Chunju An
Diaoguo An
Dingding An
Gang An
Gary An
Hailong An
Haizhong An
Hongyu An
Jing An

Lihui An
Lingling An
Nan An
Ruopeng An
Tong-Qing An
Wenfeng An
Zheng An
Allison Anacker
Christelle Anaclet
Jose Anadon
Pinelopi Anagnostopoulou
Nicholas Anagnou
Deepika Anand
Shuchi Anand
Tarandeep Anand
Madhu Anand-Srivastava
Meena Ananthanarayanan
Jaithri Ananthapavan
Kumudha Anantharajappa
Karthik Anantharaman
Bharath Ananthasubramaniam
Nantheera Anantrasirichai
Sofia Anastacio
Erin Anastasi
Panos Anastasiadis
Dimi Anastasiadou
Thomas Anastasio
Natasa Anastasov
Kathryn Anastos
Vikas Anathy
Antonio R. Anaya
Muralidharan Anbalagan
Kumarasamy Anbarasu
Ana Lia Anbinder
Periasamy Anbu
Beau Ances
Anu Anchala
Tom Anchordoguy
Ellen Anckaert
Petronela Ancuta
Marcus And
Elissar Andari
Nicole Andeen
Martina Andellini
Leander Anderegg
John Anderies
Christine Anderl
Mark Andermann
Miroslava Anderova
Hans-Joachim Anders
Juanita Anders
Alan Andersen
Asger Andersen
Barbara Andersen
Birgitte Andersen
Bogi Andersen
Erik Andersen
Hans-Erik Andersen
Kelly Andersen
Kenneth G. Andersen
Lars Andersen
Marc Andersen
Mark T. Andersen
Niels Andersen
Peter Andersen
Vibeke Andersen
Frank Andersohn
Alex Anderson
Alisha Anderson
Alison Anderson
Alyssa Anderson
Amy Anderson
Annette Anderson
Barbara Anderson
Benjamin Anderson
Burt Anderson
C.S. Anderson
Carl Anderson
Carol Anderson
Charles Anderson
Craig Anderson
Cynthia Anderson
David Anderson
David C Anderson
David P. Anderson
Deborah Anderson
Douglas Anderson
Eric Anderson
Ethan Anderson
George Anderson
Greg Anderson
Gregory Anderson
Harvey Anderson
James Anderson
Jeffrey Anderson
Joel Anderson
John Anderson
Judy Anderson
Justin Anderson
Karen Anderson
Karl Anderson
Kevin Anderson

Kirk Anderson
Kristin Anderson
Kurt I. Anderson
Larry Anderson
Laura Anderson
Lynda Anderson
Mark Anderson
Nigel Anderson
Olivia Anderson
Paul Anderson
Per Anderson
Rachel Anderson
Randy Anderson
Richard Anderson
Robert Anderson
Roger Anderson
Ronald Anderson
Ross Anderson
Samira Anderson
Sharolyn Anderson
Thomas Anderson
William Anderson
Ann Anderson-Berry
Cay Anderson-Hanley
Kristina Anderson-Teixeira
Anna-Maria Andersson
Eva Andersson
Jesper Andersson
Leif Andersson
Linus Andersson
Magnus Andersson
Matthew Andersson
Ola Andersson
Patiyan Andersson
Sven Andersson
Ann-Christine Andersson Arnten
Pia Anderwald
Denise Andia
Isabel Andia
Anuska Andjelkovic
Hideya Ando
Keita Ando
Tatsuro Ando
Annapaola Andolfo
Pulung Nurtantio Andono
Magi Andorra
Adriana Andrade
Angel Andrade
Claudia Andrade
Danielle Andrade
Denis Andrade
Francisco Andrade
Gabriel Andrade
Gustavo Andrade
Leandro Andrade
Luciano Andrade
Marcelo Andrade
Rosa Andrade
Sonia Andrade
José Dilermando Andrade Filho
Johanna Andrae
Ibolya Andras
Michele Andrasik
Prabha Andraweera
Michael Andrawes
Christelle Andre
Emilie Andre
Nicolas Andre
Kostas Andreadis
Elisabeth Andreadou
Ioanna Andreadou
Sharon Andreason
Ole Andreassen
Erik Andreasson
Massimo Andreatta
Eran Andrechek
Silvana Andreescu
Adin-Cristian Andrei
Michel Andrejak
Antoine Andremont
Felipe Andreo
Demetra Andreou
Brian Andres
Douglas Andres
Emmanuel Andres
Fernando Andres
Katrine Andresen
Jaan-Olle Andressoo
Ines Andretta
Michele Andreucci
Angeline Andrew
Balmford Andrew
Deborah Andrew
Jennifer Andrew
Christina M. Andrews
Elizabeth Andrews
Gavin Andrews
Joel Andrews
Nick Andrews
Russell Andrews
Simon Andrews
Steve Andrews

Thomas Andriacchi
Ramaroson Andriantsitohaina
Silke Andrich
Adriano Andricopulo
Luca Andrighetto
Thomas Andrillon
Jerry Andriole
Ion C. Andronache
Ovidiu Andronesi
E Andronov
Hagen Andruszkow
Stepulak Andrzej
Uduak Andy
Ritu Aneja
Uchenna Anele
Simone Anfossi
C. Wim Ang
Frederic Ang
Put Ang
Mulugeta Angamo
Michael Angarone
Narayanasamy Angayarkanni
Catherine Angel
Hui Angela Bik-Yu
Kalliopi Angelakis
Gustavo Angeles
Ricardo Angeles
Manuel Angeles-Castellanos
Andrea Angeletti
Dario Angeletti
Silvia Angeletti
Claudia Angeli
Elena Angeli
Apostolos Angelidis
Riccardo Angelini
Tommaso Angelone
Samer Angelone-Alasaad
Maria Angelova
Plamena Angelova
Diego Angelucci
Klaus Anger
Martin Anger
Karin Angerud
Kenneth Angielczyk
Francesca Angileri
Claudio Angione
Stefano Angioni
Trinidad Angosto
Nicole Angotti
Denis Angoulvant
Alessandro Angrilli
Tiziana Angrisano
Delphine Angst
Montserrat Anguera
Eduardo Anguita
Juan Anguita
Javier Angulo
Brian Angus
Bradley Anholt
Luis Anibarro
Andrea Anichini
Cuneyd Anil
Veena Anil
Gifty Aninanya
Anahita Aniri
Lesya Anishchenko
Andrey Anisimov
Joël Ankri
Pieter Annaert
Damodaran Annamalai
Christopher Annear
Justin Annes
Vito Annese
James Annesi
Brian Annex
Rajeev Annigeri
Tarmo Annilo
Giorgio Annoni
Takeshi Annoura
Clorinda Annunziata
Yasuhisa Ano
El Hassane Anouar
Arya Ansari
Athar Ansari
Daniel Ansari
Mairaj Ansari
A. Anselmi
Stéphane Ansieau
Hermann Ansorge
Nicholas Anstey
Caterina Ansuini
Rashid Ansumana
Balint Antal
Toni Antalis
Yaron Antebi
Haike Antelmann
Niels Anten
Francisco Antequera
Juliana Antero-Jacquemin
Alison Antes
Fabien Anthelme
Robert Anthenelli

Donald Anthony
Douglas Anthony
Lisa Anthony
Richard Anthony
Robert Anthony
Tracy Anthony
Tamil Selvan Anthonymuthu
Fabrice Antigny
Abigail Antigua
Joseph Antin
Marlies Antlanger
Thessicar Antoine
Sophie Antoine-Jonville
Jan Antolik
Michael Antolin
Salome Antolin
Sylvia Anton
Alessandro Antonelli
Francesca Antonelli
Guido Antonelli
Patrick Antonelli
Costin Antonescu
Charalambos Antoniades
Chrystalina Antoniades
Demetris Antoniades
Maciek Antoniewicz
Angelo Antonini
Sonir Antonini
Marco Antoniotti
Kyriakos Antoniou
Maria Antoniou
Gunther Antonissen
Boyka Antonova
Emilia Antonucci
Ivana Antonucci
Bhavna Antony
Binu Antony
Justin Antony
Veena Antony
Kevin Antshel
Flora Antunes
Leonardo Antunes
Maisa Antunes
Paula Antunes
Ainur Anuar
Dilly Anumba
Justus M. Anumonwo
Krishnamurthy Anupama
M. M. Anwar
Muhammad Anwar
Kenneth Anyomi
Naohiko Anzai
Pavel Anzenbacher
Eva Anzenbacherova
Hushan Ao
Mingxin Ao
Tomohiko Aoe
Takashi Aoi
Kazuhiro Aoki
Koh Aoki
Naohiro Aoki
Reiko Aoki
Takatoshi Aoki
Yuta Aoki
Miguel Aon
Yoichi Aota
Nassera Aouali
Satoka Aoyagi
Takuma Aoyama
Fernando Aparici
Anca Apatean
Bettye Apenteng
Lionel Apetoh
Vania Apkarian
Richard Aplenc
Andrew Aplin
John Aplin
Lucy Aplin
Gerard Apodaca
Daniel Apolinario
John Apolzan
Emilia Apostolova
I. Apostolova
Florence Apparailly
Art Appel
Emil Appel
Rudi Appels
Ruth Appeltant
Charles Apperson
Bernard Appiah
Duke Appiah
Brian Appleby
Peter Appleby
Tanya Applegate
Alison Appling
Matthew Apps
Ivan Aprahamian
Michael April
Francesco Aprile
William Apro
Alexander Apt
Swapna Apte

Udayan Apte
Alan Apter
James Aquavella
Guillermo Aquino-Jarquin
Ignacio Ara
Hany Arab
Marzieh Araban
Mohammad Arabestani
Asma Arabi
Milad Arabloo
Mallikarjuna Aradhya
Chinmay Aradhye
Amor Aradilla-Herrero
Naoko Aragane
Irene Aragao
Henio Aragão
Pablo Aragón
Pedro Aragón
Nuria Aragones
Hidenori Arai
Ken Arai
Toshiro Arai
Kimika Arakawa
Satoko Arakawa
Hitoshi Araki
Michihiro Araki
Yasuto Araki
Elisa Araldi
Spyridon Arampatzis
Pornanong Aramwit
Dvir Aran
Alicia Aranaz Martin
Agustin Aranda
Manuel Aranda
Mimi Arandjelovic
Carlos Arango
Fabrizio Araniti
Iker Aranjuelo
Praveen Arany
Bekir Aras
Siddhesh Aras
Hisashi Arase
Daniele Araujo
Edson Araujo
F.G. Araujo
Flabio Araujo
Mariana Araujo
Natalia Araujo
Nuno Araujo
Pedro Araujo
Ricardo Araujo
Ronaldo Araujo
André Araújo
Francisca Araújo
P. M. Araújo
Raimundo Araújo Júnior
Theo Araujo-Santos
David Araújo-Vilar
Ravit Arav-Boger
Shigeki Arawaka
Azizollah Arbabisarjou
Armin Arbab-Zadeh
Konstantin Arbeev
Ronen Arbel
Yael Arbel
Yaron Arbel
Jonathan Arbelle
Michal Arbel-Ornath
Nadir Arber
Caroline Arber Barth
Adrià Arboix
Joseph Arboleda-Velasquez
Vicente Arbona
Jose Arbones-Mainar
Laura Arbour
Benjamin Arbuckle
Patrick Arbuthnot
Luca Arcaini
Elsa Arcaute
Agustín Arce
Annie Archambault
Derseree Archary
Amena Archer
Edward Archer
G. Archer
Simon Archer
Stephanie Archer
Stuart Archer
Will Archer
Lisa Archibald
Sotirios Archontoulis
Govindaraju Archunan
Biagio Arcidiacono
Nicola Arcilla
Kimberly Arcoleo
Carmen Ardanuy
Reza Ardehali
Jeffrey Ardell
Gemma Arderiu
Alex Ardila-Garcia
Leopoldo Ardiles
Moshe Arditi

Navid Ardjomand
Estela Area
Francisco Areal
Ana Paula Arêas
Ignacio Arechaga
Pedro Aredes
Yupaporn Areepong
Maria Emília Guimarães Areias
Jon Arellano
Lucrecia Arellano
Claudia Arenaccio
Miguel Arenas
Francisco Arenas-Huertero
Juan Arenas-Jimenez
Cesar Arenas-Mena
Alejandro Arenas-Pinto
Douglas Arenberg
Florian Arendt
Gabriele Arendt
Josephine Arendt
Lisa Arendt
Maja Arendt
A. Katrin Arens
Philipp Arens
Maria-Angeles Arevalo
Katiushka Arévalo- Niño
Angel Arevalo-Martin
Cynthia Arfken
Frank Arfuso
Juan Arganaraz
Pedram Argani
Giuseppe Argenziano
Antonio Argiolas
Charles Argoff
Nilay Argon
Yair Argon
Ariadne Argyraki
Anthoula Argyri
Christos Argyropoulos
Paraskevi Argyropoulou
Kristopher Arheart
Adrian Arias
Natalia Arias
Pablo Arias
Renee Arias
Sandra Arias
Maria Arias Alvarez
Jose Arias-Estero
Tatiana Arias-Garzon
Jose-Antonio Arias-Montaño
Gil Ariel
Frederic Ariey
Mohammad Arif
Wan Nor Arifin
Damla Arifoglu
Cecilia Arighi
E. Arima
Taro Arima
Kohki Arimitsu
Gen-ichiro Arimura
Takuro Arimura
Yoanna Ariosa-Morejon
Robert Aris
Anna Arís
Stephane Aris-Brosou
Mikio Arita
Olli Arjamaa
Bahram Arjmandi
Nikolaos Arkadopoulos
Hendrik-Tobias Arkenau
Marco Arkesteijn
Robert Arkowitz
Guillaume Arlet
Sonke Arlt
Guillermo Armaiz
Rubén Armañanzas
Laurence Armand-Lefevre
Olivier Armant
Jonathan Armbruster
Jean Armengaud
Eleni Armeni
I. Armentano
Maickel Armenteros
Paul Armistead
Charles Armitage
Kenneth Armitage
John Armour
William Armstead
Ben Armstrong
Daniel Armstrong
David Armstrong
Elizabeth Armstrong
Eric Armstrong
Gregory Armstrong
Irene Armstrong
J. Armstrong
Jennifer Armstrong
Mary Armstrong
Nicola Armstrong
Paul Armstrong
Susan Armstrong
Wendy Armstrong

Arunmozhiarasi Armugam
Fernanda Arnaldez
Rima Arnaout
George Arnaoutakis
Nicolas Arnaud
Saskia Arndt
Miquel Arnedo
Jonas Arnemann
Alain Arneodo
J.D. Arneodo
Erik Arner
Luis Arnes
Tim Arnett
Birgit Arnholdt-Schmitt
Johan Arnlov
Steven Arnocky
Allison Arnold
Andrea Arnold
Antje Arnold
Corey Arnold
Forest Arnold
H. Arnold
John Arnold
Mark Arnold
Matthias Arnold
Melina Arnold
Robert Arnold
Solvi Arnold
Todd Arnold
W. Arnold
Will Arnold
Wolfgang Arnold
Maria Arnone
David Arnosti
Jon Arnot
Grady Arnott
Christophe Arnoult
Damien Arnoult
Samer Arnous
Aapo Aro
Hannu Aro
Ricardo Aroca
Freddy Arocha
Luiz Aroeira
Anna Arola-Arnal
David Aronoff
Maria Aronova
Bruce Aronow
Peter Aronow
Wilbert Aronow
Kristan Aronson
Myla Aronson
Ashish Arora
Manish Arora
Rajeev Arora
Sandeep Arora
Teresa Arora
Oneida Arosarena
Joe Arosh
Paolo Arosio
Asaithamby Aroumougame
Salvatore Arpaia
Johannes Arpegård
Miguel Angel Arrabal-Polo
Amaia Arranz-Otaegui
Soumaya Arraouadi
Gloria Arratia
Abdelilah Arredouani
Sergio Arregui
Marco Arrese
Agnes Arrey
Protus Arrey Tarkang
Edgar Arriaga
Francisco Arriaga
Alberto Arribas
Joaquín Arribas
Yoan Arribat
Alessandro Arrigo
Haritz Arrizabalaga
Joseph Arron
Alison Arrow
Juan Arroyo
Rosa Arroyo-García
Manuel Arroyo-Kalin
E.F. Arruda
Eurico Arruda
Luciana Arruda
Marco Antonio Arruda
Paulo Arruda
Elisabet Ars
Benoit Arsenault
Jelena Arsenijevic
Hassan Arshad
Qadeer Arshad
Vadim Arshavsky
Biljana Arsic
Salim Arslan
Zikri Arslan
Ayca Arslan-Ergul
Emilio Artacho-Pérula
Pablo Artal
Irma Arteaga

Gavin Arteel
Kyle Artelle
Panagiotis Artemiadis
Joaquín Artés
Hilana Artese
Angela Arthington
James Arthos
Agnes Arthur
Eric Arthur
J. Simon Arthur
Janelle Arthur
Michel Arthur
Terrance Arthur
Christopher Arthurs
Pablo Artigas
Katharina Artinger
Guilherme Artioli
Carol Artlett
Isabella Artner
Adriana Artola
Ferruh Artunc
Per Artursson
Augustine Arukwe
Velmurugesan Arulampalam
J.P. Arulanadam
Bernard Arulanandam
Baskar Arumugam
Paritha Arumugam
Sasi Arunachalam
J. Arunakaran
Anshu Arundhati
Willy Arung
Shalini Arunogiri
Mardjan Arvand
S. Arvedsen
Patrick Arveux
Cindy Arvidson
Sheila L. Arvikar
Henk Arwert
Rector Arya
Krishna Aryal
Juvid Aryaman
Kadek Y.E. Aryanto
Okailey Aryeetey
Shahar Arzy
Wael Asaad
Nana Asabere
Abu Bakar Ali Asad
Zahra Asadi
M. Niaz Asadullah
Md. Asaduzzaman
Michio Asahi
Kinji Asahina
Yasuhiro Asahina
David Asai
Manabu Asai
Sneha Asai
Yusuke Asai
Akihiro Asakawa
Satoshi Asakura
Tomiko Asakura
Atsushi Asano
Kenichi Asano
Kenichiro Asano
Tsunaki Asano
Kwaku Asante
Yasuhide Asaumi
Kei Asayama
Penny Asbell
Jakob Asberg
Manuel Ascano Jr.
Juan Ascaso
Felipe Ascencio
Rebecca Asch
Frank Asche
Dana Ascherman
Pablo Aschner
Phillipa Ascough
Marina S. Ascunce
Karim Asehnoune
Javier Asensio
Norberto Asensio
Maryam Asgari
Sassan Asgari
Ali Asgary
Mohammad Asghar
Hafez Asgharzadeh
Christopher Ash
Dipankar Ash
Yacov Ashani
Gregory Ashby
Michael Ashby
Nathan Ashby
Mark Ashcraft
Mark Ashe
Robert Asher
Phil Asherson
Muhammad Ashfaq
Rebecca Ashfield
Michael Ashford
Motoyuki Ashikari
Muhammad Waseem Ashiq

Tetsuo Ashizawa
Ali Ashkarran
Elizabeth Ashley
Gary Ashley
Patricia Ashley
Paul Ashley
Ryan Ashley
Helen Ashman
Jonathan Ashmore
Sarah Ashmore
Ulla Ashorn
Osama Ashour
Elham Ashouri
Ambika Ashraf
M. Ashraf
Mohammad Ashraf
Nasheeman Ashraf
Zaman Ashraf
Amit Ashtekar
Anthony Ashton
Neil Ashton
Ruth Ashton
Patricia Ashton-Prolla
Eran Ashwal
Margaret Ashwell
Mark Ashwell
Osman Asicioglu
Laureano Asico
Michael Asiedu
Abdul R. Asif
Akhtar Rasool Asif
Mehar Asif
Salman Asif
Mohammad Asim
Fotis Asimakopoulos
M.J. Asins
Ninitha Asirvatham-Jeyaraj
Kjetil Ask
David Askew
Eldon Askew
Maria Askmyr
Muhammad Aslam
Parisa Aslani
Oleg Aslanidi
David Asmuth
Laura Asnaghi
Yoshimasa Aso
Oluwatoyin Asojo
Aravind Asokan
Ahmad Asoodeh
Kewal Asosingh
Sara Aspberg
Per Aspenberg
Mauricio Aspé-Sanchez
Peter Aspinall
Mark Asplen
Linnéa Asplund
Kelly Asquith
Mark Asquith
Michael Assaf
Nazila Assasi
Gianni Assenza
Elisa Assirelli
Amanda Assis
Rafael L. Assis
Lorenz Assländer
Julian Assmann
Ayalew Astatkie
Mariana Astiz
Christopher Aston
Todd Astorino
Loukas Astrakas
Nolwenn Astruc
Giovanni Astuti
Rini Astuti
Nozomu Asukai
Oskar Aszmann
Attila Aszodi
Kamran Atabai
Ricardo Ataíde
Joel Atallah
Sergei Atamas
Kalina Atanasova
Santosh Atanur
Ryuichiro Atarashi
Siavash Atashgahi
Beste Atasoy
Hilal Atasoy
Ulus Atasoy
Michael Atchison
Nazli Atefi
Jelle Atema
Funda Ates
Dimitrios Athanasakis
Beopoulos Athanasios
Vidya Athreya
V.G. Athyros
Ahmed Atia
Levent Atici
Yacine Atif
Canan Atilgan
Emre Atilgan

Gul Atilla
William Atiomo
Nadia Atiya
Emily Atkins
G. Brandon Atkins
Janice Atkins
Katie Atkins
Marc Atkins
Timothy Atkins
Greg Atkinson
Jeffrey Atkinson
John Atkinson
Larry Atkinson
Lou Atkinson
Lucy Atkinson
Mark Atkinson
Michael Atkinson
Peter Atkinson
Prescott Atkinson
Quentin Atkinson
Samuel Atkinson
Sarah Atkinson
Stephanie Atkinson
Stephen Atkinson
Ella Atlas
Reynir Atlason
Robert Atmar
Andrej Atrens
Chintamani Atreya
José Joaquín Atria
Sílvia Atrian
Piray Atsak
Masanori Atsukawa
Tatsuya Atsumi
Atsushi Atsushi Miyamoto
Yoshiko Atsuta
Mohamed Atta
Geoffrey Attardo
Hrayr Attarian
Giovanna Attene
Alan Attie
Adolfo Francesco Attili
Liliana Attisano
Kuldeep Attri
Mukundan Attur
Stephen Attwood
Dan Atwater
Suzi Atwell
Lukoye Atwoli
Walter Atwood
Gil Atzmon
Marco Atzori
Alma Au
Jacky Au
Kin Fai Au
Kit Sing Au
Whitlow Au
Shiu Lun Ryan Au Yeung
Geraldine Aubert
Lyderic Aubert
Mylene Aubertin-Leheudre
Patrick Aubourg
Anael Aubry
Sarah Auburn
Amy Auchincloss
Apolline Auclerc
Jean-Julien Aucouturier
Jean-Nicolas Audet
Jonathon Audia
François Audibert
Stéphane Audic
Annette Audige
Lívia Audino
Riccardo Audisio
Rachel Audo
Michel Audran
Asta Audzijonyte
Tatjana Aue
Christian Auer
Manfred Auer
Matthias Auer
Joshua Auerbach
Judith Auerbach
Michael Auerbach
Randy Auerbach
Scott Auerbach
Wojtek Auerbach
Irena Auersperger
Karl Auerswald
Ulrich auf dem Keller
Eva Auffenberg
Christoph Aufricht
Christopher Auger
Nathalie Auger
Martin Augsten
Furaha August
Albert Auguste
Debra Auguste
Hellmut G. Augustin
Antonios Augustinos
Danillo Augusto
Jean-François Augusto

Leonardo Augusto
Rosângela Augusto
Gaius Augustus
Andrew Auld
Megan Auld
James Auman
Dagfinn Aune
Lynn Aung
Myo Nyein Aung
Thet Aung
Timothy Aungst
Camille Aupiais
Robin Aupperle
Pascal Auquier
Filippo Aureli
Massimo Aureli
Dumetre Aurelien
Christine Aurich
Vincenzo Aurilia
Igor Aurrekoetxea
Malcolm Ausden
Alessio Ausili
Brandon Ausk
Laurent Aussel
Emily Austen
Peter Auster
Bradley Austin
Bryn Austin
Caroline Austin
Chris Austin
Elizabeth Austin
Patrick Austin
Riccardo Autelli
Stella Autenrieth
Chantal Autexier
Andrea Auther
Matthieu Authier
Timo Autio
David Autor
Joseph M. Autry
Bertran Auvert
Malika Auvray
Paul Auwaerter
Sylive Auxilien
Narayan Avadhani
Paul Avan
Aurore Avargues-Weber
Fikri Avci
Selmir Avdic
Theresa Aveling
Juan Antonio Avellana
Carolina Avellaneda
Sergey Averkin
Antonio Aversa
David Avery
Margaret Avery
Tal Avgar
Avi Avi Mendelsohn
Jeremy Avigad
David Avigan
Gonzalo Avila
Viridiana Avila
Conxita Avila Escartin
Luis Avila-Cabadilla
Almudena Avila-Fernandez
Santiago Avila-Rios
German Avila-Sakar
Alejandro Avilés-Reyes
Marcelino Aviles-Trigueros
Richard Aviv
Selin Aviyente
Tomer Avni
Angelo Avogaro
Elisa Avolio
Judith Avrahami
Alexandru V. Avram
Dorina Avram
Michael Avram
Evangelia Avramidou
Alexis Avril
Anna Avrova
Joseph Avruch
Dimitrios Avtzis
Enrico Avvedimento
Swee Aw
Tiong Aw
Mark Awad
Mona Awadalla
Saria Awadalla
Ahmed Awadein
Ahmed Awaisu
Amit Awasthi
Mukesh Kumar Awasthi
Shanjana Awasthi
Suyash Awate
Taiwo Awolola
David Axelrod
Jacob Axelsen
Pia Axemo
Iben Axen
Jordan Axt
Michael Axtell

Cihan Ay
Ferhat Ay
Takanori Ayabe
Alfred Ayala
Juan Ayala
Julio Ayala
Marcela Ayala
Yuna Ayala
Fernando Ayala-Zavala
Manfred Ayasse
Muhammad Ayaz
Suleyman Aydin
Koksal Aydinsakir
Tandy Aye
Yimon Aye
Adejumoke Ayede
Olufemi Ayeni
Don Ayer
Duncan Ayers
John Ayers
Tracy Ayers
Mualla Aylin Arici
Juan Ayllon
Segolene Ayme
Maria Aymerich
Ashraf Ayoub
Nadia Ayoub
Outi Ayras
Emira Ayroldi
Qasim Ayub
Miriam Ayuso
Muavviz Ayvaz
Murali-Mohan Ayyanath
Abul Azad
Neelam Azad
Mahan Azadpour
Hela Azaiez
Taj Azarian
Ana Raquel Azevedo
Hatylas Azevedo
Maria Manuel Azevedo
Nuno Azevedo
Ruben Azevedo
Ricardo Azevedo Pereira
Ali Azghani
Shafquat Azim
Helena Azinheira
Moammir Aziz
Zahra Aziz
Siti Azizah
Kamalrul Azlan Azizan
Bahareh Azizi
Fereidoun Azizi
Zahra Azizi
Jose Aznar-Moreno
Joseph Azok
Daniel Azoulay
Philippe Azouvi
Elie Azria
Eiichi Azuma
Kazuo Azuma
Kenichi Azuma
Mizuki Azuma
Toshifumi Azuma
Iskandar Azwa
Claus Azzalin
Alberta Azzi
Ricardo Azziz
Eduardo Azziz-Baumgartner
Roberto Azzoni
Yi Ba
Zhaoyu Ba
Joshua Baalwa
Mauhamad Baarine
Esther Baart
Dolgor Baatar
Harald Baayen
Hideo Baba
Reizo Baba
Yuichi Baba
Fatima Baba-Ari
Masoud Babaei
Sylvie Babajko
Lamine Baba-Moussa
Babak Baban
Sankhiros Babapoor
José Babarro
Tetsuya Babazono
Shashi Babbar
Charles Babbs
Elizabeth Babcock
Russell Babcock
Molly Babel
Vladimir Babenko
Usman Baber
Igor Babiak
Fawzi Babiker
Doreen Babin
E. Babirekere-Iriso
Juliet Babirye
John Babish
Jodie Babitt

Sudhir Babji
B. Kalyana Babu
Giridhara R. Babu
M. Madan Babu
Maya Babu
Mohan Babu
Subash Babu
Petr Babula
Ozgun Babur
Özgün Babur
Ana Baburamani
Andy Babwah
Terence Babwah
Michael Babyak
Mark Baccei
Elena Bacchelli
Alberto Baccini
Olivia Bacellar
Alex Bach
Dominik Bach
Erika Bach
Eviatar Bach
Lennart Bach
Thomas Bach
Ketema Bacha
Justyna Bachanek
Abdulgafoor Bachani
Benedicte Bachelot
Daniela Bacherini
Eric Bachman
Sara Bachman
Oliver Bachmann
Talis Bachmann
Alexander Bachmanov
Abdo Bachoura
William Bachovchin
Csanad Bachrati
Adam Bachstetter
Leonardo Bacigalupe
Emma Bäck
Daniel Backenroth
Wayne Backes
H. Backman
Samuel Backman
Donald Backos
Richard Backs
Jeffrey Backstrand
Martin Bäckström
Lucinda Backwell
Charles Bacon
Aline Bacurau
T. Baczek
Michael Bada
Ekaterina Badaeva
Harun Badakhshi
Alexander Badamchi-Zadeh
Luigi Badano
Jerome Badaut
Harry Baddour
Ildiko Badea
Kerime Bademli
Lindsey Baden
Tom Baden
Dan Bader
Gary Bader
Oliver Bader
Viorel Badescu
Rajendra Badgaiyan
Brian Badgley
Leonardo Badia
Roger Badia
Behnam Badie
Anand Badigannavar
Lina Badimon
Norman Badler
Anna Badner
Aristide Bado
Ruchi Badola
Pierre-Marie Badot
Hélène Badouin
Gamal Badr
M. Badr
Hassan Badrane
Nasem Badreldin
Petr Badura
Rajendra Badwe
Stephen Badylak
Hyunsu Bae
Jeehyeon Bae
Jin-Woo Bae
S.S. Bae
Sang Kyun Bae
Sang-Cheol Bae
Sangsu Bae
Soon Wha Bae
Taeok Bae
Yun Soo Bae
Leslie Baehr
Wolfgang Baehr
Chong Wha Baek
Kwang-Hyun Baek
Seungik Baek

Jonathan Baell
Stephanie Baello
Alan Baer
Charles Baer
Richard Baer
Felix Baerlocher
Scott Baerson
Leen Baert
C. Baes
Jared Baeten
J. Antonio Baeza
Mona Bafadhel
Sumit Bag
Omar Bagasra
Amitabha Bagchi
Anindya Bagchi
Milan Bagchi
Soyhan Bagci
Fran Bagenal
Umesh Bageshwar
Deepika Bagga
Sumedha Bagga
Keith Baggerly
Giosue Baggio
Jacopo Baggio
Rodolfo Baggio
Matthew Baggott
Haleh Bagheri
Majid Bagheri
Samira Bagheri
Zahra Bagheri
Mohammad Baghery
Cedo Bagi
Emmanouil Bagkeris
Demetrius Bagley
Anne-Marie Bagnall
Sergio Bagnato
Emilia Bagnicka
Paola Bagnoli
Teresa Bago d'Uva
James Bagrow
Andrew Bagshaw
Elisabeth Bagshaw
Subhash Bagui
Thomas Baguley
Jennifer Bagwell
Laila Bahaa-el-Din
Mohammadkarim Bahadori
Ranjit Bahadur
Ahmed BaHammam
Luis Bahamondes
Ofir Bahar
El Mustapha Bahassi
Badrulhisham Bahazador
Nadia Bahi-Buisson
Suhad Bahijri
Justin Bahl
Vasundhra Bahl
Christie Bahlai
L. Charles Bahler
R.C. Bahler
Hisham Bahmad
Thomas Bahmer
Volker Bahn
David Bahner
Chelsea Bahney
Angela Bahns
Bahram Bahrambeigy
Hossein Bahrami
Soheyl Bahrami
Mohamed Ali Bahri
Mahnaz Bahri Khomami
Gurpreet Baht
Jyotika Bahuguna
Paluku Bahwere
Bing Bai
Bo Bai
Ding Bai
Donglin Bai
Gang Bai
Haibo Bai
Jiawei Bai
Julio Bai
Junhong Bai
Lian-Fa Bai
Ming-Yi Bai
Shu-Nong Bai
Songling Bai
Wenlin Bai
Wenlong Bai
Xiaoxiao Bai
Xilian Bai
Xue-Yuan Bai
Yu Bai
Yuchen Bai
Yuehong Bai
Zhi Yi Bai
I. Baiardini
Shakuntala Baichoo
Frank Baiden
David Baier
Mumtaz Baig

Jungeun Baik
Soon Koo Baik
Alexandre Bailao
Neil S. Bailard
Julian Bailes
Alistair Bailey
Charles Bailey
Craig Bailey
Donovan Bailey
Geoff Bailey
Heather Bailey
James Bailey
Kira Bailey
L. Charles Bailey
Robert Bailey
Robyn Bailey
Sean Bailey
Simon Bailey
Timothy Bailey
Travis Bailey
William Bailey
Howard Bailit
Benjamin Bailleul
Aurélien Bailly
Jean-Denis Bailly
Jean-Luc Bailly
Angus Bain
Daniel Bain
Daryl Bainbridge
Michael Baine
Anthony Baines
Christopher Baines
Darrin Baines
Richard Baines
Gianluca Baio
Leonardo Baiocchi
Andrew Baird
Duncan Baird
Emily Baird
J. Baird
Robin Baird
C. Noel Bairey Merz
Alan Bairner
L.K. Bairy
Harsh Bais
Arpita Baisantry
Daniele Baisero
Abhay Bajaj
Avinash Bajaj
Chandrajit Bajaj
Jasmohan Bajaj
Prabin Bajgain
Andrzej Bajguz
Sandra Bajjalieh
Monika Baj-Krzyworzeka
Baubak Bajoghli
Prachi Bajpai
Ali Ahsan Bajwa
Margit Bak Jensen
Murat Bakacak
Anna Bakardjiev
Tamam Bakchoul
Mona Bakeer
Amanj Baker
Andrew Baker
Anthony Baker
Brian Baker
Chris Baker
Christopher Baker
Cindy Baker
Daniel Baker
Elizabeth Baker
Erin Baker
James Baker
Jennifer Baker
Jessica Baker
Joanna Baker
John Baker
Kate Baker
Keith Baker
Kelly Baker
Marissa Baker
Max Baker
Mei Baker
Nevin Baker
Olga Baker
Philip Baker
Richard Baker
Rob Baker
Robert Baker
Simon Baker
Tracie Baker
Marian Bakermans-Kranenburg
Hooman Bakhshi
Saeideh Bakhshi
Pauline Bakibinga
Andrei Bakin
Marit Stordal Bakken
Anthony Bakker
Kevin Bakker
Marije Bakker
Martha Bakker

Matthew Bakker
Jamie Bakkum-Gamez
Omran Bakoush
Giorgos Bakoyannis
Abhijeet Bakre
Shairaz Baksh
Mansi Bakshi
Richard Bakst
Kelly Bakulski
Ercan Bal
Guerkan Bal
Harit Bal
Naresh Bal
P. Matthijs Bal
Jay Bala
Ovidiu Balacescu
Chandrasekar Balachandran
Priyanka Balaganapathy
Jacint Balaguer Coll
Sergio Balaguera-Reina
Sankarathi Balaiya
Uthra Balaji
Vishnu Balaji
Kumudha Balakrishnan
Pachamuthu Balakrishnan
Sharma Rao Balakrishnan
Krishnaswamy Balamurugan
Ivan Balan
Kantesh Balani
Amy Balanoff
George Balanos
Vicent Balanzá
Velmurugan Balaraman
Natasha Balashova
Nafisa Balasinor
Shandiya Balasubramaniam
Mohan Balasubramanian
Poonkuzhali Balasubramanian
Vimalkumar Balasubramanian
Ashok Balasubramanyam
Udeni Balasuriya
Anna Balato
Guillaume Balavoine
Matthew Balazik
Margit Balazs
Péter Balázs
M. Balbaa
Andre Balbi
Bruno Balbi
Alexandre Balbinot
John Balbus
María Elvira Balcells
Fuat Balci
Kelvin Balcombe
Michela Balconi
Cosima Baldari
Chris Baldassano
Damiano Baldassarre
Franco Baldelli
Sara Baldelli
Alfonso Baldi
Franco Baldi
Nicola Baldini
Ronei Baldissera
Vincenzo Baldo
Laura Baldomà
Jason Baldridge
Alessandra Balduini
Olafur Baldursson
Sara Balduzzi
Andrew Baldwin
Cynthia Baldwin
David Baldwin
David R. Baldwin
Matthew Baldwin
Ransom Baldwin
Susan Baldwin
William Baldwin
Michael Bale
Olivier Baledent
Pablo Balenzuela
Panagiotis Balermpas
Michael Bales
Alma Balestrazzi
Barbara Balestrieri
Damien Balestrino
Tanjore Balganesh
Vishal Bali
Christos Baliatsas
Stefano Balietti
Nitin Baliga
Virupax Baligar
Amanda Balish
Lennart Balk
Suzanne Balko
Jennifer Balkus
Frances Balkwill
Becky Ball
Jacob Ball
Jonathan Ball
Lorenzo Ball
Mark Ball

Robyn Ball
Steven Ball
Terry Ball
Anand Ballal
Ford Ballantyne
Gavin Ballantyne
Petek Ballar
Dana Ballard
Elizabeth Ballard
Guy Ballard
Sarah-Blythe Ballard
Cherry Ballard-Croft
Paolo Ballarini
Samir Ballas
Clara Ballerini
Carlos Ballester
Manuel Ballesteros
Soledad Ballesteros
Stefano Ballestri
Matthew Ballew
Max-Bernhard Ballhausen
Umut Balli
Robert Balling
Keith Ballingall
Megan Ballinger
Brian Ballios
Peter E. Ballmer
Mark Ballora
Andrea Ballotta
Jane Balme
Iftekhar Baloch
Peter Balogh
Carlos Balsalobre-Fernández
Joshua Balsters
Angel Baltanas
Valeria Baltar
Evangelos Baltas
Vincent Balter
Andrea Balthasar
Jacques Balthazart
Jay Baltz
Richard Baltz
Agnès Baltzer
Pascal Baltzer
Dimitrios Baltzis
Sudhakar Baluchamy
Rammohan Balusu
Nikola Balvin
Bijorn Balzamino
Jan Balzarini
Francisco Balzarotti
Michael Balzer
Christian Bamberg
Valentina Bambini
Shernaz Bamji
Kiwon Ban
Stephen Ban
Tomohiro Ban
Yusuke Ban
David Banach
Hailey Banack
Niaz Banaei
Ali Mohammad Banaei-Moghaddam
Benjamin Banai
Srinivas Banala
Jesus Banales
Romain Banchereau
Michael Bancks
Germana Bancone
Irina Bancos
Nirmal Banda
Tsitsi Bandason
Abdul Rouf Banday
Carla Bandeira
Maria Elba Bandeira Farias
Masashige Bando
Silvia Bando
Francesca Bandoli
Benjamin Bandowe
Robert Bandsma
Arunava Bandyopadhaya
Anirban Bandyopadhyay
Tirthankar Bandyopadhyay
Anita Bane
Jonas Banefelt
Aditi Banerjee
Anirban Banerjee
Anjan Banerjee
Arindam Banerjee
Arup Banerjee
Daliya Banerjee
Debabrata Banerjee
Debarshi Banerjee
Debasish Banerjee
Kaushik Banerjee
Malay Banerjee
Nilanjan Banerjee
Priyam Banerjee
Rahul Banerjee
Rebecca Banerjee
Sagarika Banerjee
Sandip Banerjee

Sanjay Banerjee
Sanjeev Banerjee
Santanu Banerjee
Sharmistha Banerjee
Soma Banerjee
Sreeparna Banerjee
Sulagna Banerjee
Tanmoyee Banerjee (Chaterjee)
Versha Banerji
Claudia Banescu
Gad Baneth
Mark Banfield
Duhee Bang
Jeong Kyu Bang
Si Ra Bang
Yung-Jue Bang
Paul Banga
S. Banga
Max Bangs
Aria Baniahmad
Pabitra Banik
Lindsay Banin
Michael Banissy
Paulina Bank
Roxanne Banker
Kaitlyn Bankieris
Vassya Bankova
Craig Banks
Jonathan Banks
Lena Morgon Banks
Robert Banks
Sarah Banks
Cristina Banks-Leite
Adriana Bankston
David Bann
James Bann
Jinan Banna
John Bannantine
Danika Bannasch
Wilbert Bannenberg
Norbert Bannert
Edward Banning
Melanie Bannister-Tyrrell
Michael Bannon
Rosa Banos
José Ignacio Baños-Sanz
Ajay Bansal
Anju Bansal
K. Bansal
Manju Bansal
Mukesh Bansal
Parveen Bansal
Sangita Bansal
Shyam Bansal
Rama Bansil
James Banta
Heike Bantel
Hailom Banteyerga
Erin O'Carroll Bantum
Jacob Banuelos
Anne-Laure Bañuls
Cathy Banwell
Robert Banzett
Wolfgang Banzhaf
Rita Banzi
Bin Bao
Bo-Ying Bao
Chang-Jun Bao
Guanhui Bao
Guoqiang Bao
Jie Bao
Jing Bao
Le Bao
Peng Bao
Rui Bao
Wei Bao
Xiaodong Bao
Yan-Ping Bao
Yinguang Bao
Yongbo Bao
Yuhua Bao
Yuqian Bao
Rada M. Baošic
Marine Baptissart
António Baptista
Maria Joao Baptista
Murilo Baptista
Pedro Baptista
R. Baptista
Trino Baptista
Kesha Baptiste-Roberts
Fernando Baquero
Rafael Baquero Parra
Georges Baquet
Moshe Bar
Gyorgy Barabas
Peter Barabas
Alexandra Barabasch
David Baracchi
Vickie Baracos
Behzad Baradaran
Krishna Baradhi

Andrea Baragetti
Ivano Baragetti
Lamia Barakat
Emilia I. Barakova
Himlal Baral
Eugenio Baraldi
Katherine Baran
Yoav Bar-Anan
Jeanette Baran-Gale
Petr Baranov
Anna Baranova
Tom Baranowski
Ulrike Baranyi
Sergio Baranzini
Valentim Barao
Alexander Baras
Edwine Barasa
Jonathan Barasch
Zeinab Barati
Paola Barato
Carmen Barba
A. P. Barba de la Rosa
Ignazio Barbagallo
Mario Barbagallo
Marco Barbanti
Chirullo Barbara
Maurizio Barbara
Ronald Barbaras
Janett Barbaresko
Josephine Barbaro
Luc Barbaro
Maria Barbarossa
Israel Barbash
Mary Barbe
Jean Barbeau
Anita Barbee
Alistair Barber
Fedricker Diane Barber
P.A. Barber
Paul Barber
Joan Barberà
Andrea Barberis
Massimo Barberis
Shannon Barber-Meyer
Andrea Barbero
Morgane Barbet-Massin
Florencia Barbe-Tuana
Egidio Barbi
Maria Barbi
Mariette Barbier
Chiara Barbieri
Edison Barbieri
Fabio Barbieri
Fabio A. Barbieri
Michele Barbieri
Riccardo Barbieri
Maura Barbisin
Maria Barbolina
Andres Barbosa
Angela Barbosa
Dulce Barbosa
F.T. Barbosa
Izabela Barbosa
Juliana Barbosa
Leandro Barbosa
Plínio Barbosa
Diana Barbosa Cunha
Sandra Barbosa-Silva
Alan Barbour
Joshua Barbour
Pascal Barbry
Adrian Barbu
Veronique Barbu
Sukhadeo Barbuddhe
Francesco Barca
Jacqueline Barcelar
Christovam Barcellos
Leonardo Barcellos
Pedro Barcellos-de-Souza
Julie Barcelona
Luciola Barcelos
Carlos Barcenas
Ilaria Barchetta
Lorenzo Barchi
Francesco Barchiesi
Aaron Barchowsky
Aymee Barcia
Rita Barcia
Marcello Barcinski
Pat Barclay
Andrea Barco
Renata Barczynska
Pradip Barde
Janine Barden-O'Fallon
Maria Barderas
Jaydeep Bardhan
Eric Bardinet
Bart Bardoel
Babara Maria Bardoni
Sonia Bardy
Linnea Bärebring
David Barefield

Corinne Bareham
José Barela
Serena Barello
Florence Bareyre
Michael Barfuss
Alexej Barg
Elena Bargagli
John Bargh
Thereza Bargut
Vlasta Bari
Sailen Barik
Andree-Ann Baril
Judit Bar-Ilan
Lucio Barile
Fabio Barili
Sophie Barille
Andrea Barison
Aurelio Bariviera
David Bark Jr.
Antonia Barke
Allen Barker
Bridget Barker
Fiona Barker
Guy Barker
Laurie Barker
Peter Barker
Melissa Barker-Haliski
Andrew Barkley
Fanny Barlaam
Raul Barletta
Tilda Barliya
Jay Barlow
Linda Barlow
Linda Barlow-Mosha
Michael Barmada
Malin Barman
Scott Barman
Galinos Barmparas
Mathieu Barnachon
Agusti Barnadas
Emma Barnard
Shanis Barnard
Lucy Barnard-Brak
Allison Barner
Elizabeth S. Barnert
Andrew Barnes
Anthony Barnes
Betsy Barnes
David Barnes
Deborah Barnes
Hilary Barnes
Mark Barnes
Matthew Barnes
Michele Barnes
Richard S. Barnes
Tim Barnes
Cleveland Barnett
David Barnett
Douglas Barnett
Karen Barnett
Ashton Barnett-Vanes
Anna Barney
Scott Barnhart
D. Barnidge
Cindy Barnig
Scott Barnum
Scott Barolo
Andrew Baron
Christian Baron
Emily Baron
Gino Baron
Thierry Baron
Yotam Bar-On
Francisco Barona-Gómez
Riccardo Baroncelli
Damiano Baroncini
Ines Barone
Rosario Barone
Fuad Baroody
David Bar-Or
Anthony Baross
Katie Barott
Al Baha Barqawi
Elias Barquero-Calvo
Ben Barr
Donald Barr
Martin Barr
Richard Barr
Stephen Barr
W. Andrew Barr
Adriano Barra
Mathias Barra
Laure Barrabé
José Barrabés
Penha Barradas
Antonio Barragan
Jérôme Barral
Margery Barrand
Rodolphe Barrangou
Frédéric Barras
Jonathan Barratt
Nicolas Barraud

Olivier Barraud
Tiago Barreira
Esther Barreiro
Jeffrey Barrell
Martina Barrenschee
Alvaro Barrera
Jonatan Barrera
Roberto Barrera
Benoit Barrès
Massimo Barresi
Cristine Barreto
Fellype Barreto
Savio Barreto
Adam Barrett
Alan Barrett
Angela Barrett
Bruce Barrett
Emily Barrett
James Barrett
Kim Barrett
Maeve Barrett
Perry Barrett
Tara M. Barrett
Tyler Barrett
Douglas Barrick
Antoni Barrientos
Rafael Barrientos
Keith Barrington
Sebastian Barrionuevo
Maite Barrios
Alfred Barritt
Andrew Barron
Anthony Barron
Daniel S. Barron
Christina Barron-Ortiz
Aluisio Barros
Francisco Barros
Nathan Barros
Gérard Barroso
Margarida Barroso
Ubirajara Barroso
Fernando Luís Barroso da Silva
David Barrow
Jeffery Barrow
Nicholas Barrowman
Cameron Barrows
Romina Barrozo
Benjamin Barry
Gillian Barry
John Barry
Orla P. Barry
Peter Barry
Robert Barry
Sarah Barry
Sean Barry
Thomas Barry
Daniel Barshis
Catherine Barsics
Katarzyna Barska
Lindsey Barske
Albert Barskey
Harald Barsnes
Igor Barsukov
Marcin Barszcz
Aldert Bart
Monika Bartekova
Karsten Bartels
Peter H. Bartels
Ronald Bartels
Tim Bartels
Eric Bartelsman
Alexander Bartelt
Renata Bartesaghi
Andreas Barth
Erling Barth
Holger Barth
James Barth
Johannes A. C. Barth
Jürgen Barth
Matthew Barth
Istvan Bartha
Luisa Barthauer
Ramon Barthelemy
Philipp Bartko
Suzanne Bartle-Haring
Diana Bartlett
John Bartlett
Jon Bartlett
Megan Bartlett
Nathan Bartlett
Perry Bartlett
Stephen Bartlett
Julie Bartlett-Trafford
Emily Bartley
Karen Bartley
Colleen Bartman
Thomas Bartnikas
Soraya Bartol
Erzsebet Bartolak-Suki
Francesco Bartoli
Manuela Bartoli
Marc Bartoli

Barbara Bartolini
Jordi Bartolomé
Paolo Bartolomeo
Al Bartolucci
Gianluca Bartolucci
Hazel Barton
James Barton
Jennifer Barton
Michael Barton
Philip Barton
Ludek Bartos
Carla Bartosch
Rafal Bartoszewski
Eva Bartova
Jamie Bartram
Dirk-Uwe Bartsch
Mandy Bartsch
Ronny Bartsch
Sergio Bartual
Guillermo Barturen
Raquel Bartz
Rajat Barua
Paolo Barucca
Vijay Barve
Melanie Barwick
Yaneer Bar-Yam
Jakub Barylski
Daniella Bar-Yosef
Fabiana Bar-Yoseph
Ehsan Basafa
Ajit Basak
Jolly Basak
Saroj Basak
Kanthesh Basalingappa
Randall Basaraba
Ulrike Baschant
Joseph Basconcillo
Laura Baselga-Escudero
John Basgen
Meredith Bashaw
Irfan Bashir
Naheem Bashir
Cecilia Basiglio
Carlo Basile
Franco Basile
John Basile
Stefania Basili
Abdul Basit
Nagarajan Baskaran
David Basketter
Colin Basler
Romain Basmaci
Fulvio Basolo
Lee Bass
Taha Bassal
Vinicius Bassaneze
Marco Bassani
Patricia Bassereau
Emma Bassett
Shalome Bassett
Matteo Bassi
Katia Bassichetto
Nahla Bassil
Seraina Bassin
Ahmed Bassiouni
Mohamed Bassiouni
Dirk Bassler
Steven Bassnett
Bruno Basso
Daniela Basso
Stefano Basso
Michela Bassolino
Marc Basson
Alexander Bassuk
Nick Basta
Francois Bastardie
Amir Bastawrous
Javier Basterra-Gortari
Deepak Bastia
Héloïse Bastiaanse
Jojanneke Bastiaansen
Amy Bastian
Boris Bastian
Frederic Bastian
Andre Bastos
Jairo Kenupp Bastos
Leonardo Bastos
Margarida Bastos
Rogerio Bastos
Veronica Bastos
Simone Bastrup Israelsen
Helene Bastuji
Sylvie Bastuji-Garin
David Bastviken
Ananda Basu
Anirban Basu
Debasish Basu
Maya Basu
Raj Basu
Soumya Basu
Sulagna Basu
Supratim Basu

Robindra Basu Roy
Debashree Basudhar
Vladimir Batagelj
Ken Batai
Veronique Bataille
Carmen Batanero
Sophie Batchelor
Allen Bateman
Emma Bateman
John Bates
Matthew Bates
Michael Bates
Nathaniel Bates
Steven Bates
Kevin Bath
S. Zahra Bathaie
Erik Bathoorn
Mona Batish
Dora Batista
Fernando Batista
Isabel Batista
Luis Batista
Sara Batista
Miguel Batista Junior
Sofia Batista Leite
Diego Batlla
Daniel Batlle
Enric Batllori
Janendra Batra
Nidhi Batra
Elena Batrakova
John Batsis
Will Batson
Ronald Batt
Agatino Battaglia
Donatella Battaglia
Manuela Battaglia
Salvatore Battaglia
Sebastiano Battaglia
Madhusudana Battala
Barbara Battelle
Jonathan Batten
Sonia Batten
C. J. Battey
Joeseph Battistelli
Antonio Battisti
Corrado Battisti
James Battisti
Michele Battisti
Federico Battiston
Pietro Battiston
Stefano Battiston
Michele Battle
Vecihi Batuman
Serafim Batzoglou
Matteo Bauckneht
Anthony Baucum
David Baud
Marc Baud'huin
Veronique Baudin-Creuza
Ezékiel Baudoin
Christophe Baudouin
Luc Baudouin
Alan Baudron
Stéphane Baudry
Adam Bauer
Brent Bauer
Carol Bauer
Christian Bauer
Christoph Bauer
Dana Bauer
Hans Bauer
Isabelle Bauer
Jonathan Bauer
Katherine Bauer
Lorenz Bauer
Natalie Bauer
Paul Bauer
Reinhard Bauer
Robert Bauer
Stefan Bauer
Stuart Bauer
Thomas Bauer
Wolfgang Bauer
S.K. Bauerjee
Fernando Bauermann
Amy Bauernfeind
Stefan Bauersachs
Lee Baugh
Anthony Baughn
Sebastian Bauhoff
Heiko Bauke
Brian Baum
Graham Baum
Linda Baum
Marc Baum
Martina Baum
Michel Baum
Shari Baum
Thomas Baum
Ulrike Baum
Grzegorz Bauman

Margaret Bauman
Paul Bauman
Tyler Bauman
Arnd Baumann
Bernhard Baumann
Frank Baumann
Hannes Baumann
Justin Baumann
Michèle Baumann
Peter Baumann
Ute Baumann
Zofia Baumann
Philipp Baumeister
Roy Baumeister
Eric Baumer
Thomas Baumert
Sebastian Baumgarten
Wolfgang Baumgärtner
David Baumler
Hans Bäumler
Wolfgang Bäumler
Iliana Baums
Hansjörg Baurecht
Cristina Baus
I. Baussano
Victoria Bautch
José Bautista
Luis Bautista
Tara Bautista
Joaquin Bautista Gallego
Matthias Bauwens
Luigi Bavaresco
Sina Bavari
Jasmin Bavarva
Sandeep Bavdekar
Daphne Bavelier
Edith Bavin
Benjamin Bavinton
Vassiliy Bavro
Nicholas Bax
Noortje Bax
Bryan Baxter
David Baxter
Nielson Baxter
Curtis Bay
Trina Bayard
Abolfazi Bayat
Ahmet Baydin
Ahmet Baydur
Anity Bayer
Antony Bayer
Maddalena Bayer
Monika Bayer
Till Bayer
Laurie Bayet
Satu Baylan
Kenneth Bayles
Richard Bayles
Christine Baylis
Christopher Bayliss
Douglas Bayliss
Christopher Bayne
Javier Bayod
Ozgur Bayram
Süleyman Bayram
Paul Bays
Christos Bazakos
Haydee Bazan
Fuller Bazer
Alexandr Bazhin
Lauren Bazinet
Róza Bazinska
Joel Bazira
Babak Bazrgari
Ivonne Bazwinsky-Wutschke
Riccardo Bazzardi
Jose Antonio Bea
James Beach
Terry Beacham
Joanna Beachy
Carol Beadling
Ian Beales
Christopher Beam
Jennifer Beane-Eben
Rachel Louise Beanland
Daniel Beard
Philippa Beard
John Beardall
Luke Beardon
Richard Beare
Cynthia Bearer
Daniel Bearup
Juan De Dios Beas-Jiménez
David Beasley
James Beasley
Lori Beason-Held
Ken Beath
Marco Beato
Michael Beattie
Tara Beattie
Brian Beatty
J. David Beatty

Mark Beatty
Tsevi Beatus
Roger Beaty
Terri Beaty
Raphaëlle Beau Lejdstrom
Nicole Beauchemin
Amélie Beaudet
Kathy Beaudette
Frederic Beaudry
Laurent Beaulaton
Michael Beaulieu
Christophe Beauloye
Coreen Beaumier
Kimberley Beaumont
Marc Beaumont
Thomas Beaver
Daniel Beavers
Amber Beavis
Karen Beazley
Cécile Bébéar
Lisa Bebell
James Bebko
Francisco Beca
Egle Beccalli
Tommaso Beccari
Alejandro Beceiro
Mercedes Becerra
Cristina Becerra-Castro
Baltazar Becerril
Sara Becerril
Bodil Bech
Martin Bech
Per Bech
Elias Bechara
Marc Bechard
Meagan Bechel
Ronan Becheler
Oren Becher
Peter Moritz Becher
Lars Bechmann
Jesper S. Bechsgaard
Alain Beck
Alan Beck
Christine Beck
Daniel Beck
Eleanor Beck
Hans-Peter Beck
Josh Beck
Jürgen Beck
Laurence Beck
Melinda Beck
Sarah Beck
Nicole Beckage
Abbey Becker
Aline Becker
Annette Becker
Birgit Becker
Catherina Becker
Christian Becker
Christoph Becker
Daniel Becker
Ingeborg Becker
James Becker
Janet Becker
Julia Becker
Karsten Becker
Mark Becker
Matt Becker
Pamela Becker
Silke Becker
Ulrik Becker
Walter Becker
Gabriel Beckers
Lane Beckes
Christine Beckett
Carla Beckham
Yvonne Beckham
Barbara Beckman
Manfred Beckmann
Nicholas Beckmann
Nicolau Beckmann
Consuelo Beck-Sague
Oliver Beckstein
Antonio Bedalov
Donna Bedard
Emilie Bedard
Simon Beddows
Peter Bede
Tibor Bedekovics
Harold Bedell
Branka Bedenic
Joshua Bederson
Helen Bedford
Michael Bedford
Mourad Bédir
Barbara Bednarczyk-Cwynar
Piotr Bednarek
Petr Bednarik
Robert Bednarik
Christine Bedore
Pierrick Bedouch
Claudia Bedoya

Renesh Bedre
David Bedwell
David Beebe
Kristin Beebe
Stephen Beebe
Kristian Beedholm
Aaron Beedle
Alicia Beeghly-Fadiel
Terry Beehr
Aartjan Beekman
Karen Beemon
Ella Been
Laura Been
Jan-Willem M. Beenakker
David Beer
Karlyn Beer
Philip Beer
Ronny Beer
Stephen Beer
Michal Schnaider Beeri
Peter Beernink
Paul Beeson
Michael Beeton
Bonnie Beezhold
Amer Beg
Danny Bega
Grace Begany
Gerrit Begemann
Maria Beger
Denovan Begg
Douglas Begg
Ettore Beghi
Laurent Beghin
David Begley
Thomas Begley
Chiara Begliomini
Maria Begnami
Maria T. Begonia
Mélanie Béguer-Pon
Julien Beguin
Alexander Begun
Joachim Behar
Jaideep Behari
Homira Behbahani
Fariba Behbod
Lambodar Behera
Tapan Behl
Tarannum Behlim
Brian Behm
Jinan Behnan
Carl-Ludwig Behnes
Martin Behnisch
Sascha Behnk
J. Behnsen
Björn Behr
Juergen Behr
Marcel Behr
Rudiger Behr
Volker Behrends
Maria Isabel Behrens
Martin Behrens
Michael Behring
Jason Behrstock
Jin-Xin Bei
Roberto Bei
Deborah Beidel
Frank Beier
John Beier
Paul Beier
Elizabeth Beierle
Frank Beierlein
Lars Beierlein
W. H. Beierwaltes
John Beigel
Catherine Beigelman-Aubry
Anne-Sophie Beignon
Omid Beiki
Tatsiana Beiko
Traude Beilharz
Gregory Beilman
Mariano Beiró
Kostantinos Beis
Timothy Beischlag
Tim Beißbarth
Christoph Beisswenger
Greg Beitel
Jeremy R. Beitler
Donald Beitz
Oded Beja
Sonia Bejarano
Kimon Bekelis
Isabelle Bekeredjian-Ding
Kenes Beketayev
Jolita Bekhof
Valeria Bekhtereva
Andrey Bekker
Siroon Bekkering
Mark Bekoff
Cavin Epie Bekolo
Lynn Bekris
Kemal Beksac
Yusuf Bektas

Tefera Belachew
Rachida Belaich
Angjelina Belaj
Vikas Belamkar
Richard Belanger
Sabine Bélard
Alexandra Belayew
Lazaros Belbasis
Britany Belcher
William Belcher
Ricardo Beldade
Pedro Belda-Ferre
Christien Belden
Guido Beldi
Laurent Belec
Angel Belenguer
Enrique Belenguer
Nadya Belenky
Peter Belenky
Manfred Beleut
Anna Belfer-Cohen
Antonino Belfiore
Georges Belfort
Marlene Belfort
Benjamin Belgrad
Danielle Belgrave
Eleni Beli
Nadejda Beliakova-Bethell
Lisa Belin
Steven Belinsky
John Belisle
Boris Belitsky
Murat Belivermis
Leila Belkhir
Adrian Bell
Andrew Bell
Brittany Bell
Charles Bell
Christina Bell
D. Bell
David Bell
Graeme Bell
Ian Bell
Jason Bell
Johann Bell
Margaret Bell
Matthew Bell
Michael Bell
Raoul Bell
Rebecca Bell
Scott Bell
Trevor Bell
Jonathan Bella
Giacomo Bellani
C. Bellard
Antonio Bellasi
Chandra Bellasio
Ashwin Belle
Janeil M Belle
Clémence Belleannée
Barbara Bellei
Andrea Bellelli
Giuseppe Bellelli
Andrew Bellemer
Claudio Bellevicine
Silvia Bellezza
Luca Belli
Jérémy Bellien
Jean-Pierre Bellier
Dwight Bellinger
Michelle Bellingham
Peter Bellingham
Marco Bellinzoni
Gael Belliot
Bruno Bellisario
Romuald Bellmann
Folasade Bello
Gonzalo Bello
Nicholas Bello
Rafael Bello
Martiniano Bello Ramirez
Luigi Bellocchio
Amparo Belloch
Luis E. Bello-Espinosa
Teresa Bellon
Elena Belloni
Beatriz Bellosillo
Stefano Bellosta
Juan Bellot
Pau Bellot
Deborah Bell-Pedersen
D. U. Bellstedt
Elisa Bellucci
Ilaria Belluomo
David Bellwood
John Belmont
Matthew Belmonte
Jayne Belnap
Jean-Claude Beloeil
Pavel Belolipetsky
Ron Beloosesky
Artem Belopolsky

Alexandre Belot
Travis Belote
Apostolos Beloukas
George Belov
Alexia Belperron
Jessica Belser
Denise Belsham
Graham Belsham
Daniel Belstrøm
Suzanne Belton
Jerzy Beltowski
John Beltrame
Elena Beltramo
Jose Beltran-Abaunza
Rhonda BeLue
Maria Belvisi
Olga Belyaeva
Michel Belyk
Clara Belzer
Daryl Bem
Debra Bemben
Michael Bemben
Bruno Bembi
Alexis-Pierre Bemelmans
Lynne Bemis
Moufida Ben Nasr
Izhar Ben- Shlomo
Bruria Ben Zeev
Alexandra Benachi
R. Benamouzig
Philippe Benaroch
April Benasich
Barbara Benassi
Alexandra Benavente-Perez
Jorge Benavides
Juan Benavides
Julio Benavides
Maria Benavides
Sandra Benavides
Andres Benavides-Serralde
Clover Bench
Goran Bencic
Eran Bendavid
Reina Bendayan
Jennifer Bender
Kevin Bender
Ralf Bender
Tamás Bender
Natalya Benderska
Lusiane Bendhack
Ivo Bendix
Casper Bendixsen
Michele Bendoni
Caterina Bendotti
Iddo Ben-Dov
Jason M. Beneciuk
George Beneck
György Benedek
Mathias Benedek
Andrea Benedetti
Celso Benedetti
Maura Benedetti
Sara Benedetti
Lauryn Benedict
Mark Benedict
William Benedict
Stefano Benedini
Giovanni Benelli
Itzhak Benenson
Ayse Bener
Bedrich Benes
Kylla Benes
Andrew Benest
Emmanouil Benetos
Fernando Benetti
Thomas Benfield
Eyal Bengal
Susanne Bengesser
Michael Bengfort
Enrique García Bengoechea
Xabier Bengoetxea
Andrew Bengsen
Sarah Bengston
David Bengtson
Stefan Bengtson
Boel Bengtsson
Ewert Bengtsson
Kristina Bengtsson
Moussa Benhamed
Simone Benhamou
Shomron Ben-Horin
Tal Ben-Horin
Tamir Ben-Hur
Elia Beniash
Elisa Benincà
Stefano Benini
Jean-Pierre Benitah
Bruno Benitez
Gloria Benítez-King
Alfonso Benítez-Páez
Javier Benítez-Porres
Beatriz Benitez-Temino

Agustín Benito
Elia Benito
Jon Benito
Santiago Benito
Marta Benito Garzon
Laura Benjamin
Longo-Mbenza Benjamin
Tara Benjamin
William Benjamin
Dan Benjamini
Joyce Benjamins
Kálmán Benke
Noureddine Benkeblia
Moncef Benkhalifa
Szilvia Benko
Cassandra Benkwitt
Kamel Benlagha
Peter Benn
Mar Bennasar
Ina Benner
Alan Bennett
Andy Bennett
David Bennett
Derrick Bennett
Elizabeth Bennett
Eric Bennett
Gary Bennett
Gordon Bennett
Jonathan Bennett
Marc Bennett
Matthew Bennett
Pauleen Charmayne Bennett
Phillip Bennett
Robert Bennett
Steffany Bennett
Tellen Bennett
Vann Bennett
William Bennett Jr.
Rachel Bennetts
Richard Benninger
Abby Benninghoff
Hugues Benoit
Joshua Benoit
Kelly Benoit-Bird
Eric Benotsch
Touati Benoukraf
Neal Benowitz
Hilla Ben-Pazi
Anna Benrick
Sabine Bensamoun
Olivier Bensaude
Bernadette Bensaude-Vincent
Kimberley Benschop
Mohammed Bensellam
Rotem Ben-Shachar
Miles Bensky
Al Benson
Austin Benson
Deanna Benson
Jacquelyn Benson
John Benson
Lauren Benson
Roger Benson
Stacey Benson
Thomas Benson
Valerie Benson
Judith Benstein
Andrew Bent
Smadar Ben-Tabou de-Leon
M. Bentayeb
Marina Bentivoglio
Bastian Bentlage
Margaret Bentley
Rebecca Bentley
Isabel Bento
David Bentrem
Jacob Bentzon
Gian Maria Niccolo' Benucci
Francesca Benuzzi
Amine Benyamina
Yael Benyamini
Daniel Benyshek
Dominik Benz
Roland Benz
Adele Benzaken
Sébastien Benzekry
Karim Benzerara
Michaela Benzeval
Karen Benzies
Roberto Benzo
Idrissa Beogo
Athanasios Beopoulos
Samir Bera
Subir Bera
Roy Beran
Alfredo Berardelli
Rossana Berardi
Friederike Berberich-Siebelt
Marika Berchicci
Herve (Hillel) Bercovier
Hillel Bercovier
Fred Bercovitch

Jo Berden
Aikaterini Berdiaki
Evgeny Berdyshev
Yoella Bereby-Meyer
Ludek Berec
Evelien Berends
Juan Berenguer
Laura Beretta
Lorenzo Beretta
Alexander Berezin
Maxim Berezovski
Arthur Berg
Bente Berg
Christine Berg
Karl Berg
Lisa Berg
Marc Berg
Maureen Berg
Ole Berg
Helena Bergallo
Marco Bergamin
Martina Bergant
Gaby Berg-Beckhoff
Lena Bergdahl
Eivind Berge
Lillian Berge
Stefaan Berge
Daniel Bergé
Sara Bergek
Corinna Bergelt
Mia Bergenmar
Adam Berger
Andrew Berger
Angelika Berger
Bryan Berger
Edward Berger
Elizabeth Berger
Franklin Berger
Helmut Berger
Johannes Berger
Joseph Berger
Leopold Berger
Miles Berger
Walter Berger
Eric Bergeron
Marc Bergeron
Oded Berger-Tal
Laurent Berges
Christina Bergey
Elizabeth Bergey
Thomas Berghaus
Ina Bergheim
Vincenzo Berghella
Teresa Bergholz
Herman Berghuijs
Stephen Bergin
Steven Bergink
Veerle Bergink
Helmut Bergler
Douglas Bergman
Mindy Bergman
Peter Bergman
Christina Bergmann
Dominique Bergmann
Martin Bergmann
Olaf Bergmann
Tjard Bergmann
Ulrich Bergmann
Elke Bergmann-Leitner
Lesley Bergmeier
Sebastian Bergrath
Niels Bergsland
Eva Bergstraesser
Sara M. Bergstresser
Christel Bergstrom
Goran Bergstrom
Ann-Kristin Bergström
Jason Bergtold
Anna Berim
Ioana Berindan-Neagoe
Antony Beris
Alexander Beristain
S. Berke
Sara Berkelhamer
Murat Sami Berkman
Kathleen Berkner
Bruce Berkowitz
Oliver Berkowitz
Richard Berl
Joshua Berlin
Léo Berline
Lawrence Berliner
Daniel Berlowitz
Dave Berman
Gordon Berman
Judith Berman
Nancy Berman
Robert Berman
Yonatan Berman
Jesus F. Bermejo-Martin
Emma Bermingham
Luiz Bermudez

Maria de la Luz Bermudez
Roberto Bernabei
Antonio Bernabe-Ortiz
Nicola Bernabò
Javier Bernácer
Juan Bernal
Julio Bernal
Moises Bernal
Vicente Bernal
Ernesto Bernal-Mizrachi
Daniel Bernard
David Bernard
Genevieve Bernard
Jessica Bernard
Karen Bernard
Kathryn Bernard
Laurence Bernard
Lisa Bernard
Nicole Bernard
Francesco Bernardi
Jamila Bernardi
Luciano Bernardi
Marco Bernardi
Paolo Bernardi
Stella Bernardi
Giovanni Bernardini
Léonard Bernard-Jannin
Lawrence Patrick Bernardo
Mark Bernards
Rene Bernards
Massimo Bernaschi
Françoise Bernaudin
Simon Bernèche
Espen Berner
Silke Bernert
Sue Berney
Jürgen Bernhagen
Anne Bernhard
Bernhard Bernhard
Frank Bernhard
Susanne Bernhardsson
Christopher Bernhardt
M. Brooke Bernhardt
Stephan Bernhart
Roberto Berni Canani
Steve Bernier
Max Berniker
Franco Bernini
Roberta Bernini
Stine Bernitz
David Bernlohr
Raymond Bernor
Daniel Bernoulli
Gregory Berns
Kenneth Berns
Amit Bernstein
Audrey Bernstein
Daniel Bernstein
David Bernstein
Elana Bernstein
Hans-Gert Bernstein
Jon Bernstein
Matthias Bernt
Jens Magnus Bernth-Jensen
Jordi Bernues
Audrey Bernut
Lisa Bero
Luciana Berod
Mary Beroya-Eitner
Claudine Berr
Patrick Berrebi
Jean-François Berret
Sonia Berrih-Aknin
Alan Berry
Colin Berry
Cristine Berry
Don Berry
Donagh Berry
Fred Berry
Kristin Berry
Paul Berry
William Berry
Patricia Bersanetti
Ferdinando Bersani
Graziella Berta
Maurizio Bertaina
Francesca Bertani
Cinzia Bertea
Kimberly Bertens
Thomas Bertero
Laureline Berthelot
Jessica Berthiaume
Franz Berthiller
Hans-Rudolf Berthoud
Francois Berthoux
Lionel Berthoux
Stefan Berti
Lien Bertier
Hanna Bertilsdotter Rosqvist
Stefan Bertilsson
Caterina Bertini
Elisa Bertino

Stefano Berto
Monica Bertoia
Mita Bertoldi
A. Bertoletti
Lucio Bertoli Barsotti
Giovanni Bertolini
Maria Célia Bertolini
Martinna Bertolini
Maurizio Bertollo
Antonio Bertolotto
Paulo Bertolucci
Matthew Bertone
Francesco Bertoni
Kelly Bertram
Stephanie Bertram
Alexander Bertrams
Jane Bertrand
Frédéric Bertucci
Rômulo Bertuzzi
Daniel Berwick
Olga Berwid
Nora Besansky
Kurt Beschorner
Julie Besco
Bruno Besen
Elyssa Besen
Kerim Beseoglu
Shadi Beshai
John Beshears
Khalid Beshir
Thor Besier
Huseiyin Besiroglu
Fabrice Besnard
Guillaume Besnard
Stephane Besnard
Lucinda Bessa
Carmen Bessa-Gomes
Benjamin Besse
Florence Besse
Pascale Besse
Paul Bessell
David Besselsen
James Bessen
Jean-Louis Bessereau
Alessandro Bessi
Edward Bessman
Pascal Bessong
Emma Best
Maisy Best
Stephen Best
Sébastien Besteiro
Dirk Bester
Kevin Bestgen
Andrew Beswick
Carsten Beta
Laura Betancor
Andrea Betancourt
Walter Betancourt
Michael J Betenbaugh
John Bethard
Paul Bethke
Ingrid Bethus
V. Betihavas
Ana Pilar Betrán
Cornelia Betsch
Kirsten Bett
Philip Bett
Ahmed Bettaieb
Michael Betteken
Ana Bettencourt
Lucien Bettendorff
Pete Bettinger
Dean Betts
James Betts
Saverio Bettuzzi
Christian Betzel
Richard Betzel
Penny Beuning
Tushar Kant Beuria
Manfred Beutel
Rolf Georg Beutel
Bruce Beutler
Carmen Beuzón
Charlotte Bevan
Chris Bevan
David Bevan
David Beversdorf
Danilo Bevk
Jeffrey Bewley
Peter Bex
Rudi Beyaert
Haluk Beyenal
Andreas Beyer
Eric Beyer
Wolfgang Beyer
Andreas Beyerlein
Sinem Beyhan
Amanuel Beyin
Rob Beynon
E. Beyreuther
Daniel Beysens
Nurit Beyth

Yasin Bez
Angelika Bezan
Katiuska Bezares
Ram Bezawada
Daniela Bezemer
Jorge Bezerra
Marcelo Bezerra
Rosângela Maria Neves Bezerra
Vivien Beziat
Annie Bézier
Dmitri Bezinover
Eugenia Bezirtzoglou
Ilya Bezprozvanny
Tunira Bhadauria
Punyasloke Bhadury
Sanjay Bhagani
Balasubramanian Bhagavath
Nikhil Bhagwat
V. R. Bhagwat
Mehul Bhakta
Rishi Bhalerao
Ramji Bhandari
Tulsi Bhandari
Purnima Bhanot
Syon Bhanot
Mausumi Bharadwaj
Shrikant Bharadwaj
Amrita Bharat
Anil Bharath
D. Gnana Bharathi
Rukmini Bhardwaj
Aditi Bhargava
Kalpana Bhargava
Maneesh Bhargava
B. Bharti
Kapil Bharti
Deeksha Bhartiya
L. V. K. S. Bhaskar
Sangeeta Bhaskar
Srividya Bhaskara
Ajaz Bhat
G. Jayarama Bhat
Ramesh Bhat
Zeenat Bhat
Vikrant Bhateja
Anuj Bhatia
Sabhyata Bhatia
Raj Bhatnagar
Rakesh Bhatnagar
Sandhya Bhatnagar
Shinjini Bhatnagar
Sonika Bhatnagar
Pooja Bhatnagar-Mathur
Pavan Bhatraju
Aditi Bhatt
Deepak L. Bhatt
Jay Bhatt
Veer Bhatt
Dharma Bhatta
Gopal Bhatta
Laxmi Bhatta
Arnab Bhattacharjee
Ashish Bhattacharjee
Niloy Bhattacharjee
Saikat Bhattacharjee
Subhadeep Bhattacharjee
Surajit Bhattacharjee
Akash Bhattacharya
Haimanti Bhattacharya
Jayanta Bhattacharya
Kunal Bhattacharya
Parna Bhattacharya
Sabyasachi Bhattacharya
Saswata Bhattacharya
Sharmila Bhattacharya
Sourav Bhattacharya
Sudha Bhattacharya
Indraneel Bhattacharyya
Maitree Bhattacharyya
Mrinal Bhattacharyya
P. Bhattacharyya
R. Bhattacharyya
Sanjib Bhattacharyya
Swati Bhattacharyya
Tapan Bhattacharyya
Nirjal Bhattarai
Ashay Bhatwadekar
Dheeraj Bhavanasi
Mrinal Bhave
Satyendra Bhavsar
Rajendra Bhimma
R. Bhise
Vikrant Bhosle
Alauddin Bhuiyan
Muhammed Bhuiyan
Zaver Bhujwalla
Rajinder Bhullar
Arun Bhunia
Lokesh Bhushan
Hongtao Bi
Peng Bi
Rui Bi

Shengli Bi
Shundong Bi
Shuoben Bi
Tinai Bi
Weimin Bi
Xin Bi
Ye Bi
Fantahun Biadglegne
Elena Biagi
Giacomo Biagi
Michal Bialek
Michal Bialy
Chunjing Bian
Hejiao Bian
Hui-Jie Bian
Jiang Bian
Jing Bian
Liming Bian
Xiaofang Bian
Yiwen Bian
Zhaoxiang Bian
Carlo Bianca
Tommaso Biancalani
Sylvain Biancamaria
Emily Bianchi
Francesca Antonella Bianchi
Giacomo Bianchi
Giulia Bianchi
Matt Bianchi
Matteo Bianchi
Antonio Bianco
Bianca Bianco
Pasquale Bianco
Piero Bianco
Valentina Bianco
Vittorio Bianco
Luigi Biancone
Alexandre Biasi
Emiliano Biasini
Fred Biasini
Roberto Biassoni
Nadia Biassou
Daniele G. Biasucci
Luigi Biasucci
Kyle Bibby
Knut Biber
Peter Biberthaler
Haim Bibi
Frederic Bibollet-Ruche
Rodrigo Bicalho
Thomas Bice
Daniel Bichet
Manuel Bicho
Derek M Bickhart
Penny Bickle
Myriam Bickle Graz
Stephen Bickler
Timothy Bickmore
Robert Biczak
Gavin Bidelman
Dennis Bideshi
Aurelie Bidet Caulet
Christel Bidet-Ildei
Felicitas Bidlack
Aurelian Bidulescu
Cinnamon Bidwell
Gene Bidwell
Joseph Bidwell
Peter Bie
Yiming Bie
Erhard Bieberich
Lori A. Biederman
Peter Biedermann
Madeleine Bieg
Marieke Biegstraaten
Michael Biehl
Lawrence Bielak
Maximilian Bielohuby
Ewa Bielska
Godfrey Biemba
Frank Bienaimé
Kaspar Bienefeld
John Bienenstock
Lois Biener
Paul Bienfang
Michal Bienkowski
Rachelle Bienstock
Mariann Bienz
Georg Bier
Raven Bier
Gabriele Bierbaum
Barbara Bierer
Julie Bierer
Tor Biering-Sørensen
Marc Bierkens
Wouter Bierman
Lauren Biermann
Nienke Biermasz
Beata Biernat
Bernhard Biersack
Hans Konrad Biesalski
Jan Bieschke

Leslie Biesecker
Brandon Biesiadecki
Erik Biessen
Franck Biet
James Bigelow
Kimberly Bigelow
Patrick Biggar
Mary Biggs
Gregory Biging
Erin Bigler
Jean Joel Bigna
Elena Bignami
Elaine Bignell
Darell Bigner
Christophe Bignon
Eliana Bignotti
Lucie Bigoni
Sarah Bigot
V. Bihari
Rimke Bijker
Janetta Bijl
Philippe Bijlenga
Erik Bijleveld
Maarten Bijlsma
Merijn Bijlsma
Jelle Bijma
Roxali Bijmoer
Bart Bijnens
Polly Bijur
Elisabeth Bik
Boris Bikbov
Maryam Bikhof
Daniel Bikle
András Bikov
Margaret Bikowski
Marom Bikson
Jose Ramon Bilbao
Joke Bilcke
Trine Bilde
Melissa Bilec
Martin Bilej
Bahar Bilgen
Damla Bilgin
Rasit Bilgin
Kerstin Bilgmann
Shivaprasad Bilichodmath
Andras Bilkei-Gorzo
Donna M. Bilkovic
Elisabeth Billard
Patrick Billard
Marie Billaud
Pablo Billeke
Johan Billen
Barbara Biller
Rolf Billeskov
Adrian Billeter
Sarah Billeter
Andrea Billi
Fabrizio Billi
Philippe Billiald
Annelies Billiet
Martin Billinger
Jenny Billings
Peter Billingsley
Bridgette Jeanne Billioux
Maxime Billot
W. Billups
Simon Bilodeau-Gauthier
Federico Bilotta
Osman Bilsel
Mark Bilsky
Stefan Bilz
Luis Bimbo
Liao Bin
Agnes Binagwaho
Claudia Bincoletto
Maria Binda
Paola Binda
Laure Bindels
Markus Bindemann
Andreas Binder
April Binder
Marco Binder
Mascha Binder
Vera Binder
Richard Bindler
Laurence Bindschedler
Lorena Binfa
Geoffrey Bingham
Colin Bingle
Tobias Bingold
Estela Bini
Aristea Binia
Michael Binks
Rachel Binks
James Binley
Martyn Binnie
Sandra Binning
Colin Binns
Helen Binns
Gordon Binsted
Amrei Binzer

Maria Binz-Scharf
Antonio Biondi
Marco Biondi
Giuseppe Biondi-Zoccai
Silvia Bione
Eileen Birch
Karen Birch
Rachael Birch
Adam Bird
Christopher Bird
Justin Bird
Sheila Bird
Tomas Bird
Tannaz Birdi
Kelly Birdwell
Ruthie Birger
Göran Birgersson
Sofia Birgersson
Zewdie Birhanu
Nana-Kwadwo Biritwum
Marco Birke
Sarah Birken
Gerd Birkenmeier
Matthew Birket
Michael Birkett
Wolfgang Birkfellner
Peter Birkholz
Lauren Birks
Serge Birman
Andrea Birnbaum
Ruth Birner-Gruenberger
Maté Biro
Simone Birocchi
Tal Biron-Shental
L. Birrell
Andrew Birtles
Ingvars Birznieks
Marco Bisaglia
Claudio Biscaro
Danielle Biscaro Pedrolli
John Bischof
Markus Bischoff
Serge Bischoff
Michelangelo Bisconti
S. Bisdas
Joseph Bisesi Jr.
David Bishai
Anthony Bishara
Mahendra Bishnoi
Kumar Sanjeev Bishnupuri
Christopher Bishop
David Bishop
Jacqueline Bishop
John Bishop
Laura Bishop
Melanie Bishop
Nicholas Bishop
Somer Bishop
Tom Bishop
Naveen Bisht
Tiziana Bisogno
Katerina Bišová
Marcelo Bispo de Jesus
Bernie Bissett
Nazaré Bissoli
Leslie Bisson
Marie-Josee Bisson
Annie Bissonnette
Robert Bissonnette
Akshaya Biswal
Basanti Biswal
Manas Biswal
Anup Biswas
Arijit Biswas
Ashis Biswas
Dipayan Biswas
Raja Biswas
Saswati Biswas
Shekhar Biswas
Subhra Biswas
Sumalika Biswas
Swati Biswas
Tanuka Biswas
Flávio Bitencourt
Davide Bitetto
Michael Bithell
Gulfidan Bitirgen
Ari Bitnun
Jacob Bitoun
Alan Bittles
George Bittlingmayer
Eric Bittman
Stefan Bittner
Martin Biuw
Roger Bivand
Marie Bixo
Mozhgan Bizhang
Kostadinka Bizheva
Anna Bizzarri
Matthew Bizzarro
Anders Bjartell
Reidun Bjelland

Thomas Bjerner
Morten Bjerregaard-Andersen
Peter Bjerring
James Bjork
Mats Bjork
Ingemar Björkhem
Heidi Björklund
Geir Bjørklund
Anders Björkman
Isabella Bjorkman-Burtscher
Maria Björkqvist
Dale Bjorling
Anders Bjørn
Bodil Bjorndal
Lars Bjørndal
Sigridur Bjornsdottir
Ingunn Björnsdottir
Einar Björnsson
Ghassan Bkaily
Ellen Blaak
Yoann Blache
Alyssa Blachez
Alex Black
Christopher Black
Dennis Black
Evan Black
Frank Black
James Black
Katherine Black
Kirsten Black
Lindsay Black
Mairead Black
Paul Black
Sandra Black
Stephen Black
Steve Black
Daniel Blackburn
Jason Blackburn
Todd Blackledge
Stephen Blacklow
Roger Blackman
Richard Blackmon
Murray Blackmore
Neil Blackstone
Helen Blackwell
Julie Blackwood
Nigel Blackwood
Sarah Blagden
Trenna Blagden
Evgenia Blagodatskaya
Gergin Blagoev
Rok Blagus
Victoria Blaho
Stefanie Blain-Moraes
Cindy Blair
Clancy Blair
David Blair
Jaime Blair
Paul Blair
Rachael Blair
Guillaume Blaire
Chris Blais
Damer Blake
John Blake
Miranda Blake
Neil Blake
Eleanor Blakely
Tony Blakely
Paul Blaker
April Blakeslee
Brian Blakley
J. Edwin Blalock
James Edwin Blalock
Sean Blamires
Dominique Blanc
Lionel Blanc
Mathieu Blanc
Elison Blancaflor
Danielle Blanch Hartigan
Alain Blanchard
Anita Blanchard
Frederic Blanchard
Pierre-Alexandre Blanche
Christopher Blanchette
Amalio Blanco
Antonio Blanco
Carlos Blanco
Fernando Blanco
José Ramón Blanco
Leocadio Blanco-Bercial
Carlos Blanco-Centurion
Francisco Blanco-Vaca
Helen Bland
Jeffrey Bland
Martin Bland
Fabio Blandini
Giovanna Blandino
Fabian Blank
Hartmut Blank
Melissa Blank
Ralf Blank
Robert Blank

James Blankenship
Jill Blankenship
Kevin Blankenship
Terry Blankenship-Paris
Marco Blanker
Peter Blankestijn
Paul Blankman
Andrew Blanks
Ron Blankstein
Jesse Blanton
Lucas Blanton
Susan Blanton
Ronald Blasberg
Julia Blasch
Wolfgang Blaschek
Hélène Blasco
Ruth Blasco
Vincent Blasco-Baque
Isabel Blasco-Costa
Hilario Blasco-Fontecilla
Robert Blasdel
Christopher Blase
Martin Blaser
Rachel Blaser
Francesco Blasi
Juan Blasi
Udo Bläsi
Janusz Blasiak
Gregory Blass
Ivan Blasutig
Janusz W. Blaszczyk
Alex Blaszczynski
Richard Blatchford
Cedric Blatter
Joseph Blattman
Christine Blattner
Nenad Blau
Tamilane Blaudeau
Bärbel Blaum
Burns Blaxall
Sergio Blay
Salvador Blaya
Jeremy Blaydes
Douglas Blayney
Jaine Blayney
Jane Blazeby
Stephan Blazek
Anthony Blazevich
Ma Blazquez
Charlotte Blease
J. Bledsoe
Margit Bleecker
David Bleich
Kerrin Bleicher
Nienke Bleijenberg
Konstantinos Blekas
Kenneth Blemings
Ian Blenkharn
Timothy Blenkinsop
Josefa Bleu
Sophie Bleves
Jon Blevins
Lynn Blewett
Marnie Blewitt
Claudine Bleykasten
Ronald Bleys
Lauren Blieden
David Blinder
James Bliska
Joseph Bliss
Jackie Blissett
Agnieszka Blitek
Bradley Blitvich
Dana Bliuc
Francis L. Bliven
Nikolay Bliznyuk
Carl Blobel
Carter Bloch
Jean-Francis Bloch
Eric Block
Geoffrey Block
Hannah Block
Marc Block
Matthew Block
Timothy Block
Ariel Blocker
Nichlolas Blockley
Oswald Bloemen
Marcus Bloice
Domenico Daniele Bloisi
Daan Blok
Ashley W. Blom
Henning Blom
Jeanet Blom
Erik Blomberg
Jonas Blomberg
Christine Blome
Sandra Blome
Gary Blomquist
Johanna Blomqvist
Lennart Blomqvist
Eva Blomstrand

Nicolas Blondeau
Jacques Blondel
Katherine Blondon
Linda Bloom
Marshall Bloom
Kim Bloomfield
Susan Bloomfield
Jeffrey Bloomquist
Frank Bloos
John Blosnich
Mark Blostein
Stijn Blot
Clemence Blouet
Martin Blouin
Zachary Blount
Michael Blower
Carmen Blubaugh
David Bluemke
Burton Bluhm
Claudine Blum
Marc-Michael Blum
Yuna Blum
Bruce Blumberg
Scott Blume
Margaret Blume-Kohout
Orit Blumenfeld
Yair Blumenfeld
Marcel Blumensatt
James Blumenthal
Robert Blumenthal
Ulrike Blume-Peytavi
Tom Blundell
Nicholas Blurton-Jones
Hans Bluyssen
Benjamin Blyth
Hazel Blythe
Jennifer Blythe
Michaela Blyton
Jin Bo
Marzia Bo
Sune Bo
Leigh Boardman
Godfred Boateng
Alessio Boattini
Paula Boaventura
Muhammad Bobat
Gerd Bobe
Rene Bobe
Vladimir Bobek
Maria Bobes
William Bobier
Alex Bobik
Johannes Bobjer
Marina Bobkova
Simina Boca
Anamelia Bocca
Stefano Boccaletti
Enrique Boccardo
Thomas Bochynek
M. Bocian
Mateusz Bocian
Jan Bocianowski
Beth Bock
Christian Bock
Christina Bock
Ronald Böck
Detlef Bockenhauer
Christopher Bockisch
Ildiko Bock-Marquette
Fadima Bocoum
Christian Bocti
Enrica Boda
Olaf Bodamer
Dhananjay Bodas
Manish Bodas
Christopher Boddy
Jürgen Bode
Clara Bodelon
Scott Boden
Natacha Bodenhausen
Olivier Bodenreider
Rogier Bodewes
Jason Bodily
Antonio Bodini
Benedetta Bodini
Ivan Bodis-Wollner
Richard Bodnar
Marta Bodro
Guillaume Body
Mathilde Body-Malapel
Knut Boe
Shaun Boe
Lucas Boeck
Pauline Boeckrxstaens
Karina Boege
Gregor Boehl
Susan K Boehlein
Alexandria Boehm
Daniela Boehm
Joachim Boehm
Ulrike Boehmer
Jan Boehnke

David Boehr
Jef Boeke
Kim Boekelheide
Michael Boele van Hensbroek
Arnout Boelens
Urs Boelsterli
Willehad Boemke
Guido Boening
Marianne Boeni-Schnetzler
Kristina Boerder
Marjan Boerma
Ferdinando Boero
Diederik Boertien
Donald Boesch
Claudia Boesmueller
Sanne Boesveldt
Angelika Boettger
Ryan Boettger
Alistair Boettig
Alistair Boettiger
Petra Boevink
Daiane Boff
Jean-Jacques Boffa
Mikhail Bogachev
Anthony Bogaert
Pierre Bogaerts
Alemtsehay Bogale
Jonathan Bogan
Giorgio Bogani
Jeffrey Bogart
Fausto Bogazzi
Christian Bogdan
Malgorzata Bogdan
Wieslaw Bogdanowicz
Sara Bögels
Willy Bogers
Ugo Boggi
Jose Boggia
José Boggia
Mary Boggiano
Henrik Bøggild
Nansi Boghossian
Barry Bogin
Carsten Bogler
Juliane Bogner-Strauss
Tanya Bogoslovsky
John A. Bogovic
Bjarte Bogstad
Ljiljana Bogunovic
Milena Bogunovic
Johannes Bohacek
Carolyn Bohach
Michelle Bohan Brown
Richard Bohannon
Jonathan Bohbot
Jason Bohland
Leah Bohle
Marc Bohlken
Holger Bohlmann
Jennifer Bohm
Anja Böhm
Ruwen Böhm
Stephan Böhm
Björn Bohman
Frank-D. Böhmer
Andreas Bohn
Erwin Bohn
Martin Bohn
Paul Bohn
Sarah Bohndiek
Nico Bohnen
Kyle Bohnert
Cara Bohon
Stefan Bohr
Ben Bohrer
Stefan Böhringer
Sean Bohun
Alessandro Boianelli
Michele Boiani
Isabelle Boileau
Adrien Boillot
Francois Boillot
Mirian Boim
Gilles Boire
Monica Boirivant
Matthieu Boisgontier
Detlev Boison
Sandrine Boisset
Marie-Christophe Boissier
Nathalie Boissot
Patrick Boissy
Scott Boitano
Jan Boitz
Heike Boivert
Guy Boivin
Xavier Boivin
Ester Boix
Mirela Bojan
Pavla Bojarova
Piotr Bojarski
Anders Bojesen
Stig Bojesen

Iva Bojic
Almuatazbellah Boker
Reinoud Bokkers
Thijs Bol
Gaby-Fleur Böl
David Bolam
Patrick Bolan
C Boland
Michael Boland
Pauline Boland
Sonja Boland
Carlos Bolanos
Francisco Bolas-Fernandez
Julie Bolcaen
Ingrid Boldin
Fernas Boldizsar
Paolo Boldrini
Annemarie Boleij
Annette Boles
Azam Bolhassani
Aline Boligon
Mohan Bolisetty
Subhashini Bolisetty
Michael Bölker
Roni Bollag
Wendy Bollag
Danushka Bollegala
Jerven Bolleman
Stephan Bolliger
Sveva Bollini
Paul Bollyky
Deborah Bolnick
Monique Bolotin-Fukuhara
Anne Isine Bolstad
Bart Bolsterlee
Ed Bolt
Andreas Bolte
Antoinette Bolte
Catherine Bolten
Kristy Bolton
Mark Bolton
Omotayo Bolu
Elisabeth Bolund
H.S. Bom
Johan Boman
Mauro Bombaci
Cristiano Bombardi
Jennifer Bomberger
Yannick Bomble
Morgane Bomsel
Jessica Bon
Elisa Bona
Robert Bonacci
Irene Bonaccorsi
Silvia Bonaccorsi
Juan Bonachela
Fabrizia Bonacina
Jim Bonacum
Maria Clara Bonaglia
James Bonaiuto
Carla Denise Bonan
Laura Bonanni
Daniel Bonanno
Joseph Bonanno
Giuliano Bonanomi
Sergio Bonaque-Gonzalez
Julius Bonart
I.A.S. Bonatelli
Maurizio Bonati
Mario Bonato
Diego Bonatto
Jiri Bonaventura
Rachel Bonawitz
Pedro Bonay
Bruno Bonaz
Humberto Boncristiani
Carol Bond
Gareth Bond
Jason Bond
Jason E. Bond
Michelle R. Bond
Peter Bond
Subbarao Bondada
Vladyslav Bondarenko
Robert Bonde
Claudine Bonder
Corina Bondi
Daniel Bone
Ivo Boneca
David Bonekamp
Patrizia Bonelli
Jaume Bonet
Billie Bonevski
Lynda Bonewald
Tracey Bonfield
Ricardo Bonfil
Patricia Bonfim-Mendonça
Brenda Bongaerts
Erik Bongcam-Rudloff
Malte Bongers
Marlies Bongers
Raoul Bongers

Massimo Bongiovanni
Vincent Bonhomme
Sonia Bonifacio
Heather Bonilha
Mary Bonin
Ferruccio Bonino
G. Bonkat
Maria Angeles Bonmati Carrion
Matthew Bonnan
Thomas Bonnard
Amélie Bonnefond
Mathilde Bonnefond
Eric Bonnefoy
Tyler Bonnell
Guusje Bonnema
Caroline Bonner
Daniel Bonner
Timethia Bonner
Florian Bönner
Susan Bonner-Weir
Cédrick Bonnet
Dominique Bonnet
Jacques Bonnet
Xavier Bonnet
Shilah Bonnett
Caterina Bonnin
Marcel Bonn-Miller
Céline Bonnyaud
Jeremy Bono
Silvia Bono
Renzo Bonofiglio
Francesca Bonomini
Adriana Bonomo
Elena Bonora
Stefanos Bonovas
Stephen Bonser
Maria Bonsignore
Louis Bont
Séverine Bontron
Rebecca Bonugli
Alexandre M.J.J. Bonvin
David Boocock
Jan Booij
Linda Booij
Angela Book
Michael Book
Jonathan Boomer
Scott Boomer
Mei Ying Boon
Mieke Boon
Wouter Boon
John Boone
Jelle Boonekamp
Arjan Boonman
Anthony Booth
Benjamin Booth
Carmen Booth
David Booth
Derek Booth
Josephine N. Booth
R. Booth
Stephanie Booth
Trevor Booth
Warren Booth
Thomas Boothby
Lynda Boothroyd
J. Boots
Hester Bootsma
George Booz
Rosemarie Booze
Alain Bopda Waffo
Melissa Bopp
Debajeet Bora
Namrata Bora
Anupom Borah
Alisdair Boraston
Ali Borazjani
Peter Borchardt
Jost Borcherding
Glen Borchert
Victor Borden
Christophe Bordi
Silvana Bordin
Michael Bordonaro
Liliane Borel
Nicole Borel
Erika Borella
Bridget Borg
Christophe Borg
Danielle Borg
Michael Borgas
Javier Borge-Holthoefer
Delphine Borgel
Peter Borger
Monique Borgerhoff Mulder
Luisa Borges
Thiago Borges
Henrique Borges da Silva
Jeff Borggaard
Tilman Borggrefe
Simone Borghesi
Anna Borghi

Elisa Borghi
Marta Borgi
Paola Borgiani
Anders Borgkvist
Wenche Borgnakke
Antonio Borgogni
Sara Borgomaneri
Giuseppe Boriani
Raphael Borie
Hynek Boril
Ljudmilla Borisjuk
Kathleen Boris-Lawrie
Vitaliy Borisov
Mark Borja
Anna Börjesson
Art Borkent
Gadi Borkow
Craig Borkowf
Ron Borland
James Borneman
Boris Bornemann
Jan Börner
Daniela Börnigen
Jan Bornschein
Uwe Bornscheuer
Brian Bornstein
Michael Bornstein
Stefan Bornstein
Steven Bornstein
Katy Borodkin
Abhijeet Borole
Eszter Boros
Peter Boross
Anton Borovjagin
Arielle Borovsky
Alexander Borowsky
Nica Borradaile
Maria Borrego
Antoni Borrell
Luisa Borrell
Sonia Borrell
Belinda Borrelli
Enrico Borrelli
Jon Borresen
Nicola Borri
Aiduan Borrion
Riccardo Borroni
Luigimaria Borruso
Pascal Borry
Denny Borsboom
Simone Borsci
Lubor Borsig
Ewa Borsuk
Mark Borsuk
Roque Bort
Alip Borthakur
Arianna Bortolami
Marta Bortoletto
Francesca Bortolotti
Stefania Bortolotti
Alejandro Bortolus
Marco Bortolus
David Borton
Stephen Bortone
Eric Bortz
Prachi Borude
Randy Borum
Stanislav Borysov
Jan Borysowski
Giuseppe Borzacchiello
Rosa Maria Borzi
Mauro Borzio
Dienke Bos
Elisabeth Bos
Kirsten Bos
Lieuwe Bos
Nico Bos
Wouter Bos
Pedro Boscan
Elisa Boscari
Vittorio Boscaro
Anna Bosch
Oliver Bosch
Rafael Bosch
Thijs Bosch
Giovanni Boschian
Julitta Boschman
Michael Boschmann
Francesca Boscia
Andrea Bosco
Gianfranco Bosco
João Bosco Pesquero
Francis Boscoe
B. Bose
Himangshu Bose
Jeff Bose
Kunal Bose
Thomas Bose
Michal Bosela
Biljana Mileva Boshkoska
Helena Boshoff
Paolo Bosi

Joseph Bosilevac
Adele Boskey
Maarten Bosland
Paul W. Bosland
Frank Bosmans
Johan Bosmans
Darko Bosnakovski
Andreas Boss
Emanuel Boss
Joseph Boss
Iris Bosschem
Jonathan Bossenbroek
Patrick Bosshart
Antonio Bossi
Elena Bossi
Paolo Bossi
Lara Bossini Castillo
Luciano Bosso
Xavier Bossuyt
Roger Bostelman
Chris Bostick
Julian Bostock
Christoffer Bostrom
Adrian Boström
Kim Boström
James Boswell
Kevin Boswell
Steven Bosworth
Ilze Bot
Attila Bota
Natalie Botchkareva
Revaz Botchorishvili
Ana Botelho
Joao Botelho
Maria Botelho
Monica Botelho
Roberto Botelho
Jose Botella
Rossini Botev
Graham Bothamley
Brian Bothner
Alfred L.M. Bothwell
Lewis Bott
Cristian Botta
Federico Botta
Luca Botta
Roberto Botta
Daniele Bottai
Daria Bottai
Donald Bottaro
Barbara Bottazzi
Chotima Böttcher
Cyrille Botté
Giovanni Bottegoni
Daniela Bottero
Bernd Böttiger
Andrea Bottino
Rita Bottino
Carezza Botto-Mahan
Camila Bôtto-Menezes
Jeremie Botton
Alexander Botzki
Magda Bou Dagher-Kharrat
Raed Bou Matar
Ayache Bouakaz
Hakim Bouamar
Jean-David Bouaziz
Hasna Boubakri
Jean-Philippe Bouchara
Luigi Bouchard
Maryse Bouchard
Jean-Philippe Bouchaud
Philippe Bouché
Yanis Bouchenak-Khelladi
Charles Boucher
Martin Boucher
Yves Boucher
Julie Bouckaert
Brendon Boudinot
Francois Boudreau
Jeanette Boudreau
Kevin Boudreau
Roland Bouffanais
Soufiane Boufous
Maria Bouga
Carole Bougault
Julia Boughner
Sabri Boughorbel
Berin Boughton
Marie-Elisabeth Bougnoux
Eléonore Bouguyon
Didier Bouhassira
Eric Bouhassira
Juliette Bouhours
Roger Bouillon
Pierre Bouillot
Ousmane Boukar
Bastiaan Boukens
Alexis Boukouvalas
Thierry Boulain
Martin Boulanger
Nathalie Boulanger

Pascale Boulanger
Hamid Boulares
Elizabeth Boulding
Normand Boule
Salix Boulet
Stéphanie Boulêtreau
Richard Bouley
Nicholas Boulis
Etienne Boulter
Luke Boulter
David Boulware
Louis Boumans
Farid Boumediene
Vassiliki Bountziouka
Aida Bouratbine
Nicolas Bourdel
A. Bourdin
Emmanuel Bourdon
Raymond Bourey
Anne Bourgarit Durand
Mickaël Bourge
Denis Bourgeois
Lelania Bourgeois
Marie Bourgeois
Stefan Bourgeois
Sacha Bourgeois-Gironde
Mickael Bourgoin
Jane Bourke
Virginie Bourlier
María Teresa Bourlón de los Ríos
Stylianos Bournazos
Salim Bourras
Franck Bourrat
Olivier Bourron
Teun Bousema
Chad Bousman
Bastien Boussat
Tatiana Bousse
Salah Boussen
François Boussin
Alex Boussioutas
Malaz Boustani
Jemel Boutheina
Stan Boutin
Angela Boutte
Thomas Boutton
Michael Bouvet
Anne-Marie Bouvier
Benjamin Bouvier
Corinne Bouvier
Michel Bouvier
A. Bouville
Andre Bouville
Willem H. Bouvy
Luc Bouwens
Fleur Bouwer
Abigail Bouwman
Hans Bouwmeester
Ian Bouyoucos
Thierry Bouyssou
Cecilia Bouzat
Mondher Bouzayen
Elie Bou-Zeid
Anne-Karine Bouzier-Sore
Marit Bovbjerg
Marco Bove
Fulvia Bovera
Alan Bovik
Christopher Bowd
Jocelyn Bowden
Mark Bowden
William Bowden
Scott Bowdridge
David Bowen
Deborah Bowen
Derrick Bowen
Holly Bowen
Mike Bowen
Spencer Bowen
William Bowen
Mark Bower
Peter Bower
John Bowes
Mike Bowl
Wadim Bowl
Matthew Bowler
Russel Bowler
David Bowles
Dawn Bowles
Devin Bowles
Robert Bowles
Barrett Bowling
A.S. Bowman
Alan Bowman
Andrew S. Bowman
David Bowman
Dwight Bowman
Gregory Bowman
Jeff Bowman
Leigh Bowman
Nicholas Bowman
Simon Bowman

Teresa Bowman
Kevin Bowyer
Susan Bowyer
Eric Boy
Kevin Boyack
Sarandeep Boyanapalli
L. Boyanova
Sébastien Boyas
John Boyce
Joshua Boyce
Peter C. Boyce
Richard Boyce
Walter Boyce
Anders Boyd
Andrew Boyd
Brian Boyd
Lara Boyd
Lesley Boyd
Michal Boyd
Norman Boyd
Robert Boyd
Ryan Boyd
Steven Kyle Boyd
Alexandre Boyer
Celia Boyer
Cherrie Boyer
Katharyn Boyer
Michael Boyer-Guittaut
Emma Boyland
Brad Boyle
Elizabeth Boyle
Jon Boyle
Laura Boyle
Michelle Boyle
Noel Boyle
Patrick Boyle
Sean Boyle
Theresa Boyle
Richard Boyles
Susan Boyle-Vavra
David Boynton
Sarah Boysen
Gurkan Bozdag
Serdar Bozdag
Mithat Bozdayi
Aline Bozec
Yves-Marie Bozec
Ioannis Boziaris
Alper Bozkurt
Marcelo Bozza
Marco Bozzali
Valentina Bozzetti
Yuri Bozzi
Sandra Braaf
Richard Braatz
Jan Brabek
William Bracamonte-Baran
Ana Paula Bracarense
Stefania Bracci
Selina Brace
Anthony Brach
Adelar Bracht
Elena Brachtel
Ken Bracke
Fiona Bracken
Chiara Braconi
Brad Brad
Mohamed Bradai
Isabel Bradburn
Louis Bradbury
Neil Bradbury
Oliver Braddick
Aaron Braddy
Andrew Bradford
Barry Bradford
Steven Bradfute
Cynthia Bradham
Charles Bradley
Darcy Bradley
Elizabeth Bradley
Graeme Bradley
John Bradley
Margaret Bradley
Robert Bradley
Sean Bradley
William Bradley
Shelton Bradrick
Amy Bradshaw
John Bradshaw
Pamela Bradshaw
Peter Bradshaw
William Bradshaw
L. Jeannine Brady
Matthew Brady
Dries Braeken
Ingrid Braenne
Stefan Braese
Caroline Braet
Alfésio Braga
Fernao Braga
José Braga
L.H. Braga

Raphael Braga
Rodrigo Braga
Janaina Braga do Carmo
Helga Bragadóttir
Nicola Bragazzi
Soren Brage
Darrin Brager
Anatol Bragin
Luca Braglia
Michel Brahic
Arun Brahma
Mayur Brahmania
Chloe Brahmi
Sheryl Brahnam
Janice Brahney
Nady Braidy
Stefanie Braig
David Brain
Matthew Brain
Antony Braithwaite
David Braithwaite
Victoria Braithwaite
Matthias Braito
Janina Brakel
Axel Brakhage
Marieta Braks
Julieta Brambila
Mattia Brambilla
Riccardo Brambilla
Viviana Brambilla
Clive Bramham
George Bramley
Charles Branas
Andrea Brancale
Vincenzo Brancaleone
Andrea Branch
Trevor Branch
Pascal Branchereau
Casey Branchini
Simone Branchini
Brett Branco
Rita Branco
Alfredo Brancucci
Denys Brand
Jeffrey Brand
Martin Brand
Matthias Brand
Nathan Brand
Philipp Brand
Randall Brand
Sarah Brand
Serge Brand
Tilman Brand
Karina Brandao
Michele A. Brandao
Ziany Brandao
Maria Brandão
Lars-Ove Brandenburg
W. Howard Brandenburg
Christian Brander
Keith Brander
Susanne Brander
Mirko Brandes
Ralf Brandes
Sebastian Brandhorst
Cristina Brandileone
Christopher Brandl
Martin Brandl
Simon Brandl
Pietro Brandmayr
Sabrina Brando
Amanda Brandon
Beate Brand-Saberi
Alexander Brandt
Curtis Brandt
Eric Brandt
Jessica Brandt
Kirsten Brandt
Laura Brandt
Mark Brandt
Renata Brandt
Roland Brandt
Steven Brandt
Paola Branduardi
Yaniv Brandvain
Wojciech Branicki
Marko Brankatschk
Jovan Brankov
Jessica Brann
Jonas Brannstrom
Meret Branscheidt
Fatima Brant
Sara Brant
Milam Brantley
Hargeet Brar
Jasmine Brar
Dawn Brasaemle
Jan Brascamp
Trevor Brasel
Esther Braselmann
Tom Brashers-Krug
Richard Brasington

Kevin Brasseur
Chad Brassil
Rebecka Brasso
Preston Bratcher
Rachel Brathwaite
Kaitlin Bratlie
Scott Bratman
Gennady Bratslavsky
Donna Bratton
Daniel Bratzke
Lisa Bratzke
Dale Bratzler
Jerome Braudeau
Dave Brauer
Jens Brauer
Matthew Brauer
Paula Brauer
Marie Brault
Chris Braun
Christina Braun
Clait Braun
David Braun
Dominique Braun
Edward Braun
Eyal Braun
Jerome Braun
Jonathan Braun
Jurgen Braun
Niko Braun
Sigurd Braun
Sigal Braun Miyara
Julia Braungart-Rieker
Gerhard Braus
Eric Braverman
Irus Braverman
Alberto Bravin
Alejandra Bravo
Fernando Bravo
Clinton Brawner
Emily Bray
George Bray
Signe Bray
David Brayden
F. Braza
Gintaras Brazauskas
Václav Brázda
Martin Brazeau
Nicholas Brazee
Reginaldo Brazil
Tomas Brdicka
Johanni Brea
Mariana Brea
Gloria Brea-Calvo
Gérard Breart
Siegmar Breckle
Annette Breckwoldt
Michael Breckwoldt
Eduardo Breda
Jan Bredow
Dara Bree
William Breed
Wout Breeman
Clarissa Breen
Daniel Breen
Lauren Breen
Leigh Breen
Elemi Breetvelt
J. A. Breeuwer
Simone Bregaglio
Thomas Bregenzer
Phil Bregitzer
Joana Brehm
Michael Brehm
Patrice Brehmer
Fred Breidt
Elizabeth Breininger
Denise Breitburg
Luana Cassandra Breitenbach Barroso Coelho
Fritz Breithaupt
Rainer Breitling
Ed Breitschwerdt
Gerda Breitwieser
Rolf Brekken
Andrew Breksa
Alison Breland
Anna-Katherine Brem
Henry Brem
Béatrice Brembilla-Perrot
Daniel Bremell
Erhard Bremer
Ross Bremner
Tatiana Bremova
Pierpaolo Brena
Victor Brena-Medina
Jeffrey Brender
Jay Brenman
J. Brenna
Alana Brennan
Brian P. Brennan
Irina Brennan
Keith Brennan

Leonard Brennan
Lisa Brennan
Marian Brennan
Patrick Brennan
Tim Brennan
Todd Brennan
Erin Brennand
Tracy Brennand
Christopher Brennan-Jones
Tara Brennan-Speranza
Georg Brenneis
Alina Brenner
Chad Brenner
Christoph Brenner
Darren Brenner
Eli Brenner
Lisa Brenner
Megan Brenner
Robert Brenner
Walburgis Brenner
Alexander Brenning
Colin Brent
David Brent
Gregory Brent
David Breshears
Fabiana Bressan
Patrick Brest
Stephane Bretagne
Laura Bretherton
Ghislain Breton
Juana Bretón López
Michael Brett
David Brett-Major
Christian Brettschneider
Julia Brettschneider
Corey Bretz
Frank Bretz
Stacey Bretz
Walter Bretz
Kai Breuhahn
C. Breuillard
Sophia Breusegem
Ingrid Breuskin
Thierry Brévault
Rossella Breveglieri
Damien Brevers
Marie Brevet
Bruce Brew
George J. Brewer
Marin Brewer
Michael Brewer
Molly Brewer
Tom D. Brewer
Warrick Brewer
Casey Brewster
Friedrich Breyer
Christine Breynaert
Rachel Breyta
Emilio Bria
Christopher Briand
Laurence Briant
Alex Briasoulis
Ana Bribian
Jane Brice
Dayna Brichta-Harhay
Noel Brick
Marguerite Brickman
Ian Bricknell
Juan Brida
Rob Briddon
Claire Bridel
Christy Bridges
Dave Bridges
Phillip Bridges
David Bridgett
David Brieber
Jason Bried
Olivier Briët
Michael Briga
John Brigande
Alberto Briganti
Angela Briganti
Adam Briggs
Brian Briggs
Derek Briggs
Robert Briggs
John Brigham
Filippo Brighina
Luis Brigido
Jeroen Brijs
Blandine Bril
Luca Brilante
David Briles
Daniel Briley
Florian Brill
Ilene Brill
Luca Brillante
Federico Brilli
Henrik Brinch-Pedersen
Ingar Brinck
Constance Brinckerhoff
Caterina Brindicci

Nicholas Brindle
Richard Brindle
Isabele Bringhenti
Andreas Bringmann
Laura Bringmann
Faiçal Brini
Antoinette Brink
LuAnn Brink
Signe Brinklov
Nicole Brinkmann
Martin Brinkworth
Christian Brion
François Brion
Gabriel Briones
Oscar Briones
Miguel Briones Salas
Anne Brisabois
Ryan Briscoe Runquist
Cathrin Brisken
David Briskey
Elizabeta Briski
Sylvain Brisse
Vania Brissos
Claire Bristow
Cristina Brito
Daniel Brito
Anne Britt
Jonathan Britt
Steven Britt
Evan Brittain
Kirsty Brittain
Aerika Brittian
Marcelo Britto
Adam Britton
Kate Britton
Sumudu Britton
Willoughby Britton
Anders Britze
Kevin Brix
Maria Felice Brizzi
Roland Broadbent
Matt Broadhurst
Gloria Broadwater
Maggie Broadwater
Karin Broberg
Patricia Brocardo
Stefania Brocca
Egidio Brocca-Cofano
Franck Brocherie
Bruno Brochet
Christopher Brochu
Douglas Brock
Matthias Brock
Theo Brock
Kenneth Brockman
Axel Brockmann
Kathrin Brockmann
Susan Brockmeier
Henry Brodaty
Hugh Broders
Seth Brodie
Anders Brodin
Thomas Brodnicki
Robert Brodschneider
Sergey Brodsky
Cláudia Brodskyn
André Brodtkorb
Alison Brody
David Brody
Howard Brody
Corey Broeckling
Kevin Broecks
Joost Broekens
Mans Broekgaarden
Niall Broekhuizen
Mark M.T.J. Broekman
Stefan Broer
Dieter Broering
María Noel Brogger
Juli Broggi
Simone Brogi
Christian Brogna
Heather Broihier
Caroline Bröjer
Barbara Bröker
Karl Albert Brokstad
Kristina Broliden
Jeffrey Bromaghin
Yana Bromberg
John Bromfield
Michael Bromley
Jon Brommer
Kristin Broms
Peter Bron
Annelies Bronckaers
Matt Brondum
David Broniatowski
Henrik Bronnum-Hansen
Guillaume Bronsard
Judith Bronstein
Francesco Bronzino
Cara Brook

David Brook
Greg Brooke
Sandra Brooke
Paul S. Brookes
Steven Brookes
Kathleen Brookfield
Andrew Brooks
Annabelle Brooks
Catherine Brooks
David Brooks
James Brooks
John Brooks
Kevin Brooks
Phillip Brooks
Rechele Brooks
Simon Brooks
Stephen Brooks
Susan Brooks
Jennifer Broom
Mark Broom
Patrick Brophy
Robert Brophy
Thomas Broquet
Ann-Christin Brorsson
Roland Brosch
Margaret Brosnahan
Sebastien Brosse
Maria Brossi
Florence Brossier
Hans Brostrom
Robert Brotherton
Carlos Brotons-Cuixart
Marco Brotto
Eugenia Broude
Laurent Broudiscou
George Broufas
Jonathan Brouillette
Gueorgui Broukhanski
Benedicte Brounais
Josiane Broussard
Louise Brousseau
Anne-Marie Brouwer
Melissa Brouwers
Sherryl Broverman
A.C. Brown
Adrian Brown
Andrew Brown
Angela Brown
Azby Brown
Candice M. Brown
Carolyn Brown
Charles Brown
Cheryl Brown
Christopher Brown
Chrysothemis Brown
Craig Brown
Culum Brown
Daniel Brown
Daren Brown
David Brown
Deborah Brown
Donald Brown
Graham Brown
Grant Brown
Gregory Brown
Hannah Brown
Hilary Brown
J. Brown
J. Quincy Brown
James Brown
Janine Brown
Jared Brown
Jason Brown
Jay C Brown
Jennifer Brown
Jeremy Brown
Juanita Brown
Judith Brown
Justin Brown
Karl Brown
Kim Brown
Larry Brown
Lawrence Brown
Lee Brown
Liana Brown
Lindsay Brown
Marybeth Brown
Matthew Brown
Michael Brown
Molly Brown
Monique Brown
Nicholas Brown
Nick Brown
Nina Brown
P. Brown
Patrick Brown
Paul Brown
Rachel Brown
Rafe Brown
Rebecca Brown
Russell Brown
Sara Brown

Scott Brown
Sheldon Brown
Stacy Brown
Stephen A. Brown
Steven Brown
Stuart Brown
Susan Brown
Terence Brown
Timothy Brown
Todd Brown
Wendy Brown
William Brown
Wilson Brown
Cameron Browne
Fiona Browne
Jessica Browne
Mark Anthony Browne
Christine Browne-Nunez
Carole Browner
Gina Brown-Guedira
Nancy Brown-Peterson
Jacob Brownscombe
Hal Broxmeyer
Suse Broyde
Anna Brozyna
Claudio Brozzoli
Roque Bru
Sonja Brubacher
David Bruce
Heather Bruce
Jason Bruce
Sharon Bruce
Toby Bruce
Marcel Bruchez
Wolfram Bruck
Rupert Bruckmaier
Peter Bruckner
Tim Bruckner
Sabrina Brückner
Yevgeny Brudno
Kristoffer W. Brudvik
Katrina Brudzynski
Michael Bruegger
Doerthe Brueggmann
Dennis Bruemmer
Johannes Brug
Ramon Brugada
Jean-Philp Brugal
Salvatore Brugaletta
Pedro Brugarolas
Bernard Brugg
Leslie Bruggeman
Holger Bruggemann
Radan Bruha
Alejandro Bruhn
Adriaan Bruijnzeel
Peter Bruins
Stanley Brul
Joshua Brumberg
Harry Brumer
David Brummell
Susanne Brummelte
Paola Brun
Philipp Brun
Thierry Brun
Giuseppe Brundu
Bernhard Brüne
Diede Brunen
Christophe Brunet
Frédéric Brunet
Laurence Brunet
Philippe Brunet de la Grange
Giacomina Brunetti
Natale Brunetti
Daniel Brunetto
Colby Brungard
Oliviero Bruni
Roberto Bruni
Tommaso Bruni
Kirstyn Brunker
Cornelia Brunner
J. Brunner
Peter Brunner
Agostino Bruno
Benedetto Bruno
D. Bruno
Davide Bruno
Ferry Bruno
Rafaela Bruno
Rosa Maria Bruno
Stefania Bruno
Tullia Bruno
Vincent Bruno
Andre Brunoni
Mabel Brunotto
Nico Bruns
Tony Bruns
Emily Brunson
Attila Brunyanszki
Stephen Brusatte
Lutz Brusch
Fabrizio Bruschi

Stefano Bruscoli
Thomas Brüser
Oystein Bruserud
Matthew Brush
Jeremy Bruskotter
Nele Brusselaers
Mariana Brussoni
Nickolay Brustovetsky
Birgitte Bruun
Jesper Bruun
Olivier Bruyère
Maria Bruzelius
Angela Bruzzaniti
Santina Bruzzone
Kristina Bry
A. Bryan
Arielle Bryan
David Bryan
Bruce Bryant
David Bryant
Joseph Bryant
Neil Bryant
Vaughn Bryant
Kelly Bryce
Thomas Bryce
Robert Brychta
Harry Bryden
Nicola Brydges
Anton Bryksin
Michal Brylinski
Eric Brymer
Randall Bryner
Mark Brynildsen
Marc Brysbaert
Barney Bryson
Mark Brzezinski
Edyta Brzoska
Ewa Brzozowska
Tomasz Brzozowski
Fengxiao Bu
Wen Bu
Zhongming Bu
John Buatti
Achim Bub
Luigi Bubacco
Omonigho Bubu
Maurice Bucagu
Gianpaolo Bucaneve
Filermon Bucardo
Annalisa Bucchi
Fabio Bucchieri
David Bucci
Maria Pia Bucci
Maria-Pia Bucci
Rosaria Bucci
Monica Bucciarelli
Paolo Bucciarelli
Stephan Buch
K. Buchacz
Alastair Buchan
Elizabeth Buchanan
John Buchanan
Kyle Buchanan
Thomas Buchanan
Tony Buchanan
David Buchbinder
Sibyl Bucheli
Daniel Bucher
Roman Bucher
Ralph Buchert
Bruno Buchholz
Malte Buchholz
Ursula Buchholz
Anatoly Buchin
Eckhart Buchmann
David Buchs
Henry Buchtel
Peter Buchwald
David Buchwalter
Friedrich Buck
Gregory Buck
Hudson Buck
Laura Buck
Matthias Buck
Ross Buck
Wolfgang Buckel
Jay Buckey
Karen Buckheit
Elizabeth Buckingham-Jeffery
Steve Buckland
Hallie Buckley
Hannah Buckley
Jenni Buckley
Laura Buckley
Mark Buckley
Rachel Buckley
Thomas Buckley
David Bucklin
Clarisa Buckner
Frederick Buckner
Wesley Buckwalter
R. Bucy

Silke Buda
John Budd
Salma Buddaseth
Katharina Budde
Matthew Budde
Bryce Buddle
Amit Budhraja
Mauricio Budini
Steven Budsberg
Luke Budworth
Jacek Budzynski
Bjoern Buehring
Mart Buekers
Ed Bueler
Alexander Buell
Joseph Buell
Jurgen Buenger
Borja Bueno
Carolina Bueno
Primitiva Bueno
Diana Bueno-Gutierrez
Bruno Bueno-Silva
Jason Buenrostro
Jens Buentzel
Jan Buer
Sebastian Buerklein
Kenneth Buetow
Christoph Buettner
Florian Buettner
Ilaria Bufalari
Jennifer Bufalo
Marina Bufarah
Veerle Buffel
Rochelle Buffenstein
Sara Bufferd
Annalisa Buffo
Barbara Buffoli
Mariano Buffone
Mariano Buffone Buffone
Gesine Bug
I. Bugaighis
Aurelia Bugaiska
Marie Bugarel
Shawn Bugden
Stephanie Bugden
Timothy Bugg
Heiko Bugger
Elisabetta Bugianesi
Marco Bugliani
Magdalena Bugno
Thomas Bugnyar
Beáta Bugyi
David Bühlmann
Christoph Bührer
Catalin Buhusi
Tuan Bui
Ruud Buijs
Moniek Buijzen
Louis Bujan
Luis Bujanda
Raymond Bujdoso
Anna Bukiya
Alexander Bukreyev
Michael Bukrinsky
Anna Bulanova
Mofijul Bulbul
Joseph Bulbulia
Silvia Bulfone-Paus
Cynthia Bulik
Ian Bull
James Bull
Michael Bull
Christian Büll
Jan Bulla
Kelli Bullard Dunn
Lars Bullinger
Monika Bullinger
Andrew Bulmer
Rebecca Bulotsky Shearer
Hannes Bülow
Daniel Bulte
Gopalakrishnan Bulusu
Ozgur Bulut
Ham Bumsub
J. Scott Bunch
Martin Bunch
Wendell Bunch
Madeleine Bunders
Lukas Bündgens
Mirco Bundschuh
Victoria Bunik
N.F. Bunkin
Andy Bunn
Eric Bunn
Bruce Bunnell
Filiz Bunyak Ersoy
Samantha Bunzli
Marine Buon
Tammy Buonasera
Pasqualina Buono
Francesco Buonocore
Danilo Buonsenso

Xabier Buque
F. Burada
Yoram Burak
Charles Burant
Vito Burasco
Hernan Burbano
Sara Burch
Danielle Burchett
David Burchfield
Denise Burchsted
Hynek Burda
Lyudmila Burdelya
Christy Burden
Edwin Burdette
Joanna Burdette
Jonathan Burdette
Monica Burdick
Tricia Burdo
Kathryn Burdon
Alex Burdorf
Ronan Bureau
Esther Buregyeya
Niclas Burehult
Vladimír Bureš
Andre Buret
Sarah Burgard
Concetta Burgarella
F. Burgaya
Jeffrey Burgdorf
Colleen Burge
Pierre-Régis Burgel
Sebastian Bürgel
Adam Burgener
Ben Burger
David Burger
Huibert Burger
Martin Burger
Pamela Burger
Erik Burgerhout
Adam Burgess
Andrew Burgess
Caroline Burgess
Graham Burgess
Karl Burgess
Malcolm D Burgess
Philip Burgess
Treena Burgess
Jacqueline Burgette
Warren Burggren
Robert Burghardt
Arthur Burghes
Giovanni Burgio
Patrick Burgon
Nilda Burgos
John Ashley Burgoyne
Louise Burgoyne
Robert Burgoyne
Joerg Burgstaller
Eric Burguiere
Maggie Burhans
Michele Burigo
Antje Burke
Christopher Burke
Darren Burke
Gaelen Burke
Ian Burke
John Burke
Mark Burke
Richard Burke
Robert Burke
Thomas Burke
Wylie Burke
Brian Burkel
Kent Burkey
Peter Burkhard
Brant Burkhardt
Thomas Burkholder
Michael Burkitt
Laura Burkle
Melissa Burkley
Howard Burkom
A. Wesley Burks
Deborah Burks
Romi Burks
William Burlingham
Amanda Burls
Michael Burman
Anke Burmester
Robert Burnap
Michael Burne
Phil Burnet
Barrington Burnett
Karen Burnett
Sandra Burnett
Robyn Burnham
Michel Burnier
Anne-Françoise Burnol
Anthony Burns
Brendan Burns
Christine Burns
Darren Burns
Elaine Burns

Marie Burns
Michael Burns
Phillipa Burns
Ryan Burns
Sean Burns
Tom Burns
Kerry Burnstein
Geoffrey Burnstock
Alberto Búrquez
Jamie Burr
Sarah Burr
Thomas Burr
Bryna Burrell
Louise Burrell
David Burris
Zair Burris
Carole Burrow
Casey Burrows
Vincent Burrus
Jirí Burša
Tal Burstyn-Cohen
Bryan Burt
Gordon Burtch
Stéphane Burtey
Chris Burtin
Charles Burton
Christie Burton
Elissa Burton
James Burton
Louisa Burton
Mark Burton
Michael Burton
Rachel Burton
Ron Burton
Britt Burton-Freeman
Doris Burtscher
Martin Burtscher
Chris Burwell
Michael Burwinkel
Benjamin Burwitz
Sakib Burza
Artur Burzynski
Vincent Bus
Pierpaolo Busan
Francesco Busardo
Andrew Busch
Hans Jörg Busch
Lawrence Busch
Michael Busch
Robert Busch
Theresa Busch
Marc Aurel Busche
Konrad Buscher
Philippe Büscher
Ivo Buschmann
Michael Buschmann
Thomas Busey
Daniel Bush
Jon Bush
Ronald Bush
Stephen Bush
William Bush
Kathryn Bushley
Frederic Bushman
Wade Bushman
Ttamara Bushnik
Florent Busi
Julia Busik
Doreen Busingye
Orion Buske
Erik Buskens
Susan Buskin
Jitka Buskova
Sandra Bussadori
James Bussel
Jean-François Bussières
Jan Bussink
Juan Busso
Federico Bussolino
Cyrill Bussy
Alejandro Bustamante
Eduardo Bustamante
Hedie Bustamante
M.A. Bustamante
Rodrigo Bustamante
Joaquim Bustorff-Silva
Martha Bustos
Ricardo Bustos Guajardo
Joanna Busza
Tim Buszard
Sachit Butail
Sonia Butalia
Jon Butchar
Jonathan Butchar
Matthew Butchbach
Jonathan Butcher
Rebecca Butcher
Marie-Jose Butel
Micaela Butele
Saulius Butenas
Alexandra Butler
Erin Butler

John Butler
Mark Butler
Mary Butler
Noah Butler
Stephen Butler
Javier Butragueño
Melinda Butsch Kovacic
Elke Butt
Kevin Butt
Gabriele Buttafuoco
Brigitta Buttari
Bettina Buttaro
Manish Butte
Colin Butter
Stephen Butterfill
Brian Butterworth
Valentina Buttiglione
Isabella Buttino
Mathias Buttmann
Oliver Büttner
Sabrina Büttner
Mark Button
Judith Buttriss
Ljubomir Buturovic
Alexander Butwick
Bianca Buurman
Iain Buxton
Nur Buyru
Wouter Buytaert
Ender Büyükgüzel
Nasuh Buyukkaramikli
Ozlem Buyuktanir
Biljana Buzadzic
Anton Buzdin
Lubos Buzna
Simone Buzwell
Marguerite Buzza
Carlotta Buzzoni
Peter Byass
Seth Bybee
Sergio Bydlowski
Ali Bydon
James Byers
Richard Byers
Craig Byersdorfer
Siarhei Bykau
Donita Bylski-Austrow
Emanuel Bylund
Margaret Bynoe
Siddappa Byrareddy
Desiree Byrd
Nicholas Byrd
Todd Byrem
Adam Byrne
Dallan Byrne
Elaine Byrne
Gerard Byrne
Jason Byrne
Maria Byrne
Richard Byrne
Scott Byrne
Thomas Byrne
Graham Byrnes
Mark Byrnes
Adam Byron
Egle Bytautiene Prewit
Jinyoung Byun
Wonwoo Byun
John C Markley
Shiny C.
Mirte Caanen
Armando Caballero
Primitivo Caballero
Guillaume Cabanac
Ioav Cabantchik
Jacques Cabaret
Francis George Cabarle
Aderville Cabassi
Julieta Cabello
Nora Caberoy
Oscar Cabezón
Manuela Cabiati
Simona Cabib
Florence Cabot
Antonio Cabrales
Pedro Cabrales
Patricia Cabrera
Antonio Cabrera de León
Delia Cabrera DeBuc
Giulio Cabrini
Nadia Caccamo
Laura Cacciani
Francesco Cacciatore
Michael Cacciatore
Santa Olga Cacciola
Fabio Caccioli
Carlos Caceres
Nicolas Cachanosky
Fidel Cacheda
Arnaud Cachia
Anne Caclin
Patrice Cacoub

Dan Cacsire Castillo-Tong
Massimiliano Cadamuro
Fernando Cadaveira
Joseph Cadden
Carla Caddeo
Jean Lud Cadet
Ken Cadigan
Bilge Cadirci
Eduardo Cadore
Ruggero Cadossi
Monique Cadrin
Ken Cadwell
Catia Caeiro
Alexandre Caetano
António Caetano
Laurence Caeymaex
Cecilia Café
Monica Caffara
Carlo Caffarelli
Paolo Caffarra
Stefano Caffarri
Anwen Caffell
William Cafferty
Barbara Caffery
Michael Caffrey
Tara Caffrey
Mauricio Cafiero
Eilidh Cage
Giuseppina Caggiano
Lucio Cagini
Nazan Caglar
Hayriye Cagnan
Jonathan Caguiat
Alex Cahana
Catherine Cahill
Katelyn Cahill-Rowley
Pedro Cahn
Wiepke Cahn
Monika Cahova
Niken Cahyani
Benzhi Cai
Chen-Leng Cai
Fei Cai
George Cai
Guoping Cai
Haoyang Cai
Hong Cai
Hongmei Cai
Huajian Cai
James Cai
Jian-Chun Cai
Jianfeng Cai
Jingli Cai
Jinhua Cai
Jiyang Cai
Kaiquan Cai
Lei Cai
Li Cai
Li Zhe Cai
Lu Cai
Ming Cai
Mingyong Cai
Qingqiong Cai
Ruichu Cai
Shanshan Cai
Shengguan Cai
Shi-Min Cai
Shuowei Cai
Tie Cai
Wanzhi Cai
Wei Cai
Weijia Cai
Weiming Cai
Weixing Cai
Wenfeng Cai
Wenhua Cai
Xiao-Hong Cai
Xiong Cai
Xiujun Cai
Xuehui Cai
Xueya Cai
Xuwang Cai
Yan Cai
Yingyun Cai
Yiyong Cai
Yongli Cai
Yu-Dong Cai
Zhenguang Cai
Zhiqiang Cai
Waleska Caiaffa
Gianluca Caiazzo
Ana Caicedo
Carlos Caicedo Bastidas
Romain Caillard
Maxime Cailleret
Jocelyne Caillon
Melissa Caimano
Antoni Caimari
Florian Caiment
Kristal Cain
Lauren Cain
Ricky Cain

Saverio Caini
M. Cecilia Caino
Simonetta Caira
Bruce Cairns
Rob Cairns
Francesco Cairo
Gaetano Cairo
Beth Caissie
Li Caiwen
Bulent Cakmak
Sabit Cakmak
Dimitrios Cakouros
Steven Cala
Evan Calabrese
Francesca Calabrese
Joseph Calabrese
Vittorio Calabrese
Ricardo Calado
Gloria Calaf
Riccardo Calafiore
Philippe Calain
Gemma Calamandrei
Ivan Calandra
Sebastiano Calandra
John Calarco
Victor Calatayud
Sebastien Calbo
Andrea Calcagno
Maria Calcagnotto
Rebecca Calcott
Mick Calcutt
Nigel Calcutt
Camila Caldana
Guido Caldarelli
Danielle Gregorio Caldas
Joana Caldeira
Maria Caldeira
Tina Calderon
Rosario Calderón
Richard Calderone
Lilian Calderón-Garcidueñas
Ronit Calderon-Margalit
David A Calderwood
Trinidad Caldés
Marco Caldin
Josep Calduch-Giner
Charles Caldwell
Heather Caldwell
Kevin Caldwell
Fabio Calefato
Federico Calegari
Richard Calendar
Clément Calenge
Miguel Calero
David Calhoun
Vince Calhoun
Rudy Calif
Maria Caligo
Ali Calik
Michael Calik
Mariafe Calingacion
Paolo Calistri
Marcus Calkins
Douglas Call
Jarrod Call
Melissa Call
Julie Callaert
Brian Callaghan
David Callaghan
Alison Callahan
Benjamin Callahan
Hilary Callahan
Giuliano Callaini
Marie-Pierre Callait-Cardinal
Akiko Callan
Anna Callan
Mitch Callan
Denton Callander
Andrew Callaway
Clifton Callaway
Juan C. Calle
Leonardo Calle
Juan Antonio Calleja
Jean Calleja-Agius
Daniel Calleri
Patrick Callery
Bert Callewaert
Fabrizio Calliada
Taylor Callicrate
Viviane Callier
Judy Callis
Patrik Callis
Michele Callisaya
Sebastian Calonico
Enrica Calura
Vincenza Calvaruso
Sarah Calve
Michael Calver
Aleix Calveras
Geoff Calvert
D. Calvet
Juan Calvete

Sébastien Calvignac-Spencer
Cinzia Calvio
Diego Calvisi
Cristina Calvo
Maria Angeles Calvo
Andrea Calvo Echenique
Beatriz Calvo-Merino
Jorge Calzada
Luigino Calzetta
Enrico Calzia
Mattia Calzolari
Laurence Calzone
Hakan Cam
Anton Camacho
Antonio Camacho
Francisco Camacho
Macario Camacho
Rafaela Camacho-Bejarano
Simonetta Camandola
Amadou Camara
Estela Camara
Diana Câmara
Joan-Gerard Camarena
Arley Camargo
Carlos Camargo
Ilana Camargo
Carlos Camargo Jr.
Andrea Camattari
Alice Cambiaghi
Giulia Cambie
Marie Anne Cambon-Bonavita
Gilles Cambonie
Lucieli Cambri
Erik Cambria
Edward Cambridge
Geraldine Cambridge
Emanuela Camera
Gabriele Camera
Giulia Camerino
Andrew Cameron
Catherine Ann Cameron
Chris Cameron
Daniel Cameron
Donnie Cameron
Heather Cameron
Mark Cameron
Michael Cameron
Nicole Cameron
Paul Cameron
Robert Cameron
Tim Cameron
Ross Camidge
Camilla Camilla Nøjgaard
Jeremy Camilleri
Marco Camilli
Carlo Camilloni
José Caminero
Emily Camm
G. Cammarota
Giovanni Cammarota
Marco Cammisa
Sharon Cammisuli
Ernest Camp
Giuseppe Campagna
Leo Campagna
Aurélie Campagne
Pascal Campagne
Fernanda Campagnollo
Luisa Campagnolo
Gianluca Campana
Michael Campana
Steven Campana
Joseph Campanale
Juan Campanario
Stefano Campanaro
Floriana Campanile
B. Campbell
Bruce Campbell
Colin Campbell
Daniel Campbell
Doris Campbell
Eleanor Campbell
Graeme Campbell
Hamish Campbell
Ian Campbell
Jacquelyn Campbell
Jay Campbell
Joe Campbell
John Campbell
Jonathan Campbell
Jos Campbell
Justin Campbell
Kaitlin Campbell
Lesley Campbell
Lindsay Campbell
Lisa Campbell
Marnie Campbell
Matthew Campbell
Paul Campbell
Pauline Campbell
Roderick Campbell
Sara Campbell

Thomas Campbell
W. Campbell
William Campbell
Martha Campbell-Thompson
Esther Camp-Dotlic
Silvia Campello
Matthew Campen
Stefano Campi
Enio Campiglia
Lenea Campino
Silvia Campioni
Giuseppina Campisi
Guillermo Campitelli
Gianluca Campo
Orlando Campolo
Carlos Campos
Daniel Campos
Erika Campos
Francisco Campos
Joaquin Campos
Marco Campos
Maria Campos
Mario Campos
Mateus Campos
Nicole Campos
Rodolfo Campos
Sara Campos
Eric Campos-Canton
Raquel Campos-Herrera
Jaime Campos-Valenzuela
Tomàs Camps
Susana Campuzano
Lamberto Camurri
Christophe Camus
Melinda Camus
Stéphane Canaan
Javier Cañada
David Canaday
Erick Canales-Rodríguez
Mauricio Canals
Fatih Canan
Adriana Canapa
Bruno Canard
Adelino Canário
João Canário
Rafael Cañas
Duran Canatan
Solange Canavarro
Sule Canberk
Carlos Cançado
Jessica Cance
William Cance
Laura Cancedda
Juan Cancino-Diaz
Demet Candas
Klemen Candek
Angel Candela
Pietra Candela
E. Candi
Simona Candiani
Matteo Candidi
Rowan Candy
Jim Cane
Rossella Canese
Daniela Canestrari
Emmanuelle Canet-Soulas
Pietro Canetta
Claudio Canetti
Giulia Caneva
Silvana Canevari
Amy Canevello
Richard Canfield
Gerard Cangelosi
Patrice Cani
Carolina Caniffi
E.C. Caniglia
Antonella Canini
Laetitia Canini
Rita Canipari
Michel Canis
Adrian Canizalez-Roman
Mayilee Canizares
Ali Cankaya
Ozden Canli Tasar
Barbara Canlon
Christine Canman
Marissa Cann
James Cannady
Angela Cannas
Alessandro Cannavo
Belinda Cannell
Mark Cannell
Stefano Cannicci
Carlo Vittorio Cannistraci
Barbara Cannon
Jack Cannon
Jason Cannon
Mary Cannon
Robert Cannon
Jorge Cano
Maria Isabel Cano
Miguel Cano

Cristina Canova
Dexter Canoy
Ugur Canpolat
José Cansado
John Cant
Juan Cantalapiedra
Gaetano Cantalupo
Giulio Erberto Cantarella
Francesco Paolo Cantatore
Roberto Cantello
Jaime Cantera
Chiara Cantiani
Edouard Cantin
James Cantley
Carlos Canto
Efrain Canto-Lugo
Gador Canton
Margherita Cantorna
Brendan Cantwell
Marta Canuti
Maria Canziani
Elisa Canzoneri
Bangwei Cao
Bing-Yan Cao
Bo Cao
Deliang Cao
Dianjun Cao
Erhu Cao
G.S. Cao
Guangzhong Cao
Guohua Cao
Hong Cao
Hongnan Cao
Hongsheng Cao
Hongxin Cao
Hua Cao
Jia Cao
Jianping Cao
Ji-Min Cao
Jin Cao
Jun Cao
Junli Cao
Junzhe Cao
Linlin Cao
Liping Cao
Liyong Cao
Miao Cao
Qian Cao
Qianzhong Cao
Qingjiu Cao
Qiufen Cao
Sanjie Cao
Shaunghe Cao
Shuanghe Cao
Shujuan Cao
Shunan Cao
Siyan Cao
Tian Cao
Tongyu Cao
Wei Cao
Weibiao Cao
Xiaoyan Cao
Xu Cao
Yang Cao
Yong Cao
Yue Cao
Zhen Cao
Zhigang Cao
Zhiwei Cao
Van-Mai Cao-Lormeau
Andrew Cap
Jeffrey Capadona
Colin Capaldi
Deborah Capaldi
Marinela Capanu
Esther Caparros
Antonio Caparrós Ruiz
Cristian Capasso
Jacqueline Capeau
Blanche Capel
Delphine Capela
Carlo Capelli
Gioia Capelli
Isabella Capellini
Daniel Capelluto
Encarnación Capilla
Nazzareno Capitanio
Christian Capitini
Jeff Caplan
Joel Caplan
Gina Caplen
Jonathan Caplin
G. Caplovitz
Enrico Capobianco
Vincenza Capone
Riccardo Caponetto
Daniela Caporossi
Giuseppe Capovilla
Yvan Capowiez
Alejandra Capozzo
Celine Cappe
Joseph Cappella

Angélica Cappellari
Julien Cappelle
Gianni Cappelli
Germana Cappellini
Maria Cappello
Angela Capper
Carolina Cappi
Francesca Cappitelli
Shannon Capps
Francesco Cappuccio
Justin Cappuzzo
John Capra
Valeria Capra
Laura Capranica
Valerio Capraro
Marco Caprini
Enrico Caprio
Massimiliano Caprio
Michael Caprio
Flavio Caprioli
Joseph Caprioli
Myles Capstick
Stuart Capstick
Ana Capuano
Margareth Capurro
Josip Car
Luis Caraballo
Rey Carabeo
Hélène Carabin
Barbara Caracciolo
Alfonso Caracuel
Caroline Caradu
Eric Caragata
Doina Caragea
Michele Caraglia
Martin Caraher
Paola Caramaschi
Beatriz Caramés
Gaetano Caramori
Marcelo D. Carattino
Anibal Carbajo
Ester Carballo-Jane
Claus-Cristian Carbon
Anna Carbone
Federico Carbone
Giuseppina Carbone
Larry Carbone
Marco Carbone
Pablo Carbonell
Xavier Carbonell
Franck Carbonero
Etienne Carbonnelle
Andrea Carcelen
Daren Card
Roderick Card
Ring T. Carde
Michelle Cardel
Marcus Carden
Etzel Cardeña
Andres Cardenas
Noemi Cardenas
Paco Cardenas
Susana Cardenas
Rafael Cárdenas
Julio Cardenas-Rodriguez
Alessio Cardillo
Fabiola Cardillo
Danilo Cardim
Rhonda Cardin
Francesco Cardinale
Marco Cardinale
Massimiliano Cardinale
Vincenzo Cardinale
Pablo Cardinal-Fernández
Beatrice Cardinali
Giorgia Cardinali
Flavia Cardini
Genis Cardona
Kenneth Cardona
Narcis Cardona
Silvia Cardona
Magnolia Cardona-Morrell
Amanda Cardoso
C.R. Cardoso
Claudia Cardoso
Danon Cardoso
Hugo Cardoso
Josiane Cardoso
Luiz E.M. Cardoso
M. Jorge Cardoso
Mayra Cardoso
Sandra Cardoso
Susanna Cardoso
Tereza Cardoso
Erwing Cardozo
Richard Cardullo
Kitty Cardwell
Vincent Careau
Marie-France Carette
Kathleen Carey
Susan Carey
Fabio Carfagna

Aaron Cargile
Jessica Carilli
Petronila Carillo
Paul Carini
Alexandre Carisey
Dan Cariveau
Maria Carlan Silva
Carsten Carlberg
Corinne Carle
Georges Carle
Cristel Carles
Fabio Carletti
Sanya Carley
Giancarlo Carli
Amy Carlile
Angela Carlin
Bradley Carlin
Frederic Carlin
Leo Carlin
Gregory Carling
Celia Carlini
Raul Carlini
Waldemar Carlo
Francesca Carlomagno
Gianfranco Carlomagno
Daniela Carlos
Schenck Carlos
Silvia Carlos
Carmelo Carlo-Stella
John Carlquist
Anthony Carlsen
Andrew Carlson
Christina Carlson
David Carlson
Emily Carlson
J. Andrew Carlson
Jenny Carlson
Kim Carlson
Matt Carlson
Nicholas Carlson
Paul Carlson
Susan Carlson
John Carlsson
Katarina Steen Carlsson
Kathy Carlstead
James Carlton
Jill Carlton
James Carlucci
Marcos Carlucci
Erberto Carluccio
Jason Carlyon
George Carman
John Carman
Yehuda Carmeli
David Carmena
Ruth Carmichael
Thomas Carmichael
J. Bryan Carmody
Kendra Carmon
Adriana Carmona
F. David Carmona
Ana Maria Carmona-Ribeiro
Carmelo Carmona-Rivera
Andrea Carmone
Gilles Carnac
Everardo Carneiro
Mariângela Carneiro
Regina Carneiro
Amancio Carnero
Andrés Carnero
Andrew Carnes
Molly Carnes
Mercedes Carnethon
Vincenzo Carnevale
Luca Carnevali
Dana Carney
Carla Carnovale
Tim Caro
James Carolan
Maria Cristina Caroleo
Alexandre Caron
Erik Caroselli
Francesco Carotenuto
Ylenia Carotenuto
Berit Carow
G. Carpagnano
Guy Carpenter
Margaret Carpenter
Ryan Carpenter
Susan Carpenter
Robert Carpenter Jr.
Arnaud Carpentier
Giulia Carpinelli
Guido Carpino
Pedro Carpintero
Daniel Carr
Ian Carr
Jillian Carr
John Carr
Norman Carr
Vaughan Carr
Serena Carra

Arkaitz Carracedo
Camillo Carrara
Verena Carrara
Raffaele Carraro
Gonzalo Carrasco
Jose Luis Carrasco
Miguel Carrasco
Pilar Carrasco-Garrido
Montserrat Carrasco-Triguero
Alfredo Carrato
Madeleine Carreau
Margaret Carrel
Tania Carreon
Helaine Carrer
Ana Carrera
Constanza Carrera
Mariana Carrera
Olaia Carrera
Elena Carreras
Danielle Carrick
Emma Carrick
Adam Carrico
João Carriço
Nuria Carriedo
Lucie Carrier
Marc Carrier
Rebecca Carrier
Frederic Carriere
Yves Carriere
Patrizia Carrieri
Eugenia Carrillo
Enrique Carrillo-de-Santa-Pau
Maria Carrillo-Sepulveda
Suzanne Carrington
Jose Carrion
Jerome Carriot
Juan Carrique-Mas
Belén Carro
Andrew Carroll
Dan Carroll
E. Carroll
John Carroll
Joseph Carroll
Kecia Carroll
Robert Carroll
Rosemary Carroll
Philip Carrott
Alessandro Carrozzo
Marco Carrozzo
Laura Carruth
Vern Carruthers
James Carson
William Carson
William Carson IV
Andrew Carson-Stevens
Maren Carstensen
Tina Carstensen
Chris Carswell
Angelino Carta
Anna Carta
Alexander Cartagena-Rivera
Monica Cartelle Gestal
Allison Carter
Arron Carter
Carol Carter
Clay Carter
Clive Carter
Edward Carter
Gwendolyn Carter
Jane Carter
K.C. Carter
Neil Carter
Robert Carter
Stephen Carter
Stuart Carter
Joan Cartes
Michael Carty
Marie-Line Caruana
Michele Carugno
Bethany Caruso
Geoffrey Caruso
Maria Vittoria Caruso
Ana Carvajal
Agostinho Carvalho
Airton Carvalho
Antio Carlos Carvalho
Antonio José Carvalho
Brendan Carvalho
Celso Carvalho
Claudia Carvalho
Daniel Carvalho
David Carvalho
Eneas Carvalho
Ines Carvalho
Livia Carvalho
Luis Carvalho
Luisa Carvalho
Marilia Carvalho
Sandra Carvalho
Thiago Carvalho
Marco Carvalho-Filho
Cláudia Carvalho-Santos

Charles Carver
Peggy Carver
Steve Carver
Angela Carville
Jeffrey Cary
Ray Cas
Rita Casadio
Valentina Casadio
Santiago Casado
Nicola Casagli
G. Casal
Manuele Casale
Paolo Casale
Paolo Casali
Francesca Casalini
Enrique Casalino
Núria Casals
Climent Casals-Pascual
Amelia Casamassimi
Beata Casanas
Ramon Casanova
Rubén Casanova-Sáez
Dulce Elena Casarini
Alejandro Casas
Ana Casas
Jérome Casas
Lidia Casas
Mariana Casas
Laura Casas Castano
Ana Casas Guijarro
Fabián Casas-Arenas
Jose Casasnovas
Giovanni Casazza
João Cascalheira
Marilia Cascalho
Raffaella Cascella
Filippo Caschera
Alessandro Cascioferro
Stella Cascioferro
Adam Case
Elizabeth Case
Laura Case
Natasha Case
Trevor Case
Chiara Caselli
Elisabetta Caselli
Robert Casero
Paolo Caserotti
Aleix Cases
Lachlan Casey
Patrick Casey
Rachel Casey
Sara Casey
Tristan W. Casey
Vincent Casey
Robin Cash
Sean Cash
Neil Cashman
Eduardo Casilari
Antonino Casile
Sandra Casimiro
Michele Casini
Simona Casini
Fran Casino
Francesca Casiraghi
Sherwood Casjens
Michael D. Casler
Antonella Casola
Laura Casorzo
Tamara Caspary
Brandon Casper
Corey Casper
Markus Casper
J. Caspers
Svenja Caspers
John Caspersen
Rachel Caspi
Alexandra Cassado
Nicola Cassanelli
Marlene Cassar
Anne-Marie Cassard
Marco Cassatella
Flemming Cassee
Jean-Christophe Cassel
Margaretha Casselbrant
Filip Casselman
Raul Cassia
Brittany Cassidy
Sophie Cassidy
Corinne Cassier-Chauvat
Jean-Jacques Cassiman
Patricia Cassina
Valeria Cassina
Alessandro Cassini
Nadim Cassir
Antonio Carlos Cassola
Antonio Cassone
Vincent Cassone
Antonella Castagna
Maria Grazia Castagna
Tamara Castaneda
Carlos Castañeda-Orjuela

Diego Castanera
Jessica Castanheira
Pedro Castanheira
Andréa Castanho
Camila Castanho
Emanuele Castano
Juan Castano
Rene Castelein
Stefanie Castell
Marc Castella
Carlo Castellani
Giuseppe Castellano
Gustavo Castellano
Isabella Castellano
F. Xavier Castellanos
Maria Clara Castellanos
Milagros Castellanos
Esther Castellano-Sanchez
Gustavo Castellanos-Galindo
Francesco Castelli
Joël Castelli
Luigi Castelli
Giovanni Castellini
Robert Castellino
Alfredo Castello
Jose Castellote
Lana Castellucci
Barbara Castelnuovo
Robert Castelo
Serenella Castelvecchio
Laurent Castera
Eric Castet
Umberto Castiello
Alessandro Castiglione
Kathrin Castiglione
Stefano Castiglione
Alessandra Castiglioni
Isabella Castiglioni
Patrice Castignolles
P.C. Castilho
Rita Castilho
Rocio Castilla
Andrea Castillo
Jorge Castillo
José Castillo
Karl Castillo
Pablo Castillo
Raquel Castillo-Contreras
Ricardo Castillo-Galvan
Jose Castillo-Mancilla
Steven Castle
Erica Casto
Angela Castoldi
Carlos Castorena
Gabriella Castoria
Maija Castren
Carmen Castresana
Eduardo Castrillon
Alexandre Castro
António Castro
Clara Castro
Estrella Castro
Gabriel Castro
Helena Castro
I.T. Castro
Ilse Castro
Jason Castro
L. Filipe Castro
Maite Castro
Mariana Castro
Michelle Castro
Newton Castro
Paula M.L. Castro
Rui Castro
Maria Castro Codesal
Francis Castro Paz
Ana Castro-Avila
David Castro-Diaz
Hugo Castro-Faria-Neto
Jesús Castro-Marrero
Adelaida María Castro-Sánchez
Sebastian Casu
Manuela Casula
Clayton Caswell
Neus Català
Raquel Català
Ferrán Catalá-López
Núria Catalán
Maria Graziella Catalano
Patrick Catalano
Santiago Catalano
Angel Cataldi
Annalisa Cataldi
Sule Cataltepe
Maria Valeria Catani
Francesco Catania
Ilaria Catapano
Antonino Catara
Brian Catchpole
Dolores Catelan
Carolyn Cates
Dominique Cathelin

Arnaud Catherine
Dodds Catherine
Andrew Cato
Andrea Catorci
Janet Catov
Sergiu-Bogdan Catrina
Teresa Catry
Zaira Cattaneo
Xavier Catteau
Andrea Cau
Lester F. Caudill Jr
Abigail Caudle
William Caudle
Christelle Cauffiez
George Caughey
Linda Cauley
Omar Cauli
Louise Causer
Felipe Cava
Lisa Cavacini
Robert Cavagnaro
Jerome Cavaille
Vincent Cavaillès
Jean-Marc Cavaillon
Charles Cavalcante
Jeferson Cavalcante
João Henrique Cavalcanti
Etienne Cavalier
Matteo Cavaliere
Jennifer Cavallari
Francesca Cavallaro
Federica Cavallo
Rossana Cavallo
Pauline Cavanagh
Andrea Cavanna
Alessandro Cavarape
Andrea Cavazzoni
Emanuel Cavazzoni
Claudio Cavazzuti
David Cave
Kyle Cave
Lawrence Cavedon
Matthew Cavender
Lindsay Caverly
Jen Cavet
Marta Cavo
Francesco Cavrini
Kara Cavuoto
William Cawthorn
Atilla Cayir
Alfonso Cayota
Hugo Cayuela
Luis Cayuela
Remy Cazabet
Danielle Cazabon
Yves Cazals
Marine Cazenave
Daniele Cazzato
Valentina Cazzato
Dario Cazzola
Dario Cazzoli
Michele Cea
Ausiàs Cebolla
Jose Cecatti
Pierre-Emmanuel Ceccaldi
Mauro Ceccanti
Giancarlo Ceccarelli
Michele Ceccarelli
Vania Ceccato
Alessio Cecchinato
Marco Cecchini
Franjo Cecelja
Jacopo Cecere
Chao Cecilia
Ari Cedars
Tomas Cedhagen
Leticia Cedillo-Barron
Christine Cedraschi
Wendy Cegielski
Jose Cegoñino
Lasse Cehofski
Mirnova Ceide
Petr Cejka
Marta Ceko
Julide Celebi
Peter Celec
Alessia Celeghin
Ferhat Celep
Benedetto Celesia
Angela Celetti
Alessandro Celi
Ahmet Celik
E. Celinska
Mark Celio
Adam Celiz
Eleonora Cella
Sebastien Celle
Alessandro Cellerino
Jean Celli
Jonathan Celli
Sonia Cellot
Fabio Celotti

Alessandro Cembran
Osman Cen
Nicolas Cenac
Massimo Cenciarini
Chiara Cencioni
Jan Cendelin
Fernando Cendes
Mari Carmen Cenit
Edina Cenko
Danilo Centeno
Diego Centonze
David Centurion
Pasquale Cepparulo
Regina Cer
Roberto Ceravolo
Nuno Cerca
Jack Cerchiara
Leandro Cerchietti
Jorge Cerda
Artemi Cerdà
Joan Cerdà
Pablo Cerdán
Silvia Cereghini
Lage Cerenius
Giovanni Luca Ceresoli
Ruth Cerezo-Mota
Manuela Cerina
Ibolja Cernak
Valeria Cernaro
Tomislav Cernava
Luca Cerniglia
Cheryl Cero
Marta Ceroni
Ivania Ceron-Souza
Karen Cerosaletti
Marconi Cerqueira
Alejandro Cerrada
Robert Cerrato
Matteo Cerri
Gabriela Certad
Milan Certík
Simona Cerulli
Luigi Cerulo
Emilio Cervantes
Isabel Cervantes
J.L. Cervantes
Estrella Cervantes-García
Tiziana Cervelli
László Cervenak
Simon Cervenka
Daniela Cesana
Carina Cesar
Karolina César
Paolo Cesare
Flaminia Cesare Marincola
Mario Cesaretti
Paola Cesari
Ethel Cesarman
Stefano Cesco
Petr Cesla
Carlos L. Cespedes
Daniela Cesselli
Salih Cesur
Filomena Cetani
Justin Cetas
Zeynep Cetecioglu
Merih Cetinkaya
Dragana Cetojevic - Simin
Valeria Cetorelli
Deanna Cettomai
Eva Ceulemans
Miguel Angel Cevallos
Lucia Cevidanes
Ratnasekhar Ch
Chang-Jun Cha
Hoon-Suk Cha
Hyuk-Jin Cha
Jung-Yul Cha
Sang-Ho Cha
Sang-wook Cha
Seunghee Cha
Yong Sung Cha
Hala Chaaban
Linda Chaabane
Sarah Chabal
Anne-Lise Chaber
Caroline Chaboo
Hughes Chabriat
Eric Chabriere
George Chacko
Natacha Chacoff
Pablo Chacon
Carlos Chacon-Diaz
Marucia Chacur
Amar Chadaga
Richard Chadd
George Chadderdon
Alejandro Chade
Dave Chadee
Iadine Chades
Ryan Chadha
Raymond Chadwick

Benjamin Chaffee
Samuel Chaffron
Tyler Chafin
Marcos Chagas
Avry Chagnac
Frédéric Chagué
Catherine Chague-Goff
Jaskarndip Chahal
Ann Chahroudi
Richard Chahwan
Chenglin Chai
Guohong Chai
Liraz Chai
Wei-Ming Chai
Yimin Chai
Kaisorn Chaichana
Thanapong Chaichana
Naesinee Chaiear
J. Richard Chaillet
Anaïs Chailleux
Benny Chain
Maria Chait
Vijender Chaitankar
Raphaelle Chaix
Nayden Chakarov
Jeremiah Chakaya
Donald Chakeres
Joe Chakkalakal
Santhosh Chakkaramakkil Verghese
Tapas Chakma
Abhijit Chakrabarti
Anirikh Chakrabarti
Bikas Chakrabarti
Gopal Chakrabarti
Jayprokas Chakrabarti
Manohar Chakrabarti
Oishee Chakrabarti
Ratna Chakrabarti
Rumela Chakrabarti
Subhabrata Chakrabarti
Subrata Chakrabarti
Shantanu Chakrabartty
Debasis Chakrabarty
Paramita Chakrabarty
Jui Chakraborti
Pradip Chakraborti
Sajal Chakraborti
Amit Chakraborty
Debojyoti Chakraborty
Nabarun Chakraborty
Nilanjan Chakraborty
Nirali Chakraborty
Sourav Chakraborty
Sunandan Chakraborty
Supratik Chakraborty
Tamalika Chakraborty
Venkatesan Chakrapani
Srinandan Chakravarthy
Rubel Chakravarty
Runu Chakravarty
Sujay Chakravarty
Dhruva Chakravorty
Subhajit Chakravorty
Debanjan Chakroborty
Lamis Chalak
David Chalcraft
Vladislava Chalei
Aaron Chalfin
Anna Chalfoun
Antonio Chalfun-Junior
Matthew Chalk
Victoria Chalker
Dimitra Chalkia
Spyridon Chalkiadakis
Anastasia Chalkidou
Anil Challa
Pratap Challa
Kishore Challagundla
Lavanya Challagundla
Kirsty Challen
Roberta Challener
Joseph Challenger
Gaelle Challet
John Challis
John H Challis
John Challiss
Gemma Chaloner
Norah Chalouhi
Heining Cham
Simon Chamaillé-Jammes
Arnaud Chambellan
Amanda Chamberlain
Brent Chamberlain
Catherine Chamberlain
Dan Chamberlain
Luke Chamberlain
Nancy Chamberlin
Christina D Chambers
Jeremy Chambers
Tamy Chambers
Valerian Chambon
Venkateswarlu Chamcha

Larry Chamley
Lourdes Chamorro
Susana Chamorro
Gema Chamorro-Moriana
Carolina Chamorro-Viña
Benoit Champagne
Cory Champagne
Donald Champagne
Eric Champagne
Julie Champion
Keith Champion
Alexandre Champroux
Adrienne Chan
Allen Chan
Andrew Chan
Benny Chan
Brian Chan
Chi Chan
Chi Ngai Chan
Chi Wai Chan
Chi-Ping Chan
Chiu-Shui Chan
Christian Chan
Chun Chan
Danny Chan
David Chan
Deva Chan
Elsie Chan
Farrah Chan
Gary Chan
Helen Chan
Henry Chan
Hing Chan
Hon Fai Chan
Hua-Chen Chan
Jacky Chan
Jasper Chan
Jeffrey C. Y. Chan
Jessie Chan
John Chan
Johnny Chan
Juliana Chan
Kei Hang Katie Chan
Kitty Chan
Ko Ling Chan
Kun-Ming Chan
Lai Chan
Martin Chan
Michael Chan
Ming-Cheng Chan
Rosa Chan
Sheng-Chieh Chan
Stanley Chan
Stephen Chan
Ting Chan
Ting-Fung Chan
Wai Sum Chan
Wallace Chan
Wen-Ching Chan
Weng Chan
Wing Chan
Wing Keung Chan
Gursharan Chana
Pranav Chanchani
Ramesh Chand
Sourabh Chand
Anindya Chanda
Hersh Chandarana
Douglas Chandler
Jesse Chandler
Michael Chandler
Paula Chandler-Laney
Sudhir Chandna
Tarani Chandola
Amrish Chandra
Divay Chandra
Goutam Chandra
Joya Chandra
Namas Chandra
Nastassya Chandra
Nimai Chandra
Partha Chandra
Vishal Chandra
Stanley Chandradoss
Shanmuganathan Chandrakasan
Venkatraman Chandra-Mouli
Preethi Chandran
Robert Chandran
Pratik Chandrani
Mathangi Chandrasekar
Bharath Chandrasekaran
Natarajan Chandrasekaran
Praveen Chandrasekharan
Sujith Chandy
Rufus Chaney
Andrew Chang
Anne Lyn Chang
Anne Lynn Chang
Chee-Jen Chang
Cheng Chang
Chia-Chu Chang
Chia-Chuan Chang

Chia-en Chang
Chih Chang
Chih-Chao Chang
Chih-Jen Chang
Chih-Wei Chang
Chin-Chyuan Chang
Ching-Fang Chang
Chi-Ru Chang
Chiung Chih Chang
Chuan-Fa Chang
Chun Chang
Chun-Yen Chang
Chun-Yuan Chang
Daejun Chang
Darby Tien-Hao Chang
Ding-Kao Chang
Dorita Chang
Elizabeth Chang
Eric Chang
Eugene Chang
Fang-Rong Chang
Fengshui Chang
Feng-Yee Chang
Franklin Chang
Hang Chang
Hao Chang
Hao-Xun Chang
Heng-Cheng Chang
Hsueh-Wei Chang
Huan-Cheng Chang
Huibin Chang
Hye Jin Chang
I-Shou Chang
Jae-Hoon Chang
Jang-Yang Chang
Je-Ken Chang
Jer-Ming Chang
Jiang Chang
Jinhong Chang
Jun Chang
Jung-Chen Chang
Jung-Su Chang
Jyh-Jong Chang
Kai-Chih Chang
Kai-Hsiung Chang
Kang-Ming Chang
Keith Chang
Ke-Vin Chang
King-Jen Chang
Kuan-Cheng Chang
Kwanghyeon Chang
Larry Chang
Lennon Yao-chung Chang
Liang Chang
Lili Chang
Lin Chang
Long-Sen Chang
Min Jung Chang
Ming-Che Chang
Ming-Ling Chang
Ming-Wei Chang
Nien-Tzu Chang
Pei-Ching Chang
Sandra Chang
Seon Hee Chang
Shan Chang
Sheng-Kai Chang
Sheng-Nan Chang
Shih-Chieh Chang
Shih-Ching Chang
Shih-Liang Chang
Shi-Min Chang
Shu-Sen Chang
Sui-Yuan Chang
Tammy Chang
Tara Chang
Theresa Chang
Ting-Tsung Chang
W.L. William Chang
Wei-Chiao Chang
Wei-Pin Chang
Wei-Tang Chang
Wen-Chi Chang
Wen-Hsin Chang
Wenjun Chang
Wen-Ruey Chang
Wen-Tsan Chang
Wing Chang
Woo-Suk Chang
Xiaohui Chang
Xing Chang
Yang-Chi Chang
Yao-Feng Chang
Yen-Pei Chang
Yih-Leong Chang
Ying-Jun Chang
Youngjae Chang
Yu-Kang Chang
Z.J. Chang
Zhijie Chang
Hahn Chang-Gyu
Harish Changotra

Du Changwen
Chia-Hao Chang-Yang
Rudragouda Channappanavar
Andrew Channon
Heather A. Channon
Jacqueline Channon Smith
Stephen Chanock
Mary Chan-Park
Penradee Chanpiwat
Hubert Chanson
Ekktet Chansue
Paul Chantler
Tracey Chantler
Narisara Chantratita
Pithi Chanvorachote
Nadia Chanzu
C. Thomas Chao
Chia-Ter Chao
Day-Yu Chao
Dennis Chao
Jie Chao
Jinquan Chao
Melody Chao
Ming-Wei Chao
Nelson Chao
Pen-Hsiu Chao
Shiaoman Chao
Shih-Hui Chao
Tsu-Yi Chao
Tzu-Hao Chao
Yee Chao
Zhi Chao
Aaron Chapla
William Chaplin
V. Chaplot
Colin Chapman
Dale Chapman
David Chapman
Elaine Chapman
Hanah A. Chapman
Hazel Chapman
Jeremy Chapman
Joanne Chapman
John Chapman
Robert W. Chapman
Sarah Chapman
William Chapman
Enesia Chaponda
Svetlana Chapoval
Christine Chappard
Catherine Chappell
John Chappell
M. Chappell
Maryse Chappin
François Chappuis
Paul Chapron
Jean-Louis Chapuis
Nathalie Chaput
Brahim Chaqour
Brian Chaqour
Bharat R. Char
Ruby Charak
Kelvin Charambira
Fadi Charchar
Declan Chard
Sorujsiri Chareonsudjai
A. Charidimou
Edwin Charlebois
Cyril Charles
Joanna Charles
Nicolas Charles
Roch-Philippe Charles
Sonia Charleston-Villalobos
Nicolas Charlet-Berguerand
Edith Charlier
Keyne Charlot
George Charlton
Neil Charman
Bruno Charpentier
Marie Charpentier
Gregory Charrier
Guillaume Charriere
Sybil Charrière
Gilles Charvin
Athanasios Chasalevris
Arlen Chase
Brian Chase
Christine Chase
P. Chase
Steve Chase
Carine Chassain
Benoit Chassaing
Sylvie Chastant-Maillard
Dennis Chasteen
Anne-Laure Chateigner-Boutin
Jean-Marc Chatel
Suman Chaterjee
Mark Chatfield
Arnaud Chatonnet
Katherine Chatten
Devavani Chatterjea
Amitava Chatterjee

Anushree Chatterjee
Bappaditya Chatterjee
Debashree Chatterjee
Jayanta Chatterjee
Jolly Chatterjee
Kunal Chatterjee
Mitali Chatterjee
Moniya Chatterjee
Saurabh Chatterjee
Saurav Chatterjee
Subroto Chatterjee
Sumantra Chatterjee
Susmita Chatterjee
Suvro Chatterjee
Victor Chatterjee
Somnath Chatterji
Udayan Chatterji
Robert Chatterton
Kiranam Chatti
Arnab Chattopadhyay
Debasis Chattopadhyay
Debprasad Chattopadhyay
Dipankar Chattopadhyay
Indranil Chattopadhyay
Krishnendu Chattopadhyay
Naibedya Chattopadhyay
Sharmila Chattopadhyay
Subrata Chattopadhyay
Nagendra Chaturvedi
Ratna Chaturvedi
Sarika Chaturvedi
Sonali Chaturvedi
Kyriazoula Chatzianagnostou
Lia Chatzidiakou
Antonios Chatzigeorgiou
Stylianos Chatzipanagiotou
Nikos Chatzisarantis
Kimon Chatzistamatiou
T. Chatzistathis
K Chatzistefanou
Maria Chatzou
Destiny F. Chau
Gar-Yang Chau
Lee-Young Chau
Gyaneshwer Chaubey
Abhishek Chaudhary
Kunal Chaudhary
Veer Chaudhary
Dhruva Chaudhry
Shehzad Chaudhry
Abhijit Chaudhuri
Jayanta Chaudhuri
Minu Chaudhuri
Shubho Chaudhuri
Koel Chaudhury
Kunal Chaudhury
Isabelle Chaudieu
Junaid Ahsenali Chaudry
Anuj Chauhan
Arun Chauhan
Narsigh Chauhan
Neeraj Chauhan
Nikhil Chauhan
Subhash Chauhan
Vinita Chauhan
Maximilien Chaumon
Akhilanand Chaurasia
Shyam Chaurasia
Patrick Chauvel
Sylvain Chauvette
Arnaud Chauviere
Marie-Pierre Chauzat
Sreenivas Chavali
Porntip Chavalitshewinkoon-Petmitr
Sangeeta Chavan
Leonardo Chavane
Neil Chavannes
Mira-Lynn Chavanon
Jorge Chavarro
Jean-Paul Chavas
Marina Chavchich
Jerome Chave
Matthieu Chavent
Priscila Chaverri
Alexandre Chaves
Fábio Chaves
Madalena Chaves
Rafael Chaves
Thais Chaves
Paula Chaves-da-Silva
Adolfo Chavez
Mario Chavez
Noel Chavez
Robert Chavez
Luis Chavez de Paz
Martin Chavez Hoffmeister
Jorge Chávez-Villalba
Ali Reza Chavshin
Suhash Chavva
Crystal Chaw
Louise Chawla
Bénédicte Chazaud

Robin Chazdon
Gregorio Chazenbalk
Walter Chazin
Li Che
Pulin Che
Phaik Yeong Cheah
Yoke-Kqueen Cheah
Anna Wai San Cheang
Bentley Cheatham
Carol Cheatham
Daniel-Robert Chebat
Mary Chebib
Yahia Chebloune
Antonio Checa
Katherine Checkland
Dave Checkley
Frederic Checler
Alex Chee
Hui-Yee Chee
Sukhinder Cheema
Maxim Cheeran
James Cheeseman
Michael Cheetham
Novel Chegou
E. Wassim Chehab
Nor Chejanovsky
Cosimo Chelazzi
Linda Chelico
Meenakshi Chellaiah
Ivo Chelo
Giulia Cheloni
Hiam Chemaitelly
Lucia Chemes
Jonathan Chemouny
Daniel Chemtob
Sylvain Chemtob
Abel Chemura
Peter Chemweno
Alicia Chen
An-min Chen
Anping Chen
Antonia Chen
Bang-Bin Chen
Bao Wei Chen
Baojun Chen
Ben-Kuen Chen
Bijun Chen
Bin Chen
Bing Chen
Bingdi Chen
Bingkun Chen
Bo Chen
Caiyan Chen
Casey Chen
Catherine Chen
Ceshi Chen
Chang-Han Chen
Changli Chen
Chaomei Chen
Charles Chen
Chen Chen
Cheng-Sheng Chen
Cheng-Yu Chen
Chen-Huan Chen
Chen-Tung Arthur Chen
Chen-Yun Chen
Chi Chen
Chian-Feng Chen
Chiao-Chi Chen
Chi-Chih Chen
Chien-Cheng Chen
Chien-Chou Chen
Chien-Hung Chen
Chien-Tzung Chen
Chih Chung Chen
Chih-Chieh Chen
Chih-Jung Chen
Chih-Yen Chen
Ching Chen
Ching-Hsein Chen
Ching-Shih Chen
Chiung-Mei Chen
Chiu-Ying Chen
Chi-Wen Chen
Chong Chen
Chuanfa Chen
Chuan-Yu Chen
Chu-Huang Chen
Chuming Chen
Chun Chen
Chung-Hwan Chen
Chung-Jen Chen
Chunhua Chen
Chun-Ku Chen
Chun-Li Chen
Chunyan Chen
Cong Chen
Dar-Ren Chen
David Chen
Deliang Chen
Delphine Chen
Der-Yuan Chen

Didi Chen
Ding Chen
Dongbao Chen
Duan-Bing Chen
Dun-Jin Chen
E.P. Chen
Ee Sin Chen
Elaine Chen
Eleanor Chen
Emily Chen
Eric Chen
Fadi Chen
Fang Chen
Fanglin Chen
Fang-Ming Chen
Fanguo Chen
Fei Chen
Feng Chen
Feng-Chi Chen
Feng-Jui Chen
Fu-Chen Chen
Gang Chen
Gaojian Chen
Geng Chen
Guang Chen
Guimin Chen
Guo Chen
Guoxun Chen
Haifen Chen
Haitao Chen
Han Chen
Hanbo Chen
Han-Yang Chen
Haobin Chen
Haoyu Chen
Harn-Shen Chen
Hexin Chen
Holly Chen
Hong Chen
Hongda Chen
Honglei Chen
Hongsong Chen
Hongtong Chen
Hongwei Chen
Hong-Ying Chen
Hsiang Ling Chen
Hsiang-Yin Chen
Hsin-Hua Chen
Hsuan-Yu Chen
Hua Chen
Huan Chen
Huey-Ling Chen
Hui Chen
Huichao Chen
Hui-Ya Chen
Hung Chun Chen
Hung-Lin Chen
Hungwen Chen
J. Chen
Jang-Yi Chen
Jason Chen
Jauer Chen
Jeannie Chen
Jen-Jee Chen
Jeon-Hor Chen
Jiageng Chen
Jia-Hong Chen
Jiahua Chen
Jiangang Chen
Jiangping Chen
Jiang-Xing Chen
Jianming Chen
Jiann-Chu Chen
Jianquan Chen
Jianshe Chen
Jian-ye Chen
Jie Chen
Jin Chen
Jingchang Chen
Jin-Hua Chen
Jin-Ming Chen
Jinn-Yang Chen
Jiqiu Chen
Jiun-Han Chen
Jiunn-Horng Chen
Jiun-Rong Chen
Jiwang Chen
Jixin Chen
Joseph Chen
Joyce Chen
Juliette Chen
Jun Chen
Junhong Chen
Junjie Chen
Junping Chen
Jyh-Yih Chen
Kangming Chen
Ken Chen
Ke-Neng Chen
Keping Chen
Ke-Yang Chen
Kong Chen

Kow-Tong Chen
Kuanchin Chen
Kuang-Chi Chen
Kuan-Hui Chen
Kuan-Lin Chen
Kun Chen
Kunsong Chen
Kuo-Hu Chen
Lanying Chen
Lei Chen
Lei-Shih Chen
Leslie Chen
Li Chen
Li Jia Chen
Li Min Chen
Liang Chen
Liangbi Chen
Liang-Kung Chen
Liangyi Chen
Lien-Cheng Chen
Lih Chen
Lijing Chen
Lijun Chen
Li-Jun Chen
Limei Chen
Limin Chen
Lin Chen
Lina Chen
Ling Chen
Ling-Wei Chen
Lingyi Chen
Li-Sheng Chen
Li-Song Chen
Lizhang Chen
Long Chen
Longwen Chen
Lu Chen
Luzhen Chen
Mark A Chen
Mei-Huei Chen
Mei-Jou Chen
Meng Cheng Chen
Miao-Der (Sophie) Chen
Mien-Cheng Chen
Min Chen
Ming Chen
Minglong Chen
Mingming Chen
Ming-Shun Chen
Mingzhou Chen
Mo Chen
Moon Chen
Mu Chen
N Chen
Nan-Kuei Chen
Pai-Lien Chen
Pao-Yang Chen
Peii Chen
Peiwen Chen
Peng Chen
Peter Chen
Ping Chen
Ping-Ho Chen
Ping-Jen Chen
PingKun Chen
Po Chuan Chen
Po-Chun Chen
Qi Chen
Qian Chen
Qiang Chen
Qijun Chen
Qingfeng Chen
Qingsong Chen
Qingxia Chen
Qingyong Chen
Quan Chen
Qun Chen
Rachel Chen
Renjie Chen
Robert Chen
Roger Chen
Rong Chen
Rong-Jane Chen
Rongsheng Chen
Rou-Shayn Chen
Ru Chen
Rui Chen
Ruizhen Chen
Ruoling Chen
Sanmei Chen
Shangwu Chen
Shanyuan Chen
Shanze Chen
Shaojie Chen
Shaoqing Chen
Sharon Chen
Shih-Ann Chen
Shih-Chu Chen
Shih-Hsin Chen
Shih-Pin Chen
Shinn-Cherng Chen
Shi-Yao Chen

Shuen-Ei Chen
Shun-Hua Chen
Shuo Chen
Si Chen
Siqi Chen
Sixue Chen
Songbiao Chen
Ta-Fu Chen
Taiping Chen
Ta-Liang Chen
Tao Chen
Taoyong Chen
Tenghui Chen
Tian Chen
Tien-Hsing Chen
Trevor Chung-Ching Chen
Tsung-Ming Chen
Tzurei Chen
Vincent Chin-Hung Chen
Wan Chen
Wan Tao Chen
Wanping Chen
Wan-Qing Chen
Wei Chen
Weiguo Chen
Weihua Chen
Wei-June Chen
Wei-Jung Chen
Weina Chen
Wei-Sheng Chen
Weitao Chen
Weitian Chen
Wei-Ting Chen
Weiwei Chen
Weiyi Chen
Weizao Chen
Wen Chen
Wenbiao Chen
Wenbin Chen
Wen-Chi Chen
Weng-Pin Chen
Wenhao Chen
Wen-Hui Chen
Wenjia Chen
Wen-Jone Chen
Wen-Pin Chen
Wu Chen
Xi Chen
Xiangdong Chen
Xianhong Chen
Xiaochun Chen
Xiaofeng Chen
Xiaohong Chen
Xiaojie Chen
Xiaoming Chen
Xiao-Ping Chen
Xiao-Ren Chen
Xiaosong Chen
Xin Chen
Xin-De Chen
Xing Chen
Xinhua Chen
Xinjun Chen
Xiqun Chen
Xuan Chen
Xucai Chen
Xue Chen
Xuehao Chen
Xuesen Chen
Xuewei Chen
Yabin Chen
Yajun Chen
Ya-Lei Chen
Yan Chen
Yan Qiu Chen
Yang Chen
Yang-Kun Chen
YangQuan Chen
Yanyan Chen
Yanyu Chen
Yao-Hui Chen
Yao-Mei Chen
Yaoqing Chen
Yaw-Chung Chen
Ya-Wen Chen
Y-Chuang Chen
Ye Chen
Ye-Guang Chen
Yen-Chou Chen
Yen-Fu Chen
Yen-Hao Chen
Yen-Hsu Chen
Yen-Ta Chen
Yeu-Chin Chen
Yi Chen
Yi-Chen Chen
Yi-Cheng Chen
Yi-Chun Chen
Yi-Guang Chen
Yi-Hsin Chen
Yi-Hsing Chen
Yi-Ju Chen

Yijun Chen
Yi-Min Chen
Yin Chen
Ying Chen
Ying-Chou Chen
Ying-Chu Chen
Ying-Ping Chen
Yingxi Chen
Yingyao Chen
Ying-Yeh Chen
Yingying Chen
Yi-Tien Chen
Yixin Chen
Yong Chen
Young-Mao Chen
Yousheng Chen
Yu Chen
Yuanyuan Chen
Yu-Ching Chen
Yu-Chun Chen
Yuhchyau Chen
Yuh-Lien Chen
Yuli Chen
Yulin Chen
Yuming Chen
Yun Chen
Yunching Chen
Yung Chen
Yung-Chang Chen
Yung-Che Chen
Yung-Tai Chen
Yuning Chen
Yunji Chen
Yunshun Chen
Yuping Chen
Yuqing Chen
Yushu Chen
Zaozao Chen
Zeyu Chen
Zhansheng Chen
Zhe Chen
Zhen Chen
Zheng Chen
Zhengming Chen
Zhenguang Chen
Zhenju Chen
Zhenyu Chen
Zhenyue Chen
Zhe-Sheng Chen
Zhi Xiong Chen
Zhibin Chen
Zhichang Chen
Zhilei Chen
Zhimin Chen
Zhiqiang Chen
Zhong Chen
Zhuo Chen
Zigui Chen
Ziheng Chen
Zijiang Chen
Zi-Jiang Chen
Hossein Mahmoudi Chenari
Ezhilarasi Chendamarai
Jorn Cheney
Marshall Cheney
Alan Cheng
Allen Cheng
Bi-Hua Cheng
Bin Cheng
Bingbing Cheng
Chee Leong Cheng
Cheng-I Cheng
Chin-Yi Cheng
Dominic Cheng
Edward Cheng
Fang-Min Cheng
H. Cheng
Han Cheng
Hao Cheng
He Cheng
Hewei Cheng
Hong Cheng
I-Jiunn Cheng
Jia Cheng
Jianlin Cheng
Jie Cheng
Jie-Zhi Cheng
Jimin Cheng
Ji-Yen Cheng
Joseph Cheng
Jun Cheng
June Cheng
Kai-Chun Cheng
Karis Cheng
Ke Cheng
Kuan-Chen Cheng
Lesley Cheng
Li-Ming Cheng
LiTing Cheng
Long Cheng
Ming-Huei Cheng
Pin-Nan Cheng

Qiang Cheng
Qin Cheng
Ren-Chung Cheng
Robert Cheng
Rui Cheng
Sam Cheng
Shih-Ping Cheng
Shin-Ming Cheng
Shuiping Cheng
Shunfeng Cheng
Steven Cheng
Tianyin Cheng
Wei-Yi Cheng
Wen-Hsing Cheng
Xiang Cheng
Xingguo Cheng
Xin-Yue Cheng
Yang Cheng
Yao Cheng
Yi-Bang Cheng
Yi-Chuan Cheng
Yinhe Cheng
Yu Cheng
Yuan-Lung Cheng
Yuan-Yuan Cheng
Yuchung Cheng
Yu-Ting Cheng
Zhangrui Cheng
Zhongjian Cheng
Cecilia Cheng-Mayer
Vanessa Chen-Hussey
Jean-Francois Chenot
Alice Chen-Plotkin
Chantal Chenu
Anne Chenuil
Dong-Joo Cheon
Gi Jeong Cheon
In Su Cheon
Young Koog Cheon
Ai Theng Cheong
Jit Kong Cheong
Siew Ann Cheong
Soon Hon Cheong
Ying Cheong
Farah Maria Drumond Chequer
Kartikeya Cherabuddi
Bobby Cherayil
Razvan Chereji
Yves Cherel
Jacqueline Cherfils
Ameur Cherif
Vino Cheriyan
Linda Cherkassky
Michael Cherkiss
Yijuang Chern
Mariana Cherner
Natalie Chernets
Nikita Chernetsov
Debbie Cherney
Jerry Cherney
Howard Chernick
Leonid Chernin
Leonid Chernomordik
Boris Chernyak
Anatoly Chernyshev
P.V. Chernyshov
Alan Cherrington
Catherine Cherry
Elizabeth Cherry
Julia Cherry
M. Cherry
Beata Chertok
Aravind Cherukuri
Frank Chervenak
Inna Chervoneva
Naomi Chesler
Vera Chesnokova
Alessandro Chessa
Manuela Chessa
David Chester
Douglas Chesters
Khaled Chetehouna
Sanjay Chetia
Mahandranauth Chetram
Banthit Chetsawang
Basundhara Chettri
Verusia Chetty
Alice Cheung
Allen Cheung
Celeste Cheung
Chun Hei Antonio Cheung
Gordon Cheung
Jason Cheung
Karen Cheung
Kenneth Cheung
Martin Cheung
Olivia Cheung
Peter Cheung
Po-Yin Cheung
Rocky Cheung
Vien Cheung
Wing-Hoi Cheung

Yin Bun Cheung
Yu-Yan Chloe Cheung
Wisit Cheungpasitporn
Christian Chevalier
Coralie Chevalier
François Chevalier
Mathieu Chevalier
Astrid Chevance
James Cheverud
Boon How Chew
Cindy Chew
Teng-Leong Chew
Pragti Chhabra
S.K. Chhabra
Angela C. Chi
Chia-Yu Chi
Chih-Hsien Chi
Chih-Yu Chi
Congwu Chi
Donald Chi
Hongbo Chi
Iris Chi
Lang-Ming Chi
Oak Chi
Tai-Shih Chi
Wei Chi
Yuling Chi
Brian Chia
Jean-San Chia
John Chia
Kee Seng Chia
Mathias Ahii Chia
Nicholas Chia
Giulia Chiabotto
Fulvio Chiacchiera
F. Chiacchio
Ri-Cheng Chian
Alan Chiang
Bor-Luen Chiang
Chun-Ju Chiang
Chun-Pin Chiang
Hsiu-Yin Chiang
John Chiang
Jui-Kun Chiang
Kuo-Ping Chiang
Michael Chiang
Ming-Chang Chiang
Shian-Huey Chiang
Silvia Chiang
Tzu-Ching Chiang
Yao-Yi Chiang
Yu-Chih Chiang
Yu-Chung Chiang
Mariachiara Chiantore
Michela Chiappalone
Annalisa Chiappella
Ciro Chiappini
Mark Chiappone
Alberto Chiappori
Marilo Chiara
Enrico Antonio Chiaradia
Ferdinando Chiaradonna
Agostino Chiaravalloti
Lorenzo Chiariotti
Alessandro Chiarotto
Chikafumi Chiba
Hirofumi Chiba
Koji Chiba
Rie Chiba
Sanae Chiba
Tetsuhiro Chiba
Ravindra Chibbar
Adam Chicco
Adriana Chicco
R. Matthew Chico
Louis Chicoine
Natasha Chida
Eusebio Chiefari
Alaide Chieffo
Chih-Yen Chien
Chun-Wei Chien
Hua-Hong Chien
Jeremy Chien
Meng-Yueh Chien
Yi-Wen Chien
Arturo Chieregato
Jose Chies
Claudio Chiesa
Matheo Chiesa
Kimberly Chiew
Andreia Chignalia
An-Hsuan Chih
Takahiro Chihara
Violet Chihota
Jeremiah Chikovore
Stephanie Child
Michael Childress
Emma Childs
Mark Childs
Natalia Chilingirova
Jean-Marc Chillon
Palma Chillón

Antonio Chiloeches
Jennifer Chilton
Martin Chilvers
Natsayi Chimbindi
Isotta Chimenti
Maria Sole Chimenti
Patricia Chimin
Giovanna Chimini
Emile Rugamika Chimusa
Andrew Chin
Beth Chin
Kazuo Chin
Pei Yee Chin
Mauro Chinappi
V. Gregory Chinchar
Zaida Chinchilla-Rodriguez
Vernon Chinchilli
Alessandro Chinellato
Siew Mooi Ching
Arul Chinnaiyan
Sreedhar Chinnaswamy
Ashish Chintakuntlawar
Sumana Chintalapudi
Orawee Chinthakanan
Jonathon Chio
Adriano Chiò
Francesca Chiodi
Raymond Chiong
Giuseppe Chiossi
Chiuan-Chian Chiou
Hung-Yi Chiou
Jeng-Fong Chiou
Kathy Chiou
Lih-Chu Chiou
Pinwen Chiou
Pinwen Peter Chiou
Wen Liang Chiou
Wen-Liang Chiou
Enrico Chiovetto
Shubhada Chiplunkar
Lindsay Chipman
Matthias Chiquet
Johanne Chiquette
Abhilash Chiramel
Karri Chiranjeevi
Fernando Chirdo
Monique Chireau
Gherardo Chirici
Yolanda Chirino
Mayel Chirinos
Haruna Chiroma
Salvatore Chirumbolo
Carmelo Chisari
Athar Chishti
Meg Chisolm
Mohammod Chisti
Ayman Chit
Walter Chitarra
Tommaso Chiti
Teodor Chitlaru
Ajay Chitnis
Chetan Chitnis
Shilpa Chitnis
S. Chitra
Malinee Chittaganpitch
Mahati Chittem
Prashant Chittiboina
Lars Chittka
Jessica Chitty
Vladimir Chituc
Daniel Chitwood
David Chitwood
Calvin Chiu
Chih-Yu Chiu
Chong-Chi Chiu
Christine Chiu
Dickson Chiu
Hao Chiu
Helen Chiu
Hsien-Tsai Chiu
Hui-Wen Chiu
Jen-Hwey Chiu
Kuo Ping Chiu
Loren Chiu
Wei Che Chiu
Ya-Wen Chiu
Yen-Cheng Chiu
Yi-Wen Chiu
Yu-Chiao Chiu
Tzong-Shi Chiueh
Davide Chiumello
Valerio Chiurchiù
Cody Chiuzan
Peter Chivers
Linley Chiwona-Karltun
Lu Chi-Yu
Vyacheslav Chizhevsky
Ewa Chlebda
Katerina Chlichlia
George Chlipala
Anna Chmiel
Daniela Chmiest

QueeLim Ch'ng
Dong-Hyung Cho
DongYeon Cho
Eui-Sic Cho
Eunyoung Cho
Geum Joon Cho
Hansang Cho
Hye Sun Cho
Hyunsan Cho
Je-Yoel Cho
Kyoung Sang Cho
Kyoung-Oh Cho
Mi-La Cho
Nam Hoon Cho
Nam Jeong Cho
Sang-Heon Cho
Sohee Cho
Soo-Jin Cho
Ssang-Goo Cho
Sungbo Cho
Won Kyong Cho
Wookyung Cho
Yongcheol Cho
Yongku Cho
Yoon Hee Cho
Younghak Cho
Zhang-He Cho
Gabriel Chodick
Philip Chodrow
Daniel Choe
Jun-Yong Choe
Keith Choe
Regine Choe
Wonchae Choe
Yoonsuck Choe
Álvaro Choi
Bernard Choi
Chul Young Choi
Dongseok Choi
Edmond Choi
Eun-Kyoung Choi
Gi Hong Choi
Hong-Keun Choi
Hyo Geun Choi
Hyong Woo Choi
Hyung Jin Choi
Ik-Young Choi
Inchul Choi
In-Geol Choi
Jaebok Choi
Jae-Hoon Choi
Jaeyoung Choi
Jeong-Woo Choi
Ji Woong Choi
Jin Young Choi
Ji-Yeob Choi
Joon Young Choi
Joong Sub Choi
Joon-Il Choi
Kang-Yell Choi
Kelvin Choi
Ki Choon Choi
Kwang Choi
Kwang-Wook Choi
Mi-Kyeong Choi
Murim Choi
Seong-O Choi
Serah Choi
Seung Hong Choi
SuJean Choi
Wai-Man Choi
Woo Choi
Woonsup Choi
Y. Choi
Yongwon Choi
Yongwook Choi
Yoon-La Choi
Young Ki Choi
Youngshim Choi
Youn-Soo Choi
Yu Suk Choi
Yuri Choi
Manon Choinière
Kenneth S. H. Chok
Sylvie Chokron
Richard Chole
Jacek Cholewicki
Joana Cholin
Michael Choma
Bruno Chomel
Jacques Chomilier
Alain Yee-Loong Chong
James Chong
Ka Chun Chong
Luke Chong
Mary Foong-Fong Chong
Mian-Yoon Chong
Shu Zhen Chong
Yong Chong
Karen Chong-Seng
Virasakdi Chongsuvivatwong
Sarvenaz Choobdar

Mahesh Choolani
Lisa Chopin
Louise Chopinet
Lipsy Chopra
Luc-Henry Choquet
Emmanuel Chorianopoulos
Nikos Chorianopoulos
Joanna Chorostowska-Wynimko
Shoham Choshen-Hillel
Kesinee Chotivanich
Nafiisah Chotun
Alexander Chou
C.C. Chou
Cheng-Yang Chou
Cheng-Ying Chou
Che-Yi Chou
Chi-Chung Chou
ChihChin Chou
Joshua Chou
Jyh-Horng Chou
Kelvin Chou
Lien-Siang Chou
Li-Fang Chou
Michelle Chou
Ping-Yi Chou
Shih-Feng Chou
Tsui Chou
WenChi Chou
Ying-Hui Chou
Yi-Yu Chou
Bessem Chouaia
Christos Chouaid
Hichem Chouayekh
Chunaram Choudhary
Pratik Choudhary
Dipayan Choudhuri
Anindo Choudhury
Naseem Choudhury
Arun Chougule
Jyoti Chouhan
Jean-Yves Chouinard
Valerie Choumet
Jean Pierre Chouraqui
Evgenia Chourdaki
Mathieu Chouteau
C.K. Chow
Chi Chow
Dominic Chow
Edmond Chow
Eric Chow
Franklin Wang-Ngai Chow
Janette Chow
Jessica Chow
Keng-See Chow
Louis Chow
Louise Chow
Pierce Chow
Robert Chow
Seinen Chow
Wing Yee Chow
Winyoo Chowanadisai
Susmita Chowdhuri
Abhijit Chowdhury
Dipanjan Chowdhury
Muhammad Abdul Baker Chowdhury
Nityananda Chowdhury
Pritish Chowdhury
Samik Chowdhury
Sanjib Chowdhury
Sona Chowdhury
Soumitra Chowdhury
Tamjid Chowdhury
Gerardo Chowell
Diego Chowell-Puente
Gerardo Chowell-Puente
Itay Chowers
Michal Chowers
FongChan Choy
Wing-Yiu Choy
George Chressanthis
Jean-Paul Chretien
Sylvia Christakos
Anastasia Christakou
Wilhelm Christaller
Andreas Christe
Dinesh Christendat
Brian Christensen
Daniel Christensen
Erik Christensen
Henrik Christensen
Neil Christensen
Shawn Christensen
Terje Christensen
Thomas Decker Christensen
Tove Christensen
Cory Christenson
Dino Christenson
Lane Christenson
Henrik Christesen
Olivier Christiaens
James R. Christian
Mike Christian

Sherri Christian
Sven Christian
Julian Christians
Uwe Christians
Blaine Christiansen
Danny Christiansen
Julie Christianson
Katie Christie
Michael Christie
Brandenberger Christina
Paul Christine
Gregory Christman
Karen Christman
Markus Christmann
Romy Christmann
Peter Christmas
Myron Christodoulides
Joanna Christodoulou
Ana Christoff
G. Christoffersen
Mette Christoffersen
Astrid Christoffersen-Deb
Gustaf Christofferson
Gustavo Christofoletti
John Christoforidis
Devasahayam Christopher
Edward Christopher
Elliott Christopher
Michael Christopher
Katerina Christopoulos
Vasileios Christopoulos
Demetrios Christou
Christo Christov
Iva Christova
Annette Christy
John Christy
Veronika Chromikova
Zissis Chroneos
Angeliki Chroni
Elisabeth Chroni
George Chrousos
Maksymilian Chruszcz
Evangelia Chrysikou
Caitlin Chrystoja
Zofia Chrzanowska-Lightowlers
Darek Chrzeszczyk
Yacine Chtourou
C.H. Chu
Chia-Yu Chu
Chi-Ming Chu
Chun-Yen Chu
Dong Chu
Fang-I Chu
Fong-Fong Chu
Helen Chu
Hiutung Chu
James Chu
Liang Chu
Min Kyung Chu
Mingyuan Chu
Pu Chu
Simon Chu
Tien-Min Chu
Trang Chu
Wenying Chu
Xiang Chu
Xiang-Ping Chu
Xiaogang Chu
Yiu Wai Chu
Yiwei Chu
Zhaohui Chu
Zhaoqing Chu
Brendon Chua
Felix Chua
Jacqueline Chua
Kien Hui Chua
Melvin L. K. Chua
Melvin Lee Kiang Chua
Seng-Kee Chuah
Chiou-Fen Chuang
Hsiao-Chi Chuang
Kai-Hsiang Chuang
Linus Chuang
Pao-Tien Chuang
Po-Heng Chuang
Shao-Yuan Chuang
Shuang-En Chuang
Shu-Chun Chuang
Wen-Po Chuang
Wen-Yu Chuang
Yao-Chi Chuang
Yi-Fang Chuang
Yung-Jen Chuang
Jonathan Chubb
Victor Chubukov
Magda Chudzinska
Ana Marisa Chudzinski-Tavassi
María-Cristina Chueca
Jane Chueh
Shih-Chieh Chueh
Rashmi Chugh
Sumant Chugh

Abrar Chughtai
Bilal Chughtai
F.N. Chukwuneke
Narumol Chumuang
Chang-Zoon Chun
Chen Chun
Jongsik Chun
Kyung Ah Chun
Kyung-Hee Chun
Amanda Chunco
Alexandra Chung
Benjamin Chung
Bowen Chung
Brian Chung
Charles Chung
Chi-Jung Chung
Chuhan Chung
Hau Chung
Hee Jung Chung
Hsiao-Wen Chung
Ivy Chung
J. Sook Chung
Jay Chung
Jin Chung
Kian Chung
Kuo Fang Chung
Lorinda Chung
Min Gon Chung
Moon Jae Chung
Raymond Chung
Ren-Hua Chung
S.H. Chung
Seung-Kyu Chung
Sheng-Heng Chung
Sun Sook Chung
Tae Nyoung Chung
Taijoon Chung
Vicent Chung
Won-Yoon Chung
Woo-Hyun Chung
Yan Yi Chung
Yong Eun Chung
Warangkana Chunglok
Surasak Chunsrivirot
Cheng-Ming Chuong
Michael Chuong
Austin Church
George Church
Selina Church
Owen Churches
Morgan Churchill
Steven Churchill
Thomas Churchill
Jock Churchman
Tyler Churchward-Venne
Alexander Churkin
Kate Churruca
Daniella Chusyd
Susana Chuva de Sousa Lopes
Michael Chvanov
Lily Chylek
Deborah A Chyun
Marcello Ciaccio
Michal Ciach
Lynn Cialdella-Kam
Roberta Ciampichini
Claudia Cianci
Marcus Cianciaruso
Aurelio Ciancio
Bruno Ciancio
Mae Ciancio
George Cianciolo
Nicholas Cianciotto
Pasquale Ciarletta
Pasquapina Ciarmela
Aitor Ciarreta-Antuñano
Andrea Ciavattini
Edmund Cibas
Fabio Cibella
Merih Cibis
Alice Cibois
Josef Cibulka
Claudia Cicala
Cinzia Ciccacci
Francesco Ciccarese
Francesco Ciccia
Valentina Ciccolini
Marco Ciccone
Arrigo Cicero
Carla Cicero Studley
Giancarlo Cicolini
Flavia Cicuttini
Daniele Cicuzza
Maria Cid
John Cidlowski
Jan Cieciuch
Alarcos Cieza
Richard Cifelli
Michal Cifra
Ignacio Cifre
Secundino Cigarran Guldris
Larry Cihacek

Daniela Cihakova
Andrea Ciliberto
Gennaro Ciliberto
Gustavo Cilla
Michael Cima
Thomas Cimato
Rolando Cimaz
E.A. Cimbek
Antonio Cimellaro
Giulio Cimini
Cynthia Cimino
Megan Cimino
Sebastiano Cimino
Jeannie Cimiotti
Marco Amedeo Cimmino
Veronica Cimolin
Anca Maria Cimpean
Mehmet Ulas Cinar
Resat Cinar
Michael Cinelli
Antonella Cingolani
Gilda Cinnella
Olivier Cinquin
Michela Cinquini
Paolo Cintia
Corina Ciocan
Daniel Ciocca
Gianni Ciofani
Iacopo Cioffi
Jessica Cioffi
Maria Cristina Cioffi
William Cioffi
Claudio Ciofi
Marc Ciosi
Alexander Ciota
Marco Ciotti
Carlo Cipolli
Cristiana Cipriani
Ceren Ciraci
Andre Cire
Laura Cirelli
Vincenzo Ciriello
Daniela Cirillo
John Cirillo
Lisa Cirillo
Pasquale Cirillo
Plinio Cirillo
Paul Cirino
Maria Ciriolo
Connie Cirrincione
Ion Cirstea
Francesca Cirulli
Gerardo Cisneros
Irma Cisneros
Ousmane Cissé
Peter Cistulli
Toby Citrin
Bruce A. Citron
Antonio Cittadini
Carlo Citterio
Franco Citterio
Christine Citti
Michelle Ciucci
Philippe Ciuciu
Stefano Ciurli
Michal Ciurzynski
Simone Ciuti
John Civale
Laura Civiero
Roberto Civitelli
Agne Cižauskaite
Cassidy Claassen
Mareli Claassens
Kathleen Claes
Gerda Claeskens
Heiner Claessen
Frank Claessens
Lena Claesson-Welsh
Geert Claeys
Sylvie Claeysen
Frederik Claeyssens
Michael Clague
Jérôme Clain
Audrey Claing
Alexander Clanachan
Eoin Clancy
Thomas Clanton
Steven Clapcote
Pere Clapés
Hannah Clapham
Jennifer Clapp
Justin Clar
Mora Claramita
John Clare
Frédéric Clarençon
Marianne Clarholm
Roy Clariana
Andrew Claridge
Lee Claridge
Abbot Clark
Alex Clark
Alexander Clark

Anne Clark
Barbara A. Clark
C. Graham Clark
Chase Clark
Christopher Clark
Clancy Clark
Craig Clark
David Clark
Denise Clark
Jamie Clark
Jason Clark
John Clark
Melissa Clark
Melody Clark
Nathan Clark
Ruth Clark
Samuel Clark
Sarah Clark
Theodore Clark
Torin Clark
William Clark
Yvonne Clark
Alex Clarke
Anthea Clarke
Anthony Clarke
Cathy Clarke
Celia Clarke
Christopher Clarke
Damian Clarke
Duncan Clarke
Jennifer Clarke
Julia Clarke
Keith Clarke
Laura Clarke
Luka Clarke
Murray Clarke
Paul Clarke
Shannon Clarke
Shelley Clarke
Simon Clarke
Thomas Clarke
Toni-Kim Clarke
Jenny Clarkson
Joseph Classen
Menna Clatworthy
Erika Claud
Damiani Claudia
Mario Claudino
Pier Claudio
Ilene Claudius
Rolando Claure
Berry Claus
Heike Claus
Ashley Clausen
Malene Clausen
Thomas Clausen
Paola Clauser
Viola Clausnitzer
Kersten Clauss
Marcus Clauss
Sebastian Clauss
Daniel Clauw
Antonio Clavenna
Sabrina Clavijo-Baquet
Katrina Claw
Gary Clawson
Michael Clawson
Nicole Clay
Zanna Clay
John Claydon
Christine Clayton
David Clayton
J.D. Clayton
Anne Cleary
Margot Cleary
Michelle Cleary
Anna Clebone
Amanda Cleeve
Claire Cleland
John Cleland
Catherine Clelland
Mark Clemens
Stephan Clemens
Jan Clement
Nathan Clement
Wendy Clement
Sophie Clément
Christelle Clément-Duchêne
Diego Clemente
Filipe Clemente
R. Clemente
Sabrina Clemente
A. Clements
David Clements
Dennis Clements
Janice Clements
Kendall Clements
Mark Clements
Meredith Clements
Tristan Clemons
Damien Cleret
Aldo Clerico

Elise Clerkin
Anne-Marie Cleton-Jansen
Angela Cleveland
Beth Cleveland
James Cleverly
Geremy Cliff
Anna Cliffe
Gary Clifford
Martin Clift
Chuck Clifton
Christopher Cline
Mark Cline
James Cloern
Allanise Cloete
Gisele Clofent-Sanchez
James Clomburg
Alex Clop
Gerald Clore
M. Victoria Clos Guillen
Daniel Closa
Daniel Close
Svea Closser
Bonaventura Clotet
Brian Clough
James Clough
Steve Clough
Kate Clouse
Sean Clouston
Conrad Cloutier
Guy Cloutier
Jasmin Cloutier
Pascal Clouvel
Gavin Clowry
Eric Clua
Robin Clugston
William Clusin
Tim Clutton-Brock
Jeffrey Clymer
Sven Cnattingius
Marlon Coan
Nicolas Coant
Harvey Coates
Hasan Coban
O. Coban
Fernanda Cobayashi
Jason Cobb
Matthew Cobb
Melanie Cobb
Mia Cobb
Stephen Cobbold
Charles Cobbs
Fred Cobey
James Cobley
Erik Cobo
Ramon Cobo
Teresa Cobo
Jan Coburger
Bryan Coburn
Justine Coburn
Mark Coburn
Steven Coca
Massimo Cocchi
Eliana Coccia
Pierluigi Cocco
Federico Coccolini
Clement Cochain
Helena Cocheme
Jesse Cochran
Anne Cochrane
D.J. Cochrane
David Cochrane
J. Mark Cock
Andrew Cockburn
Simon Cockell
Gillian Cockerill
John Cockrem
Giuseppe Cocuzza
Giovanni Codacci-Pisanelli
Brian Codding
Claudia Codeço
Emily Coderre
Emily Cody
Vivian Cody
Chris Coe
Kathryn Coe
Eduardo Coelho
Flávio Coelho
Francisco Coelho
João Pedro Coelho
Lara Coelho
Luis Coelho
Tatiana Coelho-Sampaio
Yann Coello
Ruben Coen-Cagli
A. Coenders-Gerrits
Volker Coenen
Johann Coetzee
Lindi-Marie Coetzee
Martin Coetzee
Maureen Coetzee
Theresa Coetzer
Dan Cogalniceanu

Craig Cogger
David Coggon
Anna Coghill
Massimo Cogliati
Anthony Cognato
Mary Cogswell
Lauren Cohee
Alan Cohen
Andrew Cohen
Dov Cohen
Ehud Cohen
Eli Cohen
Elisabeth Cohen
Emily Cohen
Fredric Cohen
Jérémie Cohen
Jonathan Cohen
Justus Cohen
K. Bretonnel Cohen
Karen Cohen
Lisa Cohen
Marc Cohen
Mauricio Cohen
Michael Cohen
Mickaël M Cohen
Mitchell Cohen
Oded Cohen
Philip Cohen
Raymond Cohen
Robert Cohen
Ronald Cohen
Roy Cohen
Scott Cohen
Stuart H. Cohen
Susan Cohen
Yigal Cohen
Martin Cohen-Gonsaud
Or Cohen-Inbar
Devora Cohen-Karni
Alain Cohen-Solal
Karine Cohen-Solal
Wendie Cohick
Jay Cohn
Jeffrey Cohn
Lee Cohnstaedt
Anna Cohuet
Laurence Coiffard
Roney Coimbra
Susana Coimbra
Frederic Coin
Jean-Philippe Cointet
Ana Coito
Monica Cojocaru
Darren Coker
R. Coker
Vladan Cokic
Sophia Colaceci
Manuela Colafigli
Ben Colagiuri
Stefano Colagrande
Ertugrul Colak
Paolo Colantonio
Antonio Colantuoni
Elizabeth Colantuoni
Annamaria Colao
Annarita Colasante
Sheri Colberg-Ochs
Tim Colbourn
Fred Colbourne
Robb Colbrunn
David A. Colby
Alessandra Colciago
Ian Colditz
Alison Cole
Jacqueline Cole
Jeff Cole
John Cole
Kelly Cole
Lisa Cole
Michael Cole
Simon Cole
Tim Cole
Pasqualina Colella
Raymond Colello
Andrew Coleman
Brenda Coleman
Craig Coleman
David Coleman
Jamie Coleman
Jason Coleman
Melinda Coleman
Michael Coleman
Nicholas Coleman
Richard Coleman
Rosalind Coleman
Stephen Coleman
Mark Coles
Matthew Coleshill
Ricardo Coletta
Dario Coletti
Ross Colgate
Elena Colicino

Alain Colige
Sean Colin
Enrico Sandro Colizzi
Vittorio Colizzi
Mar Coll
Maria Carmen Collado
Olivier Collange
Jean-Francois Collard
Martine Collart
Licio Collavin
Andres Collazo
Jérôme Collemare
Daniel Collerton
Claude Collet
Jean-Paul Collet
Tinh-Hai Collet
Rosane Collevatti
Guylaine Collewet
Helen Colley
Agostino Colli
Leandro Colli
Catherine Collier
Charles Collier
David Collier
Glen Collier
Jason Collier
Joel Collier
Lara Collier
Nigel Collier
Rosemary Collier
Scott Collier
Russell Collighan
Olivier Collignon
Peter Collignon
Rachel Collin
Joanna Collingwood
Federica Collino
Andrew Collins
Brendan Collins
Brian Collins
Catherine Collins
Courtney G. Collins
Deirdre Collins
Douglas Collins
Eric Collins
Hal Collins
James Collins
Jeannie Collins
Jeremy Collins
Michael Collins
Natalie Collins
Richard Collins
Ronan Collins
Sara Collins
Paul Collinson
Adam Collison
Luana Colloca
Martine Collumbien
Michael Collyer
José Colmenero
Luiz Colnago
Céline Colnot
D.S. Colombari
Raffaella Colombatti
Davide Colombi
Barbara Colombo
Matteo Colombo
Mª Angels Colomer
Elisabeth Colon
Checo Colon-Gaud
Bianca Colonna
Vincenza Colonna
Jan Colpaert
Che Colpitts
Tonya Colpitts
David Colquhoun
Brett A. Colson
Natalie Colson
Max Coltheart
Veronika Coltheart
Juliana Colucci
Antonio Coluccia
Amedeo Columbano
Rosana Colussi
Charlotte Colvin
Emily Colvin
Lorenza Colzato
Maurizio Comanducci
Manola Comar
Iñaki Comas
Valérie Combaret
Yves Combarnous
Laurent Combettes
Lina Combita Merchan
Fanny Comblain
Etienne Côme
Aaron Comeault
Elena Comelli
Diego Comerci
Josep Comeron
Michael Comes
Alison Comfort

Paul Comfort
Eleonora Cominelli
Pierre Comizzoli
Giorgia Committeri
Concetta Compagno
Valérie Compan
Laurie Comstock
Gavin Conant
Katherine Conant
Barbara Conboy
John Conboy
Eva Conceiçao
Teresa Conceição
Luis Concepcion
Waldo Concepcion
Angel Concheiro
Jean-Paul Concordet
João Conde
Mario Vinicius Condini
Gerolama Condorelli
Gretchen Condran
Ana Conesa
Anthony Confer
Anthony Confera
Laura Conforti
Feng Cong
Fengyu Cong
Lin Cong
Rui Cong
Yingzi Cong
Emilio Congregado
Alice Conigliaro
Annalijn Conklin
Daniel Conklin
Andrew Conlan
Dalton Conley
Terri Conley
Erin Conlisk
Noel Conlisk
Michael Conlon
Thomas Conlon
Cath Conn
David Conn
Jan Conn
Kristen Conn
Ian R. Connell
Paul Connell
Terry Connell
John Connelly
Kim Connelly
Roxanne Connelly
David Conner
Remsberg Connie M
Brianne Connizzo
Stuart Connolley
Anne Connolly
Cara Connolly
Chris Connolly
Kristie Connolly
Thomas Connolly
Avonne Connor
James Connor
Kristin Connor
James Conolly
Cheryl Conover
Amy Conrad
Claudius Conrad
Clinton Conrad
Donald Conrad
Joseph Conrad
Kirk Conrad
Udo Conrad
Henk Jan Conradi
Mark S. Conradi
Timo Conradi
Georg Conrads
Barbara Conradt
Nicola Conran
Amy Conroy
Ronan Conroy
Robert Considine
Domenico Consoli
Alicia Consolini
Massimiliano Conson
Roberto Consonni
Christos Consoulas
Jonathan Constance
Stefan Constantinescu
Naama Constantini
Annamaria Conte
Giuseppe Conte
Michael Conte
Fabiano Jares Contesini
Alfredo Conti
Fabrizio Conti
Heather Conti
Mauro Conti
Michele Conti
Francisco Contijoch
M. Contin
Alessandro Contini
Gina Conti-Ramsden

Carmen Contreras
Cristina Contreras
Oren Contreras
Ryan Contreras
Sergio Contreras
Rubén Contreras Patiño
Bruno Contreras-Moreira
Jose Clemente Contreras-Naranjo
Atilano Contreras-Ramos
S.M. Contreras-Ramos
Oren Contreras-Rodríguez
Jose Contreras-Vidal
Alexander Converse
Paul Converse
Matteo Convertino
Baqiyyah Conway
Bernard Conway
Christopher Conway
Damian Conway
F. Conway
Kevin Conway
Michael Conway
Patricia Conway
Paul Conway
Richard Conway
Klaus Conzelmann
Alex Cook
Benjamin Cook
Bryan Cook
Chelsea Cook
Edwin Cook
Erik Cook
Gary Cook
Ian Cook
Jackie Cook
James Cook
Jane K. A. Cook
Jennifer Cook
John Cook
Joseph Cook
Linda Cook
Lyn Cook
Mark Cook
Michael Cook
Richard Cook
Robert Cook
Steven Cook
David Cooke
Dylan Cooke
Graham Cooke
Jessica Cooke Bailey
Susan Cook-Patton
R. Graham Cooks
Adrian Cookson
Sarah Jane Cookson
Carol Coole
Marco Coolen
Clarissa Cooley
Joseph Coolon
Brooke Coombes
Jason Coombes
Kevin Coombes
Stephen Coombes
Jeff Coombs
Kevin Coombs
Matthew Coombs
Robert Coombs
Sheryl Coombs
Brian Coon
Chelsea Cooper
Christine Cooper
Crystal Cooper
Crystale Cooper
Daniel Cooper
Dermot Cooper
Di Cooper
Gregory Cooper
Jon Cooper
Lauren Cooper
Lee Cooper
Matthew Cooper
Melanie Cooper
Nigel Cooper
Paul Cooper
Robin Cooper
Steve Cooper
Steven Cooper
Sue Cooper
Thomas Cooper
Trevor Cooper
Craig Coopersmith
Jeremy Cooperstock
Christine Cooper-Vince
Catelijne Coopmans
Agata Copani
Davis Cope
David Copeland
Jennifer Copeland
John Copeland
Laurel Copeland
Robert Copeland
William Copeland

Massimiliano Copetti
John Copland
Richard Copley
Andrea Coppadoro
Fabio Coppedè
Michiel Coppens
Frauke Coppieters
Ken Coppieters
Helene Coppin
Mauricio Coppo
Gianluca Coppola
Mariangela Coppola
Jeremy Coquart
Jonathan Coquet
Davide Cora
Daniel Corach
Daniele Coraci
Emrah Coraman
Giuseppe Coratella
Luca Corazzini
Sitki Corbacioglu
Laure Corbari
Lynette Corbeil
Stefano Corbella
Jennifer Corbett
John Corbett
Mark Corbett
Filomena Corbo
Rosa Maria Corbo
Vincenzo Corbo
Jason Corburn
Vanessa Corby-Harris
Angela Corcelli
Juan Corchado
Brandan Corcoran
Jacob Corcoran
Katja Corcoran
Tim Corcoran
Jacques Corcos
Gennaro Cordasco
Anna Cordeiro
Juliana Cordeiro
Susan Cordell
Eugenia Cordelli
Guilherme Cordenonsi da Fonseca
Gregory Corder
Paul Cordero
Ricardo Cordero-Otero
Adolfo Cordero-Rivera
R.L. Cordioli
Ricardo Cordioli
Jose Cordoba-Chacon
Giada Cordoni
Catherine Cordonnier
Luis Cordova
Juan Cordovez
David Corey
Eva Corey
Jonathan Corey
Anthony Corfield
Carlos Coriano
H. Corke
Harold Corke
Alexandru Corlan
Luca Corlatti
Richard Corlett
Victor Corman
Xochitl Cormon
Mireille Cormont
Jennifer Cornacchione
Alyssa Cornall
Markus Cornberg
Divi Cornec
Emilie Cornec-Le Gall
Amy Corneli
Sylvie Cornelie
Elise Cornelis
Pierre Cornelis
Dawn Cornelison
Gert Cornelissen
Piers Cornelissen
Jan Cornelius
Llewellyn Cornelius
Sean Cornelius
Morna Cornell
Robert Cornell
Laura Cornelsen
Claus Cornett
Martin Cornillet
Robert Cornman
Christopher Cornwall
Jeff Cornwell
William Cornwell
Benjamin Corona
Giovanni Corona
Ligiana Corona
Miguel Corona
Jorge Coronado Daza
Minas Coroneo
Sinead Corr
Domenico Corradi
Nicola Corradi

Maria Letizia Corradini
Chiara Corrado
Alberto Corral
Ricardo Corral
Montserrat Corral Varela
Marisol Corral-Debrinski
Genevieve Corraze
Sébastien Corre
Francisco Correa
Isabel Correa
L.A. Correa
Margarita Correa
Regis Correa
Alberto Corrêa
Clynton Corrêa
Vania Maria Correa da Costa
Goncalo Correia
Maria Correia
Marta Correia
Nuno Correia
Rion Correia
Sandra Correia
Sofia Correia
Stephen Correia
Teresa Correia
Paulo Correia-de-Sá
Margarida Correia-Neves
André Correia-Santos
Xavier Correig
Marialaura Corrente
Fenella Corrick
Daniele Corridoni
Frank Corrigan
Kathleen Corriveau
Sherryse Corrow
Cesare Corselli
Angelo Corsico
Gaetano Corso
Monica Corso Pereira
Simonetta Corsolini
Timothy Corson
Ronald Corstanje
Paul Corstjens
Aitziber Cortajarena
Sonia Cortassa
Rense Corten
Adrian Cortes
Jesus Cortes
Marina Côrtes
P.A. Cortesi
Naima Cortes-Perez
Anne Cortey
David Cortez
Blaise Corthesy
Blaise Corthésy
Paulo Corti
Stefania Corti
Mario Cortina-Borja
Monica Cortinovis
Bart Cortjens
Gino Cortopassi
Ronald Cortright
Luigi Corvaglia
Tereza Corvelo
Gerardo Corzo
Paul Cos
Andrea Cosacov
Maria Raquel Cosate
Michele Coscia
Mireia Coscolla
Sandro Cosconati
Stephen Cose
Bradley Cosentino
Carlo Cosentino
Jose Cosgaya
Daniel Cosgrove
David Cosgrove
Lisa Cosgrove
Costantino Cosimo
Devrim Coskun
Erich Cosmi
Lorenzo Cosmi
Andreea Cosoveanu
Djurdjica Coss
Stephanie Cossette
Davide Cossu
Angelo Costa
Blaise Costa
Catarina Costa
Cristiane A. Costa
Daniel Costa
Eleonora Costa
Elisiio Costa
Fabrizio Costa
Fernando Costa
Gabriel Costa
Ivan Costa
Joao Costa
Jose Costa
Judite Costa
Kevin Costa
Luis Costa

Madalena Costa
Manuela Costa
Marcelo Costa
Marcio Costa
Marcos Costa
Mário Costa
Marta Costa
Ozeas Costa
Roberto Costa
Rodolfo Costa
Rodrigo Costa
Rui Costa
Soraia Costa
Teresa Costa
Thiago Costa
Tommaso Costa
Valerio Costa
Vincent Costa
Luis Fernando Costa Alberto
Adriana Costa Bacelos
Gustavo Costa Fernandes
Adele Costabile
Sabine Costagliola
Gregory Costain
Deborah Costakos
Leticia Costa-Lotufo
Mauro Costa-Mattioli
Giulio Costantini
Maria Costantini
Susan Costantini
Todd Costantini
Gabriele Costantino
Robert Costanza
Francesco Costanzo
Paola Costanzo
Pierluigi Costanzo
Sandra Costanzo
Javier Costas
Rodrigo Costas Comesaña
João Henrique Costa-Silva
Lluís Costa-Tutusaus
Carmen Costea
Paola Costelli
Catherine Costello
E. Costello
Fiona Costello
John H. Costello
Sylvain Costes
Maria Costi
Emilia Costin
Xurxo Costoya
Michel Cot
Douglas Cotanche
Helene Cote
Jean-Francois Cote
Jocelyn Cote
Franco Cotelli
E. Cothran
Giovanni Coticchio
Eliecer Coto
Sebastian Cotofana
Antonella Cotoia
Michiel Cottaar
Vinicius Cotta-de-Almeida
Aoife Cotter
James Cotterell
Alberto Cottica
Yves Cottin
Clémentine Cottineau
Jacques Cotting
Linda Cottler
John Cotton
Louise Cotton
Pascale Cotton
Robin Cotton
Grazia Cottone
Pietro Cottone
David Cottrell
Joan Cottrell
Steven Couch
Emmanuel Coudeyre
Yves Coudière
Damien Coudreuse
Evelyne Coudrier
Thomas Coughlin
Catherine Couillard
Carolyn Coulam
Olivier Coulon
Graeme Coulson
John Coulson
Peter Coulson
Sonali Coulter
Mark Coulthard
Timothy Counihan
Christopher Counter
David Couper
Jane Couperus
P. Courand
Albert Courey
Donald Court
Cari Courtenay-Quirk
Philippe Courtet

David Courtin
Emilie Courtin
Elizabeth Courtney-Long
Aurélien Courvoisier
Delphine Courvoisier
Roger Cousens
Anthony Cousien
Xavier Cousin
Aidan Cousins
Fiona Cousins
Scott Cousins
Anna Coussens
Paul Coussens
Marc Coutanche
Sheryl Coutermarsh-Ott
Julien Couthouis
Artur Coutinho
Felipe Coutinho
Francisco Coutinho
Renato Coutinho
Teresa Coutinho
Isabel Couto
Natacha Couto
Kasey Couts
Stephane Coutu
Larry Couture
Marc Couture
Cyril Couturier
Aude Couty
Margaret Couvillon
Manuel Covarrubias
Giovanny Covarrubias-Pazaran
Naima Covassin
Anabella Covazzi Harriague
Maureen Covelli
Emma F. Covelo
Dawn Coverley
Adrian Covic
Kyle Covington
Michael Covington
Yolanda Covington-Ward
Douglas Cowan
Lauren Cowan
Ronald Cowan
Zara-Louise Cowan
Charles Cowden
Richard Cowden
Rita Cowell
Philip Cowen
Christina Cowger
Chris Cowled
Daniel Cowley
Kristine Cowley
Randy Cowling
Wallace Cowling
Peter Cowman
Brian Cox
Charles Cox
Cymon J. Cox
David Cox
Eileen Cox
Georgina Cox
Helen Cox
Justin Cox
Laura Cox
Marc Cox
Michael Cox
Robert Cox
Roger Cox
Ruth Cox
Sharon Cox
Stephen Cox
Tarnya Cox
Timothy Cox
Harvey Coxson
Kim Cox-York
Pilar Coy
Beth Coyle
Brian Coyle
Christina Coyle
Douglas Coyle
Edward Coyle
Sarah Coyne
Wendy Cozen
Yvette Cozier
Bruno Cozzi
Luca Cozzi
Alessandro Cozzi-Lepri
Daniel Cozzolino
Salvatore Cozzolino
David Crabb
John Crabb
John Crabbe
Margaux Crabtree
Nicola Crabtree
Valerie Crabtree
William Craelius
David Craft
Ashley Craig
Elizabeth Craig
Hugh Craig
James Craig

Michael Craig
Paul Craig
Laila Craighero
Charles Craik
Fergus Craik
Noel Craine
Ann Cralidis
Angelique Cramer
Angélique Cramer
Christina Cramer
Holger Cramer
Karina Cramer
Christian Crandall
David Crandall
Ian Crandell
Kristen Crandell
Otis Crandell
Barbara Crane
Benjamin Crane
Melissa Crane
Nicole Crane
Julia Craner
Ross Cranston
Mirian Crapez
Audrey Cras
Karen Crasta
Louise Crathorne
Alfredo Cravador
Sandra Craveiro
Sérgio Nuno Craveiro Barra
Kelly Craven
Rolf Craven
Forrest Crawford
Joseph Crawford
Keith Crawford
Sean Crawford
Steve Crawford
Sybil Crawford
Jacob Crawshaw
Francesco Crea
Dudley Creagh
Trevor Creamer
Daniel Crean
Jenette Creaney
Susan Creary
Teresa Crease
Graham Creasey
Carmine Crecchio
Tyler Creech
Melanie Cree-Green
Sarah Creel
Esther Creemers
Laura Creemers
Sarah Creem-Regehr
Simon Creer
Bob Creese
John Creese
Judy Creighton
Lazaro Cremades
Amelieke Cremers
Anne-Claude Crémieux
Pascal Crenn
Elvira Crescenzi
Clara Crescioli
Emmanuele Crespan
Irene Crespo
Jose Crespo
José-Manuel Crespo
María Crespo
Marta Crespo
W. Cress
John Cressman
Ross Cressman
Maurizio Crestani
Daniele Cretella
Fabiiola Cretti
Carole Creuzenet
Frédéric Crevecoeur
Pedro Crevillén
Kristine Crews
Phillip Cribb
Matthew Cribbet
Neil Crickmore
Daniel Crimston
Lucio Crino
Alessandro Crippa
Richard Cripps
Ernesto Crisafulli
Michael Criscitiello
Joseph Crisco
Peter Crisp
Ioana Cristea
Mioara Cristea
Matthieu Cristelli
Bogdan Cristescu
Penny Cristinacce
Massimo Cristofaro
Elena Critselis
Leandro Crivellenti
Carlos Crivelli
Anna Croce
Jenifer Croce

Lory Crocè
Anne Crocker
Jonny Crocker
Amy Crockett
Leonard Crocombe
Julio Croda
Kevin Croft
Pascal Croiseau
Timothy Croley
Anselm Crombach
Anton Crombach
Lisa Cromer
Fuzz Crompton
Howard Cromwell
David Crone
Aidan Cronin
Christine Cronin
Katherine A. Cronin
Robert Cronin
Alice Cronin-Golomb
Mark Cronin-Golomb
Tatjana Crönlein
Darren Crook
Nigel Crook
Colin Crooks
Kevin Crooks
Regan Crooks
Benjamin Crookston
Wendell Cropper
Emma Crosbie
Martine Croset
Vincent Croset
Nicola Crosetto
Paolo Crosetto
Adrienne Crosier
Caroll Cross
Paul Cross
Robert Cross
Carla Crossman
Elena Crotti
Lia Crotti
Laura Crotty Alexander
Dustin Crouch
Nicholas Croucher
Ian Croudace
Scott Crouter
Christian Crouzet
Olivier Crouzet
Antonio Crovace
David Crowder
Larry Crowder
Andrew Crowe
David Crowe
John Crowe
James Crowe Jr.
Trevor Crowell
Brooke Crowley
Steven Crowley
Thomas Crowley
Cynthia Crowson
Ruth Croxford
Michele Crozatier
Sarah Crozier
Eric Crubézy
Aline Crucello
Tania Crucitti
Valeria Crudele
Margaret Cruickshank
Sheena Cruickshank
Nancy Crum-Cianflone
Byron Crump
Casey Crump
Carlos Crusciol
Anne-Marie Crutz-Le Coq
Maarten Cruyff
Adriano Cruz
Celia Cruz
Dianne Cruz
Fernanda Cruz
Ricardo Cruz
Tania Cruz
Vinicius Cruzat
Monica Cruz-Lemini
Edwin Cruz-Rivera
Sally Cryan
Gabor Csanyi
Edit Csapo
Robert Csapo
Arpad Csernetics
Gabor Cserni
Valér Csernus
Anita Cservenka
László Csiba
Gabor Csifcsak
J. Csiszár
Sándor Csosz
Irene Cuadrado
Miguel Cuadros
Marely Cuba-Díaz
Judit Cubedo
Francisco Javier Cubero
Francisco Cubillos

Susana Cubillos
Esther Cubo Delgado
Belen Cubria
Cankut Cubuk
Magali Cucchiarini
Andrea Cucina
Francis Cucinotta
Phillip Cuculich
Zulma Cucunubá
Will Cuddy
Gabriel Cuellar Partida
Mayra Cuellar-Cruz
Karen Cuenco
Guillermo Cuervo
Henar Cuervo
Maria Cuevas
Katarina Cufar
Andrew Cuff
Marc Cuggia
Chang-Yi Cui
Changzheng Cui
F. Cui
Fang Cui
Feng Cui
Guiyun Cui
Jin-Jie Cui
Jiujie Cui
Jixin Cui
Ju Cui
Julia Cui
Liwang Cui
Long Cui
Mei-Zhen Cui
Qinghua Cui
Taixing Cui
Wei Cui
Weiguo Cui
Wenguo Cui
Wen-Yan Cui
Xiaobing Cui
Xiao-Guang Cui
Xiaojiang Cui
Xiaoyong Cui
Xing Cui
Xuerong Cui
Yan Cui
Yuehua Cui
Yujun Cui
Yuwei Cui
Zheng Cui
Zhenling Cui
Ziyou Cui
Dajana Cuicchi
Remi Cuingnet
Fabijan Cukelj
Tolga Çukur
Heather Culbertson
Mustafa Culha
Susan Culican
Alexis Cullen
Jay Cullen
John Cullen
Kathleen Cullen
Paul Cullen
Fiona Culley
Christophe Cullin
John Culling
Ken Cullings
Maxime Culot
Valeria Culotta
Kennith Culp
Steven Culpepper
Nicolo Cultrera
Darius Culvenor
David C. Culver
Ana Cumano
Bob Cumming
David Cumming
Geoff Cumming
Oliver Cumming
Paul Cumming
Steve Cumming
Toby Cumming
Christopher Cummings
James Cummings
Joanne Cummings
Kevin Cummings
Michael Cummings
Vonda Cummings
Carolyn Cummins
Christopher Cummins
Eoin Cummins
James Cummins
Nathan Cummins
Scott Cummins
Andrew Cundy
David Cunefare
Ana Cunha
Carla Cunha
Cristina Cunha
Diogo Cunha
Eugénia Cunha

Tatiana Cunha
Joana Cunha-Cruz
Barbara Cuniberti
Toni Cunillera
Wang Cunlin
Anthony Cunningham
Christopher Cunningham
James Cunningham
Jeffrey Cunningham
Mark Cunningham
Mark O. Cunningham
Melissa Cunningham
Michael Cunningham
Rebecca Cunningham
Solveig Cunningham
Susan Cunningham
Timothy Cunningham
Aubrey Cunnington
Christina Cuomo
Ornella Cuomo
Rocío Cupeiro
Zeljko Cupic
Michele Curatolo
Christine Curcio
Giuseppe Curcurù
Gary Curhan
Sharon Curhan
Nelson Curi
Laura Curiel
Giuseppe Curigliano
Marco Curini-Galletti
Belinda Curley
James Curley
Aaron Curns
Maria Curotto de Lafaille
David Curran
E. A. Curran
Jerry Curran
Sara Curran
Matthew Currell
Cameron Currie
Craig Currie
Kim Currie
Frank Curriero
David Currow
Fitz-Roy Curry
Joseph Curry
Stephen Curry
Sue Curry
Kristina Curry Rogers
Marina Cursino
Deborah Cursino dos Santos
Abigail Curtis
Brian Curtis
Caitlin Curtis
Hilary Curtis
Jeffrey Curtis
Kathleen Curtis
Kelly Curtis
Tobey Curtis
Wayne Curtis
Jennifer Curtiss
Patrick Cusaac
Cody Cushing
Patrick Cushing
Melanie Cushion
Maria Grazia Cusi
Kathleen Cusick
Sarah Cusick
Natalie Cusimano
Christine Cuskley
Victoria Cussen
Innes Cuthill
Cristina Cutillas
Chris Cutler
Michael Cutler
Ronald Cutler
Christina Cutshaw
Gary Cutter
Angela Cuttitta
Carrie Cuttler
J.J. Cutuli
Susan Cu-Uvin
Daphne Cuvelier
Griet Cuyckens
Jack Cuzick
Lise Cuzin
Erin Cvejic
Fatima Cvrckova
Krystyna Cwiklinski
Miroslaw Cygler
Aaron Cypess
Katarzyna Cypryk
Sheila Cyril
Malgorzata Cytrynska
Colette Cywes-Bentley
Tomer Czaczkes
Laszlo Czako
Christian Czech
Karen Czeloth
Bogdan Czerniak
Rakefet Czerninski

Fabian Czerwinski
Istvan Czigler
Gábor Czirják
Andras Czirok
Mirjam Czjzek
Hanokh Czosnek
Henryk Czosnek
Marek Czosnyka
Michael Czubryt
Malgorzata Czyz
Veronica D’Amico
Andrea D’Ariano
Maurizio D’Esposito
Patrick C. D’Haese
Christophe D’Hulst
Beena Benita D’Souza
Xing Da
Ana Carolina da Costa
Bruno da Costa
André Luis da Costa da Silva
Ana Maria da Costa Ferreira
Maria Catarina Salvador da Motta
Andrea Da Poian
Joao da Rocha Fernandes
Sofia Manuela da Rocha Lopes
Renata da Rosa
Federico Da Settimo
A. Menezes da Silva
Cláudia da Silva
Denis da Silva
Edson da Silva
Gabriela Jorge da Silva
Luís P. da Silva
Moacyr Da Silva
Nancy Da Silva
Pedro da Silva
Ricardo Da Silva
Rodrigo da Silva
Sergio Da Silva
Shana da Silva
Ives da Silva Bueno
Ademar Da Silva Filho
Geraldo da Silva Jr.
Itabajara da Silva Vaz Jr.
Winnie Daamen
Malin Daase
Sarah Dababnah
Alain Dabdoub
Carole Dabney-Smith
Neha Dabral
Alessandra D'Abramo
Janusz Dabrowski
Matthieu Dacher
Berihun Dachew
Jordi Dachs
James Dachtler
Lorraine Dacre-Pool
Laura Dada
Ekaterina Dadachova
Swati Dadhich-Mandal
Michael Daehne
Stijn Daenekindt
Frank Daerden
Ted Daeschler
Kirk Daffner
Katherine Dafforn
Nishant Dafle
Amrita Daftary
Gaurang Daftary
Ron Dagan
Mohsen Daghooghi
Lina Dagnino
Gregorio D'Agostino
Paul D'Agostino
Albert Dahan
Harel Dahari
Nader Dahdaleh
Elizabeth Daher
Silvia Daher
Chitra Dahia
Audun Dahl
Christiane Dahl
Richard Dahl
Gunnar Dahlen
Hannah Dahlen
Craig Dahlgren
Maik Dahlhoff
Marie Dahlin
Frank Dahlke
Kimberly Dahlman
Annegret Dahlmann-Noor
Nils Dahlstrom
Souhayl Dahmani
Hans-Uwe Dahms
Tanya Dahms
Nabila Dahodwala
Chang-Feng Dai
Chia-Yen Dai
Chuanchao Dai
Dao-Qing Dai
Dongjuan Dai
Guohao Dai

Jihong Dai
Mu-Shui Dai
Pu Dai
Qi Dai
Rujuan Dai
Shifeng Dai
Silan Dai
Tianhong Dai
Wei Dai
Xiangyan Dai
Xi-Jian Dai
Yakang Dai
Yang Dai
Zhanwu Dai
Zhenwen Dai
Zhi-Cong Dai
Zhi-Jun Dai
Zhiyu Dai
Zhuang Dai
Andreas Daiber
Stephen Daiger
Remi Daigle
Makoto Daimon
Masao Daimon
Stephen Dain
Benjamin Dainat
Francesco D'Aiuto
Dina Dajani
Dawit Daka
Gavin Daker-White
Helen Dakin
Stephanie Dakin
Mohammed Dakna
Krishnamurti Dakshinamurti
Sarada Prasad Dakua
Francesco Dal Grande
Andrea Dal Pozzolo
Juliano Dal Pupo
Maxwell Dalaba
Dev Dalal
Ram Dalal
Rinkoo Dalan
Liliana D'Alba
Vincent Dalbo
Matthew Dalby
Fevzi Daldal
Håvard Dale
James Dale
Nicholas Dale
Pat Dale
William Dale
Jonas Dalege
Julio Daleprane
Fredrik Dalerum
Yuri D'Alessandra
Brian Daley
Ertugrul Dalgic
Mohammad Reza Daliri
Deniz Dalkara
Turgay Dalkara
Timothy Dall
Emanuela Dalla Costa
Riccardo Dalla Volta
Denize Dall'Agnol
Enrico Dall'Ara
Erica Dall'Armellina
George Dallas
Tad Dallas
Frederic Dalle
Stephane Dalle
Antonella Dalle Zotte
Jesmond Dalli
James Dalling
Geesje Dallinga-Thie
Reinhard Dallinger
John Dallon
Ronald Dalman
Mario Dalmaso
Marion Dalmasso
Claudia Dalmastri
David Dalmau
Christine Dalmazzone
Mehmet Dalmis
Cassidy D'Aloia
Marco D'Alonzo
Danilo Daloso
Maeli Dal-Pai-Silva
Jade d'Alpoim Guedes
Michele Dalponte
Daniel Dalquen
Rafael Dal-Ré
Sarah Dalrymple
Bruce Dalton
Elizabeth Dalton
Lori Dalton
Matthew Dalva
Pranjali Dalvi
Daniel Daly
Doug Daly
Ian Daly
Kevin Daly
Marymegan Daly

Roger Daly
Eswar Damaraju
Samir Damare
Mahendra Damarla
Antonio Damasio
Erika Damato
Francesca D'Amato
Michael Dambacher
Michael D'Ambrosio
Roberta D'Ambrosio
Pauliina Damdimopoulou
Gregory Dame
Remus Dame
Stefano D'Amelio
Gautam Damera
Sarah Damery
Dionne Dames
Christian Damgaard
Phong Dam-Hieu
Giovanna Damia
Bruce P. Damiano
Diane Damiano
Brian Damiata
Adele D'Amico
Gennaro D'Amico
Marcello D'Amico
John Damicone
Daniel Damineli
Daalkhaijav Damiran
Olaf Dammann
Jesper Dammeyer
Melanie Dammhahn
Chendil Damodaran
David D'Amore
Derek Damron
Fredrick Damron
Amooru G. Damu
Reza Dana
Syamal Dana
Goodarz Danaei
Cédric Dananché
David Dance
Eline Dancet
Valentin Danchev
Etienne Danchin
Nicolas Danchin
Andrew Dancis
Abhijit Dandapat
Orwa Dandash
Abhaya Dandekar
Ivana D'Andrea
Mariasilvia D'Andrea
Antonio D'Andrilli
Géraldine Daneau
Allison Danell
Nick Daneman
Radu Danescu
Seyed Danesh-Sani
Hamid Daneshvar
Cathy Danesin
Teresa Danforth
Cuong T. Dang
Haishan Dang
Tran Ngoc Dang
Weiwei Dang
Ganesh Dangal
Rolando D'Angelillo
Anne-Lise D'Angelo
Cecilia D'Angelo
Daniela D'Angelo
Maria Grazia D'Angelo
M. Carolina Danger
Peter H. Dangerfield
Volodia Dangouloff-Ros
Krishna Dani
Nika Danial
Casey Daniel
Catherine Daniel
Hannelore Daniel
Jefferson Daniel
Potter Daniel
Scherr Daniel
Volker Daniel
Antonio Daniele
Aurora Daniele
Silvia Danielian
Alberto Danielli
Salvatore D'Aniello
Anthony Daniels
Jaret Daniels
Kay Daniels
Lori Daniels
Mark Daniels
Matthew Daniels
Melinda Daniels
Ranjit Daniels
Rolf Daniels
Else Danielsen
Finn Danielsen
Anna Danielsson
Louise Danielsson
Amrita Daniere

Valery Danilack
Michael Danilenko
Sergei Danilov
Marina Danilova
Robert Danka
Scott Dankel
Charles Danko
Maciej Danko
Janine Danks
Silvestro Ennio D'Anna
Camille Danne
Erin Dannecker
H Kathleen Dannelly
Antonella D'Anneo
Ng Danny Wang-Kit
Olivier Danot
M. Carolina Danovaro-Holliday
Ina Danquah
Jan Danser
Sarah Danson
Jaya Dantas
Anthony D'Antoni
Nico Dantuma
Robert Dantzer
Heather Danysh
Natalia Danzer
Michael Danziger
Roy Danzmann
Tien Tuan Dao
Georges Daoud
Elena Daprati
Altaf Dar
Lily Dara
Masoud Dara
Masoud Darabi
Radbod Darabi
Faryad Darabi Sahneh
Mohammad Darainy
Shrinivas Darak
Davood Darban
Deepika Darbari
Swaroop Darbha
Pascal Darbon
Brian Darby
Ivan Darby
Steve Darby
Justin Darcy
Carl D'Arcy
Valérie Dardalhon
Marie-Laure Darde
Marie-Laure Dardé
Dominique Dardevet
Alan Dardik
Christina Dardis
Bernard Dardzinski
Mohammad Daremipouran
Fabien Darfeuille
Kwame Darfour-Oduro
Peter Dargaville
Felipe Dargent
Marco D'Arienzo
Michael Dark
Sune Darkner
Victor Darley-Usmar
Emily Darling
Gail Darling
Katharine Darling
Warren Darling
Ben Darlow
Pierre Darlu
Henri Darmency
Antonella d'Arminio Monforte
Michael Darmon
Balint Daroczy
Alexander Daros
Chantal Darquenne
Roland Wolfgang Därr
Taher Darreh-Shori
Amelia Darrouzet-Nardi
Anthony Darrufet-Nardi
Manjula Darshi
Alberto Darszon
Thomas Darton
Nayreen Daruwalla
Richard Darveau
Lancia Darville
Soodabeh Darzi
Amit Das
Ani Das
Anup Das
Anurag Das
Aparup Das
Archita Das
Arindam Das
Arup Das
Ashok Kumar Das
Atze Das
Bhudev Das
Bidyadhar Das
Chandrima Das
Diganta Das
Dipak Das

Gitanjali Das
Jishnu Das
Kumuda Das
Mita Das
Nabanita Das
Pradeep Das
Pranab Das
Raibatak Das
Sabyasachi Das
Sai Das
Salil Das
Sandip Das
Santasabuj Das
Satya Das
Srijit Das
Subha Das
Subir Das
Sushmita Das
Tapan Das
Undurti Das
José das Neves
Krishna Das Saha
Vinod Dasa
Ananda Dasanayake
Srinivasan Dasarathy
Subramanyam Dasari
Eric D'Asaro
Fabrizio D'Ascenzo
Indranil Dasgupta
Piyali Dasgupta
Prokar Dasgupta
Santanu Dasgupta
Sujoy Dasgupta
Aditya Dash
Padmanava Dash
Prasanta Dash
Ranjan Dash
Srikanta Dash
Umakant Dash
Elizabeth daSilva
Silvia Da-Silva
George Daskalakis
Timothy Daskivich
Asok Dasmahapatra
Shawn Dason
E.T. Dassah
Theodore Dassios
Antonino D'Assoro
Babak Dastmalchi
Mehran Dastmalchi
Ravi Dasu
Daniel Datiko
Manuel Datiles
George Datsis
Amitava Datta
Ashim Datta
Kamal Datta
Karabi Datta
Manjula Datta
S. Datta
Sandeep Datta
Simanti Datta
Raymond Dattwyler
Mike Daube
Eric Daudé
William Dauer
Alan Daugherty
Matthew Daugherty
Guenter Daum
Robert Daum
Stéphanie Daumas
Martin Daumer
Yannicke Dauphin
Romina D'Aurizio
Elizabeth Daut
Bruno Dauvier
Brigitte Dauwalder
Katarina Davalieva
Deana Davalos
Veronica Davalos
Fabian Davamani
Nazzareno D'Avanzo
Jetandra Dave
Joel Dave
Vrushank Dave
Emily Davenport
Miles Davenport
Guillaume Daver
Dvora Davey
Gavin Davey
Allan David
Denis David
Erica David
G. David
John David
Lior David
Michael David
Sascha David
Stephen David
Sucunza David
Tim David
Valentin David

Victor David
David David Camilo Corrales
David Davido
Ori Davidov
Adlai Davids
Bill Davids
Malika Davids
Alice Davidson
Ian Davidson
John Davidson
Lance Davidson
Nadia Davidson
Steven Davidson
A. Davies
Anna Davies
Barbara Davies
Benjamin Davies
Chris Davies
David Davies
Donna Davies
Eleanor Davies
Freya Davies
Geraint Davies
M Davies
Michael Davies
Noel Davies
Paul Davies
Robert W. Davies
T. Jonathan Davies
Tammy Davies
Monica Davila
Claudio Dávila-Cervantes
Claudia d'Avila-Levy
Marina Davila-Ross
Pier Paolo D'Avino
Amy Davis
Brian Davis
Charles Davis
Clodoveu Davis
Daniel Davis
George Davis
Grahame Davis
Jennifer Davis
Jenny Davis
Jonathan Davis
Joseph Davis
Josh Davis
Kelliann Davis
Lloyd Mervyn Davis
Mark Davis
Matthew Davis
Melissa Davis
Meryl Davis
Michael Davis
Paul Davis
Robert Davis
Ryan Davis
Thomas Davis
Thompson Davis
Wellington Davis
William Davis
Barbara Davis Goldman
Karen M Davison
Michelle Davison
Vincent Davisson
Christopher Davitt
Stacy Davlin
Marina Davoli
Esmaeil Davoodi-Bojd
Horvatic Davor
Gail Davoren
Martin Davoren
Dominique Davoult
Jean Davoust
Hasan Davulcu
Mohamed Daw
Anurag Daware
Robert Dawe
Amy Dawel
Colin Dawes
Diana Dawes
Piers Dawes
Stephanie Dawes
Suzanne Dawid
Marian Dawkins
R.C. Dawkins
Mahmoud Dawood
Rajinder Dawra
Alison Dawson
Andrew Dawson
Hana Dawson
Jeremy Dawson
Jesse Dawson
John Dawson
Kevin Dawson
Mary R. Dawson
Michael Dawson
Neal Dawson
Sally Dawson
Tamsin Dawson
Carol Dawson Rose
Elizabeth Dawson-Hahn

Ken Dawson-Scully
Brad Day
Carolyn Day
Casey Day
Chi-Ping Day
Darren Day
Jeanne Day
Jo Day
Louise Day
Nancy Day
Nicola Day
Sharlene Day
Stephen Day
Theodore Eugene Day
Mohamud Daya
Gargi Dayama
Victor Dayan
Sudarshan Dayanidhi
Cecilia Dayaraj
Pierre Daye
Hu Dayong
Paul Dayton
Alessandra d'Azzo
Louise D'Cruz
Jonathan D'Cunha
Abhijit De
Alok De
Arpan De
Prithwiraj De
Subhajyoti De
Mauro Henrique de Abreu
Richard De Abreu Lourenco
Enrique de Alava
Wagner de Albuquerque Pereira
Josiane de Almeida
Janice de Almeida Engler
Rafael Zambelli de Almeida Pinto
Guillermo De Anda-Jaúregui
Dominique de Andrade
Mariza de Andrade
María de Andrés
Alexis De Angeli
Kátia De Angelis
Josep Maria de Anta
Max De Antoni Migliorati
Antonio de Aquino Junior
Walter de Araújo
Pedro de Araújo Gonçalves
João de Azevedo
Soledad de Azevedo
Elfride De Baere
Jean-Pascal De Bandt
Eoghan De Barra
Andrea de Bartolomeis
Eric De Bast
Marcio de Bbarros
Pierre De Beaudrap
Louis De Beaumont
Z. Wilhelm de Beer
Dario de Biase
Filomena De Biasio
Birgitte de Blasio
Geertruida de Bock
Bart de Boer
Hugo de Boer
Jacob de Boer
Onno de Boer
Sietse de Boer
W. F. de Boer
Waldo de Boer
Xavier De Bolle
Johann de Bono
Gert Jan de Borst
Jeroen De Bresser
Felipe De Brigard
Stephane De Brito
Lycia de Brito-Gitirana
Marc de Broe
Angela De Bruin
Eduard J. de Bruin
Simone de Bruin
P. J. Nico de Bruyn
Mieke De Buck
Beatriz de Camargo
Kenneth de Camargo
Lorenzo De Carli
Elena De Carolis
Mamede de Carvalho
Gisele de Cássia Gomes
Olga De Castro
Solange De Castro
Isabel De Castro-Orós
Benito de Celis Alonso
Andrea De Cesarei
Philip de Chazal
Francesco De Chiara
Loretta De Chiara
Olivier De Clerck
Kevin De Cock
Marije de Cock
Wouter De Corte
Ayesha De Costa

Philippe De Deurwaerdere
Gerlinde B. De Deyn
Ruth de Diego Balaguer
Manlio De Domenico
Gianmarco De Donato
Magda de Eguileor
Mariarosaria De Falco
Mario De Felice
Massimo De Felici
Andrew De Filippis
Gianpaolo De Filippo
Stefan de Folter
Davide De Francesco
Francesco De Francesco
Raffaele De Francesco
Antonio de Freitas
Sergio de Frutos
Vincent de Gardelle
Bart De Geest
Natacha Marie De Genna
Eco de Geus
Christian De Geyter
Marco De Giovanni
Maria R. C. de Godoy
Dirk de Graaf
Marie-Anne de Graaf
Miranda De Graaf
Pierre de Graan
Jozina De Graff
Domenico De Grandis
Roberto De Guzman
Sybren de Hoog
Ilona de Hooge
Jan De Houwer
Magdia De Jesus
Vinicio de Jesus Perez
Bouke de Jong
Hans de Jong
Ingrid De Jong
Steven de Jong
Trynke De Jong
Hugo de Jonge
Marien De Jonge
Jos F. M. de Jonghe
Roeland de Kat
Ellen De Keyser
Frederik De Keyzer
Willem De Keyzer
Nicholas de Klerk
Annette de Kloet
A. De Koeijer
Harry de Koning
Mariken de Koning
Laetitia de Kort
David de Kretser
Saula de Kreutzenberg
Jose de la Asuncion
Silvia de la Barrera
Albert de la Chapelle
Marcelino de la Cruz
Xavier de la Cruz
Constanza de la Fuente
Leonardo De La Fuente
Ana Laura de la Garza
Rodrigo de la Iglesia
Aurelien de la Lande
Luis de la Maza
Carol de la Motte
Paloma de la Peña
Ignacio de la Riva
David De la Rosa
Ramón de la Rosa
Jorge Luis de la Rosa-Arana
Pilar De la Rua
Jimmy de la Torre
Laura de la Torre
Pedro de la Villa
Jan De Laet
Dylan De Lange
Elvira De Lange
Ellen De Langhe
Luc de Lapeyre de Bellaire
Lisa de las Fuentes
Roxana De las Salas
Vincenzo De Laurenzi
Paul De Lay
Josh de Leeuw
Reny de Leeuw
Giulio De Leo
Laurence de Leval
Enrico De Lillo
Giselle de Lima Peixoto
Mariana S. De Lorenzo
Francis de los Reyes
Antonio De Luca
Daniele De Luca
Leonardo De Luca
Manuela de Lucas
Luciana De Lucia
Ruud de Maagd
Moniek de Maat
Enrique de Madaria

Örjan de Manzano
Giovanni de Manzoni
Ario de Marco
Federico De Marco
Paolo De Marco
Nicola De Maria
Elaine De Martinis
Ivan De Martino
Luisa De Martino
Vanessa de Mello
Silas Nogueira de Melo
Ulisses de Mendonça Braga-Neto
Alex De Menezes
Elizabete de Menezes
Jo De Mey
Pierre De Meyts
Giuseppe De Michele
Carmen De Miguel
Samuele De Minicis
Joachim de Miranda
Leonardo de Miranda
Noel de Miranda
Ricardo de Miranda Azevedo
Mariane de Montalembert
Jacques de Montblanc
Valentina De Monte
Augusto César De Moraes
Carlos De Moraes
Gustavo De Moraes
Luciana de Moraes
Leandro de Morais Cardoso
Leandro de Moura
Miguel de Mulder
Kim de Mutsert
Rene de Mutsert
Dominic De Nardo
Dario De Nart
Pasquale De Negri
Stefaan De Neve
Wim De Neys
Filomena de Nigris
Carlos de Noronha
Gilberto De Nucci
Caio de Oliveira
Cassia de Oliveira
Jarbas Rodrigues de Oliveira
Joana de Oliveira
Lariza Laura de Oliveira
Marcos Roberto de Oliveira
Rodrigo de Oliveira
Rosimeire de Oliveira
Marcia de Oliveira Otto
Juan de Pablo
Annick De Paepe
Michele De Palma
Elio Franco De Palo
Maria Francesca De Pandis
Vilfredo De Pascalis
Eneida de Paula
Solange de Paula Ramos
Juliana de Paula-Souza
Edwin De Pauw
Jose M. de Pereda
Harjo de Poel
A. De Pol
Nick De Regge
Hilde de Reuse
Mark De Ridder
Jan de Riek
Adolfo de Roodt
Giusseppe De Rosa
Alessandro de Rosis
Anita De Rossi
Marco de Rossi
Marina De Rui
Ermenegildo De Ruvo
Dalia De Santis
Giovambattista De Sarro
Caroline De Schacht
Maarten De Schryver
Sophie De Seigneux
Gaston De Serres
Stefano De Servi
Daniele De Seta
Adriana De Siervi
Aruna De Silva
Dinuka de Silva
Shanaka de Silva
Shermin de Silva
Alfonso De Simone
Paolo De Simone
Enrico De Smaele
Charles De Smet
Lina De Smet
Patrícia de Soárez
Dominik de Sordi
Avinash De Sousa
David de Sousa
João Batista de Sousa
Marcilio De Souto
Alex Junior Souza de Souza
Ricardo de Souza

Ward De Spiegelaere
Luca De Stefano
Nicola De Stefano
Rik de Swart
Vincenzo De Tata
Nunziatina De Tommasi
Andre De Troyer
Jaira de Vasconcellos
Renato De Vecchis
Manuel de Vega
Natasha de Vere
Michael de Vibe
Juan Carlos de Vicente
Fabrizio De Vico Fallani
Jill de Vis
Pasquale De Vita
Darryl De Vivo
Marco De Vivo
Steven de Vleeschouwer
Alex de Vos
Anick De Vos
A. P. de Vries
Gerard de Vries
Jantina De Vries
Marieke de Vries
Sieta de Vries
Sjerp de Vries
Jo De Vrieze
Vivian de Waard
Liesbeth De Waele
Damien De Walque
Philippe de Wals
Letty de Weger
Rosemarie De Weirdt
Stephan De Wekker
Rene de Wijk
Saskia de Wildt
Joost de Winter
Emmie de Wit
G. Ardine de Wit
Lenore de Wit
Maartin De Wit
Nicole de Wit
John De Witt
Philip De Witt Hamer
Colin De Young
Barbara de Zalduondo
Daniela De Zio
Martina de Zwaan
Chatsri Deachapunya
Brian Deal
Caroline Dealy
Brian Dean
Jesse Dean
Judith Dean
Julie Dean
Justin Dean
Laura Dean
Michael Dean
Thomas Dean
Adam Deane
Rashid Deane
Don DeAngelis
Greg DeAngelis
Carrie Deans
Tara Deans
Rodrigo DeAntonio
Anthony Dear
Rajib Deb
Michel deBaar
Delphine Débarre
Jean Debarros
Neil Debbage
Isabelle Debeaujon
Florence Debecq-Chainiaux
Lucie Debeffe
Olivier Debeir
Stéphanie Debette
Tadej Debevec
He Debiao
Melissa DeBiasse
Marilyne Debieu
Thierry Debillon
Daniel DeBlasio
Emily DeBoer
Jason DeBoer
P. Debonnaire
Amber DeBono
Miguel Debono
Malgorzata Debowska
Dawid Debowski
Chitrita DebRoy
Swati Debroy
Zach DeBruine
Jennifer DeBruyn
Bettina Debû
Charlotte Debus
Marc Debuyzere
Ellen Decaestecker
Hélène Decaluwe
Julius Decano
Nicola Decaro

B. Rey deCastro
Didier Decaudin
Benoit Dechaumet
Ralf Dechend
Mark Dechesne
Dina Dechmann
Alan W Decho
Stephen Decina
John Deck
Anastasia Deckard
Stijn Deckers
Paul Declerck
Anne Emilie Decleves
Hedwig Deconinck
Lucimary Deconto
Isabelle Decosterd
Anabelle Decottignies
Lieselot Decroix
Tom Decroo
Eva Decru
Adeline Decuyper
Simon DeDeo
Dan Dediu
Scott Dee
Cornelia Deeg
Shelley Deeks
Tim Deelen
Peter Deen
Sarah Deeny
Kishore Deepak
Vishwa Deepak
George Deepe Jr.
Jacques Deere
Cassandra Deering-Rice
Christopher Deery
Pieter Defauw
Kathryn DeFea
Christopher DeFillipi
Emmanuel Defossez
Jason DeFreitas
James DeGaetano
Mariam Degani
Marta Degani
Bruno Degano
Hans Degens
Cyril Degletagne
Alessia Deglincerti
Patrick Degnan
Sandie Degnan
Detlef Degner
Bertrand Degos
Francoise Degoul
David DeGraff
Guiliano Degrassi
Geneva DeGregorio
Lionel Dégremont
Sven Degroeve
Alexi Degterev
Juno Deguchi
Shinji Deguchi
T Deguchi
Toru Deguchi
Frank Dehairs
Kong Dehan
Eric Deharo
William DeHart
Benjamin Dehay
Cristina Dehelean
Edgar Dehesa-Lopez
Mahlagha Dehghan
Mahshid Dehghan
Fariba Dehghani
Mohsen Dehghani
Morteza Dehghani
Roozbeh Dehghannasiri
Ali Dehghantanha
J. Dehling
Nathalie Dehne
Nina Dehnhard
Ahmad Dehpour
Scott Deibel
Tomas Deierborg
Mary Deily
Kristy Deiner
Katrin Deinhardt
Prescott Deininger
Heidrun Deissler
David Deitcher
Jörg Deiwick
Thomas Dejaco
Deirdre DeJean
Li Dejia
Ranjan Deka
Dragana Dekanski
Michael DeKay
Rachel Dekel
Jasja Dekker
Teun Dekker
Koen Dekkers
Hanne Dekort
Juan Carlos del Alamo de Pedro
Cristina Del Amo

Rosa Maria del Angel
David Del Bello
Rosa del Campo
Ignacio del Castillo
Marco Del Chiaro
Kleber Del Claro
Maite Del Collado
Emanuela Del Dottore
G. Del Duca
Andrea Del Fattore
Carlos Del Fresno
Liana Del Gobbo
Maria del Mar Lleò
Paola Del Porto
Borja del Pozo Cruz
Giovanna Del Pozzo
Gregory Del Prete
Maria Ilaria Del Principe
Dominic Del Re
Marzia Del Re
Ines del Rio
Rodrigo Del Rio
Ruth del Río
Silvia Del Ry
Giannino Del Sal
Lorenzo Del Sorbo
Isreal Del Toro
Michela Del Vicario
Jean Delabar
Jacques Delabie
Alexis Delabouglise
Johnny Deladoëy
Christophe Delage
Anne Delagnes
Pierre Delanaye
Matthew Delano
Alexandre Delanoe
John P. Délano-Frier
Keith Delaplane
Cecile Delarasse
Jorge-Luis de-la-Rosa-Arana
Jacques Delarue
Kathleen Delate
Benoit Delatour
Hélène Delatte
Jean-Jacques Delaunay
Benedicte Delaval
Eugene Delay
Lea Delbridge
Antonella Del-Corso
Johann Delcourt
Louise Deldicque
Calin Deleanu
Henri-Jacques Delecluse
Frank DeLeo
Teresa DeLeon
Valerie DeLeon
Krisine DeLeon-Pennell
Dirk Deleu
Thomas Deleuran
Jade Delevaux
Laurie DeLeve
Loic Deleyrolle
Christopher Delgado
Cynthia Delgado
Gabriela Delgado
Rafael Delgado
Susana Delgado
Juan Delgado Moraleda
Rafael Delgado y Palacios
Elsa Delgado-Angulo
Lissette Delgado-Cruzata
Paul Delgado-Olguin
Greg Delgoffe
Benoit Delhaye
Irfan Deli
Gabriele Delia
Matthias Deliano
Luc Deliens
Fani Deligianni
Mario D'Elios
Christine Delire
Aurélie Delisle
Brian Delisle
Jean-Sébastien Delisle
Daniel Delitto
Devy Deliyanti
Carolin Delker
Silvia Della Bella
Maria Della Chiesa
Antonio Della Della Cioppa
Helvécio Della Coletta-Filho
Marina Della Giusta
Chiara Della Libera
Maria Elena Della Pepa
Fulvio Della Ragione
Domenico Della Rocca
Giorgia della Rocca
Camilla Della Torre
Matteo Dell'Acqua
Mario Dell'Agli
Bianca Della-Guardia

Graham Dellaire
Rachael Dellar
D. M. DellaValle
Humberto Dellê
Simona Delle Monache
Sergio Dellepiane
Barbara Deller
Steven Deller
Patrizia Dell'Era
Stephanie Dellicour
K Dellimore
Olivier Dellis
Antonio Dello Iacono
Giacomo Dell'Omo
A. Dellon
Liliana Dell'Osso
Aurelien Delluc
Volker Dellwo
Christine Delmaire
Robert delMas
Eric Delmelle
Daniela Delneri
Jerome Delon
Kristine DeLong
Anita DeLongis
Alejandro Delorenzi
Francois Delori
Maria Deloria-Knoll
Regine Delourme
Marion Delous
Nicolas Delpierre
Constantinos Deltas
Gabriele DeLuca
William DeLuca
Arnaud Delval
Ek del-Val
Yvon Delville
Eric Delwart
Christophe Délye
Frances deMan
Peter Demant
Sophie Demarche
Vincent DeMarco
Jennie DeMarco
Sandra Demaria
David DeMarini
Julie Demars
Edward DeMartini
Sylvia Dematteis
Deeptankar DeMazumder
Andrew Demchuk
Michael D'Emden
Joe Demer
Zsolt Demetrovics
Youstina Demetry
Christian Demeure
Simon DeMeyer
Nele Demeyere
Michael D'Emic
Elizabeth Demicco
Vittorio DeMicheli
Eugene Demidenko
Yuetiva Deming
Ayhan Demirbas
Esra Demirci
F. Yesim Demirci
Ayse Demirhan
Murat Demirtas
Serkalem Demissie
Todd Demmy
Fernando Demora
Sharon DeMorrow
William R. DeMott
Evangelia Demou
Astrid Dempfle
Christopher Dempsey
Emma Dempster
Tim Dempster
Elisa Demuru
Svitlana Demyanets
Vasily Demyanov
Sebastián Demyda Peyrás
Heidy Den Besten
Jeroen den Dunnen
Tom den Heijer
Jeroen den Hertog
Hester M Den Ruijter
Erick Denamur
Nicolas Denancé
Olgert Denas
Jean-Bernard Denault
Thomas Denby
Adam Denes
Francisco Denes
Jeremiah Deneve
Bo Deng
Changchun Deng
Daiyong Deng
Fei Deng
Fenglin Deng
Furong Deng
Guohong Deng

Han-Bing Deng
Hongbing Deng
Jia-Hui Deng
Jian-Jun Deng
Jinyang Deng
Lih-Wen Deng
Lin Deng
Linhong Deng
Naiyang Deng
Qi Deng
Qiang Deng
Qing Deng
Rong Deng
Sophie Deng
Tao Deng
Win-Ping Deng
Xiaoling Deng
Xin Deng
Xinyang Deng
Xuefeng Deng
Xuemei Deng
Xuliang Deng
Ye Deng
Yuan Deng
Yun-Fei Deng
Zeyuan Deng
Zhenhua Deng
Zhiying Deng
ZhongBin Deng
Ziniu Deng
Jörn Dengjel
Andrew Denham
Emma Denham
Ana Denicola
Petra Denig
Cecile Denis
Gerald Denis
Max Denis
Olivier Denis
Vianney Denis
Bozena Denisow
Jeremy Denizot
Thomas Denk
Stuart Denman
Joachim Denner
David Denning
Timothy Denning
Jonathan Dennis
Louise Dennis
Robert Dennison
Jonathan Denniss
Christine Denny
Mark Denny
Thomas Denny
Jerker Denrell
Jo Dens
Arlene Dent
Jessica Dent
Joseph Dent
Paul Dent
Christiane Denys
Gael Denys
Luc Denys
Makarand Deo
Ram Deo
Sarang Deo
Atul Deodhar
Rajendar Deora
Ashok Deorari
Leon Deouell
Ralph DePalma
R. William DePaolo
Christel Depienne
Amaicha Depino
Evelyne Deplazes
Thomas Depner
Marcel Deponte
Stephen Deppen
Saskia Deppermann
Reinhard Depping
Michael DePriest
Bertrand Deputte
Wim Derave
Edward Dere
Maria Chiara Deregibus
Fevzi Derekoy
Jeniffer Derenne
Enrico Derenzini
Chris DeRenzo
Tekalign Deressa
Dusanka Deretic
Zygmunt Derewenda
Maxime Derex
Mykola Dergai
Rachel Derita
Craig Derkay
Eske Derks
Celine DerMardirossian
Veronique Dermauw
Wannes Dermauw
Birgit Derntl
Mickael Deroche

Andrew Derocher
Catherine Derom
Christophe Deroose
Laurie DeRose
Jason DeRouchey
Mathieu Derouet
Ophelia Deroy
Francesco d'Errico
Muriel Derrien
Elda Dervishi
Jill Derwin
Kathleen Derwin
Tatyana Deryugina
Aditya Desai
Karishma Desai
Shyamal Desai
Tejas Desai
Daniel DeSalvo
Anne-Marie Desaulty
Christele Desbois-Mouthon
Jane Desborough
Karl Desch
Peter Deschamps
Stephane Deschamps
Mélanie Deschasaux
Peter DeScioli
Albert Descoteaux
Renaud Descourt
Yves Desdevises
Kobe Desender
Jean Deshepper
Lalit Deshmukh
Manjeet Deshmukh
Rajesh Deshmukh
Rupesh Deshmukh
Sachin Kumar Deshmukh
Jagadish Deshpande
Rucha Deshpande
Giovambattista Desideri
Vincenzo Desiderio
Radhika Desikan
Andini Desita Ekaputri
Danielle Desjardins
Guillaume Desjardins
Camille Desjonquères
Berthony Deslouches
Ann DeSmet
Tom Desmet
François Desmeules
Ed Desmond
Nicola Desmond
Sophie Desmonde
Franck Desmoulin
Michel Desmurget
Christelle Desnues
Giuseppe Desolda
Gernot Desoye
Jean-Claude Desport
Loic Desquilbet
Annie DesRochers
Ram Dessau
Ulrich Desselberger
Clio Dessinioti
Chantal Dessy
Adey Desta
Catherine D'Este
Frank DeStefano
Thomas Desvignes
Roger Detels
Linda Detman
Michael Detmar
John Detre
Jon Detterich
Amanda Dettmer
C. Dettmers
Gabriella d'Ettorre
Alexandra Deufel
Ruud Deurenberg
Michael Deuschle
Alexander Deutsch
Anne Deutsch
Mordechai Deutsch
Douglas Deutschman
Subhabrata Dev
Krishnakumar Devadas
N. Devadasan
Lindsay DeVane
Neal Devaraj
Karthik Devarajan
Sriraman Devarajan
Niveditha Devasenapathy
Sean D'Evelyn
Steve Deverel
Benjamin Deverman
Pradip Devhare
Lakshmi Devi
Rukumani Devi
Jessy Dévieux
Kevin Devine
Rory Devine
S.G. Devine
Shane Devine

Steven Devine
José Devís-Devís
Elise DeVito
Brecht Devleesschauwer
Maureen Devlin
Holli DeVon
Nele Devoogdt
David Devos
Aishwary Devraj
Radhika Devraj
Emmanuel Devred
Bart Devreese
Thomas DeVries
Didier Devys
Oliver Dewald
Ashraf Dewan
Khushboo Dewan
Alex Dewar
Genevieve Dewar
Ken Dewar
Meagan Dewar
Michaela Dewar
Robin DeWeese
Alexander Dewerth
Marc Dewey
Mark Dewhirst
Sylvia Dewilde
Luc Dewit
Jamie DeWitt
Andre DeWolf
Arthur Dewolf
Jeroen Dewulf
Franklin Dexter
Anindya Dey
Ranadhir Dey
Tanujit Dey
Farzad Deyheim
Randy DeYoung
Guillaume Dezecache
Renata Dezengrini Slhessarenko
Cameron Dezfulian
Nicole Deziel
Nicky D'Haene
Ajaya Dhakal
Santosh Dhakal
Naranjan Dhalla
Shayesta Dhalla
Mukesh Dhamala
Danny Dhanasekaran
Saravana Mohan Dhanasekaran
Navneet Dhand
Sivashanmugam Dhandapani
Vignesh Dhandapani
Nirav Dhanesha
Anandh Dhanushkodi
Animesh Dhar
Dolly Wattal Attal Dhar
Purbarun Dhar
Abhay Dharamsi
Shalmali Dharmadhikari
Shyamali Dharmage
Arun Dharmarajan
Permphan Dharmasaroja
Shylaja Dharmesh
Rajat Dharr
Neeraj Dhaun
Jyotsna Dhawan
Punita Dhawan
Rishu Dheer
Waljit Dhillo
Gundeep S. Dhillon
Preet Dhillon
Samjot Dhillon
Sunil Dhiman
Rajinder Dhindsa
Anjali Dhingani
Sanjiv Dhingra
Ali Dhinojwala
V. Dhir
Yatin Dholakia
Thomas D'Hooghe
Eric D'hoore
Sahajal Dhooria
Raja Ram Dhungana
Emily Dhurandhar
Nikhil Dhurandhar
Shalini Dhyani
Jiang Di
Jiangli Di
Xin Di
Zhao Di
Giuliano Di Baldassarre
Mauro Di Bari
Belinda Di Bartolo
Gianluca Di Bella
Claudia Di Bene
Adriana Di Benedetto
Anna Maria Di Betta
Lazzaro di Biase
Danilo Di Bona
Raffaella Di Cagno
Giuseppe Di Caprio

Marcelo Di Carli
Marta Di Carlo
Giuseppe Di Caro
Mariachiara Di Cesare
Riccardo Di Clemente
Valeria Di Cola
Donatella Di Corrado
Valeria Di Dato
Fabio Di Domenico
Marco Di Domenico
Jeremy Di Domizio
Mirko Di Febbraro
Valentina Di Felice
Alessio Di Fonzo
Patrizia Di Fulvio
Simona Di Giambenedetto
Riccardo Di Giminiani
Nick Di Girolamo
Enzo Di Iorio
Vito Di Lernia
Annarita Di Lorenzo
Manfredi Di Lorenzo
Patricia Di Lorenzo
Marco Di Luca
Massimiliano Di Luca
Roberto Di Maio
Luca Di Marco
Alberto Di Martino
Mirko Di Martino
Vincenzo Di Marzo
Michele Di Mauro
Rocco Di Michele
Anna Di Nardo
Corrado Di Natale
Francesco Di Nocera
Javier Di Noia
Alessandro Di Nuovo
Antonella Di Palma
Gilbert Di Paolo
Giovanni Di Perri
Natalia Di Pietro
Antonella Di Pizio
Gian Di Renzo
Gianfranco Di Renzo
Antonio Di Sabatino
Gian-Pietro Di Sansebastiano
Roberto Di Santo
Anna Maria Di Sciullo
Nicoletta Di Simone
Attilio Di Spiezio Sardo
Giuseppina Di Stefano
Valerio Di Vittori
Dolores Di Vizio
Giovanni Di Zenzo
Vasile Diaconu
Marina Diakonova
Dorota Diakowska
Mawlouth Diallo
Luca Diamanti
L. Diambra
Alan Diamond
Betty Diamond
David Diamond
Don Diamond
Joshua Diamond
Honglu Diao
Yarui Diao
Yina Diao
Celeste Dias
Glaecir Dias
M. Dias
Murilo Dias
Sandra Dias
Magnus Dias da Silva
Valdo Dias da Silva
Robert Diasio
Marcus Dias-Souza
Luda Diatchenko
Alexandre Diaz
Carlos Diaz
Estibaliz Diaz
Fernando Diaz
Francisco Diaz
George Diaz
Jose Diaz
Keith Diaz
Luis Diaz
O. Diaz
Olivier Diaz
Patricia Diaz
Julio Díaz
Eva Carolina Diaz Fuentes
Roberto Díaz Peña
María Díaz Roldán
Ramon Diaz-Arrastia
Fernando Díaz-Benjumea
Patricia Diaz-Gimeno
Andrés Diaz-Méndez
Jose Díaz-Mínguez
Antonio Diaz-Quintana
Miguel Díaz-Rodríguez
Alberto Díaz-Ruiz

Kamran Diba
Claudio DiBacco
Leanne Dibbens
Sulayman Dib-Hajj
Gerald DiBona
Wolfgang Dichtl
David Dick
Jeffery Dick
Robert Dick
Marcel Dicke
Borame Dickens
Luke Dickens
Adam Dicker
Anne Dickerson
Seth Dickey
Jeffrey Dickhout
Tom Dickins
Brian Dickinson
Hayley Dickinson
Janis Dickinson
Laura Dickinson
Peter Dickinson
Sally Dickinson
Tim Dickinson
Christopher Dickman
Brett Dickson
Lindy Dickson-Hall
Rebecca Dickstein
Ruth Dickstein
Robert DiCosimo
Christine DiDonato
Aarati Didwania
Jose Die
Kai Diederich
Marc Diederich
Wibke Diederich
Amy Diedrich
Phillippa Diedrichs
Ferran Diego
Vincent Diego
Xavier Diego
Louis Diehl
Philipp Diehl
Chris Diehnelt
Jurgen Dieker
Florian Diekert
Torsten Diekhoff
Rabea Diekmann
Thomas Diekwisch
Diego Diel
Joseph Dieleman
Francesca Diella
Ricarda Diem
Igor Diemberger
Kerrilyn Diener
Benjamin Dieplinger
Andreas Diepold
Forrest Dierberg
Hans Dierckx
Rudi Dierckx
Brenda Diergaarde
Herman Dierick
Jutta Dierkes
Brian Diers
Kevin Dieter
Angela Dieterich
W. Dieterich
Christopher Dietrich
Johannes Dietrich
Lars Dietrich
Muriel Dietrich
David Dietz
Thomas Dietz
Michael Dietze
Ralf Dietzgen
Bernhard Dietzschold
Javier Diez
Nieves Diez
Maria Diez Campelo
E. Diez-Tejedor
Exuperio Díez-Tejedor
Cristina Diez-Vives
Francesco Difato
Jay Diffendorfer
Pamela Diggle
Maria Cristina Digilio
Susan DiGiovanni
Marina Diioia
Marcel P. Dijkers
Lubbert Dijkhuizen
Rick Dijkhuizen
Ronald Dijkman
Lenie Dijkshoorn
J. Dijksterhuis
Bauke Dijkstra
Martijn Dijkstra
Peter Dijkstra
Jan-Willem Dik
Willem Dik
Sergey Dikalov
Yalim Dikmen
Cees Diks

Rajesh Dikshit
Gino DiLabio
Lisabeth Dilalla
Michele Dileone
Barbara Diletto
Anna Dilger
Joseph Dillard
Eric Diller
Kenneth Diller
Norbert Dillier
Marie-Agnès Dillies
Kimberly Dill-McFarland
Michael Dillon
Patrick Dillon
Teresa DiLorenzo
Thomas Dilts
Mark Dilworth
Matt DiMaggio
Nicholas Dimakis
Eugen Dimant
Patrick Di-Martino
Paul Dimayuga
Wayne Dimech
Daniela Dimer Leffa
Sona Dimidjian
Panayiotis Dimitrakopoulos
Antonia Dimitrakopoulou-Strauss
Nicola Dimitri
Emilios Dimitriadis
Slavica Dimitrieva
Moshou Dimitrios
Charles Dimitroff
Alexios Dimitropoulos
Gina Dimitropoulos
Borislav Dimitrov
Dimiter Dimitrov
Kiril Dimitrov
Mauricio Dimitrov
Kai Stefan Dimmer
George Dimopoulos
Stavros Dimopoulos
Rumiana Dimova
Mariapaola D'Imperio
A.P. Dimri
Goberdhan Dimri
Veronica Dimuccio
Muhammad Din
Andrew DiNardo
Charles Dinarello
Renata D'Inca
Diana Dinescu
Birthe Dinesen
Lekha Dinesh Kumar
Andrii Dinets
Chan Ding
Dale Ding
De-Rong Ding
Di Ding
Fei Ding
Feng Ding
Feng-Hua Ding
Guangwei Ding
Guosheng Ding
Guowei Ding
Haitao Ding
Hao Ding
Hongbing Ding
Hu Ding
Huiling Ding
Jian-Jiun Ding
Jianqing Ding
Jie Ding
Jiu Ding
Nenggen Ding
Rui Ding
Shiming Ding
Shuai Ding
Shuzhe Ding
Tao Ding
Wei Ding
Wei-Qun Ding
Weixin Ding
Xiangdong Ding
Xiaotao Ding
Xiaoyu Ding
Xi-Qin Ding
Ya Ding
Yali Ding
Yanfeng Ding
Yezhang Ding
Yi Ding
Yimin Ding
Ying Ding
Yu-Bin Ding
Yuchuan Ding
Zhaojun Ding
Zhaotang Ding
Mark Dingemanse
Calinda Dingenouts
Rhoel Dinglasan
David Dingli
Jonathan Dingwell

Huy Dinh
Thanh Dinh
Thu Dinh
Cecilia Dini
Anca Dinischiotu
Carmen Diniz
Marcio Diniz
Pedro Paulo Diniz
José Alexandre Diniz-Filho
Danae Dinkel
Marc Dinkin
Jonathan Dinkins
Geri Dino
Ivo Dinov
Hubert Dinse
Mary Beth Dinulos
Amanda Diochon
Michel Dione
Joanna Diong
Francesco Dionisi
Giuseppe Dionisio
Audrey Dionne
Francois Dionne
Nicolas Diotel
Luisa DiPietro
Joanna Dipnall
Sabine Dippel
Leila Dirani
Mohamed Dirani
Haner Direskeneli
Manon Dirheimer
Ulrich Dirnagl
Stephan Dirnhofer
Ermias Diro
Anibal Disalvo
Giulio Disanto
Michelle Discacciati
Larry Dishaw
Rod Dishman
Salvatore Disomma
Alan Dispirito
Cheryl Dissanayake
Ottmar Distl
Oliver Distler
Gillian Dite
Michael Ditiatkovski
Ryan Dittamore
Elke Dittmann
Katharina Dittmar
Dirk Dittmer
Ian Dittmer
Keren Dittmer
Maria Dittrich
Ralf Dittrich
Mark Ditzel
Maziar Divangahi
Vincent Dive
Dario Diviani
Paola Divieti Pajevic
Adeline Divoux
Adeleh Divsalar
Brent Dixon
Dan Dixon
Ed Dixon
Elijah Dixon
Jenna Dixon
Linda Dixon
Peter Dixon
Philip Dixon
Barnaby Dixson
Debra Diz
Janine Dizon
Ali Djalilian
Mojgan Djavaheri-Mergny
Alexandre Djiane
Valentin Djonov
Steven Djordjevic
Benjamin Djulbegovic
Mikael Djurfeldt
Dragan Djuric
Petar Djuric
Zora Djuric
Darryl D'Lima
Pawel Dlotko
Daniel Dlugolenski
Jacek Dmochowski
Bao Do
Minh Do
Ton Do
Young Rag Do
Jussara Marcia do Carmo
Frederico Simoes do Couto
Jean Claude Do Rego
Jon Doan
Timothy Doane
Carlota Dobano
Charles Dobard
David Dobbelstein
G. Dobbins
Drena Dobbs
Fred Dobbs
Joannie Dobbs

Sylvia Dobbs
Aneta Dobierzewska
Enrique Doblas-Miranda
Stephan Dobner
Árpád Dobolyi
Karen Dobos
Kerry Dobransky
Anca Dobrian
Alexander Dobrovic
Hana Dobrovolny
Radoslaw Dobrowolski
Steven Dobrowolski
Ekaterina Dobryakova
Marnie Dobson
Simon Dobson
Niv Dobzinski
Fabian Docagne
Roberto Docampo
Anca Docea
Margaret Docker
Douglas Dockery
Sean Docking
Samantha Dockray
Paul Dockree
Henry Doctor
Tamas Doczi
C. Dodd
Christine Dodd
Ian Dodd
Jodie Dodd
Roger Dodd
Ravi Doddapaneni
Jodi Dodds
Peter Dodds
Seth Dodds
Richard Dodel
David Dodell-Feder
Tihomir Dodev
Kimberly Dodge-Kafka
Matthew Dodson
Sabine Doebel
Philipp Doebler
Stefanie Doebler
John Doench
Torsten Doenst
Katja Doerholt
Christian Doerig
Bettina Doering
Don Doering
Jay Doering
Benjamin Doerr
Nina Doerschner
Andrea Doeschl-Wilson
Olaf Doessel
Andreas Doetsch
Paul Doetsch
Sebastian Doetterl
Jean d'Offay
Emine Dogan
Eyup Dogan
Levent Doganay
Aleksandar Dogandzic
Tamara Dogandžic
Sami Doganlar
Catherine Dogimont
Anne Doherty
Colleen Doherty
David Doherty
Loic Dohet
Shinya Dohgu
P. Dohmen
Kent Doi
Toshiki Doi
Yohei Doi
Michel Dojat
Tansel Dokeroglu
Terje Dokland
Daniel Doktor
Martin Dokulil
Ayotunde Dokun
Conor Dolan
Eimear Dolan
Monika Dolejská
Jurij Dolensek
Adam Dolezal
Martin Dolezal
Tomas Dolezal
Jirí Doležal
David Dolezel
Jan Dolfing
Oleg Dolkart
Christine Dollaghan
Dario Doller
Matthias Dollinger
Marie-Madeleine Dolmans
Piero Dolso
Harry Dolstra
Kenji Doma
Teresa Domagala
Alice Domar
Richard Dombroski
Ronald Domen

Francesc Domènech
Alessio Domeneghetti
Kate Domett
Esteban Domingo
Gonzalo Domingo
Mariano Domingo
Ana Domingos
Pedro Domingos
Michael Domingue
Lucília Domingues
Rosa Domingues
Rosario Domingues
Carmen Dominguez
Isabel Dominguez
Ligia Dominguez
Ana Domínguez
Jorge Domínguez
Jose Domínguez
Omar Dominguez-Dominguez
Manuel Domínguez-Rodrigo
Mev Dominguez-Valentin
Paari Dominic
Fernando Dominici
Steubl Dominik
Dan Dominissini
Zachary Domire
Sami Domisch
Robert Domitrovic
Sarah Domoff
Satoru Domoto
Erna Domsgen
Massimo Donadelli
Carlo Donadio
Laura Donahoe
Timothy Donahue
Kirsten Donald
Janet Donaldson
Lloyd Donaldson
Maria Donaldson
Paul Donaldson
Mario Donate
Lars Donath
Giuseppe Donati
Simone Donati
Maria Francesca Donato
Rosario Donato
C. Doncaster
Alena Donda
W Dondorp
Alideertu Dong
Bi-Cheng Dong
Bin Dong
Bingning Dong
Chaoxuan Dong
Chen Dong
Chuanhui Dong
Chunjiaon Dong
Cun-Jian Dong
Gaogao Dong
Hezhong Dong
Hongpo Dong
Jianfei Dong
Jie Dong
Jing-Ming Dong
Jingyan Dong
Junchao Dong
Ke Dong
Li Dong
Liang Dong
Lianhua Dong
Ling Dong
Mianxiong Dong
Min Dong
Qiwen Dong
Ruoyu Dong
Shikui Dong
Shuang-Lin Dong
Shuangling Dong
Suomeng Dong
Wei-Guo Dong
Weihua Dong
Xiang Dong
Xinnian Dong
Yan Dong
Yueping Dong
Yun-Wei Dong
Yuqing Dong
Zheng Dong
Anna Dongari-Bagtzoglou
Paola Dongiovanni
Amol Dongre
Stefan Dongus
Dirk Donker
Franc Donkers
Liesje Donkin
G. Donnan
Deborah Donnell
Christopher Donnelly
Daniel Donnelly
Eve Donnelly
Melanie J. Donnelly
Ryan Donnelly

Sheila Donnelly
Tam Truong Donnelly
Albert Donnenberg
Reik Donner
Josef Donnerer
Macarius Donneyong
Domizia Donnini
Sandra Donnini
Augusto D'Onofrio
Brian D'Onofrio
Daniel Donoghue
Helen Donoghue
Phil Donoghue
Duncan Donohue
Kevin Donohue
Nikos Donos
Paulina Donoso
Lois Donovan
Lucas Donovan
Luke Donovan
Mary Donovan
Mark Donowitz
Curtis Donskey
Ismene Dontas
Monique Dontenwill
Manon Dontje
Gian Paolo Donzelli
James Dooley
Kevin Dooley
William Dooley
Robert Dooling
Xaquin Dopico
Wolfgang Doppler
Yuval Dor
Gabriel Dorado
Rajkumar Dorajoo
Evan Doran
Michael Doran
Olena Doran
Selina Doran
Timothy Doran
Dante J. Dorantes-Gonzalez
John D'Orazio
Michael Dorcas
Srdan Dordevic
David Dore
Gregory Dore
John Dore
Simone Dore
Fernanda Dorea
Sylvain Dorel
Audrey Dorélien
Karine Dore-Mazars
Marc Dorenkamp
Florian Dörfler
Ruslan Dorfman
Akbar Dorgalaleh
Kelly Dorgan
Popa Dorin
Aude Dorison
Dawa Dorje
Tsechoe Dorji
Charles Dorman
Karin Dorman
Michael Dorman
Colin Dormuth
Ronald Dorn
Samuel Dorn
Alex Dornburg
Pierre Dorny
Fabio Doro
Maria Dorobantu
Ginsberg Doron
Christopher Doropoulos
James Doroshow
Jan Dörr
Anne Dorrance
Ellen Dorrepaal
Maria Dorrucci
Caroline Dorsen
E. Ray Dorsey
Grant Dorsey
Kenneth Dorshkind
Beatrice D'Orsi
Maria D'Orsogna
Johannes Dorst
Joseph Dort
Daniel Dory
Cris Dos Remedios
Jean dos Santos
Marco Aurélio dos Santos
Miguel dos Santos
Thais dos Santos
Maria Tereza dos Santos Correia
Edson dos Santos Marchiori
Patricia dos Santos Vigario
Derek Dosdall
Strahinja Dosen
Jimit Doshi
Simit Doshi
Twinkle Doshi
Sibel Dosler

Brian Doss
Michael Xavier Doss
Michelle Dossett
Elliott Dossou-Yovo
Laure Dossus
Petr Dostal
Ejovwoke Dosunmu
Assaf Dotan
Andrea Dotta
Stefan Dötterl
David Doty
Richard Doty
Daolong Dou
Dejing Dou
Horng-Yunn Dou
Hui Dou
Weibei Dou
Wenwen Dou
Xianying Dou
Zhicheng Dou
Luc Douay
Martina Doubková
Vincent Doublet
Mikhaill Doubrovin
Gillian Douce
Alain Doucet
Christine Doucette
Greg Doucette
Karen Doucette
David Douches
Doris Doudet
Anthony Doufas
Maxime Dougados
Dougald Dougald Monroe
Darin Dougherty
Dawn Dougherty
Patrick Dougherty
Kevin Doughty
Paul Doughty
Michael Douglas
Nataki Douglas
Peter Douglas
Richard Douglas
Sandy Douglas
Timothy Douglas
Vanja Douglas
Mathieu Douhard
François Douillard
Katerina Douka
Phaedra Doukakis
Charalampos Doulaverakis
Agapi Doulgeraki
Paschalis-Thomas Doulias
Andreas Doulis
Michail Doumas
Claudie Doums
Natalia Dounskaia
Adam Doupé
Marcia Dourado
Masoumeh Douraghi
Pierre Dourlen
Gael Dournes
Xavier Dousset
Isabel Douterelo
Igor Douven
Renee Douville
Martin Dovciak
John Dovidio
Natalia Dovlatova
Dorothy Dow
Steven Dow
Runjun Dowarah
Michael Dowd
Wes Dowd
Sean Dowdy
William Dowhan
Ann Dowker
Dar Dowlatshahi
John Dowling
Soo Downe
Brian Downer
Laura Downie
Glenna Downing
Colleen Downs
J. Crawford Downs
Jenny Downs
Mark Dowton
James Dowty
Andrew Doxey
Joseph Doyle
Rebecca Doyle
Sean Doyle
Tom Doyle
Vinson Doyle
Ann Dozier
Mikhail Dozmorov
Tiina Dr. Sairanen
Tomas Drabek
Jirí Drábek
Cinthia Drachenberg
Rika Draenert
Ioannis Dragatsis

Luciano Drager
Kurt Draget
Lorenzo Drago
Massimiliano Drago
Alice Dragomir
Davide Dragone
Rebecca Dragovic
Deirdre Dragovich
Eduard Dragut
Paul Drain
Abby Drake
Alison Drake
Andrew Drake
Bettina Drake
Brandon Drake
David Drake
John Drake
Lisa A. Drake
Marcus Drake
Richard Drake
Robert Drake
Wonder Drake
Christopher Drakeley
Shaynoor Dramsi
Michel Drancourt
Maria Drangova
Mark Drangsholt
Brian Dranka
Estelle Dransart
Jaroslaw Drapala
Scott Draper
Julia Dratva
Clemens Draxler
Stéphane Dray
Dennis Drayna
Catherine Dreanno
Didier Dreau
Anna Dreber
Sten Dreborg
Susanne Drechsler
Theo Dreher
Robert Dreibelbis
Victoria Dreitz
Dimitri Drekonja
Jörg-Detlef Drenckhahn
Ray Drenner
Kristen Drescher
Valderi Dressler
Kyla Drever
Mark Drever
Christian Drevon
Joshua Drew
Mark Drew
Patrick Drew
Paul Drew
Chris Drewell
Yvonne Drewes
Sascha Drewlo
H. A. Drexhage
Erwin Dreyer
Florian Dreyer
Gavin Dreyer
Szymon Drgas
Andreas Drichoutis
Laurence Dricot
Denis Drieghe
Michael Drielsma
Steven Driever
William Driggers
Guillaume Drillet
Hans Dringenberg
Eric Drinkwater
Guillaume Drion
Carlos Driscoll
Charles Driscoll
Lori Driscoll
Mark Driscoll
Ryan Driskell
Adel Driss
Steve Dritz
Jane Driver
John Driver
Karl Drlica
David Drobes
Elliot Drobetsky
Szymon Drobniak
Igor Drobyshev
Alexander Drobyshevsky
David Drolet
Marie-Josée Drolet
Mithilesh Dronavalli
Milena Dropa
Konstantinos Drosatos
Casey Droscha
Elefterios Drosinos
Christian Drouet
Régen Drouin
Michalis Drouvelis
David Drover
James Drover
Stanislaw Drozdz
Patrick Druckenmiller

Daniel Drucker
Dorothee Drucker
Uwe Druege
Thomas Druetz
Kirk Druey
Marjan Drukker
Todd Druley
Frank Drummond
Heather Drummond
Rodrigo Drummond
Sean Drummond
Evan Drumwright
John Drury
Suzie Drury
Barry Drust
Maurice Druzin
Jonathan Dry
Nick Drydakis
Scott Dryden-Peterson
Rachel Dryer
Patrick D'Silva
Benjamin D'Souza
Martin D'Souza
Chenyu Du
Guizhi Du
Heng Du
Hongfei Du
Jianchang Du
Jianyang Du
Jinming Du
Juan Du
Li-Jun Du
Li-Lin Du
Meng Du
Min Du
Muqing Du
Ning Du
Pang Du
Peng Du
Ping Du
Pufeng Du
Rose Du
Run-Lei Du
Ruofei Du
Shufa Du
Shushan Du
Wei Du
Weihua Du
Wenbin Du
Xiangjun Du
Xiao-Bo Du
Xiaoming Du
Xuewen Du
Yan Du
Yan-Li Du
Yanzhi Du
Yi Du
Yuanda Du
Yuchun Du
Yuhui Du
Yu-Zhou Du
Zhenyu Du
Zhenzong Du
Zhimin Du
Sascha du Lac
Xianming du Prel Carroll
Johan du Toit
Joss Du Toit
Tarun Dua
Vivek Dua
Changqing Duan
Chuanhua Duan
Chunhui Duan
Fabing Duan
Fenghai Duan
Fengkui Duan
Honglang Duan
Jin-ao Duan
Jinming Duan
Jizhou Duan
Jubao Duan
Kangmin Duan
Lin Duan
Qi Duan
Qingling Duan
Xiaoli Duan
Xiaoqiong Duan
Xichao Duan
Xin Duan
Yanwen Duan
Zhao-Jun Duan
Sureewan Duangjit
Antonio Duarte
Bernardo Duarte
Carla Grigoletto Duarte
Christiane Duarte
Cristiana Duarte
Joao Duarte
Juan Duarte
Julio Duarte
Maria Duarte
Ricardo Duarte

Simone Duarte
Anna Dubaniewicz
Erik Dubberke
Anuradha Dube
John Dube
Michael Dube
Thulani Dube
Timothy Dube
Eve Dubé
Corey Duberstein
Christophe Dubessy
Praveen Dubey
Rama Kant Dubey
Vikash Dubey
Joel Dubin
Patricia Dubin
Margarita Dubocovich
Bertrand Dubois
Jean-Daniel Dubois
Jessica Dubois
Grégory Dubourg
A. Dubovi
Edward Dubovi
Alexandre Dubrac
Sandrine Dubrac
Alexandra Dubrovina
Sasha Dubrovsky
Stephen Dubsky
Laurent Dubuquoy
Zoe Duby
Laurent Duca
Simon Ducatez
Alan Ducatman
James Duce
Jordi Duch
Simon Ducheix
Sandrine Duchemin
Michael Duchen
Sebastian Duchene
Elise Duchesne
Jeff Duckett
John Duckitt
Chloe Duckworth
Joel Ducoste
Lorenzo Ductor
Dan Duda
Ernö Duda
Rachael Dudaniec
Francis Dudás
Khrys Duddleston
William Duddy
Pradeep Dudeja
Vikas Dudeja
Sabine Dudek
Karl Duderstadt
Jaquelin Dudley
Robert Dudley
Diana Dudziak
Danuta Dudzik
Morten Dueholm
P. Barton Duell
María Dueñas
Alfonso Duenas-Gonzalez
Macro Duering
Breck Duerkop
Ralf Duerr
Daniel Duerschmied
Sebastian Duetting
David Duewer
Andrew Duff
Michael Duff
Hugues Duffau
Lisa Duffett
Giles Duffield
Kate Duffus
Aaron Duffy
Margaret Duffy
Michael Duffy
Sonia Duffy
Valerie Duffy
Antoine Dufour
Boris Dufour
Denis Dufrane
Marie Dufresne
Murielle Dufresne
Christophe Dufresnes
Christina Duftner
Lara Dugas
Katherine Duggan
Emilio D'Ugo
Eva Dugoff
Mihai Duguleana
Dagne Duguma
Guy Duhamel
Jorge Duitama
Ashley Duits
Marieke Duiverman
Erwin Duizer
Kathy Dujardin
Zeljko Dujic
Theodora Duka
Jon Duke

Norman Duke
Stephen Duke
Sean Dukelow
Andrew Duker
John Duley
Angela Dulhunty
Jennifer Dulin
Nickolai Dulin
Robin Dullaart
Alexander Dullenkopf
Jacques Dumais
Ronald Duman
Guillaume Dumas
Sarah Dumas
Nicolas Dumaz
Brett Dumbauld
R.B. Dumitru
Roger Dumke
Marc Dumont
Mark Dumont
Michel Dumontier
Jerome Dumortier
John C.M. Dumoulin
Jon Andoni Duñabeitia
Susanna Dunachie
Jamie Dunaev
Joshua Dunaief
Carole Dunand
Christophe Dunand
Neil Dunavin
Ewan Dunbar
James Dunbar
Robin Dunbar
Stephanie Dunbar
W. Dunbar
David Duncan
Elizabeth Duncan
Francesca Duncan
Jacque Duncan
Margaret Duncan
Melinda Duncan
Natalie Duncan
Neil Duncan
Randall Duncan
Robert Duncan
Ryan Duncan
W. Duncan
Wendy Duncan
Ebru Dündar Yenilmez
Ruth Dundas
Frank Dunemann
Pontus Duner
Christopher Dungan
Julieta Dungca
Huzefa Dungrawala
Christine Dunham
Rex Dunham
Ivana Dunic
Curtis Dunkel
Leo Dunkel
Alison Dunkley
Travis Dunkley
Will Dunlop
Adam Dunn
Alexander Dunn
Anne Dunn
Barbara Dunn
Charlotte Dunn
Graham Dunn
Katherine Dunn
Louise Dunn
Michael Dunn
Peter Dunn
Sara-Jane Dunn
Winston Dunn
Colum P. Dunne
John Dunne
Matthew Dunne
Nicholas Dunne
Simon Dunne
Susana Dunner
Lewis Dunnigan
David Dunning
Jake Dunning
Kylie Dunning
Magda Dunowska
Martin Dünser
Joseph Dunsmoor
Micah Dunthorn
Ken Dunton
Franck Duong
Mai Duong
Vu Duong
Stephan Duparc
Benedicte Dupas
John Duperly
Sébastien Duplessis
Robin Duponnois
Bo Dupont
Christophe Dupont
Geneviève Dupont
Pierre Dupont

Sylvain Dupont
Guillaume Dupont-Nivet
Catherine Duport
Jean Dupouy-Camet
Tosha Dupras
Delphine Duprez
Daniel Duque
Tina Dura
Mithila Durai
Senthil Kumar Duraikannu Kailasam
Geetha Durairaj
Dominique Durand
Jean-Baptiste Durand
Marlene Durand
Isabelle Durand-Zaleski
Ana Durán-Quesada
Szonya Durant
Paulo Durao
Francesca Duraturo
Anna Durbin
Joan Durbin
Jaroslava Durdiaková
Andrew Durham
David Durham
Emily Durham
Jennah Durham
Megan Durham
Kerina Duri
David Durica
Nicolas Durier
Marcel Durieux
Olivier Duriez
Rita Marqueti Durigan
Rolf-Alexander Düring
Walter Durka
Hacer Durmus
Joke Durnez
Dion G Durnford
Jennifer Durocher
Olivier Duron
Sandy Durrani
Lindy Durrant
Russell Durrrett
Klaus Dürrschmid
Adil Duru
Esther Dusabe-Richards
Devendra Dusane
Georg Duscher
Robert Dusek
Stacey Dusing
Francisca Dussaillant
G.L. Dusseldorp
Stephane Dussert
Heiko Dussmann
Greg Dussor
Azger Dusthackeer
Michael Dustin
Laura Dutca
Rebecca Dutch
Frédéric Dutheil
Grant Duthie
Malcolm Duthie
Julie Dutil
Heverton Dutra
Lauren Dutra
Tania Dutra
Walderez Dutra
Marie Dutreix
Arin Dutta
Bhabesh Dutta
Dipangkar Dutta
Dipanjan Dutta
Priya Dutta
Rinku Dutta
Samikshan Dutta
Shruti Dutta
Somak Dutta
Trishna Dutta
Tumpa Dutta
Atanu Duttaroy
Melvin Duvall
Johannes Duvekot
Stephane Duvezin-Caubet
Catharina Duvigneau
Beatriz Duvilanski
Mark Duxbury
Bram Duyx
Zeev Dvashi
Bruce Dvorak
Petr Dvorák
Anna Dvorkin-Gheva
Alexey Dvornikov
Bilikere Dwarakanath
Melinda Dwinell
Michael Dwinell
Chandradhar Dwivedi
Shailendra Dwivedi
Sourabh Dwivedi
Elke Dworatzek
Symon Dworjanyn
Andrew Dwork
Jonathan Dworkin

Shari Dworkin
Daniel Dwyer
Donard Dwyer
Ross Dwyer
Laura Dwyer-Lindgren
Michel Dy
Shetty Ravi Dyavar
Kristen Dybala
Bartlomiej Dybiec
David Dyck
Philip Dydynsky
Andrew Dyer
David Dyer
William Dyer
Emily Dykhuizen
Brad Dykstra
Arielle Dylan
Lars Dyrskjot
Tomasz Dysarz
Muriel Dysli
Michele Dyson
Sue Dyson
Petter Dyverfeldt
Rhonda Dzakpasu
Susie Dzakpasu
Nicolas Dzamko
Boris Dzantiev
Rosemary Dziak
Edward Dzialowski
Roman Dziarski
Piotr Dziegiel
Michelle Dziejman
Andrzej Dziembowski
Yvonne Dzierma
Andrzej Dzionek
Thomas Dziubla
Jean Paul Dzoyem
Bogdan Dzyubak
Oleksandr Dzyubak
