# Supplementary material for: PLOS ONE 2016 Reviewer and Editorial Board Thank You
Source: PLoS One. 2017 Mar 20;12(3):e0174259. doi: 10.1371/journal.pone.0174259 (PMC5358840; doi:10.1371/journal.pone.0174259)
Supplement: S2 Reviewer List — (PDF) [file pone.0174259.s003.PDF]

PLOS ONE would like to thank all those who reviewed on behalf of the journal in 2016:

Muthukrishnan Eaaswarkhanth
Shaun Eack
Tanya Eadie
Anne Eady
Elizabeth Eakin
Michelle Eakin
Robyn Eakle
Steven Ealick
Gammon Earhart
J. Mason Earles
Eric J. Earley
David Earnest
Christine East
Malcolm East
Alan Eastman
Alison Eastman
Ronald Eastman
Alexander Easton
Douglas Easton
Roger Easton
Gareth Eaton
Jennifer Eaton
John E Eaton
Julian Eaton
Nicholas Eaton
Julian Eaton-Rye
Connie Eaves
Stephen Ebbs
Anne Ebeling
Mark Ebell
Robert Eber
William Eberhard
Meghan Eberhardt
Charles Eberhart
Johann Eberhart
Ivano Eberini
Delphine Eberle
Isaac Eberstein
Allison Ebert
Martin Ebert
Steven Ebert
Thomas Ebert
Timothy Ebert
Tom Ebert
Hiromichi Ebi
Hideki Ebihara
Ken Ebihara
Lisa Ebihara
Martin Ebinger
Takashi Ebisawa
Jeff Eble
Scott Eblen
Brendan Ebner
Augustine Ebonyi
Mahdi Ebrahimi
Mansour Ebrahimi
Mohammad R. Ebrahimkhani
Ersoy Ebruortac
Joshua Eby
Jacquelynne Eccles
David Echevarria
Maria Echeverria
Sandra Echeverria
Valentina Echeverria Moran
Reinalyn Echon
Kristin Eckardt
Wolfgang Ecke
Kristin Eckel-Mahan
Jeanette Eckel-Passow
Maryellen Eckenhoff
Gerhard Ecker
Ullrich Ecker
David Eckersall
Catherine Eckert
Danny Eckert
Erin Eckert
Gunter Eckert
Benjamin Eckhardt
Vincent Eckhart
Noam Eckshtain Levi
Anja Eckstein
Hans Eckstein
Torsten Eckstein
C. Eckstrand
Anastassios Economou
Nicholas-Tiberio Economou
Shigetoshi Eda
Chandrakanth Edamakanti
Jeffrey Edberg
Saadia Eddahibi

Kamryn Eddy
Tyler Eddy
Jean-Marc Edeline
Brad Edelman
Elazer Edelman
Robert Edelman
Winfried Edelmann
Leonard Edelstein
Paul Edelstein
Guinevere F Eden
John-Sebastian Eden
Kristin Eden
Howard Edenberg
Iris Eder
Klaus Eder
Dumessa Edessa
Rosy Edey
Christopher Edgar
John Edgar
Michael Edge
Mira Edgerton
Dianna Edgil
Gudrun Edgren
Nina Edin
Kevan Edinborough
Jeffrey Edleson
Mark Edlund
Shannon Edmed
Andrew Edmonds
Mark Edmondson-Jones
Kyle Edmunds
Peter Edmunds
Joshua Edokpayi
Sheikh Edrisi
Eric Edsinger
Rolf Edvardsen
Thor Edvardsen
Bruce Edwards
Christine Edwards
Elizabeth Edwards
Erika Edwards
Genea Edwards
Holly Edwards
Jennifer Edwards
Jeremy Edwards
Joan Edwards
Jode Edwards
John Edwards
Kathryn Edwards
Katie Edwards
Mark Edwards
Marten Edwards
Sandra Edwards
Scott Edwards
Thomas Edwards
Will Edwards
J. Edwardson
Vijay Kumar Eedunuri
Christian Eek
Jelmer Eerkens
Erkki Eerola
Burak Efe
Rita Effros
Jimmy Efird
Nikolaos Efstathiou
S. Efstathiou
Eftekhar Eftekharpour
Orestis Efthimiou
James Egan
Jonathan Egan
Paul Egan
Suhelen Egan
Thomas Egan
Timothy Egan
Naohiro Egawa
Chinaza Egbuta
Markus Ege
Marcos Egea Gutiérrez-Cortines
Thore Egeland
Agneta Egenvall
Louise Egerton-Warburton
Adam Eggebrecht
Roger Eggen
Jos Egger
Thomas Eggermann
Maren Eggers
Lori Eggert
Gösta Eggertsen
Aimee Eggler
Karen Eggleston
Shahin Eghbalsaied
Moritoki Egi
E. Sócrates T. Egito
Andrea Egizi
Markus Egli
David Eglin
Michael Egmont-Petersen
Natalia Egorova
Akiko Eguchi
Eri Eguchi
Kazuo Eguchi
Mitsuru Eguchi

Tomoharu Eguchi
Yutaka Eguchi
Manuel Eguia
Oluwaseun Egunsola
Thomas Egwang
Tatsuya Ehara
Johannes Ehinger
J. Ehlen
Bernhard Ehlers
Frederick Ehlert
Ann-Christine Ehlis
Jens Ehmcke
Ann Ehrenhofer-Murray
Eli Ehrenpreis
Mark Ehrensberger
Anja Ehrhardt
Harald Ehrhardt
Frédéric Ehrler
Henry L. Ehrlich
Lauren Ehrlich
Michelle Ehrlich
Samantha Ehrlich
Stephan Ehrmann
H. Henrik Ehrsson
Sabine Ehrt
Nasreen Ehtesham
Solmaz Ehteshami-Afshar
Matthias Eiber
Christine Eibner
Yvonne Eiby
Maryna Eichelberger
Patrick Eichenberger
Susan Eicher
Markus Eichhorn
Sabine Eichinger
Jerry Eichler
Naomi Eichorn
Olivier Eichwald
Tom Eickbush
Peter Eickholz
Olaf Eickmeier
Arne Eide
David Eide
Ivar Eide
Lee Eiden
Martin Eiden
G. Eid-Lidt
Joe Eifert
Marsha Eigenbrodt
Rachel Eiges
Bart Eijkelkamp
Robert Eikelboom
Fritz Eilber
Karl-Erik Eilertsen
Eric Eils
Takahiro Einama
Gisli Einarsson
Danit Ein-Gar
Mark Einstein
Alfonso Eirin
Jose M. Eirin-Lopez
Noemi Eiro
Anne Eischeid
Robert Eisele
Thomas Eisele
James Eisenach
Sarah Eisenacher
Lea Eisenbach
Michael Eisenbach
Jonathan D. Eisenback
Hedwig Eisenbarth
Michael Eisenberg
Tobias Eisenberg
Naomi Eisenberger
David Eisenman
Ian Eisenman
Andreas Eisenreich
Toby Eisenstein
Yoshinobu Eishi
John Eisses
Michael Eitel
Daniel Eitzman
Thomas Eiwegger
Matilde Eizaguirre
Georgia Eizenga
Elzbieta Ejdys
Keisuke Ejima
Anna Ek
Chaitanya Ekanadham
Robert Ekart
Naomi Ekas
Mikael Ekblad
Andras Eke
Orjan Ekeberg
Harald Ekedahl
Maria Ekholm
Marc Ekker
Stephen Ekker
Johan Eklof
Elizabeth Eklund
Bertil Ekman
Burcin Ekser

Ravid Ekshtain
B. Eksteen
Michael Ekstrand
Curt Ekstrom
Jorgen Ekstrom
Marie Ekstrom
Ekanem Ekure
Obinna Ekwunife
Sahar El Aidy
Hicham El Alaoui
Abderrazak El Albani
Mahmoud El Azzouny
Jamila El Baghdadi
Regina El Dib
Oussama El Far
Nady El Hajj
Nour El Helou
C. El Khoury
Graziella El Khoury
Marc El Khoury
Mohamed Réda El Ochi
Abdelfattah El Ouaamari
Rashika El Ridi
Omar El Shahawy
Adil El Taghdouini
Farah El Turk
Dominique Eladari
Sherweit H. El-Ahmady
Khalid Elased
Mohamed Elasri
Mohamed Elati
Sameh Elawady
Tatiana El-Bacha
Ahmed El-Badawy
Mohamed El-Badry
Mona El-Bahrawy
Nabila El-Bassel
Batya Elbaum
Alexis Elbaz
Mohamad Elbaz
Tamer Elbaz
Brian Elbel
Ulf Elbelt
Nieke Elbers
Roy Elbers
Thomas Elbert
Tarek El-Bialy
Roger Elble
J. Stuart Elborn
Mustapha El-Bouhssini
Adrian Elcock
Samir El-Dahr
Leanne Elder
Alice Eldridge
John Elefteriades
Ioanna Eleftheriadou
Ioannis Eleftherianos
Eleftherios Eleftheriou
Anna Maria Eleuteri
Douglas J Eleveld
Mohammed El-Faki
Iman Elfeddali
Samia Elfekih
Achim Elfering
Marlies Elfrink
Malin Elfstrand
Ahmed El-Geneidy
Christian Elger
Lynda Elghazi
Nazira El-Hage
Jeff Elhai
Jon Elhai
Yuval Elhanati
Sayed El-Hussieny
Maurizio Elia
Theodore Eliades
Damian Elias
Harold Elias
Kevin M Elias
Rosilene Elias
Sjoerd Elias
Waldir Elias
Manuel Elías-Gutiérrez
Erika Eliason
Sebastian Eliason
A. Eliassen
Mats Eliasson
Pernilla Eliasson
Brian Eliceiri
Marie-Carmelle Elie
Elaine Elion
Vladimir Elisashvili
Roman Eliseev
Andrey Eliseyev
Areej El-Jawahri
Ronit Elk
Stella Elkabes
Mohammed El-Kebir
Ashraf El-Kereamy
Michael Elkin
Peter Elkin
Robert Elkin

Karen Elkins
Kelly Elkins
Mark Elkins
Sofiane El-Kirat-Chatel
R. Ellahi
Rahmat Ellahi
Timothy Ellam
Anne Ellaway
Elizabeth Elle
Marianne Ellegaard
Nicholas Ellens
Michael Eller
Edward Ellerbeck
David Ellerby
Wolfgang Ellermeier
Sandrine Ellero-Simatos
Evan Ellicott
Lesley Ellies
David Elliman
Lotta Ellingsen
Andrew Elliot
Joshua Elliot
Candace Elliott
David Elliott
Ivo Elliott
James Elliott
Kyle Elliott
Lindsay Elliott
Michael Elliott
Carla Ellis
David Ellis
Emily Ellis
Jessica Ellis
Julie Ellis
Kathryn Ellis
Lee Ellis
Martin Ellis
Murray Ellis
Richard Ellis
Terri Ellis
Vincenzo Ellis
William Ellis
Melinda Ellison
Timothy Ellmore
J. Clive Ellory
David Ellsworth
Peter Ellsworth
Rachel Ellsworth
David Ellwood
Nader El-Mallawany
Sammy Elmariah
Matthew Elmes
Craig A. Elmets
E.M. Elmistekawy
Andrew Elmore
Adel El-Naggar
Amr M. Elnaghy
Eldad Elnekave
Mohammad N. Elnesr
Yusra Elobaid
Emiley Eloe-Fadrosh
Marc Eloit
M.T. Elola
Latesha Elopre
Felix Elortza
Roberto Elosua
Maurice Elphick
Khaled El-Razik
Mona ElRefaey
Azza El-Remessy
Wael El-Rifai
John Elrod
Orna Elroy-Stein
Mayada Elsabbagh
Marwan El-Sabban
Hend ElSayed
Dena El-Sayed
Yasser El-Sayed
Julia Sarah El-Sayed Moustafa
Hamdy Elsayed-Awad
Tobias Else
Helen Elsey
Hatem Elshabrawy
Abdelrahman Elshafay
Adel El-Shahat
Mostafa Elshahed
Hossam ElShamaa
Wael ElShamy
Ahmed El-Shamy
Morad Elshehabi
Ann Elsner
Ari Elson
Joanna Elson
Evelyne Elst
Eric Elster
John Elsworth
Mohamed El-Tahan
Maha ElTantawi
Mohamed El-Tholoth
E.M. Elvander
Bjarki Elvarsson
Rune Elvik

Mostafa Elwany
Jean Elwing
Tobias Elze
Mauricio Elzo
Hany Emam
Mohammad Hassan Emamian
Amir Emamifar
Mohammad Emaneini
Jonathan Emberson
Marina Emborg
Luni Emdad
Connor Emdin
Michele Emdin
Sarah Emel
Guillaume Emeriaud
Charles Emerson
Ben Emery
Carolyn Emery
Derek Emery
Lindsay Emery
Lisa Emery
Vince Emery
E. Emerye
Lillian Emlet
Michael Emmert-Buck
Lorenzo Emmi
Edward Emmott
Jennifer Emond
Katsura Emoto
Nuno Empadinhas
Jean-Philippe Empana
Kerry Empey
M. Emran
Tamás Emri
Scott Emrich
Theresa Emser
Emmanuel Emukah
Xie En
Masao Enari
Juan Encinas
Teresa Encinas
Vincent Encomio
Gabriele Ende
Jeffrey Endelman
Heiko Enderling
Felicity Enders
Georg Enders
Nancy Endersby
Hidenori Endo
Kenji Endo
Shogo Endo
Tamao Endo
Tomoyuki Endo
Yuichi Endo
Jérôme Endrass
Zoltan Endre
Theodore Endreny
Kristina Endres
Thomas Endres
Marco Endrizzi
Mark Endsley
Charis Eng
Kevin Eng
Swantje Enge
Annerose Engel
Corinna Engel
Esteban Engel
Jasper Engel
Jerome Engel
Paul Engel
Suzanne Engelberg
Jüri Engelbrecht
Michelle Engelbrecht
Lina Engelen
Georg Engelhard
Brabara Engelhardt
Britta Engelhardt
Katia Engelhardt
Sacha Engelhardt
Stefan Engelhardt
Simon Engelhart
Julia Engelhorn
Alan Engelman
Ilka Engelmann
Jeffrey Engelmann
Tim Engels
Kaare Engkilde
Patrick England
Sarah England
S. Walter Englander
Jan Engler
Judson Englert
Stefan Englert
Megan English
William English
Dario Englot
Erin Englund
Julieta R. Engracia de Moraes
Daniel Engstrom
David Engstrom
Francisco Enguita
Francis Enjalbert

Luis Enjuanes
Jacob Enk
Vanjildorj Enkhchimeg
Francis Ennis
Irene Ennis
Richard Ennos
Eva Enns
Gregory Enns
Murray Enns
Mary-Anne Enoch
Atsushi Enomoto
Hirayuki Enomoto
Keisuke Enomoto
Ryan Enos
Susana Enríquez
Pooria Ensafi
Sally Entrekin
Natacha Entz-Werle
Yeetey Enuameh
Young-Ho Eom
Ralph Epaud
Elissa Epel
Mariastella Epifanio
Meira Epplein
Joerg Epplen
Timothy Eppley
Margaret Eppstein
C. Epsinola
Sacha Epskamp
Irving Epstein
Jonathan I. Epstein
Lynn Epstein
Terence Epule Epule
Antti Eranti
Francois Erard
Rajiv Erasmus
Holger Erb
Matthias Erb
Tobias Erb
Christine Erbe
Raimund Erbel
Nuran Ercal
Mária Ercsey-Ravasz
Esther Erdei
Karoly Erdelyi
Scott Erdman
John Erdman Jr.
Gabor Erdoes
Mesut Erdurmus
Gulnihal Eren
Metin Eren
Ido Erev
Offer Erez
Gisela Erf
Ozgur Ergul
Özgür Ergül
Ozlem Ergun
Hans Erhard
Elvira Erhardt
Marc Erhardt
Paul Erhardt
Ghigo Eric
M.S. Erich
Amy Erickson
Mary Erickson
Peter Erickson
Keith Ericson
Aaron Ericsson
Göran Ericsson
Erik Fink Eriksen
Jason Eriksen
David Erikson
Anders Eriksson
Jonatan Eriksson
Niclas Eriksson
Ulf Eriksson
Ivan Erill
Mason Erkelens
Roy Erkens
Özüm Erkin
Berhanu Erko
Danielle Erkoboni
Armin Erlacher
Jon Erlandson
Kristine Erlandson
David Erle
Judith Erlen
Silvio Erler
Sten Erm
Burak Erman
Megan Ermler
Vinicius Ernani
Markus Ernst
Pierre Ernst
Robert Ernst
Sinja Ernst
Victor Erokhin
Onur Erol
Dirk Erpenbeck
Youssef Errami
Gian Erre
Robert Erskine

Ebru Ersoy
Burcu Ersoz
Andrew Ertel
Pernille Erthmann
Ali Erturk
Gary Ervin
Karim Erzini
Pedro Esbrit
Ananias Escalante
Carlos Escalante
Ricardo Escalante
Teresa Escalante
Arthur Escalas
Alexandre Escande
Ricardo Escarcega
Alexandre Escargueil
Jose Escario
Romain Eschalier
David Eschenbach
Oxana Eschenko
Rémi Esclassan
Ana Escobar
Carolina Escobar
María Escobar
César Escobedo-Bonilla
Francisco Escolano
Rosa M. Escorihuela
Valentina Escott-Price
Carmen Escribano
Joaquin Escribano
Julio Escribano
Silvia Escribano
Marcial Escudero
Danielle Escudero Poole
Blanca Escudero-Abarca
Ehsan Esfahani
Semih Esin
Nwadiuto Esiobu
Farsad Eskandary
Anu Eskelinen
Paul Esker
Mohammed Eslam
Medi Eslani
Amy Esler
Aryan Esmaeili
Morteza Esmaeili
Saber Esmaeili
Hanif Esmail
Mahdi Esmaily-Moghadam
Xavier Espadaler
Edgar Espana
Malena Espanol
Regina Espanol-Suner
Mateu Espasa
Carmen Espejo
Olivier Espéli
Toomas Esperk
Daniel Espes
Eduardo Espeso
Gemma Espí-López
Eloy Espin
Antonio Espín
Virginia Espina
Daniel Espino
Avelina Espinosa
Manuel Espinosa
Santiago Espinosa
J. Luis Espinoza
Luis Espinoza
Edward Esplin
Alfonso Esposito
Christian Esposito
Elga Esposito
Felice Esposito
Francesco Esposito
Giovanni Esposito
Giuseppe Esposito
Irene Esposito
Lauren Esposito
Tomaso Esposti Ongaro
Johan Espunyes Nozières
Deborah Espy
Mark Espy
Aurora Esquela-Kerscher
Kobina Essandoh
Marieke A.G. Essers
Aleks Essex
M. Essex
Jennifer Essler
Sandrine Essouri
Beverley Essue
Cristian Estades
Jennifer Estall
David Este
Andres Esteban
Maria Angeles Esteban
Irene Esteban-Cornejo
Silvia Estein
Dave Estell
Fernando Estellés
Matt C. Estep
Ryan Estep

Edward Ester
Chris Estes
James Estes
Jordi Esteve
Pierre-Olivier Esteve
David Esteve-Bruna
Maria Esteve-Gassent
Pedro Esteves
Sandro Esteves
Alicia Estevez
Ana Estevez
Mario Estevez
Rodrigo Estévez
Raul Estèvez- Povedano
Janne Estill
Ernesto Estrada
Marta Estrada
Vicente Estrada
Teresa Estrada-Garcia
Sylvie Estrela
Nicole Estrella
Ramon Estruch
Guillem Estruch Cucarella
S. Eswaramoorthi
Hari Eswaran
Ari Eszter
Adolfo Etchegaray
Heather Etchevers
Paula Eterovick
William Etges
Steen Ethelberg
Susan P. Etheridge
Tim Etheridge
Stephen Ethier
Amit Etkin
Jennifer Etnier
Masumi Eto
David Eton
Jean-François Etter
Nicole Etter
Rüdiger Ettrich
Ed Etxeberria
Vicken Etyemezian
Carol Etzel
Paul Eubig
Sung Yong Eum
Frances Eun-Hyung Lee
Andeol Evain
Pavlos Evangelidis
Adriane Evangelista
Arturo Evangelista Masip
Evangelos Evangelou
A. Evans
Alyssa Evans
Ben Evans
Brian Evans
Carlton Evans
Caswell Evans
Catherine Evans
Charlesnika Evans
Christopher Evans
Elizabeth Evans
Gareth Evans
Gethin Evans
Gregory Evans
Jacqueline Evans
Janice Evans
Jennifer Evans
Karl Evans
Mark Evans
Nathan Evans
Phil Evans
Richard Evans
Roger Evans
Theodore Evans
Todd Evans
Tom Evans
Carmella Evans-Molina
Patrick Even
Sergine Even
Maya Evenden
Andrew Evens
Øystein Evensen
Don Evenson
Daphne Everaerd
Mark Everard
Caleb Everett
Eric Everett
Mark Everett
Melanie Everitt
James Evermann
Andrea Evers
Jochem Evers
Maurits Evers
Rens Evers
Stefan Evers
Stefanie Evers
Stephen Everse
Helen Everts
Vincent Everts
Sebastian Eves-van den Akker
Antonio Evidente

Alex Evilevitch
Zoran Evtoski
William Eward
Paul Ewart
Katherine Ewel
Joshua Ewen
Andrew Ewer
John Ewer
David Ewert
James Ewing
Rob Ewing
Filippos Exadaktylos
Jean-Louis Excler
Thomas Exner
Gal Eyal
Willard Eyestone
Thomas Eykyn
Lisa Eylerd
Isobel Eyres
Thor Eysteinsson
Ilker Eyüpoglu
Nadine Ezard
Toshihiko Ezashi
Tatsuhiro Ezawa
Tariq Ezaz
Echezona Ezeanolue
Uthayashanker Ezekiel
Justin Ezekowitz
Vanessa Ezenwa
Lourdes Ezpeleta
E.C. Ezugwu
Yoichi Ezura
Shereen Ezzat
Khaled Ezzedine
Sayeh Ezzikouri
Kay Faaberg
Marijke Faas
Kate Faasse
Elsa Fabbretti
Andrea G. Fabbri
Chiara Fabbri
Elena Fabbri
Leonardo Fabbri
Carsten Faber
Cornelius Faber
Klaas Faber
Klaas Nico Faber
Pascal Faber
Tim Faber
Francois Fabi
Marie Fablet
Thomas Fabre
Jorge Fabres
Marc Fabri
Gabriel Fabricius
Jeffrey Fabrick
Luca Fabris
Angelo Facchiano
Francesco Facchiano
Luca Facchinelli
Fabio Facchinetti
Fabrizio Facchinetti
Livia Facchinetti
Gaetano Facchini
Andrea Facoetti
Mitchell Faddis
Firas Fadel
Howard Faden
James Fadool
James M. Fadool
Bruno Fady
Mario Faenza
Marina Faerman
Camille Faes
Mary Fafutis-Morris
Anne Fagan
Robert Fagan
Susan Fagan
Erik Fagerholm
Anne Fages
Sharmila Fagoonee
Nelson Fagundes
Jaime Fagúndez
Robert Fahey
Magid Fahim
Jahan Fahimi
Mia Fahlen
Noah Fahlgren
Nicole Fahner
Susan Fahrbach
Astrid Fahrleitner
Christoph Fahrni
Gregory Fahy
Ana Elizabeth Fai
Siu Fai Lee
Fernanda Faião-Flores
Dan Faibish
Jozsef Fail
Dorothée Faille
Patricia Fair
Trudee Fair
Jeremy Fairbank

Stacy Fairbanks
Matt Fairbarns
Kaci Fairchild
Kimberly Fairchild
Stuart Fairclough
Rick Fairhurst
Anna Marie Fairhust
Christopher Fairley
Derek Fairley
Robert Fairman
Andrea Fais
Fazle Faisal
Raphael Faiss
Andreas Faissner
Francesco Faita
Tyler Faith
Jean-Philippe Faivre
Dennis Faix
Jan Faix
Maryam Faiz
Carmen Fajardo
Giovanni Fajardo
Robert Fajardo
Jiri Fajkus
Merritt Fajt
Fadi Fakhouri
Kiatichai Faksri
Ahmad Falahatpisheh
Katia Falasca
Silvia Falasca
Monica Falautano
Luiz Falcao
Mario Falchi
Arnaud Falchier
Diego Falci
Elisabetta Falcieri
Silvia Falcinell
Rita Falcioni
Erik Falck-Pedersen
Marika Falcone
Henrik Falconer
Jane Falconer
Hanne-Lise Falgreen Eriksen
Bareket Falk
Roni Falk
Michael Falkenstein
Peter Falkingham
Joseph Falkinham
Michael Falkowski
Mazyar Fallah
Hossein Fallahi
Peter Fallesen
Dorothy Fallows
Anthony Falluelmorel
Tiago Falótico
Tahereh Falsafi
Benedetto Falsini
Jim Falter
Julian Falutz
Elizabeth Falwell
Laura Falzon
Owen Falzon
Barbara Fam
Mary Familari
Itziar Familiar
Giuseppe Familiari
Nicholas Famoso
Michael Famulare
Arthur Fan
Bao Jian Fan
Bing Fan
Chuanmao Fan
Chuanzhu Fan
Chuchuan Fan
Daping Fan
Dayong Fan
Dong-Li Fan
Fenliang Fan
Guichao Fan
Guo-Chang Fan
Haojun Fan
Heng-Yu Fan
Hong-Jie Fan
Hsien-Yu Fan
Hueng-Chuen Fan
Hugh Fan
Jibiao Fan
Jie Fan
Jingyao Fan
Junfeng Fan
Jun-Yu Fan
Li Fan
Lida Fan
Lin Fan
Liuping Fan
Longling Fan
Meng Fan
Ming Fan
Peilei Fan
Pi-Chuan Fan
Shengjun Fan
Shou-Zen Fan

Victoria Fan
Wei Fan
Xiaoming Fan
Xing Fan
Xing-Jun Fan
Yawen Fan
Yingchang Fan
Yingying Fan
Yong Fan
Youran Fan
Zhaofei Fan
Zhiqiang Fan
Mónica Fanarraga
Bronwyn Fancourt
Yana Fandakova
Joachim Fandrey
Vito Fanelli
Rosa Faner
Bin Fang
Carolyn Fang
Chao Fang
Chi-Tai Fang
Chun Fang
Di-An Fang
Dong Fang
Dongdong Fang
Evandro Fei Fang
Fude Fang
Guor-Cheng Fang
He Fang
Jia Fang
Jiahu Fang
Jianqiao Fang
Jing Fang
Jun Fang
Keyan Fang
Kuan-Chieh Fang
Marong Fang
Pengquian Fang
Pu Fang
Qiang Fang
Qingming Fang
Rong Fang
Shengyun Fang
Tilin Fang
Wanping Fang
Wei Fang
Xiangling Fang
Xiangming Fang
Xianjun Fang
Xianping Fang
Xu Fang
Yu Fang
Zhuo Fang
Jonatan U. Fangel
Renan Fangel
Nann A. Fangue
Christopher Fang-Yen
Melpomeni Fani
Cathy Fann
Philip Fanning
Séamus Fanning
Vassilios Fanos
Dimitrios Fanourakis
Thomas Fanshawe
Jeremie Fant
Dean Fantazzini
Paolo Fanti
Maria Pia Fantini
Silvia Fantozzi
Alessandro Fanzani
Franco Faoro Faoro
Ali Faqi
Mary Farach-Carson
Frank Faraci
Ahmed F. Farag
Mohamed Farag
Ugo Faraguna
Farzam Farahmand
Roman Farana
Norman Farb
Masood Fard
Marie-Laure Fardeau
Jeremiah Farelli
Jawad Fares
Mohamed-Bilal Fares
Diego Farfan-Arribas
Jean-Christophe Farges
Silvia Fargion
Maha Farhat
Walid Farhat
Nuno Faria
Davi Farias
Leonardo Farias
Miguel Farias
Talha Farid
Erik Farin
Almo Farina
Cinthia Farina
Marcelo Farina
Carmen Fariñas
Jose Fariñas-Franco

Alessandro Farini
Daniel Farinotti
Mariangela Farinotti
Piero Fariselli
Christopher Fariss
Robert Fariss
Dávid Farkas
Etelka Farkas
Igor Farkas
Laszlo Farkas
Michael Farkas
Szilvia Farkas
Andrew Farke
Tim Farley
Martin Farlow
Harry Farmer
Melissa Farmer
Richard Farmer
Hanna Farnelid
Omid Farokhzad
Matthew Faron
Asim Farooq
Fedja Farowski
Doug Farquhar
Alex Farr
Celest Farr
Jason Farrah
Katayoun Farrahi
David Farrar
Kerrie Farrar
Mark Farrar
Dorothy Farrar-Edwards
Magí Farré
Alvin Farrel
Colin Farrell
Lara Farrell
Nicholas Farrell
Robin Farrell
Matthew Farrelly
Arodi Farrera
Jaume Farrés
Ken Farrington
Alton Farris
Dominic Farris
Emily Farris
Andrew Farrow
John Farrow
Melissa Farrow
Claude Farrugia
Konstantinos Farsalinos
Soulmaz Fazeli Farsani
Pasquale Farsetti
Amir Farshchi
Mohammad Hosein Farzaei
Parviz Fasahat
Silvia Fasano
Olufunke Fasawe
Peter Fasching
Martin Fascione
Majid Fasihi Harandi
Ole Bernt Fasmer
Luciano Fasotti
Ronnie Fass
Matteo Fassan
Klaus Fassbender
Ambrogio Fassina
Patricia Fast
Maria Chiara Fastame
Marc Fatar
Melissa Fath
Fadi Fathallah
Mahdi Fathizadeh
Cherine Fathy
Razia Fatima
Jaume Fatjo
Mar Fatjó-Vilas
Timur Fatkhudinov
Diane Fatkin
George Fatseas
Quek Fatt
Gisel Fattore
Lanfranco Fattorini
Roberto Fattorusso
Sarah Faubel
Sébastien Faucher
Jérémy Fauconnier
Oliver Faude
Eric Faudry
Christian Faul
Emily Faulconer
Craig Faulds
Martin Faulhaber
Leanne Faulks
Christina Faull
Olivia Faull
Victor Faundez
Denis Faure
Régis Faure
Maria Faurholt-Jepsen
Cyril Fauriat
Bart Fauser
Sascha Fauser

Susanne Fauser
Maria Simonetta Faussone-Pellegrini
Helena Faust
James Faust
Karoline Faust
Michel Fausther
E. Vincent S. Faustino
Maria Amparo F. Faustino Faustino
Massimiliano Faustino
François Fauteux
John Fauth
Michael Fautsch
Maarten Fauvart
Dario Fauza
Cristiano Fava
Giammarco Fava
Luca Fava
Emmanuel Favaloro
Graziella Favarato
Livio Favaro
Francesco Favaron
Marcelo Faveri
Gaia Favero
Isabella Favia
François Favier
Oleg Favorov
Colin Favret
Amani Fawzi
Nicolas Fawzi
Adam Fay
Fabienne Fay
Michael Fay
Phillip Fay
Zahi Fayad
Alan Fayaz
Kiavash Fayyaz
Franz Fazekas
Mina Fazel
Seena Fazel
Massimo Fazio
Nicola Fazio
Linda Feagins
Mark Fear
Rachel Fearns
Philip Fearnside
Richard Fearon
David Feary
Nicholas Feasey
Robin Featherstone
Henry Fechner
Neal Fedarko
David Fedele
Giorgio Fedele
Katya Feder
Lisa Federer
Brian Federici
Alessandro Federico
Peter Federolf
Frederik Federspiel
Stacey Fedewa
Inna Fedorenko
Victor Fedorenko
Alexei Fedorov
Dmitry Fedosov
Jose Maria Fedriani
Pawel Fedurek
Richard Feelders
Brian Feeley
Ann Feeney
Mary Feeney
Alan Feest
Gregory Fegan
Michael Fehlings
Jill Fehrenbacher
Lars Fehren-Schmitz
Juntao Fei
Kezhen Fei
Khang Tsung Fei
Qiang Fei
Shui-Zhang Fei
Zheng-Hua Fei
D.S. Feig
Matthias Feige
Li Fei-Ka
Katharina Feil
Robert Feil
Irwin Feinberg
Mark Feinberg
Wiebke Feindt
Ludwig Feinendegen
Alan Feingold
Justin Feinstein
Adam Feist
Gregory J. Feist
Ten Feizi
Laura Fejerman
Thomas Fekete
Anis Feki
Barbara Felber
Jordan Feld
Paul Feldblum
Vincent Felde

Robin Felder
Marita Feldkaemper
Mark Feldlaufer
Andrew Feldman
Charles Feldman
Heidi Feldman
Inna Feldman
Lauren Feldman
Marcus Feldman
Richard Feldman
Steven Feldman
Yuri Feldman
Ester Feldmesser
Dirk Feldmeyer
Leora Feldstein
Jennifer Felger
Martin Felices
Francesco Felici
Amelia Felipo-Benavent
Paulo Felisberto
Georg Felix
Juan Felix
Leonardo Felix
David Fell
Richard Fell
Christin Fellenberg
Kathryn Feller
Ronald Fellman
Daniel Felmlee
Daniel Fels
Janina Fels
Diane Felsen
David Felson
Marcus Felson
Barbara T. Felt
Julia Felton
Pedro Femia Marzo
Grazia Daniela Femminella
Yap Wing Fen
Klaus Fendler
Sarah-Maria Fendt
Bang Feng
Bin Feng
Bo Feng
Chao Feng
Chaolu Feng
Chenglian Feng
Congjing Feng
Dai Feng
Deshun Feng
Gu Feng
Gui-Yu Feng
Hai-Zhong Feng
Hua Feng
Hualong Feng
Hui Feng
Jiachun Feng
Jian Feng
Jiang-Hua Feng
Jing Feng
Jin-Liang Feng
Jun Feng
Li Feng
Ling Feng
Li-Ping Feng
Mingqian Feng
Ninghan Feng
Peipei Feng
Qianjin Feng
Tao Feng
Tingyong Feng
Wenfeng Feng
Wen-Hai Feng
Wenyi Feng
Xi Feng
Xianzhong Feng
Xiao Feng
Xin Feng
Xing Feng
Xing Lin Feng
Yanmin Feng
Yaoyu Feng
Yi Feng
Yibin Feng
Yuan Feng
Yue-Mei Feng
Yumei Feng
Yu-Mei Feng
Zeny Feng
Zhixin Feng
Zhuo Feng
Zongdi Feng
Hongli Feng Hennessy
Deborah Fenlon
Michael Fenn
Katja Fennel
Lukas Fenner
Peter Fennessy
J. Fenno
Ama Fenny
Ivana Fenoglio
Maria Fenoglio

Albert J Fenoy
Rod Fensham
James Fenske
Aaron Fenster
Martje Fentener van Vlissingen
Owen Fenton
Francesco Feo
Robert Feranec
Mourad Ferdaoussi
Camille Ferdenzi-Lemaitre
Peter Ferdinandy
David A Ferenbach
Alberto Fereres
Seyed-Mohammad Fereshtehnejad
Shingairai Feresu
Paul Fergus
Tom Fergus
Adam Ferguson
Alyssa Ferguson
Christopher Ferguson
David Ferguson
Deborah Ferguson
Eilidh Ferguson
Elaine Ferguson
Mark Ferguson
Patricia Ferguson
Stephen Ferguson
Susan Ferguson
Malcolm Ferguson-Smith
Peter Ferket
Russell Ferland
Edoardo Ferlazzo
Roberto Fernadez-Lafuente
Pierre Olivier Fernagut
Adelaide Fernandes
Alexandra R Fernandes
Brisa Fernandes
Carla F.C. Fernandes
Carlos Fernandes
Cristina Fernandes
Elizabeth Fernandes
Fabio Fernandes
Fátima Fernandes
Flavio Fernandes
Hugo Fernandes
Isabel Fernandes
Janaina Fernandes
Kristianne Fernandes
Natalia Fernandes
Paulo Fernandes
Pedro Fernandes
Sabrina Fernandes
Stenio Fernandes
Tarcísio Fernandes
Veronica Fernandes
Maria João Fernandes Martins
Fernando Fernandes Paiva
Agustin Fernandez
Ana I. Fernandez
Antonio Fernandez
Celine Fernandez
Christopher Fernandez
Ephrem Fernandez
Esteve Fernandez
Isis Fernandez
Jose Fernandez
Jose-Jesus Fernandez
Julian Fernandez
Manuel Fernandez
Maria-Luz Fernandez
Matthew Fernandez
Pedro Fernandez
Soledad Fernandez
Thalia Fernandez
Enrique Fernández
Javier Fernández
Jose Fernández
Luis Ángel Fernández
Manuel Fernández
O. Fernández
Victoria Fernández
Juan Miguel Fernandez Alvira
Francisco Fernández de Miguel
Eva Fernández Domínguez
Fernando Fernández-Aranda
Daniel Fernandez-Ayala
Gregorio Fernandez-Ballester
Rocio Fernández-Ballesteros
Maite Fernández-Barrena
Zahira Fernandez-Bedmar
M. Milagro Fernández-Carrobles
Emilio Fernandez-Espejo
Elisabet Fernández-García
Marta Fernández-García
Rodrigo Fernandez-Gonzalez
Anabel Fernandez-Iglesias
Jose A. Fernandez-Leon
Jose Fernandez-Luna
Gloria Fernandez-Mayoralas
Diego Fernandez-Novoa
Cristina Fernández-Portero
Eduardo Fernandez-Rebollo

Juan Fernandez-Recio
Javier Fernandez-Ruiz
Antonio Fernández-Ruiz
Mario Fernández-Ruiz
Manuel Fernández-Sánchez
María José Fernández-Serrano
Ana Fernandez-Sesma
Enrique Fernández-Taboada
Jose Fernandez-Triana
Leonardo Fernandino
Alda Fernando
Anne Fernando
Narmada Fernando
Nimesha Fernando
Philip Fernbach
Bo Fernhall
David Fernig
Johan Ferno
Gordon Ferns
Philip Fernside
Jack Ferracane
Giovanna Ferraioli
Clotilde Ferrandiz
Cristina Ferrandiz
Dominique Ferrandon
Flavia Ferrantelli
Emilio Ferrara
Lisa Ferrara
Napoleone Ferrara
Santo Davide Ferrara
Cleber Ferraresi
Bucalen Ferrari
Fiorenza Ferrari
Merari Ferrari
Paola Ferrari
Renata Ferrari
Simone Ferrari
Chiara Ferrario
Ronaldo Ferraris
Angelo Ferraro
Manuel Ferraro
Pietro Manuel Ferraro
Stefania Ferraro
Enrico Ferrazzi
Juan Ferré
Fabrizio Ferrè
Catterina Ferreccio
A.C.F. Ferreira
Adaliene Ferreira
Andrêa Jacqueline Ferreira
Artur Ferreira
Bianca Ferreira
Clelia Ferreira
Daniela Ferreira
Frederico Ferreira
Helena Ferreira
Joaquim Ferreira
Jorge Ferreira
Juliano Ferreira
Julio Cesar Ferreira
Leonardo Ferreira
Luana Ferreira
Ludmila Ferreira
Marcelo Ferreira
Mónica Ferreira
Paulo Ferreira
Pedro Ferreira
Rodrigo Ferreira
Sonia Ferreira
Victor Ferreira
Zulma Ferreira
Cristiana Ferreira Alves de Brito
Marcelo Ferreira da Costa Gomes
Silvio Ferreira Jr.
Patricia Ferreira Monticelli
Graça Maria Ferreira-Dias
Albert Ferrer
Mercedes Ferrer
Miguel Ferrer
Jose Ferreras
Andres Ferreri
Laura Ferreri
Andrea Ferrero
Victoria Ferrero
Elisabetta Ferretti
Fabrizio Ferretti
Francesco Ferretti
Gilbert Ferretti
Maria Teresa Ferretti
Stefano Ferretti
Claudio Ferri
Cleusa Ferri
Evelyn Ferri
Lorenzo Ferri
Marica Ferri
Nicola Ferri
Jane Ferrie
Gary Ferrier
John Ferrier
Maite Ferrin
Juan Pedro Ferrio
Martin Ferris

Michael Ferris
Patrick Ferris
Steven Ferris
Albert Ferro
Ilario Ferrocino
Barbara Ferry
James Ferry
Andrzej Fertala
Charles Ferte
Elana Fertig
Daniel Ferullo
Gagan Fervaha
Jean-François Ferveur
Arman Fesharaki
Joshua Fessel
Andrea Feßler
Richard Festa
Richard Festenstein
Aramde Fetene
Sergueï Fetissov
Roberto Feuda
Giora Feuerstein
Jean-Paul Feugeas
Marc Feuilloley
Jamie Feusner
Paul Feustel
Davina Fevery
Rachel Fewster
Clare Fewtrell
Paul Fey
Eric Feyfant
Tesfaye Feyissa
Laura Feyrer
Enrico Fiaccadori
Christian Fiala
Michael Fialkow
Marie Kainoa Fialkowski
Wim Fias
Stefania Ficarella
Jakub Fichna
Marta Fichna
Raina Fichorova
Regis Fichot
Yann Fichou
Thomas Ficht
Thomas A. Ficht
Claudia Fichtel
Harlan Fichtenholtz
Andreas Fichter
Andrea Ficke
Darren Ficklin
Ebru Fidan
Paul Fidel Jr.
Antje Fiebig
Lena Fiebig
Klaus Fiedler
Konrad Fiedler
Paul Fiedler
Sebastian Fiedler
Walter Fiedler
J. Fiehler
Jens Fiehler
Katja Fiehler
Oliver Fiehn
Daniel Field
David Field
Gregory Field
James Field
John Field
Joshua Field
Judith Field
Kenneth Field
Martha Field
Martin Field
Matthew Field
Vanessa Field
James Fielding
Kelly S Fielding
Richard Fielding
Aaron Fields
David Fields
Gregg Fields
Kenneth Fields
Melanie Fields
Ryan Fields
Els Fieremans
Steven Fiering
Olga Fierro
Rainer Fietkau
Daniela Fietz
Ingo Fietze
Urban Fietzek
Virgil Fievet
Brian Fife
Bruno Figadare
Marc Figge
Michele Fighera
Michela Figorilli
Will Figueira
Maria Helena Figueiral
Francisco Figueiras
Agnes Marie Figueiredo

Ana Elizabeth Figueiredo
Ana Sofia Figueiredo
Ceu Figueiredo
Gustavo Figueiredo
Hugo Figueiredo
Ricardo Figueiredo
Francesc Figueras
Maria-Jose Figueras
Alvaro Figueroa
Arturo Figueroa
Carmen Figueroa
Jonine Figueroa
Rosa Figueroa
Hans Figulla
Andrea Figus
Sabina Fijan
Antonio Filareto
Francesca Filbey
Andrew Filby
János Filep
Clark Files
Ramon Filgueira
Bruno Filgueiras
Claudio Filgueiras
Edson Filho
Moacir Godinho Filho
Melanie Filiatrault
Francesco Filiciotto
Nicoletta Filigheddu
Kristian Filion
Szymon Filip
Paulo Filipe
Sergio Filipe
Robert K. Filipkowski
Joanna Filipowska
Gianluca Filippa
Gerasimos Filippatos
Christopher Filippi
Massimo Filippi
Roberto Filippi
Filippos T. Filippidis
Antonio Filippin
Nicola Filippini
Reno Filippo
Alan Filipski
Helena Filipsson
Alessandro Filla
María Fillat
Guido Filler
Roger Fillingim
Myriam Fillion
Sophie Fillon
Mirella Filocamo
Marcel Filoche
Giuseppe Filomeni
Aldo Filosa
Jessica Filosa
Stefania Filosa
Elise Filotas
Jacky Fils
David Filsoof
Katharina Filz
Dario Finazzi
Robert Finberg
Courtney Finch
Tom Finch
W. H. Finch
Brian Finck
Mark Findeis
John Findlay
Oliver Findling
Daniel Fine
Leon Fine
Philippe Fines
Vittorio Fineschi
Robert Finger
Barbara Fingleton
Chiara Fini
Lorenzo Fini
M. Elizabeth Fini
Michael Finiguerra
Astrid Fink
Katrin Fink
Linda Fink
Steven Finkbeiner
Susan Finkbeiner
Deborah Finke
Stefan Finke
Adam Finkel
Meir Finkel
Richard Finkel
Alexei Finkelstein
Eric Finkelstein
Jonathan Finlay
Kathleen Finlayson
Tracy Finlayson
Anna Finley
Dan Finley
James Finley
Kim Finley
Lucinda Finley
Russell Finley Jr.

Emily Finn
Roderick Finn
Anna Finnane
Daniel Finnegan
Silvia Finnemann
Kevin Finneran
Nanna Finnerup
Taija Finni
Mikko Finnilä
Sarah Finocchario Kessler
Paola Finoia
Kai Finster
Josef Finsterer
Csaba Finta
Andres Finzi
Alexandra Fiocco
Diego Fiol
Antonella Fioravanti
Gianfranco Fiore
Nicola Fiore
Andrew Fiore-Gartland
Fabio Fiorentino
Francesca Fiorentino
Niccolo Fiorentino
Teresa Vanessa Fiorentino
Luca Fiorenza
Lisa Fiorini
Romina Fiorotto
Francesca Fiory
Djordje Fira
Chaz Firestone
M. Elizabeth Firestone
R. Firestone
Rebecca Firestone
Cyril Firmat
Jennifer Firn
Arnaud Firon
Eric First
Jill Firszt
Louise Firth
Claudia Fischbach
Jorge Fischbarg
Alexandra Fischer
Andreas Fischer
Andrew Fischer
Andy Fischer
Annegret Fischer
Asja Fischer
Bernard Fischer
Bernd Fischer
Dagmar Fischer
David Fischer
Edward Fischer
Gabriela Fischer
Gilles Fischer
Hans-Georg Fischer
Heinz Fischer
Itzhak Fischer
Joan Fischer
Julia Fischer
Kathleen Fischer
Katja Fischer
Lutz Fischer
Mareike Fischer
Martin Fischer
Peter Fischer
Robert Fischer
Roman Fischer
Ulrike Fischer
Urs Fischer
Valentin Fischer
William Fischer
Wolfgang Fischer
Celine Fischer Fumeaux
Michael Fischereder
Tracy Fischer-Smith
Matthew Fischl
Martin Fischlechner
Gustavo E. Fischman
Florian Ph.S Fischmeister
Daniel Fiset
Frank Fish
Jason Fish
Melissa Fishel
Aron Fisher
B.A. Fisher
Celia Fisher
Christopher Fisher
Claire Fisher
Colin Fisher
Daniel Fisher
Delbert Fisher
Derek Fisher
Eric Fisher
Helen Fisher
James Fisher
Jane Fisher
John Fisher
Kevin Fisher
Mandy Fisher
Mark Fisher
Nick Fisher

Ryan Fisher
Steve Fisher
Steven Fisher
Simon Fisher-Baum
Ayelet Fishman
Inna Fishman
Mayer Fishman
Veniamin Fishman
Amy Fiske
Ane Fisker
Tecumseh Fitch
Julie Fitness
Nahuel Fittipaldi
Susan Fitzer
Dan Fitzgerald
Denise Fitzgerald
Duncan FitzGerald
Edward Fitzgerald
Felicity Fitzgerald
Lee Fitzgerald
Malinda Fitzgerald
Rebecca Fitzgerald
T.J. Fitzgerald
Una FitzGerald
Gary Fitzhugh
Anne Fitzpatrick
Annette Fitzpatrick
Ben Fitzpatrick
Courtney Fitzpatrick
Elizabeth Fitzpatrick
Lindsay Fitzpatrick
Carolyn Fitzsimmons
Ferdinando Fiumara
Giuseppe Fiume
Elizabeth Fixman
Jon Fjeldså
Hans Flaatten
Alicia Flach
Peter Flach
Andreas Flache
Vincent Flacher
Peter Flachsbart
Adrien Flahault
Scott Flamm
Josef Flammer
John M. Flanagan
Katie Flanagan
Tatiana Flanagan
Patrick Flandrin
Timothy Flanigan
Wesley Flannery
Jason Flannick
Mike Flannigan
C. Flask
Magne Arve Flaten
Kerstin Flath
Thomas Flatt
Valerie Flax
Abraham Flaxman
Stuart Flechner
Marion Flechtner-Mors
James Fleckenstein
Paul Flecknell
James Fleet
Lena Fleig
Shelby Fleischer
David Fleischman
Katrin Fleischman
Achim Fleischmann
Bernd Fleischmann
Dominik Fleischmann
Bobbi Fleiss
Adam Fleming
Alison Fleming
Alyson Fleming
David Fleming
Emily J. Fleming
Ingrid Fleming
Jodie Fleming
Marc Fleming
Paul Fleming
Robert Fleming
Roland Fleming
Sherry Fleming
Timothy Fleming
Hans-Curt Flemming
J.A. Flemming
Vicki Flenady
Michelle Flenniken
Michael Flessner
Anne Fletcher
Hansel Fletcher
Helen Fletcher
Jacqueline Fletcher
Jamie Fletcher
Jared Fletcher
Louise Fletcher
Reginald Fletcher
Robert Fletcher Jr.
Gordon Flett
Marie-Josée Fleury
Laurence Flevaud

Diego Flichman
Leon Flicker
Antje Flieger
Ulrike Flierl
Elvira Flikweert
Harry Flint
Paul Flint
S. Flint
Steve Flint
Krzysztof Flisikowski
Ismail Fliss
Michael Flister
Sabine Flitsch
Kevin Floate
Aaron Floden
Lucile Floeter-Winter
Ulrich Flögel
Camilla Fløjgaard
Phyllis Flomenberg
Pamela Flood
Paulette Flore
Andrei George Florea
Andreas Floren
Alexander T. Florence
Yuk Lin Florence Lai
Margarita Florencio
Carlos Flores
Elsa Flores
Enrique Flores
Julio Flores
Laura Flores
Liubov Flores
Oscar Flores
Eugenia Flores Figueroa
Carlos Flores-Mir
Habacuc Flores-Moreno
Igor Florez-Sarasa
Oscar Florez-Vargas
Laurence Flori
Wegwitz Florian
Belén Floriano
Chris Florides
Monica Florin-Christensen
João Batista Florindo
Tullio Florio
Ignazio Floris
Ilaria Floris
Esther Florsheim
Jean-Francois Flot
Terrance Flotte
Terry Flotte
Charles-Henri Flouzat-Lachaniette
Christopher Flowers
Jonathan Flowers
Paul Flowers
Scott Floyd
Martin Flück
Christoph Flückiger
Joelle Flueck
Tyrel Flügel
Robert Fluhr
Ad Fluit
Stephen Flusberg
Joel Fluss
Aaron Flynn
Charles Flynn
Kevin Flynn
Lawrence J. Flynn
J.E. Flythe
Michael Flythe
Andrea Foassati
Samah Fodeh
Stelios Fodelianakis
Daniela Foell
Alex Foerster
Carola Foerster
Manuel Foerster
Andrew Foey
Ismael Fofana
Mariam Fofana
Ilenia Foffa
Andréa Fogaça
Paolo Fogagnolo
Linda Fogarty
Nicole Fogarty
Patrick Fogarty
S. Fogarty
Bernhad Föger
Bruno Foggi
Agnes Fogo
Federico Fogolari
Marta Fogolari
Debora Foguel
Svenja Fohler
Lane Foil
Nicolas Foin
Pak-Wing Fok
Joseph Fokam
Alejandra Folco
Hernan Folco
Brian Foley
Edan Foley

Joe Foley
Melissa Foley
Steven Foley
Laura Folgori
Andres Folguera
Les Folio
James Folk
Gert Folkers
Antonia Follenzi
Veronica Folliero
Matilde Follo
Eelke Folmer
Heike Folsch
Lindy-Lee Folscher
Piotr Foltynski
David Foltz
Inge Fomsgaard
Jannik Fonager
Irina Fonareva
Constantino Fondevila
Manuel Fondevila
Mun Yik Fong
Laura Fonken
Virginia Fonner
Marko Fonovic
Valentina Fonsato
César Fonseca
Joao Fonseca
João Fonseca
Nuno Fonseca
Simone Fonseca
Célia Fonseca Guerra
Albert Font
S. Fontagne
Nathalie Fontaine
Andrea Fontana
Jake Fontana
Margherita Fontana
Marianna Fontana
Simona Fontana
Chiara Fontanella
Luca Fontanesi
Annick Fontbonne
Francesc Font-Clos
Pedro Fonte
Patricia Fontela
Cor Jesus Fontes
Viviana Fonti
Cilius Fonvig
Caroline Fonzo-Christe
Jonas Fooken
David Fooksman
Jasper Foolen
Kelly Foote
Robert Foottit
Ivo Foppa
Randi Foraker
Andrew Forbes
Miriam Forbes
Scott Forbes
Joseph Forbi
Frank Forcella
Christelle Forcet
Karl Forchhammer
Amanda Ford
Brett Ford
Dianne Ford
Elizabeth Ford
Jane Ford
Julian Ford
Kathleen Ford
Mark Ford
N.J. Ford
Nathan Ford
Robert Fordyce
Bo Foreman
Kyle Foreman
Carlo Foresta
Cristian Forestan
Germain Forestier
Patrice Forget
Katrien Forier
Marie-Amélie Forin-Wiart
Matthias Forkel
Nils Daniel Forkert
Thomas Forkmann
Maria Forlenza
Gianluigi Forloni
Dorota Formanowicz
Rita Formisano
Cécile Formosa
Pau Formosa-Jordan
Alice Fornari
Lorenzo Fornaro
Marco Fornazieri
Astrid Fornek
Anna Fornell
Alejandro Forner
Federico Forneris
Lui Forni
Maria Fernanda Forni
Erick Forno

Alena Fornusková
Francesco Foroni
Robert Foronjy
Serhiy Forostyak
Afra Foroud
Lucy Forrest
John Forrester
Stephanie Forrester
Bertil Forsberg
Karin Forsberg-Nilsson
Carol Forsblom
Kirsty Forsdike-Young
Zac Forsman
Maria Forsner
John Forster
Paul Forster
Samuel Forster
Asta Försti
Michael Forsting
Wolfgang Forstmeier
Elisabet Forsum
David Forsyth
Stephen Forsythe
Patrice Fort
Carlos Magno Castelo Branco Fortaleza
Emanuele Forte
Roberta Forte
Juan Fortea
Mikael Fortelius
Francesca Fortenbaugh
James Fortenberry
Patrick Forterre
Zuleica Fortes
Donald Forthal
Lucas Forti
Anne-Marie Fortier
Julien Fortier
Michelle Fortier
Carole Fortin
Daniel Fortin
Elise Fortin
Marie-Josee Fortin
Caroline Fortunato
John Fortunato
Rodrigo Fortunato
Brad Fortune
Eric Fortune
Constanza Fosco
Catherine Foss
Kari D. Foss
Silvia Fossati
Bethany Foster
Byron Foster
Carl Foster
Charles Foster
Derek Foster
Diana Foster
Emma Foster
Gary Foster
Gigi Foster
Glen Foster
Jamie Foster
Jane Foster
John Foster
Leonard Foster
Michelle Foster
Morris Foster
Paul Foster
Simon Foster
Steven Foster
Timothy Foster
Tina Foster
Vania Foster
William Foster
Maria Foti
Anastasios Fotiou
Vasileios Fotopoulos
Akbar Fotouhi
Ashraf Fouad
Fetnat Fouad-Tarazi
Jerome Foucaud
David Fouchet
Nazanin Fouladgar
Paul Foulkes
Marie Foulongne-Oriol
Ilias Fountalis
Elise Fouquerel
Yoan Fourcade
Nicolas Fourcaud-Trocme
Denis Fourches
Alexandre Fouré
Willem Fourie
Dominique Fourmy
Isabelle Fournel
Marcia V. Fournier
Philippe Fournier-Viger
Claire Fourrey
Jean-Baptiste Fourvel
Sotirios Fouzas
Freya Fowkes
Robert Fowkes
Amy Fowler

Ashley Fowler
Christie Fowler
John Fowler
Sharon Fowler
Veronica Fowler
Kasey Fowler-Finn
Andrew Fox
Barry Fox
Caroline Fox
Clive Fox
David Fox
Edward Fox
Elaine Fox
Elizabeth Fox
James Fox
James Alan Fox
Jay Fox
Jesse Fox
Karin Fox
Kathryn Fox
Keith Fox
Mary Fox
Michael Fox
Molly Fox
Rebecca Fox
Robert Fox
Simon Fox
Betsy Foxman
Christine Foyer
John Fozard
Elizabeth Fozo
Claudio Fozza
Marco Fraaije
Letizia Fracchia
Karl Frafjord
Deborah Fraga
Helder Fraga
Tatiana Fraga
Gabriele Fragasso
Dorothy Fragaszy
Sotirios Fragkostefanakis
Laura Frago
Rodrigo Rocha Fragoso
Yara Fragoso
Jimmy Fraigne
André Frainer
Gail Fraizer
David Frakes
Alessandro Fraldi
Gregory Fraley
Leigh Frame
Jason Franasiak
Nathalie Franc
Suzelei Franca
Filipe França
Marcondes França Jr.
Paolo Francalacci
Rubén Francés
Silvana Franceschetti
Francesco Franceschi
Massimo Franceschi
Guido Franceschini
Nora Franceschini
Sandro Franceschini
Harold Franch
Federico Franchi
Kleber Franchini
Massimo Franchini
Gianluigi Franci
Enrico Francia
Piergiorgio Francia
Ronaldo Francini-Filho
M. Pilar Francino
Diego Franciotta
Ashwanth Francis
David Francis
Frédéric Francis
Gregory Francis
Joseph Francis
Matthew Francis
Stephen Francis
Alex Francisco
Mercival Francisco
Sara Francisco
Robert Franciscus
Patricia Francis-Lyon
Mike Francke
Stephan Francke
Uta Francke
Andre Franco
Augusto Franco
Claudio Franco
E. Franco
Israel Franco
Jorge Franco
Jose Franco
José Carlos Franco
Lara Franco
Nuno Henrique Franco
Rafael Franco
Valentina Franco
Walfre Franco

Carlos Franco Abuín
José Franco Junior
Janusz Franco-Barraza
Carre Francois
Jean Francois
Patrice Francois
Achille François
Bruno François
Clément François
Valentina Franco-Trecu
Eric Francotte
Tiago Francoy
Tove Frandsen
Jean-François Franetich
Dimitrios Frangoulidis
Arthur Frank
Cornelia Frank
Greg Frank
Joachim Frank
Morgan Frank
Philippe Frank
Richard Frank
Robert Frank
Saša Frank
Frank Drygala Frank Drygala
Andreas Franke
Katja Franke
Leonora Franke
Molly Franke
Werner Franke
Paul Frankel
Victor Frankel
Philipp Franken
Silja Frankenbach
Christian Frankenberg
Frances Frankenburg
Cara Frankenfeld
Julia Frankenstein
Greta Frankham
Piotr Frankiewicz
M.D. Frank-Kamenetskii
Paul Frankland
Bernardo Franklin
Craig Franklin
Erik Franklin
Gary Franklin
Jeremy Franklin
Jessica Franklin
Peter Franklin
Richard Franklin
Scott Franklin
Teresa Franklin
Zoë Franklin
Thomas Frankovich
Stephen Franks
Andrew Frank-Wilson
Charles Fransen
Per-Anders Fransson
Patrick Frantom
Leonid Frantsevich
Kyle Frantz
Maria Frantzi
David Franz
Eelco Franz
Steffen Franz
Silvia Franzellitti
Daniel Franzen
David Franzen
Oscar Franzen
Giancarlo Franzese
Stefan Fränzle
Michelle Franz-Montan
Pierre-Olivier Frappart
Mirella Fraquelli
Mattia Frasca
Marianna Frascarelli
Giada Frascaroli
Simonetta Fraschetti
Alan Fraser
Bill Fraser
Clarissa Fraser
Danielle Fraser
Erin Fraser
James Fraser
Jennifer Fraser
Katharine Fraser
Robert Fraser
Tim Fraser
Nicole Fraser-Hurt
Kaitlin Frasier
Johannes Frasnelli
Michael Frass
Matteo Frasson
Filip Fratev
Antonio Fratini
Sara Fratini
Jamie Fratkin
Esteban Frauca
Thomas Frauenfelder
Sally Frautschy
Richard Frayne
Amy Frazier

Thomas Frazier
Silvia Fre
Emilie Frealle
Lenin Fred
Bruno Frederich
Renata Frederico
Elizabeth Fredheim
Merete Fredholm
David W. Fredriksson
Palle Fredsted
Stephen Free
Kenneth Freedland
David Freedman
Jane Freedman
Jonathan H. Freedman
Leonard Freedman
Lori Freedman
Neal Freedman
Sara Freedman
Suzanne Freedman
Benjamin Freeman
Christine Freeman
Claire Freeman
Hani Freeman
Jane Freeman
Joshua Freeman
Mary Freeman
Michael Freeman
Spencer Freeman
Steven Freeman
Sylvie Freeman
Willard Freeman
Nick Freemantle
Michael Freemark
Anthony Freemont
Nicole Freene
Mark Freestone
Christopher Freet
Rosa Fregel
B.J. Fregly
José Fregnani
Ralph Fregosi
Karin Frei
Michael Frei
Reno Frei
Ursula Frei
Brian Freibaum
Matthew Freiberg
Ellen Freiberger
Sandra Freiberger
Marc Freichel
Richard Freifelder
Jessica Freiherr
Karen Freijer
Amado Freire
Rafael Freire
Jose Freire da Silva Neto
Jozélio Freire de Carvalho
Eva Freisinger
Michael Freissmuth
Martin Freitag
Loreta Freitas
Lucas Freitas
Marcelo Freitas
Maria Eliza Freitas
Matheus Oliveira Freitas
Pedro Freitas
Sandro Freitas
Vanessa Freitas
Thibaut Fréjaville
Lee Frelich
Marie-Laure Frelut
Andrew French
Brent French
Dustin French
Joshua French
Martyn French
Neil French
Paul French
Jean-Benoit Frenette Charron
Cedric Frenette-Dussault
Dan Frenkel
Lisa Frenkel
Victor Frenkel
Matthias Frentzen
Michele Freppaz
Jean-Marie Frère
Luciano Freschi
Paula Fresco
Libera Fresiello
Manuel Fresno
Shane Fresnoza
Vicente Fresquet
Kristine Freude
Jan Freudenberg
John Freudenstein
Bret Freudenthal
Ramiro Freudenthal
Conrad Freuling
Jean-Noël Freund
Patrick Freund
Yonathan Freund

Paul Frewen
Paul A. Frewen
Andreas Frey
Eberhard Frey
Renato Frey
Jan Freyberg
Jörg Freyhof
Saskia Freytag
Daniela Frezzetti
Ana Friães
Sylvie Friant
Jesus Maria Frias Celayeta
Vinicius Frias de Carvalho
Jorge Frias-Lopez
Dolores Frias-Navarro
Eckhard Friauf
Anders Friberg
Magne Friberg
Jean-Chistophe Fricain
Gregory L. Fricchione
Winifred Frick
Florian Fricke
Eva Frickel
Lloyd Fricker
Hagen Frickmann
Alejandro Frid
Daniel Fridberg
Anders Fridberger
Leonid Fridlyand
Rafael Fridman
Bernd Friebe
Hermann Frieboes
Daniel Fried
Eiko Fried
Nathan Fried
Mark Friedberg
Caroline Friedel
Jonathan Friedel
Guilad Friedemann
Andreas Friedl
Claudia Friedl
Ari Friedlaender
Barbara Friedland
Kevin Friedland
Alan Friedlander
Alan Friedman
Alexander Friedman
Alon Friedman
Ariella Friedman
Carol Friedman
Eitan Friedman
Harvey Friedman
Jacob Friedman
Jennifer Friedman
Joseph Friedman
Joshua Friedman
Katherine Friedman
Lloyd Friedman
Ran Friedman
Wilma Friedman
Anne Friedrich
Christina Friedrich
Juliane Friedrich
Markus Friedrich
Max Friedrich
Nele Friedrich
Thorsten Friedrich
David Friel
James Friend
Kyle Friend
Margaret Friend
Gabriel Fries
Lisa Fries
Robert Friesel
Ingrid Friesema
Justin P. Friesen
Marcia Friesen
Robert Friesen
Tim Friesen
Martin Friess
Stuart Friess
Niels-Ulrik Frigaard
Antonio Frigeri
Daniel Frigo
Bertrand Friguet
Nina Friis-Moller
M. Frimer
Andreas Frings
Michael Frink
Gaelle Friocourt
Jean-Pol Frippiat
Ashley Frisch
Steven Frisch
Christian Frischholz
Luke Frishkoff
Laura Frishman
Mike Frisina
Steven Frisson
Brian Fristensky
Karl Friston
Rena Friswell
Xavier Fritel

Chris Frith
James Frith
Peter Fritsch
Lars Fritsche
Matthias Fritsche
Chris Fritton
Sarah Fritts
Heather Fritz
Hermann Fritz
Joelle Fritz
Julie Fritz
Megan Fritz
Stephanie Fritz
Marcus Fritze
Marvin Fritzler
Bernd Fritzsch
Pauline Frizelle
Norma Frizzell
Kerstin Fröber
Michael Froehner
Brett Froelich
Victor Froelicher
Gabriele Froemming
Robert Froese
Michael Frohman
Claudia Frola
Anne Frolich
Andreas Frölich
Andrey Frolov
Roman Frolov
Catherine Fromen
Bastian Fromm
Jörg Frommer
Gerrit Frommeyer
Ingo Frommholz
Edward Frongillo
Zacharias Frontistis
Gilberto Fronza
Jenny Frössling
H. Frost
Jeffrey Frost
Kathy Frost
Paul Frost
Peter Frost
Poul Frost
Stephen Frost
Steven Frost
Johan Frostegard
Jan Frouz
Mayra Frozoni Rebolla
Doriana Fruci
Dominique Frueh
Pedro Fruet
Sascha Frühholz
Sylvia Frühwirth-Schnatter
Rebecca Frum
David Fruman
Davide Frumento
Donald Frush
Jennifer Frustino
Marcus Fruttiger
Mario Fruzangohar
Christopher Fry
Jessica Fry
William Fry
Richard Frye
Victoria Frye
Tim Fryer
Daniel Frynta
Jon Fryzek
Baoquan Fu
Bingmei Fu
Chao Fu
Chi-Yu Fu
Chuanhai Fu
Chun Fu
Daolin Fu
Dongxu Fu
Geng-Feng Fu
Haiyan Fu
Hongtuo Fu
Hongyun Fu
J. Fu
Jian Fu
Jianhua Fu
Jianping Fu
Jie Fu
Jihua Fu
Junjiang Fu
Junzeng Fu
Li Fu
Liping Fu
Li-Wu Fu
Michael Fu
Mingui Fu
Panfeng Fu
Peifen Fu
Qi Fu
Qing-Ling Fu
Sau Nga Fu
Shi-Jian Fu
Shujun Fu

Weiguo Fu
Wenjiang Fu
Wen-Mei Fu
Xiang-Dong Fu
Xiaobing Fu
Xiaoyong Fu
Xiuju Fu
Xiujun Fu
Yi-Ping Fu
Yong-Bi Fu
Yu-Jie Fu
Zhengwei Fu
Zhongjie Fu
Helio Fuchigami
Armin Fuchs
Bruno Fuchs
Dietmar Fuchs
Dirk Fuchs
Florent Fuchs
Heidi Fuchs
Heiko Fuchs
Joerg Fuchs
Judith Fuchs
Julian Fuchs
Marc Fuchs
Peter Fuchs
Stephen Fuchs
Karolina Fucikova
Hans-Peter Fuehrer
Tobias Fuehrmann
Georg Fuellen
Bernard Fuemmeler
Carmen Fuentealba
Rodrigo Fuentealba
Manuel Fuentes
Ramon Fuentes
Eduardo Fuentes-Contreras
Enrique Fuentes-Mattei
César Fuentes-Yaco
Peter Fuerst
Elaine Fuertes
Susanne Fuessel
Laura Fugazzola
Samuel Fuhlendorf
Gwenny Fuhler
Cynthia Fuhrmann
Jeffry Fuhrmann
Kevin Fuji
Nobutaka Fujieda
Junko Fujihara
Yoshitaka Fujihara
Hiroaki Fujii
Hiroshi Fujii
Saori Fujii
Satoshi Fujii
Shin-Ichiro Fujii
Shinya Fujii
Takao Fujii
Takeshi Fujii
Wataru Fujii
Seizo Fujikawa
Ko Fujimori
James Fujimoto
Kiyoshi Fujimoto
Noriki Fujimoto
Kei Fujimura
Masato Fujimura
Yoshihisa Fujino
Keishi Fujio
Takako Fujioka
Ikuko Fujisaki
Daisuke Fujisawa
Tomoyuki Fujisawa
Yuya Fujishima
Hiroshige Fujishiro
André Fujita
Hisashi Fujita
Kazutoshi Fujita
Masatoshi Fujita
Masayuki Fujita
Mayumi Fujita
Naoya Fujita
Ricardo Fujita
Ryo Fujita
Satoshi Fujita
Takeo Fujita
Toshihiko Fujita
Yasuyuki Fujita
Yoshihiko Fujita
Yukihiro Fujita
Kei Fujiwara
Keiichi Fujiwara
Naoto Fujiwara
O. Fujiwara
Tohru Fujiwara
Toru Fujiwara
Yoshi Fujiwara
Mikihiro Fujiya
Tomoko Fujiyuki
So-ichiro Fukada
Masafumi Fukagawa
Kei Fukami

Tamo Fukamizo
Takeshi Fukao
Yoichiro Fukao
Hirotaka Fukasawa
Keita Fukasawa
Masanori Fukazawa
Nobuko Fukino
Reginaldo Fukuchi
Daiju Fukuda
David Fukuda
Hirokazu Fukuda
Naoya Fukuda
Yusuke Fukuda
Shin Fukudo
H. Fukuhara
Sadaaki Fukui
Manabu Fukumoto
Atsuko Fukunaga
Eiichiro Fukusaki
Hideto Fukushi
Shuetsu Fukushi
Ery Fukushima
Kenji Fukushima
Takeshi Fukushima
Takuya Fukushima
Hidekatsu Fukuta
Tsutomu Fukuwatari
Hidenao Fukuyama
Tomoki Fukuyama
Franco Fulciniti
Stephany Fulda
Richard Fulford
Josef Fulka
Richard Fullagar
Miquel Fullana
Douglas Fullen
Clifton David Fuller
Heidi Fuller
Patrick Fuller
Richard Fuller
Thomas Fuller-Rowell
Stephanie Fullerton
Christian Fullgrabe
Tod Fullston
Amy Fulton
David Fulton
Erika Fulton
Mark Fulton
Fabio Fumagalli
Marta Fumagalli
Laura Fumanelli
Claudia Fumarola
Christophe Fumeaux
Tulio Fumian
Tomas Funda
Nicholas Funderburg
Yiliam Fundora
Steve Fung
Thomas Fung
Colin Funk
Birgit Funke
Martha Funnell
Clay Fuqua
John Fuqua
Alexandra C.U. Furch
Andras Furedi
Carole Fureix
Alessandro Furey
Nathan Furey
István Furi
Jennifer Furin
Julio Furlan
Laura I. Furlong
Benjamin Furman
Bradley Furman
Pio Furneri
Andrew Furness
Adrian Furnham
Johannes Fürnkranz
S.A.C. Furquim
Matthias Fürst
Axel Fürstberger
Priscilla Furth
Kengo Furuichi
Takeshi Furuichi
Katsuko Furukawa
Koichi Furukawa
Shinya Furukawa
Takahisa Furukawa
Yutaka Furukawa
Fumikazu Furumi
Yuki Furuse
Ryuichi Furuya
Tetsuya Furuya
Yoichi Furuya
Luis Furuya-Kanamori
Janette Furuzawa-Carballeda
Maria Fusaro
Riccardo Fusaroli
Gabriele Fusco
Giovanni Fusco
Roberta Fusco

Shinji Fushiki
Shinya Fushinobu
Marco Fusi
Florian Fusseis
Daniel Fuster
Ryo Futahashi
Mitsuru Futakuchi
Bruce Futcher
Richard Futrell
Matthias Futschik
Clare Futter
Lisieux Fuzessy
Jackson Fyfe
John Fyfe
Alona Fyshe
Nick Fyson
Diann Gaalema
Ruchi Gaba
Vladimir Gabai
Laurel Gabard-Durnam
Belinda Gabbe
Tim J. Gabbett
Alessandro Gabbiadini
Federica Gabbianelli
Idan Gabdank
Christopher Gabel
Harrison Gabel
Rachel Gabel Shemueli
Chiara Gabellini
Davide Gabellini
Khatia Gabisonia
Christoph Gabler
Nicholas Gabler
Caitlin Gabor
Liane Gabora
Anne Gabory
David Gabriel
Robert Gabriel
Eva Gabrielova
Halina Gabrys
Amanda Gabster
Mathieu Gabut
Alessandra Gabutti
Ratan Gachhui
Frédéric Gachon
Simon Gächter
Márta Gácsi
Hayam Gad
Agata Gadaleta
Francesco Gadaleta
Michele Gadd
Giovanni Gadda
Sumanlatha Gaddam
Subhash Gaddipati
Dana Gaddy
Jennifer Gaddy
Gilles Gadea
Joaquin Gadea
Natalia Gadek
Bart Gadella
Theodorus Gadella Jr.
Niraj Gadhavi
Mayur Gadhikar
Endalamaw Gadisa
Andrea Gaedigk
Peter Gaengler
Beate Gaertner
Holger Gaertner
Roberto Gaetani
Marcia Gaete
William Gaetz
Holly Gaff
Michelle Gaffey
Angelo Gaffo
Mina Gaga
Anastasia Gage
Heather Gage
Phillip Gage
Suzanne Gage
Andrea Gaggioli
Vesna Gagic
Manjusha Gaglani
Michael Gaglia
Sarah Gagliano
Christian Gagliardi
Frank Gagliardi
Stella Gagliardi
Anna Gagliardo
Matteo Gagliolo
Jeffrey Gagne
Jean-Philippe Gagné
Robert Gagnon
Yakir Gagnon
Jorge Gago
John Gahagan
Cormac Gahan
Manuel Gahete
Jacqueline Gai
Yunchao Gai
Thais Gaiad
Virginija Gaigalaite
Carlo A.J.M. Gaillard

Melissa Gaillard
Todd Gaines
Ildar Gainetdinov
Raul Gainetdinov
Kristin Gainey
Sally Gainsbury
Fernanda Gaiotto
Udo Gaipl
Robert Gaiser
Thomas Gaiser
Luis-Alberto Gaitán-Cepeda
Sarah Gaither
Sabrina Gaito
Frédérique Gaits-Iacovoni
Karina Gajardo
Peter Gajdos
Vincent Gajdos
Zdzislaw Gajewski
Ognjen Gajic
Lokesh Gakhar
David Gal
Katya Galactionova
Serge Galam
Roberto Galán
Dennis Galanakis
Jennifer Galanis
Annia Galano
Joshua Galanter
Omar Galárraga
Matthew Galaska
Bianca Galateanu
Gabriela Galateanu
Giovanni Galati
Sue Galatowitsch
J.P. Galaud
Martyna Galazka
Alexander Galazyuk
David Galbraith
Maria Rosaria Galdiero
Stefania Galdiero
Henrique Galdino
Tarcísio Galdino
Daniel Gale
Tim Gale
Jerome Galea
Joseph Galea
Bennett Geoff Galef
James Galen
Luke W. Galen
Henrike Galenkamp
Aldo Galeone
Caroline Galeotti
Mauro Galetti
Valeria Galetti
Luciana Galetto
Hanga Galfalvy
Giovanni Galfano
Mario Galgani
Anne Galgon
Richard Galgon
Laura Galian
Robert Galiano
Pierre Galichon
Luis Galietta
Andrea Galimberti
Hubert Galinat
Cristi Galindo
Máximo Ibo Galindo
Rene Galindo
Jorge Galindo-Villegas
Jayne Galinsky
Antony Galione
Gennaro Galizia
Matteo Galizzi
Alexander Galkin
Michael Galko
Cory Gall
Hans-Joachim Galla
Tobias Galla
Márcia Gallacci
Austin Gallagher
Christopher Gallagher
M. Kate Gallagher
Phillip Gallagher
Romayne Gallagher
Ryan Gallagher
Suzanne Gallagher
Daniel Gallaher
Manon Galland
Eduard Gallardo
Rodrigo Gallardo
Jason Gallas
Eugen Gallasch
Robert Galle
Monica Galleano
Francisco Gallego
Juan Álvaro Gallego
Autumn Gallegos
Maria-Trinidad Gallegos
Claudia Gallert
Rachel Gallery
Craig Gallet

Roberta Galletti
Helen Galley
Elena Galli
Luisa Galli
Manela Galli
Stephen Galli
Fernando Galliari
G. Gallicano
Daniel Gallie
Arianna Galliera
James Galligan
Pasquale Gallina
Bill Gallo
Carmen Gallo
Dahiana Gallo
Daniela Gallo
James Gallo
Jean-Marc Gallo
Pedro Gallo
Richard Gallo
Stephen Gallo
Yann Gallot
Riccardo Gallotti
Nathan Galloway
Gordon Gallup
Silvano Gallus
David Gally
Fabienne Gally
Alessandra Galmonte
Brook Galna
Benjamin Galper
Jacob Galson
Ben Galuardi
Moses Galukande
M. Paz Galupo
Sebastian Galuska
Geraud Galvaing
Tais Galvao
Klibs Galvão
Ismael Galve-Roperh
Veronica Galvez
Sergio Gálvez
Karyn Galvin
Petrik Galvosas
Anne Galy
Edouard Galyov
Adelina Gama
José Gama
Antonio Gama Rodrigues
Miguel Gama Sosa
Margarida Gama-Carvalho
Pascal Gamas
Cristina Gamba
Gerardo Gamba
Roberto Gambari
Giulio Gambarota
Maria Cristina Gambetta
Jasvinder Gambhir
Elisa Gambini
Juan Gambini
T. Chris Gamblin
Jorge Gamboa
Michael Gamborg
Michael Gamcsik
Frances Game
Sofia Gameiro
Anna Gamell
Sofia Gameriro
Daniel Gamermann
Kenneth Games
Laurence Gamet-Payrastre
Andrzej Gamian
Sofia Gamito
Eyal Gamliel
David Gamm
Sarah Gammage
Stephen Gammie
Hilary Gammill
John Gamon
Hannes Gamper
Sonja Gamsjaeger
Guojun Gan
Rui Gan
Wenqi Gan
Yong Gan
Kamel Gana
Sabha Ganai
Arsheed Ganaie
Vadivel Ganapathy
Bela Ganatra
Fabio Ganazzoli
Sofiya Gancheva
Maria Ganczak
Virginie Gandemer
Fabian Gander
Ravi Kumar Gandham
Chandrashekhar Gandhi
Hardik Gandhi
Maher Gandhi
Manisha Gandhi
Monica Gandhi
Sagar Gandhi

Shekar Gandhi
Tapan Gandhi
Edson Gandiwa
Robin Gandley
Raffaella Gandolfi
Marta Gandolla
Sylvain Gandon
Sheetal Gandotra
Pandu Gandula
Anand Ganesan
Latha Ganesan
Vidyaramanan Ganesan
Kasturi Ganesh
Shonraj Ganeshrao
Harsha Ganga
Dharanesh Gangaiah
Raman Gangakhedkar
Arunakumar Gangaplara
Rajashekhar Gangaraju
Daniel Ganger
Llilianne Ganges
Omkaram Gangisetty
Umesh Gangishetti
Bhushan Gangrade
Abantika Ganguly
Koustav Ganguly
Nirmal Kumar Ganguly
Tridib Ganguly
Laxman Gangwani
Alexander Ganin
Douglas Ganini da Silva
Davood Ganji
Ramesh Ganju
Colleen Ganley
Peter Gann
Sreenivas Gannavaram
Damon Gannon
Lisa Gannon
Maureen Gannon
Victor Gannon
Ioannis Ganopoulos
Ziv Gan-Or
Christos Ganos
Fraide Ganotice
Hayley Gans
Lars Gansel
Claudia Ganser
David Gansler
Carolina Ganss
John Gant
Timothy Gant
Michael Gantier
Daniel Ganu
Vitaly Ganusov
Tomas Ganz
Yoav Ganzach
Giulia Ganzetti
Michael Gänzle
Bin Gao
Chen Gao
Chengjiang Gao
Daozhou Gao
Dawen Gao
Fan Gao
Feng Gao
Feng Guang Gao
Ge Gao
Guangjun Gao
Guimin Gao
Hongchang Gao
Hongjian Gao
Huijiang Gao
Jian Gao
Jianxi Gao
Jin Gao
Jingxia Gao
Jinhao Gao
Jinming Gao
Jinpeng Gao
Junling Gao
Junping Gao
Lei Gao
Liang Gao
Lianghui Gao
Lian-Ming Gao
Lihong Gao
Lin Gao
Ling Gao
Liping Gao
Lu Gao
Mingming Gao
Mu Gao
Nan Gao
Qian Gao
Qing-Hua Gao
Shaobing Gao
Shengjie Gao
Song Gao
Tianshun Gao
Ting Gao
Weimin Gao
Xiang Gao

Xiangyun Gao
Xian-Shu Gao
Xiaojing Gao
Xiaorong Gao
Xing-Huang Gao
Xiujun Gao
Xue Gao
Xuelu Gao
Yahui Gao
Yang Gao
Yaozong Gao
Ying Gao
Youhe Gao
Yu Gao
Zan Gao
Zheng-Nan Gao
Laurent Gapin
Vadim Gaponenko
Michael Garabedian
Urtzi Garaigorta
Kevin Garala
Claire Garandeau
Beatriz Garat
Andrea Garatti
Giovanni Garau
Jessica Garb
Juan C. Garbalosa
Francesca Garbarini
Sergio Garbarino
David Garber
Håvard Garberg
Anna Rosa Garbuglia
Annarosa Garbuglia
Svitlana Garbuzova-Davis
Elena Garcea
Frank Garcea
Henri-Jean Garchon
Alberto Garcia
Alejandro Garcia
Andres Calderin Garcia
Brandon Garcia
David Garcia
Gabriela Garcia
Guilherme Garcia
Irene Garcia
Jair Garcia
Jonathan Garcia
Kevin Garcia
L. Rene Garcia
Linda Garcia
Luis Garcia
M. Alejandro Garcia
Maite Garcia
Maricarmen Garcia
Meritxell Garcia
Michael Garcia
Nuno Garcia
Paul Garcia
Pilar Garcia
Sara Garcia
Serge Garcia
Tiffany Garcia
Valter Duro Garcia
Veronica Garcia
Andrés García
Beatriz García
Federico García
Miriam R. García
Rosario García
Antonio Garcia de Herreros
David García de León
Francisco J. García de León
Patricia García de Olalla
Darío Garcia de Viedma
Elena Garcia Fruitos
Guillermo Garcia Garcia
Hector Garcia Garcia
David Garcia Juan
Alexandro Garcia Mas
Marta Garcia Montojo
Alejandro Garcia Nuñez
Clara Garcia Rodenas
Mercedes García Sánchez
Monica Garcia-Alloza
Luz Garcia-Alonso
M.C. García-Alonso
A. García-Álvarez
Jaime García-Añoveros
Fernando Garcia-Arenal
Judith Garcia-Aymerich
Diego García-Ayuso
Alberto Garcia-Basteiro
María Laura García-Bermejo
Ana García-Blanco
F. García-Camacho
Patricia Garcia-Canadilla
Victoria Garcia-Cardenas
Fabio García-Castro
Luis Alberto García-Cortés
Francisco García-del Portillo
Diego Garcia-Diaz
Pablo Garcia-Diaz

César García-Díaz
Miguel García-Díaz
Guillermo Garcia-Effron
David García-Fresnadillo
Guillermo Garcia-Garcia
Lourdes García-García
Juan Garcia-Gomez
Manuel García-Goñi
Graciela García-Guzmán
Claudio Garcia-Herrera
Ricardo García-Herrera
J. Arturo García-Horsman
Vanessa Garcia-Larsen
Luz Garcia-Longoria
David García-López
Erik García-Machado
Guillermo García-Manero
Raúl García-Marcos
Jesús García-Martínez
Jordi Garcia-Mas
Mauricio García-Mateu
Jose Garcia-Mazcorro
Victor Hugo Garcia-Merchan
Xavier Garcia-Moll
Teresa García-Muñoz
Vicente Garcia-Navas
Luis García-Olmos
Fuensanta García-Orenes
Daniel García-Ovejero
Azucena Garcia-Palacios
Pablo García-Pavía
Manuel Garcia-Ramirez
María Isabel García-Real
Juan Luis García-Rodriguez
Carmen Garcia-Ruiz
Juan Garcia-Ruiz
Jose Garcia-Salcedo
Ma. Cecilia Garcia-Sancho
Javier García-Sancho
Luis M Garcia-Segura
Alfonso Garcia-Sosa
Alfonso T. Garcia-Sosa
Javier Garcia-Tirado
Margarita Garcia-Vila
M. Pilar Garcillán-Barcia
Elsa Garcin
Christophe Garcion
Stephen Garczynski
Thomas Gardali
Thomas Gardella
Laurent Garderet
Susana Gardete
Stephanie Gardham
Elizabeth Gardiner
Matthew Gardiner
Stuart Gardiner
Fausto Gardini
Beth Gardner
Caleb Gardner
David Gardner
Dianne Gardner
Humphrey Gardner
Iain Gardner
James Gardner
Jerad Gardner
Jonathan Gardner
Kevin Gardner
Kyle Gardner
Matthew Gardner
Michael Gardner
Paul Gardner
Raquel Gardner
Thomas Gardner
Fabrizio Gardoni
Jason Gardosi
Mathieu Garel
Andrew Garey
Richard Garfein
Yonathan Garfias
Steven Garfin
D. Garfinkel
Michele Garfinkel
G.K. Garg
L. Garg
Mandeep Garg
Manohar Garg
Mayur Garg
Nisha Garg
Ramandeep Garg
Sanjay Garg
Seema Garg
Andrea Gargas
Floriana Gargiulo
Giuseppe Gargiulo
Paolo Gargiulo
Manuela Gariboldi
Tara Gariepy
Mutien Garigliany
Venkata Garikipati
Nicolas Garin
Olatz Garin
Maria Teresa Garín

Filiz Garip
Theodore Garland
Cecilia Garlanda
Scott Garman
Teresa Garnatje
Malgorzata Garnczarska
Pierre Garneau
Sylvie Garneau-Tsodikova
Brett Garner
Ethan Garner
Joseph Garner
Omai Garner
Trenton Garner
Sigisfredo Garnica
Anne Garnier
Stéphane Garnier
Cesare Garofalo
Giuliano Garofolo
Olivier Garraud
Wiebke Garrels
Eilidh Garrett
Francine Garrett
Michael Garrett
R. Kelly Garrett
Timothy Garrett
Elizabeth Garrett-Mayer
Lee Ann Garrett-Sinha
Carmen Garrido
Daniel Garrido
Dolores Garrido
Juan Garrido
Lucia Garrido
Manuel Garrido-Ramos
Patricia Garrido-Vasquez
R.G. Garriga
Sebastien Garrigues
Didier Garriguet
Patton Garriott
Gregory Garrison
Kathleen Garrison
David Garry
Vincent Garry
Mark Garside
Antje Garten
Marc Gartenberg
John Garthwaite
Alison Gartland
Deirdre Gartland
Marieke Gartner
Fátima Gärtner
Brett Gartrell
Chris Garvey
Gavin Garvey
Raven Garvey
Jeffrey Garvin
Beth Garvy
Justus Garweg
Thomas Gary
Miguel Garza
Ulises Garza-Ramos
Ingrid Garzon
Tomas Garzon-Muvdi
Janvier Gasana
Caley Gasch
A.R. Gascón
Simani Gaseitsiwe
M. Hussein Gasem
Jessica Gasiorek
Brianna Gaskill
Peter Gaskill
Carl Gaspar
Tania Gaspar
Anthony Gaspari
Flavio Gaspari
Marco Gaspari
Antonio Gasparrini
Nicholas Gaspelin
Lawrence D. Gaspers
Philippe Gasque
Philip Gasquoine
Justin Gass
Peter Gass
Natig Gassanov
Pamela Gasse
Thomas Gasser
Natalie Gassman
Aaron Gassmann
Max Gassmann
Eduardo Gastal
Amalia Gastaldelli
Pablo Gastaminza
Paul Gastanaduy
Johann Gasteiger
Amelia Gaston
Anca Gaston
Jeremy Gaston
Kevin Gaston
Robert Gatenby
Paul Gatenholm
Deanna Gates
John Gatesy
David Gathara

Grace Gathungu
Sergios Gatidis
Mohamed Gatie
Blandine Gatta-Cherifi
Gilka Gattas
Valter Gattei
Roberto Gatti
Maurice Gattis
Emilia M. Gatto
Laurent Gatto
Christiane Gatz
Maxime Gauberti
John Gaudet
Mia Gaudet
Rachelle Gaudet
Silvana Gaudieri
Timothy Gaudin
Arnaud Gaudinat
Luciano Gaudio
Benoit Gaudou
Bertrand Gauffre
Phillip Gauger
Andrea Gaughan
Joseph Gaugler
Ingrid Gaugler-Senden
Natalie Gauld
Victor Gault
Tobias Gauss
Nagsen Gautam
P. Gautam
Rekha Gautam
Uma Shankar Gautam
Yograj Gautam
Jeremie Gautheron
Karine Gauthier
Sylvie Gauthier
Sidney Gauthreaux
Aude Gautier
Emmanuel-Laurent Gautier
Lara Gautier
Amandine Gautier-Stein
Oliver Gautschi
Laetitia Gauvin
Julie Gauzere
Paulo Gavaia
Floriana Gavazzi
Frances Gavelli
Olivier Gavet
Brandon Gavett
David Gavin
Tim Gavin
Timothy Gavin
Nirit Gavish
Yoni Gavish
Maria Gavrilo
Leonid Gavrilov
Andrew Gavrin
Indika Gawarammana
Katarzyna Gawron
Denise Gay
John Gay
Magdalena Gayà-Vidal
Ezra Gayawan
Joel Gaydos
Christopher Gayer
Daria Gaykalova
Philippe Gayral
Esteban Gazel
Maria Gazouli
Angelo Gazzano
Silvia Gazzin
Lidia Gazzola
Mattia Gazzola
Diego Gazzolo
Marco Gazzoni
Gaoxiang Ge
Hao Ge
Hu Ge
Jingran Ge
Juhong Ge
Lei Ge
Liang Ge
Mingwei Ge
Ruowen Ge
Xianhong Ge
Xiaoyan Ge
Xiuli Ge
Xue-Jun Ge
Yingbin Ge
Zheng Ge
Zhenming Ge
Zhi-Dong Ge
Zigang Ge
Brad Geary
Bradley Geary
David Geary
Matthew Geary
Montserrat Gea-Sánchez
Florian Geay
Jochen Gebauer
Sebastian Gebauer
Susanne Gebhard

Christiane Gebhardt
Rolf Gebhardt
Daniel Gebo
Kahsa Tadel Gebre
Abebe Gebregiorgis
Endrias Gebremedhin
Gebremedhin Gebrezgabiher
Onur Geckili
Carolyn Geczy
Rolf Gedeborg
Lealem Gedefaw
Rainer Gedeit
Jillian Gedeon
Tomáš Gedeon
Vibe Gedsø Frøkjær
Albert Gee
Dylan Gee
Shirley Gee
Alexander Geen
Vincent Geenen
Andrew Geers
Anja Geerts
Dirk Geerts
Purushothaman Geethanjali
Michael Geeves
Audrey Geffen
David Geffen
Matthew Gegg
Malia A. Gehan
Marcelo Gehara
Ricardo Gehrau
Mario Gehri
Adam Gehring
Andreas Gehring
Christoph Gehring
Dominic Gehring
Niels Gehring
Sergio Gehrke
Philip Gehrman
Mathias Gehrmann
John Geibel
Eugene Geidelberg
Bernhard Geierstanger
Dietmar Geiger
Otto Geiger
Paige Geiger
Sonja Geiger
Eric Geijteman
Janet Geipel
Steffen Geis
Roland Geisberger
Samuel Geiseler
Sven Geiselhardt
Rolf Geisen
Eveline Geiser
Thomas Geiser
Eldon Geisert
Mia Geisinger
Carsten Geisler
Jane Geisler-Lee
Florian Gekeler
A. Gelasakis
Stefan Gelcich
Hubrecht Gelderblom
Peter Geldhof
Jonas Geldmann
Lendert Gelens
Judith Gelernter
Alan Gelfand
Cecilia Gelfi
Armando Geller
David Geller
David A. Geller
Gail Geller
Ron Geller
Stacie Geller
Angela Gelli
Jean-Christophe Gelly
Dambala Gelo
Ricardo Gelpi
Stanton Gelvin
Marlene Gemelon
Robert Gemmill
Suzuki Gen
Kimberly Genareau
Thiago Genaro-Mattos
Becky Genberg
Saleh Gencer
Caroline Genco
Takuya Genda
Howard Gendelman
Mark Gendreau
Dominique Gendrel
Fernand-Pierre Gendron
Ketevan Gendzekhadze
Joseph Genereux
Nicholas Generous
Elke Genersch
Olivier Genest
Pierre Genevaux
Anne-Marie Geneviève
Jinju Geng

Liying Geng
Qingshan Geng
Shuo Geng
Tuoyu Geng
Yan Geng
Yupeng Geng
Olga Genilloud
Guy Genin
Shawn Geniole
Matthew Geniza
Maria Laura Gennaro
Robert Gennis
Jean-Luc Gennisson
Mathieu Génois
Helen Genova
Giovanni Genovese
Andrea Genre
Rudiger Gens
Henrik Gensicke
Fernando Genta
Francesco Gentile
Giorgio Gentile
Luciana Gentile
Saverio Gentile
Fred Gentili
Rodolfo Gentili
Sheridan Gentili
Nikki Gentle
Matthew Gentry
Diego Genuário
Valérie Geoffroy
P. Geoghegan
Alex Georgakilas
Alex George
Christine George
Keith George
Maureen George
Olivier George
Paul George
Phillip George
Varghese George
Barbara George-Jaeggli
Philippe Georgel
Arthur Georges
Jean-Yves Georges
Mark Georgeson
Annabelle Georgetta
Katholiki Georgiades
Pantelis Georgiades
Millie Georgiadis
Panagiotis Georgianos
Petia Georgiev
Christos Georgiou
Constantinos Georgiou
George Georgiou
Michael Georgopoulos
Urania Georgopoulou
Susanne Georgsson
Nimish Gera
Nick Geraci
Aline Gerage
Daniel Geraghty
Nadine Gerard
Chiara Gerardi
Jaline Gerardin
Patrick Gérardin
Nicole Gerardo
Dmitry Gerashchenko
Evgenia Gerasimovskaya
Cyrill Geraud
Antono Gerbase
Andrew Gerber
Brian Gerber
David Gerber
Jacobus Gerber
Ruan Gerber
Gregory Gerdeman
Norbert Gerdes
Björn Gerdle
Marco Gerdol
Volker Gerdts
Akos Gerencser
Vanessa Gerente
Erika Gergerich
Alex Gerhard
Markus Gerhard
Francesco Geri
Liesbet Geris
Justin Gerke
Theo Gerkema
Gabriele Gerlach
Martin Gerlach
Philipp Gerlach
Philip Gerlee
Denis Gerlier
Dan Gerling
Christoph Gerlinger
Christian Gerloff
Pierre Germain
Charlotte Germain-Aubrey
Georgios Germanidis
Menno Germans

Anastasios Germenis
Dori Germolec
Pierre Germon
Alison Gernand
Ramkishore Gernapudi
Christopher Gerner
Shane Gero
G. Gerold
Ioannis Gerothanassis
Philippe Gerrienne
Alida Gerritsen
Marrtijn Gerritsen
Samuel Gershman
Susan Gershman
Michael Gershon
Robyn Gershon
Timothy Gershon
Laurel Gershwin
Aleeza Gerstein
Brenda Gerull
Roland Gerull
Thomas Gervais
Will Gervais
Francesco Gervasio
Klaus Gerwert
Bill Gerwick
Taras Gerya
Matthias Gesemann
Antoine Gessain
Stefania Gessi
Ignacio Gestoso
Jason Gestwicki
Nancy Getchell
Jocelyn Getgen
Spiro Getsios
Godfrey Getz
Lutz Geue
Sebastian Geukes
Jan Geuns
Aron Geurts
Jeroen Geurts
Olivier Gevaert
Thomas Gevaert
Richard Gevirtz
Artur Gevorgyan
David Gewirtz
Felipe Geyer
Duygu Gezen-Ak
Seyyed Abolghasem Ghadami
Ebrahim Ghaderi
Santhosh Ghadge
Samir Ghadiali
Anas Ghadouani
Sina Ghaemmaghami
Noushin Ghaffari
Fariborz Ghaffarpasand
Siddhesh Ghag
Arezou Ghahghaee
Saeideh Ghahghaei
Ria Ghai
Rohit Ghai
Mohammad Ghalambaz
Bijan Ghaleh
Haitham Ghalwash
Shahram Ghanaati
Shahla Ghanbari
Mahmoud Ghandi
Khaled Ghanem
Khalil Ghanem
Makarand Ghangrekar
Mahdi Ghani
Srijani (Deb) Ghanta
Saba Gharaei
Morteza Gharib
Behrooz Ghasemishabankareh
Behnam Ghasemzadeh
Hassan Ghassemi
Shibnath Ghatak
Mohammad Amin Ghatee
Yaser Ghavami
Sandra Ghayad
Chafik Ghayor
Majid Ghayour-Mobarhan
Hussein Ghazale
Seda Ghazaryan
Arjumand Ghazi
Noureldin Ghazy
Annette Ghee
Tarik Gheit
Ariane Ghekiere
Elena Gheorghiu
Giovanni Gherardi
Lisa Gherardini
Calin Gherman
Cristian Gherman
Dario Ghersi
Pol Ghesquiere
Fabio Ghezzi
Jean-Eric Ghia
Lorenzo Ghiadoni
Michele Ghidini
Veronica Ghiglieri

Claudia Ghigna
Stefano Ghignone
Alessandra Ghigo
Rajan Ghimire
Andrew Ghio
Maria Ghirardi
Daniela Ghisotti
Hossein Ghodosi
Reza Ghodssi
Ali Gholami
Asa Gholizadeh
Hassan M. Ghomrawi
Hazem Ghoneim
Behnaz Ghoraani
Mohammad Ghorbanhoseini
Ratna Ghosal
Abhimanyu Ghose
Bishwajit Ghose
Subroto Ghose
Toorjo Ghose
Abhrajyoti Ghosh
Ajit Ghosh
Arkasubhra Ghosh
Arko Ghosh
Arpan Ghosh
Arup Ghosh
Asish Ghosh
Bikramjit Ghosh
Chandra Ghosh
Debajyoti Ghosh
Debarchana Ghosh
Debashis Ghosh
Dhimankrishna Ghosh
Dipankar Ghosh
Jagadish Ghosh
Mallika Ghosh
Manosij Ghosh
Pallavi Ghosh
Paramita Ghosh
Preetam Ghosh
Prosenjit Ghosh
Saikat Kumar Ghosh
Samiran Ghosh
Santosh Ghosh
Saswata Ghosh
Shyamasree Ghosh
Siddhartha Ghosh
Soumitra Ghosh
Sourav Ghosh
Subrata Ghosh
Sudip Ghosh
Sujit Ghosh
Sujoy Ghosh
Sumantra Ghosh
Tusharkanti Ghosh
Vijayalakshmi Ghosh
Zhumur Ghosh
Kalpana Ghoshal
Pushpankur Ghoshal
Suparna Ghosh-Jerath
Yassine Ghouzam
Maysam Ghovanloo
Sherief Ghozy
Othman Ghribi
Philippe Giabbanelli
Lorenzo Giacani
Mauro Giacca
Domenico Giacco
Giorgio Giaccone
Daniela Giachino
Daniele Roberto Giacobbe
Peter Giacobbi
Paolo Giacobini
Roberto Giacomelli
Darci Giacomini
Sylvaine Giakoumi
E. Gialafos
Evangelos Giamarellos-Bourboulis
Dario Giambalvo
Barbara Giambene
Maria Adele Giamberardino
Marcia Giambiagi-deMarval
Antonino Giambona
Giovanni M. Giammanco
Elisabetta Gianazza
Sara Gianella
Umberto Gianelli
Valentina Gianfelici
Adriana Giangrande
Angela Giangrande
Silvia Gianì
Nikolaos Giannakopoulos
G. Giannakoulas
Chiara Giannarelli
Elisabetta Di Giannatale
Sergio Giannattasio
Simone Giannecchini
Ilias Giannenas
Gregory Giannone
Alberto Giannoni
Eric Giannoni
Silvia Gianola

Elena Gianulis
Efstathios Giaouris
Charles Giardina
Paola Giardina
Traber Giardina
William Giardino
Ellen Giarelli
Debbie Giaschi
Benoit Giasson
Giorgio Giatsidis
Gianluca Giavaresi
Pedro Giavina-Bianchi
Martin Gibala
Deborah Gibbons
John Gibbons
Allen Gibbs
Benjamin Gibbs
Daniel Gibbs
Heather Gibbs
Julie Gibbs
Lee Gibbs
Lisa Gibbs
Melanie Gibbs
Anaïs Gibert
Dino Gibertoni
Maurizio Gibin
Katherine Gibney
Nicole Gibran
Amanda Gibson
Bradley Gibson
Britton Gibson
C. Gibson
Daniel Gibson
Deanna Gibson
Douglas Gibson
Frank Gibson
Gerusa Gibson
Greg Gibson
Joshua Gibson
Kristen Gibson
Laura Gibson
Peter Gibson
Robert Gibson
Todd Gibson
Dimos Gidaris
Samuel Gidding
Jasmine Gideon
Thomas Gidlewski
Albert Gidon
Risha Gidwani
Katrin Giel
Roben Gieling
Micheal Gielnik
Martin Giera
R. Gierczyňski
Mark Gierl
Karl Giese
Philip Giffard
Danna Gifford
Miriam Gifford
Antonietta Gigante
Francesco Giganti
Roman Giger
Pierre Giglio
Robson Giglio
Francis Gigliotti
Christopher Gignoux
Sylvain Gigout
Marion Gijbels
Jerome Gijselaers
Frank Gijsen
Helen G. Gika
Ana Gil
Fernando Gil
Hyo-Wook Gil
Octávia Gil
Rosario Gil
Susana Gil
Sharon Gilaie-Dotan
Luís Gilarranz
Ignacio Gil-Bazo
Caroline Gilbert
David Gilbert
Eric Gilbert
Kathleen M Gilbert
Marius Gilbert
Peter Gilbert
Richard Gilbert
Robert Gilbert
Stephen Gilbert
Asaf Gilboa
Eva Gilboa-Schchtman
Ian Gilby
Rodica Gilca
Gail Gilchrist
Graham Gilchrist
Nigel Gilchrist
Jennifer Gilda
Daniel Gildea
Kelly Gildersleeve
Jennifer Gile
Opher Gileadi

Alex Gileles-Hillel
Audrey Giles
Erin Giles
Sam Giles
Wayne Giles
Josep-Maria Gili Sardà
François Giligny
Brian Gill
Christopher Gill
Clare Gill
David Gill
Jason Gill
Jonathan Gill
Prit Gill
Ravinder Gill
Ritu Gill
Saar Gill
Simone Gill
Erin Gillam
Steve Gillard
Glenda Gillaspy
John Gilleard
Ken Giller
Brigid Gillespie
Gordon Gillespie
Joseph Gillespie
Laura Gillespie
Ulrika Gillespie
Kristen Gillespie-Lynch
Francesca Gilli
Frank Gilliam
Melissa Gilliam
Bernadette Gillick
Conor Gilligan
Anna Gillio-Tos
Jesse Gillis
Stewart Gillmor
Osnat Gillor
J. Gilman
Robert Gilman
Alice Gilman-Sachs
Joanna Gil-Mohapel
L. Anne Gilmore
Rick Gilmore
Stephen Gilmore
David Gilmour
Stuart Gilmour
Marta Gil-Ortega
Nicholas Gilpin
Aditya Gilra
James Gilroy
Vicente Gilsanz
Amy Gilson
Erik Giltay
Fabricia Gimenes
Daniel Gimenez
Estela Giménez
Luis Giménez-Lirola
Lucia Gimeno
Teresa Gimeno
Maaria Ginai
Wolfgang Gindl-Altmutte
Florelle Gindraux
Silvia Ginés
Giorgio Ginesu
Cosmina Gingaras
Anne Gingery
Bruno Gingras
Michel Gingras
Rosa Gini
Roman Ginnan
Paul Ginoux
Carren Ginsburg
Ken Ginsburg
Alexandru Ginsca
Donna Ginther
Karni Ginzburg
Antimo Gioiello
Carlo Giorda
Paolo Giordani
Antonio Giordano
Frank Giordano
Ricardo Giordano
Silvia Giordano
Thomas Giordano
Kyriaki Giorgakoudi
Assuero Giorgetti
Sophie Giorgetti-Peraldi
Emanuele Giorgi
Francesco Giorgianni
Giurato Giorgio
Paolo Giorgirossi
Nicholas Giori
Ioannis Gioulbasanis
Marta Giovanetti
Pia Giovannelli
Tania Giovannetti
Federico Giove
Luca Giovenella
Juan Pablo Giraldo
Patricia Giraldo
Rafael Giraldo

Albert Giralt
Ana Girão
Jeffrey Girard
Pascal Girard
Philpe Girard
Pierre Marie Girard
Pierre-Marie Girard
Sylvie Girard
Marco Girardello
Enrico Girardi
Massimo Girardis
Deborah Girasek
Andrew Giraud
Eric Giraud
Mathieu Giraud
Christophe Giraud-Carrier
Tomás Girbés
Tamara Girbl
Linus Girdland-Flink
Ewa Girejko
Carlo Girelli
Teresa Giret
Ashok Giri
Bhoopander Giri
Pankaj Giri
Shailendra Giri
Veda Giri
Marzia Giribaldi
Rani M. Girija
Sanal Girija
Smiline Girija
Santhosh Girirajan
Thomas Girke
Marc Girondot
José-Antonio Girón-González
Albert Girotti
Giorgia Girotto
Nathalie Giroud
Caroline Giroux
Michael Giroux
Christoph Gisinger
Angelo Gismondi
Jonathan Gisser
Carmela Gissi
Mika Gissler
Nicholas Gist
Richard Gist
Simon Giszter
Daniel Gitaí
I.Z. Gitas
Darren Gitelman
Sophie Githinji
Gitsios Gitsioudis
Adriana Gittenberger-de Groot
Gianluca Giuberti
Nick Giudice
Paolo Giudici
Alessandro Giuliani
Marina Giuliano
Richard Giulianotti
Cecilia Giulivi
Rodolfo Giunchetti
David Gius
Paola Giussani
Alain Givaudan
Barbara Given
Rodney Givney
Sotiria Gizani
Marija Gizdavic-Nikolaidis
Camilla Gizzi
Annie Gjelsvik
Julijana Gjorgjieva
Eleni Gkana
Nikolaos Gkantidis
Georgios Gkimpas
Enrico Glaab
Rebecca Gladdy
Angela Glading
Melissa Gladstone
Douglas Gladue
Mark Gladwin
Inna P. Gladysheva
Brian Glancy
Stanton Glantz
Martin Glas
Jon Glasby
Amy L. Glaser
Benjamin Glaser
Shannon Glaser
Annegret Glasow
John Glass
Kimberly Glass
Mindy Glass
Nancy Glass
Marilyn Glassberg
Allison Glasser
Adam Glassman
Amanda Glassman
Richard Glassock
Elizabeth Glater
Manuela Glattacker
Ingmar Glauche

Howard Glauert
Dominique Glauser
Frank Glaw
Elizabeth Glaze
Douglas Glazier
Daniel Gleason
Evanna Gleason
Jennifer Gleason
Stephen Gleddie
Dana Glei
Norbert Gleicher
Christian Gleissner
Arthur Glenberg
Deborah Glencross
Miriam Glendell
Anthony Glenn
Jordan Glenn
Thomas Glenn
Enrico Glerean
Isaias Glezer
David Glick
Joseph Glicksohn
Thomas Gliddon
Sherry Glied
Michael Gliem
Vladimir Gligorijevic
Gennadi Glinsky
Stephen Gliske
Ros Gloag
Ruth Globus
Erik Glocker
Bernhard Glodny
Greg Gloor
Griet Glorieux
Liviu Glosan
Mark Glover
Kira Glover-Cutter
Ashley Gluchowski
Kevin Gluck
Judith Glück
Eliane Gluckman
Stefan Glüge
Robert Glushko
Judith Glynn
Laura Glynn
Nancy Glynn
Bruce Gnade
Timo Gnambs
Daniela Gnani
Giorgio Gnavi
Antonio Gnoni
David Go
Pål Goa
Cyrille Goarant
Emanuela Gobbi
Marco Gobbi
Mauro Gobbi
Angélique Gobet
Katrina Gobetz
Michael Goblirsch
Johan Gobom
Malgorzata Goclowska
Elen Gocza
Swetha Godavarthi
Anuradha Godavarty
Ariane Godbout
Roseline Godbout
John Goddard
William Goddard
Bruno Goddeeris
Martin Godefroid
Tanja Godenschwege
Julien Godet
Alan Godfrey
Larry Godfrey
Maurice Godfrey
Tony Godfrey
Jacques Godfroid
Deepali Godha
Nallamuthu Godhantaraman
Gerardo Díaz Godínez
Catarina Godinho
Cristina Godinho
Susan Godlonton
Barbara Gödl-Purrer
Brian Godman
Veronique Godot
Pablo Godoy
Pere Godoy
Adrienne Godschalx
Hayward Godwin
Ian Godwin
Ryan Godwin
Maciek Godycki-Cwirko
Sandra Goebbels
Jens Goebel
Ted Goebel
Ulrich Goebel
Jeremy Goecks
Jann Goedecke
James Goedert
Craig Goehler

Lisa Goehler
Marco Goeijenbier
Apollina Goel
Gunjan Goel
Pranay Goel
Ramesh Goel
Sandeep Goel
Sanjay Goel
Shailendra Goel
Sudhir Goel
Vinod Goel
Anouk Goemans
Ludger Goeminne
Anu Goenka
Christine Goeppel
Sebastian Goerg
Katharina Goerlich
Christine Goertz
Paula Goes
Aristóteles Góes-Neto
Peter Goethals
Peter Goettig
Claudia Goettsch
Carolin Goetz
Thorsten Goetze
Lorraine Goeuriot
Stephen Goff
Anne Goffart
Eric Goffin
Ameena Goga
Parag Gogate
Michael Goggins
William Goggins
Rajan Gogna
Andrea Gogos
Penelope Gogou
Valérie Goguel
Vladimir Gogvadze
Chin Foo Goh
Gerald Goh
Kian Mau Goh
Kwang-Il Goh
Lee Gan Goh
Anita Gohel
Suril Gohel
Reginald Gohh
Bettina Gohlke
Andreas Gohritz
Pablo G. Goicoechea
Andrea Goijman
Scott Going
William Goins
Pedro Henrique Gois
Satoshi Gojo
Jun Gojobori
Mustafa Gök
Koichi Goka
Ziya Gokaslan
Omer Gokcumen
Markus Göker
Maya Gokhale
Kuppan Gokulan
David Golan
Janusz Golaszewski
Philippe Golay
Jennifer Golbeck
Alexandra Golby
Barry Gold
Lisa Gold
Scott Gold
Caren Goldberg
Daniel Goldberg
Daphne Goldberg
Gary Goldberg
Rachel Goldberg
Ronald Goldberg
Tony Goldberg
Ximena Goldberg
David Goldblatt
Mark Goldblatt
Christopher Golden
Sara Golden
Robert Goldenberg
David Goldfarb
David Goldgar
G. Golding
Jean Golding
Michael Golding
Ross Goldingay
Anne Goldizen
Monica Goldklang
Andrew Goldman
Barry Goldman
Dana Goldman
I. David Goldman
Irwin Goldman
Jason Goldman
Jeremy Goldman
Roberta Goldman
Seth Goldman
Emily Goldmann
Wolfgang Goldmann

Ariel Goldraij
Daniel Goldreich
Mary Goldring
Pablo Goldschmidt
Susan Goldsmith
Paul Goldspink
Abraham Goldstein
Adam Goldstein
Allan Goldstein
Debra Goldstein
Emily Goldstein
J.A. Goldstein
Jennifer Goldstein
Joshua Goldstein
Louis Goldstein
Sara Goldstein
Jason Goldstick
Sharyn Goldstien
Micah Goldwater
Blanka Golebiowski
Marek Golebiowski
Nikola Golenhofen
Srinivas Goli
Georg Goliasch
Dominik Golicki
Marko Golicnik
Hudson Golino
Mark Golitko
Sampath Gollapudi
Huw Golledge
Audra Gollenberg
Isabella Gollini
Sandra Gollnick
Peter Gollwitzer
Pablo Goloboff
Beatrice Golomb
Diego Golombek
Irina Golovleva
Thomas Golper
Rieta Gols
Vassilij Goltsev
Mari Golub
Anna Golubeva
Olga V. Golyshina
Lina Gölz
Jesús Gomar
Monica Gomaraschi
Ilias Gomatos
Sara Gombatto
Leonard Gomella
Charles Gomer
Richard Gomer
Sjaan Gomersall
Aldrin Gomes
Ana Gomes
Anderson Gomes
Anita Gomes
Bruno Gomes
Carmen Gomes
Cristiano M. Gomes
Cynthia Gomes
Edgar Gomes
Gabriela Gomes
Gayathri Gomes
Harrison Gomes
J. Gomes
José-Eduardo Gomes
Karina Gomes
Maria Salomé Gomes
Marila Gomes
Nelson Gomes
Paula Gomes
Pedro Gomes
Samirah Gomes
Viviane Gomes
Eric Gomès
Moemy Gomes de Moraes
Maria Gomes-Solecki
Adriana Gomez
Africa Gomez
Alice Gomez
Enrique Gomez
Felicia Gomez
Fernando Gomez
Frank Gomez
Hernando Gomez
Luis Fernando Gomez
Maria Adelaida Gomez
Miguel Gomez
Pierrick Gomez
Ricardo Gomez
Rocio Gomez
Timothy Gomez
Juan-Carlos Gómez
Enrique Gomez Barrena
Diego Gomez de Barreda
Sol Gomez de la Torre Canny
Gerardo Gómez Moreno
Fernando Gómez Muñoz
Jorge Gomez Tejeda Zanudo
Javier Gomez-Ambrosi
Jose Gomez-Arroyo

Julian Gomez-Cambronero
Inés Mª Gómez-Chacón
Mªpilar Gómez-Garre
Manuel Gomez-Gonzalez
Armando Gómez-Guerrero
Maria Gomez-Jimenez
Andres Gomez-Lievano
Alex Gomez-Marin
Concepcion Gomez-Mena
Diego Gomez-Nicola
Natividad Gomez-Roman
Paulina Gomez-Rubio
Celso Gomez-Sanchez
Tiziano Gomiero
Sandra Gomis Pont
Jennifer Gommerman
Athina Gompou
Alexandra Goncalves
Bruno Goncalves
Elizabeth Goncalves
Marilda Goncalves
Célia Gonçalves
I.S. Gonçalves
Teresa Gonçalves
Viviane Gonçalves
J. Gonçalves da Silva Leite
Rute Maria Gonçalves de Andrade
Thiago Gonçalves-Souza
Elena Goncharova
David Gonda
Florence Gondret
Cedric Gondro
Lee Goneau
Elena Gonella
Binsheng Gong
Chang Gong
Chengliang Gong
Diankun Gong
Feng Gong
Haipeng Gong
Hai-Qing Gong
Hong-Yi Gong
Hui Gong
Jun Gong
Lan Gong
Liang Gong
Mengyan Gong
Peng Gong
Qiang Gong
Rujun Gong
Xinqi Gong
Xun Gong
Yanhong Gong
Yiping Gong
Yongji Gong
Youhui Gong
Yu-Shun Gong
ZhiCheng Gong
Giorgio Gonnella
Mathieu Gonnet
Takehisa Gono
Constantin Gonsior
Michal Gontarz
Paolo Gonthier
Lucia Gonzales
Rueben Gonzales
Eliana Gonzales-Vigil
Aida Gonzalez
Alberto Gonzalez
Andres Gonzalez
Anjelica Gonzalez
Antonio Gonzalez
Arantxa Gonzalez
Carmen Gonzalez
Cesar Gonzalez
Cleotilde Gonzalez
Elena Gonzalez
Elizabeth Gonzalez
Ernesto Perez Gonzalez
Esther Gonzalez
Francisco Gonzalez
Guadalupe Gonzalez
Iveth Gonzalez
Javier Gonzalez
Jorge Gonzalez
Jose Gonzalez
José Gonzalez
Mileidy Gonzalez
Mirna Gonzalez
Nathalie Gonzalez
Pablo Gonzalez
Rosa Gonzalez
Salvador Gonzalez
Sonia Gonzalez
Suzanne Gonzalez
Gustavo Gonzalez Cuevas
Luis González de la Vara
Elvira Gonzalez de Mejia
Xavier Gonzàlez Farré
Carlos Gonzalez Leon
Daniel González Maglio
Manuel Gonzalez Rivero

Isidoro González-Álvaro
Sandra Gonzalez-Bailón
Sara M. González-Betancor
César González-Blanch
Claudia Gonzalez-Brambila
Lucia Gonzalez-Buendia
Joel González-Cabrera
Javier Gonzalez-Castillo
David González-Chica
Humberto González-Díaz
Victoria Gonzalez-Dugo
Daniel Gonzalez-Dunia
Javier Gonzalez-Gallego
Manuel Gonzalez-Garay
Ismael González-García
Miguel A González-Gay
Graciela Gonzalez-Gil
Mario Gonzalez-Gronow
T. González-Hernández
Verónica González-López
Javier González-Maeso
Lorena González-Manzano
Antonio González-Martín
Fernando Danilo Gonzalez-Nilo
Héctor González-Ocampo
Alejandro González-Ojeda
Emilio Gonzalez-Parra
Regino Gonzalez-Peralta
Carlos Gonzalez-Rey
Elena Gonzalez-Rey
R. Gonzalez-Reyes
José A. González-Reyes
Ana Gonzalez-Roldan
Jose Gonzalez-Valdez
Silvia Gonzali
Allen Good
Deborah Good
Erin Good
Michael Good
Misty Good
Alison Goodall
Mark Goodarzi
Patrick Goodbourn
Bruce Goode
Lynda Goodfellow
Adam Goodie
Martin Goodier
Michael Goodin
Linda Gooding
Michael Gooding
Catherine Goodman
James Goodman
Joel Goodman
Mark Goodman
Michael Goodman
Michael L. Goodman
Philip Goodney
Lisa Goodrich
Suzanne Goodrich
J. Max Goodson
James Goodson
Robert Goodspeed
Douglas Goodwin
Mark Goodwin
Matthew Goodwin
Adam Goodworth
Jonathon Gooi
Charlotte Gooskens
Tine Goossens
Elisha Gootwine
Satish Gopal
Udhayakumar Gopal
Jay Gopalakrishnan
Natarajan Gopalakrishnan
Ramakrishnan Gopalakrishnan
Ranjith Gopalakrishnan
Chaitra Gopalappa
Ulrich Göpfert
Daniel Gopher
Irina Gopich
Arvind Gopinath
Bamini Gopinath
P. Gopinath
Unni Gopinathan
Anupama Gopisetty
Margarete Goppelt-Struebe
David Gopurenko
Dhruman Goradia
Olga Goransson
Alexander Gorban
Marina Gorbatyuk
Oleg Gorbatyuk
Alex Gorbonos
Iwona Gorczynska
Victor Gordeuk
Heather Gordish-Dressman
Federico Gordo
David Gordon
Deborah Gordon
Grant Gordon
Howard S. Gordon
John Gordon

Jonathan Gordon
Karen Gordon
Keith Gordon
Leo Gordon
Louisa Gordon
Marion Gordon
Morris Gordon
Ross Gordon
Scott Gordon
Stephen Gordon
J.L. Gore
Jeff Gore
Jesse Gore
Aleksandra Górecka-Bruzda
Daniel Gorelick
David Gorelick
Fred Gorelick
Itamar Goren
Nora Goren
O. Goren
Larry Gorenflo
Marelize Gorgens
Eric Görgens
Martin Gorges
Ashraf Gorgey
Chris Gorgolewski
Ronald Gorham
Alessio Gori
Monica Gori
Simone Gori
H. Ulrich Göringer
Shela Gorinstein
Renata Gorjão
Gregor Gorkiewicz
Jamie Gorman
Shelley Gorman
Eamonn Gormley
Isobel Gormley
Kevin Gormley
German Gornalusse
Krzysztof Gornik
Sebastian Gornik
Nico Görnitz
Oksana Gorobets
Guy Gorochov
Jan Gorodkin
Jorg Goronzy
Myriam Gorospe
Rena Gorovits
Matt Gorr
Juan Gorraiz
Andrea Gorrell
Sasha Gorrell
Josef Görres
Andrew Gorringe
Magdalena Gorska
David Gorski
Andrzej Górski
Christian Gortazar
Ankush Gosain
Marko Gosak
Shachi Gosavi
Reinoud Gosens
Kirk Gosik
Roland Gosling
Yvan Gosmain
David Goss
Erica Goss
Kara Goss
Julie Gosse
Laure Gossec
Edmund Gosselin
Sophie. Gosselin
Philippe Gosset
Dana Gossett
Toni Gossmann
Celine Gossner
Johanna Gostner
Achintya Mohan Goswami
Varun Goswami
Carolin Gotay
Yoav Gothilf
Atsushi Goto
Katsumasa Goto
Koji Goto
Shin Goto
Tadahiro Goto
Hiroki Gotoh
Naoko Goto-Inoue
Nicole Gottdenker
Martin Gotte
Martin Götte
Joel Gottesfeld
Michael Gottesman
Natalia Gottig
Geoffrey Gottlieb
Yuval Gottlieb
John Gottsch
Alan Gottschalk
Michael Gottschalk
William Gottschalk
Paul Gottschall

Friedrich Götz
Jens Peter Gøtze
Lars Götzenberger
Hans Götzsche
Shaohua Gou
Xiaohua Gou
Louis Gouagna
Bruno Goud
Maryam Goudarzi
Anneke Goudriaan
Steven Goudy
Christopher Gough
Maria Gougouli
Catarina Goulao
Luis Goulao
Luis F Goulao
Cibelly Goulart
Alison Gould
Dinah Gould
Kathleen Gould
Rebecca L. Gould
Robert Gould
Todd Gould
Celia Goulding
James Goulding
Henri Goulet
Sacha Goultiaev
Dimitrios S. Goumenos
Gunnar Gouras
Guillaume Gourcerol
Robert Gourdie
Alexander Gourine
Pierre-Antoine Gourraud
Richard Gourse
Ivan Gout
Nadege Goutagny
Romain Goutagny
Jérôme Gouttenoire
Nikolaos Gouvas
Ricardo Gouveia
Sonia Gouveia
Mieke Gouwy
Valerie Gouyer
Danielle Goveia
Fabio Governato
Francine Govers
Chhabi Govind
Varan Govind
Velu Govindan
Brandon Govindarajoo
Kasivelu Govindaraju
Darshini Govindasamy
Govindjee Govindjee
Elena Govorkova
Vadim Govorun
Andrew Govus
Chien-Hung Gow
Timothy Gowan
Gopal Gowane
Anilkumar Gowda
Chandrika Gowda
Charitha Gowda
Juan Gowda
Kymberly Gowdy
Jim Gower
Humaira Gowher
Josh Gowin
Penny Gowland
Uthaman Gowthaman
Aaksh Goyal
Madhav Goyal
Mayank Goyal
Neeraj Goyal
Rajiv Goyal
Jana Goyens
Aymeric Goyer
Illana Gozes
Jeremy Grabbe
Michael Grabchak
Marco Grabemann
Rebecca Graber
Bryan Grabias
John Grable
Daniel Grabner
Roland Grabner
Igor Grabovac
Marcin Grabowicz
Przemyslaw Grabowicz
Mark Grabowski
Peter Grabowski
Vincent Gracco
Katherine Grace
Kathryn Grace
Mike Grace
Sheryl Gracewski
Elena Gracheva
Enrique Gracia
Luis Gracia
Carlos Gracia-Lazaro
Miguel Graciano
Carolina Gracitelli
Aaron Grade

Sergio Gradilone
Michael Gradisar
Ana Gradissimo
Luigi Gradoni
Gérard Gradwohl
Sue Grady
Daniel Graeber
Martin Graef
Christine Graf
Erika Graf
Ethan Graf
Joerg Graf
Kelly Graf
Peter Graf
Rolf Graf
Wilhelm Graf
Christian Graff
Rebecca Graff
Jan Graffelman
Loren Gragert
Benjamin Graham
Carolyn W. Graham
David Graham
E. Scott Graham
Eva Graham
Reiko Graham
Ryan B. Graham
Terry Graham
John Grahame
Nicholas Grahame
Jennifer Graham-Engeland
Florian Grahammer
Louise Grahnemo
Konstantia Graikou
Benoit Graillot
Ian Grainge
David Grainger
Marie Grall-Bronnec
Klaus Gramann
Laura Gramantieri
Maria Gramatges
Boris Gramatikov
Marina Gramiccia
Greta Gramig
Vera Gramigna
Paola Grammatico
Astrid Grams
Maricel Graña Grilli
Miriam Granado
Daniel Granato
Ann Grand
Marie-Angèle Grandbastien
Anjana Grandhi
Eleonora Grandi
Giuseppe Grandi
Laura Clara Grandi
Louis Grandjean
Valérie Grandjean
Scott A. Grandy
Marcus Granegger
Érika Graner
Roser Granero
A. Granfeldt
Cristina Grange
Jacques Grange
Thierry Grange
Christophe Grangeasse
Thalles Grangeiro
Christopher Granger
Julie Granger
Oleg Granichin
Ludomira Granicka
Sophie Granier
Juan R. Granja
Ben Grannan
Fred Grannis
Caterina Grano
Maria Grano
Michal Granot
Zvi Granot
Erik Granquist
Heather Gransee
Andrew Grant
Anthony Grant
Evan Grant
Lindsay Grant
Michael Grant
Paul Grant
Richard Grant
Stephen Grant
Suzanne Grant
William Grant
Jared Grantham
Ana Granville-Garcia
Cristina Granziera
Serena Granziera
Alessandro Grapputo
Jorge Grasa
Corinna Grasemann
Hartmut Grasemann
Felix Grases
Juris Grasis

Beatriz Gras-Miralles
Robert Grass
Susanne Grässel
Christian Grasshoff
Claudio Grassi
Fabrizio Grassi
Massimo Grassi
Dora Grassi-Kassisse
Guntram Grassl
Andre Grassmann
Felix Grassmann
Ludovica Grasso
Michal Grat
Jonathan Gratch
Caterina Gratton
Maud Gratuze
Alois Gratwohl
Cai Grau
James Grau
Jim Grau
Nadine Graubardt
Michael Graupner
Stavros Gravas
Sarah Gravem
Barbara Gravendeel
Dana Graves
Edward Graves
Stephen Graves
Steven Graves
Michael Gravett
Maria Flavia Gravina
Janko Gravner
Jochen Graw
Debra Gray
Dennis Gray
Kurt Gray
Linsay Gray
Meeghan Gray
Miranda Gray
Morgan Gray
Richard Gray
Rob Gray
Stephen Gray
Steven Gray
Eva Grayck
Donald Grayson
Alexander Grayver
Giovanni Grazioso
Anna T. Grazul-Bilska
Madeleine Grealy
Mary Greaney
Jane Greatorex
David Greaves
Erin Greaves
Thomas Greb
Godfrey Grech
Andrea Greco
Azzura Greco
Brian Greco
Claudio Greco
Frank Greco
Luigi Greco
Massimiliano Greco
Nancy Greco
John Grecula
Sara Gredmark Russ
Alan Green
Barry Green
Bart Green
Beverly Green
Daniel Green
Dannielle S. Green
David Green
Jennifer Green
Jeremy Green
Judith Green
Kim Green
Michael Green
Sarah Green
Sol Green
Timothy Green
Katharine Greenaway
Eli Greenbaum
Alan Greenberg
David Greenberg
Kenneth Greenberg
Miriam Greenberg
Richard Greenberg
Joel Greenberger
David J. Greenblatt
Catherine Greene
Ciara Greene
Correigh Greene
Ernest Greene
Joshua Greene
Mark Greene
Michael Greene
Michelle Greene
Naomi Greene
Nicholas Greene
Stephanie Greene
Talya Greene

William Greene
Alex Greenfield
Ben Greenfield
Edward Greenfield
Michael Greenfield
Paul Greenhaff
David Greenhalgh
Scott Greenhalgh
Stuart Greenhill
Julia Green-Johnson
Matthew Greenlees
C. Greenlief
Sarah Greenman
Anne Greenough
Thomas Greenough
Penelope J. M. Greenslade
Matt Greenstone
Aaron Greenville
Frank Greenway
Matthew Greenwold
Alex Greenwood
Brian Greenwood
Daniel Greenwood
Edward Greenwood
Matthew Greenwood
Michael Greenwood
Beau Greer
Charles Greer
Joe Greet
Robert Greevy
Giuliana Gregato
Dale Gregerson
Lauro Jose Gregianin
Marcos Gregnani
Sonia Grego
Cesare Gregoretti
Giovanni Gregori
Matjaž Gregoric
Lesley Gregoricka
Bianca Gregorio
Andrew Gregory
Anthony Gregory
Christopher Gregory
Richard Gregory
T. Gregory
Irene Gregory-Eaves
Meredith Gregory-Ksander
Celia Gregson
Duncan Greig
Nigel Greig
Thomas Greig
Steffen Greilich
Andreas Greinacher
Andreas Greiner
Kenneth Greis
Gorm Greisen
Michelle Greiver
Timothy Greives
Sasha Grek
Anna Greka
Rosa Grembiale
Jennifer Gremer
Hendrik Gremmels
Ryszard Grenda
Marián Grendár
Ruth Grene
Bryan Grenfell
Pippa Grenfell
S.E. Grenfell
Alexander Greninger
Troy Grennan
Melissa Gresle
Gilbert Greub
Harleen Grewal
Finn Grey
Jonathan Grey
Giorgia Gri
Jay Gribble
Elizabeth Grice
James Grice
Thomas Gridley
Melanie Grieb
Anne Griep
Yannick Griep
Tyson Grier
Oliver Griesbeck
Uta Griesenbach
Juergen Grieser
Christoph Griessinger
David Grieve
Gregg Griffenhagen
Amy Griffin
Courtney Griffin
Darren Griffin
Frank Griffin
James Griffin
Kristen Griffin
Lewis Griffin
Lisa Griffin
Marie Griffin
Michael Griffin
Nicholas Griffin

Russell Griffin
Mari Griffioen
Boyce Griffith
Daniel Griffith
Gareth Griffith
Jack Griffith
Kevin Griffith
Malachi Griffith
Simon Griffith
Thomas Griffith
Anthony Griffiths
Caroline Griffiths
Emily Griffiths
Mansel Griffiths
Mark Griffiths
Dominique Griffon
Tomas S Grigera
Ryan Griggs
Stefano Grignolio
Michel Grignon
Paolo Grigolini
Linda Grigoraki
Elena Grigorenko
Boyan Grigorov
M. Grigoryan
Sergei Grigoryev
Perry Grigsby
Carlos Grijalva
Eva Grill
Jessica Grill
Joshua Grill
Jacopo Grilli
Luciane Grillo
Renato Grillo
Antoine Grillon
Gianluca Grimalda
D. Grimaldi
Francisco Grimaldo
Katerina Grimani
Catherine Grimes
H. Leighton Grimes
Shelley Grimes
Alexander Grimm
Bernhard Grimm
Marcus Grimm
Sabine Grimm
Timo Grimmer
Eleni Grimpampi
Gina Grimshaw
Julia Grimwade
Alex Grinberg
M Grinholc
Mariusz Grinholc
Frederick Grinnell
Steven Grinspoon
Kristen Grinstead
Konstantinos Grintzalis
Helena Grip
Jonathan Grip
Karen Gripp
Enrico Grisan
Olaf Grisk
Mark E. Grismer
Laura Grisotto
Cortland Griswold
Michael Griswold
Valeriya Gritsenko
Ulrike Grittner
Igor Griva
Jean-Charles Grivel
Koni Grob
Leanne Groban
Justin Grobe
Nadja Grobe
Anna Grochot-Przeczek
Lucie Grodecká
Florian Groeber
Ulrike Groemping
Albert Groen
Ewout Groen
Harry Groen
Iris Groen
Thomas Groen
Martien Groenen
Eildert Groeneveld
Mathijs Groeneweg
Alexander Groffen
Dennis Grogan
Alexander Groh
Janos Groh
Patrick Grohar
Elisabeth Grohmann
Markus Grohme
Guillaume Grolez
Andreas Groll
M. Michael Gromiha
Gregory Gromowski
Wulfila Gronenberg
Detlef Gronenborn
Maya Groner
Claude Gronfier
Christian Grønhøj Larsen

Hunter Groninger
Sebastian Grönke
Line Grønning-Wang
Gloria Gronowicz
Tove Grönroos
Wolfram Gronwald
Leif Groop
Astrid Groot
Maartje Groot
Patrick Grootaert
Hilke Grootelaar
Paul Grootendorst
Ton Groothuis
Roswitha Gropp
Michael Gropper
Philippe Gros
Stjepan Gros
Alexander Grosberg
Chester Grosch
Antje Grosche
Kasper Grosen
Allison Groseth
Henri Grosjean
Julie Gros-Louis
Xavier Grosmaitre
Aaron Gross
Alan Gross
Alden Gross
Alecia Gross
Alexander Gross
Andrin Gross
Briana Gross
Catharina Gross
Christine Gross
Christopher E. Gross
Gideon Gross
Harald Gross
Jeff Gross
Josef Gross
Joshua Gross
Markus Gross
Raphael Gross
Steven Gross
Tom Gross
Volker Gross
Hans-Peter Grossart
Stephen Grossberg
Ian Grosse
Robert Grosse
Scott Grosse
Christine Große-Brinkhaus
A. V. Grossestreuer
Jean-Francois Grosset
Ewald Grosse-Wilde
Marco Grossi
Dominik Grosskinsky
Julian Grosskreutz
Daniel Grossman
Guy Grossman
Mathis Grossmann
Jessica Grossmeier
Dolores Grosso
Giuseppe Grosso
Ludger Grote
Rüdiger Grote
Chad Grotegut
G. Groth
Huw Groucutt
Miranda Grounds
Anne Grove
Sara Grove
Abhinav Grover
Ajay Grover
Ashok Grover
Martha Grover
Sandeep Grover
Sonam Grover
Andy Groves
Colin Groves
Mitchell D. Groves
Christina Grozinger
Janice Grskovic
Nathan Grubaugh
Susanne Grube
Ludwig Gruber
Reinhard Gruber
Harald Gruber-Vodicka
Lisa Grubisha
Martin Grübler
Catherine Grueber
Sonja Gruen
Johannes Grueneisen
Samantha Gruenheid
Viola Grugni
Jelena Grujic
Rebecca Grumet
Olga Grum-Grzhimaylo
Janie Grumley
Ruth Grümmer
Lawrence Grummer-Strawn
Dan Grun
Walter Grünberg

Carsten Gründemann
Oliver Grundmann
Christoph Grundner
Richard Grundy
Tilman Grune
Katarzyna Grunt-Mejer
Gerald Grunwald
Larry Gruppen
Philip Gruppuso
Forest Gruss
Andreas Grützkau
John Gruzelier
Ryszard Grygorczyk
Ouriel Grynszpan
Rafael Grytz
Dariusz Grzebelus
Raphael Grzebieta
K. Grzelkowska-Kowalczyk
Andrzej Grzybowski
Tomasz Grzybowski
Anna Grzywacz
Maher Gtari
Chao Gu
Chen Gu
Dongfeng Gu
Dongmin Gu
Fei Gu
Feng Gu
Haifeng Gu
Huaguang Gu
Jian Gu
Jianying Gu
Ji-Dong Gu
Jin Gu
Keyu Gu
Leyi Gu
Lijuan Gu
Luo Gu
Peili Gu
Qilin Gu
Wan-Jie Gu
Weikuan Gu
Xianhong Gu
Xiaoli Gu
Xiaolian Gu
Xiaowu Gu
Yian Gu
Ying Gu
Yumei Gu
Zhen Gu
Amelia Guadalupe-Grau
Antonio Guaita
Michele Guala
Bruno Gualano
Maria Gualano
Fabio Gualtieri
Maurizio Gualtieri
Mirella Gualtieri
Feng Guan
Lan Guan
Min Guan
Shane Guan
Shengxi Guan
Tao Guan
Weihua Guan
Wenjian Guan
Xiangmin Guan
Xin-Yuan Guan
Xuefeng Guan
Yongqiang Guan
Yongtao Guan
Yue Guan
Frederico Guanais
Fu Guangmiao
Li Guanwu
Pietro Guaraldi
Blake Guard
Jean Guard
Ornella Guardamagna
Joan Guàrdia-Olmos
Christine Guardino
Simone Guareschi
Manuel Guariguata
Fabio Guarino
V. Guarino
Jeannette Guarner
Alessandra Guarneri
Biancamaria Guarnieri
Michael Guarnieri
Fabio Guarracino
Ann Guassora
Luigina Guasti
Sandra Guauque-Olarte
Juan Manuel Guayasamin
Carmen Guaza
Larisa Gubareva
Marc-Jan Gubbels
Matthias Gube
Albert Gubern-Mérida
Tomasz Gubiec
Ernesto Guccione
Henk-Jan Guchelaar

Ewa Gucwa-Przepióra
Lorraine Gudas
Ramachandra Gudde
Severin Gudima
Aparna Gudlur
Gunnar Gudmundsson
Lukas Gudmundsson
Volker Gudziol
Jerome Guechot
Joana Guedes
Rubem Carlos Araujo Guedes
Vivian Guedes
Eric Guedj
Jeremie Guedj
Romain Guedj
Jean-Pierre Gueffet
Naïg Gueguen
Frederico Gueiros-Filho
Faikah Gueler
Anis Guelmami
Charles Guenancia
Sylvie Guenette
Dominik Guensch
Matthias Guenther
Sebastian Guenther
Marina Guenza
Natacha Gueorguieva
Alfredo Guéra
Yann Guerardel
Mireia Guerau-de-Arellano
Chloé Guerbois
Pierre Gueriau
Jean-François Guerin
Pierre Guermonprez
Carmen Guerra
Concettina Guerra
Eliete Guerra
Javier Guerra
Marcelo Guerra
Miguel Guerra
Rudy Guerra
Richard Guerrant
Anthony Guerrero
Cesar Guerrero
Erick Guerrero
Jose Guerrero
Lourdes R. Guerrero
Eloisa Guerrero Barona
Hugo Guerrero-Cazares
Antonieta Guerrero-Plata
Rafael Guerrero-Preston
Alfredo Guerreros
Michele Guerreschi
Christopher Guerriero
Patricia Guerry
Benoit Guery
Andrew Guess
James Guest
Ramon Guevara
Ramon Guevara Erra
Miguel Angel Guevara Lopez
Laetitia Guevel
Nicolas Guex
Francois Gueyffier
Muriel Gugger
G. Guggino
William Guggino
Eugenio Guglielmelli
Jean Guglielminotti
Bernard Guglielmo
Letterio Guglielmo
Rajarshi Guha
Sonia Guha
Udayan Guha
Jian-Fang Gui
Jie Gui
George Guibas
Christelle Guibert
Jean Guibourdenche
Marco Guicciardi
Elisa Guida
Natascia Guida
Stefano Guido
Giovanna Guidoboni
Alessandro Guidotti
F. Guidozzi
Bruno Guigas
Adrienne Guignard
Vincent Guigonis
Freddy Guiheneuf
Beatriz Guijarro
Romain Guilhaumou
Orane Guillaume-Gentil
Rhian Guillem
Matthieu Guillemain
Marilys Guillemin
Christian Guilleminault
Jordi Guillen
Carlos Guillén
Julie Guillermet-Guibert
Lauren Guillette
Martin Guilliams

Maude Guillier
Christophe Guillon
Quentin Guillon
Aymeric Guillot
Martin Guillot
Hervé Guillou
Yannick Guilloux
Claudia Guimaraes
Roger Guimerà
Martin Guimond
John Guinan
Claire Guinat
Valeria Guinder
Josee Guindon
Stephane Guindon
Sarah Guindre-Parker
Benjamin Guinhouya
Jose Guinot Saporta
Joan Guinovart
Ruth Guinsburg
Nuno Guiomar
Peng Guiqing
Ricardo Guiraldo
Amélie Guitart
Pascale Guitera
Pascale Guiton
Marina Guizetti
Unjali Gujral
Anna Gukovskaya
Taza Gul
Ashu Gulati
Gaurav Gulati
Omer Faruk Gulban
Gundega Gulbe
Per Guldberg
Nurdan Güldiken
Ali Guler
Reto Guler
Rakeshwar Guleria
Andrew Gulick
Vladislav Gulis
Penny Gullan
Frances Gulland
S. Guller
Francesca Gullo
Alexander Gultyaev
Andras Gulyas
Bertil Gummeson
Jonathan Gumucio
Yosephine Gumulya
Alper Gumus
Koray Gumus
M Gumusay
Michelle Gumz
Karthigayan Gunalan
Don Gunasekera
Hasantha Gunasekera
Rudiyanto Gunawan
Manjula Gunawardana
Shermali Gunawardena
Ersin Gunay
Gul Gunaydin
Hediye Gunbey
Sampath Gunda
Nina Gunde-Cimerman
Craig Gundersen
Vidar Gundersen
A. Gunderson
Lee Gunderson
Ursula Gundert-Remy
Rebekah Gundry
Aysegul Gunduz
Mehmet Gunes
Monica Gunetti
Emre Guney
Akash Gunjan
Yurii Gun'ko
Alistair Gunn
Anne Gunn
Ingibjorg Gunnarsdottir
Gunnar Gunnarsson
Yanni Gunnell
Purnima Gunness
William Gunning
Faith Gunning-Dixon
Emma Guns
Raf Guns
Olivia Guntarik
Lisa Gunter
Stacey Gunter
Thomas Gunter
Huldrych Günthard
Catrin Gunther
Orlando Guntinas-Lichius
Jenny Gunton
Richard Gunton
Saketeh Guntupalli
Philipp Gunz
Dorothee Günzel
Arthur Gunzl
Aizhen Guo
Beichu Guo

Bin Guo
Caixia Guo
Changkui Guo
Changrun Guo
Chao-Yu Guo
Cheng-Xian Guo
Chih-Hung Guo
Chuanyong Guo
Chun Guo
D Guo
Da-Long Guo
Dan Guo
Danhuai Guo
Daqing Guo
Enen Guo
Fangjian Guo
Fei Guo
Fen Guo
Feng Guo
Fengjin Guo
Fu Guo
Grace Guo
Haitao Guo
Hongbin Guo
Hou-Fu Guo
Huan Guo
Hui Guo
Jia Guo
Jianyang Guo
Jing Guo
Jingxin Guo
Junming Guo
Ju-Tao Guo
Laodong Guo
Lei Guo
Li Guo
Lifei Guo
Lin Guo
Longbiao Guo
Maozu Guo
Mei-Hui Guo
Min Guo
Ming Guo
Mingzhou Guo
Peng Guo
Pengfei Guo
Qiiang Guo
Qinfeng Guo
Qing Guo
Qinglan Guo
Ren Guo
Ren-Yong Guo
Shanshan Guo
Shaodong Guo
Shiwei Guo
Song Guo
Songchang Guo
Sun-Wei Guo
Wangzhen Guo
Wei Guo
Weidong Guo
Weilong Guo
Wenbin Guo
Wenhu Guo
Wenwu Guo
Xiao Guo
Xiaohui Guo
Xiaojuan Guo
Xiaoming Guo
Xijie Guo
Xinxing Guo
Xinyu Guo
Yanrong Guo
Yan-Yan Guo
Yi Guo
Ying Guo
Youmin Guo
Yunliang Guo
Yunqian Guo
Zaoyang Guo
Zhangbao Guo
Zhaojiang Guo
Zhefeng Guo
Zhiqiang Guo
ZhongMao Guo
Zhongxin Guo
Jin Guoliang
Fabio Guolo
Jiang Guoquan
F.L. Gup
Aditi Gupta
Aditya Gupta
Ajay Gupta
Akhilendra Gupta
Amit Gupta
Amita Gupta
Amod Gupta
Anil Gupta
Archana Gupta
Bhaskar Gupta
Devanand Gupta
Dipika Gupta

Divakar Gupta
Gopal Gupta
Himanshu Gupta
Kalpna Gupta
Madhulika Gupta
Manveen Gupta
Neetu Gupta
Nidhi Gupta
Nishant Gupta
Pawan Gupta
Prakash Gupta
Rajeev Gupta
Rajesh Gupta
Rani Gupta
Ravi Gupta
Rishi Gupta
S.K. Gupta
Sanjay Gupta
Sanjeev Gupta
Santosh Gupta
Sarita Gupta
Shailendra Gupta
Shishir Gupta
Subash Gupta
Surya Gupta
Tanush Gupta
Vikrant Gupta
Vinod Gupta
Vivek Gupta
Ankur Gupta-Wright
Anisha Gupte
C. Guptill
Rob Guralnick
Ali Gure
Slava Gurev
Eugenia Gurevich
Michael Gurevich
Irina Gureviciene
Jessica Gurevitch
Helen Gurgel
Ricardo Gurgel
Artur Gurgul
Nathalie Guriec
Kelly Gurka
Umut Gurkan
James Gurney
Uxia Gurrarian Rodriguez
Thomas Gurry
Olga Gursky
Volker Gurtler
Kotambylu Vasudeva Gururaja
Anand Gururajan
Mayank Gururani
David Gurwitz
Jonathan Guryan
Esteban Gurzov
Niraj Gusani
Vladimir Gusarov
Viktoria Gusarova
Franco Guscetti
Tharina Guse
Nikolai Gusev
Narcis Gusi
Leonor Gusmão
Dimitri Gusmao-Flores
Markus Gusset
Kurt Gust
Christopher Gustafson
Daniel Gustafson
Kathleen Gustafson
Keith Gustafson
Jan-Ake Gustafsson
Jeff Gustin
Agneta Gustus
Ian Gut
Nils Gutacker
Ryan Gutenkunst
Elan Guterman
Arvid Guterstam
Amanda Guth
Nagesha Guthalu Kondegowda
Martina Guthoff
O'neil Guthrie
Sally Guthrie
Alejandro Gutierrez
Claude Gutierrez
Crisanto Gutierrez
Daniel Gutierrez
Gabriel Gutierrez
Gregory Gutierrez
Jose Gutierrez
Maximiliano Gutierrez
Osman Gutierrez
Peter Gutierrez
Roberto Gutierrez
Juan Gutiérrez
Orlando Gutiérrez
Antonio Gutierrez de Blume
Alfonso Gutierrez-Adan
Mario Gutiérrez-Bedmar
Irene Gutiérrez-Cañas
Cayetano Gutiérrez-Cánovas

Carmina Gutiérrez-González
Roger Gutierrez-Juarez
Jose Gutierrez-Maldonado
Mario Gutiérrez-Roig
Kelly Gutpell
Julian Gutt
Adrian Gutteridge
Emine Guven
David Guwatudde
Jon Guy
Jonathan Guy
Robert Guy
Olivier Guyader
Gordon Guyatt
Christophe Guyeux
David Guyton
Frank Guzman
Jose Guzman
Raul Guzman
Bernardo Guzman Herrador
Ernesto Guzman-Novoa
John F. Guzowski
Goran Gužvica
Diana Guzys
Mariangela Guzzardi
Rosa Guzzo
Hila Gvirts
Yousang Gwack
Marianne Gwechenberger
Le Teuff Gwenael
Goude Gwenaëlle
Rodger Gwiazdowski
Laura Gwilliams
Wilfried Gwinner
Carole Gwizdek
Chaubey Gyaneshwer
Adam Gyedu
Klara Gyires
Charlotte Gyllenhaal
Sandor Gyorke
Attila Gyucha
Chien Ha
Hunjoo Ha
In-Hyuk Ha
Jin-Yong Ha
Jong-Won Ha
Kwon-Soo Ha
Kyoo-Man Ha
Nam-Chul Ha
Sehoon Ha
Seung Ha
Sol Ha
Suk-Jin Ha
Brian Haab
Günter Haag
David Haaga
Michel Haagdorens
Juanita Haagsma
Chris Haak
Scott Haake
Wieke Haakma
Benjamin Haaland
Rita Haapakoski
Markku Haapamäki
Markus Haapasalo
Jarrod Haar
Alexander Haas
Brian Haas
David Haas
G. Haas
Kalina Haas
Karen Haas
Michael Haas
Mitchell Haas
Stefan Haas
Verena Haas
Wilhelm Haas
Astrid Haase
Hajo Haase
Steven Haase
Tina Haase
Burkhard Haastert
Ylva Haasum
Marc Habash
Antje Habekuss
Michael Haber
Georg Haberer
Sabina Haberlen
Tilmann Habermas
Abdulrazaq Habib
Bilal Habib
Michael Habib
Muhammad Atif Habib
Nadia Habib-Bein
Maryam Habibi
Mahmood Habibiyan
Gregor Habl
Mounira Habli
Angelita Habr-Gama
Dereje Habte
Mussie Habteselassie
Chizu Habukawa

Satoshi Hachimura
Akira Hachiya
Helena Hachul
Piraye Haciguzeller
Gokce Hacioglu
Jochen Hack
David Hackam
Mathias Hackenbroch
F. Hacker
Hans Häcker
Katie Hackett
Maree Hackett
Troy Hackett
Anthony Hackney
David Hackos
Ted Hackstadt
Franz Hadacek
Farzad Hadaegh
Liat Hadar
Yitzhak Hadar
Esmaiel Hadavandi
Luciana Haddad
Nick Haddad
Steven Haddock
David Haddon
James Haddow
Lewis Haddow
Erinn Hade
Donat Häder
Nyssa Hadgraft
Naila Hadi
Raafat Hadi
Samy Hadjadj
Fadila Hadj-Bouziane
Christos Hadjichristodoulou
Alkis Hadjiosif
James Hadler
David Hadley
Narelle Hadlow
Jacob Hadnett-Hunter
Heike Hadrys
Emir Hadzijusufovic
Udo Haecker
Walter Haefeli
Saskia Haegens
Sebastian Haen
Melissa Haendel
Gregory Haenel
Ronny Haenold
Beate Haertel
Thomas Haertle
Julie Haesebaert
Aaron Haeusler
Daniel Haeusser
Julius Clemence Hafalla
Gaudenz Hafen
Carolyn Hafer
Ali Hafezi-Moghadam
Gregory Haff
Christof Haffner
Michael Haffner
Sepehr Hafizi
Louise Hafner
Iva Hafner-Bratkovic
Gertrud Hafstad
Susanne Haga
Holly Hagan
Iain Hagan
Suzanne Hagan
Chadi Hage
Monica Hagedorn
Edward Hagen
Ferry Hagen
Knut Hagen
Kristin Hagen
Marco Hagen
T.J. Hagenaars
Rachel Hagey
Gareth Hagger-Johnson
Kerstin Häggqvist
Olle Häggström
AliAkbar Haghdoost
Ghazaleh Haghiashtiani
Shahpar Haghighat
Aiden Haghikia
Daisuke Hagiwara
Masatoshi Hagiwara
Cornelia Hagl
Michael Haglund
York Hagmayer
Stefanie Hagner-Benes
William Hagopian
Ken Haguenoer
James Hagy
Hyeouk Hahm
Torsten Hahmann
Andreas Hahn
Christopher Hahn
Daniel Hahn
Judith Hahn
Juergen Hahn
Mariah Hahn

Matthias Hahn
Sinuhe Hahn
Tim Hahn
Ulf Hahnel
Jennifer Hahn-Holbrook
Richard Hahnloser
Amy Hahs
Michael Hahsler
Chi-Ming Hai
Rong Hai
Vu Ngoc Hai
Guillaume Haiat
Hossam Haick
Ghady Haidar
Malak Haidar
Haula Haider
Khawaja Haider
Rukhsana Haider
Paul Haidet
Cathryn Haigh
Marcia Haigis
Candace Haigler
Shalin Hai-Jew
Frank Hailer
Bereketeab Haileselassie
Asrat Hailu
Abraham Haim
Niina Haiminen
Ariel Haimovici
James Hain
Helen Haines
Russell Haines
Terrance Haines
Atticus Hainsworth
Bridget Haire
Carl Haisch
Nitai Hait
Adrian Haith
Behzad Hajarizadeh
Eric Hajduch
André Hajek
Parvana Hajieva
Inaya Hajj Hussein
Waseem Hajjar
Mustapha Hajjou
Mihaly Hajos
Regine Hakenbeck
Samer Hakim
Vincent Hakim
Morgan Hakki
Hakon Hakonarson
Helle Håkonsen
Christian Hakulinen
Gwen Halaas
Ajay Halai
Eliran Halali
Anna Halama
Niels Halama
Mohammad Halami
John Halamka
A. Halaris
Natasha Halasa
Tariq Halasa
Andrew Halayko
Demetrios Halazonetis
Richard Halberg
Chanita Halbert
Erin Hald
Devyani Haldar
Jayanta Haldar
Sagarika Haldar
Ingfrid Haldorsen
Iago Hale
Laura Hale
Lauren Hale
Melina Hale
Melissa Hale
Abdul Haleem
Derek Hales
Andrew Halestrap
Guillaume Halet
Andreana Haley
Danielle Haley
Nicholas Haley
William Haley
Ole Halfdan Larsen
Nigel Halford
Stephanie Halford
Eni Halilaj
Nafisa Halim
Jean-Michel Halimi
Sofia Halin Bergström
Sinan Haliyo
Arja Halkoaho
Anne Hall
Bradley Hall
Brian Hall
Cameron Hall
Carina Hall
Carol Hall
Chad Hall
Charles Hall

Charlotte Hall
David Hall
Deborah Hall
Edward Hall
Emily Hall
Eric Hall
Frank Hall
Gerod Hall
Howard Hall
James Hall
Jennifer Hall
Jeremy Hall
Julian Hall
Mark C Hall
Marlous Hall
Matthew Hall
Michael Hall
Myrna Hall
Peter Hall
Robert Hall
Roy Hall
Stephen Hall
Steven Hall
Wayne Hall
Wendy Hall
Nadim Hallab
Joelle Hallak
Dean Hallam
Jennifer Hallam
Vipin Hallan
Joshua Hallas
Mryka Hall-Beyer
Thorhildur Halldorsdottir
Gustaaf Hallegraeff
Jostein Hallén
Heather Hallen-Adams
Maria Haller
Bernard Hallet
Paul Hallett
Richard Hallett
Julianne Halley
Gene Hallford
Kevin Hallgren
Oskar Hallgren
David Halliday
Bassam Hallis
William Hallman
Christian Hallmann
Bernard Halloran
Gayle Hallowell
Adrienne Hall-Phillips
Michael Hallquist
Serena Halls
Jason Hall-Spencer
Timothy Hallstrom
Luke E. Hallum
G. Michael Halmagyi
Michael Halmagyi
Sandra Halonen
Israel Halperin
James Halperin
John Halperin
Andrea Halpern
Casey Halpern
Malka Halpern
Melissa Halpern
Stacey Halpern
Patrick Halpin
Stuart Halse
Eric Halsey
Kimberly Halsey
Lewis Halsey
Scott Halstead
Mary Halter
Jacqueline Halton
Arda Halu
Marc Halushka
Halvor Halvorson
Ineke Ham
Jong Hyun Ham
Aldric Hama
Dennis Hamacher
Samera Hamad
Masakazu Hamada
Masashi Hamada
Ali Hamadeh
Issam Hamadeh
Randah Hamadeh
Masatsugu Hamaji
Nobuyuki Hamajima
Takeshi Hamamura
Dörte Hamann
Johannes Hamann
Mark Hamann
Takayuki Hamano
Alain Hamaoui
Karim Hamaoui
Juho Hamari
Yoshifumi Hamasaki
Chisato Hamashima
Rukman Hamat
Ridha Hambli

Esther Hamblion
K. David Hambright
Anne Hamburger
Malika Hamdane
Zoya Hameed
Candyce Hamel
Richard Hamelin
Davidson Hamer
Gabriel Hamer
Sarah Hamer
Raph Hamers
Karim Hamesch
Simon Hametner
Mash Hamid
Rizwan Hamid
Saeed Hamid
Mohammad Hamidaddin
Samer Hamidi
Blake Hamilton
Dale Hamilton
David Hamilton
Joshua Hamilton
Justin Hamilton
Marcus Hamilton
Thomas Hamilton
Trevor Hamilton
William Hamilton
Christian Hamilton-Craig
J. Kiley Hamlin
Robert Hamm
Sayamwong Hammack
Samar Hammad
Deepa Hammaker
Sarah Hamm-Alvarez
Christian Hammann
Karin Hammarberg
Harald Hammarström
Adam Hammer
Brian Hammer
Tobin Hammer
Alexander Hammers
Christoph Hammers
Margaret Hammerschlag
Kurt Hammerschmidt
Richard Hammersley
Mary Hammes
Bruce Hammock
Billy Hammond
Constance Hammond
Edward Hammond
Geoffrey Hammond
Gerry Hammond
Max Hammond
Scott Hammond
Sean Hammond
Evelynn Hammonds
Mohamed Hammoud
Sue Hammoud
Thierry Hamon
Abdul Hamood
Rifat Hamoudi
Arndt Hampe
Christiane Hampe
Jochen Hampe
Wolfgang Hampe
Beth Hamper
Greg Hampikian
Vladimír Hampl
Shalaka Hampras
Lynne Hampson
Lauren Hampton
Pedram Hamrah
Harald Hamre
James Hamrick
Mark Hamrick
Eliakim Hamunyela
Mouad Hamzaoui
Bing Han
Bo Han
Buhm Han
Chang Yeop Han
Dai Hoon Han
Daishu Han
Daphne Han
Dennis Han
Eun-Taek Han
Fangpu Han
Fei Han
Feng Han
Gencheng Han
Guohong Han
Haiyong Han
Hong-Xiang Han
Huili Han
Jae Woo Han
Jian Han
Jie Han
Jin Han
Jing Han
Jing-Dong Han
Jing-Yan Han
Jun Han

Kihwan Han
Kyungsook Han
Lanzhi Han
Li Han
Lide Han
Liebao Han
Lijun Han
Lin Han
Ling Han
Litao Han
May Han
Mingyuan Han
Peng Han
Quan-Bin Han
Sang Won Han
Sang-Sun Han
Sang-Wook Han
Seung Hyeok Han
Shanshan Han
Shengtong Han
Shiying Han
Song-I Han
Su Han
Sun-Young Han
Sushan Han
Tianxu Han
Weidong Han
Woong Kyu Han
Xiao Han
Xiaodong Han
Xiaohong Han
Xiaomei Han
Xiaopu Han
Xiao-Pu Han
Xiaosi Han
Xin Han
Xuemei Han
Xuesong Han
Xueying Han
Yeon Soo Han
Yifan Han
Ying Han
Yingying Han
Yong Han
Yuchi Han
Zheng Han
Zhenhai Han
Zhiming Han
Zongchao Han
Emily Han Shao
Kazuomi Hanada
M. Hafiz Hanafi
Manal Ibrahim Hanafi Mahmoud
Ahmad Ali Hanafi-Bojd
Norio Hanafusa
Ryuzo Hanaie
Ken-Ichi Hanaki
Hiroshi Hanamoto
Hironari Hanaoka
Kazushige Hanaoka
Stefania Hanau
David Hanauer
Merlin Hanauer
Yutaka Hanazono
Brice Hanberry
David Hanbury
Barry Hancock
C. Nathan Hancock
Dana Hancock
Lynn Hancock
Meaghan Hancock
Peter Hancock
Robert Hancock
Jenny Hand
Steven Hand
Suzanne Hand
Avtar Handa
Avtar K. Handa
Rajash Handa
Tomoya Handa
Nils Olav Handegard
Garry Handelman
D.J. Handelsman
Zafar Handoo
Stephan Handschuh
Todd Handy
Francis Hane
Helmut Hanenberg
Kurt Hanevik
Chad Haney
Bernd Hänfling
Chunhua Hang
Sandra Hangartner
Jürgen Hänggi
S.M. Hanifi
Pejman Hanifi Moghaddam
Patrick Hanington
Michael Hanke
Tomas Hanke
Mark Hankins
Emily Hankosky
Brent Hanks

Ephraim Hanks
Mark Hänle
Brian Hanley
Daniel Hanley
James Hanley
Kathryn Hanley
Torrance Hanley
Charlotte Hanlon
Erin Hanlon
Patrick Hanly
Alexander Hann
Hie-Won Hann
David Hanna
Jacob Hanna
Jandy Hanna
Philip Hanna
Kirsten Hannam
Anthony Hannan
David Hannapel
Marja-Liisa Hänninen
Eilis Hannon
Deborah Hannula
Jean-Baptiste Hanon
Colleen Hanrahan
Chetan Hans
Wilhelm Hansberg
Marc Hanschen
Rita Hansdorfer-Korzon
Eric Hanse
Anna Hansell
Alex Hansen
Anette Tarp Hansen
Benni Winding Hansen
Cecilie Hansen
Diana Hansen
Egon Hansen
Espen Hansen
Hans Hansen
Jason Hansen
Jeffrey Hansen
Marc Hansen
Mette Hansen
P. Hansen
Stephanie Hansen
Thor Willy Ruud Hansen
Thorsten Hansen
Tine Hansen
Wiebke Hansen
Simon Hanslmayr
Buck Hanson
Erik Hanson
Jarod Hanson
Jeffrey Hanson
Joshua Hanson
Kimberly Hanson
Paul Hanson
Jan Hanspach
Thor-Erik Sandberg Hanssen
Regina Hanstein
Constanze Hantel
Oliver Hantschel
Israel Hanukoglu
Chunyi Hao
Jijun Hao
Junwei Hao
Lingxin Hao
Ming Hao
Yue Hao
Yujiang Hao
Zhen-Zhen Hao
Zhuan-Fang Hao
Huang Haochu
James Haorah
Alexander Hapfelmeier
Janet Hapgood
Brenda Happell
Hapuarachchige Chanditha Hapuarachchi
Sudath Hapuarachchige
Inam Haq
Rizwan Ul Haq
Arsalan Haqqani
Abedul Haque
Azizul Haque
Mazharul Haque
Rashidul Haque
Azusa Hara
Hirokazu Hara
Hiromitsu Hara
Manami Hara
Takeshi Hara
Yuka Hara
Hisashi Harada
Ichiro Harada
Mamoru Harada
Naoki Harada
Naozumi Harada
Seiki Haraguchi
Takashi Haraguchi
Erin Haramoto
Takashi Haramura
Maciej Harat
Hayat Harati

Kengo Harato
Nina Harawa
Simon Harbarth
Stephan Harbarth
Kent Harber
Jeremy Harbinson
Sveinn Hardarson
Alison Hardcastle
Timothy Hardcastle
Nicholas Harden
Daniel Harder
David Harder
Jens Harder
Timm Harder
Grahame Hardie
Cary Harding
Eleanor Harding
Ian Harding
Scott Harding
Stephen Harding
Alex Hardisty
Oliver Hardt
Matthias Hardtke-Wolenski
Robert Hardwick
Philip Hardwidge
Elaissa Hardy
Giles Hardy
Ian Hardy
Richard Hardy
Rowan Hardy
Serge Hardy
Sheila Hardy
Dominic Hare
Jonathan Hare
Rowena Hare
T.N. Haregu
Roi Harel
Carla Harenski
Alan Hargens
Eszter Hargittai
Svasti Haricharan
Sajeet Haridas
Hideo Harigae
Nirmala Hariharan
K.B. Harikumar
Kaleeckal Harikumar
Nair R. Harikumaran
Robin Haring
Seenu Hariprasad
Sam Harirforoosh
Kusum Harjai
Mark Harker
Alex Harkess
Kari Harland
Carolyn Harley
David Harley
Naomi Harley
Guy Harling
Barbara Harlizius
Halil Harman
Wolf Harmening
Christopher Harmer
Clive Harmer
Jason Harmon
Julie Harmon
Mark Harmon
Thomas C Harmon
Cindy Harmon-Jones
Craig Harms
Kyle Harms
Lauren Harms
Bart Harmsen
Hermie Harmsen
Mats Harms-Ringdahl
Stevan Harnad
Chris Harnish
Ryan Harnish
Julian Harnoss
Jay Haron
Muhammad Haroon
Emily Haroz
Armie Harper
Elizabeth Harper
Matthew Harper
Nathan Harper
Sam Harper
Scott Harper
Willie Harper
Vincent Harraca
Balázs Harrach
Vanessa Harrar
Loic Harrault
Niki Harré
Frank Harrell
J. Chuck Harrell
Paul Harrell
Trisheena Harricharran
Jane Harries
Priscilla Harries
Paul Harrigan
Robert Harrigan
Ryan Harrigan

Elizabeth Harrington
Laura Harrington
Matt Harrington
Peter Harrington
Adam Harris
Anette Harris
David Harris
Edward Harris
Elizabeth Harris
Holly Harris
J. Harris
Janna Harris
Jenine Harris
John Harris
Julie Harris
Katie Harris
Keith Harris
Larry Harris
Laurence Harris
Lisa Harris
Lynda Harris
Magdalena Harris
Marion Harris
Mark Harris
Matthew Harris
Meagan Harris
Melissa Harris
Michael Harris
Neil Harris
Nicola Harris
Peter R. Harris
R. Alan Harris
Rachel J. Harris
Reid Harris
Reuben Harris
Richard Harris
Robert Harris
Ross Harris
Samantha Harris
Stephen Harris
Tajie Harris
Tony Harris
Helen Harris-Fry
Ben Harrison
Earl Harrison
Hugo Harrison
Jennifer Harrison
Jon Harrison
Margo Harrison
Maria Harrison
Patrick Harrison
Rene Harrison
Robert Harrison
Wendy Harrison
Lisa Harrison-Bernard
Katherine Harrisson
James Harrop
Jean Harrowing
Issifu Harruna
Alastair Harry
Elizabeth Harry
Laura Harsan
Adam Hart
David Hart
Jo Hart
Joanna Hart
Malcolm Hart
Prue Hart
Robbie Hart
Roger Hart
Sara Hart
Sarah Hart
Trevor Hart
Tibor Hartel
Volker Hartenstein
Tom Hartgill
Volker Harth
Sean Hartig
Juha Hartikainen
Andreas Hartkopf
Sylvia Hartl
Marek Hartleb
Carol Hartley
Geoffrey Hartley
Matthew Hartman
Sheri Hartman
Andreas Hartmann
Anton Hartmann
Boris Hartmann
Daniel Hartmann
Erica Hartmann
Henrik Hartmann
Jessica Hartmann
Petra Hartmann
Sonja Hartnack
Christiane Hartog
Peter Hartsough
Adam Hartstone-Rose
Robert Hartsuiker
Benno Hartung
Hans Peter Hartung
Matthew Hartwig

Saskia Hartwig
Werner Hartwig
G. Hartwigsen
Ronald Harty
H. Hartzell
Helia Harumi Sato
C. Harvell
Lindsey Harvell-Bowman
Alan Harvey
Ben Harvey
Brandon Harvey
Danielle Harvey
Erin Harvey
Hannah Harvey
Jeffrey Harvey
Kirsten Harvey
Lara Harvey
Michael Harvey
Nigel Harvey
Philip Harvey
Rebecca Harvey
Richard Harvey
Simon Harvey
R. Donald Harvey III
Emily Harville
Sarah Harvolk-Schoening
Jake Harwood
Thomas Harwood
Tim Harwood
Anne-Wil Harzing
Alan Harzman
Alkomiet Hasan
Baktiar Hasan
David Hasan
Gaiti Hasan
M. Kamrul Hasan
Md. Kamrul Hasan
Mizaton Hazizul Hasan
Qurratulain Hasan
Tayyaba Hasan
Uzma Hasan
Parisa Hasanein
Mahdi Hasanlou
Vincent C. Hascall
Wanda Haschek
Naoyuki Hasebe
Goji Hasegawa
Hideyuki Hasegawa
Junichi Hasegawa
Koji Hasegawa
Paul Hasegawa
Shunji Hasegawa
Takehisa Hasegawa
Yoshiaki Hasegawa
Yu Hasegawa
Jiri Hasek
Mark Haselgrove
Jan Hasenauer
Timothy Hasenoehrl
Jonathan Hasford
Farhad B. Hashemi
Parastoo Hashemi
Hesam Hashemian
Seyed Javad Hashemian
Dana Hashim
Hideki Hashimoto
Ken Hashimoto
Koshi Hashimoto
Kouichi Hashimoto
Motomu Hashimoto
Naohiro Hashimoto
Takashi Hashimoto
Takeshi Hashimoto
Yoshiaki Hashimoto
Hiroshi Hashizume
Masahiro Hashizume
F.R. Hashmi
Javeria Hashmi
S. Shahrukh Hashmi
Yasha Hasija
Marie Haskell
György Hasko
Sumaira Hasnain
Nurit Haspel
Joel Hass
Afif Hassairi
Christopher Hassall
Ahnaf Hassan
Amin Hassan
Cesare Hassan
Elham A. Hassan
Jaythoon Hassan
Mahmoud Hassan
Ranya Hassan
Samah Hassan
Syed Hassan
Tidi Hassan
Behrooz Hassani-Mahmooei
Gholamreza Hassanzadeh
Barbara Hasse
David Hassel
Kristian Hassel

Monika Hassel
Daniel Hasselman
Fred Hasselman
Amanda Hassinger
Shayne Hassler
Terry Hassold
Christopher Hasson
Dan Hasson
Michael Hast
Caroline Hastings
Karen Hasty
Hiroki Hata
Shingo Hata
Shoji Hata
Yutaka Hata
Hiroyasu Hatakeyama
Katsunori Hatakeyama
Masatsugu Hatakeyama
Kevin Hatala
Jaclyn Hatala Matthes
Jun Hatazawa
Leon Dupree Hatch
Nan Hatch
Janet Hatcher-Roberts
Hiroyasu Hatekayama
Ronald Hatfield
Timothy Hatfield
Camilla Hatleberg
Erez Hatna
Yuta Hatori
Mathieu Hatt
Shilpa Hattangadi
Kirsten Hattermann
Andrew Hattersley
Rajni Hatti-Kaul
Maximilian Hatting
Elke Hattingen
Toshio Hattori
Ioannis Hatzaras
Vassilla Hatzitaki
Julia Hatzold
Stavros Hatzopoulos
Marianne Hatzopoulou
Peter Hau
Sebastian Haubitz
Christina Haubrich
Christof Hauck
Katharina Hauck
Ruediger Hauck
Stefanie Hauck
Dominik Haudenschild
Martin Hauer-Jensen
Sabine Hauert
Thierry Hauet
Alexander Tobias Haug
Severin Haug
Kristina Haugaa
Håvard Haugen
Havard Jostein Haugen
Kjetil Haugen
Thomas Haugen
Matthew Haugh
Norman Haughey
Sean Haughian
Sylvie Hauguel-de Mouzon
Jon Hauksson
Catharina Hauman
Dominique Haumont
Hans Hauner
Robin Haunschild
Joachim Haupt
Thomas Haupt
Giselbert Hauptmann
Steffen Hauptmann
Claudine Hauri
Ben Hause
Bettina Hause
Derek Hausenloy
Andrea Hauser
David Hauser
Donna Hauser
Hansjörg Hauser
Kurt Hauser
Michael Hauser
Peter Hauser
Pierre Hausfater
Rainer Hausler
Darius Häusler
Dorothy Hausman
Axel Hausmann
Ludger Hausmann
Georg Hausner
Thomas Hausner
Helena Hauss
Kevin Haussler
Mark Haussler
Stefanie Haustein
Sampsa Hautaniemi
Richard Hautmann
Denis Hauw
Caroline Hauxwell
Viktoria Havas

Andrea Havasi
Mária Havašová
Arie Havelaar
Jennifer Havens
Karl Havens
Remco Havermans
Michael Havey
Jeannette Haviland-Jones
Philippe Ha-Vinh
Martin Havlicek
Jaroslav Havlik
Stepan Havranek
Daniel Hawcutt
Stefan Hawelka
Ian Hawes
Stephen Hawes
Ljac Hawinkels
Kristen Hawkes
Lucy Hawkes
Michael Hawkes
Barbara Hawkins
C. Matthew Hawkins
Claudia Hawkins
David Hawkins
Guy Hawkins
Hal Hawkins
Helen Hawley-Hague
Gregory Hawryluk
David Hawthorne
Claudie Haxaire
Dale Hay
Eric Hay
Jesse Hay
Phillipa Hay
Phillipa J. Hay
Simon Hay
Takashi Hayakawa
Yoshihiro Hayakawa
Junichiro Hayano
Ryugo Hayano
Naoto Hayasaka
Kanna Hayashi
Kazohiko Hayashi
Ken-ichiro Hayashi
Shigehiko Hayashi
Shigeo Hayashi
Taichi Hayashi
Terumasa Hayashi
Teruo Hayashi
Toshio Hayashi
Yasunori Hayashi
Yukiko Hayashi
Morihiro Hayashida
Tomoko Hayashida
Tassawar Hayat
Brian Hayden
Kathleen Hayden
Matthew Hayden
Melissa Hayden
Salim Hayek
Andrew Hayen
Heather Hayenga
Manajit Hayer-Hartl
Alan Hayes
Bronwyn Hayes
Heather Hayes
John Hayes
Louise Hayes
Mark Hayes
Matthew Hayes
Richard Hayes
Sara Hayes
Taylor Hayes
Andrea Hayes-Jordan
Boris Hayete
Laura Hayman
Gary Haynes
Graeme Hays
Kimberly Hays
Rebecca Hays
Andrew Hayward
Christopher S. Hayward
Gary Hayward
Matt Hayward
Steven Hayward
Vincent Hayward
Susan Haywood
Christina Hazard
Florette Hazard
Surovi Hazarika
Khaled Hazaymeh
Susan Hazel
Leslie Hazell
William Hazelton
Kevin Hazen
Wouter Hazenbos
Einat Hazkani-Covo
Lori Hazlehurst
Alain Hazotte
Tapas Hazra
Baoji He
Bei He

Biao He
Cheng He
Chenyang He
Chunyan He
Congcong He
Daqing He
Debiao He
Dekui He
Dianning He
Dongli He
Dongxiao He
Feng He
Gen He
Haiqi He
Hao He
Haohua He
Hong-Di He
Hongjian He
Hongping He
Hong-Wen He
Hua He
Jian He
Jiang He
Jiangping He
Jianguo He
Jianhua He
Jianzhong He
Jiayuan He
Jin He
Jing He
John He
Jun He
Junna He
Junxian He
Jun-Xian He
Kai He
Kongwang He
Li He
Liang-Nian He
Lin He
Meian He
Miao He
Mingrong He
Peng He
Ping He
Pingan He
Rong-Rong He
Shaomei He
Shuilin He
Shun-Min He
Songqing He
Tao He
Tianyu He
Tong-Chuan He
Wei He
Weilue He
Wei-Ming He
Wenqing He
Xiang He
Xiao He
Xiaoming He
Xiaoxiao He
Xiaoyan He
Xin He
Xuesong He
Xuezhong He
Yayi He
Ye He
Yiping He
Yong He
Yonghan He
Yu He
Yu-Long He
Zangdong He
Zengyou He
Zhe He
Zhengbing He
Zhigang He
Zhiheng He
Zhuohao He
Michael Head
Stewart Head
Simon Header
Selwyn Headley
Christopher Healey
John Healey
Brian Healy
Genevieve Healy
Kristen Healy
Luke Healy
Geoff Heard
Stephen Heard
Patrick Hearing
Alex Hearn
David Hearn
Lynn Heasley
Lenwood Heath
Matthew Heath
Jason Heaton
Nicholas Heaton
Robert Heaton
Tim Heaton

Lionel Hebbard
Jeremy Hebden
Marianne Heberlein
Mason Heberling
James Hebert
Lee Hebert
Luciana Hebert
Renske Hebing
Maxime Hebrard
Beatrice Hechler
Tanja Hechler
Stephen Hecht
Lily Hechtman
Albert Heck
Julia Heck
Scott Heckathorn
Frank Heckel
Gerald Heckel
Reinhard Heckel
Tobias Hecker
Andrew Heckert
James Heckman
Michael Heckman
Stefan Heckmann
Stefanie Hectors
Craig Hedberg
Jakob Hedegaard
Donald Hedeker
Peter Hedera
Matthias Hediger
Darren Hedley
John Hedley
Richard Hedley
Khedidja Hedna
Laetitia Hédouin
Christian Hedrich
Margaret Hedstrom
Tobias Heed
Niels Heegaard
Hannelore Heemers
Jennifer Heemstra
Adel Heenan
Alexandre Heeren
Joerg Heeren
Ron Heeren
Ralf Heermann
C. Heesen
Abraham Hefetz
James Heffernan
Henry Heffner
Ahmed Hefny
Dominic Hegarty
Kelsey Hegarty
Matthew Hegarty
Yamen Hegazy
Madhuri Hegde
Inga Hege
Dwayne Hegedus
Zoltan Hegedus
Richard Hegele
Robert Hegele
Harald Hegen
Michal Heger
Tina Heger
Janice Hegewald
Lars Heggelund
Daniel Hegglin
K.W. Hegland
Kurt Hegmann
Rainer Hegselmann
Jan-Hendrik Hehemann
Wouter Hehenkamp
Stephanie Hehlgans
Tom Hei
Raphael Heiberger
Soroush Heidari Pahlavian
Fatemeh Heidary
Isabel Heidegger
Joel J Heidelbaugh
Britt Heidinger
Benjamin Heidrich
Timo Heidt
Irene Heijink
Jordi Heijman
M. Heijnen
Reinout Heijungs
Oskari Heikinheimo
Lawrence Heilbronn
Lance Heilbrun
C. Heilig
Charles Heilig
Christine Heilmann
Albert Heim
Masahiro Heima
Mark Heiman
Mikael Heimann
Harry Heimberg
Robert Heimer
Gaston Heimeriks
George Heimpel
Grit Hein
Nadine Hein

Travis Hein
Jyrki Heinämäki
Sirpa Heinävaara
Jerrold Heindel
Gunnar Heine
Holger Heine
Martin Heine
Steven Heine
Jörg Heineke
Katie Heineman
Udo Heinemann
Uwe Heinemann
Monika Heiner
Erin Heiniger
Andreas Heinimann
Dietmar Heinke
Indrek Heinla
Margit Heinlaan
Mikko Heino
Ari Heinonen
Ilkka Heinonen
Krista Heinonen
Seppo Heinonen
Jussi Heinonsalo
Jud Heinrichs
Els Heinsalu
Ellen Heinsbroek
Ruth A Heinz
Cailin Heinze
Frank Heinzel
Sebastian Heinzel
Katharina Heinzelmann
Robert Heinzen
Silke Heinzmann
Thomas Heise
Alex Heiser
Clemens Heiser
Peter Heisig
Margit Heiske
Florian Heiss
Georg Heiss
Wolf-Dieter Heiss
Jennifer Heissel
Vigo Heissmeyer
Donald Heistad
Joseph Heitman
Ryan Heitmann
Thierry Heitz
Mary Heitzeg
Gilles Hejblum
J. Fielding Hejtmancik
Evon Hekkala
P. Hekking
Eric Hekler
A. Helander
Heikke Helantera
Ilkka Helanterä
Karla Helbig
Kathryn Held
Paraskevi (Evi) Heldin
Scott Heldt
Carrie Heleski
Fabrice Helfenstein
Valeska Helfinger
Christian Helfrich
Christine Helfrich
Charlotte Helfrich-Forster
Ásgeir Helgason
Jorn Helge
Luisa Helguero
Sebastien Helie
Jean-Marie Helies
Kari Heliövaara
Markus Hell
Rosalee Hellberg
Erika Hellenbart
Daniel Heller
Loree Heller
Markus Heller
Meera Heller
Rebecca Heller
Stefan Heller
Per Hellman
Jens Hellmann
Rick Hellmich
Wayne Hellstrom
Kerstin Hellwig
Nathan Hellyer
Lothar Helm
Mark Helm
Rebecca Helm
Robert Helm
John Helmann
Francoise Helmbacher
Helena Helmby
Stefanie Helmer
Anjel Helms
Hans Christian Helms
My N. Helms
Volkhard Helms
Moritz Helmstaedter
Brian Helmuth

Liat Helpman
Anne-Sofie Helvik
Mohammad Helwani
Ashok Hemal
David Hembry
Hanan Hemead
Bahram Hemmateenejad
Farhid Hemmatzadeh
Bernhard Hemmer
Andrea Hemmerlin
Bradley Hemminger
Casper Hempel
Natalie Hempel de Ibarra
Moritz Hempen
Marcela Henao-Tamayo
Kimiora Henare
Richard Henchman
Andrew Henderson
Claire Henderson
Davin Henderson
Elizabeth Henderson
Janet Henderson
Joanna Henderson
Ken Henderson
Lorna Henderson
Theodore Henderson
Paul Hendrick
Eric Hendrickson
Jeanne Hendrickson
Antoni Hendrickx
Friederike Hendriks
Steven Hendriks
Wiljan Hendriks
Peter Hendriksen
Harmen Hendriksma
Roger Hendrix
Paul Hendry
Brian Henen
Henry Heng
Hong-Qiang Heng
Wee Ling Heng
Zhang Heng
Tomislav Hengl
Ulrich Hengst
Peter Henke
Graeme Henkelman
R. Henkelman
Maciej Henneberg
Paul Henneberger
Sylvianne Hennebicq
Julie Hennegan
Christophe Hennequin
Claire Hennequin
Ludovic Henneron
John Hennessey
Annemarie Hennessy
Dwight Hennessy
Ellis Hennessy
Michael Hennessy
Sean Hennessy
Frauke Hennig
Jürgen Hennig
Matthias Hennig
Lars Henning
M. Henning
Helen Henningham
Jamie Henningson
Gwenn Hennon
Mark Hennon
Roger E Hennriken
Deborah Hennrikus
A. K. Henras
Michael Henrich
Natalie Henrich
Bethany Michel Henrick
Lena Henriksen
Pontus Henriksson
Cristina Henriques
Jorge Henriques
Jose Henriques
Maria das Graças Henriques
Sónia T. Henriques
Flavio Henrique-Silva
Craig Henriquez
Fiona Henriquez
Yves Henrotin
Brandon Henry
Charles Henry
David Henry
Donald Henry
Jaymie Henry
Kevin Henry
Laurence Henry
Lea-Anne Henry
Mary Henry
Michael Henry
Molly Henry
Robert Henry
Stephen Henry
Philipp Henschel
Devon Hensel
Michael Hensel

Bernadette Hensen
Jonathan Henshaw
Gunnel Hensing
Yvonne Henskens
Ken Hensley
Scott Hensley
Joseph Henson
Peter Henson
Thomas Henthron
Benjamin Henwood
Hye-Young Heo
Ji Hoe Heo
Jung Sun Heo
Jungmin Heo
Yoonseok Heo
Lee Heow Pueh
Robert Hepach
Leanne Hepburn
Jussi Hepojoki
Scott Heppell
Shelley Hepworth
Chengtao Her
Benji Heran
Chandana Herath
Frederic Herault
Samuel Herberg
Holly B. Herberman Mash
Marie Herberstein
Cristan Herbert
Debroski Herbert
Gregory Herbert
Rob Herbert
Terence Herbert
Andreas Herbst
Christian Herbst
Sophie Herbst
Werner Herbst
Melissa Herbst-Kralovetz
Pierre Herckes
Maria Teresa Herdeiro
Christiane Herden
Hubertine Heremans
Gustavo Heresi
Ivar Herfindal
Lydie Herfort
Paul Herijgers
A. Heritage
Johannes Herkel
Eva Herker
Kristjan Herkül
Christopher Herlihy
Erin Herlihy
Anders Herlin
Stefan Herlitze
Holger Herlyn
Meenhard Herlyn
Ira Herman
Joseph Herman
Lule Herman
Michael Herman
Peter Herman
Anna Herman-Antosiewicz
J. Hermanides
Brian Hermann
Dirk Hermann
Norbert Hermanns
Slawomir Hermanowicz
Amy Herman-Roloff
Emmanuel Hermans
Sabine Hermans
Robert Herman-Smith
Ola Hermanson
Michael Hermanussen
Souhail Hermassi
Jean-Michel Hermel
Frouke Hermens
Gerben Hermes
Dominique Hermier
Louis Hermo
John Hermos
Carlos Hermosilla
Jochen Herms
Joachin Hermsdorfer
Jerome Hermsen
Juan Hernadez-Tamames
Istvan Hernadi
Jean-Pierre Hernalsteens
Roberto Hernán Gonzalez
Ana Hernandez
Arturo E. Hernandez
Brenda Hernandez
Christopher Hernandez
Diana Hernandez
Elena Hernandez
Felix Hernandez
Jesus Hernandez
Luis Hernandez
M. M. Hernandez
Maria Hernandez
Mauro Hernandez
Rebecca Hernandez
Teri Hernandez

Victor Hernandez
Cristina Hernández
Laura Hernández
Roberto Hernandez-Alejandro
Luis Hernandez-Garcia
Jorge Hernandez-García
Ismael Hernandez-Gonzalez
Manuel Hernández-Guerra
Ana Hernández-Hernández
Rey Hernandez-Julian
Salvador Hernández-Martínez
Gabriela Hernandez-Molina
Jesús M Hernández-Rivas
J. Luis Hernandez-Stefanoni
Francisco Hernández-Torres
Esteban Hernandez-Vargas
Fernando Luis Hernando
Victoria Hernando
Dennis Hernaus
Philippe Hernigou
Dorrit Herold
Christel Herold-Mende
Sarah Heron
Martin Héroux
Paul Héroux
Sabine Herpertz
Daniel Herr
Deron Herr
John Herr
Joshua Herr
Nadine Herr
Etienne Herrbach
Anthony Herrel
Anthony Herren
Andrea Herrera
Antonio Herrera
Carolina Herrera
Ileana Herrera
Luisa Herrera
Maria Herrera
Gerardo Herrera Corral
Enrique Herrera Viedma
José Herrero
Markus Herrgard
Jason Herrick
Jennifer Herricks
Andrew Herries
John Herring
Paul Herring
Stehanie Herring
Sue Herring
Burkhard Herrmann
Carl Herrmann
Christian Herrmann
Dominik Herrmann
Joerg Herrmann
Macus Herrmann
Martin Herrmann
Michael Herrmann
Thomas Herrmann
W. Herrmann
Matthew Herron
Christophe Herry
Jason Herschkowitz
Harvey Herschman
Florian Herse
Israel Hershkovitz
Carlton Hershner
Ronald Hershow
Mona Hersi
Mette Herskin
Hans-Petter Hersleth
Jana Hertel
Thomas Hertel
Claudiu Herteliu
Jan Herter
Troy Herter
Jerald Herting
Kate Hertweck
Edgar Hertwich
Andreas Hertz
Daniel Hertz
Jens Michael Hertz
Tomer Hertz
Alexis Hervais-Adelman
Antonio Hervás
Jactel Hervé
Rodolphe Hervé
Loreen Herwaldt
Christian Herweh
Mark Herzberg
Hanspeter Herzel
Eytan Herzig
Denise Herzing
Daniel Herzka
Sharon Herzka
Erica Herzog
Rebecca Herzog
Roland Herzog
Stefan Herzog
Ulrike Herzschuh
Kathryn Hesketh

David Heslop
Luke Heslop
João Hespanha
Anna Hess
Jochen Hess
Kenneth Hess
Leonardo Hess
Maureen Hess
Michael Hess
Rebecka Hess
Robert Hess
Constanze Hesse
Guido Hesselmann
Amanda Hessels
Amelia Hessheimer
Elisabeth Hessmann
Robert Hester
Matthew Hethcoat
Kristina Hettne
Manuel Hetzel
Rainer Heuchel
Benjamin Heumann
Rolf Heumann
Klaus Heuner
Martin Heur
Marco Heurich
Marc Heuschkel
Michael Heuser
Nicole Heussen
Marjolein Heuvelmans
Mark Hew
Geoffrey Hewings
A. J. Mark Hewison
Jenny Hewison
Alex Hewitt
Joanne Hewitt
Judi Hewitt
Miles Hewstone
Nick Hex
Dennis Hey
Mahyar Heydarpour
Robert Heyderman
Stanley Heydrick
Karolina Heyduk
Tomasz Heyduk
Tobias Heye
Arnd Heyer
Geoffrey Heyer
Dominik Heyers
Daren Heyland
Gunter Heylens
Beate Heym
Anthony Heymann
Eckhard Heymann
James Heys
Jeffrey Heys
Evelien Heyselaar
Wendy Heywood
Bernd Hezel
Dusan Hhadzi-Ppavlovic
Yoichi Hiasa
Robert Hiatt
Kenichiro Hibara
Bruce Hibbard
Judith Hibbard
Ryan Hibbs
Terumasa Hibi
Hiroshi Hibino
Michael Hicke
Aodhan Hickey
Daniel T. Hickey
Lee Hickey
Deb Hickman
Brian Hicks
David Hicks
Joan Hicks
Lanis Hicks
Paul Hicks
Stephanie Hicks
Andres Hidalgo
Bertha Hidalgo
Jose Hidalgo
Matias Hidalgo-Sánchez
Diana Hide
Geoff Hide
Pieter Hiemstra
Nguyen Duc Hiep
Martin Hieronymi
Brian Hiestand
Ville Hietakangas
Jari Hietanen
Elisa Higa
Jason Kenji Higa
Akinori Higaki
Katsumi Higaki
Takumi Higaki
Michiyo Higashi
Yasuhiro Higashi
Yukihito Higashi
Haruhiro Higashida
Matt Higger
Denice Higgins

Des Higgins
John Higgins
Julian Higgins
Steven Higgins
Christopher Higginson
Irene Higginson
Elizabeth Higgs
Henry Higgs
Paul Higgs
Sarah Highlander
Akihiro Higuchi
Anita Hiippala
Diego Hijano
Taiichi Hikichi
Hayato Hikita
Leena Hilakivi-Clarke
Amaya Hilario
Constantinos Hilas
Thomas Hilberg
David Hilbert
Martin Hilbert
Yamandu Hilbert
Ray Hilborn
Frank Hildebrand
Michael Hildebrand
Andrea Hildebrandt
Gerhard Hildebrandt
Helena Hildenwall
Robert Hilderbrand
Alan Hildrew
Eberhard Hildt
Roger Hilfiker
Joseph Hilgard
Rolf Hilgenfeld
Ralf-Dieter Hilgers
Leandro Hilgert
Norma Hilgert
Medard Hilhorst
Frederic Hilke Meier
Ines Hilker
Alison Hill
Andrew Hill
Annie Hill
Antony A Hill
Bradford Hill
Darryl Hill
David Hill
Deborah Hill
Eliza Hill
Erin Hill
Janet Hill
Kathleen Hill
Lauren Hill
Linda Hill
Melissa Hill
Michael Hill
Nicholas S. Hill
Richard Hill
Robert Hill
Ryan Hill
Frank Hillary
Georgios Hillas
R.J. Hillen
Patrick Hillenbrand
Maureen Hillenmeyer
Natalia Hiller
John Hillier
Susan Hillier
Frances Hillier-Brown
Sophie Hillion
Argye Hillis
Noah Hillman
Falk Hillmann
Ansel Hillmer
Danny Hills
Kimberly Hills
Nancy Hills
Ronald Hills
Thomas Hills
Nathan Hillson
Roger Hillson
Jane Hillston
Julian Hillyer
Robert Hilsden
Markus Hilty
Hauke Hilz
Misako Himeno
Seiichiro Himeno
Heinz Himmelbauer
David Himmelgreen
Brett Himmler
Tobias Himmler
Xavier Hinaut
Dirk Hincha
Monique Hinchcliff
Erica Hinckson
Michael Hinczewski
Katherine Hinde
Sajedah Hindi
Charles Hindmarch
Gerhard Hindricks
Terry Hinds Jr.

George Hindy
Christopher Hine
Donald Hine
Andrew Hines
Ian Hines
Jes Hines
Lisa Hines
O. Hines
Paul Hines
Sunil Hingorani
Darko Hinic
Simon Hinke
Rabea Hinkel
Jorma Hinkula
Anke Hinney
Keisuke Hino
Norihide Hinomoto
Saba Hinrichs-Krapels
Stephen Hinshaw
Damien Hinsinger
Shelley Hinsley
David Hinton
Jay Hinton
Tina Hinton
Arend Hintze
Niels Hintzen
Rainer Hinz
Lori Hinze
Catarina Hioe
Daniel Hippe
Go Hirabayashi
Toru Hiraga
Hideyo Hirai
Hiroki Hirai
I. Hirai
Midori Hirai
Satoshi Hirakawa
Koji Hiraki
Kazuhiko Hiramatsu
Katsuya Hirano
Masayuki Hirano
Shigeru Hirano
Tsutomu Hirano
Pornpan Hiranpradit
Atsushi Hirao
Koichi Hiraoka
Kotaro Hiraoka
Akira Hirasawa
Tsuyoshi Hirashima
Isao Hirata
Rogerio Hirata
Yoshihito Hirata
Masahiro Hiratsuka
Nobuhito Hirawa
Yoshitaka Hirayama
Bertrand Hirel
Swapnil Hiremath
Marcia Hiriart
E. Hiris
Marco Hirnstein
Makoto Hiroi
Ikuo Hirono
Moritoshi Hirono
Kazuyuki Hirooka
Masashi Hirooka
Yoshiki Hirooka
Masamichi Hirose
Tanaka Hiroshi
Yasushi Hirota
Camila Hirotsu
Sadao Hiroya
Elizabeth Hirsch
Ivan Hirsch
Lawrence Hirsch
Matthew Hirsch
Oliver Hirsch
Philipp Hirsch
Gilad Hirschberger
Matthew Hirschey
Lisa Hirschhorn
V. Hirschler
Jon Hirshon
Petra Hirsova
Andrew Hirst
Martin Hirst
Ed Hirt
Edward Hirt
Hal Hirte
Kalle Hirvonen
Noora Hirvonen
Yohei Hisada
Hajime Hisaeda
Shin-ichi Hisanaga
Yu Hisano
Peter Hiscock
Jan Hiss
Francisco Hita Garcia
Peter Hitchcock
Carol Hitchon
Mary Hitt
Shailaja Hittalmani
Claire Hivroz

Kyoko Hiwasa-Tanase
Y. Hiyama
Clara Hjalmarsson
Jesper Hjortdal
Peter Hjortrup
Michelle Hladik
Myron Hlynka
Brian Ho
Chen-Hsun Ho
Chen-Lung Ho
Chi-Tang Ho
Derek Ho
Dora Ho
Emmanuel Ho
Hao Ho
Jemima Ho
Jennifer Ho
Jessica Ho
Joshua Ho
Kwok Ho
Mengfei Ho
Peggy Ho
Sai-Yin Ho
Sheng Yow Ho
Shinn-Ying Ho
Steven Ho
Yen-Yi Ho
Yuh-Shan Ho
Zih-Ping Ho
David Hoaglin
Michael Hoane
Quyen Hoang
Trang Hoang
Richard Hoare
Zoe Hoare
Thierry Hoareau
Sean Hoban
Brian Hobbs
Guy Hobbs
Joanne Hobbs
Robin Hobbs
Roderick Hobbs
Ryan Hobbs
Tom Hobbs
Alistair Hobday
Calvin Hobel
Markus Hobert
Eleanor Hobley
Tom Hobman
Charles Hobson
David Hobson
Elizabeth Hobson
Keith A Hobson
Peter Hobson
Jody Hobson-Peters
Roman Hobza
Tomaž Hocevar
John Hoch
Peter Hoch
Dorit Hochbaum
Michael Hochberg
Berthold Hocher
Valérie Hocher
Daniela Hochfellner
Ute Hochgeschwender
Alejandro Hochkoeppler
Hartwig Hochmair
Guy Hochman
Sonja Hochmeister
Dieter Hochuli
Barbara Hock
Andreas Hocke
Joel Hockensmith
Gregory Hockerman
Darren Hocking
Denise Hocking
Matthew Hocking
John Hoddinott
Eva Maria Hodel
Richard Hodel
Allison Hodge
David Hodge
Alastair Hodges
Karen Hodges
Nikolas Hodges
Sara Hodges
Sheilagh Hodgins
Conrad Hodgkinson
James Hodgkinson
Tammy Hodgskiss
Amr Hodhod
Israel Hodish
Bridget Hodkinson
Duncan Hodkinson
Didier Hodzic
Christian Hoecht
C.W. Hoedemaekers
M. Hoedjes
Julia Hoefer
Andreas Hoeflich
Kristin Hoeft
Jens Høeg

Sandra Hoegl
Guenter Hoeglinger
Kyle Hoehn
Thomas Hoehn
Gerard Hoek
Bert Hoeksema
Hopi Hoekstra
Michael Hoelscher
Rus Hoelzel
Caroline Hoemann
Miriam Hoene
Thomas Hoenen
Hans Hoenicka
Martin Hoenigl
Astrid Hoeppner
Stephane Hoerle
André Hoerning
Nicolas Hoertel
Stefan Hoerzer
Peter Hoet
Alexander Hoetzel
Konrad Hoetzenecker
Andrew Hoey
Anouschka Hof
Gunther Hofbauer
Markus Hofer
Stefan Hofer
Stefan Höfer
Erika Hoff
John Hoffecker
Barry Hoffer
Michael Hoffer
Brenton Hoffman
Caroline Hoffman
Carsten Hoffman
Daniel Hoffman
Dennis Hoffman
Geoffrey Hoffman
George Hoffman
James Hoffman
Jill Hoffman
Karen Hoffman
Keith Hoffman
Marian Hoffman
Matthew Hoffman
Melissa Hoffman
Paul Hoffman
Rhonda Hoffman
Richard Hoffman
Robert Hoffman
Stanley Hoffman
Andreas Hoffmann
Ary Hoffmann
Benjamin Hoffmann
Boris Hoffmann
Charles Hoffmann
Christopher Hoffmann
Donata Hoffmann
Jedrzej Hoffmann
Klaus Hoffmann
Markus Hoffmann
Peter Hoffmann
Petra Hoffmann
Rasmus Hoffmann
Robert Hoffmann
Uwe Hoffmann
Daniel Hofius
Johannes Hofland
Manfred Höfle
Winni F. Hofman
Douglas Hofmann
Ilse Hofmann
Johann Hofmann
Laurie Hofmann
Mathias Hofmann
Richard Hofmann
Tobias Hofmann
Volker Hofmann
Marion Hofmann Bowman
Martin Hofmann-Apitius
Craig Hofmeister
Erik Hofmeister
Stefanie Hofstede
Richard W. Hofstetter
Simon Hofstetter
Romana Höftberger
Benedict Hogan
Deborah Hogan
Ellizabeth Hogan
Harry Hogan
Robert Hogan
William Hogan
Mark Hogarth
Michael Hogarty
Eef Hogervorst
Janneke Hogervorst
Hans Hogerzeil
John Hogg
Neil Hogg
Peter Hogg
Robert Hogg
Ruth Hogg

Donna Hogge
Petra Högger
Wolfgang Högler
Günter Höglinger
Jacob Hoglund
Johan Hoglund
Petter Höglund
Jean-Yves Hogrel
Brenda Hogue
Charles Hogue
Josephine Hoh
Johann Hohenegger
Daniel Hohl
Tobias Hohl
Pierre Hohmann
Martin Hohmann-Marriott
Herbert Hoi
Anneli Hoikkala
Arne Didrik Høiseth
Lone Høj
Aki Hoji
Radovan Hojs
Diego Hojsgaard
Akira Hokama
Seiji Hokimoto
Cornelis Hokke
Akishige Hokugo
Scott Holaday
Matthew Holahan
Joanna Holbrook
Luke Holbrook
Michael Holbrook
John Holcomb
Arun Holden
John Holden
Anthony Holder
Marc Holderied
David Holding
Lesca Holdt
Graham Hole
Elisabeth Holen
Mandë Holford
Fernando Holguin
Nilsson Holguinn
Michael Holick
Toril Holien
Karen Holl
Marc Höll
Carol Holland
Doron Holland
Jason Holland
Maggie Holland
Nicholas Holland
Christine Hollander
Johan Hollander
John Hollander
Gareth Hollands
Justin G Hollands
Kristen Hollands
Trina Hollatz
Rolf Holle
Bregje Holleman
Anthony Hollenberg
Ernst Holler
Richard Holley
Jay Hollick
Casey Holliday
Jason Holliday
Clayton Hollier
Richard Hollingsworth
Andrew Hollingworth
Mark Hollins
Emily Hollister
Maria-Graciela Hollm-Delgado
Gábor Holló
Alison Holloway
Graham Holloway
Kathleen Holloway
Jennifer Hollowell
Thomas Holly
Helle Holm
Lars-Erik Holm
Sverre Holm
Eric Holman
Dan Holmberg
Thomas Holme
Gregory Holmes
Jacinta Holmes
John Holmes
N. Holmes
Philip Holmes
Thomas Holmes
Tom Holmes
Arne Holmgren
Noél Holmgren
Trygve Holmøy
Wolfgang Holnthoner
Mark Holodniy
Joseph Holoshitz
Eleanor Holroyd
Jayna Holroyd-Leduc
Hannah Holscher

J.J. Holst
Lone Holst
Daniel Holstein
Joerg Holstein
Andrew Holt
Ben Holt
Bethany Holt
Kathleen Holt
Martin Holt
Natalie Holt
Stephen Holt
Harald Holte
Derald Holtkamp
Julianne Holt-Lunstad
Daniel Holtmann-Rice
August Holtyn
Tim Holtz
Nick Holtzman
Kirsten Holven
Kristin Holvik
Bryan Holvoet
Paul Holvoet
David A. Holway
Margaret Holyday
Todd Holyoak
Olaf Holz
Claus Holzapfel
Christina Hölzel
Martin Holzenberger
Astrid Holzer
Max Holzer
Andreas Holzinger
Dirk Holzinger
Emily Holzinger
A. Holzkämper
George Hölzl
Iris Holzleitner
Gregg Homanics
Arne Homann
Jihane Homann-Ludiye
Michael Hombach
R. Homburg
Caroline Homer
Carol Homko
Bernhard Hommel
Eduardo Homsi
Jaco Homsy
Behnam Honarvar
Ayako Honda
Kazufumi Honda
Kazuhisa Honda
Shigeru Honda
Sumihisa Honda
Tetsumi Honda
Yasushi Honda
Yoshitomo Honda
Robert Hondal
David Hone
Jim Hone
Jonathan Honegger
Johannes Hönekopp
Rob Honey
Bonghee Hong
Changjin Hong
Chen Hong
Christine Hong
Chun-Ming Hong
Dengfeng Hong
Hyun Ju Hong
Jiarong Hong
Jiaxu Hong
Jin Tae Hong
Jiong Hong
Jong Wook Hong
Kar Wai Hong
Kyeong-Man Hong
Kyung Sue Hong
Liu Hong
Michelle Hong
Minsun Hong
Sang-Bum Hong
Seok Jong Hong
Seongjin Hong
Seung Bong Hong
Seung-Heon Hong
Seungpyo Hong
Sheng Hong
Sok Chul Hong
Song Hee Hong
Sung-Ha Hong
Wei Hong
Yang Hong
Yanguo Hong
Yi Hong
Yijiang Hong
Yoon-Ho Hong
Young-Kwon Hong
Yunhan Hong
Zhongkui Hong
Zhou Hong
Dai Hong-Jie
Simone Honikman

Kaori Honjo
Ken-ichi Honjoh
Juuso Honkanen
Patrick Honore
Jean Honorio
Marcel Honza
Ruby Hoo
Anna Hood
Shveta Hooda
Hans Hoogduin
Ignace Hooge
Mia Hoogenboom
Ron Hoogenboom
Mark Hoogendoorn
Martine Hoogendoorn
Rudolf Hoogenveen
Wouter Hoogkamer
Edward Hook
Magnus Höök
Bryan Hooks
Amanda Hooper
Gary Hooper
Michael H. Hooper
Scott Hooper
Stuart Hooper
Tony Hooper
Daniel Hoops
Ewout Hoorn
David Hoos
Mevin Hooten
Donald Hoover
Edward Hoover
Joe Hoover
Kelli Hoover
Timothy Hoover
Paul Hooykaas
Aluko Hope
Andrew G. Hope
Michael Hope
Harriet Hopf
Jens-Max Hopf
Lan Ho-Pham
Robert Hopkin
Ashley Hopkins
Debbie Hopkins
Gareth Hopkins
Jason Hopkins
Katie Hopkins
Will Hopkins
Brian Hopkinson
Sebastian Hoppe
Julie Hopper
Keith Hopper
Lydia Hopper
Felix Hoppe-Seyler
Jessica Hoppstädter
Matthew Hoptman
Mohammad Hoque
Ricardo Hora
Faye Horak
Rita Hõrak
John Horan
William Horan
Amjad Horani
Michael Horberg
Craig Horbinski
Faouzi Horchani
P. Hore
Tal Hörer
Danny Horesh
Kazuhiro Hori
Koji Hori
Masatoshi Hori
Sharon Hori
Rie Horie
Gorou Horiguchi
Toshihiro Horiguchi
Yasuhiko Horiguchi
Arata Horii
Takuro Horii
Nobuo Horikoshi
Takahiro Horinouchi
Fumihiko Horio
Masaru Horio
Tetsuya Horita
Akira Horiuchi
Keisuke Horiuchi
Pavel Horky
Sandrine Horman
David Horn
Martin Horn
George Hornberger
M. Hornberger
David Horne
James Horne
Jon Horne
Simone Hornemann
Christopher Horner
Mark Horner
Willi Horner-Johnson
Jeremy Hornibrook
Matthew Hornsey

Daniela Hornung
Thomas Hornyak
Ioana Horodnic
Alice Horowitz
John Horowitz
Nicholas Horrocks
Robert Horsburgh
Alex Horsley
William Horsnell
K. Horst
Maya Horst
Walter Horst
Olaf Horstick
Roarke Horstmeyer
Alexander Horswill
Craig Horswill
Mariana Hort
Lisa Horth
Marcus Hortmann
Radley Horton
Simon Horton
Sue Horton
Susan Horton
William Horton
Anatolij Horuzsko
Andela Horvat
David Horvath
Gyongyi Horvath
Klara Horvath
Marc Horwitz
Marshall Horwitz
Mitchell Horwitz
Anna Horwood
Geoffrey R Hosack
Ramegowda HosahallyVenkate
Moshe Hoshen
Naoto Hoshi
Takashi Hoshiba
Satoshi Hoshide
Katsuyuki Hoshina
I. Hoshino
Junichi Hoshino
Tatsuhiko Hoshino
Yoichiro Hoshino
Kathryn Hosig
Andrew Hoskins
Jason Hoskins
Paul Hoskisson
Jon Hosler
Clemens Hosman
Karim Hosni
Masako Hosoi
Koji Hosokawa
Yoshitaka Hosokawa
Takashi Hosono
Ken-ichi Hosoya
Takahiro Hosoya
Duane Hospenthal
Blake Hossack
John Hossack
Faisal Hossain
Jahangir Hossain
Md. Iqbal Hossain
Mohammad Hossain
Shamim Hossain
Laure Hossard
Marzieh Hosseini
Saman Hosseinkhani
Ali Hosseinsabet
Leila Hosseinzadeh
Abasalt Hosseinzadeh Colagar
Amir Hossini
Bruce Hostager
Jesse Hostetter
Nathan Hostetter
Shannon Hostetter
Thomas Hostetter
Camelia Hostinar
Eric Hosy
Swetansu Hota
Richard Hotchkiss
Stefan Hotes
Markus Hoth
Torsten Hothorn
H. Hotokezaka
Harumi Hotta
Michael Hottiger
Eugenio Hottz
Maria Hötzel
Anfu Hou
Chen Hou
Cheng-Lin Hou
Chenping Hou
Cong-Cong Hou
Fan Hou
Fujun Hou
Hailong Hou
Jing Hou
Jinghui Hou
Joshua Hou
Jun Hou
Juzhi Hou

Lin Hou
Lingmi Hou
Maomao Hou
Qi Hou
Quanwen Hou
Shaoping Hou
Sheng Hou
Shengping Hou
Sukuan Hou
Susan Hou
Tingjun Hou
Wei Hou
Xianqing Hou
Yan Hou
Yiping Hou
Zhanjun Hou
Zheng-Kun Hou
Zhuocheng Hou
Alfons Houben
Edith Houben
Michael Houbraken
John Houde
Gunnar Houen
Josselin Houenou
Jonathan Houghton
Lauren Houghton
Michael Houghton
James Hougland
Pascal Houillier
Peter Houk
Henry Houlden
Brian Houle
Timothy Houle
Tobias Houlton
Eric Houpt
Joseph Houpt
Katherine Houpt
Nicolette Houreld
Yael Houri-Haddad
Justin Jahon Hourmozdi
Martine Hours
Michael House
Stacey House
Gregory Houseman
Linda Houser
James Houston
Kelly Houston
Stan Houston
Carl Houtman
Renate Houts
Peter Houweling
Jan Hovanec
Outi Hovatta
David Hovda
Kevin Hovel
Philipp Hövel
R.C. Hovey
Tapani Hovi
Steve Hovick
Jeffery Hovis
Joppe Hovius
Amber Howard
Brian Howard
Christine Howard
Eric Howard
Guy Howard
James Howard
Louise Howard
Rebecca Howard
Thomas Howard
Clive Howard-Williams
Dianella Howarth
Gordon Howarth
Leigh Howarth
Mark Howarth
Sam Howarth
Kembra Howdeshell
Alan Howe
Franklyn Howe
Lewis Howe
Philip Howe
Alexandra Howell
Andrew J. Howell
David Howell
Jennifer Howell
Kate Howell
Matthew Howell
Michael Howell
Paul Howell
David Howells
Emily Howells
Timothy Howells
James Hower
Melanie-Jayne Howes
Rosalind Howes
Erin Howie
R. Nicole Howie
Susan Howitt
Mohammad Sajid Ali Howlader
Laurens Howle
Tim Howle
Philip Howles

Susan Howlett
Brendan Howlin
Elion Hoxha
Robert Hoy
Bimba Hoyer
Wolfgang Hoyer
Larry Hoyle
Cathrine Hoyo
Jaroslav Hrabak
Richard Hrabal
Terry Hrubec
Adela Hruby
Daniel Hruschka
Katarzyna Hrynkiewicz
Edward Hsiao
George Hsiao
Haoyuan Hsiao
Ing-Tsung Hsiao
Jong-Kai Hsiao
Kai-Long Hsiao
Liang-Tsai Hsiao
Li-Li Hsiao
Pei-Wen Hsiao
W.L. Wendy Hsiao
William Hsiao
Yaling Hsiao
Bety Hsie
Chia-Hsun Hsieh
Chia-Ling Hsieh
Ching-Liang Hsieh
Chung-Cheng Hsieh
Fang-I Hsieh
Hernyi Hsieh
Hsu-Liang Hsieh
Hui-Min Hsieh
Jen-Chuen Hsieh
Li-Chun Hsieh
Meng-Hsuan Hsieh
Ming-Hsiun Hsieh
Pang-Hsin Hsieh
Po-Jang Hsieh
Shie-Liang Hsieh
Shu-Ling Hsieh
Teng-Fu Hsieh
Yi-Fang Hsieh
Yun-Cheng Hsieh
David Hsiehchen
Yue-Ie Hsing
Chen-Ming Hsu
Chia-Hsien Hsu
Chia-Lin Hsu
Chih-Cheng Hsu
Chih-Hung Hsu
Ching-Fang Hsu
Ching-Sheng Hsu
Chin-Ying Hsu
Christine Hsu
Chun Liang Hsu
Chung-Yao Hsu
Chun-Nan Hsu
David Hsu
Hui-Chuan Hsu
Hwei-Jan Hsu
Jeffrey Hsu
Jue-Liang Hsu
Jui-Ting Hsu
Justine Hsu
Kai-Cheng Hsu
Kai-Chih Hsu
Kean Hsu
Lewis Hsu
Li-Chi Hsu
Pang-Hung Hsu
Pin-I Hsu
Po-Chao Hsu
Po-Lin Hsu
Shao-Jung Hsu
Shu-Hao Hsu
T. Hsu
Ted Hsu
Tsai-Wen Hsu
Wan-Tseng Hsu
Yao-Chun Hsu
Bih-Jen Hsue
I-Ping Hsueh
Pei-Yun Hsueh
Yi-Jen Hsueh
Tai-Chiu Hsung
Aixue Hu
Anyi Hu
Bang-Chuan Hu
Bin Hu
Bo Hu
Brian Hu
Caihong Hu
Changwei Hu
Chaosu Hu
Chaur-Jong Hu
Cheng-Jun Hu
Chenhui Hu
Chunxiang Hu
Dan-Ning Hu

Dongsheng Hu
Dongwei Hu
Fenghua Hu
Fu-Liang Hu
G.Q. Hu
Gang Hu
Guanggan Hu
Guochang Hu
Guocheng Hu
Gwo-Chi Hu
Haitao Hu
Han-Hwa Hu
Hao Hu
Hongli Hu
Hong-Yu Hu
Houchun Hu
Hou-Yuan Hu
Hu Hu
Huanhuan Hu
Jer-Ming Hu
Jiafen Hu
Jian Hu
Jianfang Hu
Jianjun Hu
Jianming Hu
Jianping Hu
Jiaxi Hu
Jie Hu
Jiehui Hu
Jin Hu
Jing Hu
Jinghua Hu
Jinzhou Hu
Juan Hu
Jun Hu
Jun-Feng Hu
Junmei Hu
Kai Hu
Kaijin Hu
Kun Hu
Li Hu
Li-Fang Hu
Longxing Hu
Lufeng Hu
Mao-Bin Hu
Marian Hu
Min Hu
Ming Hu
Ming-Chang Hu
Peng Hu
Pingzhao Hu
Qinghua Hu
Qinhong Hu
Qiong Hu
R. Hu
Rongbin Hu
Rundong Hu
Shang-Ying Hu
Shen Hu
Shengwei Hu
Shengwu Hu
Sherry Shu-Jung Hu
Shiau-Ting Hu
Susan Hu
Tao Hu
Tiffany Hu
Tsung-Hui Hu
Wen-Pin Hu
Wenwei Hu
Xia Hu
Xiao Hu
Xiao Wen Hu
Xiaoli Hu
Xiaolin Hu
Xiaopeng Hu
Xiaoping Hu
Xiaoqing Hu
Xiaoxiang Hu
Xiaoyang Hu
Xinyu Hu
Xiufang Hu
Xiuli Hu
Xuebo Hu
Yali Hu
Yan Hu
Yanru Hu
Yaowu Hu
Yi Hu
Yi-Juan Hu
Yingang Hu
Yin-Gang Hu
Yu Hu
Yue Hu
Yuefei Hu
Yufeng Hu
Yunzhao Hu
Yuxin Hu
Z. Hu
Zanmin Hu
Zhen Hu
Zhen Hua Hu
Zhenghui Hu

Zhihong Hu
Zhiyuan Hu
Binh-Son Hua
Fei Hua
Jingyu Hua
Jinlian Hua
Jinping Hua
Jinshui Hua
Lei Hua
Susan Hua
Xiuguo Hua
Yuchao Hua
Zhihua Hua
Thong Hua-Huy
José Hualde
Jeannette Huaman
Juan Huan
Shitong Huan
Xiujia Huan
Yanjun Huan
Alden Huang
Bing Huang
Bu-Miin Huang
Canhua Huang
Carol Huang
Chang-Ming Huang
Changsheng Huang
Chang-Wen Huang
Changzhi Huang
Chao Huang
Chao Hui Huang
Chao-Li Huang
Cheng-Ya Huang
Chen-Yu Huang
Chien-Hua Huang
Chih-Yang Huang
Ching-Ying Huang
Chiun-Sheng Huang
Chi-Ying Huang
Chuangxia Huang
Chung-Feng Huang
Chun-Jen Huang
Chun-Ta Huang
Chu-Ren Huang
Congxin Huang
Daisie Huang
David Huang
Dongyang Huang
Fenghong Huang
Fu-Chen Huang
Fuqiang Huang
Fuxin Huang
Gan Huang
Gloria Huang
Greg Huang
Guangbin Huang
Guomin Huang
H. Huang
Hailiang Huang
Haiming Huang
Haishui Huang
Haiyan Huang
Hao-Jen Huang
Helai Huang
Henry Huang
Heqing Huang
Hong Huang
Hong-Yuan Huang
Hsiang-Wen Huang
Hsien-Da Huang
Hsin-Cheng Huang
Hsuan-Ming Huang
Huan Huang
Hui Huang
Hui-Chun Huang
Jee-Fu Huang
Jian Huang
Jianhui Huang
Jianliang Huang
Jiean Huang
Jing Huang
Jiun-Yan Huang Huang
Jun Huang
Junqi Huang
Kai Huang
Kai-Wen Huang
Keke Huang
Kevin Huang
Kunlun Huang
Laurence Huang
Lei Huang
Libin Huang
Lijie Huang
Linfang Huang
Ling Huang
Lingkang Huang
Linshan Huang
Liquan Huang
Li-Tung Huang
Longbin Huang
Lu Huang
Luqi Huang

Meng-Chuan Huang
Min Huang
Mingjin Huang
Ning Huang
Paul Huang
Peter H. Huang
Pintong Huang
Po-Hsien Huang
Po-Hsun Huang
Qiang Huang
Qiaobing Huang
Qichao Huang
Qi-Liang Huang
Qinghui Huang
Qiu Huang
Renhua Huang
Rixiang Huang
Ruby Yun-Ju Huang
S.X. Huang
San-Yuan Huang
Shan Huang
Shaoying Huang
Shau-Ku Huang
Shengfeng Huang
Sheng-Jun Huang
Shiang-Fu Huang
Shiang-Lin Huang
Shile Huang
Shiu-Feng Huang
Shi-Wei Huang
Shiyong Huang
Shuang Huang
Shunping Huang
Steve Huang
Steven Huang
Sui Huang
Suming Huang
Tao Huang
Taosheng Huang
Ting-Ting Huang
Tsang-Hai Huang
Tze-Sing Huang
Tzu-Chou Huang
Tzung-Chi Huang
Wei Huang
Wen Huang
Xiaofeng Huang
Xiao-San Huang
Xiaowei Huang
Xiaoxi Huang
Xin Huang
Xinyi Huang
Xinyuan Huang
Xiuzhen Huang
Xiwei Huang
Xuefeng Huang
Xuehui Huang
Yadong Huang
Yafan Huang
Yan Huang
Yao-Ting Huang
Yao-Wei Huang
Yaw-Bin Huang
Yina Huang
Ying-Hsien Huang
Yingxin Huang
Yiran Huang
Yi-Wen Huang
Yong Huang
Yongxiang Huang
Youjia Huang
Yuan Huang
Yuan-Li Huang
Yuanyuan Huang
Yu-Jei Huang
Yunda Huang
Yung-Kai Huang
Yvonne Huang
Zachary Huang
Zhaofeng Huang
Zhiqing Huang
Zhongwei Huang
Zi-Gang Huang
Zuyi (Jacky) Huang
Wei-Chun HuangFu
Bertrand Huard
Aurélie Hua-Van
Nicholas Hubbard
Rebecca Hubbard
Stevan Hubbard
Timothy Hubbard
Mark Hubbe
Paula Hubber
Ryan Hubble
Florent Hube
Carl Hubel
Amy Huber
Brian Huber
Jason Huber
Joel Huber
Julie Huber
Katrin Huber

Kurt Huber
Lars Huber
Marcel Huber
Michael Huber
Peter Huber
Sally Huber
Tobias Huber
Jan Hubert
Nicolas Hubert
Robert Hubley
C.L. Hubley-Kozey
Norbert Hübner
Ronald Hübner
Charles Hubscher
Megan Huchko
Ferdinand Hucho
Christian. Huck
Olivier Huck
Rachel Huckfeldt
Laura Huckins
Luis Huckstadt
Tanvir Huda
Katalin Hudak
Gregory Hudalla
Sophie Huddart
Daniel Huddleston
Tara Hudiburg
Teresa Hudock
Denis Hudrisier
Amanda Hudson
Andrew Hudson
Briony Hudson
Damien Hudson
Darrell L. Hudson
Gavin Hudson
James Hudson
Marie Hudson
Matthew Hudson
Robert Hudson
Robyn Hudson
Isabelle Hue
Sophie Hue
Stephane Hue
Peter Huebener
Ryan Huebinger
Cynthia Huebner
David Huebner
Kay Huebner
Rudolf Huebner
Rachel Huelin
Dueng-Yuan Hueng
Arturo Huerta
Sergio Huerta
Alicia Huerta-Chagoya
Anne-Caroline Hueser
Lynn Huestegge
Guillemette Huet
Sylvie Huet
Maria Huete-Ortega
Robert Hueter
Fabian Huettig
Joel Huey
Murray Huff
Damien Huffer
Derek Huffman
Lynne Huffman
Gary Huffnagle
Matthew Hufford
Koen Hufkens
Christopher Hug
Jane Huggins
Jonathan Huggins
Adam Hughes
Alun Hughes
Bob Hughes
Charles Hughes
David Hughes
Grant Hughes
James Hughes
Jane Hughes
Kerri-Ann Hughes
Matthew Hughes
Michelle Hughes
Sara Hughes
Myra Hughey
Martin Hugh-Jones
Richard Hughson
Jean-Philippe Hugnot
Christian Hugo
Julien Hugon
Elisabeth Huguet
Florence Huguet
Jun Huh
James Huhta
Ilpo Huhtaniemi
David Hui
Elliot Hui
Fei Hui
Jerome Ho Lam Hui
Kenrie Hui
L.L. Hui
Linping Hui

Pei Hui
Zhang Hui
Zi Hui
Laetitia Huiart
Kristi Huik
Heikki Huikuri
Eugénie Huillet
Barbara Huisamen
Monique Huisman
Bionka Huisstede
Philippe Hujoel
Sabine Huke
Vladimír Hula
Scot Hulbert
Jiri Hulcr
Mahmoud Huleihel
Christine Hulette
Rebecca Hull
Vanessa Hull
John Hulleman
Philippa Hulley
Katherine Hullsiek
Gerben Hulsegge
Amanda Hulse-Kemp
Darrin Hulsey
Marcel Hulst
Rebecka Hultgren
Claes Hultling
Jelmer Humalda
Mir Humayun
Sajid Humayun
James Humble
Anne L. Hume
Sandrine Humez
Edith Hummler
Helmut Hummler
Christian Humpel
Hans-Ulrich Humpf
Jay Humphrey
Timothy Humphrey
Lee Humphreys
Paul Humphreys
Austin Humphries
Debbie Humphries
Mark Humphries
R. Keith Humphries
Romney Humphries
Anna K. Hundsdoerfer
Jean-Francois Huneau
Chao-Hung Hung
Chi Hung
Chi-Chih Hung
Chien-Ching Hung
Chih-Hsing Hung
Kun-Long Hung
Kuo-Hsiang Hung
Ling-Shen Hung
Min-Sheng Hung
Noelyn Hung
Pei-Hsuan Hung
Rayjean Hung
Samuel Hung
Sandy Hung
Shu-Chen Hung
Shuen-Iu Hung
Szu-Chun Hung
Yen-Con Hung
Christina Hunger
Portia Hunidzarira
Sabine Hunke
Gary Hunninghake
Elizabeth Hunsperger
Andrew Hunt
Brian Hunt
Chris Hunt
Dana Hunt
Edmund Hunt
George Hunt
Gillian Hunt
Harriet Hunt
John Hunt
P.S. Hunt
Peter Hunt
Randy Hunt
Steven Hunt
George Hunt Jr.
Chad Hunter
David Hunter
Gary R Hunter
Gillian Hunter
Ginger Hunter
James Hunter
MaryCarol Hunter
Myra Hunter
Ryan Hunter
Tony Hunter
Wayne Hunter
Felicity A. Huntingford
Scot Huntington
Rafaële Huntjens
Jason Huntley
László Hunyady

Mary Hunzicker-Dunn
Kaifu Huo
Li-Jun Huo
Ran Huo
Yunlong Huo
Minna Huotilainen
Ted Hupp
Berthold Huppertz
Peter Huppke
Muhammad Huq
Dae Young Hur
Pilwon Hur
Katarzyna Hura
Juan Hurle
Brian Hurley
E. Hurley
James Hurley
Katrina Hurley
Marja Hurley
Neil Hurley
Robert Hurley
Mark Hurlstone
Cameron Hurst
Douglas Hurst
Greg Hurst
Samia Hurst
Christopher Hurt
Reyna Hurtado
Matthew Hurteau
Mary Hurwitz
Ahsan Husain
Fohad Husain
Lewis Husain
Matloob Husain
Daniela Husarik
Steffen Husby
Vojtech Huser
Aydin Huseynov
Jesse Husk
Daniel Huson
Janice Huss
Arif Hussain
Khalid Hussain
M. Iftikhar Hussain
Shamima Hussain
Syed Hussain
Tajammul Hussain
Abid Hussanan
Khaled Hussein
Ahmed Hussen
Claudia Husseneder
Juliette Hussey
Henrik Husted
Laura Huston
Michael Huston
Robert Huston
Wilhelmina Huston
Joshua Hutcheson
Joshua D Hutcheson
Steven Hutcheson
David Hutchins
Michael Hutchins
Sean Hutchins
Raymond Hutchinson
Lindsay Hutley
Alan Hutson
Malo Hutson
Michael Hutson
Darren Hutt
Maik Huttemann
Anna Huttenlocher
Adam Huttenlocker
O. Huttin
David Hutton
Mara Hutz
Hugo C. Huurdeman
Johanna Huusko
Sharon Huws
Mark Huxham
Roger Huybrechts
Kris Huygen
Flavia Huygens
Ho Huynh
Vân Anh Huynh-Thu
Anne-Mette Hvas
Christian Hvas
Andrew Hwang
Chin-Fa Hwang
Chung-Feng Hwang
Geum-Sook Hwang
Han Sung Hwang
Hyisung Hwang
Hyonson Hwang
Jenn-Kang Hwang
Jimee Hwang
Jiwon Hwang
Juey-Jen Hwang
Jun-Eul Hwang
Jung Joo Hwang
Jungwook Hwang
Kwang Yeon Hwang
Lee-Ching Hwang

Lu-Yu Hwang
Ming-Jing Hwang
Pung-Pung Hwang
Rosa Hwang
Soon Hwang
Tae Hyun Hwang
Tsong-Long Hwang
Tzung-Jeng Hwang
Tzyh-Chang Hwang
Wen-Juh Hwang
Wen-Li Hwang
Young Hoon Hwang
Youngdeok Hwang
Dae-Sung Hwangbo
Wuh-Liang Hwu
Khaled Hyari
Kevin Hybiske
David Hyde
Janet Hyde
Jenny Hyde
Melissa Hyde
R. Katherine Hyde
Emily Hyle
Phillip Hylemon
James Hyman
Michael Hyman
Paul Hyman
Weston Hymas
Tim Hyndman
Richard Hynes
Ulla Hynönen
Kullervo Hynynen
Masamitsu Hyodo
Susumu Hyodo
Stephen Hyslop
Richard Hyson
David Hyten
Sang-Hwan Hyun
Kim Hyun Koo
Giovanni Iacca
Guido Iaccarino
Matthew Iacchei
Chiara Iacconi
Tina Iachini
Ioan Iacovache
Daniela Iacoviello
Licia Iacoviello
Massimo Iacoviello
Stefania Iametti
Adriana Iamnitchi
Ovidiu Iancu
Giuseppe Ianiri
Alessandro Iannaccone
Susan Iannaccone
Sergio Iannazzo
M.A. Iannelli
Natale Ianno
Fabio Iannotti
Lora Iannotti
Felice Iasevoli
Glenn Iason
Gladys Ibanez
Antonio Ibañez
Javier Ibañez
Inés Ibáñez
Sebastián Ibáñez Vodnizza
Alejandro Ibáñez-Costa
Noelia Ibanez-Escriche
Jorge Ibanez-Gijon
Masanobu Ibaraki
Borja Ibarra
Fernando R Ibarra
Jose Ibeas
Adriana Ibelli
Tobias Ibfelt
Ibinabo Ibiebele
Mohamed Ibrahem
Ali Ibrahim
Ehab Ibrahim
George Ibrahim
Halah Ibrahim
Salam Ibrahim
Amir Ibrahimagic
Turgay Ibrikci
Yoko Ibuka
Yuko Ibuki
Larry Icard
John Ice
Adriane Icenhour
Joseph Icenogle
Eric Ichesco
Viraj Ichhaporia
Narita Ichiei
Masamitsu Ichihash
Hirohito Ichii
Hiroshi Ichimura
Mayuko Ichimura
Kunihiro Ichinose
Tomomi Ichinose
Toshiharu Ichinose
Stefanie Ickert-Bond
Jaime Ide

Tatsuya Ide
Tomomi Ide
Evgeny Idelevich
Folami Ideraabdullah
Marco Idiart
Susan Idicula-Thomas
Djaudat Idiyatullin
Ellen Idler
Alexander Idnurm
Yasuo Ido
Miguel Angel Idoate
Olubukola Idoko
O.K. Idowu
Muhammad Idrees
Dominika Idziak
Ichiro Ieiri
Motoyuki Iemitsu
Riccardo Ientile
Demian Ifa
Benjamin Iffland
Shafia Iftekhar
Thomas Iftner
Jun-ichi Iga
Kazuhiko Igarashi
Tasuku Igarashi
Takeshi Igawa
Larry Igl
Ana Iglesias
Isabel Iglesias
José Roberto Iglesias
Mar Iglesias
P. Iglesias
Alejandro Iglesias Linares
Bernhard Iglseder
Maria Ignatieva
Anita Ignatius
Alexander Ignatov
Robert Igo Jr.
Oleg Igoshin
Marina Igreja
Seitaro Iguchi
Taisen Iguchi
Tatyana Igumenova
Hidekatsu Iha
Friedrich Ihler
Michael Ihnat
Timothy Ihongbe
Satoshi Ii
Kazuhiro Iida
Kyo Iigaya
Hideki Iijima
Katsunori Iijima
Katsuya Iijima
Kazumoto Iijima
Yuichi Iino
Antti Iivanainen
Hisashi Iizasa
Shigeho Ijiri
Takashi Ijiri
H Ijsselstijn
Hans Ijzerman
Jan N. M. Ijzermans
Katsunori Ikari
Yuji Ikari
Masahito Ikawa
Akihiro Ikeda
Kazunari Ikeda
Kei Ikeda
Kota Ikeda
Takeshi Ikeda
Toshiyuki Ikeda
Yoichiro Ikeda
Tempei Ikegame
Kohta Ikegami
Mitsunori Ikeguchi
Melanie Ikeh
Susumu Ikehara
Rona Ikehara-Quebral
Takehito Ikejiri
Kristian Ikenberg
T. Alp Ikizler
Vasiliki Ikonomidou
John Ikonomopoulos
Shin-ichi Ikushiro
Togo Ikuta
Toshikazu Ikuta
Lena Ilan
Micha Ilan
Harry Iland
Andrey Ilatovskiy
Govindasamy Ilavazhagan
Adeodat Ilboudo
Uwe Ilg
Winfried Ilg
Milena Ilic
Jasminka Ilich-Ernst
Lucian Ilie
Dimitrios Iliopoulos
Ozlem Ilk
Xavi Illa
Peter Illes
Hazlee Illias

Gudrun Illmann
Giulio Illuminati
Babatunde Ilori
Titilayo Ilori
Heh-In Im
Kyoungbin Im
Sung Gap Im
Enyu Imai
Hiroo Imai
Hissei Imai
Katsushi Imai
Kazushi Imai
Shin-ichiro Imai
Takeshi Imai
Yumi Imai
Kazunori Imaizumi
Yuji Imaizumi
F. Imamura
Fumiaki Imamura
Fumihiko Imamura
Hiromi Imamura
Hiroshi Imamura
Minako Imamura
Yutaka Imamura
Toshiyuki Imasawa
Arkaitz Imaz
Isabelle Imbert
Véronique Imbert
Anne Imberty
Bruno Imbimbo
Carol Imbriano
Roland Imhoff
James Imlay
Jean-Luc Imler
Stephan Immenschuh
Maarten Immink
Richard Immink
Genji Imokawa
Andrea Imperatori
Claudio Imperatori
Francesco Imperi
Juan Imperial
Belen Imperiale
Simona Imperio
Soren Impey
Dante Implicito
Asma Imran
Mudassar Imran
Albert Imsland
Maria Imtiaz
Mohammad Imtiaz
Ryo Inada
Takayuki Inagaki
Takeshi Inagaki
Yoshinori Inagaki
Behcet Inal
Takashi Inamoto
Nagihan Inan
Takashi Inaoka
Can Ince
Hüseyin Ince
Ümit Incekara
Sara Incera
Pablo Inchausti
Lewis Incze
Peter Indefrey
Inderjit Inderjit
Ciro Indolfi
Giuseppe Indolfi
Sabarish Indran
Dasgupta Indranil
Pillaveetil Sathyadas Indu
Nibaldo Inestrosa
Claire Infante -Rivard
E. Infusino
Alberto Inga
Chantal Ingabire
Landon Inge
Magnus Ingelman-Sundberg
Pablo Ingelmo
Anne Ingenbleek
Y. Ingenbleek
Kate Ingenloff
Richard Inger
Victoria Ingham
Suzanne Ingle
David Ingleby
Chris Inglehearn
Marta Ingles
Matthew Inglis
Timothy Inglis
Alyssa Ingmundson
Katherine Ingram
Simon Ingram
Travis Ingram
Ylenia Ingrasciotta
Erin Ingvalson
Par Ingvarsson
Klaus Ingvartsen
Marcia Inhorn
Loretta Iniaghe
Gerardo Iñiguez

Sergio Iñiguez
Iwona Inkielewicz-Stêpniak
Denise Inman
Michele Innangi
David Innes
John Innes
Steve Innes
Hamish Innes-Brown
Bernardo Innocenti
Marco Innocenti
Metello Innocenti
Thomas Inns
Naohiro Inohara
Mikito Inokuchi
Haruhisa Inoue
Hideki Inoue
Hirosuke Inoue
Hiroyasu Inoue
Jun-ichiro Inoue
Kentaro Inoue
Maiko Inoue
Masayuki Inoue
Naoki Inoue
Nobutaka Inoue
Rintaro Inoue
Shiji Inoue
Shin-Ichi Inoue
Shintaro Inoue
Teruo Inoue
Tsuyoshi Inoue
Yasuhiro Inoue
David Inouye
Michael Inouye
Robert Insall
Heribert Insam
Maite Insausti
Andrea Introini
Masayuki Inui
Baba Inusa
Pietro Invernizzi
Thomas Inzana
Rivka Inzelberg
Ioannis Ioakimidis
Andreea Ioan-Facsinay
Ioannis Ioannidis
Andriani Ioannou
Christos Ioannou
Efstathia Ioannou
Pier Paolo Iodice
Elena Iomdina
Clara Ionescu
Radu Ionescu
Ed Ionides
Mihaela Iordanova
Egidio Iorio
Massimo Iorizzo
Dan Iosifescu
George Iosifidis
Maria Rosaria Iovene
M. Kathryn Iovine
Radu Iovita
Renato Iozzo
Dennis Ip
Hon Ip
Philip Ip
Giovanbattista Ippoliti
Rodolfo Ippoliti
Antonio Ippolito
Anwar Iqbal
Ehtesham Iqbal
Javeed Iqbal
Jawed Iqbal
M. Iqbal
Najeeha Iqbal
Omer Iqbal
Ramsha Iqbal
Syed Iqbal
Zafar Iqbal
Maria Iraburu
Srinivas Iragavarapu
Vijaya Iragavarapu-Charyulu
David Irani
Esperanza Iranzo
Luis Irastorza Eguskiza
Omid Iravani
Shahriar Iravanian
Susan Ireland
Joel Ireta
M. Okan Irfanoglu
Jose Iriarte-Diaz
Rafael Iribarren
Manuel Irimia
Tatsuro Irimura
Andrey Irintchev
Brian Irish
Brian Iritani
Ryosuke Iritani
Kristopher Irizarry
Sandra Irmisch
M.O. Iroezindu
Pui-Ying Iroh Tam
Maria Ironside

Andres Iroume
Carlos Irribarren
Elizabeth Irungu
Kenneth Irvine
Scott Irvine
Brian Irving
Hyacinth Irving
Amy Irwin
Andrew Irwin
Christopher Irwin
David Irwin
Jane Irwin
Adam Isaac
Mathew Isaac
Nick Isaac
Aaron Isaacs
Charles Isaacs
Kim Isaacs
Sandra Isabel
Maria Isaguliants
Ioannis Isaias
Sven Isaksson
Christina Isaxon
Lynne Isbell
Geoffrey Isbister
Sachiko Iseki
Esma Isenovic
Ana Iseppon
Berend Isermanm
Joan Isern
Sharon Isern
Peter Isfort
Shareena Ishak
Waguih Ishak
Yoshitaka Ishibashi
Kazuto Ishida
Yoji Ishida
Akemi Ishida-Yamamoto
Masami Ishido
Hideyuki Ishiguro
Hiroki Ishiguro
Hiroshi Ishiguro
Hisamitsu Ishihara
Junko Ishihara
Kazuyuki Ishihara
Masaharu Ishihara
Shuji Ishihara
Daisuke Ishii
Haruyuki Ishii
Hiroki Ishii
Hiroko Ishii
Kenichi Ishii
Masaru Ishii
Shinya Ishii
Takeshi Ishii
Tetsuya Ishii
Reiko Ishii-Nozawa
Goro Ishikawa
H. Ishikawa
Naoto Ishikawa
Riyuuji Ishikawa
Satoru Ishikawa
Tetsuya Ishikawa
Yasuyuki Ishikawa
Rieko Ishima
Yoshiko Ishimi
Toshihiko Ishimitsu
Takatsugu Ishimoto
Takuya Ishimoto
Eiji Ishimura
Tomoko Ishino
Alessio Ishizaka
Sally Ishizaka
Kimitsune Ishizaki
Yasuki Ishizaki
Akihiro Ishizu
Tauheed Ishrat
Andrea Isidori
Dalia Iskander
Ahsan Islam
Ishrat Islam
Md Islam
Md. Mofakkarul Islam
Mohammad Islam
Mohammad Safiqul Islam
Rafiq Islam
Shahidul Islam
SK Hafizul Islam
Tanvir Islam
Leon Islas Suarez
Krishna Isloor
A. Ismail
Ayesha Ismail
Hanafy Ismail
Mohannad Ismail
N.A. Ismail
Nahed Ismail
Salmah Ismail
Shehab Ismail
Wael Ismail
Bob Isman
Murray Isman

Stefanie Ismar
Tatsuya Iso
Kazuo Isobe
Sachiko Isobe
Masanori Isogawa
Yoichiro Isohama
Hajime Isomoto
Michael Ison
Esther Isorna
David Israeli
Abukari Issaka
Ayuba Issaka
Eric Isselbacher
Sujay Subbayya Ithychanda
Akinori Ito
Hiroki Ito
Hiromu Ito
Hiroyasu Ito
Junya Ito
Katsuhiko Ito
Keiichi Ito
Ken-ichi Ito
Koichi Ito
Nobutoshi Ito
Osamu Ito
S. Ito
Satoru Ito
Satoshi Ito
Shigeki Ito
Tetsufumi Ito
Wulf Ito
Yasuhiko Ito
Yasuhiro Ito
Yoko Ito
Yoshihiro Ito
Yutaka Ito
Junichi Itoh
Takeshi Itoh
Eiji Itoi
Richard Ittenbach
Lars Ittner
Rodrigo Iturriaga
Miren Iturriza-Gómara
Jennifer Iudicello
Rodolfo Iuliano
Cristina Ivan
Mircea Ivan
Lacramioara Ivanciu
Viatcheslav Ivanenko
Andrada Ivanescu
Vladimir Ivanisenko
Julijana Ivanisevic
Alexander Ivanov
Alexey Ivanov
Andrei Ivanov
Delyan Ivanov
Ivan Ivanov
Pavel Ivanov
Plamen Ivanov
Valeriy Ivanov
Diana Ivanova
Natalia Ivanova
Tatyana Ivanovska
Saso Ivanovski
Maksym Ivanya
Angela Ivask
Domagoj Ivastinovic
Rebecca Ivers
Jenny Iversen
John Iversen
L. Iversen
Maura Iversen
Christopher Ives
Rachel Ives
Alan Ivkovic
Vladimir Ivkovic
Antoni Ivorra
Benjamin Ivorra
J. Ivory
Ioannis Ivrissimtzis
Jamie Ivy
Masato Iwabu
Kikuo Iwabuchi
Masao Iwagami
Atsushi Iwai
Hideo Iwai
Moriya Iwaizumi
Hiro-oki Iwakawa
Atsushi Iwama
Takayuki Iwamoto
Takuya Iwamura
Masako Iwanaga
Ritsuko Iwanaga
Urszula Iwaniec
Andrew Iwaniuk
Shingo Iwano
Takahiro Iwao
Kengo Iwasaki
Kenta Iwasaki
Koh Iwasaki
Masanori Iwasaki
Yasumasa Iwasaki

Akira Iwase
Yoshio Iwashima
Akira Iwata
Atsushi Iwata
Hiroshi Iwata
Koichi Iwata
Minoru Iwata
Takashi Iwata
Yasuhide Iwata
Yasunori Iwata
Hirotsugu Iwatani
Emilia Iwu
Conrad Iyegbe
Puneeth Iyengar
Raghuram Iyengar
Aparna Iyer
Arun Iyer
Jyoti Iyer
Lakshmanan Iyer
Malliga Iyer
M. Iyyappan
Felipe Iza
Nadia Izadi-Pruneyre
Reza Izadpanah
Fariborz Izadyar
Benjamin Izar
Jacques Izard
Jun Izawa
Adam Izdebski
David Izquierdo-Garcia
Kenji Izuhara
Hirohisa Izumi
Keisuke Izumi
Yasuhiro Izumiya
Cristina Izura
Ken-ichi Izutsu
Alberto Izzotti
William Ja
Liiro Jaaskelainen
Suha Jabaji
Fereshteh Jabalameli
Sairam Jabba
Esmaiel Jabbari
Ramazan Jabbarli
Ammar Jaber
Mohamed Jaber
David Jablons
Agata Jablonska-Trypuc
Nina Jablonski
Mary Ann Jabra-Rizk
Rodrigo Jacamo
Cynthia Jacelon
David Jachowski
Estela Jacinto
Bejamin Jack
Thomas Jack
Connie Jackaman
Markus Jäckel
Robert Jackler
Joshua Jackman
William Jackman
Andrew Jackson
Ben Jackson
Benjamin Jackson
Catherine L. Jackson
Charlene R. Jackson
Colin Jackson
George Jackson
Graham Jackson
James Jackson
Jeffrey Jackson
Lisa Jackson
Louise Jackson
Mandy Jackson
Michael Jackson
Michelle Jackson
Monica Jackson
Philip Jackson
Philippa Jackson
Rodney Jackson
Sarah Jackson
Shelley Jackson
Terri Jackson
Vaughn Jackson
Walker Jackson
William Jackson
Mark Jackwood
Francis Jacob
George Jeryn Jacob
Merle Jacob
Nicholas Jacob
Ralf Jacob
Rojymon Jacob
James Jacobberger
Aaron Jacobs
Anna Jacobs
Ashley Jacobs
Bertram Jacobs
Cale Jacobs
Christopher Jacobs
Daniel Jacobs
David Jacobs

Elizabeth Jacobs
Jonathan Jacobs
Julia Jacobs
Lotte Jacobs
Maartje Jacobs
Michael Jacobs
W. Bradley Jacobs
William Jacobs
Zenobia Jacobs
David Jacobs Jr.
Anne Jacobsen
Jette Bredahl Jacobsen
Julius Jacobsen
Kathryn Jacobsen
Nis Jacobsen
Thomas Jacobsen
Alana Jacobson
Cindy Jacobson
David Jacobson
Elaine Jacobson
Jeffrey Jacobson
Karen Jacobson
Kevan Jacobson
Magdalena Jacobson
Mark Jacobson
Myrna Jacobson
Sandra Jacobson
Steven Jacobson
Gunnar Jacobsson
Marc Jacofsky
A.A. Jàcome
Michael Jacox
Helene Jacqmin-Gadda
Vincent Jacquemet
Benedicte Jacquemin
Marc Jacquemin
Vincent Jacquemond
Hans Jacquemyn
Claire Jacques
Constanza Jacques
Frédéric Jacques
Peter Jacques
Steven Jacques
Alain Jacquet
Jennifer Jacquet
Pierre Jacquet
Herve Jacquier
Marine Jacquier
Emmanuelle Jacquin-Joly
Maude Jacquot
Jacqueline Jacysyn
Janusz Jadasz
Sudarshan Jadcherla
Jyoti Jadhav
Rohit Jadhav
Snehal Jadhav
Sae Jae
Volker Jaedicke
Herbert Jaegle
John Jaenike
Hartmut Jaeschke
Tazeen Jafar
Behrouz Jafari
Mahtab Jafari
Peyman Jafari
Zahra Jafari
Majid Jafari Khaledi
Miran Jaffa
Klaus Jaffe
Aravindakshan Jagadeesan
Pradeep Babu Jagadeesh Reddy
Santosh Jagadeeshan
Guru Jagadeeswaran
Pudur Jagadeeswaran
Ramasamy Jagadeeswaran
Aarti Jagannath
Madan Jagasia
Franc Jager
Benedikt Jäger
Tomasz Jagielski
Krzysztof Jagla
Susan Jaglal
Elzbieta Jagusztyn-Krynicka
Ismat Jahan
Sadaf Jahan
Leila Jahangiry
Hesamoddin Jahanian
Rahman Jahanian
Faezeh Jahanmiri-Nezhad
Dieter Jahn
Klaus Jahn
Regine Jahn
Helena Jahncke
Michael Jahncke
Lisa Jahns
Peter Jahns
Gustuv Jahoda
Teresa Jaijo
Enrique Jaimovich
Aklank Jain
Amish Jain
Arsh Jain

Deepti Jain
Gaurav Jain
Neeta Jain
Pawan Jain
Priyesh Jain
Rajeev Jain
Rekha Jain
Sanjay Jain
Vikas Jain
Stephanie Jainta
Mohamad Jairajpuri
Teele Jairus
Amritha Jaishankar
Dinesh Jaishankar
Richa Jaiswal
Vandana Jaiswal
Blessing Jaja
Anjana Jajoo
Anne Jakel
Danielle Jake-Schoffman
Mark Jakiela
Martina Jakob
Stefan Jakobs
Tatjana Jakobs
Jette Jakobsen
Johannes Jakobsen
Magnus Jakobsson
Catarina Jakovac
Vladimir Jakovljevic
Jernej Jakse
Jill Jaksetic
Fabian Jaksic
Andrzej Jakubczyk
Nicholas Jakubovics
Daniela Jakubowicz
Anna Jakubowska
Aleksandra Jakubowski
Kelly Jakubowski
Claudia Jakubzick
Diana Jalal
Niloufar Jalali Moghadam
Estelle Jaligot
Rohana Jalil
Juho Jalkanen
Sirpa Jalkanen
Archana Jalligampala
George Jallo
Vincent Jallu
Rahman Jamal
Fakhreddin Jamali
Jamshid Jamali
Alexander James
Andrew James
Eddie James
Elizabeth James
Euan James
Gary James
Harvey James
Joshua James
Judy James
Karin James
Louisa James
Margaret James
Michael James
Ogutu James
Philip James
Steve James
Thomas James
Timothy James
William James
Laurie James-Hawkins
Geoffrey Jameson
Kimberly Jameson
Tamara James-Todd
Elie Jamie
Nigel Jamieson
Kaiser Jamil
Muhammad Jamil
Keith Jamison
Frederic Jamme
Abdou Jammeh
Heather Jamniczky
Aria Jamshidi
Deepa Rana Jamwal
Zima Jan
Asis Jana
Karolina Janacsek
Naveena Janakiram
Malvin Janal
Leila Janani
Srinivas Janaswamy
Petr Janata
Margit Janát-Amsbury
Guilhem Janbon
Monika Janczarek
Claudia Janda
Milan Janda
Tibor Janda
Robert Jandl
Jennifer Jandt
Raimon Jané
Stepan Janecek

Michael Janech
Jan Janecka
Nathalie Janel
Holly Janes
Robert Janes
James Janetka
Elena Jovanovska Janeva
Byoung Kuk Jang
Cheol Seong Jang
Hee-Chang Jang
Hyeung-Jin Jang
Jin-Young Jang
Sang-Min Jang
Seogjoo Jang
Seonghoe Jang
Woncheol Jang
Woong Sik Jang
Wooyoung Jang
Yuh Jang
Sarath Chandra Janga
Jaganmohan Jangamreddy
Kamlesh Jangid
Bhautesh Jani
Meghna Jani
Elizabeth Janiak
Reiner Jänicke
Matthew Janicki
Piotr Janik
Philipp Janitza
Jagadeesh Janjanam
Zorica Janjetovic
Yelena Janjigian
Wolfgang Jank
Stacey Jankelowitz
Anuradha Jankiraman
Robert Jankowiak
Matthew Jankowich
Marta Jankowska
Joachim Jankowski
Maciej Jankowski
Piotr Jankowski-Mihulowicz
Michael Jann
Emmanuele A. Jannini
Claire Janoir
Amol Janorkar
Alexis Janosik
Isabelle Janoueix-Lerosey
Miroslaw Janowski
Jan Jansa
Benjamin Janse
Ben Jansen
David Jansen
Gunther Jansen
Jacobus Jansen
John Jansen
Michaela Jansen
Moritz Jansen
Patrick Jansen
Petra Jansen
Ray Jansen
Vincent Jansen
Pidder Jansen-Dürr
Shelley Jansky
Charles Janson
Nomdo Jansonius
Paul Jansons
Christian Janssen
Emiel Janssen
Luke Janssen
Marco Janssen
Matthijs Janssen
Niels Janssen
Patricia Janssen
Paul Janssen
Xanne Janssen
Geert Janssens
Thierry Janssens
Lauren Jansson
Per-Anders Jansson
Jonathan Jantsch
Jennifer Jantz
Fraser Januchowski-Hartley
Christopher Janus
Dorte Janussen
Aleksandra Januszewska
Katy Janvier
Philippe Janvier
Tavan Janvilisri
Kathleen Janz
Bonnie Janzen
Christian Janzen
Uta Jappe
Ainhoa Jarammillo-Gutiérrez
Laura Jarboe
Elliott Jardin
Claire Jardine
Dinchen Jardine
Paul Jardine
Debra Jared
Jacob Jaremko
Narumol Jarernsiripornkul
Sven Jarius

Narissara Jariyapan
Mahita Jarjapu
Andrew Jarman
David P. Jarmolowicz
Artur Jarmolowski
Wieslawa Jarmuszkiewicz
Kamil Jaron
Ernst Jarosch
Beata Jarosiewicz
Paul Jarosinski
David Jarrard
Michael Jarstfer
Veeravich Jaruvongvanich
Hanna Jarva
Erich Jarvis
Jason Jarzembowski
Wolfgang Jaschinski
Anna Jasinska
Magdalena Jasinska-Stroschein
Miriam Jasiulionis
Bernard Jasmin
Lina Jasmontaite
Aaron Jasnow
Cornelia Jaspers
Richard Jaspers
Sarbjit Vanita Jassal
Yasir Jassam
Vincent E.J. Jassey
Sunitha Jasti
Kris Jatana
Devcharan Jathanna
Shantanu Jathar
Amit Jathoul
Edward Jauch
Sameer Jauhar
Matti Jauhiainen
Kathrine Jauregui-Renaud
A.A. Java
Amir-Homayoun Javadi
Pasha Javadi
Sabzali Javadov
Teymour Javaherchi
Khashayar Javaherian
Kassim Javaid
Gulnaz Javan
Taraneh Javanbakht
Marco Javarone
Mustafa Javed
Zeeshan Javed
M. Javorka
Mohammed Jawad
Sofia Jawed-Wessel
James Jawitz
Anthony Jaworowski
Coline Jaworski
Deborah Jaworski
Jacek Jaworski
Philippe Jay
Steven Jay
Panneer Selvam Jayabal
Muthuvel Jayachandran
Selwyn Jayakar
Thayyil Jayakrishnan
R. Jayakumar
Harikrishnan Jayamohan
P. Jayamurthy
B. Jayaram
Arul Jayaraman
Dhileepkumar Jayaraman
Shiva Jayaraman
Thottala Jayaraman
Varshini Jayaraman
Vasanthi Jayaraman
Nishad Jayasundara
Madawa Jayawardana
Lewis Jayes
Avindra Jayewardene
Lee-Ann Jaykus
Bruce Jayne
James Jaynes
Mohammad-Ali Jazayeri
Seyed Jazayeri
Seyed Mehdi Jazayeri
Ali Jazirehi
Anna Jazwinska
S. Jazwinski
Aurelie Jean
Aurelie Jeandron
Angela Jeanes
Freddy Jeanneteau
Jamel Jebali
Larry Jech
Sven Jechalke
Julie Jedlicka
Sun Ha Jee
Gursimer Jeet
Janina Jeff
William Jeffcoate
Kimberly Jefferson
Thomas Jefferson
Constance J Jeffery
Ian Jeffery

David Jeffries
Matlock Jeffries
Andrew Jeffs
Ramprasad Jegadeesan
Chockalinngam Jeganathan
Jean-François Jegou
Jeremie Jegu
George Jeha
Francois Jehl
Beatrice Jeinsen
John Jeka
Bojan Jelakovic
Elissa Jelalian
Musharraf Jelani
Sven Jelaska
Aline Jelenkovic
Joanna Jelenska
Linda Jelicks
Herbert Jelinek
Murray D Jelinski
Kishore Kumar Jella
Eric Jellen
W. Jellish
John-Eric Jelovsek
Jennifer Jelsma
Katalin Jemnitz
Per Jemth
G.B. Jena
Jiiang-Huei Jeng
Mei-Jy Jeng
Wen-Juei Jeng
Rajesh Jeniton
Holger Jenke-Kodama
Gabrielle Jenkin
Alicia Jenkins
Andrew Jenkins
Brooke Jenkins
Cheryl Jenkins
David Jenkins
Helen Jenkins
Jill Jenkins
Louis Jenkins
P. Vince Jenkins
Wiley Jenkins
Graeme Jenkinson
J. Charles Jennette
Garry Jennings
J. Jennings
Jackson Jennings
Megan Jennings
Simon Jennings
Skyler Jennings
Poul Jennum
Matt Jenny
Nancy Jenny
Andrew Jensen
Bo Jensen
Boye Jensen
Brian Jensen
Carsten Jensen
Esther Jensen
Evelyn Jensen
Helen Jensen
Henrik Jensen
Henry Jensen
Jacob Jensen
Jamie Jensen
Jan Kristian Jensen
Jens-Ulrik Jensen
Keith Jensen
Klavs Jensen
Kristian Kiim Jensen
Kristin Jensen
Kristoffer Jensen
Lasse Jensen
Lora M. Jensen
Megan Jensen
Michael Jensen
Oliver Jensen
Paul Jensen
Randy Jensen
Taylor Jensen
Thomas Jensen
Vivi Jensen
Erika Jensen-Jarolim
Matthias Jentschke
Sabrina Jenull
Christie Jeon
Hong Jin Jeon
Kyeongman Jeon
Seong Woo Jeon
Taejoon Jeon
Chang Wook Jeong
Choongwon Jeong
Hee-Jeong Jeong
Hyunyoung Jeong
Juhee Jeong
Kwang Cheol Jeong
Kwang Won Jeong
Myung Ho Jeong
Soung Won Jeong
Sujong Jeong

Sunjoo Jeong
Yong Seok Jeong
Erik Jeppesen
Karl Jepsen
Patricio Jeraldo
Christopher Jerde
Nadia Jeremiah
Albert Jergens
Igor Jerkovic
Susanna Jernelöv
David Jernigan
Robert Jernigan
Terry Jernigan
Valarie Jernigan
Bertus Jeronimus
Carsten Jers
Guy Jerusalem
Lori Jervis
Gunnar Jeschke
Marc Jeschke
Kristjan Jessen
Sarah Jessen
Kwok Jessica
Ryan Jessup
Ederson Jesus
Jitendra Jethani
Nathalie Jette
Anton Jetten
Espen Jettestuen
Marie-Helene Jeuffroy
Julie Jeukens
Marc Jeuland
Camille Jeunet
Eui-Bae Jeung
Derek Jewell
Sarah Jewell
Michael Jewett
Aaron Jex
Ayyamperumal Jeyaprakash
William Jeynes
Aashish Jha
Awadhesh Jha
Dhruva Kumar Jha
Gopaljee Jha
Prabhat Jha
Pranaw Kumar Jha
Prashant Jha
Priyanka Jha
Rajesh Jha
Rajesh Kumar Jha
Uday Jha
Yadvendradev Jhala
Niyati Jhaveri
Bao Ji
Butian Ji
Daode Ji
Fei Ji
Feng Ji
Hong Ji
Hongbin Ji
Honglong Ji
Jianguang Ji
Jianguo Ji
Kun Ji
Lei Ji
Lili Ji
Lin Ji
Peng Ji
Ruijun Ji
Songbai Ji
Xiaoxi Ji
Xiaoyang Ji
Yewei Ji
Yong Ji
Zai-Si Ji
Zhi-Liang Ji
B. Jia
Fanli Jia
Jianhang Jia
Jie Jia
Junjing Jia
Lin Jia
Linpei Jia
Rong Jia
Tao Jia
Wangcun Jia
Wei-Hua Jia
Wenkai Jia
Xia Jia
Xiaoshan Jia
Xu Jia
Yanbin Jia
Yulin Jia
Zhenquan Jia
Zhilong Jia
Zheng Jiali
Cao Jian
Yifan Jian
Zhen Jian
Baoming Jiang
Bo Jiang
Chunling Jiang

Daohong Jiang
Dianhua Jiang
Dong Jiang
Gangyi Jiang
Guihua Jiang
Guirong Jiang
Guo-Liang Jiang
Guosheng Jiang
Haibo Jiang
Haiteng Jiang
Haobo Jiang
Helong Jiang
Hongen Jiang
Hongmei Jiang
Houbo Jiang
Houshuo Jiang
Hui Jiang
Jack Jiang
Jean Jiang
Jheng-Jie Jiang
Jiafu Jiang
Jinjin Jiang
Joy Jiang
Lei Jiang
Lianghai Jiang
Libo Jiang
Lili Jiang
Lixi Jiang
Lixin Jiang
Luo-Luo Jiang
Peihua Jiang
Pengyao Jiang
Qi Jiang
Qing Jiang
Qinghua Jiang
Qiuping Jiang
Rays Jiang
Renyan Jiang
Rulang Jiang
Shibo Jiang
Shigui Jiang
Shiguo Jiang
Song Jiang
Tingting Jiang
Wei Jiang
Wenjie Jiang
Xi Jiang
Xia Jiang
Xiang-Chen Jiang
Xiao Hua Jiang
Xiao Zhen Jiang
Xiaodong Jiang
Xiaogang Jiang
Xiaoyan Jiang
Xing Fu Jiang
Yangzi Jiang
Yanliang Jiang
Yannan Jiang
Yan-Yi Jiang
Yi Jiang
Yinan Jiang
Yiwei Jiang
Yizhang Jiang
Yong Jiang
Yonghou Jiang
Yu Jiang
Yuan Jiang
Yuan-Qing Jiang
Yueyang Jiang
Yun-Jin Jiang
Yusheng (Jason) Jiang
Yuwei Jiang
Zhaozhao Jiang
Zhenghui Jiang
Zhihong Jiang
Zhihua Jiang
Zhi-Qiang Jiang
Zhongyuan Jiang
Zide Jiang
Ziyan Jiang
Wang Jianguang
Li Jianping
Jian Jiao
Junfeng Jiao
Li Jiao
Licheng Jiao
Ping Jiao
Wei-Wei Jiao
Xiaoguo Jiao
Xiaohui Jiao
Xuanmao Jiao
Yang Jiao
Yongjun Jiao
Glen Jickling
Biao Jie
Gong Jie
Wei Jie
Francis Jiggins
Humberto Jijon
Nikolaus Jilg
Jeffrey Jim
Alejandro Jimenez

Carlos Jimenez
Joaquin Jimenez
Juan Jimenez
Juan J Jimenez
M. Angeles Jimenez
Mike Jimenez
Sergio Jimenez
Carlos Jiménez
Diego Jiménez
José Jiménez
Juan Jiménez
Natalia Jiménez
Juan Miguel Jimenez Andrade
Mónica Jimenez-Castro
Miguel Angel Jimenez-Clavero
Rodrigo Jiménez-Garcia
José Jiménez-Heffernan
Félix Javier Jiménez-Jiménez
Pedro Jimenez-Mejias
Pedro Jiménez-Reyes
Joohi Jimenez-Shahed
Lai Jimmy
Cheng Jin
Chongfei Jin
Chunyuan Jin
Dazhi Jin
Dezhe Jin
Di Jin
Dong-Kyu Jin
Fengliang Jin
Fulai Jin
Guanfu Jin
Guangfu Jin
Guangze Jin
Guiyun Jin
Hong Jin
Hongfang Jin
Hui Jin
Jianping Jin
Jie Jin
Jing Jin
Jing Bo Jin
Kunlin Jin
Lei Jin
Lihua Jin
Meilin Jin
Meiyan Jin
Moonsoo Jin
Qiming Jin
Qiyu Jin
Ran Jin
Shaosheng Jin
Shengyu Jin
Shuangxia Jin
Shuoguan Jin
Songqing Jin
Tengchuan Jin
Tianbo Jin
Tong Jin
Wei Jin
Xia Jin
Xiaochun Jin
Xiaowei Jin
Yan Jin
Yi Jin
Yong-Su Jin
Yufang Jin
Zhao Jin
Zhenlan Jin
Zhiqiang Jin
Zi-Bing Jin
Mary Anne Tan Jin Ai
Amit Jinabhai
Rahul Jindal
K. Jindo
Goodwin Jinesh
Fan Jing
Hai-Chun Jing
Ruilian Jing
Yang Jing
Tsung-Luo Jinn
Umesh Jinwal
Chuleeporn Jiraphongsa
Paulin Jirkof
Milan Jirsa
Kim Jiseon
Jiaming Jiu
Aliya Jiwani
Javier Jo
Jun-ichiro Jo
Min-Woo Jo
Michael Joannidis
Taufique Joarder
Dominique Job
Xavier Job
Timothy Jobe
Chacko Jobichen
Andrew Jobling
Malcolm Jobling
Eun-Hye Joe
William Joe
Rolf Joerger

Valerie Joers
Victoria Joffe
Manuel Joffre
Gerwald Jogl
Sarb Johal
Matthew Johannes
Jason Johannesen
Helle Johannessen
Kerstin Johannesson
Gary Johanning
Kerri Johannson
Espen Johansen
Kirsten Johansen
Maria Johansen
Michelle Johansen
Steinar Johansen
Zerina Johanson
Adam Johansson
Anders Johansson
Björn Johansson
Cecilia Johansson
Charlotte Johansson
Frank Johansson
Fredrik Johansson
Hanna Johansson
Henrik Johansson
Jörgen Johansson
Kjell Arne Johansson
Lina Johansson
Mattias Johansson
Marlene Johansson Falck
Sophia Johler
Annie John
Chandy John
Dolly John
Gareth John
Leslie K. John
Oommen John
Susan John
Vijay John
Henry John-Alder
Benjamin Johns
Jill Johnsen
Allison Johnson
Andrew Johnson
Anna Johnson
Arlen Johnson
Asal M. Johnson
Blair T. Johnson
Brian Johnson
Bruce Johnson
Cage Johnson
Catrina Johnson
Chris Johnson
Christopher Johnson
Craig Johnson
Daniel Johnson
David Johnson
Dawn Johnson
Donn Johnson
Dwain Johnson
Edward Johnson
Elizabeth Johnson
Elizabeth K. Johnson
Eric Johnson
Evan Johnson
Greg Johnson
Howard Johnson
Jacob Johnson
James Johnson
James D. Johnson
Jeffrey Johnson
Jerald Johnson
Jill Johnson
Joseph Johnson
Joshua Johnson
Kenneth Johnson
Kevin Johnson
Laura Johnson
Leigh Johnson
Liam Johnson
Maria Johnson
Mark A Johnson
Mike Johnson
Miriam Johnson
Natalie Johnson
Nathan Johnson
Nessa Johnson
Ofonime Johnson
Patrick Johnson
Paul Johnson
Peter Johnson
Philip Johnson
R.J. Johnson
Rabia Johnson
Randall Johnson
Reed Johnson
Renee Johnson
Richard Johnson
Sam Johnson
Simon Johnson
Steven Johnson

Stuart Johnson
Susan Johnson
Teresa Johnson
Timothy Johnson
Victoria Johnson
W. Evan Johnson
Warren Johnson
Wendy Johnson
Martin Johnsson
Per Johnsson
Alison Johnston
Christine Johnston
Christopher Johnston
Christopher. D Johnston
David Johnston
Desmond Johnston
Doug Johnston
Fabian Johnston
Jamie Johnston
Lucy Johnston
Matthew Johnston
Nathaniel Johnston
Randal Johnston
Robert Johnston
Sebastian Johnston
Stuart Johnston
Susan Johnston
Jill Johnstone
Sören Johst
Richard Joiner
Wilsaan Joiner
Merja Jokelainen
Nobuhiko Joki
Paul Jokiel
Vilija Jokubaitis
Darukeshwara Joladarashi
Fariborz Jolai
Jacob Jolij
Geneviève Jolivet
Jukka Jolkkonen
Dietsje Jolles
Didier Jollivet
Kate Jolly
Meenakshi Jolly
Cornelia Jol-van der Zijde
Florence Joly
Jean-Stephane Joly
Philippe Joly
Pierre Joly
Mayuko Jomura
Karpliow Jon
Cabell Jonas
Elisabeth Jonas
Jean-Christophe Jonas
Maureen Jonas
Wayne Jonas
Eric Jonasch
Peter Jonason
Charles Jonassaint
Jon Jonasson
Elisabeth Jonckers
Alex Jones
Andrew Jones
Arwyn T. Jones
Ben Jones
Bradley Jones
Byron Jones
Carolyn Jones
Christopher Jones
Clinton Jones
Cynthia Jones
Darryl Jones
David Jones
Deborah Jones
Douglas Jones
Elena Jones
Elizabeth Jones
Emily Jones
Eric Jones
Gareth Jones
Garett Jones
Gary Jones
Graeme Jones
Gregory Jones
Hazel Jones
Hugh Jones
J. Iwan Jones
Jame Jones
Janice Jones
Jay Jones
Jeremy Jones
Jessica Jones
Kim Jones
Lara Jones
Lesley Jones
Michael Jones
Nancy Jones
Neil Jones
Nicholas Jones
Nick D. Jones
Nina Jones
Oliver Jones

Patrik Jones
Paul Anthony Jones
Peter Jones
Rachael Jones
Richard Jones
Robert Jones
Rodney Jones
Roland Jones
Salene Jones
Steven Jones
Stuart Jones
Tamekia Jones
Theresa Jones
Thomas Jongens
Anan Jongkaewwattana
Somchai Jongwutiwes
Donatas Jonikaitis
Daisy Jonkers
Krishna Jonnalagadda
Teppei Jono
Nataša Jonoska
Ian Jonsen
Melissa Jonson-Reid
Colleen Jonsson
Jón Einar Jónsson
K. Jönsson
Helmut Jonuleit
Changhee Joo
Dong Jin Joo
Hwang-Soo Joo
Rocio Joo
Marie Joossens
Antoinette Jooste
Leo Joosten
Simone Joosten
Yupin Jopang
Chris Jordan
Craig Jordan
Helen Jordan
Irmgard Jordan
Mark Jordan
Philip Jordan
Rebecca Jordan
Robert Jordan
Sue Jordan
Cristina Jordán
Ekaterina Jordanova
Joerg Jores
Frederike Jörg
Jorge Jorge
Marco Jorge
Veronique Jorge
Christopher Jorgensen
James Jorgensen
Joan Jorgensen
Christoffer Jørgensen
Hanne Skou Jørgensen
Rikke Bramming Jørgensen
Silje Jørgensen
Lars Jorgensens
Vanda Jorgetti
Carolina Jorgez
Robert Jorissen
Patricia Jorquera
Rudolf Jörres
Jesus Jorrin
Joachim Jose
Matthew Jose
Sophie Jose
Alf Josefson
Elisabet Josefsson
Christine Josenhans
Eron Jr. Joseph
Jomon Joseph
K. Joseph
Leo Joseph
Lucy Joseph
Sujith Joseph
Suresh Joseph
Yeboah Joseph
Dvora Joseph Davey
Emily Josephs
David Josephy
Ameeta Joshi
Ashwini Joshi
Atul Joshi
Bharat Joshi
Deepika Joshi
Madhav Joshi
Manish Joshi
Neelendra Joshi
Pheroze Joshi
Sadhana Joshi
Satish Joshi
Shantaram Joshi
Suhasini Joshi
Sunil Joshi
Taruna Joshi
Kaumudi Joshipura
John Joska
Naomi Josman
Stephanie Jost

Holger Joswig
Ana Jotic
Jerwen Jou
M.J. Jou
Jacques Joubert
Vianney Jouhet
Pierre-Simon Jouk
Shoou-Jeng Joung
N. Jourde-Chiche
François Jouret
Fabrice Journe
Laurent Journot
Céline Jousse
Nicolás Jouve
Eric Jouvent
Philippe Jouvet
Miljana Jovandaric
Borko Jovanovic
Nikolina Jovanovic
Branko Jovcic
Mariona Jové
Richard Jovelin
J. Joven
Miguel Jover Cerdá
Sara Jover-Gil
Alan Jovic
Jorge Jovicich
Edward Joy
Domino Joyce
Robin Joyce
Martin Joyce-Brady
Michael Joyner
Damian Józefiak
Cynthia Ju
Dianwen Ju
Jyh-Cherng Ju
Yan-Ying Ju
Young Seok Ju
Yu-Ten Ju
Chun-Jung Juan
Joon Ching Juan
Yung-Shun Juan
Ana Juan-Garcia
Bryan Juarez
Marc Jubeau
Pavel Juda
Matt Judah
Walter Judd
Howard Judelson
Stefan Judex
Andrew Judge
Kevin Judge
Sarah Judge
Susan Judge
Richard Judson
Freimut Juengling
Alexander Jueterbock
Andre Juffer
Angela Juhasz
Csaba Juhasz
Gabor Juhasz
Ted Juhl
Talis Juhna
Laura Juignet
Jose Manuel Juiz
Takayuki Jujo
Chantal Julia
Jason Julian
Timothy Julian
Esther Julián
Stuginski-Barbosa Juliana
Claire Julian-Reynier
Douglas Julin
Ilkka Julkunen
Petro Julkunen
Thomas Julou
Elizabeth Juma
Catherine Jumarie
Donald Jump
Ari Jumpponen
Jae Yeoul Jun
JinWoo Jun
Liu Jun
Howard Junca
Romina Juncos
Hugo Júnez-Ferreira
Andreas Jung
Byung Hwa Jung
Corinne Jung
Friedrich Jung
Ho Won Jung
Il Lae Jung
Jae Won Jung
Ji Ye Jung
Jin Tae Jung
Joohee Jung
Jung Jung
Ki Won Jung
Kirsten Jung
Kwang-Woo Jung
Kyu Sik Jung
Manfred Jung

Paul Jung
Ranju Jung
Seunho Jung
Sukgeun Jung
Sunghwan Jung
Wi Hoon Jung
Won Hee Jung
Won-Kyo Jung
Woo-Sung Jung
Yong Tae Jung
Young Hoon Jung
Alois Jungbauer
Sean Jungbluth
Claudia Junge
Christian Junghanss
Sandra Junglen
Tanja Jungmann
Berit Jungnickel
Pilar Junier
Robert Junker
Guillaume Junqua
Ana Paula Junqueira-Kipnis
Stacy Jupiter
Bianca Jupp
Petr Juracka
Georgiana Juravle
Michael Jurczak
Jon Jureidini
Russell Jurenka
Arnis Jurevics
Joanna Jurewicz
Diana Juriloff
Piotr Jurkiewicz
Janine Jurkowski
Suzanne Jurriaans
Todd Jusko
William Jusko
Lothar Just
Eric Justes
Sheryl Justice
Jean-Lou Justine
Jessica Justman
Giselle Justo
Julie Justo
Rosicka-Kaczmarek Justyna
Marko Jusup
Antarpreet Jutla
Didier Jutras-Aswad
Catherine Jutzeler
Jukka Juutilainen
Judith Juvancic-Heltzel
Praveen Juvvadi
Garima Juyal
Petras Juzenas
