# Supplementary material for: PLOS ONE 2016 Reviewer and Editorial Board Thank You
Source: PLoS One. 2017 Mar 20;12(3):e0174259. doi: 10.1371/journal.pone.0174259 (PMC5358840; doi:10.1371/journal.pone.0174259)
Supplement: S3 Reviewer List — (PDF) [file pone.0174259.s004.PDF]

PLOS ONE would like to thank all those who reviewed on behalf of the journal in 2016:

Gopal K.
Hakhyun Ka
Anne Marije Kaag
Anu Kaakinen
Johannes Kaanders
Tommi Kääriäinen
Achim Kaasch
Valtteri Kaasinen
Anna Kaatz
Martin Kaatz
David Kaawa-Mafigiri
Conrad Kabali
Tamon Kabata
David Kabelik
Parijat Kabiraj
Nadja Kabisch
Sandor Kabos
Gilberto Kac
Shannon Kachel
Markus Kächele
Sumita Kachhwaha
Amir Kachooei
Priyanka Kachroo
Tim Kacprowski
Petra Kaczensky
Andreas Kaczmarczyk
Leonard K. Kaczmarek
Lukasz Kaczmarek
Monika Kaczmarek
Sandeep Kadam
Assaf Kadar
John Kaddis
Hussein Kaddour
Mark Kaddumukasa
Rajendra Kadel
Fazlul Kader
Paul Kadetz
Mohannad Kadhum
Pinar Kadiroglu
Krishna Kadirvelu
Mikiko Kadohisa
James Kadonaga
Takafumi Kadono
Narendra Kadoo
Mariko Kadota
Mitsutaka Kadota
Komei Kadowaki
Norimitsu Kadowaki
Giouliana Kadra
Milosz Kadzinski
Igor Kaefer
Lutz Kaelber
Shawn Kaeppler
Sven Kaese
Michael Kaess
Mirjam Kaestli
Ivo Kaethner
David Kaetzel
Jaranit Kaewkungwal
Wafaa Kaf
Tina Kaffenberger
Uzi Kafkafi
Bjorn Kafsack
Jonathan Kagan
Masaharu Kagawa
Azusa Kage
Ashraf Kagee
Shinichi Kageyama
Lucila Kagohara Elias
Hassan Kahal
Daniel Kahan
Alon Kahana
Chaim Kahana
Eva Kahana
Shoshana Kahana
Gila Kahila Bar-Gal
Barbara Kahl
J. Michelle Kahlenberg
Charlene Kahler
Ulf Kahlert
Sonja Kahlmeier
Andrew Kahn
C. Ronald Kahn
James Kahn
Matthew Kahn
Shulamit Kahn
Lyn Sue Kahng
Danny Ka-Ho Wong
Alem Kahsai
Hirofumi Kai

Hisashi Kai
Angela Kaida
Toshimi Kaido
Georgia Kaidonis
Darnell Kaigler
Ishpinder Kailey
Kevin Kain
B. Kaina
Bernd Kaina
Hans Kainz
Martin Kainz
Philipp Kainz
Benny Kaipparettu
Armin Kaiser
Daniel Kaiser
Jerome Kaiser
Jochen Kaiser
Kathryn Kaiser
Lucia Kaiser
Maria Kaiser
Peter Kaiser
Raphaela Kaisler
Pekka Kaitaniemi
Eloise Kaizar
Murali Kaja
Olli Kajander
Andrey Kajava
Aiko Kaji
Hideko Kaji
Hiroshi Kaji
Izumi Kaji
Kazuaki Kajimoto
Yukie Kajita
Mikihito Kajiya
Robert Kajobe
Adriana Kajon
Lukasz Kajtoch
Evdoxia Kakani
Sham Kakar
Kazuhiro Kakimi
Yoshihiko Kakinuma
Masako Kakizaki
Mayumi Kako
Masao Kakoki
Shaka Kaku
Ritsuko Kakuma
Madhavi Kakumanu
Hiroki Kakuta
Yoshimitsu Kakuta
Chandra Prakash Kala
Tamas Kalai
Evangelos Kalaitzakis
Amanda Kalamar
Theodoros Kalampokas
Spyros Kalams
Mohammad Ali Kalantar Motamedi
Mohsen Kalantari
Sundeep Kalantry
Raj Kalaria
Siripen Kalayanarooj
Sita Kalayanarooj
Miriam Kalbitz
Matina Kalcounis-Rueppell
Lee Kalcsits
Sandip Kale
Amy Kaleita
Fatch Kalembo
Smilja Kalenic
Dimitri Kalenitchenko
Tobias Kalenscher
Gonul Kaletunc
Sameer Kalghatgi
Satish Kalhan
Amy Kalia
Manjula Kalia
Sufiyan Kalikan
Kyriaki Kalimeri
Hannu Kalimo
Shirin Kalimuddin
Pawel Kalinski
Karen Kalinyak
Jennifer Kalish
Marcia Kalish
Katarzyna Kalita
Grigorios Kalivas
Peter Kalivas
Thangavelu Kaliyappan
Susana Kalko
Dheeraj Kalladka
Bhaskar Kallakury
Avyakta Kallam
Michael Kallen
Michael Kaller
Kimberly Kalli
Asha Kallianpur
A.S. Kallimanis
Athanasios S. Kallimanis
Tuomo Kalliokoski
Maija-Liisa Kalliomäki
Tuula Kallunki
Eric R. Kallwitz
I.D. Kalmar

Jayne Kalmar
Uldis Kalnenieks
Emily Kalnicky
Zoltan Kalo
Eleftheria Kalogera
Stefanos Kalogirou
Ameeta Kalokhe
Lukáš Kalous
Oliver Kalpak
Evanthia Kalpazidou Schmidt
Bhawna Kalra
Pramila Kalra
Sanjay Kalra
Alon Kalron
Sukhvinder Kalsi-Ryan
Anna Kaltenboeck
Guruprasad Kalthur
Heino R. Kaltiala
Nishan Kalupahana
Peter Kalvass
Ramki Kalyanaraman
Ramaswamy Kalyanasundaram
Joan Kalyango
Robert Kalyesubula
Umut Kalyoncu
Marco Kalz
Jason Kam
Tae-In Kam
Yusuke Kamachi
Kyousuke Kamada
Masamitsu Kamada
Nobuhiko Kamada
A. Kamal
Ritul Kamal
Manoj Kamalanathan
Fahimeh Kamali
Balu Kamaraj
Thomas Kamarck
Venu Kamarthapu
Rujvi Kamat
Hideaki Kamata
Veena Kamath
Beena Kamath-Rayne
Edwin Kamau
Mary Kamb
Avinash Kambadakone
Rukundo Kambarami
Devaiah Kambiranda
Pravin Kamble
Mini Kamboj
Sumalee Kamchonwongpaisan
Hideto Kameda
Jun Kamei
Mohamed Gomaa Kamel
Mohammad Kamely
Alexey Kamenskiy
Takaharu Kameoka
Maarten Kamermans
Masaki Kameyama
Hidehiro Kamezaki
Kei Kamide
Naoto Kamide
Taro Kamigaki
Keita Kamijo
Astuko Kamijo-Ikemori
Shinji Kamimura
Alexandra Kamins
Bozena Kaminska
Karol Kaminski
Susan Kaminskyj
Toshinori Kamisako
Kubra Kamisoglu
Akihide Kamiya
Takehiro Kamiya
Yusuke Kamiya
Yuusuke Kamiyoshihara
Madhu Kamle
Jan Kamler
Meena Kamlesh
Christina Kamma-Lorger
Thomas Kammer
Christian Kammerer
Jody Kamminga
Masashi Kamo
Marcel Kamp
Olle Kämpe
Gueneter Kampf
Thomas Kampf
Carlijn Kamphuis
George Kampis
Ulla Kampmann
Jan. A.A.M. Kamps
Thomas Kamradt
Ruth Kamrowski
Collins Kamunde
Daniel Kamykowski
Chi-Chuan Kan
Kees-Jan Kan
Tao Kan
Bavesh Kana
Motoi Kanagawa
Nobuhisa Kanahara

M. Kanai
Ioannis Kanakis
M. Kanakis
Jill Kanaley
Keiko Kanamori
Keizo Kanasaki
Rushed Kanawati
Gen Kanaya
Gen Kanayama
Motoyori Kanazawa
Rajashekhar Kanchanapally
Vijaya Kancherla
Tatsuo Kanda
Teru Kanda
Tomonori Kanda
Yoshinobu Kanda
Matheswaran Kandasamy
Yoga Kandasamy
Abraham Kandathil
Eugene Kandel
Rita Kandel
Sunil Kandel
Farid Kandil
Raju Kandimalla
Ramesh Kandimalla
Anne Kandler
Akihiko Kandori
Eeshani Kandpal
Adam Kane
Daniel Kane
Jennifer Kane
Jeremy Kane
Joanne Kane
Olivia Kane
Robert Kane
Sarah Kane
Yasuhiro Kaneda
Kazue Kanehara
Akira Kaneko
Shuji Kaneko
Yoshikatsu Kaneko
Yuji Kaneko
Yuko Kaneko
Angelos Kanellis
Kenneth Kaneshiro
Bum-Yong Kang
Byoung-Cheorl Kang
Byung-Ho Kang
Changwon Kang
ChulHee Kang
Chunying Kang
CongBao Kang
Dae-Kyung Kang
Dongchon Kang
Gagandeep Kang
Gyeong Hoon Kang
Hakmook Kang
Hojeong Kang
Hwan-Goo Kang
Hyunseung Kang
Insoo Kang
Jian Kang
Jihee Lee Kang
Jing X. Kang
Jin-Ho Kang
Jong-Sun Kang
Kai Kang
Keon Wook Kang
Kristopher Kang
Kyu-Suk Kang
Lin Kang
Min Kang
Minchul Kang
Minglei Kang
Pauline Kang
Sanghee Kang
Sang-Hee Kang
Sanghoon Kang
Se Woong Kang
Seungbum Kang
Seungha Kang
Sun Kang
Sung Wook Kang
Wanmo Kang
Wonku Kang
Wonseok Kang
Xiang Kang
Yanyong Kang
Yuan Yuan Kang
Yubin Kang
Yuejun Kang
Yun Kang
Yunhee Kang
Zhensheng Kang
Artur Kania
Gabriela Kania
Stephen Kania
Urša Kanjir
Phyllis Kanki
Yasuharu Kanki
Georgette Kanmogne
Oliver Kann

Meganathan Kannan
Nagarajan Kannan
Ram Kannan
Srinivasaraghavan Kannan
T.R. Kannan
Ramaswamy Kannappan
Stephen Kanne
Kasturi Kanniah
Juho Kanniainen
Yoshihiko Kannno
Sridhar Kannurpatti
Flora Kano
Fumihiro Kano
Yasunori Kano
Michael Kanost
Anuraag Kansal
Sandip Kanse
Alpdogan Kantarci
Anu Kantele
Julie Kanter
Thomas Kantermann
Steve Kanters
Sree Kanthaswamy
Jimut Kanti Ghosh
John Kantner
Tatsuya Kanto
Shawn Kantor
Yashpal Kanwar
Mufaro Kanyangarara
Go Kanzaki
Hiroyuki Kanzaki
C. Cheng Kao
Cheng-Fu Kao
Hao-Yun Kao
Jill Kao
Peter Kao
Rowland Kao
T.C. Kao
Tze-Wah Kao
Wei-Yu Kao
Wen-Yuan Kao
Winston Kao
Yi-Hsuan Kao
Suray Kapa
Bhaveen Kapadia
Dharmi Kapadia
Bryan Kapella
Adam Kapelner
Maria Kapetanaki
Panagiotis Kapetas
Vikram Kapila
Michael Kapiloff
Daniel Kaplan
David Kaplan
Gilaad Kaplan
Isaac Kaplan
Mark Kaplan
Ozgur Kaplan
Robert C Kaplan
Warren Kaplan
Socrates Kaplanis
Atya Kapley
Nicholas Kaplinsky
Kevin Kaplowitz
Anil Kapoor
Rupa Kapoor
Vaishali Kapoor
Andras Kaposi
Vijaya Kappala
L.J. Kappelle
János Kappelmayer.
Charles Kappenstein
Astrid Kappers
Mattes Kappert
Matt Kappes
Efstathia Kapsogeorgou
Ted Kaptchuk
Stephen Kaptoge
Martin Kapun
Gaurav Kapur
Navin Kapur
Reuben Kapur
Aurélie Kapusta
Nestor Kapusta
Pawel Kapusta
Sandip Kar
Firat Kara
Murat Karabatak
Turan Karaca
Bilge Karacali
Niki Karachaliou
Spyros Karadimas
Levente Karaffa
Apostolos Karagiannis
Jim Karagiannis
Tom Karagiannis
Julia Karagicheva
Domna Karagogeos
Nabil Karah
Mahir Karakas
Ioannis Karakikes
Giorgos Karakousis

Gokhan Karakulah
Firdes Saadet Karakulak
Ahmed Karam
Azza Karam
Yannis Karamanos
Rallis Karamichalis
Sofia Karamintziou
Panagiotis Karampourniotis
Belgin Karan
Ratna Karan
Victor Karandashev
Paul Karanicolas
Styliani Karanika
Marina Karanikolos
Panagiotis Karanis
Ivana Karanovic
Santhosh Karanth
Christos Karapiperis
Georgios Kararigas
Alicia Karas
Lana Karasik
William Karasov
Ece Karatan
Ilia Karatsoreos
Georgios Karavas
Kamilu Karaye
Hamid Reza Karbalaei-Heidari
Musturay Karcaaltincaba
Celeste Karch
François Karch
Suzan Kardong-Edgren
Marty Kardos
Joseph Kardouni
Isabella Kardys
Hassen Kared
David Kareken
Stephen Karesh
Rolf Karez
Mehdi Kargar
Fatemeh Kargarfard
Axel Karger
Andrei Karginov
Joanna Kargul
John Karijolich
Rashed Karim
Zubair Karim
Ehsan Karimi
Hamid Reza Karimi
Mahdi Karimi
Mansour Karimi
Parisa Karimi
Morteza Karimipoor
Anastasia Karioti
Samuel Kariuki
Symon Kariuki
Rupesh Kariyat
Juha Karjalainen
Abhilasha Karkey
Subhas Karki
Antti Karkman
Anna Kärkönen
Heidrun Karlic
Apostolos Karligkiotis
Joel Karliner
Kinga Karlinger
Eric Karlins
Gennady Karlov
Trine Karlsen
Brynjar Karlsson
Erik Karlsson
Fredrik Karlsson
Laila Karlsson
Oskar Karlsson
Marcus Karlstetter
Rolf Karlstrom
Faisal Karmali
Amol Karmarkar
Wilfried Karmaus
Konstantinos Karmiris
Christof Karmonik
Dimitrios Karmpaliotis
Tuomas Karna
Anupama Karnam
Robert J. Karnes
Sasha Karnes
Daniel Karney
Rahul Karnik
A.E. Karnoub
Nina Karnovsky
Piotr Karolewski
Dwi Setyowati Karolina
György Károlyi
Nitsara Karoonuthaisiri
Evgenia Karousou
Jordan Karp
Sarah Karpanty
Adam Karpf
Andrew Karplus
P. Karplus
Dimitrios Karpouzas
Aisté Karpušenkaité
Nancy Karraker

Locke Karriker
Maile Karris
Istvan Karsai
Marton Karsai
Stephanie Karst
Sven Karstens
Balasubramanian Karthick
Mikko Karttunen
Tyler Kartzinel
S. Karumanchi
Sheela Karunanithi
Indrani Karunasagar
Senthilkumar Karuppagounder
Jaishankar Karuppannan
Shunmugiah Karutha Pandian
Shilpa Karvande
Jacek Karwowski
Maciek Karwowski
Kenny Karyadi
Anastasios Karydis
Hideko Kasahara
Kota Kasahara
Kiyoto Kasai
Parastu Kasaie
Tsuyoshi Kasama
Devan Kasangara
Annemarie Käsbohrer
Daniel Kaschek
Elena Kaschina
Kristin Kaschner
Ahmed Kaseb
Sabine Kasel
Moses Kasembeli
Benjamin Kasenda
Thomas Kash
David Kashatus
Nasim Kashef
Hideaki Kashima
Shinichiro Kashiwagi
Ali Kashkouli
Rajpal Kashyap
Anna Kasielska-Trojan
Ramanathan Kasimanickam
Sabine Kasimir-Bauer
Balakuntalam Kasinath
Poothapillai Kasinathan
Andrea Kasinski
Konstantinos Kasiotis
Yakup Kaska
Frederick Kaskel
Manish Kasliwal
Enkelejda Kasneci
Bernd Kasper
Dennis Kasper
Erich Kasper
Fred Kasper
Michael Kasper
Susan Kasper
Gregor Kasprian
Miroslaw Kasprzak
Daniel Kass
Robert Kass
Mulualem Kassa
Amir Kassam
Charles Kassardjian
Victor Kassey
Alexei Kassian
Stavros Kassinos
Radwan Kassir
Zamaneh Kassiri
Mika Kastarinen
Mary Jo Kasten
Gerald Kasting
Irida Kastrati
Panagiotis Kastritis
Jens Kastrup
Sudhir Kasturi
Jun Kasuga
Shoko Kasuga
Michael Kasumovic
Beata Kasztelan-Szczerbinska
Anna Kata
Takenobu Katagiri
Toyomasa Katagiri
Prasad Katakam
Yoshinori Katakura
Ramesh Katam
Martijn Katan
Hiroyuki Katano
Toshiya Katano
H. Kataoka
Ranjit Kataria
Naoki Katase
Kan Katayama
Tsutomu Katayama
Lorig Kate
Rohit Kate
Fredrick Kateera
Koutra Katerina
Wendy Kates
Pantelis Katharios
Luciana Katiki

Kishore Katikireddy
L. Katirayi
Akihiko Kato
Fumi Kato
Gregory Kato
Hisanori Kato
Ikuma Kato
Juntaro Kato
Kazuhisa Kato
Kikuya Kato
Koichi Kato
Makoto Kato
Masaru Kato
Massuo Kato
Mitsuo Kato
Naoki Kato
Nobuhiro Kato
Noriko Kato
Ryuji Kato
Satoshi Kato
Shigeaki Kato
Takamitsu Kato
Takehide Kato
Yasumasa Kato
Kiran Katoch
Akira Katoh
Hironori Katoh
Kazutaka Katoh
Ioannis Katramados
Guy Katriel
Lee Kats
Emmanuel Katsanis
Konstantinos Katsanos
Olga Katsarou
Pavel Katsel
Panagiotis Katsonis
Konstantinos Katsoulis
Antonios Katsounas
Sugie Katsuji
Toshio Katsukawa
Hiroshi Katsuki
Yukako Katsura
Shutaro Katsurabayashi
Tomohiro Katsuya
Ramesh Katta
Daniel Kattnig
Karen Katula
Abram Katz
David Katz
Joanne Katz
Keziah Katz
Lee Katz
Lisa Katz
Matthew Katz
Richard Katz
Sharyn Katz
William Katz
Hans Katzberg
Mary Katzenberg
Marco Katzenberger
David Katzenstein
Helmut Katzgraber
Gadi Katzir
Philip Katzman
Wendy Katzman
Hannah Katznelson
Laurence Katznelson
Todd Katzner
Benedikt Kaufer
Norbert F. Käufer
Mathias Kauff
Gilles Kauffenstein
Louis Kauffman
Elizabeth Kaufman
Jason Kaufman
Jim Kaufman
Laura Kaufman
Lloyd Kaufman
Miriam Kaufman
Pamela Kaufman
Erica Kaufman West
Christopher Kaufmann
Rainer Kaufmann
Abhishek Kaul
Rashmi Kaul
Sanjana Kaul
Barbara Kaup
Anu Kauppinen
Gunveen Kaur
Gurcharan Kaur
Indu Pal Kaur
Jasbir Kaur
Jaspreet Kaur
Kamaljit Kaur
Rupinderjeet Kaur
Sukhbir Kaur
Sukhwinder Kaur
Antti Kause
Gagan Kaushal
Gur Kaushal
Manoj Kaushal
Navin Kaushal

Charu Kaushic
Azad Kaushik
Sadasivam Kaushik
Markus Kautz
John Kavanagh
Justin Kavanagh
Phillip Kavanagh
Musa Kavas
Gitit Kave
P. Kavi Kishor
Amol Kavishwar
Zahra Kavosi
Panos Kavvadas
Hiroyoshi Kawaai
Saneyuki Kawabata
Shun-ichiro Kawabata
Y. Kawabata
Kentaro Kawabe
Soichiro Kawabe
Yousuke Kawachi
Norifumi Kawada
Yasushi Kawaguchi
Genri Kawahara
Manabu Kawahara
Tetsuya Kawahara
Yutaka Kawahito
Gota Kawai
Kenichiro Kawai
Masanobu Kawai
Motofumi Kawai
Koji Kawakami
Toshiaki Kawakami
Yasuhiko Kawakami
Yutaka Kawakami
Tetsuya Kawakita
Agata Kawalec
Shweta Bagewadi Kawalia
Hiroaki Kawamichi
Elisa Kawamoto
Ryuichi Kawamoto
Shinya Kawamoto
Akane Kawamura
Kazuhiro Kawamura
Kouichi Kawamura
Hideki Kawanishi
Kenji Kawano
Ryo Kawasaki
Tomoyuki Kawase
Koichiro Kawashima
Masahiro Kawashima
Motoko Kawashima
Suguru Kawato
Satoko Kawauchi
Takeshi Kawauchi
Thomas Kawula
Alan Kay
Anthony Kay
Mathew Kay
Robert Kay
Ali Kaya
Yalcin Kaya
Ziya Kaya
Huseyin Kayadibi
Mohsen Kayal
Ghazi Kayali
Kou Kayamori
Elizabeth Kaye
Thomas Kaye
Patrick Kayembe
Kassoum Kayentao
Herbert Kayiga
James Kayima
David Kaylie
Harvey Kayman
George Kaysen
Bengt Kayser
Gian Kayser
Leonard Kaysser
Reem Kayyali
Lawrence Kazak
Hokto Kazama
Junichiro Kazama
Kemal Kazan
Rumeyza Kazancioglu
Ilias Kazanis
Zeinab Kazemi
Yukinori Kazeto
Andrius Kazlauskas
Victoria Kazmerski
Nabila Kazmi
Clarice Kazue Fujihara
Toshinobu Kazui
Santosh KC
Hengning Ke
Jiyuan Ke
Po-Ju Ke
Ruian Ke
Ruimin Ke
Wenshan Ke
Yang Ke
Yi-Ni Ke
Sarah Keadle

Paul Keall
Jacob Kean
Brian Keane
Joseph Keane
Graham Kearn
David Kearney
Jennifer Kearney
Mary Kearney
Robert Kearney
Russell Keast
Rebekah Keating
Sheila Keating
Shelley Keating
Emily Keats
Lisa Keay
David Keays
Bereket Kebede
Melkam Kebede
Moritz Kebschull
Defne Kecik
Noemi Kedei
Nancy Kedersha
Sasko Kedev
Haji Kedir
Ureporn Kedjarune-Leggat
Ross Kedl
Kwong-Ming Kee
Younghoon Kee
Edward Keedwell
Lucas Keefer
Kevin Keegan
Kim Kee-Hong
Christoph Keel
Brandon Keele
Annika Keeley
Jon Keeley
Thomas Keeley
Linda Keeling
Stephen Keely
Olya Keen
Phillip Keen
Melody Keena
Daniel Keenan
Jacqueline Keenan
Katherine Keenan
Tiarnan Keenan
Douglas Keene
Malcolm Keeping
Richard Keers
Daniel Keeser
Mark Keezer
Sarah Kefayati
Angie Kehagia
Alexander Kehl
Thomas Kehl-Fie
Corinna Kehrenberg
Uri Keich
Bart Keijser
Deborah Keil
Guenther Keil
Kimberly Keil
Thomas Keil
Kenneth Keiler
Ashley Keiser
Olivia Keiser
Julie Keister
Lisa Keister
Akilah Keita
Douglas Keith
Jonathan Keith
Scott Keith
Anouk Keizer
Peter Kekenes-Huskey
Mari Kekkonen
Ramesh Kekunnaya
Brendan Kelaher
Uddhav Kelavkar
Saskia Kelders
Henrich Kele
Theodoros Kelesidis
Roya Kelishadi
Barbara Kellam
Sebastian Kelle
Colin Kelleher
Kelly Kelleher
Alexandre Keller
Amy Keller
Barbara Keller
Bettina Keller
Evan Keller
Kate Keller
Klaus Keller
Kristin Keller
Maria Keller
Matthieu Keller
Roberta L. Keller
Simona Keller
Susanna Keller
Troy Keller
Ulrich Keller
Walter Keller
Maureen Keller-Wood

Jeff Kelleway
Colleen Kelley
Darshan Kelley
David Kelley
Eric Kelley
George Kelley
Joanna Kelley
Mark Kelley
Matthew Kelley
Stephen Kelley
Stuart Kellie
Christian Kellner
Kenneth Kellner
Michael Kellner
Joshua Kellogg
John Kellum
Anita Kelly
Brian Kelly
Charles Kelly
Christine Kelly
Clint Kelly
Daniel Kelly
Dominic Kelly
Emer Kelly
J. Kelly
James D. Kelly
Jeremiah Kelly
John Kelly
Krista Kelly
Pat Kelly
Paul Kelly
Robert Kelly
Ryan Kelly
Steven Kelly
Malte Kelm
Karl Kelsey
Katharine Kelsey
J.A. Scott Kelso
Terhi Keltanen
David Kelton
Damian Kelty-Stephen
George Kemenes
Lajos Kemeny
Martin Kemler
Markus Kemmelmeier
Andre Kemmling
Joseph Kemnitz
Darrell Kemp
Elizabeth Kemp
G. Kemp
John Kemp
Kevin Kemp
Björn Kemper
Hervé Kempf
Marie Kempf
Stefan Kempf
Tibor Kempf
Robert Kemppainen
Vivek Kempraj
Wolfe Ken
Shifra Ken Dror
Eben Kenah
Christopher Kenaley
Carl Kendall
Matt Kendall
William Kendall
Pinar Kendigelen
Tetyana Kendzerska
István Kenessey
Dror Kenett
Yoed Kenett
Shian-Ling Keng
Andre Kengne
Paul-Ariel Kenigsberg
Andrew Keniry
Marc Kenis
Richard Kennaway
Ashley Kennedy
Breandán Kennedy
Brian Kennedy
Briana Kennedy
Cassie Kennedy
Chinaro Kennedy
David Kennedy
Emma Kennedy
Lindsey Kennedy
Lorna Kennedy
Malcolm Kennedy
Michael Kennedy
Nicholas Kennedy
Sheela Kennedy
Tay Kennedy
William Kennedy
Pierre Kennepohl
John Kenneth
Erica Kenney
Joanie Kenney
Larry Kenney
M. Kenney
Scott Kenney
William Kenney
Jason Kennington

David Kenny
Glen Kenny
Rose Anne Kenny
Tsuyoshi Kenri
Paul Kenrick
Ronald Kensinger
Angius Kent
Christopher Kent
David Kent
Erin Kent
L. Kent
Michael Kent
Shia Kent
Tara Kent
William Kent
Alexander Kentikelenis
Amanda Kentner
Alex Kentsis
Tuomas Kentta
Ben Kenward
Nicholas Kenyon
Justin Keogh
Sven Kepes
George Kephart
Olga Kepinska
Thomas Kepler
Gunnar Keppel
Brian Keppler
Daniel Keppler
Georgia Keramida
Roxanne Kerani
Robert Kerbel
Stefan Kerber
Robert Kerby
Garry Kerch
Carole Kerdelhue
Saadia Kerdine-Romer
Halima Kerdjoudj
John Kerekes
Nir Keren
Gael Kergoat
Micheline Kergoat
Hugo Kerhervé
Ruth Keri
Sam Kerk
Annette Kerkhoff
Johan Kerkhofs
Jens Kerl
Ali Kermanizadeh
Stephanie Kermorgant
Florian Kern
Peggy Kern
Petra Kern
Timothy S Kern
Winfried Kern
Walter Kernan
Berit Kerner
Steven Kernie
J. Kero
Andrew Kerr
Bethany Kerr
Ian Kerr
Peter Kerr
Rachel Kerr
Ian Kerridge
Steven Kerrigan
Julia Kerschbaum
Erin Kershaw
Arik Kershenbaum
Alan Kersten
Phil Kersten
Sander Kersten
Anette Kersting
Uwe Kersting
Wilhelmina Kerstjens-Frederikse
Janos Kertesz
Attila Kertesz-Farkas
Aparna Kesarwala
Lakshmyya Kesavalu
James Kesby
Ali Keshavarzian
Shashank Keshavmurthy
Abbas Keshtkar
Mahtab Keshvari
Dilek Keskin
Emre Keskin
Stefan Keslacy
Shelli Kesler
Recep Kesli
Can Kesmir
Sarah Kessans
David Kessel
Dominique Kessel
Line Kessel
Benedikt Kessler
Danny Kessler
Felix Kessler
Ian Kessler
Michael Kessler
Thomas Kessler
V.G. Kessler
Yoav Kessler

Thomas Kesteman
Luc Kestens
Martin Keszler
Kirsi Ketola
Markus Ketteler
Johannes Kettunen
James Ketudat Cairns
Young-Sam Keum
Philipp Keune
Els Keunen
Michaela Keuper
Helmut Keupp
Zela Keuylian
Peter Kevan
Christopher Kevil
Felix Key
Nigel Key
Anthony Keyburn
Nemat Keyhani
Yoav Keynan
Khandan Keyomarsi
Ulrich Keyser
M.A. Keyzer
Dineo Khabele
Jyoti Khadka
Maksim Khadkevich
Moncef Khairallah
Md. Zain Khaironizam
Alka Khaitan
Subhas Khajanchi
Razieh Khajehkazemi
Salim Khakoo
Farid Khalafalla
Annette Khaled
Olfa Khalfallah
Syma Khalid
Michael Khalil
Mohd Ibrahim Khalil
Mostafa Khalil
Davood Khalili
Hamed Khalili
Kamel Khalili
Amir Khaliq
J. Khalsa
Bavornlak Khamnamtong
Abdul Khan
Abdur Khan
Ajmal Khan
Ali Khan
Amir Khan
Arif Khan
Arshad Khan
Asis Khan
Faisal Khan
Hafiz Khan
Haseeb Khan
Ilyas Khan
Imdad Khan
Imran Khan
Izhar Khan
Khalid Khan
M. Asim Khan
Matiullah Khan
Md Gulam Khan
Meraj Khan
Mobarak Khan
Mohsin Khan
Muhammad Altaf Khan
Mushfiquddin Khan
Nadeem Khan
Naveed Khan
Nilofar Khan
Nooruddin Khan
R.J. Khan
Rashid Khan
Razib Khan
Rizwan Khan
Sameer Khan
Sardar Khan
Seema Khan
Shahid Khan
Sheema Khan
Suleman Khan
Tahir Khan
Umar Khan
Waqas Khan
Wasif Khan
Wasim Khan
Wazir Zada Khan
Yusuf Khan
Rasheda Khanam
Hashem Khanbabaei
Lidita Khandeparker
Ashutosh Khandha
Ashish Khanna
Ashwani Khanna
Sahil Khanna
Savita Khanna
Tarun Khanna
Rizwana Khanum
Neelam Khaper
Michael Kharas

Jenan Kharbush
Sharad Khare
Ritu Kharer
Akram Kharroubi
Samer Kharroubi
Vyacheslav Kharuk
Ali Khashan
Khaled Khatab
Camilo Khatchikian
Hasan Khatib
Mehar Khatkar
Hafeeza Khatoon
Bhavin Khatri
Resham Khatri
Vishal Khatri
Yogan Khatri
Sunil Khattar
Anthony Khawaja
Mourad Khayati
Meleckidzedeck Khayesi
Y. Khaykin
Yasser Khazaal
Maryam Khazaee-Pool
Habib Khazaie
Mazen Kheirbek
Valid Kheirollahi
Robinder Khemani
Ajay Kher
Amit Khera
Reetika Khera
Amir Kheradmand
Saeed Reza Kheradpisheh
Salman Khetani
Sumeet Khetarpal
C.L. Khetrapal
Vitaly Kheyfets
Hossein Khiabanian
Aurelie Khimoun
Michelle Kho
Fariba Khodagholi
Ahmad Khodayari Rostamabad
Anwar Khoirul
Samannaaz Khoja
Bee Ee Khoo
Edmund Khoo
Joan Khoo
Lip Khoon Kho
Alok Khorana
Yasamin Khorramzadeh
Sabra Khorsand Ahmadi
Alexander Khoruts
Celso Khosa
Habibeh Khoshbouei
Sohail Khoshnevis
Samaneh Khoshrou
Nidhi Khosla
Ali Reza Khosravi
Pegah Khosravi
Christine Khosropour
David Khoury
Noura Khoury
Thaer Khoury
Konstantin Khrapko
Andrei Khrennikov
Sadik Khuder
Nonhlanhla Khumalo
Surapan Khunamornpong
Syed Khundmiri
Navadon Khunlertgit
Dhanachandra Khuraijam
Ashwani Khurana
Satish Khurana
Tejvir Khurana
Anzar Khuroo
Kiran Khush
Sieu Khuu
Arif Khwaja
Jong Ki
Sung Hwan Ki
Jean-Fu Kiang
Richard Kiang
Mohammad Kiani
Nima Kianoush
Suda Kiatkamjornwong
Melina Kibbe
Frederick Kibenge
Alex Kibitov
Muhammad Kibriya
Emre Kiciman
Philipp Kickingereder
Kozui Kida
Kenneth Kidd
Linda Kidd
Martin Kidd
Thomas Kidd
Steven Kiddle
Rachel Kidman
Yasutoshi Kido
Thomas Kieber-Emmons
Ursula Kiechl-Kohlendorfer
Christiane Kiefer
Florian Kiefer

Todd Kiefer
Pawel Kiela
Jennifer Kielczewski
I.N. Kieler
Jan Kielstein
Patrick Kiely
Lambertus Kiemeney
Petra Kienesberger
Ellen Kienzle
Michael Kienzler
Ann Kier
Justyna Kierat
Amy Kiernan
Jeffrey Kiernan
Bernd Kieseier
Aaron Kiess
Rogier Kievit
Peter Kiffney
Godfrey Kigozi
N.G. Kigozi
Juliet Kiguli
Takanori Kihara
John Kihlstrom
Lars Kihm
Gustavo Kijak
Gregor Kijanka
Takahiro Kikawada
Don Kikkawa
Lisette Kikkert
Rainer Kiko
Colin Kikuchi
Eiji Kikuchi
Haruhito Kikuchi
Jun Kikuchi
Mitsuru Kikuchi
Naoki Kikuchi
Ryosuke Kikuchi
Taisei Kikuchi
Yoshitomo Kikuchi
Takashi Kikukawa
Takefumi Kikusui
Mariana Kikuti
Hiroe Kikuzaki
Raouf Kilada
Sreenivasulu Kilari
Angela Kilb
John Kilbane
Cherry Kilbride
Kelly Kilburn
Liam Kilduff
Sean Kiley
Alixe Kilgour
Stephen Kilianski
Ayse Kilic
Mustafa Kilickap
Anthony Killard
Shaun Killen
Kristina Killgrove
Timothy Killingback
Nabil Killiny
Elizabeth Killion
Rebecca Kilner
A. Kilpatrick
Michelle Kilpatrick
Charlotte Kilstrup-Nielsen
Konstantina Kilteni
Anne Kiltie
Uta Kiltz
Anthony Kim
Beom Kyung Kim
Beomsoo Kim
Bo Hyun Kim
Bong-Wan Kim
Brian Kim
Bum Jung Kim
Byeong Kim
Byoungsoo Kim
Byung-Seok Kim
Changhoon Kim
Changsun Kim
Cheol Sang Kim
Cheol Soo Kim
Christopher Kim
Dae Woo Kim
Daehyun Kim
Daejin Kim
Daniel Kim
Deok Ryun Kim
Do-Geun Kim
Do-Gyoon Kim
Dokyoon Kim
Dong Joon Kim
Dong Wook Kim
Donghee Kim
Dong-Hyun Kim
Dong-Joo Kim
Dong-Jun Kim
Dong-Ki Kim
Dong-Yun Kim
Ella Kim
Eui-Jin Kim
Eun Ran Kim

Eunjoo Kim
Eun-Kyoung Kim
Francis Kim
Gheun-Ho Kim
H. Nina Kim
Ha Won Kim
Hae Won Kim
Hak-Jae Kim
Hang-Rae Kim
Hangun Kim
Hankyul Kim
Hee Jin Kim
Hee Taek Kim
Heebal Kim
Hee-Jin Kim
Heejong Kim
Hei Sung Kim
Heui-Soo Kim
Hocheol Kim
Hong Joo Kim
Hong-Jin Kim
Hoon Kim
Hunkyung Kim
Hwa-Jung Kim
Hwan Kim
Hwi Young Kim
Hye Young Kim
Hye-Ji Kim
Hyeon Ho Kim
Hye-Sook Kim
Hye-Sun Kim
Hyeun Bum Kim
Hyoung-Il Kim
Hyoung-Ryoul Kim
Hyoungsoo Kim
Hyun Jin Kim
Hyun Kyung Kim
Hyun Soo Kim
Hyunggee Kim
Hyung-Seok Kim
Hyunjee Kim
Ilman Kim
Il-Man Kim
In Jung Kim
Inki Kim
J. Kim
Jae Bum Kim
Jae Heon Kim
Jaeil Kim
Jae-Jin Kim
Jae-Sung Kim
Jangwon Kim
Jeansok Kim
Jee Hyun Kim
Jenny Kim
Jeong Do Kim
Jeong Hun Kim
Jeonga Kim
Jeong-Min Kim
Jeongyun Kim
Ji Yeon Kim
Jieun Kim
Jihye Kim
Jin Kim
Jin Hyoung Kim
Jin Il Kim
Jin-Cheol Kim
Jin-Hwan Kim
Jinman Kim
Jinny Kim
Jinsoo Kim
Jinwon Kim
Jin-Wook Kim
Jisoo Kim
Ji-Won Kim
Jiyeun Kate Kim
Jong Seung Kim
Jongbin Kim
Jong-Ho Kim
Jonghwa Kim
Jonghwan Kim
Jongoh Kim
Jong-Shik Kim
Joo Seung Kim
Joohan Kim
Joomyeong Kim
Joon Mo Kim
Joong Hee Kim
Joonki Kim
Ju Young Kim
Jung-Ae Kim
Jung-Hyun Kim
Jungrack Kim
Jungsu Kim
Jung-whan Kim
Kami Kim
Kevin Kim
Ki Woo Kim
Ki-Jong Kim
Kwang Seok Kim
Kyong-Tai Kim
Kyoung-Mee Kim

Kyoungmin Kim
Kyoung-Nam Kim
Kyu Kim
Kyuhyung Kim
Kyung Kim
Kyung-Jin Kim
Kyungmi Kim
Leo Kim
Maengjo Kim
Manho Kim
Mi Na Kim
Min Hwan Kim
Min Jung Kim
Min Kyoung Kim
Mina Kim
Mi-Na Kim
Minkyoung Kim
Miran Kim
Myeung Kim
Myoung-Hwan Kim
Myung-Hee Kim
Nacksung Kim
Nak-Kyeong Kim
Nam Chul Kim
Nam Keun Kim
Nam Kyu Kim
Namshin Kim
Nayoung Kim
Peter Kim
Pilyoung Kim
Rakhyun Kim
Roger Kim
Sangho Kim
Sang-Ki Kim
Sangwoo Kim
SangYun Kim
Se-Chan Kim
Sejoong Kim
Seok-Won Kim
Seong Hwan Kim
Seong-Gon Kim
Seongho Kim
Seong-Hyop Kim
Seong-Jang Kim
Seong-Jun Kim
Seul Ki Kim
Seungchan Kim
Seung-Nam Kim
Seung-Sep Kim
Seungwon Kim
Shin-Hee Kim
Simon Kim
So Yeon Kim
Sohye Kim
Soo Wan Kim
Sooah Kim
Soochong Kim
Suhyun Kim
Suil Kim
Sun Kim
Sun Jung Kim
Sunae Kim
Sung Han Kim
Sung Joon Kim
Sung Woo Kim
Sung-Hoon Kim
Sung-Wan Kim
Sungwon Kim
Suwon Kim
Tae Kim
Tae Hoon Kim
Tae Jin Kim
Tae-Bum Kim
Tae-Hyung Kim
Tae-Woo Kim
Tae-Yoon Kim
Tania Kim
W Kim
W.K. Kim
Weon Kim
Won Kim
Won Tae Kim
Won-Il Kim
Woo Ho Kim
Woo Joo Kim
Woo Taek Kim
Wook Kim
Y.J. Kim
Yanghee Kim
Yang-Hyun Kim
Yeonjoo Kim
Yong Bae Kim
Yonggyun Kim
Yong-Mi Kim
Yoon-Young Kim
You-Me Kim
Young Bong Kim
Young Tae Kim
Young-Joon Kim
Young-Kyun Kim
Young-Mee Kim
Yu Shin Kim

Peter Kima
James Kimani
Bruce Kimball
Mark Kimberley
Kristopher Kimbler
Steven Kimbrough
Young-In Kim-Hoehamer
Ismael Aaron Kimirei
Yoshifumi Kimizuka
Bruce Kimler
Marek Kimmel
Joachim Kimmerle
Eva Kimonis
Sohei Kimoto
Michelle Kimple
Akihiro Kimura
Hideki Kimura
Hideo Kimura
Hirokazu Kimura
Hiroshi Kimura
Kazuhiro Kimura
Li Kimura
Shunsuke Kimura
Tadashi Kimura
Takuma Kimura
Tsuyoshi Kimura
Yoko Kimura
Yoshinobu Kimura
Cindy Kin
David Kinahan
Robert Kinast
Rebecca Kinaston
Paul Kinchington
Zsigmond Kincses
Zsigmond Tamas Kincses
Robert Kinders
Detlef Kindgen-Milles
Eveline Kindler
Heinz Kindler
Jochen Kindler
Joseph Kindler
Andrea Kindler-Röhrborn
Mark Kindy
Aaron King
Annie King
Bethia King
Brett King
Carina King
Charlotte King
Chih-Yen King
Deborah King
Derek King
Doug King
Gregory King
Jason King
Jessica King
Joshua King
Kevin King
Malcolm King
Marissa King
Michael King
Rachel King
Richard King
Robert King
Samuel King
Tommy King
Wendy C. King
Wade Kingery
Paul Kingham
John Kingma
Michael Kingsford
Richard Kingsford
Dawn Kingston
Tigga Kingston
Sanjay Kini
Milan Kinkhabwala
Margaret Kinnaird
Peter Kinnevey
Suna Kinnunen
Tomoshige Kino
Makoto Kinoshita
Tetsu Kinoshita
Kenichiro Kinouchi
Osame Kinouchi
Stefan Kins
Elaine Kinsella
Janni Kinsler
Vincent Kint
Hideaki Kinugasa
Damaris Kinyoki
Karin Kiontke
Deborah Kipp
Margaret Kipp
Matt Kipper
Tod Kippin
John Kipritidis
Yulia Kipryushina
Gokhan Kir
Orsolya Király
Koneti Kiran
Daniel Kirby
James Kirby

Janine Kirby
Karen Kirby
Russell Kirby
Tyler Kirby
Karin Kirchgatter
Christiane Kirchhoff
Louis Kirchhoff
Ann Kirchmaier
David Kirchman
Varvara Kirchner
Wolfgang Kirchner
Bruce Kirchoff
Girish Kirimanjeswara
Kohtaro Kirimura
Yohei Kirino
Erkan Kiris
Yoshimitsu Kiriyama
Adam Kirk
Jonathan Kirk
Nathan Kirk
Ryan Kirkbride
Alexis Kirke
Karla Kirkegaard
Lawrence Kirkendall
Grant Kirker
Natasha Kirkham
Pertti Kirkinen
Theo Kirkland
Kevin Kirkman
Jamie B. Kirkpatrick
Michael Kirkpatrick
John Kirkwood
Renata Kirkwood
Nameer Kirma
Laurence Kirmayer
Antonis Kirmizis
George Kirov
Yonal Kirsal
Alexander Kirsch
Heidi Kirsch
Irving Kirsch
Max Kirsch
Wollf Kirsch
Lawrence Kirschner
Joseph L. Kirschvink
Paul Kirshen
Lorrie Kirshenbaum
Julia Kirshner
Frank Kirstein
Mark Kirstein
Olivia Kirtley
Adam Kirton
Stewart Brian Kirton
Matt Kirwin
Krzysztof Kiryluk
Anna Kis
Bernhard Kis
Anton Kiselev
Antonina Kiseleva
Laszlo Kish
Shigeki Kishi
Shoji Kishi
Takuya Kishi
Taro Kishi
Osamu Kishida
Yasushi Kishimoto
Stephen Kishner
Thomas Kishor
Avinash Kishore
Nand Kishore
Raj Kishore
Sandeep Kishore
Dagmara Kisiela
Tomasz Kisielewicz
Andor Kiss
Hubert Kiss
Ibolya Kiss
Jozsef Kiss
Lorand Kiss
Rita Kiss
William Kisseberth
Alexei Kisselev
Katarzyna Kissimova-Skarbek
S. Kissler
Grace Kissling
Logan Kistler
Anna Kistner
Katalin Kis-Toth
Jeremy Kiszka
Munehiro Kitada
Hiroshi Kitagaki
Go Kitahara
Toru Kitahara
T. Kitai
Ken Kitajima
Shin-ichiro Kitajiri
Keiichi Kitajo
Yasunori Kitamoto
Akihiro Kitamura
Kenichiro Kitamura
Seiya Kitamura
Shumpei Kitamura

Katsunori Kitano
Satomi Kitano
Shigehisa Kitano
Azusa Kitao
Mitsutoshi Kitao
Yuichi Kitasako
Kazuyuki Kitatani
Jiro Kitaura
Haruki Kitazawa
Masashi Kitazawa
Takanari Kitazono
Douglas Kitchen
Maria Kitchen
Mark Kitchen
Newell Kitchen
Scott Kitchen
Henry Kitchener
John Kitchin
James Kite
Mary E. Kite
Soressa Kitessa
Maksim Kitsak
Paraskevi Kitsiou
Glenn Kitsune
Todd Kitten
Daniel Kitterer
Clemens Kittinger
John Kittinger
Roman Kittl
Rick Kittles
James Kitts
Yoshiaki Kiuchi
Dietmar Kivelitz
Minna Kivimäenpää
Vesa Kiviniemi
Shaye Kivity
Noah Kiwanuka
Ryoiti Kiyama
Yulia Kiyan
Eugene A. Kiyatkin
Tomoko Kiyohara
Toko Kiyonari
Ken Kiyono
Anil Kizha
Muhammed Kizilgul
Magnus Kjaergaard
Søren Kjærgaard
Jens Kjeldsen-Kragh
Birthe Kjellerup
Olav Sigurd Kjesbu
Elin Kjørsvik
Morten Kjos
Fayette Klaassen
Ingeborg Klaassen
Eline Klaassens
Cheryl Klaiman
Riina Klais
Silke Klamer
Petr Klan
Oya Klanten
Barbara Klapcinska
Rebecca Klaper
Sarah Klapproth
Jaroslav Klápste
Avihu Klar
Samara Klar
Eva Klaric
Philipp Klaritsch
George Klarmann
Ursula Klaschka
Zachary Klase
Henrikje Klasen
K.C. Klasing
Per Johan Klasse
Karen Klassen
R. Klassen
Nichole Klatt
Roberta Klatzky
Christoph Klauer
Sebastian Klaus
Miriam Klausberger
Jeffrey Klausner
Dag Klaveness
Caroline Klaver
Irena Kleckova
Leszek Kleczkowski
Claude Klee
Jorg Kleeff
Andis Klegeris
Juliane Klehmet
David Kleijn
Birgit Kleim
Evan Kleiman
Carina Klein
Cliff Klein
Eili Klein
Emily Klein
Günter Klein
Jochen Klein
John Klein
Karl Klein
Lloyd Klein

Mark Klein
Marlise Klein
Michael Klein
Peter Klein
Pierre Klein
Raymond Klein
Richard Klein
Ronald Klein
Sabine Klein
Stanley Klein
Ulf Klein
Petra Kleinbongard
Marisa Klein-Gitelman
Alex Kleinjan
Tobias Kleinjung
Daniel Kleinman
Hynda Kleinman
Christoph Kleinn
Kevin M. Kleinow
Ruth Kleinpell
Immo Kleinschmidt
Christoph Kleinschnitz
Maria Kleinstaeuber
Maria Kleinstäuber
Sabine Kleinsteuber
Thomas Kleinteich
Angelika Klein-Theyer
Ingo Kleiter
Sabina Kleitman
Bertrand Kleizen
Karel Klem
Michael Klemba
Friederike Klempin
Jochen Klenk
Elizabeth Klerman
Hilit Kletter
Alexa Klettner
Arnulf Kletzin
Alexander Klibanov
Alan Klide
Reinhold Kliegl
Catharina Klijn
Vaclav Klika
Richard Kliman
Lars Klimaschewski
Magdalena Klimek
Corrine Kliment
Yann Klimentidis
Paul Klimo
Pavel Klimov
Holger Klinck
Anthony Kline
David Kline
Michele Klingbeil
Carolyn Klinge
Aloysius Klingelhutz
Georgeanna Klingensmith
Jonathan Klingler
Elizabeth S Klings
Barbara Klink
Eveline Klinkenberg
Kenneth Klinker
Dennis Klinman
Amira Klip
Alexander Klishko
Janne Klitgaard
Kirstine Klitgaard
Vanja Kljajevic
Goran Klobucar
Agnieszka Kloch
Michael Klockmann
Andrzej Kloczkowski
Lorie Kloda
Roman Kloeckner
Brian Kloeppel
Stefan Kloeppel
Jennifer Kloepper
Casey Klofstad
Peter-Christian Klöhn
Jan Klohs
Michael Klompas
Bernard Klonjkowski
Elizabeth Klonoff
Kimberly Klonowski
Wlodzimierz Klonowski
Rafael Klorman
Gerhard Klösch
Karl Klose
Kathy Kloss
Thiago Kloss
Hilde Kloster Smerud
Fabian Klostermann
Christian Klotz
Luisa Klotz
Matthias Klotz
Shahnaz Klouche
Vasily Klucharev
Jochen Klucken
Bryan Kluever
Achim Klug
Christian Klug
Judith Kluge

Ricardo Kluge
Michael D. Kluger
Georgette Kluiters
Bernhard Klumpp
Chonticha Klungthong
Barbara Klupp
Michael Klüppel
Jan Klusmann
Glenn Klute
Harald Klüter
Markus Knaden
Sara Knaeps
Christine Knaevelsrud
Balázs Knakker
Tomas Knapen
David Knapp
Gregory Knapp
Karen Knapp
Michael Knapp
Oliver Knapp
Pamela Knapp
Peter Knapp
Sandra Knapp
Sonja Knapp
Stian Knappskog
Ewelina Knapska
Siegfried Knasmüller
Shirley Knauer
Brian Knaus
Ulla Knaus
Beat Knechtle
Mark A. Knepper
Paul Knepper
Friederike Knerlich-Lukoschus
Sarah Knerr
Rachel Knevel
Kali Kniel
Olaf Kniemeyer
Andrea Knight
Andrew Knight
Dave Knight
David Knight
Mark Knight
Robert A. Knight
Rod Knight
Dan Knights
Hanna Knihtila
Maria Knikou
Duleeka Knipe
Kristen Knipe
Corina Knipper
Marlies Knipper
Uwe Knippschild
Yuriy Knirel
Charles Knirsch
Darryn Knobel
M. Knobf
Jürgen Knobloch
Mirjam Knol
Lisa Knoll
Maximilian Knoll
Björn Knollmann
Thomas Knoop
Thomas Knopfel
Michael Knopp
Gerhard Knothe
Kirstin Knouse
Ben Knowles
Edd Knowles
Joshua Knowles
Anne Knowlton
Katharine Knowlton
Christine Knox
Dayan Knox
Travis Knuckles
Anders Knudby
Thomas Knudsen
Cheryl Knudson
Duane Knudson
Christina Knussen
Mitchell Knutson
Andrew Ko
Benjamin Ko
Bor-Sheng Ko
Byung Joon Ko
Chang-Yong Ko
Fanny Ko
Fung Chi Ko
Hsin-Kuo Ko
Hyun-Jeong Ko
In Kap Ko
Ja Kyong Ko
Jesang Ko
Ji Ko
Joshua Ko
Kisung Ko
Wen-Chien Ko
Wing Ko
Yu Ko
Yu-Lin Ko
Yoshihiro Kobae
Darwyn Kobasa

Gen Kobashi
Jon Kobashigawa
Akio Kobayashi
D. Kobayashi
Hisataka Kobayashi
Ichiro Kobayashi
Ichizo Kobayashi
Kazuto Kobayashi
Kazuya Kobayashi
Lindsay Kobayashi
Masahiro Kobayashi
Masaki Kobayashi
Miwako Kobayashi
N. Kobayashi
Scott Kobayashi
Shin Kobayashi
Shoko Kobayashi
Taisuke Kobayashi
Taku Kobayashi
Tamaki Kobayashi
Tatsuya Kobayashi
Teruyoshi Kobayashi
Tetsuo Kobayashi
Tetsuya Kobayashi
Tohru Kobayashi
Yasuki Kobayashi
Yasuo Kobayashi
Yoshinao Kobayashi
Yoshiro Kobayashi
Yuko Kobayashi
Yusuke Kobayashi
Dennis Kobelt
Martina Köberl
Tomasz Kobiela
Gary Kobinger
Beryl Koblin
Jennifer Koblinski
Stephan Koblmüller
Kerri Kobryn
Dylan Kobsar
Christopher Kobylecki
Çetin Koç
Jaroslav Koca
Rüya-Daniela Kocalevent
Bekir Kocazeybek
Simon Kocbek
E. Ahmed Koceir
Ayhan Kocer
Alexander Koch
Christof Koch
Flávia Koch
Florian Koch
Hagen Koch
Hans-Georg Koch
Ina Koch
Iring Koch
Jonathan Koch
Marcus Koch
Paul Koch
Philipp Koch
Thea Koch
Wayne Koch
Jacob Kocher
Thomas Kocher
Nils Koch-Henriksen
Yuta Kochi
Judith Kochmann
Marguerite Koch-Rose
Marleen Kock
Robin Köck
Ferdinand Köckerling
Janwillem Kocks
Maaike Kockx
Yoshiro Koda
Maheedhar Kodali
Hiroaki Kodama
Stephen Kodish
Gergana Kodjebacheva
Krithika Kodumudi
Dwight Koeberl
Muriel Koehl
Jeffrey Koehler
Karl Koehler
Karsten Koehler
Sara Koehler-McNicholas
Ger Koek
Thomas Koelewijn
Katja Koelkebeck
Christian Koelling
Philipp Koellinger
Albert Koelmans
Stefan Koelsch
Jessie Koen
Gijsje Koenderink
Rory Koenen
Christoph Koenig
Inke Koenig
Julian Koenig
Melissa Koenig
Sarah Koenig
Niko Koeniger
Joerg Koenigstorfer

Hannah Koenker
Constantianus Koenraadt
Tyler Koep
Klaus-Peter Koepfli
Michael Koeppen
Veronika Koeppen
Leonardo Koerich
Jane Koerner
Mirjam Koerner
Sally Koerner
Henk Koerten
Janneke Koerts
Johannes Koestel
Dirk Koester
Steve Koester
Ad Koets
Peter Koetsier
Mikhail Koffarnus
Jonathan Koffman
Barbara Kofler
David Kofler
Jiri Kofranek
Paulo Kofuji
Fumitaka Koga
Jun-ichiro Koga
Kenichi Koga
Masatoshi Koga
Ryuichi Koga
Donat Kögel
Manjunatha Kogenaru
Lydia Kogler
Michael Kogut
Tehila Kogut
Andrew Koh
Ho-Jin Koh
Hwee Ling Koh
Jae-Young Koh
Jin Koh
Mariko Koh
Seong-Ho Koh
Timothy Koh
Victor Koh
Woon-Puay Koh
Yasuhiro Koh
Kentaro Kohagura
Alison Kohan
Hamid-Reza Kohan-Ghadr
R. Sarah Kohansal
Katsuhiko Kohara
Michinori Kohara
Kevin Kohl
Karin Köhl
Connie Kohler
Pamela Kohler
Dieter Köhler
Iris Köhler
Ajay Kohli
Rahul Kohli
Rohit Kohli
Ruhail Kohli
Rüdiger Köhling
Rebekka Kohlmann
Sepp Kohlwein
Andre Kohn
David Kohn
Jacqueline Kohn
Satomi Kohno
Mark Kohr
Takahide Kohro
Holbrook Kohrt
Jun Kohyama
Ayato Kohzu
Masayo Koide
Roger Koide
Eddie Koifman
Masato Koike
Shinsuke Koike
Tatsuya Koike
Teruhiko Koike
Juha Koikkalainen
Rajeshwari Koilkonda
Diwa Koirala
Jari Koistinaho
Ari-Pekka Koivisto
Hisashi Koiwa
Akio Koizumi
Chie Kojima
Gotaro Kojima
Itaru Kojima
Seiji Kojima
Shihoko Kojima
Takashi Kojima
Chee Choy Kok
Jan Kok
Jen Kok
Niels Kok
Philippe Kok
Sang-Heng Kok
Victor Kok
Wouter Kok
Lauren Kokai
Khor Kok-Chin

David Kokel
Alina Kokhanenko
Yusuf Köklü
Ladislav Kokoska
George Kokotos
Sulev Koks
Daisuke Kokuryo
John Koland
Andrew Kolarik
Miroslav Kolarík
James Kolasinski
E. Kolb
Frederic Kolb
Nikolay Kolchanov
Michael Koldehoff
Crystal Kolden
John Kolega
Hemanta Koley
Nitin Kolhe
Vassilis Koliatsos
Jelena Kolic
Andrzej Kolinski
Yolanda Kolisa
Judith Kolkman
Miriam Kolko
Roxy Koll
Anup Kollanoor Johny
Nicole Kollars
Akos Koller
Ingrid Koller
Robert Koller
Sonja Kollers
Avinash Kollipara
Laxmikanth Kollipara
Stefan Kollmannsberger
Martin Kollmar
Richard Kollmar
Kathrin Kollndorfer
Afsal Kolloli
Jay Kolls
Nageswara Rao Kollu
Harald Kolmar
Carolin Kolmeder
Alex Kolodkin
Gerald Kolodny
Jeremi Kolodziejek
Dmitry Kolomenskiy
M. B. Kolozsvary
Mette Kolpen
Anne-Brit Kolsto
Simon Kolstoe
Ismail S. Koltas
James Koltes
Walter Koltun
H. Komai
Kanako Komaki-Yasuda
Padmini Komalavilas
Anton Komar
Nicholas Komar
Martina Komarkova
Anthony Komaroff
Haruki Komatsu
Yoshihiro Komatsu
Takao Komatsuda
Toshihiko Komatsuzaki
Nobuyuki Komazawa
Francis Kombe
Barry Komisaruk
Andrey Komissarov
Ramakrishna Kommagani
Yoshihiro Komohara
Akinola Komolafe
Satoshi Komoto
Katie Kompoliti
Elizaveta Kon
Valentina Kon
Koffi Konan
Jürgen Konczalla
Kelika Konda
Ganesh Kondabattula
Paturu Kondaiah
Anand Kondapi
Kanthi Kiran Kondepudi
Subramanyam Kondeti
Hidehiro Kondo
Hideki Kondo
Kazuhisa Kondo
Michelle Kondo
Saturo Kondo
Takashi Kondo
Toshikazu Kondo
Yutaka Kondo
Michio Kondoh
Dániel Kondor
Ganesh Konduri
Nagarjun Konduru
Anthony Kong
Bo Kong
Christina Kong
Fanbin Kong
Fanmei Kong
Feng Kong

Grace Kong
Jiming Kong
Jin Kong
Ka-Yiu Edwin Kong
Lan Kong
Ling-Dong Kong
Lingrang Kong
Lingyi Kong
Lisheng Kong
Qingfei Kong
Qingpeng Kong
Qingzhong Kong
Qiusheng Kong
Richard Kong
Rui Kong
Shengchun Kong
Wei Kong
Weijun Kong
Xiangfeng Kong
Xiangrong Kong
Xiangyu Kong
Yichun Kong
Yun Kong
Anine Kongelf
Alice Kongsted
Gerhardt Konig
Jochem König
Kai König
Matthias König
Frits Koning
Ruud H. Koning Koning
Y. Konishi
Prasad Konkalmatt
Anish Konkar
Barbara Konkle
Deborah Konkle-Parker
Monika Konnert
Kotaro Konno
Norifumi Konno
Tomohiro Konno
Masahiro Kono
Elisa Konofagou
Veronika Konok
Tadeusz Kononowicz
Ivo Konopásek
Matt Konopinski
Anna Konovalova
Daniel Konrad
Franziska Konrad
Sara Konrath
Mary Konsolaki
Evdokimos Konstantinidis
Eleni Konstantinou
Ioannis K Konstantinou
Tsuneo Konta
Vesa Kontinen
Vasilis Kontis
Dimitris Kontodimas
Evangelos Kontopantelis
Georgios Kontorinis
Despina Kontos
Dimitris Kontoyiannis
Irina Kontsevaya
Kevin Konty
Kishori Konwar
Viktoria Konya
Brian Koo
Chi Wan Koo
Hyun Koo
Sunwoo Koo
Terry Koo
Winston Koo
Michael Koob
Shahriar Koochekpour
Katherine Kooij
Sander Kooijmans
Mirjam Kool
Olivier Koole
Jeroen Kooman
Yen Ling Koon
Karanjit Kooner
Demian Koop
René Koopman
Sietse-Jan Koopmans
Joseph Koopmeiners
Kathelijne Koops
Maarten Koornneef
Bernd Koos
B.P.G. Koot
Neeltje Kootstra
Ian Kopacka
Alexander Kopatz
Dennis Kopecko
Jan Willem Koper
Helen Kopnina
Jeffrey Kopp
Marcel Kopp
Ulla Kopp
Georgia Koppe
Janna Koppe
Laetitia Koppe
Kadri Koppel

Joel D. Kopple
Ravi Koppolu
Germán Kopprio
Robert Koprowski
Suzanne Koptur
Jakub Kopycinski
Jenya Kopylov
Karen Korabik
Bato Korac
Günther Koraimann
Sergei Koralov
Nicolas Koranyi
Judith Korb
Jan Korbel
Masaaki Korenaga
Daniel Korevaar
Tim Korevaar
Martina Korfei
Mayuresh Korgaonkar
Kaveri Korgavkar
Julia Koricheva
Netanel Korin
Anton Korinek
Gilly Koritzky
Miikka Korja
Hannu Korkeala
Henri Korkes
Selcuk Korkmaz
Thomas Korn
Anita Körner
Roman Körner
Konstantin Kornev
Panagiotis Kornilios
Peter Kornprat
Abraham Korol
Alexey Koronovskii
G. Korosoglou
Konstantin Korotkov
Siran Koroukian
Panagiotis Korovessis
Ilkka Korpela
Katri Korpela
Nicholas Korres
Nicholas E. Korres
Sergey Korshunov
Monica Korsnes
Zoltan Korsos
Lise Korsten
Insa Korten
Marleen Kortenoeven
Michiel Korthals
Ozlem Korucuoglu
Izabela Korwel
Marek Korzeniowski
Donna Korzick
Viktor Korzun
Lidia Kos
Martin Koš
Monika Kosacka
Takayuki Kosaka
Carl Koschmann
Mirja Koschorke
Isabelle Koscinski
Ewa Kosciuczuk
Tomokazu Koshiba
Isao Koshima
Ajeesh Koshy Cherian
Urszula Kosikowska
Jiri Kosinka
Carolin Kosiol
Matthew Koski
Kostantinos C Koskinas
Ralf Kosma
Arkadiusz Kosmala
Cassandra Kosmidou
Victor Kosmopoulos
Simone Kosol
Klára Kosová
Michael Kosoy
Michael Koss
Jens Kossmann
Michael Kossmeier
Malgorzata Kossowska
Amanda Kost
Christian Kost
Rumen Kostadinov
Vladimír Koštál
Zvonko Kostanjcar
Andreas Koster
Annemarie Koster
Raphael Koster
Roelof Koster
Reinhard Köster
Rena Kosti
Vladimir Kostic
Deliana Kostova
Tatiana Kostrominova
Shunichi Kosugi
Patrycja Koszalka
Jacek Kot
Janaiah Kota
Eiji Kotani

Ahmed Kotb
B. Kotchoubey
Adaikala Koteswari
Chandrasekhar Kothapalli
Kumar S. Kothapalli
Naga Rama Kothapalli
Ute Kothe
Kavitha Kothur
Ewa Kotlarska
Aleksandra Kotlinska-Lemieszek
Primoz Kotnik
Roman Kotov
Martyna M. Kotowska
Apostolos Kotsialos
Ioannis Kotsianidis
Joanne Kotsopoulos
Nikolaos Kotsopoulos
Dimitra Kotsougiani
Mark Kotter
Shyamasundaran Kottilil
Jack Kottwitz
M. Kotula-Balak
Atul Kotwal
Paul Kotzbauer
Andrew Kotze
Joerg Kotzerke
Junping Kou
Roland Yao Wa Kouassi
Andrew Kouba
Georgios Koubouris
Namkje Koudenburg
Panagiotis Kougias
Azam Kouhkan
Ourania Koukoura
M. Koukourakis
Nikolaos Koundourakis
Richard Koup
Dimitrios Kouretas
Anargyros Kouris
Yiannis Kourkoutas
Mahmoud Koushesh Saba
Evanthia Kousi
Chandrasekar Kousik
Antonios Kousoulis
Irena Koutna
Michael Koutsilieris
George Koutsoudakis
Wilma Koutstaal
Andrej Kovac
Dejan Kovac
Eva Kovacevic
Melissa Kovach
Borut Kovacic
Jason Kovacic
Agnes Melinda Kovacs
Krisztina Kovacs
Laszlo Kovacs
Stephanie Kovacs
Mihály Kovács
Tibor Kovács
Susan Koval
Zuzana Kovalova
Ilona Kovalszky
Petri Kovanen
Joy Kovar
Zrinka Kovarik
Jaromír Kovárík
Petia Kovatcheva-Datchary
Charles Koven
Sara Kover
Michal Kovo
Stephanie Kowal
Bernd Kowall
M. Anna Kowalska
Markus Kowarik
Akihiro Koyama
Alain Koyama
Asuka Koyama
Maki Koyama
Tatsuki Koyama
Toshihiro Koyama
Kaloian Koynov
Igor Kozak
Leslie Kozak
Dima Kozakov
Richard Kozarek
Elisa Kozasa
Sebastian Kozerke
Jacek Koziel
Alan Kozikowski
Elena Kozina
Zoltan Kozinszky
Ken Kozloff
Andrey Kozlov
Sergey Kozlov
Pamela Kozlowski
Zbynek Kozmik
David Kozono
Lukasz Kozubowski
Renata Kozyraki
Varvara Kozyreva
Anita Kozyrskyj

Samory Kpotufe
Claudine Kraan
Anne Marie Krachler
Anne-Marie Krachler
Joep Kraeima
Alexandra Kraemer
Fredric Kraemer
Kenneth Kraemer
Nicolle Kraenkel
Kurt Kraeuchi
Mary Kraft
Peter Kraft
Christian Krägeloh
Kristian Kragholm
Thomas Krahe
Peter Kraiczy
Dimitri Krainc
Douglas Krakower
Andrej Kral
John Kral
Martina Kralinger
Anastasia (Natasha) Kralli
Rodger Kram
Achim Kramer
Andrew Kramer
Boris Kramer
Helmut Kramer
Jennifer Kramer
Jill Kramer
John Kramer
Mario Kramer
Phil Kramer
Randall Kramer
Richard Kramer
Benjamin Krämer
Bernhard Krämer
Julia Krämer
Oliver Krämer
Andrei Kramerov
Julia Krammer
Kamil Kranc
Elena Kranioti
Branka Kranjc
Julie Krans
Keshav Kranthi
Slavica Krantic
David Krantz
Alexander Kranz
Daniel Krappmann
Sven Krappmann
Christian Krarup
Alla Krasikova
Maria Krasilnikova
Allan Krasnik
Ines Krass
Vessela Krasteva
Lukáš Kratochvil
Anton Kratz
Molly Kratz
Robert A. Kratzke
Mary Krauland
Alexander Kraupner
Edward Kraus
Helen Kraus
Johann Kraus
Nina Kraus
Peter Kraus
Annalinda Krause
Duncan Krause
E. Tobias Krause
Kerstin Krause
Martin Krause
Matthew Krause
Mauricio Krause
Niklas Krause
Philip Krause
Robert Krause
Roland Krause
Vanessa Krause
Ben Krause-Kyora
Stefan Krauss
Csilla Krausz
Allen Kraut
Ellen Krautkrämer
Andra Krauze
Alexandra Kravchenko
Vladimir Kravchenko
Richard Kravitz
Meg Krawchuk
Peter Krawczel
Greg Krawczyk
Marcin Krawczyk
Michal Krawczyk
Pawel Krawczyk
Przemek Krawczyk
Michael Krawinkel
Klaus Kraywinkel
Emanuel Krebs
Mark Krebs
Michael Krebs
Nancy Krebs
Shelly Krebs

Cory Krediet
Paul Krediet
Raymond Krediet
Pamela Kreeger
Antonia Krefeld-Schwalb
Stefan Kreft
Clint Krehbiel
Brent Kreider
Benjamin Kreifelts
Bernd Kreikemeyer
Rebecca Kreiling
Gabriel Kreiman
Aimee Kreimer
Alexandra Kreins
Barry Kreiswirth
Martyna Krejmer-Rabalska
Natali Krekeler
Iwona Krela-Kazmierczak
Frank Krell
Peter Krell
Dimitry Krementsov
David Krementz
Colin Kremer
Patricia Kremer
Johanna Kremer Hovinga
Jan Kremers
Vladimír Kren
Marco Krengli
Hartmut Krentz
William Krenzer
Joan Krepinsky
Vladimir Kreslavski
W. John Kress
Jochen Kressler
Irene Kretchy
Alexander Kretschmer
Dorothee Kretschmer
Karsten Kretschmer
Robert Kretsinger
Antoniana Krettli
Colin Kretz
Carole Kretz-Remy
Michael Kreuter
Susanne Kreuzer-Redmer
Jürgen Kreuzwieser
Carmen Krewer
Grigorios Krey
Wolfgang Kreyling
Skirmantas Kriaucionis
Steven Kridel
Verena Kriechbaumer
Peter Krieg
Alison Kriegel
Patrik Krieger
Sophie Krieger
Anja Krieger-Liszkay
Lance Kriegsfeld
Susi Kriemler
John Kriesel
Detlef Krieter
Jillian Kril
Sheldon Krimsky
Michael Krings
Sharon Krinsky-McHale
James Krinsley
Kewal Krishan
Rebecca Krisher
Anand Krishna
Neel Krishna
D. Krishna Kumar
R. Krishnakumar
Subramanian Krishnakumar
Nandini Krishnamoorthy
Narayanan Krishnamurthi
Kaarkuzhali Krishnamurthy
Karthik Krishnamurthy
Prasanna Krishnamurthy
Anand Krishnan
Arjun Krishnan
Giri Krishnan
K. Krishnan
Manju Krishnan
Natraj Krishnan
Nithya Krishnan
Ramaswamy Krishnan
Saloni Krishnan
Vengadesan Krishnan
Venkatesh Krishnan
Ashok Krishnaswami
Jagdish Krishnaswamy
Jayendra Krishnaswamy
Ghattu Krishnaveni
Bernhard Krismer
Jennifer Kriss
Eugene Krissinel
William Kristan
Erik Kristensen
Michael Kristensen
Søren Kristensen
Tibor Kristian
Glen Kristiansen
Christopher Kristich

Hook Kristin
Arni Kristjansson
Julijana Kristl
Michael Kristo
Ladislav Kristoufek
Delane Kritsky
Paula Kriz
David Krizaj
Kristina Krklec
Saskia Kroeckel
Candyce Kroenke
Hannes Kröger
Jesper Krogh
Trine Krogh-Madsen
Michelle Krogsgaard
Tore Krogstad
Jona Krohn
Stephan Krohn
Tim Krohne
Matthias Kroiss
Thomas Kroj
Paal Krokene
Fernanda Kroker
Jessica L Krok-Schoen
Elzbieta Krol
Andrzej S. Krolewski
Bastiaan Krom
Irving Kron
Michael Kron
Tomas Kron
Andreas Kronbichler
Lise Kronborg
Ziad Kronfol
Martin Krönke
James Kronstad
Erna Kroon
Ian Krop
Eric Kropf
Matthias Kropf
Andrew M. Kropinski
Matthew Krosch
Ursula Krotscheck
Johns Krouse
Susan Krown
Janet Krska
Jürgen Krücken
Dilja Krueger
Joerg Krueger
Robert Krueger
Stacy Krueger-Hadfield
Edward Krug
Joachim Krug
Ruan Kruger
Andreas Krüger
Björn Krüger
Karsten Krüger
Kerstin Krüger
Markus Krüger
Nadine Krüger
Ilja Kruglikov
Andrey Kruglov
Nicole Kruh
Willem Kruijer
Marieke Kruip
Marta Kruk-Slomka
Marcos Krull
Thierry Krummel
Christian Krupke
Michael Kruppa
Clemens Kruse
Michael Kruse
Paul Krusic
Oleg Krut
Michael Krützen
Maarja Kruusmaa
Frank Kruyt
Per Kryger
Vera Krymskaya
Angelos-Miltiadis Krypotos
Richard Kryscio
Andriy Kryshtafovych
Karolina Krysinska
Gerald Krystal
Dominik Krzanicki
Marcin Krzystanek
Martin Krzywinski
Daniel Ksepka
Nicholas Ktistakis
Nam Su Ku
Seung-Yup Ku
Meng-Kiat Kuah
Jie Kuai
Chee Sian Kuan
Chien-Tsun Kuan
Garry Kuan
Win Sen Kuan
Yu-Hsiang Kuan
Yung-Shu Kuan
Jialiang Kuang
Jian-Fei Kuang
Rui Kuang
Yan-Ping Kuang

Barthelemy Kuate Defo
Hiroshi Kuba
Steven Kubalak
Karen Kubena
Bruno Kubiak
Jacek Kubiak
Andreas Kubicek
John Kubie
Lubica Kubikova
Leszek Kubin
Robert Kubina
Jason Kubinak
Ralf Kubitz
Janet Kübler
Keitaro Kubo
Tomohide Kubo
Tomohiko Kubo
Toshio Kubo
Chieri Kubota
Kengo Kubota
Naoto Kubota
Satoko Kubota
Yoshitaka Kubota
Flavia Kubrusly
Gregory Kucera
Michal Kucera
Otto Kucera
Amy Kuceyeski
Andreas Kuchar
Karoline Kuchenbaecker
Florian Kuchenbauer
Anna Kuchina
Stefanie Kuchinsky
Suresh Kuchipudi
Rachel Kuchtey
Ulrich Kück
Can Kucuk
Murat Küçükevcilioglu
Ozlem Kucukoglu
Krzysztof Kuczera
Takashi Kuda
Raphael Kudela
Andrzej Kudlicki
Makoto Kudo
Naomi Kudo
Seishi Kudo
Misha Kudryashev
N.S. Kudryasheva
Indira Kudva
Yee Cheng Kueh
Christian Kuehn
Sarah Kuehne
Thomas D. Kuehne
Dana Kuehnel
Christine Kuehner
Mathias Kuemmerlen
C. Kuempel
Christopher Kuenze
Wayne Kuenzel
Joshua Kuethe
Jan Kuever
Donald Kufe
Jennifer Kugel
Dimitris Kugiumtzis
Heiner Kuhl
Anja Kühl
Hilmar Kühl
Michael Kühl
Angela Kuhla
Barry Kuhle
Kate Kuhlman
Martin Kuhlwilm
Joachim Kuhn
Peter Kuhn
Randall Kuhn
Sabine Kuhn
Steven Kuhn
Thomas Kuhn
Tobias Kuhn
Bernhard Kühn
Hartmut Kühn
Lukas Kühn
Wolfgang Kühn
Lisa Kuhns
Anne Marie Kuijpers-Jagtman
Gerhardus Kuiper
Jan-Herman Kuiper
Benjamin Kuipers
Praveen Kujal
Miiamaaria Kujala
Sanni Kujala
Omar Kujan
Elizabeth Kujawinski
Jennifer Kuk
Thomas Kukar
Anna Kukekova
Akiko Kukita
Toshio Kukita
Jyrki Kukkonen
Piotr Kukla
Pawel Kuklik
Pavel Kuksa

Maria Kukuruzinska
Katherine Kula
Alanna Kulchak Rahm
Franceli Kulcheski
Gretchen Kuldau
Josipa Kules
David Kulesh
Margarete Kulik
Irene Kuling
Jaime Kulisevsky
Janina Kulka
Avanti Kulkarni
Gourihar Kulkarni
Hemant Kulkarni
Jayashri Kulkarni
Mandar Kulkarni
Mangesh Kulkarni
Nachiket Kulkarni
Rahul Kulkarni
Ritwij Kulkarni
S. Kulkarni
Sakil Kulkarni
Savita Kulkarni
Shibani Kulkarni
Snehal Kulkarni
Subhash Kulkarni
Tejaswini Kulkarni
Louisa Kulke
Martin Kulldorff
Christopher Kullenberg
Lewis Kuller
Ottmar Kullmer
Christopher Kulp
Parul Kulshreshtha
Ritu Kulshreshtha
Erik Kulstad
Jens Kultima
Hye-Chung Kum
Kathleen Kuman
Cyrus Kumana
Abhishek Kumar
Addanki Kumar
Ajay Kumar
Ajit Kumar
Alok Kumar
Ambuj Kumar
Amit Kumar
Anil Kumar
Anupriya Kumar
Arun Kumar
Arvind Kumar
Ashnil Kumar
Ashok Kumar
Ashutosh Kumar
Ashwani Kumar
Ashwini Kumar
Aundy Kumar
Bhavna Kumar
D. Kumar
Deepak Kumar
Dhiraj Kumar
Dhirendra Kumar
Dinesh Kumar
Haribalan Kumar
Himanshu Kumar
Jitendra Kumar
Juhi Kumar
Manish Kumar
Manoj Kumar
Maya M Kumar
N. Pradeep Kumar
Nagi Kumar
Naveen Kumar
Neelima Kumar
Neeraj Kumar
Nishant Kumar
P.L. Kumar
Parameet Kumar
Paul Kumar
Pawan Kumar
Pradeep Kumar
Prashant Kumar
Rahul Kumar
Rajeev Kumar
Rajesh Kumar
Rajiv Kumar
Ramaiah Kumar
Ramesh Kumar
Ranjeet Ranjan Kumar
Sachil Kumar
Sachin Kumar
Sameer Kumar
Sandeep Kumar
Sanjay Kumar
Santhosh Kumar
Santosh Kumar
Satish Kumar
Shaji Kumar
Smita Kumar
Srividya Kumar
Subodh Kumar
Sunil Kumar

Suresh Kumar
Surinder Kumar
Sushil Kumar
Uttam Kumar
Vijay Kumar
Vijaya Kumar
Vikas Kumar
Vinod Kumar
Vipan Kumar
Vipin Kumar
Vivek Kumar
Mohan Kumar Haleyur Giri Setty
Santosh Kumar T.R.
Darshani Kumaragamage
Govindasamy Kumaramanickavel
Dinakantha Kumararatne
N. Pradeep Kumarasamy
Vasanthapuram Kumaraswami
Deepak Kumaresan
Jaya Kumari
Jyothi Kumari
Neelam Kumari
Saru Kumari
Veena Kumari
Dharam Kumbhani
Suchit Kumbhare
Toshiaki Kume
Tsutomu Kume
Joseph Kumka
Michael Kummer
Terrance Kummer
Wolfgang Kummer
Robert Kumpf
Karol Kumpfer
Mont Kumpugdee-Vollrath
Kattareeya Kumthip
Li Kun
Uday P. Kundap
Dale Kunde
Wilfried Kunde
Anna Kundel
Vikas Kundra
Bijoy Kundu
Gopal Kundu
Kousik Kundu
Manikuntala Kundu
Pallob Kundu
Shilajit Kundu
C.T. Kung
Chun-Chia Kung
Takekazu Kunieda
Mark Kuniholm
William Kunin
Ken Kunisaki
Yoshihiko Kunisato
Jun Kunisawa
Dorit Kunkel
Brian Kunkle
Mervi Kunnasranta
Sreenivasan Kunnatheeri
Radhika Kunnavil
Ajaikumar Kunnumakkara
Nikolaj Kunøe
Anton Kunst
Hans-Jörg Kunte
Duangkamol Kunthalert
Hiroshi Kunugi
Setor Kunutsor
Patrik Kunz
Stefan Kunz
Werner Kunz
Wolfram Kunz
Micha Kunze
Reiner Kunze
Andreas Kunzmann
Marcus Kunzmann
Caroline Kuo
Chang-Fu Kuo
Chao-Yang Kuo
Chao-Yin Kuo
Chen-Yen Kuo
Chia-Hua Kuo
Ching-Chuan Kuo
Chin-Lung Kuo
Chi-Yun Kuo
Christin S Kuo
Hann-Chorng Kuo
Han-Yueh Kuo
Hsiao-Che Kuo
I. Fan Kuo
John Kuo
Li-Min Kuo
Li-Wei Kuo
Mei-Chuan Kuo
Michael Kuo
Shiu-Ming Kuo
Sung-Hsin Kuo
Yao Kuo
Ya-Wen Kuo
Yong-Fang Kuo
Yuan-Hung Kuo
Yu-Ting Kuo

Alptekin Küpçü
Kornelius Kupczik
Frieke Kuper
Marcelo Kuperman
Victor Kuperman
Ron Kupers
Gary Kupfer
John Kupfer
Linda Kupfer
Peter Kuppens
Sundaram Kuppu
Saranya Kuppusamy
Dhandapani Kuppuswamy
Alexander Kuprin
Hiroki Kurahashi
Shigehiro Kuraku
Takashi Kuramoto
Makoto Kurano
Harley Kurata
Kosaku Kurata
Shigeru Kuratani
Lisa Kurczewski
Shigeo Kure
Boris Kurganov
Gino Kurian
Haruko Kurihara
Hidemi Kurihara
Toshihide Kurihara
Danielle Kurin
Tomoko Kurita-Ochiai
Ajay Kuriyan
Daniel Kurjak
Satu Kurkela
Helen Kurki
Katelyn Kurkul
Nyoman Kurniawan
Satoshi Kuroda
Shinji Kuroda
Asato Kuroiwa
Kenji Kurokawa
Manabu Kurokawa
Mineo Kurokawa
Keiichi Kuroki
Kimiko Kuroki
Masayuki Kurosaki
Deborah Kurrasch
Florian Kurschus
Beth Kurt
Bollmann Kurt
Özgür Kurt
Vartan Kurtcuoglu
Stefan Kurtenbach
Ann Kurth
Margaret Kurth
Krishna Kurthkoti
Senem Kurtoglu
Christian Kurts
Mikko Kurttila
Ira Kurtz
Theodore Kurtz
Isaac Kurtzer
Teja Kuruganti
Shree Kurup
Michio Kuruso
Ralf Kurvers
Patrick Küry
Eduard Kurz
Mateusz Kurzawski
Shigeru Kusagawa
Josip Kusak
Yuko Kusakabe
Peter Kusalik
Stefan Kusch
Donna Kusewitt
Toshihiro Kushibiki
Tetsuo Kushiro
Akifumi Kushiyama
Jake Kushner
Alena Kushniarevich
Vitaly Kushnir
Avadhesh Kushwaha
Ashima Kushwaha Bhardwaj
Kwadwo Kusi
Claudia Kusmic
Oliver Kuss
Edo Kussell
Diederik Kuster
Niels Kuster
Dennis Küster
Shigeru Kusumoto
Andrzej Kutarski
Mzia Kutateladze
Jason Kutch
Anton Kutikhin
Maja Kutlaca
Hana Kutlikova
Bryan Kutner
Verena Kutschera
Tiit Kutser
Nobuyuki Kutsukake
Elizabeth Kutter
Raman Kutty

Marta Kutwin
Satu Kuure
Johanna Kuusisto
Jaana Kuuskeri
Mikko Kuussaari
Masanari Kuwabara
Takashige Kuwabara
Keisuke Kuwahara
Ichiro Kuwahira
Tomoyuki Kuwaki
Tomomi Kuwana
Serdar Kuyucak
Chris Kuzawa
Alan Kuzirian
Ivan Kuzmin
Andrei Kuzminov
Alexey Kuznetsov
Boris Kuznetsov
Kiyotaka Kuzushima
Masafumi Kuzuya
Miloslav Kverka
Jan Kvet
Kristina Kvile
Brian Kvitko
Hau Kwaan
Bernard Kwabi-Addo
Cheol Kwak
Mi-Kyoung Kwak
Suryang Kwak
Youn-Sig Kwak
Ada Kwan
Ann Kwan
Johnny Kwan
Raymond Kwan
Douglas Kwazneski II
Artur Kwiatkowski
Malgorzata Kwiecien
Marcin Kwissa
Anne Kwitek
Boon-Chong Kwok
Hang Fai Kwok
Man Ki Kwok
Sylvia Y. C. L. Kwok
Timothy Kwok
Xinjin Kwok
Young Kwok
Hyuk-Moo Kwon
Hyuk-Sang Kwon
Hyung Wook Kwon
Janice Kwon
Jung-Hwan Kwon
MinHyuk Kwon
MiYoung Kwon
Ohbyung Kwon
Ohseok Kwon
Oran Kwon
Ronald Kwon
Seung-Hwan Kwon
Sung Woo Kwon
Tae-Geon Kwon
Tae-Hwan Kwon
Yong Hoon Kwon
Young Jik Kwon
Younghoon Kwon
Jacky Kwong
Peter Kwong
Tin Kyaw
Jennifer Kyle
Tina Kyndt
Kyriakos Kypreos
Chris Kypridemos
Una Kyriacos
Constantinos Kyriakis
Stavroula Kyriazi
Ioannis Kyrou
Vasileios Kyttaris
Kim Kyunggon
Evan Kyzar
Coralie L'Ollivier
Antonio La Cava
Anne Camille La Flamme
Susanne la Fleur
Stefano La Malfa
Roberto La Marca
Rafael La Perna
Patrick La Riviere
Camilo La Rota
Giuseppe La Torre
Timo Laaksonen
Liedewij Laan
Maris Laan
Tiina Laatikainen
Tracey-Lea Laba
Daniel LaBarbera
José Labarere
Jean Labarre
Vincent Labatut
Jessy Labbe
Cyril Labbé
A. Desiree LaBeaud
Bernard Labedan
Vinod Labhasetwar

Nazzarena Labo
Patrick Labonte
Mario Laborda
Aurore Labourel
Mariano Labrador
Pascal Labrousse
Lucja Labuda
Andrew Lac
Lucas Lacasa
Federica Lacasella
Ricardo Lacchini
Donato Lacedonia
Elisa Lacerda
Joao Lacerda
James Lacey
Simon Lacey
Joseph Lachance
Marc-André Lachance
John Lachin
Salil Lachke
Nico Lachmann
Nathan Lachowsky
Birgit Lackner
Michaela Lackner
Alicia Lacoma
Lilian Lacourpaille
Tamara E. Lacourt
Francesco Lacquaniti
Benoit Lacroix
Christophe Lacroix
Sébastien Lacroix-Desmazes
D. Borden Lacy
Dean Lacy
Ryan Lacy
Lara LaDage
John Ladbury
Andrea Ladd
Bob Ladd
Brenton Ladd
Michael Ladegaard
Nimzing Ladep
Friedrich Ladich
Ana Ladio
Stephan Ladisch
Andrew Ladle
Jason Ladner
Magalie Ladouceur
Mary Jo LaDu
Stephanie Läer
Oliver Laeyendecker
Virginie Lafage
Pierre Lafaye
Rosa Lafer-Sousa
Shawn Laffan
Muriel Laffargue
Kevin Lafferty
John Laffey
Susan E. LaFlamme
Monique Lafon
Kathryn Lafond
Jerome Lafont
Eric Lafontaine
Yves Lafort
Audrey Lafrenaye
Benjamin Lafrentz
William Lafuse
Robert Lafyatis
Marie Laga
Erwann Lagabrielle
Diane Lagace
Thomas Lagace
Neil Lagali
Marina Laganaro
Alfonso Lagares
Joao Lagarto
Linda Lagasse
Claudia Lage
Ricardo Lage
Vincent Lagente
Karin Lagesen
Claire Lagesse
Stephan Laggai
Franco Laghi-Pasini
Kamel Laghmani
Joao Lago
Francesco Lagona
Carlos Lago-Pena
Susanna Lagorio
Nelson A. Lagos
Doris Lagos-Kutz
Chad LaGrange
Paméla Lagrange
Manuel Lagravere
Mark Lagrimini
Katrien Lagrou
Clément Lagrue
Fabiana Laguna
Pablo Laguna
Andres Laguna-Fernandez
Ljerka Lah
Bireswar Laha
James Laham

Ian Lahart
A. Lahat
Yael Lahav
Ismail Laher
Vicente Lahera
B. Lahey
Timothy Lahey
Kajal Lahiri
Ramanuj Lahiri
Soumen Lahiri
Tim Lahm
Salim Lahmiri
Chloé Lahondère
Lies Lahousse
Agustin Lahoz
Leo Lahti
Robert Lahue
Angeline Lai
C. H. Lai
Chengguang Lai
Chien-Chen Lai
Chih-Cheng Lai
Ching-Shu Lai
Chin-Hsing Lai
Chi-Yu Lai
Chou-Cheng Lai
Chun-Fu Lai
Chyh-Ming Lai
Derrick Y.F. Lai
Eric C.H. Lai
Feipei Lai
HuiChuan Lai
Hung-Wen Lai
Jonathan Lai
Kuan-Lin Lai
Lucy Lai
Meng-Chuan Lai
Ming-Derg Lai
Ming-Wei Lai
Mon Jou Lai
Olimpia Lai
Rachel Lai
Samuel Lai
Shengjie Lai
Shen-Hao Lai
Tai-Shuan Lai
Te-Jen Lai
Timothy Lai
Tsung-Hsuan Lai
Wai Lung Lai
Wen Cheng Lai
Wenli Lai
Xiaochun Lai
Xinsheng Lai
Yu Kay Lai
Yu-Kuen Lai
Evagelia Laiakis
Rebekah Laidsaar-Powell
Christian Laier
Christopher Laine
Carlo Laing
Ingrid Laing
William Lainhart
Ines Lains
Angela Laird
David Laird
Diana Laird
Sue Laird
Triin Laisk-Podar
Dennis LaJeunesse
Todd LaJeunesse
Kate Lajtha
Timo Lajunen
Gabriella Lakatos
Douglas Lake
Jordan Lake
Spencer Lake
Daniël Lakens
Nelly Lakestani
Yihunie Lakew
Laila Lakhal
Samira Lakhal-Littleton
T. N. Lakhanpal
Naoufal Lakhassassi
Subhash Lakhotia
Aparna Lakkaraju
Cynthia Lakon
Jeffrey Lakritz
Dan Laks
Madepalli Lakshmana
Imayavaramban Lakshmanan
Manu Lakshmanan
D. Shanthana Lakshmi
Sribalaji Lakshmikanthan
Kamakshi Lakshminarayan
Thangavel Lakshmipriya
Laura Lakusta
Ashish Lal
B. Lal
Geeta Lal
Girdhari Lal
Harbans Lal

Jawahar Lal
Rup Lal
Rahmi Lale
Wim Laleman
Hariharan Lalgudi
Sarasi Lalithsena
Patrice Lalive
Kristi Lall
Marco Lalle
Enzo Lalli
John Lally
Alexis Lalouette
Benjamin Lam
Byron Lam
Ching-Wan Lam
Connie Lam
Ernest Lam
Hanh Lam
Jenny Lam
Phung Lam
Sandi Lam
Tommy Lam
Ying Wai Lam
Theresa Lamagni
Fotini Lamari
Trond Lamark
Laurent Lamarque
Jonathan LaMarre
José Ramón Lamas
Monica Lamas
Ruben Lamas-Pinheiro
Damon Lamb
David Lamb
Janine Lamb
Sharon Lamb
Tracey Lamb
Daniel Lambert
Elisabeth Lambert
Lauren Lambert
Nathalie Lambert
Paul Lambert
Stephen Lambert
Thierry Lambert
Walter Lambert
Luca Lambertini
Regis Lamberts
Robert Lamberts
Pia Lamberty
Mark Lambie
K.C. Lambirth
Rob Lambkin-Williams
Olivier Lambotte
Joeri Lambrecht
Marcel Lambrechts
Julia Lambret-Frotte
Petar Lambrev
Ariana Lambrides
Irene Lambrinoudaki
Dimitra Lambropoulou
Raphael Lamed
Adriano Lameira
Ryan Lamers
Rolf Lamerz
Daniel Lametti
Jay Ram Lamichhane
Saneer Lamichhane
Santosh Lamichhane
Guillaume Lamirault
Todd Lamitina
Tobias Lammel
Wim Lammers
Patrick Lammie
Dudley Lamming
Christoph Lämmler
Elise Lamont
Iain Lamont
Ruth Lamont
Scott Lamont
Susan Lamont
Lucie Lamontagne
R. Jason Lamontagne
Kristopher Lamore
Elizabeth Lamos
Claudine Lamoth
Frederic Lamoth
Mark Lamotte
Shona Lamoureaux
Ecosse Lamoureux
Francois Lamoureux
Jed Lampe
Paul Lampe
Angelika Lampert
Kirsten Lampi
Thomas Lampoltshammer
Vasileios Lampos
Maria Grazia Lampugnani
Amel Lamri
Rosa Lamuela-Raventós
Andre Lamurias
Pierre-Jean Lamy
Cheng-Che Lan
Chung-Yu Lan

Ganhui Lan
Keng-Hsin Lan
Li Lan
Weizhong Lan
Yeqing Lan
Zi-Jian Lan
Susan Lana
Michele Lanan
Miguel Lanaspa
Claudio Lanata
Gwendolyn Lancaster
Marcus Lance
Stacey Lance
Antonio Lancha
Tiziana Lanciano
Hovirag Lancioni
Kenneth Land
Mary Anne Land
Ulf Landegren
Rebecca Lander
Timothy Landers
Giora Landesberg
Erik Landfeldt
Stefan Landgraeber
Dominic Landgraf
Kathrin Landgraf
Alessandro Landi
Ana Marie Landin
Paolo Landini
Eric Landis
Bennett Landman
Neil Landman
Cecilie Landmark
Bruce Landon
Federico Landriel
John Landrum
Gregory Landry
Guillaume Landry
Steven Landry
David Landsberger
Anne-Marie Landtblom
Arthur Landy
Michael Landy
Alison Lane
Jeffrey Lane
Jerome Lane
Joseph Lane
Nancy Lane
Sean Lane
Steven Lane
Timothy Lane
Tristan Lane
Heinrich Lanfermann
Gerolamo Lanfranchi
Alex Lang
Andrew Lang
Carol Lang
Frieder Lang
Iain Lang
Irene Lang
James Lang
Jillian Lang
Joshua Lang
Moritz Lang
Muriel Lang
Sinead Langan
Chris Langdon
Christopher Langdon
Simon Langdon
Angela Lange
Anke Lange
Christian Lange
Dale Lange
Denise Lange
Jens Lange
Johannes Lange
Katharina Lange
Lene Lange
Miles Lange
Tobias Lange
Vinzenz Lange
Carl Langefeld
Geeske Langejans
Ralph Langen
Christopher Langendorf
Harald Langer
Max Langer
Nicolas Langer
Nelleke Langerak
Christelle Langevin
Helene Langevin
Dianne Langford
Paul Langford
Terry Langford
Alexander Langford-Smith
Peter Langguth
Morgan Langille
Kathryn Langin
Philip Langlais
Keith Langley
Ray Langley
Sean Langley

Marc-André Langlois
Ryan Langlois
Craig Langman
Cord Langner
Marek Langner
Soenke Langner
Sophie Langouet
Georg Langs
Lisa Langsetmo
Rosamund Langston
Christian Langton
Stephen Langton
Kate Langwig
Jessica Lanini
Ruth Lanius
David Lank
Kamran Lankarani
Jason Lanman
Gisela Lannig
Donald Lannin
Michael Lannoo
Marianna LaNoue
Van Lansingh
Beate Lanske
Daniele Lantagne
Fanny Lanternier
Paul Lantos
Olivier Lantz
Alessandra Lanubile
Sasha Lanyon
Gaetano Lanza
Giacomo Lanza
Rupert Lanzenberger
Giuseppe Lanzino
Giordano Lanzola
Lifeng Lao
Oscar Lao
Terence Tzu-Hsi Lao
Wongsa Laohasiriwong
Damya Laoui
Marie-Eve Lapalme
Nicolas Lapaque
Timothy LaPara
Scott LaPatra
Paul Lapchak
Syria Laperche
Suzanne Lapi
Moshe Lapidot
Oriol Lapiedra
Stephen Lapinsky
Michelle LaPlaca
Laurent Laplaze
Scott LaPoint
Paul LaPointe
Lauren Lapointe-Shaw
Thomas Lapole
Marinella Laport
Jimena Laporta
Rosaria Laporta
Martin Laporte
Rosamaria Lappano
Martha Lappas
Therese Lapperre
Otto Lappi
Robert LaPrade
James Lara
Lúcia Lara
Jason Larabee
Pablo Lara-Gonzalez
Matthew Laramie
Muriel Larauche
Oscar Lara-Velasco
Alfred Lardizabal
Philip Larese-Casanova
Matthew Large
Etienne Larger
Christine Largeron
Martina Lari
Kirill Larin
Vladimir Larionov
Markku Larjavaara
Bob Larkin
Celine Larkin
Joseph Larkin
Lisa Larkin
Joseph Larkin III
Anthony Larkum
Jan Larmann
S. Larney
Andre Laroche
Marc LaRochelle
Louise Larose
M.E. Larraga
Ricardo Larrainzar-Garijo
Hernan Larralde
Adriana Larregina
Daniel Larremore
Veronique Larreta-Garde
Antoine Larrieu
Pierre Larrivee
Gérald Larrouy-Maumus
Johan Larsbrink

Anders Larsen
Erik Larsen
Junilla Larsen
Mark Larsen
Melinda Larsen
Michael Larsen
P. Larsen
Peter Larsen
Randy Larsen
Sofus Larsen
Andrew Larson
Brendon Larson
Elaine Larson
Elysia Larson
Eric Larson
Heidi Larson
Julie Larson
Michael Larson
Nicholas Larson
Paul Larson
Peder Larson
Shawn Larson
Timothy Larson
Anders Larsson
Elin Larsson
Ellen Larsson
Elna-Marie Larsson
Jonas Larsson
Lotta Larsson
Maria E.H. Larsson
Mikael Larsson
Per-Göran Larsson
Margaret Lartey
Bruno Lartiges
Michelle LaRue
Lillebeth Larun
Tiziana Larussa
Roberta Lasagna
Laura Lasagni
Janine LaSalle
Cesar Lascala
Cecilia Laschi
Ion Lascurain
Victor Lasebikan
Lawrence Lash
Tamás Laskay
Judith Lasker
B.L. Laskin
Kristofer Lasko
Daniel Laskowitz
Marta Laskowski
Stephen Lasley
Amandine Lassalle
Francesco Lassandro
Andrew Lassar
William Lassek
Jesper Lassen
Gorka Lasso
Mariano Lastra
Elena Lasunskaia
Linda Lasure
Sarah Laszlo
Charu Lata
Matthieu Latapy
Maria Ujue Latasa
Giovanni Latella
Howard Laten
John Laterra
Jean-Paul Latge
Jeremy Latham
Kenzie Latham
Wyndham Lathem
Vinny Lather
Justin Lathia
Saeed I. Latif
Amel Latifi
Alice Latinne
Guillaume Latombe
Vito Latora
Ramon Latorre
Paulina Latos
Agnieszka Latosinska
Steven Latosinsky
Lawrence Latour
Augustin Latourte
Perle Latre de Late
Alban Latremoliere
David Lattanzi
Frank Lattanzio
Susan Latter
Jean-Baptiste Lattouff
Tatiana Latychevskaia
Vit Latzel
Yael Latzer
Aik Jiang Lau
Bonnie K. Lau
Calvin Ho-Fung Lau
Candy C. Y. Lau
Christopher Lau
Colleen Lau
Darryl Lau
Eric Lau

Esther Y.Y. Lau
Frank Lau
Gee Lau
Hang Lau
Kwok-Fai Lau
Laurie Lau
Loretta Lau
Melisa Lau
Phyllis Lau
Sue Mei Lau
Susanna K. P. Lau
Wayne Bond Lau
Wei Ling Lau
Chris Lauber
Christian Lauber
Agne Laucyte-Cibulskiene
Augustinus Laude
Vincent Laudenbach
Tsai-Ling Lauderdale
Vincent Laudet
Cheyenne Laue
Jeannet Lauenborg
Antje Lauer
Michael Lauer
Daniel Laufer
Miriam Laufer
Shlomi Laufer
Beatrice Lauga
Ane Laugen
Ruple Laughlin
Kathryn Laughon
Pascal Laugier
Charles Lauhon
Christina Laukaitis
Sandrine Launois
Juliano Laura
Martincich Laura
Raphaelle Laureau
Endres Lauren
Martin Laurence
Cato Laurencin
Kristin Laurens
Matthew Laurens
Alexandre Laurent
Arnaud Laurent
Catherine Laurent
Louise Laurent
Luca Laurenti
Sandra Laurentino
Douglas Laurents
Fulvio Lauretani
Matthew Lauretta
Gordon Laurie
Nikia Laurie
Anssi Laurila
Diana Laurillard
Kristin Laurin
Michel Laurin
Marco Laurino
Chiara Lauritano
Davide Lauro
Karsten Laursen
Lisbeth Laursen
Martin Laursen
Torben Laursen
Volker Lauschke
Ludwig Lausser
Christoffer Laustsen
Jeffrey Laut
Tim Lautenschlaeger
Matthias Lauth
Sylvie Lautru
Lena Lautscham
Dale Lauver
Hady Lauw
Peter Laux
Philippe Lavalle
Blas Lavandero
Sergio Lavandero
Patricia Lavand'homme
Mitra Lavasani
Johann Lavaud
Catherine Lavazec
Garry Laverty
Gary Laverty
Gareth Lavery
James Lavery
Richard Lavery
Enrico Lavezzo
Hugo Laviada
Alessandro Laviano
Arnon Lavie
Eric Lavigne
Pierre Lavigne
Gaute Lavik
Maurice Laville
Martin Lavin
Daniel Lavinsky
Luigi Laviola
Matthew LaVoie
Suzie Lavoie
Anne-Violette Lavoir

Peter Lavrentyev
Inna Lavrik
Harish Lavu
Adi Lavy
Barbi Law
Darin Law
Mansun Law
Yuen Kwan Law
Alfons Lawen
Mary Lawhon
Joshua Lawler
Patrick Lawler
Sean Lawler
Nicole M. Lawless DesJardins
Andrew Lawrence
Chip Lawrence
Christian Lawrence
Elizabeth Lawrence
John Lawrence
Melanie Lawrence
Michael Lawrence
Paulraj Lawrence
Shelley Lawrence
Susan Lawrence
John Lawrenson
Kate Lawrenson
Nathan Lawrentschuk
Brabara Lawrenz
Matthew Lawrenz
Edward Laws
Michael Laws
Becki Lawson
Brian Lawson
Julia Lawson
Michelle Lawson
Paul Lawson
Peter W. Lawson
Robert Lawton
Glenn Lawyer
Simon Lax
Sunil Laxman
Michael Laxy
Fides Lay
Suzanne Laychock
Brian Layden
Siddhartha Layek
Lester Layfield
Georg Layher
Laura Layland
Curtis Layton
Adina Lazar
Alexander Lazar
Nicole Lazar
Peter Lazarakis
Vassili Lazarev
Ljiljana Lazarevic
Asimina Lazaridou
Amparo Lazaro
Luisa Lázaro
Cathy J. Lazarus
Philip Lazarus
Sophie Lazarus
Cristina Lazcano
Helen Lazear
Žiga Laznik
Lambros Lazuras
Chiara Lazzari
Lorenza Lazzari
Vincent Lazzari
Brian Lazzaro
Douglas Lazzaro
Cheng Foh Le
Dao Khanh Long Le
Phuc Le
T.T. Yen Le
Thuc Le
Trung Le
Yun Le
Yoann Le Bagousse-Pinguet
Sophie Le Bellu
Anne-Pascale Le Berre
Sylvie Le Bomin
Lionel Le Bourhis
Yoann Le Breton
Florence Le Calvez-Kelm
Steven Le Cam
Yann Le Cunff
Joost le Feber
Christelle Le Foll
Grégoire Le Gal
Maude Le Gall
Sylvain Le Gall
Gaelle Le Goff
Ronan Le Goffic
Jacques Le Gouis
Fabienne Le Grand
Xavier Le Guével
Richard Le Heron
Isabelle Le Huërou-Luron
Mona Le Luyer
Olivier Le Maitre
Jean-Claude Le Mevel

Alain Le Moine
Catherine Le Moine
Vincent Le Moing
Hervé Le Moual
Olivier le Polain de Waroux
I. Le Poole
Jean Yves Le Reste
Virginie Le Rolle
Muriel Le Romancer
Carel Le Roux
Peter le Roux
Marion Le Texier
Florent Le Ven
Isabelle le Viol
Janice Lea
Amanda Leach
David Leach
Jennie Leach
Katie Leach
Matthew Leach
Jim Leafloor
Erin Leahey
Rehana Leak
Daniela Leal
Manuel Leal
Rodrigo Leal
Soraya C. M. Leal-Bertiol
Ernesto Cesar Leal-Junior
Ian Lean
Michael Lean
Roger Léandri
Vânia Aparecida Leandro-Merhi
Luigi Leanza
Sylvia Leão
Michael Leapman
Alberto Leardini
Christopher Learn
Mark Leary
Eric Leas
Hilary Lease
Francesca Leasi
Andrew Leask
David Lea-Smith
J. Leigh Leasure
Simon Leather
David Leavens
Michael Leaver
Gerard Leavey
Patrick Leavey
Joanna Leaviss
Camille Lebarbenchon
Simon Lebaron
Fiona LeBeau
Richard LeBeau
Aleksandra Lebedeva
Iryna Lebedyeva
Sarah Lebeis
Alexandre Lebel
Sarah Lebel
Bettina Leber
Limakatso Lebina
Normand Leblanc
Pascal Leblanc
Paul LeBlanc
Roger M. Leblanc
Arthur Leblois
Matthew Lebo
Fabien Leboeuf
Florent Lebon
Vincent Lebot
Nicolas Leboulanger
Muriel Lebourgeois
Jill Lebov
Adam Jon Lebowitz
Nathan LeBrasseur
Mario Lebrato
Carlito Lebrilla
Christine Lebrun
Ivo Lebrun
Maryse Lebrun
Renaud Lebrun
Fernando Lecanda
Yara Lechanteur
André Lechel
Claude Lechene
Shirley Lecher
James Lechleiter
P. Lechler
Michael Lecholop
Aron Lechtig
Jill Leckey
Grégoire Leclair
Estelle Leclerc
Nicole Leclerc
Alexandre Leclercq
Florence Leclercq
Sébastien Leclercq
Lucas Leclère
Chloe Lecoq
Magalie Lecourtois
Frédéric Lecouvet
Ramón Lecumberri

Valérie Lecureur
Farideh Ledari
Helmut Leder
Karin Leder
Eleanor Lederer
James Lederer
Ruben Ledesma
John Lednicky
Jonathan Ledoux
Kerry Ledoux
Alan Yueh-Luen Lee
Albert Lee
Aline Lee
Arier Chi Lun Lee
Ashlynn Lee
Baek-Seok Lee
Barry Lee
Benhur Lee
Benjamin Lee
Beth Lee
Brian Lee
Byong Lee
Byung Lee
Chang Lee
Chang Hee Lee
Changhan Lee
Chao-Yang Lee
Charles Tzu Chi Lee
Che-Hsin Lee
Cheng-Chi Lee
Cheng-Chung Lee
Cheng-I Lee
Cheuk Kwong Lee
Chiachi Bonnie Lee
Chia-Hwa Lee
Chia-Jung Lee
Chien-Kuo Lee
Chien-Nan Lee
Chien-Te Lee
Chih Lee
Chih-Hao Lee
Chih-Hsin Lee
Chih-Hung Lee
Chioun Lee
Chi-Ying Lee
Choong Hwan Lee
Choong-Eun Lee
Christina Lee
Christopher Lee
Chun Yi Lee
Dae Ho Lee
Danbi Lee
Daniel Lee
Darren Lee
Desy Lee
Do Yup Lee
Dong Ho Lee
Dong Wan Lee
Dongkuk Lee
Dong-Yup Lee
Douglas Lee
Duk-Hee Lee
Eng Hin Lee
Eun Kyung Lee
Eun-Jig Lee
Gabsang Lee
Geun-Shik Lee
Hae-Jeung Lee
Harry Lee
Hey-Kyoung Lee
Ho Joung Lee
Ho Seong Lee
Hon Lee
Howon Lee
Hsiang-Chieh Lee
Hsuan-Shu Lee
Hyang Woon Lee
Hye Seung Lee
Hye Won Lee
Hyung Chul Lee
Hyunhwa Lee
I-Cheng Lee
I-Chi Lee
Ik Jae Lee
In Su Lee
Iris Lee
Jae Cheol Lee
Jae Gil Lee
Jae Wook Lee
Jae-Hyung Lee
Jae-Kyung Lee
Jaeseong Lee
James Lee
Janet Lee
Janice Lee
Jay Lee
Jean Lee
Jeannette Lee
Jeffery Lee
Jeong-Hoon Lee
Jeonghun Lee
Jia-Jung Lee

Jih-Hsiang Lee
Jin-Ching Lee
Jinseok Lee
Jinwoo Lee
John Lee
Jong-Koo Lee
Jongmin Lee
Jongsoon Lee
Joo Yong Lee
Joo Young Lee
Joon Lee
Joo-Yong Lee
Joy Lee
Julian Lee
Jun Lee
Jun Hee Lee
Jung-Hoon Lee
Jung-Kul Lee
Jungkwan Lee
Jungnam Lee
Junhee Lee
Junsoo Lee
Kang-Yun Lee
Keun Hwa Lee Lee
Kevin Lee
Kheng Hock Lee
Kichun Lee
Kiho Lee
Kuei-Chuan Lee
Kwang Ho Lee
Kwang Pum Lee
Kwangwon Lee
Kwonmoo Lee
Kyongbum Lee
Kyu-Ho Lee
Kyung-Min Lee
Kyungwon Lee
Li-Ang Lee
Lik Chuan Lee
Marianne Lee
Mei-Hsien Lee
Mei-Hsuan Lee
Mei-Yueh Lee
Mi Ji Lee
Michael Lee
Miin-Huey Lee
Mike Lee
Min Geol Lee
Min Goo Lee
Ming-Been Lee
Ming-Ching Lee
Ming-Jen Lee
Ming-Shinn Lee
Ming-Shyue Lee
Minjin Lee
Moo Hyun Lee
Myeongwoo Lee
Myung-Shin Lee
Natuschka Lee
Ni-Chung Lee
Nikki Lee
Nora Lee
Oscar Lee
P. Lee
Patrick Lee
Paul Lee
Pei-Chang Lee
Pei-Lin Lee
Peter Lee
Phyllis Lee
Po-Shun Lee
R.K. Lee
Randall Lee
Robert Lee
Robin Lee
S. Lee
Sa Ra Lee
Samuel Lee
Sandra Lee
Sang Lee
Sang Eun Lee
Sang Hyun Lee
Sang Jin Lee
Sanghee Lee
Sangheun Lee
Sangho Lee
Sanghoon Lee
Sangkyu Lee
Sang-Myeong Lee
Sang-Rae Lee
Sean Lee
Seok-Yong Lee
Seong-Whan Lee
Seung Duk Lee
Seung Heon Lee
Seung-Ju Lee
Shaun Lee
Shin Yin Lee
Shyh-Jye Lee
So Hee Lee
Soo Lee
Soo Chan Lee

Soomin Lee
Spike Lee
Su Seong Lee
Sue Lee
Sukhyang Lee
Sun Hee Lee
Sung Chul Lee
Sung Kuk Lee
Sung-Ho Lee
Sung-Jae Lee
Sungkyu Lee
Sun-Kyeong Lee
Sylvia Lee
T. H. Lee
Tack Lee
Tae Lee
Taek Lee
Teng-Yu Lee
Theresa Lee
Tian-Ren Lee
Tse-Min Lee
Tsong-Hai Lee
Tsung-Han Lee
Tsung-Ming Lee
Tzong-Shyuan Lee
Vincent Lee
W. Lee
Wai-Nang Paul Lee
Wei-Ju Lee
Wen-Chung Lee
Wendy Lee
Wen-Ying Lee
Weon-Young Lee
Wing-Kee Lee
Won Ki Lee
Wonchoel Lee
Wonik Lee
Won-Joon Lee
Wooin Lee
Woo-Kyun Lee
Y. Lee
Yi Chen Lee
Yi-Chia Lee
Yi-Chung Lee
Yong Lee
Yong-Hwan Lee
Yoo Kyung Lee
Yoonkwang Lee
Young Ho Lee
Yueh Lee
Yuong-Nam Lee
Yu-Shang Lee
Zarraz Lee
Edwin Leeansyah
Christine Leeb
Tosso Leeb
Michelle Leech
Stephen Leeder
Simon Leedham
Daniel Leeds
Jong Han Leem
Lawrence Leeman
William Leenders
Celine Leenen
Frans Leenen
Alon Leeor
Elisabeth Leermakers
Jessica Leers
Alexander Lees
David Lees
Rosemary Lees
Julia Lee-Thorp
Christiaan Leeuwenburgh
S. Leeuwenburgh
Chen Lee-Wei
Alice Lefebvre
Francine Lefebvre
Jérémie Lefebvre
Jonas Lefevere
Carmen Lefevre
Jo-Anne LeFevre
Sarah LeFevre
Alexander Leff
Todd Leff
Hyam Leffert
Lisa Leffert
Jonatan Leffler
Ivan Lefkovits
Marie-Caroline Lefort
Avigdor Leftin
Cecile Legallais
Sandra Legan
Cristine Legare
Frédéric Legendre
Bertrand Leger
James Legg
Francesco Legge
Kevin Legge
Justin Legleiter
Patricia Legler
Tobias Legler
Benoit LeGoff

Jean-Luc Legras
Stephane Legriel
Alexander Legwegoh
V'yacheslav Lehen'kyi
Jean-Yves Lehesrand
Tanya Lehky
Joel Lehman
Jon Lehman
Michael Lehman
Vance Lehman
Birthe Lehmann
Edouard Lehmann
Helmar C Lehmann
Hugo Lehmann
Michael Lehmann
Roger Lehmann
Sylvain Lehmann
Wolfgang Lehmann
Klaus Lehmann-Horn
Markus Lehmkuhl
Paul Lehner
Linn Sophia Lehnert
Martin Lehnert
Erik Lehnhoff
Edgar Lehr
Stefan Lehr
Laura Lehtinen
Samuli Lehtonen
Sanna Lehtonen
Mikko Lehtovirta
Bo Lei
Chao-Liang Lei
Chengwei Lei
Chu-Zhao Lei
Guang-Hua Lei
Hao Lei
Hong Lei
Hongxing Lei
Kai Lei
Minggang Lei
Peng Lei
Shufeng Lei
Xia Lei
Xiaoying Lei
Xin Gen Lei
Xinglin Lei
Yu Lei
Zhen Lei
Zhenmin Lei
Zhigang Lei
Margarita Leib
Raz Leib
Eric Leibert
Jessica Leibler
Elizabeth Leibold
Eugene Leibovitz
Arleen Leibowitz
Gregor Leibundgut
James Leichter
Andrew Leidner
Maren Leifheit-Nestler
Andreas Leiherer
Ralph Leijenaar
Patty Leijten
George Leikauf
Peter Leimgruber
Ed Lein
Trese Leinders-Zufall
Marie Leiner
Olof Leinhard
Ville Leinonen
Enrico Leipold
Claudia Leiros
Jonathan Leis
Tanya Leise
Malcolm Leissring
Ana Lúcia Leitão
Jorge Leitão
Victoria Leitch
Marcio Leite
Maria Leite
Maria Isabel Leite
Nuno Leite
Lucas Leite Cunha
Ed Leiter
Jürgen Leitner
Stefan Leitner
Thomas Leitner
Andrea Leiva
Tomás Lejarraga
Fabrice Lejeune
Veerle Lejon
Michel Lejoyeux
Evgeny Lekchnov
Malgorzata Lekka
Marilena Lekka
Arne Lekven
Pushkar Lele
Tanmay Lele
Frederik Leliaert
Irene Lelieveld
Jean-Daniel Lelievre

Sophie A. Lelievre
Chiara Lelli
Sophie Lelorain
Julie Leloup
Benjamin Lelouvier
A. Titia Lely
Gareth M. Lema
Valerie Lemaine
Christophe Lemaire
Edward D. Lemaire
Patrick Lemaire
Jean-François Lemaitre
Dominick Lemas
Isabelle Lemasson
Matthew Lemay
Giuseppe Lembo
Ludovic Lemée
Tagrid Lemenager
Marieke Lemiengre
Isabelle Lemieux
Joseph Lemire
Michael Lemke
Ney Lemke
Kevin Lemley
Eshetu Lemma
Joelle Lemmen
Robin Lemmens
Trudo Lemmens
Gunnar Lemmer
Florian Lemmerich
Frederic Lemoine
Sandrine Lemoine
Gerald Lemole
Roberto Lemoli
Jim Lemon
Francois Lemonnier
Carlos Lemos
Eliana Lemos
Pedro Lemos
Robert LeMoyne
Cynthia Lenaerts
Brigita Lenarcic
Rebekka Lencer
Heather Lench
Chuan Leng
Gareth Leng
Huijie Leng
Qibin Leng
Roger Leng
Shuguang Leng
Xiangjun Leng
Yue Leng
Etienne Lengline
Michael Lenhard
Audrey Lenhart
Gustavo Lenis
Balint Lenkei
Jenny Lenkowski
Cleridy Lennert-Cody
Alex Lennon
Frances Lennon
Rachel Lennon
Jeffrey Lennox
Matthieu Lenoir
Maxime Lenormand
Marc Lensink
Alex Lentsch
Hartmut Lentz
Michael Lentze
Jack Lenz
Mark Lenz
Peter Lenz
Petra Lenz
Sigurd Lenzen
Jack Leo
Lisa Leon
Oscar Leon
Paul Leon
Anthony Leonard
Christopher Leonard
Hayley Leonard
Jennifer Leonard
Laurence Leonard
Paul Leonard
Warren Leonard
Stefano Leonardi
Jo Leonardi-Bee
Margareta Leonardsson-Hellgren
Gabriella Leonarduzzi
Alfonso León-Del-Río
Alessandro Leone
Umberto Leone Roberti Maggiore
Christine Leong
Darryl Leong
Fong Yew Leong
Kelvin Leong
Lex Leong
Michael Leong
Susanna Leong
Howard Leong-Poi
Ralf Leonhardt
Carolina Leoni

Lara Leoni
Isabel Leonor
Vladimir Leon-Salazar
Marco Leonti
Georgios Leontidis
Bruce Leopold
Christine Leopold
Jane Leopold
Mardik Leopold
Dario Leosco
Marco Leotta
Jean-François Lepage
Martin Lepage
Malgorzata Lepczynska
Antonio Lepedda
Bernard Lepetit
Konstantin Lepikhov
Angelo Lepore
Domenico Lepore
Benedetto Lepori
Andrew Lepp
Jenni Leppanen
John Leppert
Phyllis Leppert
Fabien Leprieur
Corinne Leprince
Olivier Leprince
Anais Leproux
Thierry Lequerre
Emmanuelle Leray
Alexander Lerch
Jessica Lerch
Holger Lerche
Trond Leren
Marja-Kristiina Lerkkanen
Maria Lerm
Amir Lerman
Lilach Lerman
Nancy Lerner
Seth Lerner
Ulf Lerner
Anne-Marie Leroi
Iracema Leroi
Tanya LeRoith
Nicolas Lerolle
Olivier Leroux
Carri LeRoy
O. Leroy
Sandrine Leroy
Amnon Lers
Noppon Lertwattanasakul
Unax Lertxundi
Florian Lesage
Claude LeSaux
David Lesbarrères
Roxana Lescano
Halard Lescinsky
Mylene Lesenechal
Ulf Leser
Alan Lesgold
Eyal Leshem
Olivier Lesieur
Clémence Lesimple
Katarzyna Leskinen
Alasdair Leslie
Elizabeth Leslie
Ken Leslie
Wieslawa Lesniak
Philippe Lesnik
Saskia Lesnik Oberstein
Hervé Lesot
Christopher Lessard
Samuel Lessard
Laurence Lessard-Phillips
Heidi M.B. Lesscher
Alan Lesse
Richard Lessells
Glenn Lesser
Iris Lesser
Daniel Lessner
Lawrence Lessner
Susan Lessner
Henry Lester
Mark Lester
Sarah Lester
Emilio Letang
Peter Letcher
Juan-Carlos Letelier
Elisabeth Letellier
Jean-Jacques Letesson
Craig Leth-Steensen
Mike Letnic
Gaetano Leto
Yves Letourneur
Anthea Letsou
Christophe Lett
Philippe Letteron
Guillaume Lettre
Jiann Horng Leu
Shao-Yuan Leu
Nicolas Leuenberger
Pedro Leunda

Angela Leung
Anskar Leung
Carson K. Leung
Hoi-Chung Leung
Howard Leung
Jacqueline Leung
Janice Leung
Janni Leung
Johahn Leung
June Leung
Justin Leung
Lai Yee Leung
Lawrence Leung
Marcus Leung
Mark Leung
Ming-Ying Leung
Nicki Leung
P.C. Leung
Patrick Leung
Sharon Leung
Susan Leung
Tin Leung
Ting Fan Leung
Tommy Leung
Victor Leung
Wai Leung
Yuet-Kin Leung
Baptiste Leurent
Florian Leuschner
Hartmut Leuthold
Heather Leutwyler
Henri Leuvenink
Vincenzo Leuzzi
Sima Lev
Ernesto Leva
Kateryna Levada
Katherine E. Levan
Sonja Levanat
Erez Levanon
Beth Levant
Stephen Levas
Eylem Levelt
Alytia Levendosky
David Levens
Vasiliki Leventakou
Vicky Leventakou
Jean Leveque
Nicolas Leveque
Friedhelm Leverkus
Eric Levesque
Jean Pierre Levesque
Maxime Levesque
Mitch Levesque
Amnon Levi
Benjamin Levi
Edi Levi
José Levi
Marinella Levi
Sonia Levi
Taal Levi
Valeria Levi
Yossi Levi-Belz
Jorge Levican
Scott Levick
Yuri Levik
Adeera Levin
Barry Levin
Carina Levin
Carol Levin
Harvey Levin
Lisa Levin
Oron Levin
Petra Levin
Rachel Levin
Aaron Levine
Adam Levine
Benjamin Levine
Brenna Levine
Brett Levine
Edward Levine
Jon Levine
Lisa D. Levine
Martin Levine
Steven Levine
Todd Levine
Randall Levings
David Levinson
Jeffrey Levinton
Brooke Levis
Francesca Levi-Schaffer
Judy Levison
Zoe Leviston
Irena Levitan
Orly Levitan
David Levitsky
Bodo Levkau
Zlatko Levkov
Zoran Levnajic
Anna-Liisa Levonen
Liat Levontin
Jacques Levraut
Florence Levréro

Luca Levrini
Elena Levtchenko
Ariel Levy
Avram Levy
Becca Levy
Benjamin Levy
Bruce Levy
Corinne Levy
David Levy
Doron Levy
Estrella Levy
Gary Levy
Marc Levy
Michael Levy
Mitchell Levy
Nadav Levy
Oren Levy
Philip Levy
Sary Levy
Steven Levy
S. Levy-Tzedek
Jie-Bin Lew
Michael Lew
Eric Lewallen
Eva Lewandowski
Jorg Lewandowski
Gregory Lewbart
Pawel Lewek
Shawn Lewenza
Susanna Lewerin
Astrid Lewin
Sarah Lewington
Amanda Lewis
Bryan Lewis
Cara Lewis
David Lewis
Janina Lewis
Joanna Lewis
Joshua Lewis
Kathleen Lewis
Kemper Lewis
Lawrence Lewis
Lynn Lewis
Margaret Lewis
Martin Lewis
Mary Catherine Lewis
Michael Lewis
Rohan Lewis
Sheena Lewis
Simon Lewis
Valerae Lewis
Warren Lewis
Zachary Lewis
Zachery Lewis
Grant Lewison
Rebecca Lewison
Maxim Lewkowski
Joseph Lewnard
Joanne Lewohl
Alexandra Ley
Benedikt Ley
Ruth Ley
Serej Ley
Sylvia Ley
Luc Leybaert
Loet Leydesdorff
Ciara Leydon
Werner Leyh
Dieter Leyk
Stefan Leyk
Alastair Leyland
David Leys
Eugene Leys
Misti Leyva
Victor Leyva-Grado
Juan Leza
Claudio Lezama-Davila
Frank Lezoualc'h
Thibault Lhermusier
Nicolas L'Heureux
Monique L'Hoir
Lenka Lhotska
Karl Lhotta
Arnaud L'Huillier
Aihua Li
Ai-Jun Li
An Li
Anan Li
Ang Li
Angsheng Li
Annan Li
An-Wei Li
Bai-Yan Li
Baocai Li
Baojuan Li
Baolei Li
Baolin Li
Benyi Li
Bichun Li
Bin Li
Bing Li
Bingjin Li

Bingshan Li
Bingxue Li
Bingyun Li
Bo Li
Bob Li
Chang Xian Li
Changchun Li
Changwei Li
Changzheng Li
Chao Li
Chaoyang Li
Chen Li
Chenghua Li
Cheng-Qing Li
Chengtao Li
Cheng-Yun Li
Chia-Cheng Li
Chia-Lin Li
Chia-Yang Li
Chi-Yuan Li
Chunfeng Li
Chung-Pin Li
Chunhao Li
Chunhua Li
Chunming Li
Chunyan Li
Cun Li
Da Li
Daijiang Li
Daiqin Li
Danielle Li
Danni Li
Dapeng Li
Dawei Li
Dayong Li
De-Hua Li
Dejun Li
Dezhi Li
De-Zhu Li
Dong Li
Dongmei Li
Dongsheng Li
DongYe Li
E. Li
Ellen Li
Erchao Li
Fa-Jun Li
Fan Li
Fei Li
Feng Li
Fengna Li
Fengri Li
Fuhong Li
Gang Li
Ge Li
Gen Li
Gordon Li
Guang Li
Guangbin Li
Guangye Li
Guibing Li
GuoDong Li
Guohui Li
Guojun Li
Guoping Li
Guoqing Li
Guorong Li
Hai Li
Haichang Li
Haihong Li
Haipeng Li
Haitao Li
Haiyun Li
Hang Wun Raymond Li
Haobo Li
Hao-Sen Li
He Li
Henan Li
Heng Li
Henghong Li
Ho Cheung William Li
Hong Li
Hong-Fu Li
Hongge Li
Hongjiao Li
Hongju Li
Honglang Li
Hong-Li Li
Honglian Li
Hongliang Li
Hong-Sheng Li
Hong-Tao Li
Hong-Ye Li
Hongyi Li
Hongzhe Li
Hu Li
Hua Li
Huabin Li
Huadl Li
Huaqing Li
Hui Li
Huiguang Li

Huihua Li
Hui-Jie Li
Huirong Li
Huixin Li
Huiying Li
James J. Li
Jia Li
Jia-Da Li
Jiale Li
Jiali Li
Jiamei Li
Jian Li
Jianfeng Li
Jianhong Li
Jiani Li
Jian-Jun Li
Jian-Ming Li
Jianxiong Li
Jianyong Li
Jiaqi Li
Jiarui Li
Jia-Tang Li
Jiawen Li
Jiaxu Li
Jie Li
Jih-Heng Li
Jiliang Li
Jin Li
Jinchen Li
Jing Li
Jing Jing Li
Jinghua Li
Jingyi Jessica Li
Jinhua Li
Jinjun Li
Jinpeng Li
Jinping Li
Jinrong Li
Jisu Li
Juan Li
Judy (Yaqin) Li
Jun Li
Junming Li
Junsheng Li
Kaccie Li
Kaiming Li
Ke Li
Laifang Li
Lan Li
Lanjuan Li
Lei Li
Li Li
Liang Li
Lianhui Li
Lihua Li
Li-Hua Li
Lihui Li
Lijia Li
Liming Li
Lin Li
Lin-Feng Li
Ling-Hui Li
Ling-Jun Li
Linyan Li
Liping Li
Long Li
Lulin Li
Mai Suan Li
Man-Wah Li
Maoteng Li
Mei Li
Meng Li
Meng-Hua Li
Menghui Li
Mengjiao Li
Miaoxin Li
Miao-Xin Li
Min Li
Min-Dian Li
Ming Li
Mingguang Li
Ming-He Li
Minghua Li
Mingshun Li
Ming-Tao Li
Mingxun Li
Mingyu Li
Mingzhou Li
Mulin Li
Muwang Li
Na Li
Nan Li
Nanlin Li
Ning Li
Norman Li
P. Andy Li
Paul Li
Pei-Tzu Li
Peng Li
Phillip Li
Qi Li
Qian Li

Qiang Li
Qiao Li
Qinfei Li
Qingguo Li
Qinglei Li
Qingsheng Li
Qiu Li
Qiuhong Li
Qiutang Li
Qiwei Li
Quan Li
Quanmin Li
Ran Li
Rende Li
Renfeng Li
Rong Li
Rongfeng Li
Rui Li
Ruijiang Li
Ruiying Li
Runsheng Li
Ruosha Li
Sai-Ping Li
Sean (Xuguang) Li
Shanshan Li
Shao Li
Shaokun Li
Shaoying Li
Shau-Hsuan Li
Sheng Li
Shengnan Li
Shi-Fang Li
Shihua Li
Shi-Ming Li
Shisheng Li
Shouli Li
Shu Li
Shuo Li
Shuxin Li
Shuzhao Li
Song Li
Taisheng Li
Tangliang Li
Tao Li
Tiangang Li
Tianjing Li
Tianqing Li
Tiejun Li
Ting Li
Tingqiang Li
Tongtong Li
Wei Li
Weigang Li
Weikai Li
Wei-Wei Li
Weizhong Li
Wen Li
Wenhui Li
Wenjun Li
Wentao Li
Wenyuan Li
Wenzhi Li
Wu Li
Xia Li
Xiang Li
Xiang-An Li
XiangRui Li
Xiangyang Li
Xiao Li
Xiaobai Li
Xiao-Dan Li
Xiaohong Li
Xiaojiang Li
Xiaojuan Li
Xiaokun Li
Xiaoming Li
Xiaonan Li
Xiao-Nan Li
Xiaopeng Li
Xiaoping Li
Xiaoqiang Li
Xiaorong Li
Xiaowei Li
Xiaoyan Li
Xiao-Yu Li
Xin Li
Xinde Li
Xine Li
Xingfeng Li
Xingnan Li
Xingshan Li
Xin-Jian Li
Xinjing Li
Xinxu Li
Xinying Li
Xinyuan Li
Xinzheng Li
Xiong Li
XiuJun Li
Xu Li
Xuan Li
Xue-Bao Li

Xuechao Li
Xue-Dong Li
Xuejun Li
Xue-Jun Li
Xuexian Li
Xuping Li
Xuqi Li
Y.F. Li
Yan Li
Yan Chun Li
Yang Li
Yaning Li
Yanqing Li
Yanzhang Li
Yao Li
Yaoming Li
Ye Li
Ye Lennon Li
Yi Li
Yin Li
Yinchuan Li
Ying Li
Ying Ying Li
Yingkui Li
Yingyue Li
Yinxin Li
Yi-Ping Li
Yiwen Li
Yong Li
Yongfang Li
Yonghua Li
Yongjun Li
Yongqing Li
Yongxin Li
Yuan Li
Yuanfa Li
Yuanyou Li
Yuanyuan Li
Yuchang Li
Yue Li
Yuelin Li
Yue-Zhong Li
Yuhuan Li
Yuju Li
Yulin Li
Yuling Li
Yumei Li
Yun Li
Yunjie Li
Yunqi Li
Yupeng Li
Yurong Li
Yuxin Li
Yuxing Li
Yuzhan Li
Yvonne Li
Zechao Li
Zhang Li
Zhanguo Li
Zhanjun Li
Zhaohui Li
Zhaojun Li
Zhaoshen Li
Zhao-Shen Li
Zhe Li
Zhen Li
Zheng Li
Zhengguo Li
Zhengke Li
Zhenqing Li
Zhi Li
Zhibin Li
Zhicheng Li
Zhigang Li
Zhiguo Li
Zhihao Li
Zhihua Li
Zhiming Li
Zhi-Ming Li
Zhineng Li
Zhipeng Li
Zhiqiang Li
Zhiyong Li
Zhongpei Li
Zhongqi Li
Zhong-Qian Li
Zhongwu Li
Zhongyi Li
Zhoujun Li
Zhuyun Li
Zibo Li
Zichao Li
Zongjin Li
Zongyun Li
Bertrand Liagre
Christine Lian
Chunlan Lian
Jane Lian
Jie Lian
Lu-Yun Lian
Qizhou Lian
C. Liang

Carol Liang
Changhong Liang
Chih-Ming Liang
Decui Liang
Deguang Liang
Eryuan Liang
Fenghe Liang
Gangning Liang
Guang Liang
Guanxiang Liang
Hai Liang
Hao Liang
Houjie Liang
Hualou Liang
Huey-Wen Liang
Hung Hua Liang
Jialiang Liang
Jie Liang
Jingjing Liang
Juan Boo Liang
Kelly Liang
Lingyi Liang
Matthew Liang
Mingxiang Liang
Nu-Chu Liang
Pei Liang
Qin Liang
Qiu-Xia Liang
Weili Liang
Weiqian Liang
Xi Liang
Xiao Liang
Xiaohui Liang
Xibin Liang
Xihui Liang
Xinchen Liang
Xing-Jie Liang
Xiubin Liang
Xuanwei Liang
Xufang Liang
Yajie Liang
Yajun Liang
Yantao Liang
Yong Liang
Yu Liang
Yuan Liang
Yuanbo Liang
Yuzhen Liang
Zhenchang Liang
Zheng-Lun Liang
Zheng-Wei Liang
Zhiqing Liang
M.A. Liangli
Georgios Lianos
Alexandra Lianou
Chen Liao
Chun-Hou Liao
D. Joshua Liao
Eric Liao
Felix Haifeng Liao
Fuyuan Liao
Gui-Qing Liao
Guo-Shiou Liao
Hong Liao
Hung-Chang Liao
Jie Liao
Joseph Liao
Jyh-Fei Liao
Katherine Liao
L.L. Liao
Li Liao
Lili Liao
Ming Liao
Pei-Chun Liao
Ping Liao
Riqiang Liao
Rongzhen Liao
Shih-Cheng Liao
Shunyao Liao
Sumei Liao
T.Y. Liao
Wei Liao
Weijia Liao
Wenbo Liao
Xiaoyun Liao
Xing Liao
Yalin Liao
Yaping Joyce Liao
Yi Liao
Yi-Chun Liao
You-Di Liao
Yu-Cai Liao
Yu-Chien Liao
Irfana Liaqat
S.Y. Liaw
Yung-Po Liaw
Marc Libault
Lisa Libby
Stephen Libby
Gary Libecap
Camilo Libedinsky
Giulia Liberati

Krzysztof Liberek
Frederic Libersat
Claude Libert
William Liberti III
Melissa Libertus
Alexander Liberzon
Alexandre Liborio
Massimo Libra
Silvia Libro
Diana Libuda
Hongmei Li-Byarlay
Vincent Licata
Orazio Licciardello
Paul Licciardi
Brigitte Licht
Jonathan Licht
Michael Lichten
Michael Lichtenauer
Erik Lichtenberg
Kenneth Lichtenstein
Wandy Lichtenthal
Miriam Lichtner
M.A. Lictenauer
Joshua Liddy
Kerstin Lidén
Lena Lidfors
Stefan Liebau
Andre Lieber
Diana Lieber
Justin Lieber
Michael Lieber
Richard Lieber
Daniel Lieberman
Debra Lieberman
Harris Lieberman
Jay Lieberman
Paul M Lieberman
Phillip Lieberman
Wilfred Lieberthal
Jürgen Liebig
Andrea Liebl
Daniel Liebl
Hans Liebl
Elisabeth Liebler-Tenorio
Edward Liechty
Pablo Liedo
Carole Liedtke
Michelle Liedtke
Wolfgang Liedtke
Justin Liefer
Victor Lieffers
Jean-Francois Liegeois
Angela Liegey Dougall
Robert Liem
Edith Liemburg
Eric Lien
Judit Lienert
Sascha Liepelt
Inga Liepelt-Scarfone
Marc Liesa
John Lieske
Arthur Liesz
Daniel Lietha
Judith Lieu
Deah Lieurance
Oi Wah Liew
Woei Chang Liew
Yi Jin Liew
Zeyan Liew
Alan Lifson
Libby Liggins
Claudio Liguori
Giovanna Liguori
Jaan Liira
Diego Lijavetzky
Walter Lilenbaum
Brandi Liles
Mikael Lilja
Markus Lill
Roland Lill
Troels Lillebaeck
Catherine Lilley
Martin Lilley
Cathrine Lillo
Concepcion Lillo
Triantafillos Liloglou
Albert Lim
Boon L. Lim
Bora Lim
Chunghun Lim
Gui Yin Grace Lim
Han Lim
Hui Fang Lim
Hyuk Lim
Ilhan Lim
Jae-Yol Lim
Jihyeon Lim
Jongil Lim
Jong-Seok Lim
Joon Lim
Julian Lim
Kah-Leong Lim

Kian Lim
Kian Meng Lim
Kihong Lim
Kuang Kuay Lim
Lina Lim
Lipyeow Lim
Norman Lim
Phaik-Eem Lim
Rebecca Lim
Renly Lim
Robert Lim
Sungkyun Lim
Tit Meng Lim
Un Taek Lim
Unhee Lim
Wai Lim
Yen Ying Lim
Yiheng Lim
Yoongho Lim
Young Woon Lim
Young-Suk Lim
Carla Lima
Joni Lima
Kledoaldo Lima
Lidia Lima
Marcelo de Oliveira Lima
Marcos Lima
Mauricio Lima
Renata Lima
Rui Lima
Vilma Lima
Walter de Paula Lima
Paulo Lima Gomes
Gipsi Lima-Mendez
Thomas Liman
Matheus Lima-Ribeiro
Rupali Limaye
Jacqueline K. Limberg
Kirsten Limesand
Wu Limin
Thawornchai Limjindaporn
B. N. Limketkai
Direk Limmathurotsakul
Charles Limoli
Agenor Limon
Jean Limongi
Sophie Limou
Maarten Limper
Savitree Limtong
Aifen Lin
Chen Lin
Cheng-Li Lin
Cheng-Wen Lin
Chen-Yong Lin
Chi Lin
Chia-Hua Lin
Chien-Chi Lin
Chien-Liang Lin
Chih-Che Lin
Chih-Lin Lin
Chih-Peng Lin
Chi-Hung Lin
Chin-Hsien Lin
Chin-Kai Lin
Chin-Tarng Lin
Chin-Yo Lin
Chuan Lin
Chun Chieh Lin
Chung-Ping Lin
Chung-Yen Lin
Chung-Ying Lin
ChunLiang Lin
Chun-Liang Lin
Chun-Mao Lin
Chun-Pin Lin
Chunye Lin
Chun-Yen Lin
Chun-Yu Lin
Deshu Lin
Fan-Xue Lin
Feng-Yen Lin
Fuchun Lin
Fuhua Lin
Ge Lin
Geng Lin
Guo-Le Lin
Han-You Lin
Hao Lin
Harrison W. Lin
Honghuang Lin
Honghui Lin
Hsien-Chang Lin
Hsi-Hsun Lin
Hsin-Ching Lin
Hsing-Juh Lin
Hung-Du Lin
Hung-Yun Lin
Jack Lin
Jen-Jia Lin
Jiang-Jen Lin
Jiaxing Lin
Jieru Lin

Jinxiu Lin
Jo-Fu Lin
Jules Lin
Jun Lin
Kangyu Lin
Kun-Jhih Lin
Lawrence Lin
Lei Lin
Liang-Ching Lin
Liang-Tzung Lin
Li-Mei Lin
Lin Lin
Liu Lin
Mao Lin
Mei-Hui Lin
Meng Lin
Ming-Chih Lin
Mingli Lin
Mingqun Lin
Ming-Tsan Lin
Ming-Tsung Lin
Ming-Wei Lin
Ming-Yen Lin
Nan Lin
Nengming Lin
Nina Lin
Ning Lin
Pan Lin
Patrick Lin
Peter Lin
Ping-Ting Lin
Qiang Lin
Qinhua Lin
Ruo-Kai Lin
Shih-Hua Lin
Shin Lin
Shinn-Zong Lin
Shu Lin
Shu-Chun Lin
Shuei-Liong Lin
Shu-Fu Lin
Shu-Min Lin
Shyh-Hsiang Lin
Si-Min Lin
Sue-Hwa Lin
Suewei Lin
Susan Lin
Sze-Kwan Lin
Tao Lin
Tiao-Yin Lin
Tong-Jun Lin
Tsai-Lien Lin
Tsai-Yu Lin
Tsan-Piao Lin
Tsung-Hsien Lin
Tzu-Hao Lin
Tzung-Jin Lin
Valerie Lin
Wan-Wan Lin
Wan-Yu Lin
Wei-Ning Lin
Wei-Yang Lin
Wen-Chang Lin
Wen-Yuan Lin
Xiaochen Lin
Xiaojing Lin
Xin Lin
Xiongjie Lin
Xubo Lin
Y. C. Lin
Yan Lin
Ya-Wen Lin
Yen-Chang Lin
Yeong Shin Lin
Yi Pu Lin
Yi-Ching Lin
Yi-Chung Lin
Yi-Ling Lin
Yi-Mei Lin
Ying-Chi Lin
Ying-Hung Lin
Yingsong Lin
Ying-Ting Lin
Yi-Ting Lin
Yi-Tsen Lin
Yi-Tsung Lin
Yongjun Lin
You-Min Lin
Yowyu Lin
Yuan-Feng Lin
Yucong Lin
Yueweu Lin
Yu-Hsuan Lin
Yu-Kai Lin
Yu-Shan Lin
Zhao Lin
Zhifen Lin
Zhi-Qing Lin
Zhiqun Lin
Zhiyong Lin
Zhongxu Lin
Zibei Lin

Zu-Yau Lin
Benedetto Linaldeddu
Christine Linard
Maria Linares
Marco Linari
Olle Lind
Anders Lindahl
Paul Lindahl
Per Lindahl
Tomas Lindahl
R. Lindahl-Jacobsen
Eva Lindberg
Greger Lindberg
Iris Lindberg
Påvel Lindberg
Søren Lindberg
Kim Lindblade
Annika Lindblom
Joni Lindbohm
Klaus Linde
Karl-Fredrik Lindegaard
Annukka Lindell
Catherine Lindell
Dennis Lindell
Amanda Lindeman
Marjaana Lindeman
Neal Lindeman
Ulrich Lindemann
Petra Lindemann-Matthies
Dan Linden
Jérôme Linden
Rafael Linden
Thomas Lindenstrøm
Thies Lindenthal
Ulrike Lindequist
Joshua Linder
Maria Linder
Nicolas Linder
Stefan Linder
Stig Linder
Michael Linderman
Jutta Lindert
Steven Lindfield
Björn Lindgren
Helena Lindgren
Jussi T. Lindgren
S.R. Lindheim
Anna Lindholm
Bengt Lindholm
Dan Lindholm
Torun Lindholm
Amanda Lindholm-Perry
Richard Lindley
Steven Lindley
Angelica Lindlöf
Alberto Lindner
Daniel Lindner
Ewald Lindner
Volkhard Lindner
Zoe Lindo
David Lindo-Atichati
Steven Lindow
Derick Lindquist
Pelle Lindqvist
Roland Lindqvist
Richard Lindroth
Everett Lindsay
Peter Lindsey
Marcus Lindskog
Carita Lindstedt
Anna Lindstrand
Jon Lindstrom
Nils Lindstrom
Sandra Lindstrom
Eva Lindström
Kristina Lindström
Tom Lindström
Bernt Lindtjørn
Charlotta Lindvall
Antonio Linero
Lisa Lines
Italo Linfante
Erjun Ling
Fangqiong Ling
Feng Ling
Hui Ling
Jiqiang Ling
Kai-Shu Ling
Pei-Ra Ling
Shiqi Ling
Sajan Lingala
Jairam Lingappa
Nura Lingawi
Annett Linge
Theagarten Lingham-Soliar
Enqiang Linghu
Angelika Lingnau
Guido Lingua
Robert Linhardt
Daniel Linhares
Alexander Link
Dan Link

Emma Link
Dirk Linke
Wayne Linklater
Rita Linko
Paul Links
Charles Linn
Jennifer Linn
Sabine C. Linn
Vesa Linnamo
Adrian Linnane
Alexandra Linnemann
Sebastian Linnemayr
Andreas Linninger
Teresa Lino-Neto
T. Linsenmeyer
Anja Linstädter
Joerg Linstaedter
Carole Linster
Martin Linster
MacRae Linton
Paul Lintott
Nicholas Lintzeris
Alexandra Linz
Dominik Linz
John Linz
Alexis Lion
Thomas Lion
Michail Lionakis
Lilla Lionetti
Vincenzo Lionetti
Clifford Liongue
Bill Lionheart
Christos Lionis
Geraldine Liot
Gregory Liou
Jyh-Ming Liou
Saou-Hsing Liou
Jeih-San Liow
Lee Hsiang Liow
Patrycja Lipinska
Marta Lipinski
Peter Lipke
Steven Lipkin
Heather Lipkind
Stanley Lipkowitz
Hans-Peter Lipp
Ottmar Lipp
Peter Lipp
Richard Lippa
Frank Lippert
Gunther Lippert
Donatella Lippi
Sonia Lippke
Sheri Lippman
Jocelyn Lippman-Bell
Arto Lipponen
Hans Joachim Lipps
Esther Lips
Katrin Lips
Paul Lips
Joshua Lipschutz
John Lipscomb
David Lipson
Jack Lipton
John LiPuma
Samuel LiPuma
Wendy Lipworth
Fabio Lira
John Lis
Stefanie Lis
Lynda Lisabeth
Robert Lisak
Thiago Lisboa
Dennis Lischewski
Silke Lischka
Nadra Lisha
Adam Liska
Petra Liskova
Pawel Liskowski
John Lisle
John Lisman
Ton Lisman
Sarka Lisonkova
Katarzyna Lisowska
Steven Liss
Rurik List
Gianluca Lista
Aaron Liston
Chrysa Lithari
Karen Lithgow
Trevor Lithgow
Gianni Liti
Gary Litman
Jordan Litman
Jerzy Litniewski
Paloma Liton
Alan Litsky
Jonathan Litt
Benjamin Littenberg
Alexander Little
Daniel Little
David Little

Dianne Little
Jonathan Little
Joshua Little
Mark Little
Tanya Little
Chris Littlewood
Zachary Litvack
Matthew Litvak
Irene Litvan
Taras Litvin
Anastasia Litvintseva
Sheldon Litwin
Kristin Litzelman
Aiping Liu
Aizhong Liu
Albert Liu
Alice Liu
An Liu
Angela Liu
Bang Liu
Bao Liu
Baodong Liu
Baogang Liu
Baohua Liu
Baoming Liu
Bei Liu
Beidong Liu
Bin Liu
Bing Liu
Binghe Liu
Bingqian Liu
Bingqiang Liu
Bo Liu
Bolan Liu
Botao Liu
Can Liu
Canran Liu
Cathy Liu
Chang Liu
Changchun Liu
Chao Liu
Chaojie Liu
Che-Ming Liu
Chen Liu
Chenggang Liu
Cheng-Tzu Liu
Chien-An Liu
Chi-Hsiu Liu
Ching-Chuan Liu
Ching-Ti Liu
Chin-hsin Liu
Chuang Liu
Chuanhe Liu
Chun-Chi Liu
Chun-Feng Liu
Chun-Hung Liu
Chunsheng Liu
Cindy Liu
Cuiqing Liu
Cun-Zhi Liu
Dandan Liu
David Liu
Deede Liu
Degao Liu
Deguang Liu
Delei Liu
Dengcai Liu
De-Pei Liu
Desheng Liu
Dexiang Liu
Di (Richard) Liu
Dongcheng Liu
Dongfang Liu
Dongfei Liu
Dongxu Liu
Dongyou Liu
Dun Liu
Enke Liu
F. Liu
Fabao Liu
Fang Liu
Fangfang Liu
Fei Liu
Feng Liu
Fu-Tong Liu
Gang Liu
Gangcai Liu
George Liu
Guanjun Liu
Guansheng Liu
Guanshu Liu
GuoShi Liu
Guoxiang Liu
Hai Liu
Haihu Liu
Haijin Liu
Haipeng Liu
Haitao Liu
Hai-Yan Liu
Hao Liu
Hao-Li Liu
Haoyu Liu

Helene Minyi Liu
Hong Liu
Hongbin Liu
Hongbo Liu
Hongling Liu
Hongmin Liu
Hongting Liu
Hongyan Liu
Hsiao-Dung Liu
Hsin-Fu Liu
Hsuan Liu
Hua Liu
Huacheng Liu
Huan Liu
Hu-Chen Liu
Hui Liu
I-Chung Liu
I-Hsin Liu
I-Min Liu
Ingrid Liu
Jeffrey Liu
Jer-Yuh Liu
Jia Liu
Jiabin Liu
Jialing Liu
Jian Liu
Jiandong Liu
Jiang-Qin Liu
Jianguo Liu
Jianhua Liu
Jian-Min Liu
Jian-Ping Liu
Jianquan Liu
Jianzhu Liu
Jiashou Liu
Jiayong Liu
Ji-Bin Liu
Jie Liu
Jifeng Liu
Jin Liu
Jing Liu
Jinghua Liu Liu
Jingwu Liu
Jingze Liu
Jinhua Liu
Jinping Liu
Jiong Liu
Juan Liu
Jue Liu
Ju-Fang Liu
Jun Liu
Jun-Jen Liu
Kai Liu
Kaiyu Liu
Ke Liu
Kebin Liu
Kede Liu
Ken Liu
Kenneth Liu
Keyan Liu
Leqian Liu
Li Liu
Liang Liu
Liezhao Liu
Lili Liu
Lin Liu
Ling Liu
Ling-Zhi Liu
Liwang Liu
Lu Liu
Lukai Liu
Manhua Liu
Man-Qing Liu
Meilian Liu
Meilin Liu
Min Liu
Ming Liu
Minghui Liu
Mingxia Liu
Nan Liu
Ning Liu
P. Liu
Pan-Ping Liu
Peggy Liu
Peng Liu
Pengyuan Liu
Philip Liu
Pi-Jen Liu
Ping Liu
Pingsheng Liu
Po-Hong Liu
Qi Liu
Qiang Liu
Qin Liu
Qing Liu
Qingchang Liu
Qinghua Liu
Qing-Lin Liu
Qingsheng Liu
Qing-Song Liu
Qingyun Liu
Qiong Liu

Qiu-Ning Liu
Ren-Shyan Liu
Renyu Liu
Richard Liu
Rui Liu
Ruicui Liu
Runran Liu
Ryan Wen Liu
Saifeng Liu
Sanzhen Liu
Sha Liu
Shanlin Liu
Shanrong Liu
Shaojun Liu
Shelan Liu
Shichao Liu
Shiguang Liu
Shiguo Liu
Shih-An Liu
Shih-Jen Liu
Shihui Liu
Shikai Liu
Shing Liu
Shu Liu
Shufeng Liu
Shujun Liu
Shuo Liu
Shuqing Liu
Shuyu Liu
Shuzhen Liu
Sichao Liu
Song Liu
Su-Hsun Liu
Suyang Liu
Tao Liu
Taosheng Liu
Teng Liu
Tian Liu
Tianming Liu
Tie Fu Liu
Tiffany Ting Liu
Tsunglin Liu
Wei Liu
Weibo Liu
Weiguo Liu
Weiru Liu
Wen Liu
Wende Liu
Wenhai Liu
Wen-Hsien Lewis Liu
Wenjie Liu
Wenjun Liu
Wenshe Liu
Wen-Tso Liu
Wenya Liu
Wenzhe Liu
X. Sherry Liu
Xiang Dong Liu
Xiangrong Liu
Xiangye Liu
Xianyun Liu
Xiao Liu
Xiao Fan Liu
Xiaobing Liu
Xiaohui Liu
Xiaojing Liu
Xiaojuan Liu
Xiaole Shirley Liu
Xiaolu Liu
Xiaoning Liu
Xiaoping Liu
Xiaorong Liu
Xiaoting Liu
Xiaoyun Liu
Xigang Liu
Ximeng Liu
Xin Liu
Xindong Liu
Xinhai Liu
Xinwang Liu
Xinyi Liu
Xi-Qiang Liu
Xiufan Liu
Xiuwen Liu
Xu Liu
Xuan Liu
Xuanshi Liu
Xuanyao Liu
Xubing Liu
Xuefeng Liu
Xue-Li Liu
Xueyan Liu
Xuhui Liu
Xulei Liu
Xuming Liu
Xun Liu
Xuyang Liu
Y. Liu
Yahui Liu
Yan Liu
Yang Liu
Yanhe Liu

Yanhong Liu
Ya-Wen Liu
Ye Liu
Yecai Liu
Yen-Lin Liu
Yen-Nien Liu
Yen-Wen Liu
Yi Liu
Yi-Chang Liu
Yike Liu
Yiliu Liu
Yilun Liu
Ying Liu
Ying-Bin Liu
Ying-Hsang Liu
Yingyi Liu
Yin-Quan Liu
Yi-Wen Liu
Yongbo Liu
Yongchao Liu
Yongfeng Liu
Yonghong Liu
Yongqin Liu
Yongsheng Liu
Yong-Yu Liu
Yong-Zhong Liu
Yu Liu
Yuan Liu
Yu-Chi Liu
Yudong Liu
Yun Liu
Yuncaho Liu
Yunhua Liu
Yuru Liu
Yusen Liu
Yushi Liu
Yutao Liu
Yuwei Liu
Z. Lewis Liu
Zeming Liu
Zewen Liu
Zexian Liu
Zhandong Liu
Zhangkui Liu
Zhao Liu
Zhaoming Liu
Zhen Liu
Zhenghui Liu
Zhenguo Liu
Zhenzhen Liu
Zhi Liu
Zhi-Hai Liu
Zhihu Liu
Zhihua Liu
Zhilei Liu
Zhili Liu
Zhipeng Liu
Zhi-Ping Liu
Zhixia Liu
Zhiyong Liu
Zhiyuan Liu
Zhongbao Liu
Zhonghua Liu
Zhongsong Liu
Zhongyuan Liu
Zili Liu
Ziqing Liu
Zonghua Liu
Zuojia Liu
Marco Tullio Liuzza
Britt Livak
Giacomo Livan
Todd Livdahl
Helen Liversidge
Gill Livingston
Daniel Livorsi
Adam Liwo
Lisa Lix
Namal Liyanage
Lucylynn Lizarondo
Diego Lizcano
Anne Lize
Lotta Ljung
Karl Ljungberg
Bastien Llamas
Elena Llano
Alejandro Llanos
Paola Llanos
Alberto Lleo
Matilde Lleonart
Adrián Llerena
Martin Llewelyn
Josep M. Llibre
Rafael Llinas
Marc Lliros
Joaquim Llisterri
Nùria Lloberas
Ana Llopart
Xavier Llor
Bertrand Llorente
Briardo Llorente
Josep Lloreta

Celia Lloret-Linares
J. M. Lloris Carsí
Matxalen Llosa
Roland Lloubes
Josep M Llovet
Amanda Lloyd
Andrew Lloyd
Dafydd Lloyd
Donna Lloyd
James Lloyd
Joyce Lloyd
K.C. Kent Lloyd
R. Stephen Lloyd
Shane Lloyd
Mari Lloyd-Williams
Salvador Lluch-Cota
Laura Llull
Chu-Fang Lo
Chung-Ming Lo
Gin-Ho Lo
Janet Lo
Jeng-Fan Lo
Kuang-Yao Lo
Li-Wei Lo
Wai U Lo
Yi-Chun Lo
Yuan-Hung Lo
Yu-Shu Lo
Giosuè Lo Bosco
Claudio Lo Iacono
Lorenzo Lo Muzio
Alessandra Lo Presti
Claudia Lo Sicco
Jordan Loader
Hugo A. Loaiciga
Gwenolé Loas
Z.I. Lobato
Elizabeth Lobb
Rebecca Lobb
Peter Lobel
Phillip Lobel
Jenny Loberg
Guillaume Lobet
Peter Lobie
Muriel Lobier
Gerald Lobley
Anders Løbner-Olesen
Anderson Lobo
Jorge Lobo
Jose Lobo
Peter Lobo
Roanna Lobo
Ronstan Lobo
Sonja Lobo
Valentine Lobo
Jorge Lobo Arteaga
Steven Lobritto
Donald Lobsien
Stephen Locarnini
Joseph Locascio
Denise Locatelli
Francesco Locatelli
Amanda Lochner
Adam Locke
Jayme Locke
Robert Locke
Joseph Locker
Nicolas Locker
Shawn Lockery
Sarah Lockie
Oksana Lockridge
Brent Lockwood
Craig Lockwood
Julie Lockwood
Randall Loder
Andrew Lodge
Caroline Lodge
Jean Lodge
Nilanjan Lodh
Aemen Lodhi
Irfan Lodhi
Sara Lodi
Melissa Lodoen
Gregory Lodygensky
Susan Loeb
Natascha Loebnitz
Bettina Loeffler
Craig Loehle
Ruth Loellgen
Hugues Loemba
Jeremy Paul Loenneke
Andreas Loepke
Adrian Loerbroks
Thomas Loescher
Hannes Loeser
Tobias Loetscher
Markus Loeven
Mark Loewen
Peter Loewen
Yonatan Loewenstein
Alexander Loewer
Joanne Loewy

Johannes Loffing
Romaric Loffroy
Eric Lofgren
Jennifer Lofgren
Christer Lofstedt
Aisha Lofters
Mark Loftin
Elizabeth Loftus
Tyler Loftus
Maria Logacheva
Cathy Logan
Corina Logan
Gordon Logan
K.E. Logan
Kenneth Logan
Susan Logan
Theodore Logan
M. Loganathan
Delphine Logeart
David Logerstedt
Carmen Logie
Colin Logie
Giancarlo Logroscino
Catherine Logue
Everett Logue
Salvatore Loguercio
Denis Logunov
Ping Yeap Loh
Y. Peng Loh
Birgit Lohberger
Katharina Lohmann
Mare Lõhmus
Michael Lohoff
A. Lohrasebi
Andrew Lohrer
Michael Loik
Scott Loiler
Torsten Loin
Hubert Loisel
Alayna Loiselle
Bette Loiselle
Ursula Loizides-Mangold
Monica Loizzo
James Lok
Lynette Loke
Mun Fai Loke
Yoon Loke
Mette Økeland
Yuliya Lokhnygina
Stephen Lokitz
Zerina Lokmic
Anna Lokshin
Irakli Loladze
Kavita Lole
Bernhard Loll
Donald Lollar
Pier Luigi Lollini
Niklas Loman
Koba Lomashvili
Dean Lomax
Isabelle Lombaert
Julian Lombard
Marlize Lombard
Francesca Lombardi
Giovanni Lombardi
Guido Lombardi
Raul Lombardi
Caterina Lombardo
Marco Lombardo
Michael Lombardo
Roberto Lombardo
Antoni Lombarte
Marek Lommatzsch
Slawo Lomnicki
Amedeo Lonardo
András London
Erwin London
Josh London
Stephanie London
Gustavo Londoño
Maria Londoño
Sarah Londrigan
Patrick Lonergan
Hoan Long
Jian-Er Long
Jun Long
Katie Long
Lawrence Long
Meixiao Long
Mian Long
Michael Long
Mingsheng Long
Robert Long
Weiwen Long
Wenxing Long
William Long
Xiaochun Long
Yan Long
Ying Long
Yong Long
Zaiyang Long
Adhemar Longatto-Filho

Ben Longdon
Daniele Longhi
Sonia Longhi
Eleonora Longhin
Ana Leda Longhini
John Longino
Jeffrey Longmate
Mathew Longnecker
Antonio Longo
Benedetto Longo
Francesca Longo
Matthew Longo
Sarah Longo
V. Longo
Valter Longo
Thiago Regis Longo Cesar da Paixão
Claudio Longobardi
Patrizia Longone
Nicholas Longrich
Nick Longrich
Fred Longstaffe
Francesco Longu
Andrew Lonie
Elena Lonjedo
Oona Lönnstedt
Claire Lonsdale
Darcy Lonsdale
Maria Lonzano
Jennifer Loo
Jun-Hun Loo
Keatwei Loo
Lit-Hsin Loo
Rolf Lood
Torey Looft
Chung Yeng Looi
J.L. Looi
Lai-Meng Looi
Brett R. Loomis
Mark Looney
Matthew Loop
Bruno Loos
Maarten Loos
Matthew Loose
Anne Loosen
Andreas Lopata
Kristina Lopatiene
Anatoli Lopatin
Y.M. Lopatin
Tatiana Lopatina
Joyce Loper
Ana Lopes
Anne Lopes
Carina Lopes
Coeli Lopes
Isabel Lopes
Joao Marcelo J. Lopes
John Lopes
José Lopes
Lucia Lopes
Luisa Lopes
Norberto Lopes
Silvio Lopes
Ulisses Lopes
José Lopes de Faria
Iscia Lopes-Cendes
Christophe Lopez
Luis Lopez
Mandi Lopez
Marta Lopez
Rosaria Lopez
Daniel López
Félix Antonio López
Juan López
Maria Celeste Lopez Abbate
José López Bucio
Evangelina López de Maturana
Adolfo Lopez de Munain
Juan Lopez Gappa
Carlos Lopez Garcia
Laura López Hoffman
Elena Lopez Isac
Marcelo Lopez Lastra
Milagros Lopez Mendilaharsu
Rafael Lopez Olarte
Arturo Lopez Pineda
Maria Cristina López Roberts
Monserrat López Sanmartín
Marilucy Lopez Sublet
Diana Lopez-Alvarez
Natalia López-Andrés
Sandra Lopez-Arana
Attener López-Arencibia
Francisco Javier López-Baena
Adria Lopez-Baucells
Abel López-Bermejo
Clemente Lopez-Bote
César López-Camarillo
Jose Luis López-Campos
Elena Lopez-Cancio
S. Lopez-Cortez Mdel
Juan Carlos Lopez-Delgado
Antonio Lopez-Fuenzalida

Esther Lopez-Garcia
Mariana Lopez-Gongora
Luz Lopez-Hernandez
Patricio Lopez-Jaramillo
Pia López-Jornet
Liliana López-Kleine
Luis Lopez-Llorca
Lucia Lopez-Lopez
Jose Lopez-Miranda
Nacho Lopez-Moreno
Francisco López-Muñoz
Rodrigo López-Muñoz
Jose López-Olmeda
Jorge Ramón López-Olvera
Andrés López-Pérez
Enrique A. Lopez-Poveda
Juan Lopez-Raez
Valeriano López-Segura
Reynold Lopez-Soler
Margarita Lopez-Uribe
Carlos López-Vaamonde
Beatriz López-Valcárcel
Leonardo Lopiano
Benjamin Lopman
Paul Loprinzi
Irene Lorand-Metze
Betty Lorch
Gwendolen Lorch
Janice Lord
Kevin Lord
Gaspar Lorén
Natalie Lorent
Mar Lorente
Belén Lorente-Galdos
Axel Lorentz
Annika Lorenz
Claudia Lorenz
Jan Lorenz
Klaus Lorenz
Kristina Lorenz
Sonja Lorenz
Teresa Lorenz
Christian Lorenzen
Johan Lorenzen
Niels Lorenzen
Martina Lorenzetti
Silvio Lorenzetti
Antonello Lorenzini
Bramanti Lorenzo
Óscar Lorenzo
Jacob Lorenzo-Morales
Ramon Lorenzo-Redondo
Cristian Loretelli
Vittorio Loreto
Matthias-Claudio Loretto
Brigitta Loretz
Jody Lori
Francesco Loria
Stephanie Loria
Sylvain Loric
Rik Lories
Balazs Lorincz
Remy Loris
Stefan Lorkowski
Gauthier Loron
Christian Lorson
Alessio Lorusso
Marek Los
Sander Los
Giovanni Losano
Jose Losa-Reyna
Thomas Löscher
Julia Loseff-Silver
Mario Losen
Paul Losty
Katarzyna Lota
Damien Loterie
Nathan Lothrop
Jörn Lötsch
Hannelore Lotter
Christopher Lotz
Jeffrey Lotz
Emil Lou
Hua Lou
Pei-Jen Lou
Qingqing Lou
Qunfeng Lou
Vivian Lou
Xuelin Lou
Ya-Huan Lou
Yanjing Lou
Yilai Lou
Yuan Lou
Zhiyong Lou
Celine Louapre
Philippe Louâpre
James Loudon
Remi Louf
John Loughlin
Psyche Loui
Thomas Louie
Elan Louis

Joe Louis
John Louis
Julien Louis
Petra Louis
Valérie Louis
Valère Lounnas
Alexandre Loupy
Barbara Lourenco
P. Lourenço
Sebastian Lourido
Ricardo Louro
Diana Lousa
Maria Lousada-Ferreira
Denis Loustau
Helder Louvandini
Guillaume Louvel
Goedele Louwagie
Leoni Louwe
Yoram Louzoun
Sandro Lovari
Francesca Lovat
Arianna Lovati
Martin Lövdén
David Love
Robert Love
Ryan Love
Marian Loveday
Jasmine Loveland
Charles Lovell
David Lovell
John Lovell
Jonathan Lovell
Andrew Lovering
Marlina Lovett
Irby Lovette
Johan Lövgren
Jeffrey Lovich
Thelma Lovick
Frank Lovicu
Pierre Lovinfosse
William Lovis
Goran Lovric
Eric LoVullo
Andrew Lovy
Daren Low
Lian Leng Low
Elaine Low-Decarie
Alan Lowe
Anne-Marie Lowe
David Lowe
Graeme Lowe
Martin Lowe
Rachel Lowe
Samantha Lowe
William Lowe
Clifford Lowell
Hubert Löwenheim
Susan Lowerre-Barbieri
Jonathan Lowery
Warren Lowman
E. Anne Lown
Mark Lown
Janet Loxterman
Andre Loxton
Anna Loy
Francisco Lozano
Oscar Lozano
A-Lien Lu
Cheng Lu
Chia-Chen Lu
Chih-Hao Lu
Chunming Lu
Congming Lu
Daru Lu
De-Jian Lu
Dengsheng Lu
Fan Lu
Fu-I Lu
Fung-Jou Lu
Guangquan Lu
Gui Lu
Gui-Hua Lu
Han Lu
Henry Horng-Shing Lu
Hong Lu
Hongbin Lu
Hongzhou Lu
Hua Lu
Hui Jing Lu
Huimin Lu
Jenny Lu
Jiachun Lu
Jianhong Lu
Jin-Jian Lu
Jinsong Lu
Jun Lu
Jyh-Feng Lu
Kai Lu
Kim Lu
Kun Lu
Kuo-Cheng Lu
Lanyuan Lu

Lihua Lu
Lin Lu
Ling Lu
Lingeng Lu
Linlin Lu
Lin-Yu Lu
Liqun Lu
Long Lu
Lu Lu
Mei-Yeh Lu
Meng Lu
Ming-Kuei Lu
Ming-Wei Lu
Mingyang Lu
Mujun Lu
Peiou Lu
Peng Lu
Qi Long Lu
Qiang Lu
Qianjin Lu
Qing-Chang Lu
Qun Lu
Riyu Lu
Rongwen Lu
Ru-Band Lu
Sangwei Lu
Shiang-Ru Lu
Shiyong Lu
Shun Lu
Ting Lu
Tong Lu
Tzong-Shi Lu
Tzu-Pin Lu
Wei Lu
Weiping Lu
Weiqun Lu
Wuxun Lu
Wuyuan Lu
Xiang Lu
Xiang-Jun Lu
Xiaoping Lu
Xiaoyin Lu
Xin Lu
Yanhui Lu
Yanrong Lu
Yi Lu
Yongbo Lu
Yuan Lu
Zhe Lu
Zheming Lu
Zheng-Rong Lu
Zhenyu Lu
Zhijun Lu
Zhiqiang Lu
Zhiyong Lu
Zhongjing Lu
Zhongxian Lu
Zhongyuan Lu
Zunli Lu
Zuxun Lu
Jianhua Lü
Ling Lü
Chun Juan Luan
Junbo Luan
Xianghong Luan
Yun-Xia Luan
Arkadiusz Lubas
Erik Lubberts
Ronald Lubelchek
Mark Lubell
David Lubensky
Pesach Lubinsky
David Lubman
Jim Luby
Marie Luby
Stephen Luby
Corneliu Luca
Sorin Luca
Mark Lucanic
Paulo Lucareli
Giuseppe Lucarelli
Alexander Lucas
Bruno Lucas
Gale Lucas
Michaela Lucas
Philippe Lucas
Richard Lucas
Robyn Lucas
Sophie Lucas
Spencer Lucas
Stuart Lucas
Thabata Lucas
Dani Lucas-Barbosa
Andrea Lucas-Hahn
Magnus Lucassen
Ugo Lucca
Philippe Luccarini
Maurizio Lucchesi
Naomi Lucchi
Sacha Lucchini
Anthony Lucci
Juan Lucena

Rainaldo Lucena
Antônio Roberto Lucena-Araújo
Modesto Luceño
Ersila Lucenteforte
Don Eliseo Lucero-Prisno III
Jean-Christophe Lucet
Adriano Lucheta
Fred Luchette
Andrea Luchetti
Claudio Luchini
Vivian Luchsinger
Ferreiro Lucía
Michelle Luciano
Randy Luciano
Olivier Lucidarme
Julie Lucifora
Diana Lucio-Arias
Gary Luck
Katja Luck
Tobias Luck
Christian Lück
Brian Luckett
Tim Luckett
Brandon Lucke-Wold
Tracy Luckhardt
John Lucocq
Michaela Luconi
Tyrone Lucon-Xiccato
Colwell Lucy
Lukasz Luczaj
Artur Luczak
Juan Ludert
Herbert Ludewick
Ingo Ludolph
Richard Luduena
Elliot Ludvig
Björn Ludwar
Andreas Ludwig
Arne Ludwig
Marie-Gabrielle Ludwig
Stephan Ludwig
Jutta Ludwig-Mueller
Ko-Huang Lue
Lih-Fen Lue
Neal Lue
Aaron Luebbe
Stefan Luebbers
Laura Luebke
Hendrik Luesch
Jose Ruben Luevano Enriquez
Suchaya Luewan
Jennifer Luff
J. Christopher Luft
Francesca Lugani
Aurelia Lugea
Abbas Lugemwa
Fulgentius Lugemwa
Maria Luger
Alessandra Lugo
Joaquin Lugo
Halyna Lugova
Ben Lugtenberg
Dih-Ling Luh
Renke Luhken
Niklas Luhmann
Arthur Luhur
Roger Lui
Cembalo Luigi
Maartje Luijten
Actis Luis
Olinda Luiz
Osmar Luiz
Dip-Kei Luk
Mária Lukácová-Medvidová
Nicholas Lukacs
Dieter Lukas
Thomas Lukas
Piotr Lukasik
Katarzyna Lukasiuk
Henry Lukaski
Katerina Lukasova
Anne-Claire Lukaszewicz
Adam Lukaszewski
Amy Luke
Elizabeth Hanna Luke
Sheila Lukehart
John Lukens
Jodi Lukkes
Suryani Lukman
Angela Lukowski
Robert Lukowski
Andrew Luks
Gediminas Luksys
Konstantin Lukyanov
Dorothée Lulé
Matteo Lulli
Bertram Lum
Sooky Lum
Jean-Pierre Lumaret
Julie Lumeng
Katalin Lumniczky
Mary Ann Lumsden

Desmond Lun
Wenhui Lun
Antonio Luna
Beth Luna
M. Lunar
Adriana Lunardi
Amie Lund
Jay Lund
Troy Lund
Hannah Lundberg
Kristjen Lundberg
Yunxia Lundberg
Jesper Lundbom
Thomas Lundebeberg
Nils Lundeheim
Marjorie Lundgren
Andreas Lundh
Daniel Lundin
Knut Lundin
Lars Lunding
Emma Lundman
Carolyn Lundquist
Erik Lundquist
Andreas Lundqvist
Johan Lundqvist
Maria Lung
Mariia Lunova
Yona Lunsky
David Lunt
Dominique Lunter
Chaoxi Luo
Chen Luo
Cheng Luo
Chunling Luo
Dahai Luo
Daya Luo
Fangli Luo
Gang Luo
Geping Luo
Hai Luo
Hao Luo
Jiangtao Luo
Jianhua Luo
Jiankai Luo
Jian-Ping Luo
Ji-Dung Luo
Jiing-Chyuan Luo
Jing Luo
Jingqin Luo
Kaijun Luo
Ling-Feng Luo
Ming Luo
Mingyue Luo
Minnan Luo
Na Luo
P. Luo
Qian Luo
Robert Luo
Sheng Luo
Sheng-Dean Luo
Shouhua Luo
Shu-Jin Luo
Wanjun Luo
Wenhong Luo
Wenting Luo
Xiaojin Luo
Xiaolan Luo
Xiaoping Luo
Xin Luo
Xiongbiao Luo
Xiong-Jian Luo
Yangmei Luo
Yawei Luo
Yi Luo
Yi-Bo Luo
Yong Luo
Yongfeng Luo
Yu Luo
Yuan Luo
Yufeng Luo
Yukun Luo
Yunjian Luo
Zhe Luo
Zhehui Luo
Zhenyao Luo
Zhijun Luo
Zhongkui Luo
Zisheng Luo
Kaisa Luostari
Angela Lupattelli
Antonella Lupetti
Christopher Lupfer
Richard Lupia
Blanca Lupiani
Philip Lupo
Shawn Lupold
Peter Luppa
Ivan Luptak
Mihai Lupu
Antoni Luque
Ignacio Luque
María D. Luque de Castro

Antonio Luque-Casado
Daniela Luquetti
Rosario Luquin
Nell Lurain
Diana Lurie
Mark Lurie
Jason Luscier
Francis Luscinskas
Natalie Luscombe-Marsh
Donal Luse
Lucy Lush
John Lusingu
Nicholas Lusk
Katherine Lust
Maryam Lustberg
Wioleta Luszczek
Molly Lutcavage
Glen Lutchman
Alexandra Lutnick
Dominique Luton
Irja Lutsar
Pamela Lutsey
Andreas Lutterotti
Monika Lutters
Jeffrey Luttrull
Jens Lutz
Joseph Lutz
Kai Lutz
Manfred Lutz
Thomas Lutz
Hung Luu
Jeffrey Luvall
Koen Luwel
R. Luwor
Thomas Lux
G.W. Gant Luxton
Valerie Luyckx
Paula Luz
Bin Lv
Chen Lv
Fan Lv
Jiale Lv
Linyuan Lv
Tianyang Lv
Wenhua Lv
X. Lv
Zhengmei Lv
Cheng Ly
Meghan Lybecker
Stephen Lycett
Ruth Lyck
David Lydall
Patrick Lyden
Sinéad Lydon
Munn-Sann Lye
Deirdre Lyell
David Lyerly
Amy Lykins
Kenneth Lyles
Lee Lyman
Anastasios Lymperopoulos
Christopher Lynch
Conor Lynch
David Lynch
Elizabeth Lynch
James T. Lynch
Jerome Lynch
Matthew Lynch
Michael Lynch
Shannon Lynch
Stacey Lynch
Michael Lynes
Heidi Lyng
Elsebeth Lynge
Fiona Lynn
Alexander Lyon
Richard Lyon
Elizabeth Lyons
Ian Lyons
Jeri-Anne Lyons
John Lyons
Leslie Lyons
Robert Lyons
Traci Lyons
Karen Lyons-Ruth
Divaldo Lyra Junior
Philippe Lyrer
Tamra Lysaght
Martin Lysak
Inna Lysnyansky
Lisa Lyssenko
Jonathan Lytton
Dmytro Lytvyn
Yuliya Lytvyn
Lyubomyr Lytvynchuk
Sang Woo Lyu
Xiaoxuan Lyu
Julia Lyubovitsky
Phil Lyver
Saraswathi M. S.
Baojun Ma
Buyong Ma

C.Y. Ma
Changle Ma
Chaoran Ma
Cheng-Cang Ma
Chi Ma
Chun-Sen Ma
Cuiping Ma
Cuiqing Ma
Cynthia Ma
Dan-Dan Ma
David Ma
Ding Ma
Dong Ma
Dongyun Ma
Duan Ma
Eric Yue Ma
Estella Ma
Guangyong Ma
Guojia Ma
Hao Ma
Hengyun Ma
Hongshen Ma
Hongxia Ma
Hong-Xia Ma
Hongxiang Ma
Hsiao-Li Ma
Jian Ma
Jianhua Ma
Jian-Xin Ma
Jiaqi Ma
Jin Yeul Ma
Jincai Ma
Jinxuan Ma
Jun Ma
Jun-Yu Ma
K. Ma
Kede Ma
L. Ma
Lei Ma
Liang Ma
Lina Ma
Linlin Ma
Miaojun Ma
Ming-Chieh Ma
Patrick Ma
Peisong Ma
Qianli Ma
Qin Ma
Qing Ma
Renming Ma
Rong Ma
Ruijie Ma
Shao-Gang Ma
Shuangtao Ma
Stephanie Ma
Suk Ma
Ting Ma
Wanbiao Ma
Wan-Li Ma
Wei Ma
Weigang Ma
Weihua Ma
Weimin Ma
Weiqiang Ma
Wen Ma
Wen-Lung Ma
Wenxiu Ma
XiaoChun Ma
Xiaogang (Marshall) Ma
Xiaoguang Ma
Xiaojing Ma
Xiaoning Ma
Xiaoyin Ma
Xie-Min Ma
Xin-Long Ma
Xuejun Ma
Yalong Ma
Yan Ma
Ying Jie Ma
Yitong Ma
Yongqing Ma
Yongxin Ma
Yufang Ma
Zhiyong Ma
Zhonghua Ma
Yuh-Fun Maa
Thomas Maal
Marie Maar
Amy Maas
Angela Maas
Bea Maas
Coen Maas
Renke Maas
Rutger Maas
Benjamin Maasoumy
Alexander Maass
Jorma Maatta
Taylor Maavara
Ulaganathan Mabalirajan
David Mabey
Guillaume Mabilleau
Yo Mabuchi

Micheál Mac Aogáin
Tim Mac Gloughlin
David Mac Lean
María Mac Swiney
David MacAlpine
Fabio Salvatore Macaluso
Filippo Macaluso
Michelle Macaraig
Calum MacAulay
Lance Macaulay
Matthew Macauley
Erasmo Macaya
Paul Maccabee
Marco Maccaferri
Donna MacCallum
Laura Maccari
Alberto Macchi
Mariana Maccioni
Joy MacDermid
Angus MacDonald
Anita MacDonald
Anna MacDonald
Becky Macdonald
Clinton MacDonald
Douglas MacDonald
Ellen Macdonald
Ian MacDonald
Kelly MacDonald
Nicholas MacDonald
Noni MacDonald
Robert Macdonald
Ross MacDonald
Shannon MacDonald
Stuart Macdonald
Suzanne MacDonald
Jacqueline MacDonald Gibson
Conan MacDougall
Scott MacDougall-Shackleton
Emma Mace
Mark MacEachern
Alberto Maceda Veiga
António Macedo
Vaughan Macefield
Bruce MacFadden
Janet Macfall
Shane MacFarlan
David MacFarlane
Peter MacFarlane
Alastair MacGibbon
Harold MacGillavry
François Mach
Nuria Mach
Khaled Machaca
Ana Margarida Machado
Carlos Machado
Ednildo Machado
Fabiana Machado
Fernanda Machado
Heather Machado
José Machado
Jose Carlos Machado
Maria de Fatima Machado
Mariana Machado
Moara Machado
Ricardo Machado
Roberto Machado
Rosangela Machado
T.D. Machado
Miroslav Machala
Zdenko Machala
Boguslaw Machalinski
Jürgen Machann
William Macharia
Olivier Macherey
Keigo Machida
Kiego Machida
Masayuki Machida
Ryuji Machida
Takuji Machida
David Machin
Rikuo Machinami
Maria Teresa Machini
Miloš Macholán
Guillermo Machuca-Portillo
David MacHugh
Fernando Macian
Diego Macias
Juan Macías
Constantino Macias Garcia
Jose G. Maciá-Vicente
Raphael Macieira
Kristine Maciejewski
Gabriel Maciel
Manoela Maciel
Rafael Maciel-de-Freitas
Janet MacInnes
Robert MacInnis
Bradley MacIntosh
Gustavo MacIntosh
Salvador Macip
Hugh MacIsaac
Rachael MacIsaac

J. Scott MacIvor
Benjamin Mack
Katya Mack
Sally MacKain
Alex Mackay
Brendan MacKay
Duncan Mackay
Dylan MacKay
Ian Mackay
Joel Mackay
Judith Mackay
Laura Mackay
Rose-Marie Mackay
Joreintje Mackenbach
Alasdair MacKenzie
Bryan Mackenzie
Gerardo Mackenzie
Grant Mackenzie
Kelly Mackenzie
Roger MacKenzie
Tippi MacKenzie
Todd MacKenzie
Sarah Jane Mackenzie Ross
Gina Mackert
Stephen Mackessy
Abigail Mackey
David Mackey
Dawn Mackey
Roderick Mackie
Sarah Mackie
Robert Mackiewicz
William Mackin
Alexander Mackinnon
Colum MacKinnon
Ruth MacKinnon
Claire Mackintosh
Shylie Mackintosh
Nigel Mackman
Stephen Macknik
Cheryl Mackowiak
Robert MacLaren
Andrew MacLean
Kenneth Maclean
Paul Maclean
Michael MacLellan
Calman MacLennan
Megan MacLennan
Catriona Macleod
Hannah MacLeod
James MacLeod
Kirsty MacLeod
Malcolm Macleod
Megan MacLeod
Norman MacLeod
Katie MacLure
Lee Ann Macmillan-Crow
Katherine MacNamara
Camille Macnaughton
Wallace MacNaughton
Jill Macoska
Sonya MacParland
Alison Macpherson
Ewan MacPherson
Helen Macpherson
Jenny MacPherson
Peter MacPherson
Rebecca MacPherson
Kerry MacQuarrie
Amy MacQueen
Calum MacRae
Kristie Macrakis
Simone Macri
Thomas Macrini
John MacSharry
Jason MacTaggart
Jean-Marc Mac-Thiong
Libor Macurek
Thomas MacVittie
Brian MacWhinney
Aaron Macy
Parvaz Madadi
Vince Madai
Zeynep Madak-Erdogan
Satish Madala
Kazimierz Madalinski
Datta Madamwar
Jason Madan
Karan Madan
Rajat Madan
Taruna Madan
Vanesa Madan
Tariq Madani
Parani Madasamy
Rupalatha Maddala
Anne Madden
Chris Madden
Kenneth Madden
Ros Madden
Guy Maddern
Ted Maddess
Krishna Rao Maddipati
Leena Maddukuri

Mohan Maddur
Scott Maddux
Burkhard Madea
Jean-Yves Madec
Giordano Madeddu
Paolo Madeddu
Luciana Madeira da Silva
Agnieszka Madej-Pilarczyk
Pascal Madeleine
Simone Mader
Stephan Madersbacher
Panagiotis Madesis
Greg Madey
Shabir Madhi
Neha Madhiwala
Mohammad Madhoun
G.M. Madhu
Rama Sashank Madhurapantula
Mallur Madhusudhan
Guillermo Madico
S.R. Murthy Madiraju
Nyovani Madise
Daniel Madison
Zahra Madjd
Rosalinda Madonna
M. Mador
E. Madoroba
Anne-Mette Madsen
Ole Madsen
Steffen Madsen
Anazoeze Madu
Alex Madva
Andres Mae
Akito Maeda
Isseki Maeda
Ken Maeda
Naoyuki Maeda
Shin Maeda
Tatsuya Maeda
Yasuhiro Maeda
Yoshinobu Maeda
Yukihide Maeda
Yumi Maeda
Micha T. Maeder
Marc Maegele
Goro Maehara
Kenji Maehashi
Yasuhiro Maejima
Shinya Maekawa
Takuya Maekawa
Daniel D. Maeng
Steven Maere
Art Maerlender
Charline Maertens de Noordhout
Jeroen Maertzdorf
Dominiek Maes
Gregory Maes
Louis Maes
Akito Maeshima
Elena Maestri
Michelangelo Maestri
Elena Maestrini
Anna Maestroni
Jarek Maestu
Silvia Maffei
Vincenzo Maffei
Claudio Maffeis
Michele Maffia
Andrea Mafficini
Tania Maffucci
Susana Magadan-Mompo
Ana Magalhaes
Elma Izze da Silva Magalhães
Fernando Magalhães
Constantinos Maganaris
Everett Magann
Jules Magda
Anthony Magee
Christopher A. Magee
Jesse Mager
Aude Magerus-Chatinet
Marcio Mageski
Mario Maggi
Matias Maggi
Ivan Maggini
John E. Maggio
Marcello Maggiolini
Lillian Maggio-Price
Zhila Maghbooli
Brian Magi
Kesson Magid
Jessica Magidson
Clint Magill
Peter Magill
Shelley S. Magill
Parker Magin
Thomas Magin
Tara Maginnis
Lucia Magis-Weinberg
Adriano Magli
Alessandro Magli
Dangelo Magliano

Nicholas Magliocca
Fabio Magliulo
Antoine Magnan
Christophe Magnan
Heather Magnan
Jared Magnani
Mauro Magnani
Amandine Magnaudeix
Nicola Magnavita
Ronald Magness
Fulvio Magni
Paolo Magni
Stefano Magni
Brooke Magnus
Maria Magnus
David Magnuson
Mark Magnuson
C.G. Magnussen
Ulf Magnusson
Katharine Magor
Neil Magoski
Priscilla Magrath
Jocelyne Magré
Donatella Magri
Rafael Magris
C Maguire
David Maguire
Jamie Maguire
Kerry Maguire
Beth Mah
Vaikom Mahadevan
W. F. Mahaffee
Joseph Mahaffy
Omid Mahain
Ankit Mahajan
Sahil Mahajan
Supriya Mahajan
Vikram Mahajan
Yatin Mahajan
Smita Mahale
Aba Mahamat
James R. Mahan
John Mahan
Nitish Mahapatra
Sadhan Mahapatra
Saugata Mahapatra
Sidharth Mahapatra
Tanmay Mahapatra
Atsushi Mahara
Surakameth Mahasirimongkol
Krishna Kishore Mahato
Martyn Mahaut-Smith
Sara Mahdavi
Hamideh Mahdaviazad
Hamid Mahdiuni
Fréderic Mahé
Frédéric Mahé
Jaideep Mahendra
Eshwar Mahenthiralingam
Caitriona Maher
Geoffrey Maher
Sean Maher
L. Maher III
Zaynah Maherally
Rajat Maheshwari
Samuel Maheswaran
Shyamala Maheswaran
Mathieu Maheu-Giroux
Lenin Mahimainathan
Sofiane Mahjoub
Hally Mahler
Simon Mahler
Robert Mahley
Jörg Mahlich
Hani Mahmassani
Iftekhar Mahmood
Javed Mahmood
Sadiqa Mahmood
Zafar Mahmood
Ahmed Mahmoud
Haytham Mahmoud
Thair Mahmoud
Shahrom Mahmud
Mehmet Mahmut
Karim Mahnam
Alexander Mahnert
Severine Mahon
Megan Mahoney
Jennifer Mahony
Mohammad Mahoor
Alexander Mahura
Chun-Wai Mai
Knut Mai
Xiaoqin Mai
Ana Luiza Maia
Caroline Maia
Yara Maia
Cláudio Jorge Maia Baptista
Mari Maia da Silva
Juan Pablo Maianti
Helder Maiato
Edward Maibach

Inbal Maidan
Daniel Maidana
Ekaterina Maidji
Ian Maidment
Mark Maienschein-Cline
Bernhard Maier
Christoph Maier
Jeanette Maier
Lisa Maier
Raina Maier
Werner Maier
Wolfgang Maier
Klaus Hermann Maier-Hein
Andreas Maieron
Maxime Maignan
David Maij
Nicolas Maillard
Gary Maillet
Marjorie Maillet
Arnaud Mailleux
Ryan Mailloux
Russell Main
Daniel Maina
Pier Paolo Mainenti
Caterina Mainero
Philippe Maingon
Joel Mainland
Mark C. Mainwaring
Tatiani Maioli
Luigi Maiorano
Eva Maire
Nicolas Maire
Tim Maisch
John Maisey
Vimal Maisuria
Hiroshi Maita
Shishir Maithel
Mrinal Maiti
Nikhil Kumar Maiti
Panchanan Maiti
Kristen Maitland
Arindam Maitra
Sudipta Maitra
Blandine Maitre
N. Maitre
Nathalie Maitre
Matthias Maiwald
Frank Maixner
Aleksandra Majchrzak-Celinska
Marek Majdan
Antonio Majdandzic
Jasminka Majdandžic
Waqar Majeed
Mark Majesky
Shahana Majid
Bonaventura Majolo
Danielle Majoor-Krakauer
Amy Major
Marian Major
Matthew Major
Laszlo Majoros
Shannon Majowicz
Muhammad Majrooh
Ireneusz Majsterek
Claudio Majul
Adhip Majumdar
Indrajit Majumdar
Mala Majumdar
Sanghamitra Majumdar
Subeer Majumdar
Subrata Majumdar
Amita Majumder
Mrinmoyee Majumder
Paromita Majumder
Sadhan Majumder
Alan Mak
Donna Mak
Susanna Mak
Vincent Mak
Azure Makadzange
Maja M. Makagon
Peter Makai
Kepher Makambi
Prestige Makanga
Mariano Makara
Helen Makarenkova
Evgeny Makarov
Slobodan Makarov
Balahan Makay
Paul Makdissi
Miia Mäkelä
Pirjo Mäkelä
Nodar Makharashvili
George Makhatadze
James Maki
Satoshi Maki
Takakuni Maki
Ermei Mäkilä
Tamar Makin
Oluwole Daniel Makinde
Taija Makinen
Veli Makinen

Ville-Petteri Makinen
Shinji Makino
Toshiaki Makino
Wataru Makino
Akifumi Makita
Karim Makkawi
M. Makki
András Makó
Mokhantso Makoae
Yoshida Makoto
Amber Makowicz
I. Joanna Makowska
Liza Makowski
László Makra
Evgenia Makrantonaki
Manousos Makridakis
Christos Makris
Konstantinos Makris
Stergios Makris
Hernán Makse
Mohammad Maktoomi
Jan Makurat
Maria Makuwa
Magdalena Malachowicz
Mokenge Malafa
Davide Malagoli
María Malagón
Pamella Malagrino
Willy Malaisse
Laurence Malandrin
Stefanie Malan-Müller
Umberto Malapelle
Ceila Malaque
Natalia Malara
David Malaspina
Manuela Malatesta
Krishnamurthy Malathi
E.M Malathy
Lorenzo Malatino
Nuria Malats
Aduli Malau-Aduli
Stéfano Malavasi
Iran Malavazi
Alexis Malavazos
Jerry Malayer
Marzia Malcangio
Sebastian Malchow
Hamish Malcolm
Stephen Malcolm
Frank Maldarelli
Stephen Malden
Edio Maldonado
Eduardo Maldonado
Carmen Maldonado-Bernal
Yurixhi Maldonado-López
Ian Male
Teresa Malecka-Massaslka
Kristen Malecki
Goldis Malek
Naveed Malek
Sami Malek
Thomas Malek
Michael Malek-Ahmadi
Mahsa Malekmohammadi
Arati Maleku
Charles Malemud
René Malenfant
Mario Malerba
Kenneth Maleta
Jozsef Maleth
M. Malet-Martino
Fransiska Malfait
Angela Malheiros
Harmeet Malhi
Ripan Malhi
Nawar Malhis
Anshu Malhotra
Arun Malhotra
Bansi Malhotra
Deepak Malhotra
Indu Malhotra
Neena Malhotra
Samir Malhotra
Surbhi Malhotra-Kumar
Sladjana Malic
Patricia Malico Alexandre
Pal Maliga
Kauser Malik
Marek Malik
Neil Malik
Richard Malik
Ruchi Malik
Tanu Malik
Vasanti Malik
Waqar Malik
Zaki Malik
Lenka Malikova
Steven Malin
Tommi Malinen
Andrei Malinovschi
Kristina Malinovskaja
Manuel Maliqueo

Robert Malison
Agustina Malizia
David Malkin
Sebastian Malkusch
Francesca Mallamaci
Kimberley Mallan
Surya Mallapragada
Carina Mallard
Simonette Mallard
Maria Mallardo
Jihad Mallat
Ziad Mallat
Eva Malle
Jennie Mallela
Benoit Malleret
Clément Mallet
Robert Mallet
Andrew Mallett
Sue Mallett
Roland Malli
Luca Mallia
Patrick Mallia
Ujjal Mallick
Hari Mallidi
Karthik Mallilankaraman
Rachel Mallinger
Sandeep Mallipattu
Fedirico Mallo
David Mallon
Jordan Mallon
Nuria Mallorquí-Bagué
Conor Mallory
Ellen Mallory
George Mallory
Carol Mallory-Smith
Allison Malloy
Sneha Mallya
Noah Malmstadt
Andras Malnasi-Csizmadia
Bettina Malnic
Ailish Malone
Brendan Malone
David Malone
James Malone
Christopher Maloney
Ryan Maloney
Anna Malovannaya
Gertraud Malsiner-Walli
Maira Malta
Monica Malta
Jansen Malte
Adam Maltese
Jessica Maltha
João Malva
Amir Malvandi
Mauro Malve
Matteo Malvezzi
Paolo Malvezzi
Anna Malykhina
Khursheed Mama
Adamantios Mamais
Ioannis Mamais
David Maman
Mamas Mamas
Lisley Mambelli
Muhammad Mamdani
Adam Mamelak
Chiara Mameli
Sujan Mamidi
Arvydas Maminishkis
Joshua Mammen
Manoj Mammen
Caterina Mammina
Akiko Mammoto
Tadanori Mammoto
Hassen Mamo
Rogemar Mamon
Emanuelle Mamroud
Manju Mamtani
Hadii Mamudu
Célia Manaia
Efrosyni Manali
Mark Manary
Justen Manasa
Steven Manchester
Laxmaiah Manchikanti
Francesca Mancianti
Anthony Mancini
Elena Mancini
Gianni Mancini
Manuela Mancini
Falk Mancke
Giuseppe Manco
L. Manco
Massimo Mancone
Mauro Manconi
Patrizia Mancuso
Peter Mancuso
Raivo Mänd
Abhyuday Mandal
Atin Mandal
Chandi C. Mandal

Diptasri Mandal
Nripendranath Mandal
Sandip Mandal
Supreet Mandala
Sundhiya Mandalia
Carlos Mandarim-de-Lacerda
Jeffrey Mandel
Jeffrey H. Mandel
Jennifer Mandel
Mark Mandel
Roi Mandel
Jennifer Mandelbaum
Michal Mandelboim
Arthur Mandelin
Jami Mandelin
Erica Mandell
Samuel Mandell
Ulo Mander
Sandy Mandic
James Mandigo
Pierre Mandin
Judith Mandl
Kenneth Mandl
Giuseppe Mandolino
Mattias Mandorfer
Giuseppe Mandraffino
Pranoti Mandrekar
Mauro Mandrioli
Antonia Manduca
Francis Mandy
Chitra Mandyam
Adrian Manea
Kittipong Maneechotesuwan
L. Manenti
Nathan Manes
Zoi Manesi
Nicholas Maness
Mirko Manetti
Giovanni Manfredi
Pablo Manfredi
Piero Manfredi
Michael Manfredo
Vinicio Manfrin
Andrea Manfrinati
Olivia Manfrini
Roberto Manfro
Cameron Mang
Sharad Mangal
Vidya Mangala Prasad
Ashutosh Mangalam
Nilam Mangalmurti
Nitin Mangalvedhe
Sara Manganelli
J. A. Manganello
Stefano Mangani
Carlo Mangano
Francesco Mangano
Philippe Mangeot
Altaf Mangera
Peter Mangesho
Fabio Mangiacapra
Giuseppe Mangialardi
Brigitte Mangin
Paulo Mangini
Martin Mangino
Emily Mangone
Marco Mangone
Arduino Mangoni
Maria Luisa Mangoni
Lara Mangravite
Sangeeta Mangubhai
Mary Kate Manhard
Lisa Manhart
Chinnadurai Mani
Nivedita Mani
Jayanti Mania-Pramanik
Santhakumar Manicassamy
Chaysavanh Manichanh
Cordelia Manickam
Alagu Manickavelu
Anne Manicone
Serge Manie
P Manikandan
Elena Manini
Alexandre Manirakiza
Juliane Manitz
Elias Manjarrez
Mohd Manji
Paul Manka
Gauri Mankekar
Justin Mankin
Richard Mankin
Jamel Mankouri
Robert Mankowski
Bradley Manktelow
Andrew Manley
Kezia Manlove
Brian Mann
David Mann
Dean Mann
Fanny Mann
Jake Mann

John Mann
Karlheinz Mann
Mellissa Mann
Zoe Mann
Adhar Manna
Prasenjit Manna
Inge Mannaerts
Ilaria Mannazzu
Bhanukanth Manne
Jennifer Manne
Claudia Männel
Paolo Mannelli
Jörg Männer
Kaisa Mannerkorpi
Steve Mannheimer
Franz Manni
Amity Manning
Shannon Manning
David Mannino
Gaia Mannino
Marcello Mannino
Melissa Mannion
Peter Mannon
Trish Manns
Yoshiro Mano
Catherine Manoha
Aswin Manohar
Murli Manohar
Muthu Saravanan Manoharan
Narayanan Manoj
Spililios Manolakopoulos
Irini Manoli
Ayumi Manome
Poramate Manoonpong
Carrie Manore
Melinda Manore
Ali Manouchehrinia
Nicholas Manoukis
Arjun Manrai
Aruna Manrakhan
German Manríquez
Norma Manríquez-Morán
Janet Mans
Steve Mansberger
Gemma Mansell
Gordon Mansergh
Aaron Mansfield
Avril Mansfield
Elise Mansfield
Jen Mansfield
Jessica Mansfield
Kate Mansfield
Kim Mansky
Joseph Manson
Michael Manson
Steven Manson
Adnan Mansoor
Hameed Mansoor
Lucia Manso-Silvan
Maedeh Mansoubi
Ahmad Mansour
Hani Mansour
Ahmed Mansouri
Alf Månsson
Luis Manssuer
Leticia Mansur
Jean-Michel Mansuy
Massimo Mantegazza
Renato Mantegazza
Fernando Mantelatto
Lin L. Mantell
Aukje Mantel-Teeuwisse
Daniel Manter
Carlos Mantilla
César Mantilla
Christa Manton
David Manton
Alberto Mantovani
Roberto Mantovani
Shrikant Mantri
Christopher Mantyh
Patrick Mantyh
Dimitra Mantzouka
Candida Manuel
Jennifer Manuel
Oriol Manuel
Remy Manuel
Paolo Manunta
Solichin Manuri
Jennifer Manuzak
Catriona Manville
Christy Manyi-Loh
K. Manz
Rudolf Manz
W. Manzanares
R. Manzanas
Antonio Manzaneda
Edgar Manzanilla
Glen Manzano
Raquel Manzano Martinez
Ann Manzardo
Enzo Manzato

Giancarlo Manzi
Laura Manzo
Claudia Manzoni
Nikhat Manzoor
Binchen Mao
En-Qiang Mao
Gaowei Mao
Hui Mao
Jiafu Mao
Jian-Feng Mao
Kangshan Mao
Limi Mao
Qi Mao
Ri.Cheng Mao
Shu Mao
Weiming Mao
Weiyu Mao
Yinghui Mao
Yubin Mao
Zhiyong Mao
Tonderai Mapako
Rudo Mapanga
Francesca Mapelli
Jill Maples
Salwan Maqdasy
Pierre Olivier Maquart
Lochner Marais
Panagiotis Marakos
Avudaiappan Maran
Christa Marandino
Dario Marangoni
Teodoro Marañon
Athanasios Maras
Felipe Maraschin
Ramona Marasco
Sayed-Amir Marashi
Anna Maria Marata
Nachiket Marathe
Eleftheria Maratos-Flier
Alessandro Maravalle
Antonio Maraver
Pablo Maravilla
Aniko Maraz
Monica Marazuela
Maria Cristina Marazzi
Moreno Marazzi
Daniel Marbach
Eduardo Marbán
Daniel Marc
Robert Marc
Matteo Marcantonio
Paola Marcato
Louis Marceau
Michael Marceau
Jasmine Marcelin
La Noce Marcella
Elena Marcello
Alessandro Marcengo
Alexadre Marceniuk
Arnald Marcer
Brice Marcet
Frederic Marchal
Cyril Marchand
Fabien Marchand
Justine Marchand
Serge Marchand
Veronique Marchand-Pauvert
Gary Marchant
Alessandro Marchese
Giulio Marchese
Federica Marchesi
Giulio Marchesini
Silvia Marchesotti
Dario Marchetti
Giulia Marchetti
Michael Marchetti
Philippe Marchetti
Piero Marchetti
Rosa Marchetti
Martina Marchetti-Deschmann
Paolo Marchettini
Nicola Marchi
Nina Marchi
Timoteo Marchini
Caterina Marchio
Serena Marchio
Davide Marchiori
Paola Marchisio
Douglas Marchuk
David Marciano
Arlei Marcili
Pawel Marciniak
Stefan Marciniak
Ludmila Marcinowicz
Adrian Marciszak
Beato Marco
Francisco Marco
Milena Marcolino
Luciano Marcon
Fernanda Marcondes
Rodrigo Marcondes
Sisi Marcondes

Gianpiero Marconi
Vincent Marconi
Marcos
Fabrizio Marcucci
Lorenzo Marcucci
Carole Marcus
Jeffrey Marcus
Ulrich Marcus
Cecile Marczinski
Karim Mardani
Stefania Mardente
Adil Mardinoglu
Gonzalo Mardones
Curtis Marean
František Marec
Eric Marechal
Jean-Didier Maréchal
Thomas Mareci
Edson Mareco
Hans Maree
Roger Marek
Marc Marenda
Barbara Marengo
Olga Mareninova
Julie Mares
Pavel Mares
Andrea Maresca
Marc Maresca
Isabelle Mareschal
Jennifer Maresh
Thorsten Maretzky
Mary Marfori Christie
Jutta Marfurt
Paolo Margaria
Nikos Margaritelis
Paris Margaritis
G. Margaritopoulos
John Margaritopoulos
Andrew Margenot
Marta Margeta
Peter Margetts
Kim Margolin
William Margolin
David Margolis
Gabriele Margos
Jean-Luc Margot
Samuel Marguerat
Raphael Margueron
Elizabeth Margulis
Karol Marhold
Zoltan Mari
Neus Mari Mena
Mahendra Mariadassou
Babu Mariadoss
Francesca Mariani
Massimiliano Mariani
Patrizio Mariani
Thomas Mariani
Marichelvam Mariappan Kadarkarainadar
Jean Mariaux
Brian Maricle
Boudsocq Marie
Garcia Marie-Isabelle
Hans Marien
Peter Mariën
Eric Marietta
Christophe Mariette
Mylene Mariette
Maria A Mariggiò
Romain Marignier
Ionan Marigomez
Urko Marigorta
Tatiane Mariguela
Pericic Salihovic Marijana
Eloi Marijon
José Maria Marimon
Catherine Marimoutou
Mohan Marimuthu
Bóris Marin
Jose Marin
Maria Marin
Michel Marin
Francisco Marín
Anthony Marinaki
Ferenc Marincs
Daniel Marinho
Francesco Marini
Maddalena Marini
Rich Marini
Angela Marino
Daniel Marino
Francesco Marino
Jennifer Marino
Lori Marino
Maria Marino
Tiziana Marino
Eliana Mariño
Guillermo Mariño
Ines Mariño
Ismael Mariño
Cristina Marino Buslje
Antonella Marino Gammazza

Georgi Marinov
Welber Marinovic
Luyat Marion
Céline Marionneau
François Mariotti
Marco Mariotti
Encarnita Mariotti-Ferrandiz
Christine Maritz-Olivier
Lilian Mariutti
Jose Mariz
Sadie Marjani
David Marjanovic
Varpu Marjomaki
Christie Mark
Faries Mark
Patrick B. Mark
Paul Marker
Sebastian Markett
Charlotte Markey
Kate Markey
Michael Markey
Patrick Markey
Margaret Markiewicz
Erik Marklund
Doris Marko
Lajos Marko
Mollie Marko
Peter Marko
Melissa Markofski
Desalegn Markos
Maria Markoulli
Eleni Markoutsa
Gabriel Markov
Milica Markovic
Silvana Markovska-Simoska
Therese Markow
Beth Marks
Bonita Marks
Donald Marks
Florian Marks
Jennifer Marks
Kathie Marks
Michael Marks
Ray Marks
Stanley Marks
Burgmer Markus
Valentina Markusova
Thor Nygaard Markussen
Matthew Marlay
Ferdinand Marlétaz
W. Marlicz
Jeffrey Marlow
Srinivas Marmamula
Roland Marmeisse
Nelson Marmiroli
R. Marmo
Adam Marmon
Miriam Marmontel
Bradley Maron
Luca Maroni
Gergely Maróti
Vincenzo Marotta
Brian Marples
David Marples
Nicola Marples
Andre Marquand
Quentin Marquant
Gregory Marquart
Catherine Marque
Adilson Marques
Catarina Marques
Fernanda Marques
Joao Marques
Joao Tiago Marques
Joaquim Marques
Márcia Maria Marques
Marcia Martins Marques
Maria Marques
Mario Marques
Miriam Marques
Oriana Marques
Patricia Marques
Sonia Marques
Tiago Marques
Idoia Marqués-Iturria
Pierre Marquet
Rafael Marquez
Johann Marquez-Barja
María Marquine
Robert Marquis
Flavia Marquitti
Robert Marr
Alexandre Marra
Camillo Marra
Kacey Marra
Paolo Marra
Michel Marre
João Marreiros
Emmanuel Marret
Stéphane Marret
Thomas Marrie
Kelly Marrin

Gaetano Marrone
Giusi Marrone
Oreste Marrone
Fabio Marroni
Carl Marrs
Glenn Marrs
Lennart Mars
Martin Marsala
Peter Marschang
Rachel Marschang
Gunther Marsche
Barry Marsden
Islay Marsden
Janet Marsden
John Marsden
Matthew Marsden
Elliot Marseille
Anthony Marsh
Glenn Marsh
Peter Marsh
Samantha Marsh
Wallis Marsh
Aaron Marshall
Andrew Marshall
Brandon Marshall
Jennifer Marshall
John Marshall
Julian Marshall
Katie Marshall
Lynn Marshall
Mark Marshall
Steve Marshall
Tara Marshall
Sonya Marshall-Gradisnik
Zoe Marshman
Michael Marsiske
Marie-Claude Marsolier-Kergoat
Laurent Marsollier
Lukas Eugen Marsoner Steinkasserer
Denise Marston
Marta Marszalek
Bosia Marta
Carlos Marta
Enzo Martegani
An Martel
Jan Martel
Sylvain Martel
Frank Martela
Alberto Martelli
Francesca Martelli
Pier Luigi Martelli
Kirill Martemyanov
Johannes Martens
Jonas Martens
Elena Martens-Uzunova
Christina Marth
Amelia Marti
Hans-Peter Marti
Joan Marti
Melanie Marti
Sara Marti
Janne Martikainen
Alberto Martin
Allan Martin
Allison Martin
Anne-Celine Martin
Arnaud Martin
Bradley Martin
Carlos Martin
Cesar Martin
Charles Martin
Christian Martin
Christina Martin
Damali Martin
Daniel Martin
Denis Martin
Donel Martin
Donna Martin
Douglas Martin
Eileen Martin
Emily Martin
Estelle Martin
Fabiola Martin
Francis Martin
Francisco Martin
Francois-Pierre Martin
Georg Martin
Gerardo Martin
Glynn Martin
Gregory Martin
James Martin
Janet Martin
Javier Martin
Jean-Rene Martin
Jérémy Martin
John Martin
Juan Martin
Julien Martin
Kathy Martin
Kendall Martin
Lana Martin
Lance Martin

Loren Martin
Marcel Martin
Maria J. Martin
Mario Martin
Meghan Martin
Michael Martin
Miguel Martin
Olivier Martin
Pablo Martin
Patrick Martin
Priya Martin
Randy Martin
Regina Martin
Robert Martin
Samuel Martin
Seth Martin
Tammy Martin
Thomas Martin
Tom Martin
Travis Martin
Francisco Martín
Margarita Martín
Carlos Martín Ardila
Tamara Martin Gimenez
Joan Martin Montaner
Camille Martina
Jose Martina
Boris Martinac
Aaron Martin-Alonso
Beatriz Martin-Antonio
Santiago Martín-Bravo
Juan Martin-Caballero
Miguel Martin-Caraballo
Sanda Martincic-Ipsic
Ruben Martin-Clemente
Ana-Belen Martin-Cuadrado
José Martín-Durán
Joe Martineau
Tim Martineau
Jan Martinec
Axel Martinelli
Elena Martinelli
Erika Martinelli
Marcella Martinelli
Paola Martinelli
Pasquale Martinelli
Marianne Martinello
Guillaume Martinent
Laetitia Martinerie
Edoardo Martinetto
Aline Martinez
Ari Martinez
Chris Martinez
Ciera Martinez
Darwin Martinez
Denis Martinez
Diego Martinez
Esteban Martinez
Fernando Martinez
Francisco Martinez
Gustavo Martinez
Jayson Martinez
Jessica Martinez
Leandro Martinez
Leonardo Martinez
Luis Martinez
Manuel Martinez
Maria Carmen Martinez
Neus Martinez
Octavio Martinez
Rafael Martinez
Ramses Martinez
Salvador Martinez
Vicente Martinez
Victor Martinez
Agustín Martínez
Ana Martínez
Carmen Martínez
José Martínez
Lara Martínez
Miguel Ángel Martínez
Octavio Martínez
Alejandro Martinez Bueno
Gonzalo Martinez Fernandez
Miguel Martínez González
Johann Heinz Martinez Huartos
Xavier Martinez Lacasa
Agustin Martinez Molina
Edurne Martínez Moreno
Guillermo Martínez Pérez
Claudia Martínez-Anaya
Olga Martínez-Augustin
Jesús Martínez-Barnetche
Roberto Martínez-Beamonte
Jose Martinez-Costas
Miguel Ángel Martinez-del-Amor
Luis Martínez-Dolz
Ana Martinez-Donate
Flor Martinez-Espinosa
Antonio Martínez-Fuentes
M.A. Martinez-Garcia
Blanca Martinez-Garriga

Luis Martinez-Gil
Pedro Martínez-Gómez
Maite Martinez-Granado
José Armando Martínez-Guarneros
Pablo Martinez-Legazpi
Luis Martinez-Lemus
Enrique Martínez-Meyer
Eduardo Martínez-Montes
Juan Martínez-Pinna
Daniel Martinez-Ramirez
Javier Martínez-Reina
Esperanza Martinez-Romero
Carlos Martinez-Salgado
Aida Martinez-Sanchez
Eduardo Martinez-Valdes
Inigo Martinez-Zubiaurre
Raquel Martín-Hernández
Pierre Martin-Hirsch
Frederico Martinho
Olga Martinho
Andrea Martini
Claudia Martini
Filippo Maria Martini
Rudolf Martini
Severine Martini
Alexandre Martinière
Natália Martínková
Alejandro Martin-Montalvo
José Martín-Nieto
David Martino
Davide Martino
Giovanni Martino
Mikaël Martino
Rosemary Martino
Frédéric Martinon
A. Martin-Padillos
Ines Martin-Padura
Raúl Martín-Palma
Ademir Martins
Alexandra Martins
Ana Martins
André Martins
Antonio Martins
Bruno Martins
Carlos Henrique Martins
Cesar Martins
Elisabete Martins
Gustavo Martins
Helio Martins
João Martins
Lourdes Martins
Rodrigo Martins
Samuel Martins
Viviana Martins
Wellington Martins
Yuri Martins
Sheryl Martin-Schild
Brian Martinson
Jeremy Martinson
Vince Martinson
Marli Martins-Pinge
Lisa Martinsson
Daniel Martin-Vega
Jose-Luis Martin-Ventura
Adam Martiny
Jennifer Martiny
Araks Martirosyan
Nikolay Martirosyan
Christopher Martius
Erwin Märtlbauer
Zsolt Mártonfalvi
Francesca Martora
Reynaldo Martorell
Jeffrey Martus
Regina Martuscello
Alexander Martynov
Anatoly Martynyuk
Lucia Marucci
Liliana Marum
Takeshi Marumo
James Maruniak
Antonello Maruotti
Hiroyuki Marusawa
Jaroslaw Marusiak
Uros Marusic
Ana Marušic
Karen Maruska
Shinsaku Maruta
Eizo Marutani
Fumito Maruyama
Hiroki Maruyama
Jun-ichi Maruyama
Koutatsu Maruyama
Mitsunori Maruyama
Yukio Maruyama
Adriana Marvaldi
Paul Marvar
Giulia Marvaso
Hamid Marvi
Mohamed Marwan
Gary Marx
Steven Marx

Alison Mary
Cristina Marzachi
Shin-Yi Lee Marzano
Caren Marzban
Hassan Marzban
Lucy Marzban
Emanuele Marzetti
Andrea Marzi
Ingo Marzi
Stefano Marzi
Tessa Marzi
Mario Marzilli
Jan Marzinek
Ezequiel Marzinelli
Martin Marziniak
Marco Marzioni
Mary Marzke
William Marzluff
Stefania Marzocco
Gemma Mas de Xaxars
Atsushi Masamune
Honorati Masanja
Segula Masaphy
Mario Masarone
Laurent Mascarell
Françoise Mascart
Celine Mascaux
Julia Mascherbauer
Ulrich Maschwitz
Silvina Masciotra
Joe Masdeu
Masaji Mase
Pavel Masek
Bernd Masepohl
Renato Maserati
Rosalinde Masereeuw
Jose Masero
Biancaelena Maserti
Linnet Masese
Bob Mash
Deborah Mash
Tivani Mashamba-Thompson
Felistas Mashinya
Rahia Mashoodh
Saidur Mashreky
Muriel Masi
Moses Masika
Matilde Masini
Felix Masiye
Cheryl Maslen
Dana Maslovat
Carlos Mas-Moruno
Saber Masmoudi
Isabelle Masneuf
Gianpaolo Maso
Cristina Masoller
Carl Mason
Charlie Mason
Craig Mason
Hugh Mason
Jeffrey Mason
Kevin Mason
Linda Mason
Neil Mason
Nicola Mason
Peggy Mason
Ralph Mason
Richard Mason
Robert Mason
Robin Mason
Sarah Mason
Shaun Mason
William Mason
Sajid Masood
Hassan Masoud
Mohamed Masoud
Ali Masoudi-Nejad
Bruno Masquelier
Tali Mass
Àlex Mas-Sandoval
Serge Massar
Judy Massare
Paola Massari
Emanuele Massaro
Benoit Masse
Eric Masse
Jana Masselink
Jorg Massen
Andrew Massey
P.R. Massey
Ruth Massey
Allan Massie
Charlie Massie
Lourdes Massieu
Chandirasegaran Massilamany
Tattini Massimiliano
Mathieu Massinon
Pierre Massion
Silvia Massironi
Ingrid Masson
Jean-Baptiste Masson
Serge Masson

Tarik Massoud
Patricia Masso-Welch
Francesco Massucci
Nicola Massy-Westropp
Susan Masta
Nikolaos Mastellos
Emma Master
Forrest Masters
William Masters
Grant Mastick
Svetlana Mastitskaya
Teresa Mastracci
Lucy Mastrandrea
Anna Maria Mastrangelo
Salvatore Mastrangelo
Claudio Mastroianni
Gianna Mastroianni Kirsztajn
Salvatore Andrea Mastrolia
Gabriela Mastromonaco
Angela Mastronuzzi
Francesco Mastrototaro
Lisa Masucci
Tahsin Masud
Munetaka Masuda
Ryuichi Masuda
Satohiro Masuda
Tatsuru Masuda
Gwinyai Masukume
Kazuko Masuo
Howard Masuoka
Shazia Masus
Chikara Masuta
Ignacio Mata
Manuel Mata
Sara Mata
Eugenia Mata-Greenwood
Eva Matalova
Miriam Matamales
Valerie Matarese
Bashir Matata
Victor Mateevitsi
José Luis Maté-Muñoz
Pilar Mateo
Rafael Mateo
Rocio Mateo-Gallego
Ana Mateos
Luis Mateos
Mariana Mateos
Maria-Victoria Mateos
Pablo Mateos
Manuela Matesan
Enric Mateu
Guillermo Mateu
Mauricio Mateu
Nohra Mateus-Pinilla
Ewy Mathe
Anthony Mathelier
Ashley Matheny
Amanda Mather
Karen Mather
Kieren Mather
Melissa Mather
William Mather
Colin Mathers
Jonathan Mathers
Kate Mathers
Ulrike Mathesius
Anuja Mathew
Ashish Mathew
Ginson Mathew
Mathew T. Mathew
Oommen Mathew
Remy Mathew
Adam Mathews
Clayton Mathews
Debra Mathews
Maureen Mathews
Michael Mathews
Sarah Mathews
Zmita Mathews
Heather Mathewson
Bernard Mathey-Prevot
Lydia Mathger
Clinton Mathias
Paula Mathiasen
Candace Mathiason
Alistair Mathie
Patrick Mathieu
Veronique Mathieu
Sebastiaan Mathôt
Archana Mathur
Manu Mathur
Maya Mathur
Purva Mathur
Sameer Mathur
Sunita Mathur
Christian Mathys
Isabelle Matias
Miguel Matias
Jordi A. Matias-Guiu
Marie Matignon
Alexis Matikas

Mariano Matilla-Garcia
Maryam Matin
Cleverson Matiolli
Gabor Matis
Daniel Matisoff
Janos Matko
Scot Matkovich
José Mato
Susana Mato
Tetsuya Matoba
Olga Matos
Andreas Matouschek
Joseph Matovu
Takashi Matozaki
Carmela Matrone
Michael Matschiner
Cindy Matsen
Hidenori Matsubara
Ikki Matsuda
Ken-Ichi Matsuda
Koichi Matsuda
S. Matsuda
Shinji Matsuda
Tadashi Matsuda
Tetsuya Matsuguchi
Elizabeth Matsui
Hiroshi Matsui
Reiko Matsui
Chisa Matsumoto
Chota Matsumoto
David Matsumoto
Gen Matsumoto
George Matsumoto
Hidetaka Matsumoto
Hiroyuki Matsumoto
Isao Matsumoto
Kazumasa Matsumoto
Kazunori Matsumoto
Ken Matsumoto
Kunihiro Matsumoto
Kunio Matsumoto
Takashi Matsumoto
Takuya Matsumoto
Yasuharu Matsumoto
Yasuhiko Matsumoto
Yoh Matsumoto
Yoshihisa Matsumoto
Hirofumi Matsumura
Takeshi Matsumura
Tsuyoshi Matsumura
Yasufumi Matsumura
James Matsunaga
Sachihiro Matsunaga
Keita Matsuno
Michinori Matsuo
T. Matsuo
Hiroyuki Matsuoka
Makoto Matsuoka
Shin Matsuoka
Kazuyuki Matsushita
Cristiane Matsuura
Eiji Matsuura
Keita Matsuura
Motokazu Matsuura
Y. Matsuura
Ryota Matsuyama
Shigemi Matsuyama
Takatoshi Matsuyama
Hideo Matsuzaki
Yoko Matsuzaki
Omar Mohamed Mattar
Ursula Matte
Facundo Mattea
Alberto Matteelli
Denise Mattei
Michelle Matter
Thomas Mattern
Joerg Mattes
Andrea Mattevi
Mario Matthaei
Richard Matthew
Alison Matthews
Brya Matthews
Elizabeth Matthews
Geoffrey Matthews
Gregory Matthews
Jason Matthews
Karen Matthews
Keith Matthews
Naomi Matthews
Peter Matthews
R.W. Matthews
Stephen Matthews
Tony Matthews
William Matthews
Stephen Matthey
Suzette Matthijsse
Patrick Matthys
Gianfranco Mattia
Maurizio Mattia
Heather Mattila
Joshua Mattila

W. Brett Mattingly
Monica Mattioli-Belmonte
Aras Mattis
Siobhán Mattison
Simonetta Mattiucci
Holly Mattix-Kramer
Sarah Mattonen
Camilo Mattoni
Carla Mattos
Garrett Mattos
Dirceu Mattos Jr.
Douglas Mattox
Robert Matts
Neil Mattson
Sarah Mattson
Sven Mattys
Giuseppe Matullo
Anke Matura
David Matus
Daniel Matute
Gustavo Matute-Bello
Karen Matvienko-Sikar
Csaba Matyas
Sarah Matyjaszek
A. Matynia
Karl Matz
Sandra Matz
Manolis Matzapetakis
Louis Matzel
Nick Matzke
Felix Mauch
Carmelo Maucieri
Andrew Maudsley
Volker Mauerhofer
Gersende Maugars
Florence Mauger
Jeff Maughan
Maximilian Mauler
Michael Maulucci
Jonathan Maupin
Julie Maupin-Furlow
Joan Maurel
Andrew Maurer
Christoph Maurer
Joachim Maurer
Jurgen Maurer
Kirk Maurer
Peter Maurer
Uli Maurer
Urs Maurer
Mathias Mäurer
Sebastian Maurer-Stroh
Marco Mauri
Donald Maurice
Michèle Maurice
Thomas Maurin
Marguerite Mauritz
Annunziata Mauro
Theodora Mauro
Pál Maurovich-Horvat
Wendy Maury
Ashish Maurya
Carlos Mautalen
Nasim Mavaddat
Vidya Mave
Fabio Mavelli
Uroš Maver
Laurent Maveyraud
A.H. Mavhandu-Mudzusi
Webster Mavhu
Nirmala Mavila
Kenneth Mavor
Clio Mavragani
Penelope Mavragani-Tsipidou
Lazaros Mavridis
Evgeny Mavrodiev
Eleni Mavrogonatou
Sean Maw
Ludo Max
Anton Maximov
Justin Maxwell
Karen Maxwell
Toby Maxwell
Alexis May
Anne May
Clive May
Margaret May
Meghan May
Michael May
Peter May
Victor May
Christopher Mayack
Katerine Mayall
Oleg Mayboroda
Laura J. May-Collado
Peter Maye
Adam Mayer
Audrey Mayer
Bruce Mayer
Christoph Mayer
Deborah Mayer
Gaétan Mayer

Gert Mayer
Günter Mayer
Jens Mayer
Marcos Mayer
Monique Mayer
Otto Mayer
Peter Mayer
Veronika Mayer
Heino Mayer-Bahlburg
Katrin Mayer-Barber
Marius Mayerhoefer
Artur Mayerhofer
Mariana Mayer-Pinto
Theresa Mayes
William Mayes
Anderson Mayfield
Alexandra Mayhew
Christopher Mayhorn
Abdelrahman Mayhoub
Maskit Maymon
C. Maynard
Elizabeth Mayne
Richard Mayne
Kevin H. Mayo
Nancy Mayo
Maria Mayol
Karla Mayolo-Deloisa
Stephen Mayor
Juan Mayordomo-Colunga
Mary G. Mayorga
Evan Mayo-Wilson
Gerald Mayr
Philipp Mayr
Michaela Mayrhofer
Thomas Mayrhofer
Michael Mayrhofer-Reinhartshuber
Darren Mays
Helen May-Simera
Edward Maytin
Nishi Mayumi
Sylvie Mazan
Nicholas Mazarakis
George Mazariegos
Antonios Mazaris
Ian Maze
Mervyn Maze
Michael Maze
Didier Mazel
Marshall Mazepa
Lorraine Mazerolle
Fabienne Mazerolles
Marie-Christine Mazeron
T.D. Mazgajski
Dominique Mazier
Mikael Mazighi
Sarkis Mazmanian
Jean-Xavier Mazoit
Yariv Mazor
Michael Mazourek
Angela L. Mazul
Barsanjit Mazumder
Rajarshi Mazumder
Wojciech Mazur
Vera Mazurak
Gerald Mazurek
Frederic Mazurier
Emilia Mazza
Sandro Mazzaferro
Giuseppe Mazzarella
Michael Mazzeffi
Anna Teresa Mazzeo
Ilaria Mazzini
Mario Mazzocchi
Ketti Mazzocco
Michèle Mazzocco
Paolo Mazzola
Silvia Mazzola
Carlotta Mazzoldi
Stefano Mazzoleni
Guillermo Mazzolini
Annamaria Mazzone
Davide Mazzoni
Rosana Mazzoni
Marco Mazzorana
Diego Mazzotti
Niccolo Mazzucco
Walter Mazzucco
Elisabetta Mazzucotelli
Joseph Mazzulli
Gabriel Mbalaviele
Godfrey Mbaruku
Justice Mbizo
Gerald Mboowa
Lawrence Mbuagbaw
Zizipho Mbulawa
Lazarous Mbulo
Conor Mc Donnell
Kathleen Mc Entee
John Mc Evoy
Jennifer Mc Sharry
Colm McAlinden
Jan McAllister

Lisa McAllister
Sandra McAllister
Sean McAllister
Denise McAloose
Lisa McAndrew
Mary McAndrews
Darragh McArt
J. McArthur
Kate McArthur
Michelle McArthur
John McAteer
Julian McAuley
Julie McAuley
W. J. McAuley
Gary McAuliffe
Joe McAuliffe
Alex McAvoy
Andrew McBain
Karen McBee
Megan McBee
Joyce McBeth
Alan McBride
Anne McBride
Dawn McBride
Jere McBride
Kevin McBride
Mark McBride
Richard McBride
Shonna McBride
William McBride
Keith McBurnett
Brian McCabe
Candy McCabe
Colm McCabe
Kira McCabe
Laura R McCabe
Paul McCabe
Dominic McCafferty
Rebecca McCaffery
Anton McCaffrey
Jeanne McCaffrey
Nikki McCaffrey
Charles McCall
Jennifer McCall
K. D. McCall
Kimberly McCall
Matthew McCall
Shawna McCallin
Ian McCallum
Sarah McCallum
Jon McCalmont
Alana McCambridge
Lynn McCane
Stewart McCann
Robert McCarley
John McCarrey
Kenneth McCarson
Cameron McCarthy
Cathal McCarthy
Danielle McCarthy
David McCarthy
Denis McCarthy
Elizabeth McCarthy
Heather McCarthy
Helen McCarthy
John McCarthy
Justin McCarthy
Peter McCarthy
Robert McCarthy
Catherine McCarty
Douglas McCarty
Jack McCarty
Jessica McCarty
Nael McCarty
Nami McCarty
Owen McCarty
Stacy McCarty
Geoffrey McCaughan
Euan McCaughey
Douglas McCauley
Laurie McCauley
Timothy McCavit
Hilary McClafferty
Amanda McClain
Tim McClanahan
Bruce McClane
Leigh McClarty
Penn McClatchey
Alec McClay
Michael McClean
Angela McCleary-Wheeler
William McClellan
R. McClelland
Raymond McClelland
Robyn McClelland
David McClements
Loren McClenachan
Beverly McClenaghan
James McClintock
Peter McClintock
Karen McCloskey
Elizabeth McClure

Matthew McClure
Roderick McClure
Tristan McClure-Begley
Stuart McCluskey
Kenneth E. L. McColl
Susanna McColley
Jacalyn McComb
Mark McComb
Jonathan McConathy
Brendan McConkey
David McConkey
Bradley McConnell
David McConnell
Kevin McConnell
Margaret McConnell
Michael McConnell
Brian McCool
David McCord
Gordon McCord
Ed McCormack
Koorosh McCormack
Stephen D. McCormic
David McCormick
Samantha McCormick
Joy McCorriston
Paul McCoubrie
James McCoy
Mark McCoy
Rajiv McCoy
Sophie McCoy
Vance McCracken
Pierre McCrea
Aaron McCright
Rory McCrimmon
Brian McCrindle
Jean McCrory
Megan Ann McCrory
Robert Duncan McCuaig
Marshall McCue
Peter McCullagh
Christopher A. McCulloch
Richard McCulloch
Timothy McCulloch
Louise McCullough
Matthew McCullough
Robert McCullumsmith
Kevin McCully
Lorraine McCune
Donald McCurnin
J. McCutchan
Ian McCutcheon
Vivia McCutcheon
Simon McDade
Thomas McDade
Liam McDaid
Larry McDaniel
Lee McDaniel
Michael McDaniel
Allison McDermott
Jennifer McDermott
John C. McDermott
Paul McDermott
Robyn McDermott
Suzanne McDermott
Brian McDonagh
Andrew McDonald
Christine McDonald
Elizabeth L. McDonald
Ellie McDonald
John McDonald
Kerrie McDonald
Laura McDonald
Megan McDonald
Michael McDonald
Mike McDonald
Neville McDonald
Sarah McDonald
Scott McDonald
Adam C McDonnell
Susan McDonnell
Timothy McDonnell
Alicia McDonough
Ian McDonough
Suzanne McDonough
Lotus McDougal
Graham McDougall
Andy McDowell
Mary Ann McDowell
William McDowell
Michael McEachern
Robert McElderry
Patrick McElduff
Teresa McElhinny
Anthony M. McElligott
James McElnay
Thomas McElrath
J. Scott McElroy
Noel McElvaney
Donald McElwain
Sean McElwain
Mark McEntee
John McEvoy

Alistair McEwan
Iain McEwan
Scott McEwen
Joseph McFadden
Sally McFadden
Amelia McFarland
Braden McFarland
Dennis McFarland
Elizabeth McFarland
Karen McFarland
Michael McFarland
Nikolaus McFarland
Robert McFarland
Barbara McFarlin
Melanie McField
Patrick McGah
John McGann
Patrick McGann
Kenneth McGarry
Trudy McGarry
Stephen McGarvey
Suzanne McGaugh
David McGaughey
Jonathan McGavock
Aaron McGee
Lesley McGee
Monnie McGee
Richard G. McGee
Richard McGee Jr.
Meghan McGee-Lawrence
J. Graham McGeown
William McGeown
James McGettigan
Helen McGettrick
George McGhee
Jodi McGill
Mitchell R. McGill
Jessica McGillen
Amanda McGillivray
Rowan McGinley
James McGinnis
William McGinnis
Edwina McGlinn
Rory McGloin
Chris McGlory
Ellen McGough
Alice McGovern
Mark McGovern
Naomi McGovern
Thomas McGovern
Ian McGowan
Jennifer McGowan
Suzanne McGowan
Matthew McGrail
Maura McGrail
Catherine McGrath
Christine McGrath
Colman McGrath
Deirdre McGrath
Margaret McGrath
Michael McGrath
Patrick McGrath
Hillary McGraw
James McGraw
Sloane McGraw
Rose McGready
William McGreevey
Morgan McGregor
Mike McGrew
William McGrew
Rankin McGugin
Betty McGuire
Grant McGuire
Kathleen McGuire
Shawn McGuire
Leah McHale
Andrew McHill
Peter McHugh
William McIlhagga
Gerald McInerney
Michael McInerney
Colin McInnes
Eric McInnes
John McInroy
John McIntosh
Michael McIntosh
Rebecca McIntosh
Stuart McIntosh
Joseph McIntyre
Nancy McIntyre
Thomas McIntyre
Trevor McIntyre
Kathryn McIsaac
David McIver
Paul McKay
Stephanie McKay
Timothy McKay
Jack McKay Fletcher
Martin McKee
Mehri McKellar
Duane McKenna
Leanda McKenna

Sean McKenna
Cheryl McKenna-Neuman
Kwame McKenzie
Matthew McKenzie
Susan McKernan
Luseadra McKerracher
Shane McKie
Aine McKillop
Iain McKillop
Audrey McKinlay
Jake McKinlay
Eliot McKinley
Ross McKinney
Britt McKinnon
Elizabeth McKinnon
John McKinnon
Lyle McKinnon
Ross McKitrick
Thomas McKone
Andrew McLachlan
Angus McLachlan
Craig McLachlan
James McLachlan
Michael S. McLachlan
Robert McLachlan
Sandra McLachlan
Jean McLain
Christine McLaren
Lindsay McLaren
Zoë McLaren
John McLaughlin
Kelly McLaughlin
Nicole McLaughlin
Patricia McLaughlin
Stephen McLaughlin
Thomas McLaughlin
Margaret McLaughlin-Drubin
Marc McLawhorn
Mary-Louise McLaws
Colin McLay
Carmen McLean
Robert McLean
Sara McLean
A. Thomas McLellan
Jason McLellan
Conor McLennan
Jenny McLennan
Damian McLeod
Graem McLeod
Lynette J. McLeod
Kenneth McLeroy
Linda McLoon
Grainne McLoughlin
Alan McLuckie
Caleb McMahan
Melanie McMahan
Francis McMahon
Kathryn McMahon
Kelton McMahon
Michelle McMahon
Sean McMahon
Kimberly McManama O'Brien
James McManaman
Concepta McManus
David McManus
George McManus
Lara McManus
Róisín McManus
Ross McManus
Corey McMillan
Dean McMillan
Elizabeth McMillan
Dave McMillen
Robert McMillen
Matthew McMIllin
Terry McMorris
Tara McMorrow
Caroline McMullan
Rachel McMullan
Colleen McMullen
Mary McMullin
Troy McMullin
Paul McMurdie
David McMurray
Helene McMurray
Josephine McMurray
Kelly McNagny
Margaret McNairy
Mark McNally
James McNamara
Nancy McNamara
Patrick McNamara
Clea McNeely
Marie McNeely
Matthew McNeil
Alison McNeilly
Tom McNeilly
Margaret Windy McNerney
Ruth McNerney
Erin McNerny
Janet McNicholl
Gord McNickle

Archibald McNicol
Helene McNulty
Penelope McNulty
Louise-Anne McNutt
Catherine McParlin
Joanne McPeake
Jamie S. McPhee
Kevin McPhee
Megan McPhee
Misty McPhee
Shannon McPherron
Michael McPherson
Mike McPherson
Matthew McQueen
Robert McQueen
Brad McRae
Kateri McRae
Hayden McRobbie
Neil McRoberts
Ronald McRoberts
Brian McSharry
William McShea
Daniel McSkimming
F. McVerry
Emily McWalter
David McWethy
Ketrell McWhorter
Todd McWhorter
Daniel McWilliams
James Meabon
Julie Meachen
R. Meacock
Kieran Meade
Philip Meade
Mark Meadowcroft
Graham Meadows
Melissa Meadows
Alan Meagher
Matthew Meagher
Rebecca Meagher
Robert Meagher
Thomas Meagher
Judith Meakin
Lee Meakin
Peter Meaney
Sarah Meaney
Steve Meaney
Anna Means
G. Measey
L. Mech
Jasmin Mecinovic
Giuseppe Meco
Blazej Meczekalski
Nuria Medarde
Lorilee Medders
Ray Meddis
David Medeiros
Felipe Medeiros
Jand Venes Medeiros
Liciane Medeiros
Renata Medeiros
Rodrigo Medellin
Jan Paul Medema
Benjamin Meder
Richard Medford
G. Medhi
Marco Medici
Valentina Medici
Enzo Medico
Alexandre Medina
Antonio Medina
Catalina Medina
Gisselle Medina
Milagros Medina
Nagore Medina
Rafael Medina
Raul Medina
Reinhold Medina
Antonieta Medina Lara
Gema Medina-Gomez
Sara Mednick
Matus Medo
Igor Medyna
Paul Mee
Jennifer Meece
Cheryl Meehan
Sean Meehan
Thomas Meehan
Katheryn Meek
Mariah Meek
Mark Meekan
Rick Meeker
Krai Meemon
M.L. Meena
Mukesh Meena
Sebastiaan Meenderink
Jochen Meens
Mariana Meerhoff
Geert Meermans
Annegret Meermeier
Joanne Meers
Melanie Meersch

Esther Meerwijk
Ivan Meeus
Eric Meffre
Persefoni Megalofonou
Reitske Meganck
Salim Megat
Jean-Louis Mege
Sara Meger
Aida Meghraoui-Kheddar
Alberto Megías
Itamar Megiddo
Igor Meglinski
Matteo Megna
Francis Mégraud
Tarek Meguid
Kenichi Meguro
Carolina Mehaffy
Muthamilarasan Mehanathan
Caroline Meharg
Alem Mehari
Céline Mehats
Roopaei Mehdi
Sanjay Mehendale
Kamiya Mehla
Shawn Mehlenbacher
Heinz Mehlhorn
Amjad Mehmood
Thomas Mehner
Mohammad Mehrabadi
Michael Mehring
Lindsay Mehrkam
Arne Mehrkens
Mohammad Mehrmohammadi
R. Mehrotra
Rajesh Mehrotra
Sanjay Mehrotra
Shikhar Mehrotra
Aditi Mehta
Bhairav Mehta
Daryush Mehta
Gautam Mehta
Hemant Mehta
Nehal Mehta
Raaj Mehta
Ranjana Mehta
Sanjeev Mehta
Shivan Mehta
Shwetal Mehta
Sumi Mehta
Marc Mehu
Han-Wei Mei
Jie Mei
Lingyun Mei
Michele Mei
Karin Meibom
Andreas Meid
Christian Meier
Emily Meier
Jeffery Meier
Kathryn Meier
Richard Meier
Rudolf Meier
Liliane Meignen
Carine Meignin
Franck Meijboom
Chris J. Meijer
Harold Meijer
Inge Meijer
Ingeborg Meijer
Karina Meijer
William Meikle
Richard Meilan
Tobias Meilinger
Serge Meimon
Christoph Meinel
Rudi Meir
Pedro Meirelles
Susan Meiring
Christian Meisel
Richard Meisel
Robert Meisel
Zachary Meisel
Christa Meisinger
Dominik Meissner
Eric Meissner
Markus Meissner
Wassilios Meissner
Elissa Meites
Shannon Mejia
Asuncion Mejias
Jorge Mejias
Kh Mekheimer
Hayelom Mekonen
Giuseppe Melacini
Esther Melamed
Michal Melamed
Paul Melancon
Andrew Melbourne
Marc Melcher
Martin Melchers
Maria Gabriella Melchiorre
Alessia Melegaro

Jaime Melendez
Loyda Melendez
Antonio Melendez-Martinez
Thomas Melendy
Carolina Melero
Pedro Melgar-Lesmes
Nereida Melguizo-Ruiz
Hassan Melhem
Paco Melià
Bohuslav Melichar
Heather Melichar
Lester Melie-Garcia
Paolo Melillo
Sharon Melin
Victoria Meliopoulos
Claudia Melis
Giulia Melis
Roberta Melis
Sara Melito
Anette Melk
Isabelle Melki
Loren Mell
Emilia Mellado
Amit Meller
Jarek Meller
Victoria Meller
George Mellick
Michelle Mellis
Alexander Mellmann
Bruno Mello
Claudio Mello
Maria Luiza Mello
Michelle Mello
Lisa Mellon
Ugo Mellone
Duane Mellor
Nathan Mellor
Amine Mellouk
Andreas Melmer
Ana Melo
Eduardo Melo
Felipe Melo
Gisely Melo
Miguel Melo
Ilaria Meloni
Sandro Meloni
Carlo Meloro
Henry Melosh
Cindy Melotte
Wayne Melrose
Alida Melse-Boonstra
Keegan Melstrom
Douglas Melton
Phillip Melton
Andrea Meltzer
Sara J. Meltzer
Geoffrey Meltzner
Andrew Meltzoff
Massimo Melucci
Espen Melum
Shiri Melumad
Stephen Melville
Ryan Melvin
Amir-Hossein Memari
Ali Memarian
Alireza Memarian
Erdogan Memili
Esra Memili
Solomon Memirie
Haris Memiševic
Muhammed Memon
Hongsheng Men
Yujie Men
Ciro Menale
Didier Menard
Armelle Ménard
Idan Menashe
Rima Menassa
Antonella Mencacci
Jörg Menche
Chang Men-Chi
Jose M Menchon
Arianna Menciassi
Jerry Mendel
Lorne Mendell
Mark Mendell
Robert Mendelsohn
Bryan Mendelson
Alexander Mendenhall
Fausto Mendes
Marta V. Mendes
Renato Mendes
Tiago Mendes
Ian Mendez
Pedro Mendez
Ramiro Méndez
Carmen-Dora Mendez-Hernandez
Rosália Mendez-Otero
Cristina Mendez-Vidal
Alberto Mendez-Villanueva
Amy Mendham
Christopher Mendias

Carlos Mendivil
Franklin Mendivil
Murlidhar Mendki
Michael Mendl
Luca Mendler
Janine Mendola
David Mendonça
Marcelo Mendonça
David Mendoza-Cozatl
Rodolfo Mendoza-Denton
Bernardo Mendoza-Santoyo
Naranpurev Mendsaikhan
Maimuna Mendy
Vincent Mendy
Rachel Menegaz
Michela Menegon
Margarita Menéndez
Carlos Menendez-Castro
Ersilia Menesini
Jerome Menet
Charlene Menezes
Jorge Menezes
Ritesh Menezes
Telmo Menezes
Rafael Menezes-Reis
Daniel Menezes-Souza
Fan-Gang Meng
Fanyin Meng
Hongdao Meng
Huicui Meng
Jianjun Meng
Jin Meng
Jinhong Meng
Kangpei Meng
Kyle Meng
Le Meng
Lei Meng
Linghui Meng
Maxwell Meng
Pei-Jie Meng
Qinglai Meng
Ran Meng
Shu Meng
Weihua Meng
Xiangbing Meng
Yan Meng
Yuan Meng
Yuan Xiang Meng
Zheng Meng
Zhipeng Meng
Zhiqiang Meng
Zhuoxian Meng
Cristiane Mengatto
Bruce Menge
Til Menge
Jonas Mengel-From
Catherine Mengelle
Zelalem Mengesha
Elena Mengheri
Dominique Mengin-Lecreulx
Maria Cecilia Mengoli
Alessio Mengoni
Manuela Mengozzi
Ole Mengshoel
Lourdes Mengual
Francesco Menichetti
Lorenzo Menichetti
Donald Menick
Mario Menk
Ricarda Menke
Franck Mennechet
Brian Mennecke
Steven Mennerick
Balaraj Menon
Deepthy Menon
Madhav Menon
Pradeep Menon
Purnima Menon
Ram Menon
Ramkumar Menon
Saras Menon
Swapna Menon
Vijay Menon
Cesar Menor-Salvan
Fiona Mensah
Robert Mensah
Allen Mensinger
Gregorio Mentaberre
Janet C. Mentes
Rolf Mentlein
Giovanni Mento
Alexander Mentzer
Steven Mentzer
Elisabeth Menu
Arnaud Menuet
Karen Menuz
Myles Menz
Barbara Menzaghi
Charles Menzel
Stephan Menzel
Johannes Menzel-Severing
Robert Menzies

Sultan Meo
Giovanni Meola
Flavia Meotti
Zul Merali
Serena Meraviglia
Nofel Merbahi
Sebastiano Mercadante
Jesus Mercado
Diego J. Mercanti
Annette Mercer
Paul Mercer
Stewart Mercer
Tom Mercer
Vicki Mercer
Miguel Merchan
Aziz Merchant
Nathan Merchant
Annie Mercier
Jean-Christophe Mercier
Joffre Mercier
Manuel Mercier
Norbert Mercier
Romain Mercier
Anais Merckx
Genevive Meredith
Robert Meredith
Clement Meredith E
Matthew Meredith-Williams
Pierre Merel
Ivan Merelli
J.J. Merelo
Daniel Merenstein
Daniel M. Merfeld
Irmgard Merfort
Ján Merganic
Stefan Mergler
Thomas Mergner
Stephanie Merhar
Joe Merheb
Seppo Meri
Ashley Merianos
Mathias Mericksay
Daniele Merico
Karine Merienne
Alberto Meriggi
Juha Merilä
Tania Merinas-Amo
Diana Merino
Janie Merkel
Mathias Merker
Rainer Merkl
Rebecca Merkley
Arben Merkoçi
Daphne Merkus
Carsten Merkwirth
Maarten Merkx
Roberto Merletti
Manuela Merli
Didier Merlin
Juan Merlo
Elodie Merlot
George Mermelekas
D. Merrell
Claire Merrifield
Jacqueline Merrill
Michael Merrill
Rebecca Merrill
Scott Merrill
Amy Merrill-Brugger
Tony Merriman
Allen Merritt
Edward Merritt
Russell Merritt
Thomas Merritt
Lisa Merry
Sally Merry
W. David Merryman
Laura Merson
Samuel Merson
Gaetan Mertens
Frederic Mery
Erin Merz
Julian Merz
Maximilian Merz
Hiltrud Merzenich
Ruben Mesa
Annamaria Mesaros
Omar Mesarwi
Tiziana Meschi
Elena Meseguer
Marcos Meseguer
Pablo Mesejo
David Mesher
Dar Meshi
Mena Mesiha
Michael Mesler
Thibault Mesplede
Raquel Mesquita-Ferrari
Evangelos Messaris
Arnaud Messé
Anne Messer
Regina Messer

William Messer
Geralyn Messerlian
Antoine Messiah
G. Messina
Joseph Messina
Albee Messing
Daniel Messinger
Ioannis Messinis
Vanessa Messmer
Andrea Messori
Karen Mestan
Jiri Mestecky
Daniel Mestre
Tiago Mestre
Nadine Mestre-Francès
C Mestres
Heather Metcalf
Kelly Metcalf Pate
Andrew Metcalfe
D. Metcalfe
Jessica Metcalfe
Richard Metcalfe
Mark Metersky
Andrew Metha
Pat Metharom
Spyridon Methenitis
Richard Methot
Shona Methven
Mathieu Métifiot
Matteo Metruccio
Markus Metsälä
Brian Metscher
George Metsios
Amel Mettouchi
Jonathan Metts
Tricia Metts
Ramgopal Mettu
Christine Metz
Silke Metzelthin
Eric Metzen
Michael Metzen
David Metzger
Florian Metzger
Gregory Metzger
Jean-Paul Metzger
Jochen Metzger
Wolfgang Metzger
Dirk Metzler
Rebecca Metzler
B.U. Metzler-Zebeli
Walter Metzner
Sascha Meudt
Yvette Meuleman
Ingrid Meulenbelt
Daniele Meunier
David Meunier
Jean Christophe Meunier
Jean-Dominique Meunier
Joel Meunier
LaVonne Meunier
Patrice Meunier
François Meurens
William Meurer
Jukka Meurman
Ralph Meuwissen
Ludovic Mevel
Dik Mevius
Louise Mewton
Nathan Mewton
David Meya
Patrick Meybohm
Bernd Meyburg
Anne Meyer
Christoph Meyer
David Meyer
Dawn Meyer
Diogo Meyer
Evelyne Meyer
Everett Meyer
Francois Meyer
Gretchen Meyer
Haakon Meyer
Jacob Meyer
Jaimie Meyer
Jeffrey Meyer
Joel Meyer
Julie Meyer
K. Meyer
Kate Meyer
Meghan Meyer
Nicole Meyer
Richard Meyer
Ron Meyer
Sara Meyer
Thomas Meyer
Tim Meyer
Ulrich Meyer
Wallace Meyer III
Gerd Meyer zu Horste
Almut Meyer-Bahlburg
José Meyer-Fernandes
Andreas Meyerhans

Hauke Meyerhoff
Ulf Meyer-Hoffert
David Meyerholz
Gesine Meyer-Rath
Victor-Benno Meyer-Rochow
Amos Meyers
Arthur Meyers
Gregor Meyers
James Meyers
Jason Meyers
Sandra Meyers
Tammy Meyers
Catherine Meyer-Schwesinger
Delphine Meynard
David Meyre
Rafael Meza
Takahiro Mezaki
Leonardo Meza-Zepeda
Edward Mezones-Holguin
Briana Mezuk
Teresa Mezza
Sergio Mezzano
Maria Lina Mezzatesta
Maura Mezzetti
Nyaradzo Mgodi
Leithen M'Gonigle
Nizar Mhaidat
Oussama Mhamdi
Oussama M'Hamdi
Gu Mi
Guohua Mi
Jun Mi
Shengli Mi
Xiangcheng Mi
María Miana
Joseph Miano
Maria Giuseppina Miano
Silvia Miano
Chiyuan Miao
Junying Miao
Long Miao
Qing Miao
Qiucheng Miao
Sheng Miao
Shida Miao
Weibing Miao
Wen Miao
Xuexia Miao
Yi-Liang Miao
Zhongrong Miao
George Mias
Claude Miaud
Tomasz Miazgowski
Alfredo Miccheli
Luca Micci
Laura Miccoli
Antonio Miceli
Cristina Miceli
Francesco Miceli
Rosalba Miceli
Corinne Miceli-Richard
Davis Michael
Drew Michael
W. Michael
Shalom Michaeli
Jacob Michaelson
Jakob Michaëlsson
Peter Michaely
Pavlos Michailopoulos
Johannes Michalak
Marcin Michalak
Marek Michalak
S.J. Michalak
Tomasz Michalak
George Michalopoulos
Lynn Michalopoulos
Christoph W. Michalski
Dominik Michalski
Fernanda Michalski
Nicolas Michalski
Stefan Michalski
Dominique Michaud
J.P. Michaud
Chloé Michaudel
Johan Michaux
Olivier Micheau
Andrew Michel
Brady Michel
Clotaire Michel
Maximilian Michel
Sarah Michel
Stephan Michel
Helen Michelakakis
Frank Michelangeli
Daniel Michele
Manuele Michelessi
Thomas Michelet
Cristian Micheletti
Vania Micheletti
Fabienne Micheli
Giorgia Michelini
Théo Michelot

Aaron Michels
Jan Michels
Nathalie Michels
Anders Michelsen
Brigitte Michelsen
N. Michelutti
Carine Michiels
Joris Michiels
Peter Michielsen
Toshimi Michigami
Matsushita Michinari
Bozena Michniak-Kohn
Frederic Michon
François Michonneau
Franziska Michor
Emilia Michou
Dejan Micic
Alan Mickelson
James Mickelson
Beata Mickiewicz
James Mickle
Marla Mickleborough
Joan C. Mico
Kelli Micocci
V. Micol
Silvia Middei
Mathias Middelboe
Esther Middelkoop
Candace Middlebrooks
Frank Middleton
James Middleton
Mark Middleton
Edoardo Midena
Havard Midgard
Meghan Midgley
Miriam Midoun
Anne Midwinter
Thomas Miedaner
Harald Miedema
Isabelle Miederer
Zofia Miedzybrodzka
Wolfram Miekisch
Marco Mielcarek
Michal Mielcarek
Luca Miele
Mara Miele
H. Mielke
Daniela Mier
Andreas Mierau
Claudia Mierke
Gabry Mies
Nova Mieszkowska
Alexander Miething
Elizabeth Mietlicki-Baase
Maija Miettinen
Markus Miettinen
Muhammad Miftahussurur
Egidia Miftode
Karen Miga
Michael Miga
Americo Migliaccio
Anna Rita Migliaccio
Giovanni Migliaccio
Federica Migliardo
Maria Pia Miglietta
Luciana Migliore
Marcello Migliore
Marco Donald Migliore
Diana Miglioretti
Giovanni Battista Migliori
Adriana Migliorini
Christine Migliorini
Paola Migliorini
Paolo Mignatti
Eve Miguel
Joaquin Miguez
Rada Mihalcea
Joseph Mihaljevic
Jozsef Mihaly
Takahiro Mihara
S.J. Mihic
Sabine Mihm
Nagasawa Miho
Mircea Radu Mihu
Fumika Mi-ichi
Peierdun Mijiti
Paiyz Mikael
Aase Mikalsen
K Mikami
Katalin Mikecz
Emad Mikhail
Dimitri Mikhailidis
Theresa Mikhailov
Alexandra Mikhailova
Atsuya Miki
Toshio Miki
Aleksandar Mikic
Jason Mikiel-Hunter
Irene Mikkelsen
Jørn Mikkelsen
Tuija Mikkola
Mozes Miklos

Sebastian Mikolajczak
Rafael Mikolajczyk
Katsuhiko Mikoshiba
Christian Mikutta
Branko Miladinovic
Lucimar Milagres
Fermin Milagro
Alberto Milan
Valentina Milana
Marco Milanesi
Liliana Milani
Narges Milani
Giuseppe Milano
Marciane Milanski
Béatrice Milard
Marco Milazzo
Paolo Milazzo
Michael Mildner
Dan Milea
Vladimir Milenkovic
Anna Miles
Helen Miles
Michael Miles
Lorin Milescu
Maria Elena Miletto Petrazzini
Dianna Milewicz
Catarina Milheiriço
Andreas Milias-Argeitis
Fabio Milioni
Ingrid Miljeteig
Nadica Miljkovic
Goran Miljus
José Geraldo Mill
Javier Millan
Jose Millan
Ana Millanes
Anthony Millar
Cameron Millar
David P. Millar
Heather Millar
Jocelyn Millar
Russell Millar
Charles Millard
James Millard
Peter Millard
Laura Millares
Douglas Millay
Amy Millen
Dominic Millenaar
Frank Millenaar
Allison Miller
Amy Miller
Andrew Miller
Benjamin Miller
Bill Miller
Brandon Miller
Brenda Miller
Bruce Miller
Charles Miller
Craig Miller
D. Shane Miller
Daniel Miller
David Miller
Dennis Miller
Edgar Miller
Elizabeth Miller
Freeman Miller
Gabrielle F. Miller
Galen Miller
George Miller
Holly Miller
Ingrid Miller
Jason Miller
Jeffrey Miller
Jennifer Miller
Jeremy Miller
John Miller
Jonathan Miller
Jordan Miller
Joshua Miller
Justin Miller
Kai Miller
Karla Miller
Kenneth Miller
Kyle Miller
Laura Miller
Lawrence Miller
Lisa Miller
Luke Miller
Lyndsey Miller
M. Miller
Marcia Miller
Margaret Miller
Mark Miller
Michael Miller
Michele Miller
Patti Miller
Rachel Miller
Russell Miller
Ryan Miller
Seth Miller
Shelley Miller

Steve Miller
Timothy Miller
Tom Miller
W. Miller
Wolfgang Miller
Grégoire Millet
Juan-Pablo Millet
Kobe Millet
Oscar Millet
Jonathan Millett
Stefania Millevoi
Sophia Millington-Ward
Alexander Millman
Alexander Millner
Bradford Mills
Candice Mills
Claudia Mills
Daniel Mills
David Mills
Edward Mills
Kingston Mills
Joshua Millstein
Jason Millward
Elizabeth Milne
Adrienne Milner
Joshua Milner
Matthew Milner
Stasa Milojevic
Staša Milojevic
Marco Milone
Nebojsa Milosevic
Marie-Helene Milot
Leonard Milstone
Hendrik Milting
Jacqueline Milton
Sarah Milton
Ada Miltz
Barbara Milutinovic
R Mimoto
Meryl Mims
H. Mimura
Byoung-Kyong Min
Fan Min
Lei Min
Myo Min
Yoshio Minabe
Tomonori Minagawa
Manabu Minami
Shujiro Minami
Tetsuto Minami
Susumu Minamisawa
Toshifumi Minamoto
Budiman Minasny
Elaine Minatel
Laura Mincarelli
Pierpaolo Mincarone
Todd Minchinton
Thomas Minckley
John Minda
Mark Minden
Joseph Minei
Alessandra Minello
Chieko Mineo
Chundung Miner
Jeffrey Miner
Melissa Miner
A. Minervini
Fabio Minervini
Massimo Minervini
Emmanuel Minet
Geoffrey Minett
Karsten Minette
Carlo Minetti
Liu Ming
Long Chiau Ming
Shi Ming Luo
Claro Mingala
John Mingers
Matteo Minghetti
Marco Minghini
Jesus Mingorance
Lidia Minguez-Alarcon
Pham Minh
Quang Bui Minh
Darren Minier
Anne Marie Minihane
Vladimir Minin
F. Minion
Jessica Mink
Peter Minko
Cho Minkyoung
Liaan Minnie
Helen Minnis
Mari Mino
Yoshihisa Mino
Subhash Minocha
Laëtitia Minodier
Mari Mino-Kenudson
Parviz Minoo
Daniel Minor
Thomas Minor
Samuel Minot

Giorgio Minotti
Lisa Minter
Coilin Minto
Jonathan Minton
Akiva Mintz
Toby Mintz
Filippo Minutolo
Roberto Minutolo
Lisa Miorin
Eneida Mioshi
Paolo Miotto
Sylvie Miquel
Dale Miquelle
Johanna Miquet
Joan Mir
Pablo Mir
Reyazul Rouf Mir
Snober Mir
Giovanni Mirabella
Lucia Mirabella
Massimiliano Mirabella
Clelia Miracco
Maria Miragaia
Daniel Miralles
Angelica Miranda
Carlos Miranda
Daniel Miranda
Kildare Miranda
Marco Miranda
Rafael Miranda
Rajesh Miranda
Susan Miranda
Juan Miranda Olivares
Gustavo Miranda-Carboni
Mohammad Miransari
Cindy Miranti
Gabriel Mircescu
Austin Mircheff
Clotilde Mircher
B. Mirdha
Chad Mire
E. Mireles-Cabodevila
Andrew Mirelman
Brenda Mirembe
Gladys Mirey
Mohammad Miri
Luca Mirimin
Mario Mirisola
Gianluca Mirizzi
Alemnesh Mirkuzie
Daniel Mirman
Parvin Mirmiran
Marco Miroddi
Richard Miron
Veronique Miron
Talya Miron-Shatz
Jon Mirsalis
Alireza Mirshahi
Veriko Mirtskhulava
Sabiruddin Mirza
Shama Mirza
Farah Mirzaagha
H. Mirzabozorg
Ali Mirzazadeh
Takumi Misaka
Masaya Misaki
Kiyoshi Misawa
Harald Mischak
Paul Mischel
Lisa Mische-Lawson
Naobumi Mise
Laurent Misery
Brian Mishara
Kei-ichiro Mishiba
Kenji Mishima
Yuji Mishina
Abhishek Mishra
Asht Mishra
Avinash Mishra
Biswajit Mishra
Debasish Mishra
Geetanjali Mishra
Lopa Mishra
Nilamadhab Mishra
Nishant Mishra
Nitish Mishra
Paras Mishra
Parul Mishra
Phool Mishra
Rakesh Mishra
Sachidananda Mishra
Shailendra Mishra
Sharmistha Mishra
Vikas Mishra
Vimal Mishra
Blazej Misiak
Andrzej Miskiewicz
Lino Misoguti
Anoop Misra
Ashish Misra
Diva Kant Misra
Jyoti Misra

Madhusmita Misra
Prashant Misra
Smita Misra
Manuela Missana
Benjamin Missbach
Markus Missler
Charlotte Mistretta
Chuck Mistretta
Falguni Mistry
Jayalaxshmi Mistry
Toshihiro Mita
Delphine Mitanchez
Yoshihide Mitani
Nilay Mitash
William Mitch
Aaron Mitchell
Anne Mitchell
Brett Mitchell
Cameron Mitchell
Cassie Mitchell
David Mitchell
Elizabeth Mitchell
Gregory Mitchell
Hugh Mitchell
John Mitchell
Julie Mitchell
Pamela Mitchell
Paul Mitchell
Peter Mitchell
Piers Mitchell
Rebecca Mitchell
Robert J. Mitchell
Suzanne Mitchell
Tanecia Mitchell
William Mitchell
Ayoub Mitha
Steven Mithen
Gilles Mithieux
Axel Mithöfer
Carole Mitnick
Adinpunya Mitra
Anirban Mitra
Aniruddha Mitra
Dipak Mitra
Monika Mitra
Pralay Mitra
Sophie Mitra
Sunanda Mitra
Nico Mitro
John Mitrofanis
Alex Mitropoulos
Ioannis Mitroulis
Ragai Mitry
Georgios Mitsis
Kohji Mitsubayashi
Nobutaka Mitsuda
Akira Mitsuhashi
Takahiko Mitsui
Shigeki Mitsunaga
Ashwani Mittal
Jeetain Mittal
Manish Mittal
Payal Mittal
Vijay Mittal
Vinay Mittal
Gary Mittelbach
Maurice Mittelmark
Birgit Mitter
Sayak Mitter
Holger Mitterer
Annat Mitterhofer
Philipp Mitteroecker
Jeffry Mitton
Daisuke Miura
Kazutoyo Miura
Nobuhiko Miura
Shin-ichiro Miura
Takeshi Miura
Tetsuji Miura
Yuri Miura
Hiroto Miwa
Hiroyuki Miwa
Masaki Miya
Masaru Miyagi
Yohei Miyagi
Makoto Miyagishi
Yoshihiro Miyahara
Tempei Miyaji
Fabio Miyajima
Megumi Miyakawa
Sanae Miyake
Masatoshi Miyakoshi
Yuichiro Miyamatsu
Shigeki Miyamoto
Takeshi Miyamoto
Yumiko Miyamoto
Akio Miyao
Makoto Miyara
Akinori Miyashita
Jun Miyata
Kanjiro Miyata
Masaaki Miyata

Shingo Miyata
Takaki Miyata
Makoto Miyazaki
Mariko Miyazaki
Shinya Miyazaki
Tadaaki Miyazaki
Tetsuro Miyazaki
Keisuke Miyazawa
Takayuki Miyazawa
Eiji Miyoshi
Norio Miyoshi
Noriyuki Miyoshi
Takemasa Miyoshi
Tomohiro Miyoshi
Toru Miyoshi
Anja Mizdrak
Atsushi Mizoguchi
Tetsuya Mizoue
Takashi Mizowaki
Yaffa Mizrachi Nebenzahl
Boaz Mizrahi
Mineyuki Mizuguchi
Hiroki Mizukami
Yoichi Mizukami
Kenji Mizumoto
Kazue Mizumura
Makoto Mizunami
Atsushi Mizuno
Cassia Mizuno
Hideaki Mizuno
Hiroshi Mizuno
Masaki Mizuno
Masashi Mizuno
Takuya Mizuno
Tooru Mizuno
Shusei Mizushima
Tsunekazu Mizushima
Akiko Mizuta
Sayaka Mizutani
Giovanni Mizzi
Amosy M'Koma
Premysl Mladenka
David Mladenoff
Ivona Mladineo
Kristin Mmari
Lucas Mmonwa
Volodymyr Mnih
Ji-Hun Mo
Jinhan Mo
Tor Atle Mo
Xiulei Mo
Yin-Yuan Mo
Marocolo Moacir
William Moar
Helen Susannah Moat
Rex Moats
Massieh Moayedi
Danesh Moazed
Ralph Mobbs
Mia Moberg
Aaron Moberly
Nicholas Moberly
Mauro Mobilia
William Mobley
Izabela Mocaiber
Stefano Mocali
Francesco Moccia
Simone Mocellin
Holger Moch
Yoshiyuki Mochida
Manabu Mochizuki
Naoki Mochizuki
Daria Mochly-Rosen
Daniel Mochon
Jason Mock
Kenrick Mock
Amirhossein Modabbernia
Subhasish Modak Chowdhury
Dalit Modan-Moses
Yahya Modarres-Sadeghi
Charin Modchang
Pietro Modesti
Pietro Amedeo Modesti
Sean Modesto
Alessandra Modi
Gopesh Modi
Shiva Kumar Modi
Surbhi Modi
Jaime Modiano
Maria Vittoria Modica
Helmout Modjtahedi
Ute Modlich
Luzia Modolo
Børge Moe
Luke Moe
Rikke H. Moe
Stein R. Moe
Marius Moebius
Carolyn Moehling
Jeff Moehlis
Wenke Moehring
Seyedsina Moeinzadeh

Ralf Moeller
Tobias Moellers
Ron Moen
Thomas Moench
Michel Moenner
Marie-Francine Moens
Yves Moens
Annelies Moerman
Cyril Moers
Caroline Moeser
Joachim Moessner
Siver Moestue
John Moffat
Aldo Moggio
Minoo Moghaddam
Shaida Moghaddassi
Akshata Moghe
Gaurav Moghe
Paolo Moghetti
Alex Mogilner
Daniel Mograbi
David Mogul
Harriette Mogul
Magda Mohamad
Amin Mohamadi
Feroze Mohamed
Islam Mohamed
Junaith Mohamed
Moemen A. Mohamed
Muhanad Mohamed
Salahtoumi Mohamed
Vidya Mohamed Ali
Firdaus Mohamed Hoesein
Khalid Mohamedali
Mahjoub Mohamed-Sofiane
Asif Mohammad
Haroon Mohammad
Saif Mohammad
Ali Mohammadi
Ehsan Mohammadi
Fakhrodin Mohammadi
Hadi Mohammadi
Moosa Mohammadi
Rahim Mohammadi
Altaf Mohammed
Amr Mohammed
Atif Mohammed
Emad A. Mohammed
Hisham Mohammed
Khaled Mohammed
Amita Mohan
Diwakar Mohan
John Mohan
Mahesh Mohan
Pavitra Mohan
Ram Mohan
Subburaman Mohan
Suyash Mohan
V. Mohan
Viswanathan Mohan
Manoj Mohanan
Harini Mohanram
Tapan Mohanta
Ashok Mohanty
Debasisa Mohanty
Keshar Mohanty
Pankaj Mohanty
Sanghamitra Mohanty
Devi Prasad Mohapatra
Saroj Mohapatra
Fady Mohareb
Rosminah Mohd
Mohd Ridzuan Mohd Abd Razak
Mohd Lila Mohd Azmi
Fauziah Mohd Jaafar
Vindhya Mohindra
Muhammad Mohiuddin
Emily Mohl
Martha Mohler
Maura Mohler
Stephan Möhlhenrich
Angelika Mohn
Trine Moholdt
Vijayanand Moholkar
Alicia Mohr
Christine Mohr
Nicholas Mohr
Yasuhiko Mohri
Masuda Mohsena
Vahid Mohsenin
Muhammad Raheel Mohyuddin
Amir Moin
Hervé Moine
Marie-Pierre Moisan
Pia Moisander
Alexander Moise
Vera Moiseenkova-Bell
Genndiy Moiseyev
Steven Moisyadi
Luis Moita
Ethan Moitra
Subhabrata Moitra

Lorenzo Moja
Chris Mojock
Sanyu Mojola
Chi Chiu Mok
Chris Mok
Mo Yin Mok
Peggy Mok
Pooneh Mokarram
Parisa Mokhtari
Lidwine Mokkink
Mikael Mokkonen
Andreas Mokros
Lauren Mokry
Ben Mol
Peter Mol
Monica Molano
Kåre Mølbak
Simone Mölbert
Samat Moldakarimov
Marthe Moldes
Mihai Moldovan
Lucy Moleleki
Dylan Molenaar
Marc Molendijk
Marcelo Molento
Marcos Moleon
Yasmina Molero
Alessio Molfino
Vera Moliadze
Marianne Molin
Mikael Molin
Anthony Molina
Frank Molina
Gustavo Molina
Jean-Michel Molina
Rafael Molina
Samuel Molina
Rafael Molina Soriano
Alvaro Molina-Cruz
Zinnia Judith Molina-Garza
Mariam M Molina-Molina
Eugenio Molina-Navarro
Filippo Molinari
Marco Molinari
Maurizio Molinari
N. Molinari
Sergio Molinari
Antonio Molinaro
Sabrina Molinaro
Ivan Molineris
Ian Molineux
Blanca Molins
Mark Molitch
Gert Moll
Heidrun Moll
Isabella Moll
Remington Moll
Roland Moll
Ashagre Molla
Gianluca Molla
Nathan Mollberg
Catherine Molleareau-Manauté
Freerk Molleman
Lucas Molleman
Brit Mollenhauer
Hans-Jurgen Moller
Luciana Moller
T.P. Moller
Luciana Möller
Michael C. Möller
Sören Möller
Anders Møller
Niels Møller
Per Møller
Marta Mollerach
Geraldine Mollet
Mike Mollet
Luca Mollica
Marcelo Mollinari
Ernesto Mollo
John Mollon
Justin Molloy
Katharine Molloy
Barbara Molnar
Zoltan Molnar
Zsolt Molnar
István Molnár
Nicolas Molnarfi
Fergal Moloney
Nicholas Moloney
David Molony
Lewis Molot
Elena Moltchanova
Anthony Molteno
Maria Molto
María Moltó
Attila Molvarec
Philip Molyneaux
Elizibeth Molyneux
Michelle Momany
Navneet Momi
Paolo Momigliano

Anne Mette Momsen
Cristián Monaco
James Monaghan
Pat Monaghan
Sean Monaghan
Matteo Monami
Umrao Monani
Jean Claude Monboisse
Darren Monckton
Gemma Moncunill
Abdul Mondal
Debasis Mondal
Dinesh Mondal
Kunal Mondal
Pinki Mondal
Mario Mondelli
Stefania Mondello
Guillaume Mondesert
Stanislas Mondot
Bernardo Monechi
Ryan Monello
Rafael Moner
Nigel Mongan
Céline Mongaret Kossmann
Otto Monge
Susana Monge
Luc Mongeau
Philippe Mongeon
Gianluigi Mongillo
Marco Mongillo
Skorn Mongkolsuk
Morgana Mongraw-Chaffin
Cojocaru Monica
Francisco Moniche
Tom Monie
Garg Monika
Mohammed Moniruzzaman
Paul Monis
Anna Moniuszko
Norihito Moniwa
Lucas Monje
M. Monje
Paula Monje
Akira Monji
David Monk
Ian Monk
Jennifer Monk
Juha Mönkäre
Fanny Monneaux
Tony Monnet
Catherine Monnier
Patricia Monnier
Marc Monot
Anna Monreale
Andrew Monroe
Courtney Monroe
Antonio Monroy-Noyola
Maria Monsalve
Stephen Monsell
José Monserrat
Bruce Monson
Ken Monson
Jean-Jacques Monsuez
Michael A. Mont
Dirk Montag
Cristina Montagna
Matteo Montagna
Paul Montagna
Virginie Montagne
Alexandra Montagner
Arnau Montagud
Carl Montague
Karli Montague
Xavier Montagutelli
Esmelda Montalban
Carlos Montalvo
Giovanna Montana
Shaena Montanari
Giuseppe Montanaro
Joan Montaner
Yuri Montanholi
Jean-Marc Montanier
Aldo J. Montano–Loza
Didier Montarras
Hamidreza Montazeri Aliabadi
Andrew Monte
Celia Monteagudo
Jane Montealegre
Andréa Monte-Alto-Costa
Martin Montecino
Alvaro Monteiro
Fernando Monteiro
Gisele Monteiro
Lara Monteiro
Mervyn Monteiro
Renato Monteiro
Ricardo Monteiro
Wuelton Monteiro
Juliana Monteiro de Almeida Rocha
Glaucia Monteiro de Castro
R.S. Monteiro-Junior
Giovanni Monteleone

Denise Montell
Maurizio Montella
Cinzia Montemurro
Filippo Montemurro
Daniel Montero
Carolina Montero-Lopez
Camilo Montes
Fernando Montes
Catharine Montgomery
Elizabeth Montgomery
Janet Montgomery
Jim Montgomery
John Montgomery
McKale Montgomery
Ruth Montgomery
Stephen Montgomery
Eugenio Monti
Gustavo Monti
Lucia Monti
Luca Monticelli
Marco Monticone
Alonso Montiel-Luque
Robson Q Montiero
Giovanni Montini
Guillermina Montoliu
Raquel Montorio
Estrella Montoya
Jared Montoya
Julio Montoya
Quimi Montoya
Ben Montpetit
Marina Montresor
Silvina Montrul
Marta Montserrat
Burrell Montz
Michael Monuteaux
A.K.M. Monwarul Islam
Alice Monzani
Satoru Monzen
Anand Moodley
Clinton Moodley
Jagidesa Moodley
Jayajothi Moodley
Riya Moodley
James Moody
M. Moody
Frits Mooi
Shona Mookerjee
Neeloffer Mookherjee
Ellen Moomaw
Anne Moon
Chan-Hong Moon
Hyo-Bang Moon
Jae Hoon Moon
Min Kyong Moon
Minho Moon
Richard Moon
Seok Jun Moon
Seongwuk Moon
Sung-Sil Moon
Yong-Hwan Moon
Patrick Moonan
Jan-Renier Moonen
Justine Moonen
Andrew Mooney
David Mooney
Edward Mooney
Mark Mooney
Robert Mooney
Scott Mooney
Skyler Mooney
T. Mooney
Aimee Moore
Andrew Moore
Ann Moore
Brian Moore
Catrin Moore
Daniel Moore
Francis Moore
Frederick Moore
Holly Moore
James Moore
Jeff Moore
Jennifer Moore
John Moore
Joseph Moore
Julie Moore
Katrina Moore
Lee Moore
Martin Moore
Nathan Moore
Penny Moore
Richard Moore
Robert Moore
Roger Moore
Sean Moore
Suzanne Moore
Thomas Moore
Elisabeth Moores
Priya Moorjani
Anthony Moorman
J. Moorman

Thomas Moorman
Eddy Moors
Sita Moorthi
Mohammed Moosa
Mahmood Moosazadeh
Kerli Mooses
Eric Mooshagian
Henning Mootz
Iain Moppett
Orna Mor
Delio Mora
Jaume Mora
Francesco Morabito
Santo Morabito
Ricardo Mora-Cartin
Ghobad Moradi
Maziar Moradi-Lakeh
José Moraes
Theo Moraes
Thyago Moraes
Anderson Moraes de Oliveira
Elisangela Morais
Joao Morais
Albert Morales
Aleman Morales
Alfredo J. Morales
Angie Morales
Antonio Morales
Celina Morales
Hernan Morales
Javier Morales
Juan Morales
Marcia Morales
Maria Aurora Morales
Miguel Morales
Noppawan Morales
Rodrigo Morales
Santiago Morales
Luis E. Morales-Buenrostro
Daniela Morales-Espinosa
Rosario Morales-Espinosa
Sonia Morales-Miranda
Jorge Morales-Montor
J. Morales-Rosello
Adriana Morales-Trejo
Hector Mora-Montes
Bruce Moran
Charles Moran
Chris Moran
David Moran
Gary Moran
Joe Moran
Jose Moran
Mary Moran
Michael Moran
Niamh Moran
Xosé Anxelu G. Morán
Enrique Morán Tejeda
Serge Morand
Maria Moran-Diez
Ana Morandini
Jose Moran-Mirabal
Jamie Morano
Kevin Morano
Milena Morano
Roser Morante
José Carlos Morante-Filho
Borja Mora-Peris
Raphael Morard
Esmaeil Morasae
Pietro Morasso
Manuela Morato
Lenka Moravcova
Jirí Moravec
Henning Morawietz
Dean Morbeck
Umberto Morbiducci
Sonsoles Morcillo
Alexa Morcom
Erin Mordecai
Rua Mordecai
Benjamin Mordmueller
Dana Mordue
I. Mordukhovich
Debra Moreau
Regis Moreau
Richard Moreau
Jerome Moreaux
Virginie Moreaux
Andrea Morehouse
Ana Paula Moreira
Daniel Moreira
Ernesto Moreira
Gabriel Moreira
Ivone Moreira
Joao Moreira
Leila Moreira
Leonilde Moreira
Luis Fernando Moreira
Maria Aparecida Moreira
Paula Moreira
Rodrigo Moreira

Xoaquin Moreira
Carlos Moreira-Filho
Etienne Morel
Franck Morel
Francois Morel
Jean-Michel Morel
Laurence M Morel
Penelope Morel
Marie-Christine Morel-Kopp
Nicolas Morellet
Annamaria Morelli
Eugenio Morelli
Federico Morelli
Laura Morello
Kelley Moremen
Maria Morena
Beatriz Moreno
Carmen Moreno
Claudia Moreno
Javier Moreno
Jonathan Moreno
Juan Antonio Moreno
Luisa Moreno
Pablo Moreno
Sylvain Moreno
Yara Moreno
Juan Carlos Moreno Saiz
Oihana Moreno-Arotzena
Gema Moreno-Bueno
Mª Teresa Moreno-Flores
Laura Moreno-López
Ricardo Moreno-Rodríguez
Anneke Moresco
Rosamaria Moresco
Viviana Moresi
Yannick Moret
Antonio Moretti
Marcelo Moretti
Riccardo Moretti
Vittorio Moretti
Ann Morey
Rayo Morfin-Otero
Mariza Morgado
Bryan Morgan
Daniel Morgan
Drake Morgan
Elise Morgan
Ian Morgan
James Morgan
Jason Morgan
Jennifer Morgan
Jess Morgan
Jessica Morgan
John Morgan
Jonathan Morgan
Kevin Morgan
Lucy Morgan
Matthew Morgan
Philip Morgan
Richard Morgan
Rosemary Morgan
Sarah Morgan
Stephanie Morgan
Steven Morgan
Victoria Morgan
William Morgan
Rachael Morgan-Kiss
Francesca Morgante
Lígia Morganti
Michelangelo Morganti
Clement Morgat
Diego Morgavi
Camilla Morgen
Maria Grazia Morgese
Floriana Morgillo
Emiliano Mori
Fumiko Mori
Giorgio Mori
Hitoshi Mori
Kazumasa Mori
Kiyoshi Mori
Lucia Mori
Masaaki Mori
Mattia Mori
Shintaro Mori
Shunsuke Mori
Takefumi Mori
Tetsuya Mori
Tomohisa Mori
Toshifumi Mori
Tara Moriarty
Thomas Moriarty
Hiroki Moribe
Atieh Moridi
Mario Luca Morieri
Takashi Moriguchi
Yusuke Moriguchi
Kohji Moriishi
Kozo Morimoto
Libby Morimoto
Naoki Morimoto
Satoshi Morimoto

Alain Morin
Charles Morin
Cory Morin
Jean Benoit Morin
Randall Morin
Shai Morin
Alberto Morini
Gabriella Morini
Sergio Morini
Akio Morinobu
Béatrice Morio
H. Morioka
Ichiro Morioka
Tomoaki Morioka
Andrea Moriondo
Demetrios Moris
Naho Morisaki
Filomena Morisco
Anselmo Moriscot
Yoshiyuki Morishima
Motoyoshi Morishita
Ryuichi Morishita
Takashi Morishita
Teresa Morishita
Ian Morison
Mathieu Morissette
Hironobu Morita
Masaya Morita
Satoru Morita
Shigeki Morita
Toshisuke Morita
Wataru Morita
Yasu Morita
Gerald Moritz
Robin Moritz
Hiroyuki Moriuchi
Kenta Moriwaki
Shinichi Moriwaki
Hidekazu Moriya
Hideaki Moriyama
Minoru Moriyama
Mitsuhiko Moriyama
Tatsuya Moriyama
Yuki Morizane
Tone Morken
Barbara Morley
Bruce Morley
Georgina Morley
James Morley
Peter Morley
Pierre Mormède
Ana Moro
Esteban Moro
Pedro Moro
Ana Moro-Egido
Diane Morof
Francisco J Morón
Renato Morona
Flaviano Morone
Jose Morones-Ramirez
Elisabetta Moroni
Gota Morota
Michael Morowitz
Ludmilla Morozova-Roche
Russell Morphew
Giulia Morra
Mostafa Morra
Nuria Morral
Sarah Morran
Hans Morreau
C. Jane Morrell
Maureen Morrin
Aaron Morris
Alison Morris
Ann Morris
Brian Morris
Carol Morris
Christopher Morris
Claudia Morris
David Morris
Douglas Morris
Gilbert Morris
James Morris
John Morris
Katherine Morris
Kevin Morris
Luc Morris
Margaret Morris
Meg Morris
Michele Morris
Molly Morris
Paul Morris
Peter Morris
Rebecca Morris
Richard Morris
Shaun Morris
Sheldon Morris
Steven Morris
Van Morris
Zachary Morris
Donald Morrison
Ivan Morrison

Katherine Morrison
Kathleen Morrison
Kathryn Morrison
Lynda Morrison
Rob Morrison
Sherie Morrison
Trudy Morrison
Vicky Morrison
Naomi Morrissette
Anne Morrissey
Christy Morrissey
Eimear Morrissey
Doralisa Morrone
Giovanni Morrone
Jennifer Morrow
Johnica Morrow
Richard Morrow
Joachim Morschhäuser
Caryn Morse
David Morse
Gene Morse
Michael Morse
Wayde Morse
Herbert Morse III
Alexander Mörseburg
John Morser
Bashir Morshed
Saam Morshed
Dina Morshedi
Richard Mort
Renato Mortara
Marina Mortati Dias
Beatriz Morte
Ashley Mortensen
Chris Mortensen
Motazakker Morteza
Arthur Mortha
Geert Mortier
Alec Morton
Ben Morton
Cynthia Morton
Geraint Morton
Gerard Morton
James Morton
Jennifer Morton
Katie Morton
Golam Mortuza
Alicia Morugan
Joanna M Morys
Anita Morzillo
Marco Moscarelli
Alessandro Moscatelli
David Moscatelli
Roberto Moscetti
Marco Moschetta
Peter Moschovis
Salerwe Mosebi
Hunter Moseley
Michael Moseley
Mosepele Mosepele
André Moser
Florian Moser
Franklin Moser
Jason Moser
Katrina Moser
Markus Moser
René Moser
Susan Moser
Harold Moses
Nick Mosey
Fausta Mosha
Han Moshage
Deane Mosher
Javid Moslehi
Jonathan Mosley
Ian Moss
Jacqueline Moss
Laura Moss
Madonna Moss
Sarah Moss
T. Moss
Travis Moss
Enrico Mossello
Richard Mosser
Peter Mossey
Jim Mossman
Joel Mossong
Heba Mostafa
Ehsan Mostafavi
Sara Mostafavi
Elahe Mostaghel
Mohammad Mostofa
Anna Moszczynska
Luís Mota
Manuel Mota
Paula Mota
Babu Motagi
Achenef Motbainor
Bianca Mothé
Carmel Mothersill
Mohammad Reza Safari Motlagh
Lukas Motloch

Norio Motohashi
Ikuko Motoike
Chie Motono
Yuri Motorin
Edith Motschall
Elizabeth Motta
Fábio Motta
Daisy Motta-Santos
Hans Motte
Roberto Motterlini
Denis Mottet
Shan Mou
Xiaozhen Mou
Zhonglin Mou
Dany Pascal Moualeu
Pauline Mouawad
Kamal Moudgil
Gregory Mouille
Ahmad Moujahed
Abdelmalik Moujahid
Judd Moul
John Moulder
Richard Moulding
Panagiotis Moulos
John Moult
Lawrence Moulton
Michael Moulton
Stephane Mouly
Luke Mounce
Lourdes Mounien
Kate Mounsey
George Mount
Giorgos Mountrakis
Nina Mounts
John Mountz
Giannis Mountzios
Kostas Mountzouris
Claire Mouquet-Rivier
Alexandra Moura
Egberto Gaspar Moura
Mirian Moura
Ana M. Moura-da-Silva
Gulherme Mourao
Miguel Mourato
Johann Mourier
Rogier Mous
Ahmed Mousa
Haider Abdul-Lateef Mousa
Mohamed Mousli
Ahmed Moussa
Mehdi Moussaid
N. Moussaoui
Nabila Moussaoui
Aline Moussard
Normand Mousseau
Dina Moustafa
Mahmoud Moustafa
Mohamed Moustafa
Naïma Moustaïd-Moussa
Petros Moustardas
Dimitrios Moutopoulos
Christina Moutsiana
Athanasia Mouzaki
Dionysios Mouzakis
Farahnaz Movahedzadeh
Hesam Movasagh
Jamileh Movassat
Allan Mowat
Danielle Mowery
Sarah Mowry
Linda Moxey
John Moxnes
Joe Moxon
Cristina Moya
Mark Moyad
Francisco Moyano
Nieves Moyano
Cheryl Moyer
Jeffrey Moyer
Scott Moye-Rowley
Amie Moyes
Andrew Moyes
David Moyes
K. Moyes
Robert Moyle
Wendy Moyle
Sizulu Moyo
Robert Moyzis
Hadi Mozafari
Masoud Mozafari
Mikhail Mozerov
Pallab Mozumder
Andrea Mozzarelli
Arthur Mpimbaza
Anastasios Mpotsaris
J. Mrázek
M. Mrinalini
Krzysztof Mrozek
Michal Mrug
Michael Msall
Gerry Mshana
T. Mthiyane

Chang-Kao Mu
Huiling Mu
Jianbing Mu
Jung-Jung Mu
Ren Mu
Tingwei Mu
Wanmeng Mu
Wenbo Mu
Xian-Yun Mu
Yifen Mu
Yuguang Mu
Chatchai Muanprasat
Davoodbasha MubarakAli
Samira Mubareka
Adele Mucci
Giulio Muccioli
Wellington Muchero
Antoine Muchir
Martin Mücke
Thomas Mücke
Eric Mucker
Mauricio Mudadu
Joseph Mudd
Giridhar Mudduluru
Mary Beth Mudgett
Amritpal Mudher
Maria Mudryj
Gary Muehlbauer
Jochen Muehlschlegel
Ann-Kristin Mueller
Benjamin Mueller
Beth Mueller
Casey Mueller
Christian Mueller
Christopher Mueller
Dominik Mueller
Elizabeth Mueller
Erik Mueller
Eugene Mueller
Gregory Mueller
Johannes Mueller
Judith Mueller
Karsten Mueller
Markus Mueller
Martin Mueller
Notger Mueller
Rebecca Mueller
Scott Mueller
Sebastian Mueller
Steffen Mueller
Thomas Mueller
Doris Mueller-Doblies
Christoph Mueller-Pfeiffer
Oliver Mueller-Stricker
Marcus Muench
Peter Muennig
S. Muenst
Christian Muenz
Joseph Muenzer
Julia Muenzker
Anne Muesch
Kim Mueser
Stefan Muetzel
Ilgiz Mufazalov
Alexis-Michel Mugabushaka
Raja Mugasimangalam
Sam Mugford
Javier Muguerza
Teja Muha
A. Muhammad
Muhammad Marwan Muhammad Fuad
Abdul Kadar Muhammad Masum
M. Mühlau
Kristin Mühldorfer
Paul Muhle
Marianne Muhlebach
Paul Muhle-Karbe
Christian Mühlfeld
Paolo Muiesan
Amanda Muir
Keith Muir
Kanae Mukai
Galina Mukamolova
Samson Mukaratirwa
Liliane Mukaremera
Geetha Mukerji
Tapan Mukerji
A. Mukherjee
Jean Mukherjee
Jogeshwar Mukherjee
Konark Mukherjee
Nabanita Mukherjee
Pranab Mukherjee
Pulok K. Mukherjee
Raju Mukherjee
Satyam Mukherjee
Shrabani Mukherjee
Murembiwa Mukhola
Abhiroop Mukhopadhyay
Anirban Mukhopadhyay
Bani Mukhopadhyay
Debashis Mukhopadhyay

Partha Mukhopadhyay
Rupak Mukhopadhyay
Sangita Mukhopadhyay
Satinath Mukhopadhyay
Maowia Mukhtar
Trilochan Mukkur
Fungai Mukome
Sharif Mukul
Harshini Mukundan
Joram Mul
Agata Mulak
Ajitkumar Mulavara
Lawrence Mulcahy
Nicholas Mulcahy
Christa Mulder
Mark Mulder
Pamela Mulder
Petra Mulder
Eavan Muldoon
Katherine Muldoon
Sarah Muldoon
Flavia Mule
Albert Mulenga
Rosario Muleo
Rita Mulherkar
Shalaka Mulherkar
Adrian Mulholland
Padraig Mulholland
Pablo Mulieri
Luke Mullany
Isis Mullarky
Craig Mullen
M. Mullen
Sean Mullen
Tim Mullen
William Mullen
Christo Muller
Elmi Muller
Ferenc Muller
François L.L. Muller
Laurent Muller
William Muller
Yunhua Muller
Andreas Müller
Astrid Müller
Barbara Müller
Bettina Müller
Caroline Müller
Dafne Müller
Hans-Peter Müller
Henning Müller
Henry Müller
Hermann Müller
Johannes Müller
Kristina Müller
Marcel Müller
Markus Müller
Martin Müller
Susanne Müller
Thomas Müller
Thorsten Müller
Tobias Müller
Viktor Müller
Florian Müller-Dahlhaus
Jennifer Mulligan
Christopher Mullin
Gerard E. Mullin
Conrad W. Mullineaux
Lucille Mullins
Niamh Mullins
Robert Mullins
Benjamin Mullish
Joaquim Mullol
Barbara Mulloy
Siva Mulpuru
Melissa Mulraney
Daniel Mulrooney
Gerd Multhaup
Gerhard Multhaup
Yeshalem Mulugeta
Kelly Mulvey
John Mulvihill
Minal Mulye
Joyce Mumah
K. Mummenhoff
Eun-Young Mun
Masanori Munakata
Daniele Munari
Sarah Munce
Steven Muncer
Elizabeth Munch
Inger Christine Munch
Jan Munch
Andreas Mund
John Munday
Philip Munday
Emily Mundorff
Roger Mundry
Christopher J. Mundy
William Mundy
Tomoatsu Mune
Alejandro Múnera

Jun Muneuchi
Chris Mungall
Joshua Munger
Kassandra Munger
Kristen Munger
Givemore Munhenga
Carolina Munhoz
Renato Munhoz
Nira Munichor
Tariq Munir
Arasambattu Kannan Munirajan
Sakthivel Muniyan
Raquel Muñiz-Salazar
Carmine Munizza
Samuel Munjita
Carsten Münk
Tamara Munkemuller
Sarah Munkholm
Maureen Munn
Sergi Munné-Bosch
Alvaro Munoz
Colleen Munoz
Francisco Munoz
Raquel Munoz
Ana Maria Muñoz
Carlos Muñoz
Estela Muñoz
Juan Muñoz
Luis Muñoz
Miguel Muñoz
Mónica Muñoz
Nacira Muñoz
Pilar Muñoz
Miquel Muñoz Pérez
Carmen Muñoz-Almagro
Teresita Muñoz-Antonia
Isabel Muñoz-Barroso
Juan Luis Muñoz-Bellido
R. Munoz-Cano
José A. Muñoz-Cueto
José M. Muñoz-Felix
Gustavo Muñoz-Fernández
Cesar Munoz-Fontela
Violeta Muñoz-Fuentes
Manuel Munoz-Herrera
Angelica Muñoz-Meléndez
Maritza Muñoz-Pareja
Maria Teresa Munoz-Quezada
Miriam Munoz-Rojas
Claudia Munoz-Zanzi
Erin Munro
Mac Munro
Anjana Munshi
Erik Munson
Stefan Münster
Andrea Münsterberg
Andrew Muntean
Shannon Munteanu
Francesco Muntoni
Matthias Munz
Zuzana Münzbergová
Thomas Münzel
Anne-Christine Mupepele
Ivan Mura
Mohammad Hassan Murad
Yoshihiro Muragaki
Koji Murai
Noriyuki Murai
Hiroshi Murakami
Ikuya Murakami
Kenji Murakami
Mario Murakami
Masashi Murakami
Michio Murakami
Tomoaki Murakami
Yoshiki Murakami
Yuki Murakami
Takayuki Murakoshi
Chisako Muramatsu
Giacomo Murana
Eduard Murani
Konstantin Muranov
Pavel Muranski
Wayne Muraoka
Yuki Muraoka
Elena Muraro
Paolo Muraro
Jonathan Muraskas
Mariko Murata
Shigeo Murata
Takatoshi Murata
Takayuki Murata
Claudia Muratori
Moses Muraya
Louisa Murdin
David Murdoch
Giuseppe Murdolo
Dafin Muresanu
Joseph Muretta
Walter Murfee
Harvey Murff
Roberto Murgas

Chris Murgatroyd
Alessandra Murgia
Alessio Murgia
Carla Murgia
Leonardo Murgiano
Juan Murias
R. Murillo
Marina Murillo-Arcila
Juan Murillo-Maldonado
Jeffrey Murley
Kevin Murnane
Bridin Murnion
Andrés Muro
Manuel Muro
Yoshinao Muro
Sergio Murolo
Hiroyuki Murota
Susumu Muroya
Adrianna Murphy
Alex Murphy
Barbara Murphy
Brian Murphy
Carol Murphy
Dean Murphy
Dennis Murphy
Edward Murphy
Erin Murphy
Fionnuala Murphy
John Murphy
Kate Murphy
Kenan Murphy
Michael Murphy
Paddy Murphy
Peter Murphy
Regina Murphy
Richard Murphy
Robert Murphy
Ryan Murphy
Shannon Murphy
Timothy Murphy
Robert Murphy Jr.
Emerson Murphy-Hill
Joanne Murphy-Ullrich
Alan Murray
Alison Murray
Andrea Murray
Brad Murray
Cathryn Murray
Danielle Murray
Deirdre Murray
Emily Murray
Graeme Murray
Grant Murray
Greg Murray
Gregg Murray
James Murray
Joanna Murray
John Murray
Kevin Murray
Kristy Murray
Lyndsay Murray
Paul Murray
Robert Murray
Laura Murray-Kolb
Ebony Murrell
Mora Murri-Pierri
James Murrough
Monzur Murshed
Silvane Murta
Michael Murtaugh
G. Murtaza
Muhammed Murtaza
Amy Murtha
Padma Murthi
Srinivas Murthy
Sriyutha Murthy
Venkatesh Murthy
Lasse Murtomäki
Alejandro Murua
Kadarkarai Murugan
N. Arul Murugan
S. Murugan
Amudhan Murugesan
Ahmed Musa
Michael J Musacchio
Imadidden Musallam
Marcelo Muscará
Beate Muschalla
Moritz Muschaweck
Rashelle Musci
Giovanna Muscogiuri
Evan Muse
Mario Musella
Eustasius Musenge
Philippe Musette
Nick Musi
Massimo Musicco
Erik Musiek
Victor Musiime
Sergey Musikhin
Vijaya Musini
Eri Muso

David Musoke
Dmitry Musolin
Antonino Musolino
Godfrey Musoro
Ferdinando Mussa-Ivaldi
Michele Mussap
Jeffrey Musser
Siegfried Musser
Boris Musset
Margherita Mussi
Andreas Müssigbrodt
Tofy Mussivand
S.M. Mussmann
Steven Mussmann
Abu S. Mustafa
Ahmed Mustafa
Kamal Mustafa
M. Mustafa
Mohd Rais Mustafa
Osama Mustafa
Devkumar Mustafi
Ananda Mustafiz
Mustapha Mustapha
Julie Mustard
Mathias Muszlak
Toshizumi Muta
Vivek Mutalik
Marko Mutanen
Effie S. Mutasa-Göttgens
Carol Mutch
Nicola Mutch
Alexandr Muterko
Günther Muth
Norris Muth
Mehanathan Muthamilarasan
Lucy Mutharia
Gladson Muthian
Jens-Oliver Muthmann
Magesh Muthu
Suresh Muthukumaraswamy
R. Muthuraj
R. Muthurajan
Stella Muthuri
Hariharan Muthusamy
Natarajan Muthusamy
Saminathan Muthusamy
Kerim Mutig
Benjamin Mutin
Apiwat Mutirangura
Onur Mutlu
Unal Mutlu
Evren Mutlugun
Jun Muto
Tatsushi Mutoh
Mutsa Mutowo
Ankur Mutreja
H. Mutsaerts
Nico Mutters
Jasdeep Mutti
Luciano Mutti
Winifred Mutuku
Massy Mutumba
Ephantus Muturi
Philippe Mutwa
Venkateshwar Mutyam
Rüdiger Mutz
Greg Mutze
Kathrin Mutze
Olav Muurlink
Monde Muyoyeta
Otto Muzik
Javier Muzon
Vladimir Muzykantov
Innocenzo Muzzalupo
Mercy Mvundura
Violet Mwaffo
Amos Deogratius Mwaka
Benson Mwangi
Matilu Mwau
Mark Myatt
Elmarie Myburgh
Renier Myburgh
Terence Myckatyn
Laura Mydlarz
Robert Myerburg
Alan Myers
Amanda Myers
Candice Myers
Christopher Myers
Corinne Myers
Jennifer Myers
Kevin Myers
Kristin Myers
Leann Myers
Scott Myers
Timothy Myers
Samuel Myers Jr.
Mary Myerscough
Merle Myerson
Hellen Myezwa
Naja Mygind
Donald L Mykles

Sean Myles
Mathios Mylonakis
Jonathan Mynard
Timo Myohanen
Fumiyoshi Myouga
Angela Myracle
Tyler Myroniuk
Arben Myrta
Knut Myrvold
Barbara Mysona
Indira Mysorekar
Atle Mysterud
Karthikeyan Mythreye
Joanniis Mytilineos
Jihwang Myung
Soon Chul Myung
Marie N'Diaye
Chan-Hyun Na
Dong Na
Il-Kang Na
Muzi Na
Thierry Naas
Kesara Na-Bangchang
S. Masood Nabavizadeh
Mostafa Nabawy
M. Naber
Steffie Naber
Toru Nabika
Joao Nabout
Juliet Nabyonga-Orem
Samia Naccache
Abdeldjallil Naceri
Raffael Nachbagauer
Mathieu Nacher
Senthil Kumar Nachimuthu
Vishnu Varthini Nachimuthu
Paul Nachtigall
Max Nachury
Lorina Naci
Yamama Naciri
Maliwan Naconsie
Philippe Nacry
Altafhusain Nadaf
Chiara Nadai
Ernest Nadal
Jean-Pierre Nadal
Jesús Nadal
Marcos Nadal
Yuji Nadatani
Vincenzo Naddeo
Jay Nadeau
Stephen Nadeau
Sajid Nadeem
Knute Nadelhoffer
Koonlawee Nademanee
Helena Nader
Ingo W. Nader
Nader Nader
Mercader Nadia
Nael Nadif Kasri
Satish Nadig
Nagalakshmi Nadiminty
Sarah Nadimpalli
Jerry Nadler
Celine Nadon
Marc Nadon
Sophie Nadot
Wilson Nadruz Jr.
Sergiy Nadtochiy
Robert Naeije
Lisbeth Naess
Dilip Nag
Tapas Nag
Yukitoshi Nagahara
Maria Nagai
Taku Nagai
Takashi Nagaishi
Jyothi Nagajyothi
Kumaran Nagalingam
Hidetoshi Nagamasu
Masanori Nagamine
Athi N. Naganathan
Seiichi Nagano
Yuriko Nagano
Kyoko Nagao
Norio Nagao
Shoji Nagao
Kentaro Nagaoka
T. Nagaoka
Tadahiro Nagaoka
Mahesh Nagappa
Amruthesh Kestur Nagaraj
Ramaiah Nagaraja
Sharath Nagaraja
Sridevi Nagaraja
T. G. Nagaraja
Tavarekere N. Nagaraja
Niranjan Nagarajan
Priyadharsini Nagarajan
Rajamani Nagarajan
Prabha Nagareddy
Rana Nagarkatti

Kazue Nagasawa
Tasuku Nagasawa
Daisuke Nagata
Jason Nagata
Noriyo Nagata
Tetsuya Nagata
Yukihiro Nagatani
Jiro Nagatomi
Satoshi Nagayama
Mohammed Nagdee
Armin Nagel
Bonnie Nagel
Maria Nagel
Raimund Nagel
Stefan Nagel
Thomas Nagel
Valentin Nagerl
Jurgen Naggert
Susanna Naggie
Chandrashakaharam Nagineni
Maurice Nagington
Elizabeth Nagle
James Nagler
Narihito Nagoshi
Rodney Nagoshi
Tomohisa Nagoshi
B.N. Nagpal
Seema Nagpal
Varun Nagpal
Deepak Nagrath
Elisabeth Nagy
Éva Nagy
István Nagy
László Nagy
Nándor Nagy
Zoltan Nagy
Zoltán T. Nagy
Kyeongah Nah
Maurice Nahabedian
Vinayak Nahar
A. Naheed
Hareth Nahi
Catarina Nahlén Bose
Moon Nahm
Marcos Nahmad
Rooban Nahomi
Azin Nahvijou
Antonella Nai
Saraladevi Naicker
Andrew Naidech
Stanley Naides
Kershney Naidoo
Pren Naidoo
Robin Naidoo
Akhilender Naidu
Rahki Naik
Ram Naikawadi
A. Naiki-Ito
Naim Naim Maalouf
David Naimark
Timothy Naimi
Amrinder Nain
Chandrasekar Nainarpandian
Cho Naing
Ajay Nair
Anil Nair
Dina Nair
Jayasree Nair
Manisha Nair
Meera Nair
Nisanth Nair
P. K. Ramachandran Nair
Rajesh Nair
Rajeshwari Nair
Satish Nair
Sujithkumar Nair
Brittany Nairn
Campbell Nairn
Ramez Nairooz
Robert Naismith
Masanori Naito
Yoshiro Naito
Takeshi Naitoh
Tasnim Najaf
Mehdi Najafzadeh
Kayvan Najarian
Lukáš Najdekr
Ullah Najeeb
Hossein Najmabadi
Kazumi Nakabayashi
Tsutomu Nakada
Susumu Nakae
Hironori Nakagami
Etsuko Nakagami-Yamaguchi
Fumiyo Nakagawa
Kazuo Nakagawa
Kiyotaka Nakagawa
Masanori Nakagawa
Naoki Nakagawa
Osamu Nakagawa
Shigeki Nakagawa
Shin Nakagawa

Shinsuke Nakagawa
Shunsuke Nakagawa
Takahiko Nakagawa
Tsuyoshi Nakagawa
Yoshinori Nakagawa
Shigeki Nakagome
Tsutomu Nakahara
Michiko Nakai
Yousuke Nakai
Peter Nakaji
Katsuyuki Nakajima
Kei Nakajima
Kenichi Nakajima
Kenichiro Nakajima
Motowo Nakajima
Shinichiro Nakajima
Mayuko Nakamaru
Kentaro Nakaminami
Kent Nakamoto
Robert Nakamoto
Shingo Nakamoto
Akihiro Nakamura
Akinobu Nakamura
Fabio Nakamura
Fumio Nakamura
H. Nakamura
Haruki Nakamura
Hitomi Nakamura
Kazufumi Nakamura
Kazuhiko Nakamura
Masato Nakamura
Mashio Nakamura
Michinari Nakamura
Mieko Nakamura
Miho Nakamura
Mio Nakamura
Motoaki Nakamura
Satoki Nakamura
Satoko Nakamura
Shin-ichi Nakamura
Shugo Nakamura
Takashi Nakamura
Takuya Nakamura
Tatsufumi Nakamura
Tomoki Nakamura
Tomomichi Nakamura
Tsutomu Nakamura
Yoshiki Nakamura
Toru Nakanishi
Toshio Nakanishi
Yoshinobu Nakanishi
Damalie Nakanjako
Daisuke Nakano
Eliana Nakano
Koji Nakano
Satoshi Nakano
Tamami Nakano
Atsuhito Nakao
Lia Nakao
Shintaro Nakao
Christos Nakas
Junsuke Nakase
Akio Nakashima
Hiroaki Nakashima
Kazuo Nakashima
Rieko Nakata
Yoshio Nakata
Yoshihisa Nakatani
Hitoshi Nakatogawa
Tetsuya Nakatoh
Yoshimichi Nakatsu
Teruaki Nakatsuji
Haruaki Nakaya
Naoki Nakaya
Tadashi Nakaya
Hideki Nakayama
Jiro Nakayama
Kazuhiro Nakayama
Koji Nakayama
Masaaki Nakayama
Shingo Nakayamada
Takefumi Nakazawa
Toru Nakazawa
Inaam Nakchbandi
Maryam Nakhaei
Hira Nakhasi
Luay Nakhleh
Morad Nakhleh
Ethel Nakimuli Mpungu
Lydia Nakiyingi
Nienke Nakken
Teofil Nakov
Harikrishna Nakshatri
Vamsi J. Nalam
Kodanda Rami Reddy Nalapa Reddy
Luigi Naldi
Allison Naleway
Ma. Jenina Nalipay
Eugene Nalivaiko
Mike Nalls
Jarlath Nally
Nicolas Nalpas

Hojung Nam
Hong Gil Nam
Kyoung Hee Nam
Yunjun Nam
Gautham Namasivayam
Subhalaxmi Nambi
Byju Nambidiyattil Govindan
Sreejith Namboodiri
Vijay Mohan Namboodiri
Dmitry Namgaladze
Ho Namkoong
Amine Namouchi
Hongmei Nan
Weizhi Nan
Gayani Nanayakkara
Daisuke Nanba
Y.V. Nancharaiah
David Nanchen
Vikas Nanda
Jayakrishnan Nandakumar
Saikat Nandi
Shyamal Nandi
Sulakshana Nandi
Vijay Nandula
Sushmita Nandy
Tina Nane
Deepak Nanjappa
Som Nanjappa
Meera Nanjundan
Joaniter Nankabirwa
Toshihiro Nanki
Sara Nannarone
Laura Nanni
Gerrit Nanninga
Rebecca Nantanda
Chanin Nantasenamat
Julie Nantel
Nanda Nanthakumar
Noelina Nantima
Zvi Naor
Marina Naoumkina
Kathrin Näpflin
Bruce Napier
T. Celeste Napier
Thiago Napoleão
Marcelo Napoli
Nicola Napoli
Pietro Napoli
L. Christian Napp
Herbert Nar
Ram Naraian
Satoshi Naramoto
Vivek Naranbhai
Atul Narang
Ritu Narang
A. Naranjo
David Naranjo
Steve Naranjo
Steven Naranjo
Victoria Naranjo
Giri Narasimhan
Shuba Narasimhan
Markus Narath
Gopeshwar Narayan
Prakash Narayan
Prema Narayan
Sanjiv Narayan
Badri Narayanan
Chitra Narayanan
Divya Narayanan
Karthikeyan Narayanan
Nandakumar Narayanan
Sampath Narayanan
Ganesh Narayanasamy
Francesca Nardecchia
Ed Nardell
Enio Nardi
Flavia Domizia Nardi
Serenella Nardi
Claudio Nardiello
Davide Nardo
Valerio Nardone
Simona Nardozza
Ann Nardulli
Anjaparavanda Naren
Nivedita Naresh
Masashi Narita
Vihang Narkar
Steven Narod
Waldemar Narozny
Hema Narra
Georgia Narsavage
Masataka Narukawa
Satoshi Narumi
Kenneth Narva
Darcia Narvaez
Tarun Narwani
Antonio Narzisi
Fabio Nascimbeni
Ana Nascimento
Ana Cristina Nascimento
Daniele Nascimento

Marcos Nascimento
Maristela Nascimento
Rafaella Nascimento
Luiz Eurico Nasciutti
Somia Nasef
Saleh Naser
Abedelmajeed Nasereddin
Benjamin Nash
David Nash
Denis Nash
Kirsty Nash
Madlen Nash
Merinda Nash
Robert Nash
Masayuki Nashimoto
Sonali Nashine
Raad Nashmi
Sazzad Nasir
Mohammad Javad Nasiri
Mohammad Nasiriavanaki
Michael Nasr
Rihab Nasr
Maryam Nasr Esfahani
Fatima Nasrallah
Gheyath Nasrallah
Ilya M. Nasrallah
Lara Nasreddine
Mohammad-Hossein Nasr-Esfahani
Abdelmajid Nassar
Antonio Paulo Nassar Junior
Jeanette Nassif
Fatiha Nassir
Seyed Mahdi Nassiri
Gabriel Nastase
K. Nasu
Tiago Natal-da-Luz
Valerie Natale
Antonino Natalello
Andrea Natali
Charles Natanson
Lisa Natanson
Karaba N. Nataraja
Arutselvan Natarajan
Chandrasekhar Natarajan
Jeyakumar Natarajan
Krishnamurthy Natarajan
Mohan Natarajan
Purushothaman Natarajan
Savithiry Natarajan
Sivaraman Natarajan
Viswanathan Natarajan
Baker Nate
Alexander Nater
Anick Nater
Ricarda Nater-Mewes
Amar Nath
Aritro Nath
Biswajit Nath
Jay Nath
Kavindra Nath
Neetika Nath
Utpal Nath
Lucas Nathan
Sheila Nathan
Andrew Nathanson
Michael Nathanson
Ruvandhi Nathavitharana
Pavan Kumar Nathella
M. Natic
Mario Natiello
Gabriel Natividad
Yasodha Natkunam
Atsushi Natsume
Jacob Nattermann
Romain Nattier
Michael Nauck
Felix Naughton
Ulrike Naumann
Anna Naumova
Shaik Mohammad Naushad
Fauzia Nausheen
Chandra Nautiyal
Prakash Nautiyal
Mohamad Navab
Roya Navab
S. Navakode
Krupa Navalkar
Carryl Navalta
Gabriel Navar
Mirko Navara
Michele Navarra
William Navarre
Eduardo Navarrete
Miguel Navarrete
Eva Maria Navarrete-Muñoz
David Navarro
Ferran Navarro
Isabel Navarro
J.F. Navarro
Monica Navarro
Nicolas Navarro
Noemi Navarro

Pilar Navarro
Raúl Navarro
Rosa Navarro
Samuel Navarro
Sandi Navarro
Lucia Navarro de Lara
Fernando Navarro-Garcia
Nora Navarro-Gonzalez
Juan Navarro-Lopez
Daniel Navarro-Martinez
Adolfo Navarro-Sigüenza
Alfonso Navas
Josè Maria Navas
Juan Navas
Nicolas Navasa
Adrián Naveda-Rodríguez
Manuel Navedo
Babak Navi
Bradford Navia
Ali Navid
Samuele Naviglio
Ami Navon
Nora Navone
Pavla Navratilova
Nicolas Navrot
Tadeusz Nawarycz
Fahim Nawaz
Agnieszka Nawrocka
Ahsan Nawroj
Elizabeth Nawrot
Martin Nawrot
Christian Nawroth
Heinrich Nax
Francisco Naya
Alok Nayak
Arabinda Nayak
Jayakar Nayak
Jennifer Nayak
Ramesh Nayak
Tapan Nayak
Ribhu Nayar
Umit Nayki
Claire Naylor
Gavin Naylor
Justine Naylor
Sadaf Naz
Saeeda Naz
Alison Nazareno
Javad Nazarian
Lusine Nazaryan-Petersen
Mohammad-Reza Nazem-Zadeh
Kim Nazi
Rashid Nazir
Mustafa Naziroglu
Bashoo Naziruddin
A.S.A. Nazri
Pietro Nazzaro
Francesco Nazzi
Albert Ndagijimana
Momar Ndao
Pius Nde
Rawlance Ndejjo
Nicaise Ndembi
Mamadou Ousmane Ndiath
Joseph Ndisang
G. Ndrepepa
Ruth Nduati
Edjah Nduom
Bruce Neal
Joel Neal
Matthew Neal
Matthew D. Neal
Zachary Neal
Patrick Neale
Joshua Nealon
Dimitri Neaux
Linda Neaves
Angela Nebbioso
Alex Nechiporuk
Sarah Necker
Len Neckers
Lucky Nedambale
Aurora Nedelcu
Mathieu Nedelec
Dobrin Nedelkov
Claire Nedellec
Paul Nederkoorn
Christa Nederstigt
Saharnaz Nedjat
Lee Nedkoff
Pavel Nedvetsky
Sriram Neelamegham
Aravind Neelavara Ananthram
Kristina Neely
Melody Neely
Robert Neely
Claire Neema
Matthias Nees
Albrecht Neesse
John Neetu
Christopher Neff
Karl Neff

Kristin Neff
Roni Neff
Axel Neffe
Irina Neganova
Pradeep Singh Negi
Kazuno Negishi
Michiro Negishi
Danilo Neglia
Sonia Negrao
Deborah Negrão-Corrêa
Carlos Negrato
Florence Negre
Nicolas Nègre
Mateus de Oliveira Negreiros
Oscar Negrete
Andrew Negri
Rodolfo Negri
Sylvie Negrier
Daniela Negrini
Stefano Negrini
Anna Negroni
Oana Negru-Subtirica
Deborah Neher
Arye Nehorai
Volker Nehring
Holly Neibergs
Cornelia Neidlinger-Wilke
Pascal Neige
Gretchen Neigh
Amanda Neil
Smart Neil
Tor Neilands
Chris Neill
Andrew Neilson
Julie Neilson
Alexander Neiman
Alexander Neimark
Angelica Neisa
Jay Neitz
Ayna Nejad
Kari Nejak-Bowen
Fatemeh Nejatzadeh-Barandozi
K. Nekaris
Sergei Nekhai
Praveen Nekkar Rao
Ronel Nel
Leif Nelin
Rob Nelissen
Israel Nelken
Barry Nelkin
Wolfgang Nellen
Luca Nelli
Aimee Nelson
Alan Nelson
Andrew Nelson
Andy Nelson
Annmarie Nelson
Arnold Nelson
Brady Nelson
Charles Nelson
Corwin Nelson
Danielle Nelson
David Nelson
David M. Nelson
Erik Nelson
Gregory N. Nelson
Kara Nelson
Kenrad Nelson
Laura Nelson
Mark Nelson
Mike Nelson
Peggy Nelson
Peter Nelson
Randy Nelson
Rebecca Nelson
Richard Nelson
Scott Nelson
Sherry Nelson
Stanley Nelson
Toben Nelson
William Nelson
Karl Emil Nelveg-Kristensen
Houshang Nemati
Mohammad Ali Nematollahi
Alexandr Nemec
Pavel Nemec
Georges Nemer
Charles Nemeroff
Benoit Nemery
Elisa Nemes
Matthias Nemeth
Eiji Nemoto
Tomomi Nemoto
Tooru Nemoto
Mateja Nenadovic
Andrew Nencka
Nuno Nene
Ilona Nenko
Antonio Nenna
Mei Lin Neo
Yan Ning Neo
Charalambos Neophytou

John Neoptolemos
Madhav Nepal
Benjamin Nephew
T. Nepolean
Pablo Nepomnaschy
Juan A. Nepomuceno-Chamorro
Johannes Nepp
A. Neri
Christian Neri
Dario Neri
Francesco Neri
Giovanni Neri
Patrizia Neri
Tauro Neri
Andreas Nerlich
John Nerva
Clara Nervi
Jeanne Nervina
Arthur Nery
Mariana Nery
Ingolf Nes
Amen Ness
Thomas Ness
Steffen Nestler
Marika Nestor
Peter Nestor
Mihai Netea
Chris Netherton
Lauro Neto
Theoden Netoff
Jeniel Nett
Dan Nettleton
Regina Netto
Yael Netz
Corey Neu
Andreas Neubauer
Henning Neubauer
Thomas Neubauer
Lis Neubeck
Arthur Neuberger
John Neubert
Reinhard Neubert
Oliver Neudert
Stefan Neuenschwander
Alfred Neugut
Nina Neuhaus
Peter Neuhaus
Duncan Neuhauser
Anna Neuheimer
John Neuhoff
Jeffrey Neul
Eva Neumaier-Probst
Yair Neuman
Gabriele Neumann
Heiko Neumann
Heinz Neumann
Inga Neumann
Jens Neumann
Johannes Neumann
Kerstin Neumann
Markus Neumann
Roland Neumann
Wolf-Julian Neumann
William Neumeier
Bruno Neuner
Binod Neupane
Subas Neupane
Danielle Neut
Cécile Neuvéglise
Jaakko Nevalainen
Paul Neve
Richard Neve
Adriana Neves
Elizabeth Neves
Frederico Neves
Josiane Neves
Maria Helena Neves
Helene Neville
Remington Nevin
Hannah Nevins
Cynthia Nevison
Jason Newbern
Rodney D. Newberry
Carrie Newbold
Sarah Newbury
Gretchen Newby
Jill Newby
L. Kristin Newby
Dawn Newcomb
James Newcomb
George Newcombe
Peter Newcombe
Marie-Louise Newell
Annie Newell-Fugate
Scott Newey
Di Newham
James Newham
Christina Newhill
Ian Newhouse
Jessica Newlin
Aaron Newman
Christopher Newman

Dianne Newman
Eric Newman
Greg Newman
Jennifer Newman
John Newman
Jonathan Newman
Mari-Anne Newman
Molli Newman
Robert Newman
Roger Newman
Roger Newman-Norlund
David Newman-Toker
Roger Newport
David Newsham
Mary Newsome
Timothy Newsome
Allen Newton
Hayley Newton
Irene Newton
Kelly Newton
Michael Newton
Peter Newton
Tim Newton
Ebba Nexo
Denise Ney
Samuel Neymotin
Helene Neyret-Kahn
Levent Neyse
A. Sanati Nezhad
Katerina Nezvalová-Henriksen
Charles Nfon
Adeline S.L. Ng
Annalyn Ng
Austin Chin Chwan Ng
Benedict Ng
Benjamin Ng
Carmina Ng
Caroline Ng
Chee Ng
Chin Ng
Chong Guan Ng
Colin Ng
Danny Ng
Davis Ng
Eric Ng
Felix Ng
Heok Hee Ng
Jason Ng
Kevin Tak-Pan Ng
Kwan Ng
Lauren Ng
Manwa Ng
Norman Ng
Peter Ng
Raymond Ng
Shu-Hang Ng
Sophia Ng
Terry Ng
Valerie Ng
Victoria Ng
Wan-Fai Ng
Wei Ng
Sai-Ming Ngai
H.W. Ngan
Tiia Ngandu
Umakanta Ngangkham
R. Ngara
Matilda Ngarina
Nlandu Ngatu
Joanne Ngeow
Oscar Ngesa
Anaclet Ngezahayo
Phioanh Nghiemphu
Nicole Ngo
Van Ngo
Jeremiah Ngondi
Corine Ngufor
Anthony Ngugi
John Ngunjiri
Kenneth Ngure
Anh Thi Van Nguyen
Anthony Nguyen
Bich Nguyen
Daniel Nguyen
Hai Nguyen
Hoang Nguyen
Hue Nguyen
Hung Nguyen
Huong Nguyen
Jennifer Nguyen
Khoa Nguyen
Lam Son Nguyen
Linh Nguyen
M. Hong Nguyen
Mindie Nguyen
Nam-Phuong Nguyen
Nguyen Hong Nguyen
Nha Nguyen
Patricia Nguyen
Phuong Nguyen
Quan Nguyen
Quang Nguyen

Tam Nguyen
Thanh Nguyen
Thao Nguyen
Thi Bich Ngoc Nguyen
Thi-Phuong-Lan Nguyen
Tho Nguyen
Tung Nguyen
Ut Nguyen
Alfred Ngwira
Ngoc Nham
Aquino Nhantumbo
Joo Young Nho
Andy Ni
Bing Ni
Cheng Ni
Heyu Ni
Huangjing Ni
Liqiang Ni
Michael Ni
Zhen Ni
Zhongfu Ni
Naz Niamul Islam
Hui Nian
Ali Niazi
Javed Niazi
Nathan Nibbelink
Emanuele Nicastri
Giampaolo Niccoli
Chris Nice
Timothy Nice
Siobhan Nichoileain
Frank Nicholas
Jennifer Nicholas
Richard Nicholas
Robin Nicholas
Barbara Nicholl
Stuart Nicholls
Tonia Nicholls
Austin Nichols
Benjamin Nichols
Charles Nichols
Frank Nichols
James Nichols
Jeremy Nichols
Joan Nichols
Krista Nichols
Michael Nichols
Timothy Nichols
Caroline Nicholson
Dan Nicholson
Emily Nicholson
James Nicholson
Linda Nicholson
Maribeth Nicholson
Wayne Nicholson
Harry Nick
Bert Nickel
Christian Nickel
Jason Nickell
Robert Nickells
Martin Nickels
Stefanie Nickels
Georg Nickenig
Kenneth W Nickerson
D. Nickla
Stuart Nicklin
Thomas Nickolas
Daniele Nico
Alcina Nicol
Grant Nicol
Stephen Nicol
Steve Nicol
André Nicola
Anthony Nicola
Massimo Nicola
Alexandru Nicolae
Herisanu Nicolae
Gerry Nicolaes
Nicolas Nicolaides
Theodore Nicolaides
Ove Nicolaisen
Gaël Nicolas
Guillaume Nicolas
Ezequiel Nicolazzi
Bruna Nicoletto
Andrea Nicolini
Francesco Nicolini
Franck Nicolini
Teresa Nicolson
Roberto Nicosia
Vincenzo Nicosia
Franck Nicoud
Helene Niculita-Hirzel
Thibault Nidelet
Bernd Nidetzky
Aiqing Nie
Guangjun Nie
Guiying Nie
Jing Nie
Jingxin Nie
Pin Nie
Qian Nie

Qinghua Nie
Yaohui Nie
Yuqiang Nie
Zuoming Nie
Karen Nieber
Glen Niebur
Andreas Nieder
Ellen Niederberger
Daniel Niederer
Simone Niederhäuser
Laura Niedernhofer
David Niederseer
Harald Niederstätter
Matthias Niedrig
Inga Niedtfeld
Michelle Niedziela
Beeri Niego
James Nieh
Diederick Niehorster
Christof Niehrs
Manel Niell
Brent Nielsen
Claus Nielsen
Clayton Nielsen
Emil Nielsen
Finn Nielsen
Heber Nielsen
Henrik Nielsen
Jens Nielsen
Kaare Nielsen
Liza Nielsen
Mark Nielsen
Nete Munk Nielsen
Shaun Nielsen
Søren Nielsen
Tommy Nielsen
Christina Nielsen-Leroux
Anne Nielson
Jessica Nielson
Ryan Nielson
Torsten Nielson
Marvin Nieman
Markus Niemann
Ulrich Niemann
Hermann Niemeyer
Jarad Niemi
Matthew Niemiller
Anna-Liisa Nieminen
Jaakko Nieminen
Juha Niemistö
Neal Niemuth
Aleksandra Niemyjska
Sandra Niendorf
Albert Nienhaus
Eberhard Nieschlag
Judith Niesen
Carien Niessen
Wiro Niessen
Gema Nieto
Nathan Nieto
Ramfis Nieto-Martínez
Jose M. Nieto-Villar
Natalie Nieuwenhuizen
Pythia Nieuwkerk
Helene Nieuwoudt
Caroline Nievergelt
Stefan Niewiesk
James Nifong
Yeshambul Nigatu
Joel Nigg
Hugo J. Niggli
Chandylen Nightingale
Sheila Nightingale
Cecilia Nigro
Giovanna Nigro
Lisa Nigro
Steven Nigro
Caetano Nigro Neto
Sagar Nigwekar
Susumu Nii
Takahiro Niida
Tomiharu Niida
Hiroyuki Niihara
Mamoru Niikura
Tim Niiler
Yoshihito Niimura
Kristjan Niitepõld
Tomihisa Niitsu
Ank Nijhawan
Ard Nijhof
Reindert Nijland
Ivan Nijs
E.R. Nijsse
Spyros Nikas
Pablo Nikel
Etsuo Niki
Nikos Nikiforakis
Mikko Nikinmaa
Jana Nikitin
Anastasia Nikitina
Mehdi Nikkhah
Martin Niklas

Laura Niklason
Johan Niklasson
Alexander Nikolaev
Eugene Nikolaev
Sergey Nikolaev
Pantelis G. Nikolakopoulos
Chistoforos Nikolaou
Molly Nikolas
Basil Nikolau
Dragana Nikolic
Mikeljon Nikolich
Zoran Nikoloski
Anna Nikonova
Raisa Nikula
Alexey Nikulin
Kjell Nikus
Kanishka Nilaweera
Richard Niles
Kayzad Nilgiriwala
Charles Nilon
Frank Nilsen
Kristian Bernhard Nilsen
Ola Nilsen
Anders Nilsson
Erik Nilsson
Henrik Nilsson
Lars Nilsson
Markus Nilsson
Melanie R Nilsson
Kristina Nilsson Ekdahl
Johan Nilvebrant
Stephen Nimer
Shahid Nimjee
Akimoto Nimura
Andrea Ninfo
Chuanyi Ning
Daliang Ning
Huan Ning
Kang Ning
Shunbin Ning
Xia Ning
Yongzhong Ning
Zhangyong Ning
Shira Ninio
Elina Nino
David Nipperess
Eyal Nir
S.R. Niranjana
Henry Nisell
Hiroshi Nishi
Shawn Nishi
Yutaro Nishi
Toshiya Nishibe
Gohei Nishibuchi
Atsushi Nishida
Kohji Nishida
Motohiro Nishida
Sachiko Nishida
Akihiro Nishiguchi
Michele Nishiguchi
Ryuichi Nishii
Kazutoshi Nishijima
Jun Nishikawa
Hiroshi Nishimune
Goro Nishimura
M. Nishimura
Noriyuki Nishimura
Reiki Nishimura
Tomoko Nishimura
Yukihide Nishimura
Adrienne Nishina
Hiroshi Nishina
Takashi Nishina
Ichizo Nishino
Kunihiko Nishino
Mizuki Nishino
Mizuho Nishio
Shin-ya Nishio
Shinta Nishioka
Hideki Nishitoh
Takumi Nishiuchi
Junko Nishiwaki
Chiharu Nishiyama
Hiroshi Nishiyama
Masahiko Nishiyama
Shoko Nishiyama
Hitoshi Nishizawa
Alina Niskanen
Corey Nislow
Lisa Nissen
Andrea Nistri
Kevin Niswender
John Nitiss
Lucio Nitsch
Craig Nitschke
Anjana Nityanandam
Bjoern Nitzsche
Franziska Nitzsche
Gang Niu
JianJun Niu
Jingwen Niu
Jinzhi Niu

Junqi Niu
Liang Niu
Ongge Niu
Suyan Niu
Tianhui Niu
Tianye Niu
Wenquan Niu
Wenxin Niu
Xi Niu
Xinxin Niu
Yaron Niv
Lisa Nivison-Smith
Brett Nixon
Mark Nixon
Sophie Nixon
Christopher Niyibizi
Vandana Niyyar
Ahamed Nizam
Sheikh Nizamuddin
Nizar Nizar
Antonio Nizza
Sylvestre Njakou Djomo
Jackson Njau
M. Njenga
Emmanuel Njeuhmeli
Branimir Njezic
Dolores Njoku
Maureen Njue
Njambi Njuguna
Céline Nkenfou
Jaqcueline Nkhoma
Kabwe Nkongolo
Esther Nkuipou Kenfack
Marcel Nkuize
Jerome Noailly
Angela Nobbs
Clarissa Nobile
Flavio Nobili
Anaïs Noblanc
Charleston Noble
Linda Noble
Luke Noble
Rachel Noble
William Noble
Ligia Nobre
Franklin Nobrega
Giuseppe Nocella
Annalura Nocentini
Joe Nocera
Luca Nocetti
Tomonori Nochi
Kotaro Nochioka
Matthew Nock
Akiko Noda
Mami Noda
Takuji Noda
Koichi Node
Justin Nodwell
Francesco Noè
Alexandra Noel
Richard Noel Jr.
Heidi Noels
Andre Noest
Haruo Nogami
Eishi Noguchi
Emiko Noguchi
Hiroshi Noguchi
Hitoshi Noguchi
Ko Noguchi
Masayuki Noguchi
Satoru Noguchi
Takako Noguchi
Y Noguchi
Yasuki Noguchi
Yoshihiro Noguchi
Claudio Nogueira
Fatima Nogueira
Maurício Nogueira
Miguel Nogueira
Waldo Nogueira
Guilherme Nogueira-Neto
Pablo Nogues
Gyu-Jeong Noh
Hyunjin Noh
Jihyun Noh
MiJin Noh
Sung Noh
Anja Nohe
Amit Noheria
Quentin Noirhomme
Eisei Noiri
Ossi Nokelainen
Miriam Nokia
Erica Nol
Fiona Nolan
Francis Nolan
Jessica Nolan
Laura Nolan
Seonaid Nolan
Stefano Nolfi
Markus Noll
Ellen Nollen

Martin Nollenburg
Harry Noller
Michael Noll-Hussong
Elke Nolte
Sandra Nolte
Kensuke Noma
Hidetaka Nomaki
Cristina Nombela
Josep Nomdedeu
Jason Nomi
A. Nomura
Masatoshi Nomura
Shosaku Nomura
Takeshi Nomura
Yasuyuki Nomura
Satoko Nonaka
Michel Nonent
Yibing Nong
Maria Noni
Larisa Nonn
Michael Nonnemacher
Ken-Ichi Nonomura
Justice Nonvignon
Curtis Noonan
Douglas Noonan
Vanessa Noonan
Chadanat Noonin
Forhana Rahman Noor
Khalida Inayat Noor
Mohamed Noor
Syed Noor
M. Noordzij
Sander Noort
Sunil Nooti
Ursula Nopp-Mayr
Khairun Nain Nor Aripin
Nurshamimi Nor Rashid
Gray Nora
Fernando Norambuena
Teresa Norat
Dan Norbäck
Johannes Norberg
Margareta Norberg
Catarina Nordander
Lars Nordenmark
Maria Nordfang
Anna Synnøve Røstad Nordgard
Tara Nordgren
Verena Nordhoff
Robert Nordon
Rebecca Nordquist
Daniela Nordzieke
Jon Norenburg
Mette Nørgaard
Julian Norghauer
David Noriega
Tsuru Noriko
Suzuki Noriyuki
Joanna Norkko
Barbara Norman
Janette Norman
Kristina Norman
Paul Norman
Philippe Normand
David Normando
Simona Normando
Nicola Normanno
Benjamin Normark
Henrique Noronha
Santosh Noronha
Marina Noronha Ferraz de Arruda Colli
Rikke Norregaard
Gill Norris
Laura Norris
Michelle Norris
Pauline Norris
Steven Norris
Grant Norte
Ace North
Rachel North
Diana Northup
Joelle Nortier
Gareth Nortje
Gavin Norton
George Norton
Jeanette Norton
Jeffrey Norton
Michael I. Norton
Pamela Norton
Samuel Norton
William Norton
Justice Norvienyeku
Ken Nosaka
Andrew Nosal
Artur Nosalewicz
Joshua Nosanchuk
Chiara Nosarti
Rodrigo Noseda
Michael Noseworthy
Sergei Noskov
Andrew Noss
Ralph Nossal

Marika Nosten-Bertrand
Bohdan Nosyk
Michael Notaro
Wim Notebaert
Justin Notestein
Warren Nothnick
Eduardo Notivol
Davide Noto
Shoji Notomi
Caroline Nott
Ill-Sup Nou
Xiangwu Nou
Vincent Nougier
Semir Nouira
Anastasios Noulas
Mazen Noureddin
Mohammad-Zaman Nouri
Mehdi Nourozi Nourozi
Mahdad Noursadeghi
Deborah Novack
Victor Novack
Elizabeth Novak
Jiri Novak
Natalija Novak
Richard Novak
Julia Novak Colwell
Fernando Novas
Andrea Nove
Antonio Novelli
Lucia Novelli
Giovanni Novembre
Mairi Noverr
Gina Novick
Peter Novick
Richard Novick
Dmitry Novikov
M. Lelinneth (Len) Novilla
Chad Novince
Marta Novo
Pedro Novo
Shenia Novo
Ana Novoa
Alexey A. Novoselov
Moritz Nowack
Andrzej Nowak
Carsten Nowak
Glen Nowak
Renata Nowak
Robert Nowak
Roman Nowak
Romana Nowak
Stefan Nowak
Thaddeus Nowak
Monika Nowak-Imialek
Adam Nowakowski
Alexandra Nowakowski
Andrzej Nowakowski
Khalid Nowar
Katarzyna Nowicka-Sauer
Bogdan Nowicki
Cristina Nowicki
Piotr Nowicki
Cindy Nowinski
Krzysztof Nowosielski
Norbert Nowotny
Ariela Noy
Ilan Noy
Noa Noy
Genevieve Noyce
Noelle Noyes
Andrew Noymer
Marilyn Noz
Miho Nozaki
Sylvie Nozaradan
Ala Nozari
Hajime Nozawa
Susan Nozell
Tomoko Nozoye
Kandai Nozu
Silvia Nozza
Sabin Nsanzimana
Célestin Ndosimao Nsibu
George Ntaios
Jackson Nteeba
Vardis Ntoukakis
Francis Ntumngia
Paolo Nucci
Michael Nuccio
Richard Nuccitelli
Carmelo Nucera
Jürg Nuesch
Rolf Nuesing
Alan Nugent
Jim Nugent
Michael Nugent
Nicole Nugent
Mark Nuijten
Hitoshi Nukada
R. Nulit
Amit Nulkar
Kazuyuki Numakura
Keiji Numata

Alexandra Nunes
Altacílio Nunes
Bruno Nunes
Manoel Nunes
Maria Nunes
Vania Nunes
Ulisses Nunes da Rocha
Lígia Nunes de Morais Ribeiro
Adriano Nunes-Nesi
Caroline Nunes-Xavier
Alfredo Nunez
Antonio Nunez
Silvia Nunez
Cristina Núñez
José Núñez
Marco T. Núñez
Juan Núñez-Farfán
Ivan Nunez-Gill
Charles Nunn
Joanne Nunnerley
Leonard Nunney
Juan Carlos Nuño
Natalia Nuño
Takuro Nunoura
Kulmira Nurgali
Alessandra Nurisso
Gertrud K. Nürnberg
Yuana Nurulita
Dmitri Nusinow
Asma Nusrat
Robert Nussbaum
Thomas Nussbaumer
Ruth Nussinov
Stephen Nutt
Alfred Nuttall
Amy Nuttall
James Nuttall
Kristo Nuutila
Iman Nuwayhid
Anne Monique Nuyt
Regina Nuzzo
Chike Nwabuo
Tibor Nyari
Árpád Nyári
Anna Nyberg
Laura Nyblade
Hilde Nybom
Karine Nyborg
Janet Nye
Henrik Nygård
Brendan Nyhan
Erika Nyhus
Philip Nyhus
Sylvia Nyilas
Ildiko Nyilasi
Miklos Nyitrai
László Nyitray
Ivan Nyklicek
Karin Nylander
Susanne Nylen
Göran Nylund
Andy Nyman
Charles Nyman
Jeffry Nyman
Paul Nyquist
Johanna Nystedt
Fredrik Nystrom
Lennarth Nyström
Justine Nzweundji
Manoj Oak
Christopher Oakes
Jessica Oakes
Patrick Oakes
Scott Oakes
Berl Oakley
Brian Oakley
Miranda Oakley
Megan Oaten
James Oates
Thomas Oates
Desmond Oathes
Jon Oatley
Jeremy Oats
Victor Obach
Akira Obana
Joshua Obar
Boguslaw Obara
Isaiah Obara
Marcos Obara
Taku Obara
Keisuke Obase
Alvaro J. Obaya
Darren Obbard
Glen Obear
Rima Obeid
Anna C. Obenauf
Holly Ober
Raimund Ober
Martin Oberbarnscheidt
Anita Oberbauer
Daniel Oberfeld
Cary Oberije

T.F. Oberlander
Barbara Obermayer-Pietsch
H.S. Oberoi
Steve Oberste
Timm Oberwahrenbrock
Tatiana Oberyszyn
Elisabeth Oberzaucher
Katja Obieglo
Walter Obiero
Alex Obinikpo
Christian Obirikorang
Elia Obis
Paula Oblessuc
Biel Obrador
Natalie Obrecht
Carolina Obregon
Paulo Obreli-Neto
T. Obreza
Alastair O'Brien
Colm O'Brien
Emily O'Brien
Erin O'Brien
Fergal O'Brien
John O'Brien
Joseph O'Brien
Kevin O'Brien
Kimberly O'Brien
Louise O'Brien
Maureen O'Brien
Meagan O'Brien
Oliver O'Brien
Oonagh O'Brien
Robert O'Brien
Sarah O'Brien
Travis O'Brien
Bill O'Brien-Penney
Neil O'Brien-Simpson
Karl Obrietan
Martin Obrist
John O'Bryan
Moira O'Bryan
David O'Callaghan
Emma Kate O'Callaghan
Joanne Michelle Ocampo
Alberto Ocaña
Paola Occhetta
Alejandro Oceguera-Figueroa
Anna Ochab-Marcinek
Oche Oche
Begoña Ochoa
Francisco Ochoa-Corona
Olga Ochoa-Gondar
Emmanuel Ochola
Ira Ockene
David O'Connell
Grace O'Connell
Kristen O'Connell
Mary O'Connell
Ryan O'Connell
Anna O'Connor
Benjamin O'Connor
Brendan O'Connor
Christine O'Connor
Daniel O'Connor
David O'Connor
James O'Connor
Jason O'Connor
John O'Connor
Kathleen O'Connor
Kristian O'Connor
Mairead O'Connor
Nancy O'Connor
Patrick O'Connor
Peter O'Connor
Shelby O'Connor
Tamara O'Connor
Eiji Oda
Gisele Oda
Yasuhiro Oda
Patrick Odawo
Francesco Oddone
Hirotaka Ode
Eamon O'Dea
Kieran O'Dea
Anders Ödeen
James Odei
Adam Odell
Katja Odening
Helena Ödesjö
Emmanuel Odic
Monica Odlare
Jack Odle
George O'Doherty
James Odom
Robert Odom
Stephen Odom
Alison O'Donnell
Anne O'Donnell
Colin O'Donnell
Ryan O'Donnell
Greg Odorizzi
Mike O'Driscoll

Nelson O'Driscoll
Ayodele Odutayo
Olabisi Oduwole
Patrick J. O'Dwyer
Walt Oechel
Ketil Oedegaard
Stefan Oehler
Maren Oelbermann
Anika Oellrich
Oene Oenema
Erich-Christian Oerke
Ken Oestreich
Jordan Oestreicher
William Oetting
Orit Oettinger-Barak
Jaap Oever
Noa Ofen
Vittoria Offeddu
Andreas Offenhausser
Dietmar Offenhuber
Erika Offerdahl
Bernard Offmann
Dana Ofiteru
Martin O'Flaherty
Sarah O'Flaherty
Bahadorreza Ofoghi
Sandra Ofori
Ighovwerha Ofotokun
Enoche Oga
Toru Oga
Vasily Oganesyan
James O'Gara
Anne O'Garra
Tanya O'Garra
Sadahisa Ogasawara
Atsushi Ogata
Toshiyasu Ogata
Aiko Ogawa
Rei Ogawa
Susumu Ogawa
T. Ogawa
Yoko Ogawa
Romà Ogaya
Francis Ogbonnaya
Elizabeth Ogburn
Bryan Ogden
Nicholas Ogden
Rowan Ogeil
Javier Ogembo
Robert Ogg
Mitsunori Ogihara
Gina Ogilvie
Shuji Ogino
Soichi Ogishima
Satoshi Ogiso
Bjorn Oglaend
Graham Ogle
Amanda Oglesby-Sherrouse
Mylène Ogliastro
Haluk Ögmen
Adam Ogna
Takeshi Ogo
Martin Ogonowski
Jernej Ogorevc
David O'Gorman
Donal O'Gorman
Jimmy O'Gorman
Sven Ove Ögren
Rajna Ogrin
Taichi Oguchi
Emeka Oguejiofor
Adesola Ogunfowokan
Ifedayo Ogungbe
Benson Ogunjimi
Atsushi Ogura
Hideki Ogura
Yuji Ogura
Ezgi Ogutcen
Joseph Ogutu
Chang Seok Oh
Chang Sik Oh
Ding Yuan Oh
Dong-Chan Oh
Doo-Byoung Oh
Edwin Oh
Esther Oh
Eun Joong Oh
Goo Taeg Oh
Hyunsung Oh
Il-Hoan Oh
Jong-Ryool Oh
Juhwan Oh
Kook-Hwan Oh
Minyoung Oh
Myoung-Don Oh
Sae-Ock Oh
Sang-Yun Oh
Se-Hong Oh
Sei Oh
Seieun Oh
Sejong Oh
Seunghan Oh

Yu-Kyoung Oh
Justin O'Hagan
Ken O'Halloran
Siobhan O'Halloran
Mamiko Ohara
Naoya Ohara
Caitlin O'Hara
Kevin O'Hara
Ross O'Hara
Steven O'Hara
Mica Ohara-Imaizumi
Helen O'Hare
Finbarr O'Harte
Jun Ohashi
Kazuharu Ohashi
Kazutomo Ohashi
Koji Ohashi
Kyoko Ohashi
Fredrik Öhberg
Kenji Ohgane
Kazutaka Ohi
Ryoma Ohi
Akhiro Ohira
Masaichi Ohira
Mitsuru Ohishi
Noriaki Ohkawa
Nobuhiro Ohkohchi
Takayoshi Ohkubo
Siew-Wan Ohl
Sven Ohl
Jan Ohlberger
Kevin Ohlemiller
Jennifer Ohlendorf
Annemarie Ohler
Uwe Ohler
Andreas Ohlin
Mats Ohlin
Carsten Ohlmann
Knut Ohlsen
Henrik Ohlsson
Robin Ohm
Dennis Ohman
Makoto Ohmoto
Osamu Ohneda
Koji Ohnishi
Seiko Ohno
Richard Ohrbach
Soren Ohrt-Nissen
Hisaji Ohshima
Takayuki Ohshima
Yusei Ohshima
Shinichiro Ohshimo
Jun Ohta
Yasutaka Ohta
Misato Ohtani
Toshiyuki Ohtani
Susumu Ohtsuka
Sumio Ohtsuki
Eric Ohuma
Yoshinari Ohwaki
Yusuke Ohya
Hideaki Oike
Masahiro Oike
Vesa Oikonen
Georgios Oikonomou
Akio Oishi
Hisashi Oishi
Shinya Oishi
Dario Ojeda
Francisco Ojeda
Norma Ojeda
Anil Ojha
Heidi Ojha
Suvash Ojha
Takao Ojima
Ayako Oka
Shinichi Oka
Kimiko Okabe
Yoshihiro Okabe
Hirokazu Okada
Kazunori Okada
Morihito Okada
Shinji Okada
Toshiyuki Okada
Yohei Okada
Yukinori Okada
Chika Okafor
Katsunori Okajima
Yuko Okamatsu-Ogura
Hiroshi Okamoto
Kunio Okamoto
Maristela Okamoto
Nobuhiko Okamoto
Ryuichi Okamoto
Shigefumi Okamoto
K. Okamoto-Mizuno
Tomonori Okamura
Yasushi Okamura
Yukiyasu Okamura
Cahir O'Kane
Sharon Okanga
Masanori Okanishi

Sezer Okay
Ryuichi Okayasu
Kazuichi Okazaki
Ken Okazaki
Shigetoshi Okazaki
Richard O'Kearney
F. Robin O'Keefe
Lannie O'Keefe
Meredith O'Keeffe
Thomas Okey
Eiji Oki
Yasuhiro Oki
Noriaki Okimoto
Klaus Okkenhaug
Chizimuzo Okoli
Uduak Okomo
Joshua Okonya
Michael Okorie
Andrei Okorokov
Marina Okoshi
Atte Oksanen
Ole Andreas Økstad
Ozgur Oktem
Hidehiro Oku
Hiromi Oku
Kenji Oku
Akihiko Okuda
Yoshinaga Okugawa
Mobolaji Okulate
Sakiko Okumoto
Kenji Okumura
Satoshi Okumura
Takahiro Okumura
Yasuo Okumura
Michael Okun
Christopher Okunseri
Takafumi Okura
Toru Okuyama
Derick Okwan-Duodu
Ifeoma Okwor
Maite Olaciregui
Andrew Olagunju
Iñigo Olalde
Miguel Olalla-Tárraga
Wayne Olan
Lydia Olander
Funmilola OlaOlorun
Banky Olatosi
Richard Olawoyin
Sebastian Olbrich
Natasha Olby
James Olcese
Umberto Olcese
Vanessa Olcese
Johan Oldekop
Catherine Oldenburg
Joppe Oldenburg
Sally Olderbak
Paul Oldham
William Oldham
Ben Oldroyd
Brett Olds
Tim Olds
Pedro Olea
Alvaro Oleaga
Francisco Olea-Popelka
Daniel O'Leary
Heather O'Leary
John O'Leary
Mary O'Leary
Nora Oleas
Natalia Oleinik
Mikolaj Olejniczak
Anna Olejnik
Malgorzata Olejnik
Ola Olen
Balaji Olety
I. Olfert
Ivan Olfert
Emily Olfson
Natalie Olifiers
Pierpaolo Olimpieri
Michael Olin
Tom Olino
Ryszard Olinski
Kevin Olival
Pilar Olivar
Andy Olivares
Christopher Olivares
Felipe Olivares
Manuel Olivares
Rene Olivares-Navarrete
Aires Oliva-Teles
M. Foster Olive
Sébastien Olive
Irene Olivé
A. Sofia Oliveira
André Oliveira
Andreia Oliveira
Camila Oliveira
Carla Oliveira
Claudio Oliveira

Cleida Oliveira
Cristieli Oliveira
Djenane Oliveira
Eugênio Oliveira
Fabiano Oliveira
Fabrício Oliveira
Fernando Oliveira
Giancarlo Conde Xavier Oliveira
Guilherme Oliveira
Halley Oliveira
J.T.A. Oliveira
Jaquelline Oliveira
Jhones Oliveira
José Luis Oliveira
Karliana Oliveira
Katia Oliveira
Marcos Oliveira
Marcus Oliveira
Maria Oliveira
Maria Leonor Sarno Oliveira
Norton Luis Oliveira
Pedro Oliveira
Raquel Oliveira
Rui Oliveira
Sofia Oliveira
Vanessa Oliveira
Pedro Paulo Oliveira Jr.
João Luís Oliveira-Carvalho
Joao Paulo Oliveira-Costa
Anthony Oliver
Antonio Oliver
Bonamy Oliver
Brian Oliver
Haley Oliver
Jonathan Oliver
Jose Oliver
Melvin J. Oliver
Nick Oliver
P. Graham Oliver
William Oliver
Antonio Olivera-La Rosa
Jesus Olivero-Verbel
Antonio Oliver-Roig
Chris Olivers
Alicia Olivier
Christoph Olivier
Jill Olivier
Magali Olivier
Martin Olivier
Nicolas Olivier
Alejandro Olivieri
Anna Olivieri
Carla Olivieri
Antonio Oliviero
Alfonso Olivos-García
Jean-Marc Olivot
Jacquie Oliwa
Maria Olkkonen
Henri Olkoniemi
B.O. Olley
Charlotta Olofsson
Jennifer Olori
Samantha O'Loughlin
Samuel Olowookere
Alexander Olsen
Anna Olsen
Erik Olsen
Jeanine Olsen
Jorn Olsen
Margaret Olsen
Michael Olsen
Michelle Olsen
Nora Olsen
Odd-Arne Olsen
Richard Olsen
Robert Olsen
Sandra Olsen
Sonja Olsen
Steven C. Olsen
Yulia Olshanskaya
Richard Olshen
David Olson
Eric Olson
Jonathan Olson
Jordan Olson
Kenneth Olson
Lorin Olson
Mary Olson
Matthew Olson
Michael Olson
Randal Olson
Sara Olson
Steven Olson
Jens Olsson
Rene Olsthoorn
Deborah Olszewski
Horatiu Olteanu
Allard Olthof
Margreet Olthof
Elisa Oltra
Olushayo Olu
Bode Olukolu

Aria F. Olumi
Bolajoko Olusanya
Bolutife Olusanya
Oluyinka Olutoye
Fasina Oluyemi
Heidi Olze
Yosuke Omae
K. Omagari
Liam O'Mahony
Siobhain O'Mahony
Paul O'Maille
Dervla O'Malley
Ronan O'Malley
Dario Omanovic
Daibhid O'Maoileidigh
M. O'Mara
Takeshi Omasa
Kazumi Omata
Perera Omaththage
Michael Ombrello
Gilbert Omenn
Jeff Omens
Diana Omigie
Elisa Omodei
Bernard Omolo
George Omondi
Lanre Omotayo
Celso Omoto
Dalia Omran
Anders Omsland
Ahmet Omurtag
Geoffrey Omuse
Olusegun Onabajo
Nobuyuki Onai
Mark Onaitis
Cagdas Onal
Cem Onal
Aytug Onan
Altan Onat
Patrick Onck
Paola Ondarza
Graziano Onder
Lucas Onder
Andrew Onderdonk
Pascale Ondoa
Brian Ondov
Vladan Ondrej
Nancy Ondrusek
Wanda O'Neal
Wesley O'Neal
Dion O'Neale
F.G. (Barry) O'Neil
Alex O'Neill
Charles O'Neill
Eric O'Neill
Hester O'Neill
Ian O'Neill
Jennifer O'Neill
Liam O'Neill
Sandra O'Neill
Stephen O'Neill
Chin Siang Ong
Han Kiat Ong
Hui Xin Ong
Irene Ong
Janus Ong
Jennifer Ong
Lee-Ling Ong
Pei-Shi Ong
Peter Ong
Qunya Ong
John Ong'echa
Francesco Onida
Lucy Onime
Gabriel Oniscu
Maurizio Onisto
Anna Oniszczuk
Itay Onn
Lim Onn
Atsushi Ono
Koh Ono
Kotaro Ono
Takahiro Ono
Yusuke Ono
Yusuke Onoda
Osamu Onodera
Silvano Onofri
Akio Onogi
Anyebe Onoja
Sam Ononge
Dorothy Ononokpono
IfeanyiChukwu Onor
Kenji Onoue
Ikushi Onozaki
Todd Ontl
Alexey Onufriev
Ezenwa Onyemata
Alphonsus Onyiriuka
Akishi Ooi
Geraldine Ooi
Keith Ooi
Kenneth Ooi

Charlotte Oomen
Antonius Oomens
Chris Oostenbrink
Mark Oostergo
Peter Oosterveer
Mariska Oosterveld-Vlug
Egbert Oosterwijk
Agnieszka Opala-Berdzik
Bertram Opalka
Juan Opazo
Ghislain Opdenakker
Brent Opell
Stanley Opella
Joseph Opferman
Roel Ophoff
George Opie
George Opit
Alexander Opitz
Bastian Opitz
Stephen Opiyo
Christian Opländer
Mark Opler
Onya Opota
Alexander Opotowsky
Steffen Oppel
Ben Oppenheim
Sara Oppenheim
Charles Opperman
Brenda Oppert
Pablo Oppezzo
Anne Oppliger
Kwaku Oppong Asante
Alice Oprandi
Patricia Opresko
Tanja Opriessnig
Trine Opstad
Marjorie Opuni
Michael O'Quinn
Etti Or
Alexander Oraevsky
Berk Orakcioglu
Hakan Oral
Richard Oram
Christine Oramasionwu
J.B. Orange
Nicole Orange
Elkanah Orang'o
Peter Orazem
Tamas Orban
Jean-Jacques Orban de Xivry
Amaia Orbea
Paul Orchard
Francesc Ordines
Monica Ordway
Jacopo Oreglia
Ciara O'Reilly
Michael O'Reilly
Philip O'Reilly
Elena Orekhova
Laura Orellana
Elena Orellano
Pablo Orellano
Ronald Oremland
Mark Oremus
Aharon Oren
Eyal Oren
Isabel Orenes
Ivan Oresnik
Sotiris Orfanidis
Alberto Orfao
Caroline Orfila
Jason Organ
Jose Orgaz
Dennis Orgill
Virginie Orgogozo
Eniyou Oriero
Pedro Orihuela
Michele Orini
Koichi Orino
Zane Orinska
Nancy Oriol
Timothy Oriss
Jérome Orivel
Rita Orji
Pamela Orjuela-Sanchez
Augusto Orlandi
Rosaria Orlandi
Jéssica Orlandin
Giuseppe Orlando
Stefano Orlando
Marek Orlik
Thorsten Orlikowsky
David Orlov
Sergei Orlov
Marina Orlova
David Orlovich
Irene Orlow
Seth Orlow
John Orlowski
Rena Orman
Ian Orme
Mark Orme

Michelle Ormseth
Joanna Orne-Gliemann
Patricia Ornelas
Fernanda Ornellas
David Ornelles
Asher Ornoy
Katherine Ornstein
Uri Oron
Yoram Oron
Jorge Orós
Eyleen O'Rourke
Kathleen O'Rourke
Anthony Orr
Brent Orr
Miranda Orr
Robin Orr
Teri Orr
Isabel Orriss
Christina Orru
Andrea Orsi
Caitlin Orsini
Giovanna Orsini
Nicola Orsini
Sara Orsini
Anna Orsola
Nada Orsolic
Christophe Orssaud
Brian Ort
Donald Ort
Enrique Ortega
Francisco Ortega
Hugo Ortega
Jason Ortega
Sofia Ortega
Christian Ortega-Loubon
Joel Ortega-Ortiz
Guadalupe Ortega-Pierres
Julio Ortega-Usobiaga
Norberto Ortego-Centeno
Jason Ortegren
Thomas Ortel
Monica Ortenzi
Michael Orth
Guillermo Orti
Tania Ortiga-Carvalho
Ana Ortin
Alberto Ortiz
Alexis Ortiz
Amber Ortiz
Jorge Ortiz
Rodomiro Ortiz
Angelica Ortiz de Gortari
Eduardo Ortiz-Cruz
Christian Ortolf
David Orton
Ipek Oruc
Raoul Orvieto
Benjamin Ory
Ahmad Oryan
Miguel O'Ryan
Steven Orzack
Stephan Orzada
Francesco Orzi
Eneko Osaba
Naoki Osada
Tadeusz Osadnik
Luciana Osaki
Shahrokh Osaloo
Remus Osan
Takahiro Osawa
Takeshi Osawa
Sophia Osawe
Nosayaba Osazuwa-Peters
Danny Osborne
James Osborne
Melisa Osborne
Nicholas Osborne
Olivia Osborne
Jan Oscarsson
Padraig O'Seaghdha
Jacek Osek
Simona Osella
Cecilia Osera
Mohammad Oshaghi
Nir Osherov
Junko Oshima
Minako Oshima
Tadayuki Oshima
Yuji Oshima
Mitsuo Oshimura
Atsushi Oshio
Attila Osi
Faith Osier
Hans-Juergen Osigus
Evgeny Osin
Carole Oskeritzian
Andrea Oskis
Augustine Osman
Malina Osman
Mohammed Osman
Clive Osmond
Deanna L. Osmond

Johan Osorio
Flávia Osório
Gustavo Ospina-Tascón
Pauly Ossenblok
Alice Ossoli
Mike Osta
Michael Ostacher
Dirk Ostareck
Teresa Ostaszewska
Anna-Lena Ostberg
Gudrun Østby
Bertram Ostendorf
Florian Ostendorf
Harriet Oster
Henrik Oster
Michael Oster
Philipp Oster
Karen Østergaard
Marlies Ostermann
Nikolaus Osterrieder
Nancy Ostiguy
Örjan Östman
Bjørn Østman
Julia Ostner
Ljerka Ostojic
Julie Ostrander
Lisa Ostrin
Jamie Ostrov
Irina Ostrovnaya
Yan Ostrovski
Andrew N. Ostrovsky
Bruce Ostrow
Jerzy Ostrowski
Michael Ostrowsky
Renato Ostuni
Vikas Ostwal
Marcin Osuchowski
Yutaka Osuga
Mitsuru Osugi
Cait O'Sullivan
David O'Sullivan
Donal O'Sullivan
Patricia O'Sullivan
Mathias Osvath
Szabolcs Osváth
Andrew Oswald
Sara Oswalt
Hideaki Ota
Masao Ota
Motonori Ota
Tatsuya Ota
Yuko Ota
David Otaegui
Fernando Otálora-Luna
Gonzalo Otazu
Juan Oteiza
Dan Otelea
Selda Oterkus
Jaime Otero
Larissa Otero
Olga Otero
Pablo Otero
Jorge Otero-Millan
Carol Otey
Noordin Othman
Shatrah Othman
Megan Othus
Perrie O'Tierney-Ginn
Simon Otjes
Renee Otmar
Timo Otonkoski
Ronan F O'Toole
Atsushi Otsuka
Toshiaki Otsuka
Yasumasa Otsuka
Denise Otsuki
Naofumi Otsuru
Jurg Ott
Sascha Ott
Marte Otten
Andrew Ottens
Jonathan Otter
Leo Otterbein
Katrin Ottersbach
Inger Ottestad
Carola Otth Lagunas
Cornelia Ottiger
Laura Ottini
Karl Öttl
Caitlin Otto
Cynthia Otto
Kevin Otto
Lars-Gernot Otto
Mirko Otto
Ross Otto
Siegmar Otto
Timothy Otto
Claudio Ottoni
Eduardo Ottoni
Carl-Otto Ottosen
Akaninyene Otu
Hasan Otu

Gail Otulakowski
Daniel Otzen
Chun-Quan Ou
Fang-Shu Ou
Horng-Yih Ou
Qishui Ou
Shuo-Ming Ou
Yu-Yen Ou
Mehdi Ouaïssi
Eric Ouattara
H. Oubrahim
Lavi Oud
Raoul Oudejans
André Lin Ouédraogo
Scot Ouellette
Dennis Ougrin
Lobna Ouldamer
Merry Oursler
Tiago Outeiro
Alex Outkin
David Outomuro
Wayne Outten
Janneke Ouwerkerk
Ronald Ouwerkerk
Bo Ouyang
Fang Ouyang
Hanlin Ouyang
Hongsheng Ouyang
Jia Ouyang
Lin Ouyang
Min Ouyang
Minhui Ouyang
Pengfei Ouyang
Meric Ovacik
Dan Ovando
Attila Ovari
Howard Ovens
David Over
Nickola Overall
Annelies Overbeek
Paul Overbeek
Anna Overby
Casey Overby
Simon Overduin
Gudrun Overesch
Oyvind Overli
Robert Overman
Acacia Overoye
Turner Overton
Ken Overturf
Krista Overvliet
Per Ovesen
Lenin Oviedo
Mahmoudreza Ovissipour
Catherine Ovitt
Pavel Ovseiko
Mohammad Owais
Caroline Owen
Jennifer Owen
Julia Owen
Leah Owen
Mechelle Owen
Michael Owen
Nick Owen
Robert Owen
Hannah Owens
Leigh Owens
Max Owens
Rebecca Owens
Christopher J. Owers
Patrick Owili
Peter Owira
Olumuyiwa Owolabi
Jan W. Owsinski
Cynthia Owsley
Chuma Owuamalam
Aniwaa Owusu Obeng
Ebenezer Owusu-Addo
Annette Oxenius
Gregory Oxenkrug
David Oxman
Jun-ichi Oyama
Lila Oyama
Tonny Oyana
Jan Oyebode
Olusola Oyedeji
Yomi Oyelere
Ganiyu Oyetibo
Olufemi Oyewole
Adewale Oyeyemi
Soo Oym
Michiko Oyoshi
Murat Öz
Amit Oza
Shefali Oza
Kiyokazu Ozaki
Kouichi Ozaki
Sevgi Ozalevli
Shelly Ozark
Kazunari Ozasa
Nesrin Ozatac
Keiko Ozato

Koichiro Ozawa
Tetsutaro Ozawa
Umut Ozbek
Yusuf Ozbel
Ertugrul Ozbudak
Aydogan Ozcan
Orhan Ozcan
Berkay Ozcelik
G. Ozcengiz
Engin Ozcivici
Derya Ozdemir
P. Hande Özdinler
Mutlu Özdogan
Isao Oze
Bulent Ozel
Margareth Ozelo
Mustafa Ozen
Volkan Özenci
Egon Ozer
Mahmut Ozer
Nesrin Ozer
Michal Ozery-Flato
Ceyda Ozfidan-Konakci
Eren Ozguven
Gül Özhan
Lijo Cherian Ozhathil
Mukoso Ozieh
Hakan Ozkan
Selman Özkan
Yusuf Özkay
Elif Ozkirimli Olmez
David Ozog
Ryoji Ozono
Mecit Öztop
Selcen Öztürk
Anna Ozyczka
