# Supplementary material for: PLOS ONE 2016 Reviewer and Editorial Board Thank You
Source: PLoS One. 2017 Mar 20;12(3):e0174259. doi: 10.1371/journal.pone.0174259 (PMC5358840; doi:10.1371/journal.pone.0174259)
Supplement: S4 Reviewer List — (PDF) [file pone.0174259.s005.PDF]

PLOS | ONE

PLOS ONE would like to thank all those who reviewed on behalf of the journal in 2016:

Ramya P.
P. Paal
Yoav Paas
Noel Pabalan
Karel Pacak
Francesca Pacchierotti
Betty Pace
Edward Pace-Schott
Verity Pacey
Sabino Pacheco
F. Pacheco-Torgal
Jesús Pacheco-Torres
Elena Pachera
David Pacheu-Grau
Alicja Pacholewska
Joel Pachter
Marinella Paciello
Severina Pacifica
Lucia Pacifico
Maurizio Pacilli
Furio Pacini
Christopher Pack
Quinn Pack
Julie Packard
Craig Packer
Rowena Packer
Charles Packianathan
Percy Pacora
Martin Paczynski
Nesri Padayatchi
James Padbury
Michelle Paddack
Christopher Paddock
Cristina Padez
Shruti Padhee
Abinash Padhi
Pedro Padilha
Dianna Padilla
Monica Padilla
Ranjith Padinhateeri
Vered Padler-Karavani
Vasantha Padmanabhan
Roshan Padmashali
Raul Padron
David Padua
Francesco Paduano
Matthew Padula
Eung-Kwon Pae
Brian Paegel
Ki Young Paek
Jin Chul Paeng
Bosco Paes
Gabriel Paës
Jan Paeshuyse
Slobodan Paessler
Guenter Paeth
Evangelos Pafilis
Ascensión Pagán
Fernanda Paganelli
Giorgio Pagani
Luca Pagani
Marco Pagani
Aldo Pagano
Ester Pagano
Eva Pagano
Livio Pagano
Patrick Pagano
Amy Page
Anne-Laure Page
Clive Page
Kathleen Page
Kimberly Page
Lawrence Page
Timothy Page
Benjamin Pageaux
Mark Pagel
Natalie Pageler
Janet Page-Reeves
Guilhem Pages
Jean-Christophe Pages
Liesl Page-Shipp
Christophe Paget
Stefano Pagiolo
Giuseppe Paglia
David Pagliarini
Pasquale Pagliaro
Vincenzo Pagliarulo
Paulo Pagliosa
Andrea Pagnani
Giorgio Pagni

Cristiano Pagnini
Francesco Pagnini
Giuseppe Pagnoni
Daniela Pagnozzi
Gabriela Pagnussat
Efstathios Pagourelias
Bhavna Pahel
Madeleine Pahl
Niema Pahlevan
Savita Pahwa
Clive Pai
Emil Pai
Ming-Chyi Pai
Alessandro Paiardini
Judith Paice
Samantha Paige
Hae Jung Paik
Soon-Young Paik
Taejong Paik
Patrick Paillet
Romain Paillot
Bertrand Pain
Matthew Pain
Robert Paine
Tim Paine
Tracie Paine
Dean Paini
Francesca Paino
I. Painter
L.M. Painter
Matthias Paireder
Monique Pairis-Garcia
Bruno Paiva
Sandra Paiva
Vitor Paiva
María Pajares
Jukka Pajarinen
Kostandin Pajcini
Gholam Pajenda
Krisztián Pajer
Dasja Pajkrt
Eloísa Pajuelo
Virpi Pajunen
Hui-Nam Pak
On Shun Pak
Veli-Matti Pakanen
Trevino Pakasi
Abhijit Pakhare
Mariya Pakharukova
Serguei Pakhomov
Mina Pakkhesal
Amir Pakpour
Diana Paksarian
Chiranjib Pal
Krishnendu Pal
Mahadeb Pal
Probir Pal
Ranadip Pal
Subhamoy Pal
Sukumar Pal
Tuya Pal
Tibor Pál
Maria Pala
B. Palabiyik
Manuel Palacín
Montse Palacio
Adrian Palacios
Diego Palacios
Jose Palacios
Sara Palacios
Miriam Palacios-Callender
Jose Palacios-Jaraquemada
Fabiana Paladini
Giuseppe Paladini
Paolo Paladini
Claudia Azucena Palafox-Sánchez
Patricia Palagi
Laura Palagini
Mathew Palakal
Joseph Palamar
Karthikeyan Palanisamy
Manikandan Palanisamy
Nallasivam Palanisamy
Thirumoorthy Palanisamy
Marimuthu Palaniswami
Kanagaraj Palaniyandi
Senthilkumar Palaniyandi
Suresh Palaniyandi
Diego Palao
Victoria Palau
Santiago Palazón
Luis Palazzesi
Clémence Palazzo
Anandan Paldurai
Andres Palencia
Brian Palenik
Ferran Palero
Jordi Pales
Alvisa Palese
Peter Palese
Rafael Palhares
Fleur Paling

Miltiadis Paliouras
James Palis
Isabella Pali-Schöll
Gergely Palla
Arianna Palladini
Miranda Pallan
Mark Pallansch
Pierlorenzo Pallante
Virginia Pallante
Roman Pallares
Alberto Pallavicini
Maria Pallayova
Mark Pallen
Channing Paller
Lars-Peder Pallesen
Ståle Pallesen
Nicolas Pallet
Suresh Pallikkuth
Eletherios Pallis
Jannik Pallisgaard
Thomas Pallone
Valentina Pallottini
Carlo Pallotto
Johannes Pallua
Günther Palm
Stig Palm
Anton Palma
David Palma
Mário Palma
R. Eduardo Palma
Paul Palmberg
Andre Palmer
Cameron Palmer
Chris Palmer
Clovis Palmer
Colin Palmer
Jonathan Palmer
Mark Palmer
Michael Palmer
Mitchell Palmer
Richard Palmer
Robert Palmer
Tracy Palmer
Antonio Palmeri
Tom Palmeri
Vincenzo Palmeri
Gaby Palmer-Lourenco
Cristina Palmero
Arianna Palmieri
Michelle Palmieri
Stefano Palminteri
Alida Palmisano
Richard Palmiter
Carolyn Palmquist
Lars Palmqvist
Glenn Palomaki
Stefano Palomba
Leonardo Palombi
Iván Palomo
Jennifer Palomo
Michael Palopoli
William Paloski
Erik Palsson
Arjan Palstra
Yniv Palti
Yoram Palti
Ora Paltiel
Giorgio Palu
Wojtek Palubicki
Paul Palumbo
R. Palumbo
S. Palumbo
Milan Palus
Marjo Palviainen
Frederic Pamoukdjian
João Pamphile
Jeremy Pamplin
Reinald Pamplona
Elzbieta Pamula
Aaron Pan
Bin Pan
Bing-Xing Pan
Chong-Xian Pan
Chuxiong Pan
Edward Pan
Feng Pan
Fenghua Pan
Guoqing Pan
Huichin Pan
Huipeng Pan
Hung-Chuan Pan
Jay Pan
Jeff Pan
Jen-Jung Pan
Jianbo Pan
Jing Pan
Jinger Pan
Jonathan Pan
Linqiang Pan
Liping Pan
Lizhi Pan
Min-Hsiung Pan

Min-Hui Pan
Qing Pan
Qiuhui Pan
Rong Pan
Ruqian Pan
Sheng Pan
Sheng-Wei Pan
Songqin Pan
Stephen Pan
Tai-Long Pan
Tsorng-Whay Pan
Wei Pan
Wensheng Pan
Xinghua Pan
Xiongfei Pan
Xu Pan
Xueliang Pan
Ya-Ping Pan
Yi-Ju Pan
Yuan Pan
Yuchun Pan
Yue Pan
Z.Y. Pan
Zhen-Qiang Pan
Daniel Panaccione
Ales Panacek
Katherine Panageas
Costas Panagiotakis
Demostenes Panagiotakos
Demosthenes Panagiotakos
Korovessis Panagiotis
Grigorios Panagiotou
Costas Panagopoulos
Efstathios Panagou
Farhad Panahi
Stefan Panaiotov
David Pañaranda
Maria Serena Panasiti
Ganna Panasyuk
Aude Panatier
Donatella Panatto
Alessandra Panattoni
Panagiotis Panayiotidis
Bhavik Panchal
Karthik Panchanathan
Manikandan Panchatcharam
Anna R Panchenko
Vijay Pancholi
Marina Pancic
Gianfranco Pancino
Julia Panczuk
Amaresh Panda
Brahma Panda
Chinmay Panda
D. Panda
P.K. Panda
Prasanna Panda
Rajmohan Panda
Arvind Panday
Paritosh Pande
Tripti Pande
Abhishek Pandey
Ajay Pandey
Akhilesh Pandey
Ashutosh Pandey
Gaurav Pandey
Ghanshyam Pandey
Janmejay Pandey
Kavita Pandey
Manish Pandey
Manoj Pandey
Pramod Pandey
Ramendra Pandey
Ravindra Pandey
Saurabh Pandey
Shilpa Pandey
Sona Pandey
Virendra Pandey
Vishal Pandey
Akash Pandhare
Jul Pandhare
Nikolaos Pandis
Hemant Pandit
Maharaj Pandit
Ram Pandit
Shashi Pandit
Ramesh Pandita
Stephen Pandol
Assunta Pandolfi
Silvia Pandolfi
Chiara Pandolfini
Victoria Pando-Robles
Lavanya Pandranki
Ivona Pandrea
Marcus Pandy
Amit Pandya
Ronney Panerai
Isabella Panfoli
Bo Pang
Daniel Pang
Hong Pang
Iok-Hou Pang

Junxiong Pang
Ka Ming Pang
Li-Li Pang
Myung-Geol Pang
Yanli Pang
Yi Pang
Ying Pang
Galen Panger
Qiang Pan-Hammarström
Ajaya Pani
Giuseppe Pani
Ramón Paniagua
Narel Paniagua-Zambrana
Rita Paniccia
Mauro Panigada
Gatikrushna Panigrahi
Rajesh Panigrahi
Sunil Panigrahi
Valéria Panissa
Frank Panitz
Peter Panizzi
Mojtaba Panjehpour
Tibor Pankotai
Dejana Pankovic
Nathan Pankratz
Jules Panksepp
Anna Pannaccione
Sigamani Panneer
Shan Panneer Selvam
Jürgen Pannek
Kerstin Pannek
David Pannell
Lewis K Pannell
Simona Panni
Thomas Pannicke
Angela Pannier
Evan Pannkuk
Jasdeep Pannu
Georgios Panos
Ralph Panos
Renata Panosso
Elena Panou
Emerson Pansarin
Maurizio Pansini
Chaitanya Pant
Deepak Pant
Vinod Pant
Sergio Pantano
Katerina Pantavou
Eric Pante
Marija Pantelic
Matthew Pantell
Jenna Panter
Igor Pantic
Omar Pantoja
Costas Pantos
Annalisa Pantosti
Gabriella Panuccio
Bharat Panwar
Jitendra Panwar
Sneh Panwar
Irina Panyushkina
Francesco Panza
Manuela Panzacchi
Ute Panzenboeck
Matthew Panzer
Ursula Panzner
Luca Panzone
Derek Pao
Wansa Paoin
Gabriele Paolacci
Pierre Paoletti
Antonio Paoli
Chiara Paoli
George Paoli
Stefania Paolillo
Guido Paolini
Stefania Paolini
Nazareno Paolocci
Ildiko Pap
Federica Papaccio
Efstathios Papachristou
Sophia Papadakis
Chrysa Papadaniil
Evangelina Papadavid
Chrysoula Papadeli-Kourtidou
Evangelia Papadimitriou
Konstantinos Papadimitriou
Athanasios Papadopoulos
N. Papadopoulos
M. Papafaklis
Dimitrios Papageorgiou
Louis Papageorgiou
Andriana Papaioannou
Evangelos Papalexakis
Konstantinos Papamichail
Nikolaos Papanas
George Papandonatos
Alexie Papanicolaou
Nikolaos Papanikolaou
Nikolas Papanikolaou
Nikos Papanikolaou

Seraphim Papanikolaou
Katerina Papanikolopoulou
Joseph Papaparaskevas
Andreas Papapetropoulos
Sara Paparini
Eric Papas
Yannis Papastamatiou
George Papatheodoridis
Stefania I. Papatheodorou
Ian Papautsky
Vasileios Papavasileiou
Jason Papazian
Lars Pape
Jesse Papenburg
Frank Papenmeier
Fabio Papes
M. Papes
Mikhail Papisov
Roger Papke
Zoi Eirini Papliaka
Garegin Papoian
Balázs Papp
Zoltan Papp
Scott Pappada
Anna Maria Pappalardo
Salvatore Pappalardo
Christos Pappas
Ioannis Pappas
Maria L. Pappas
Matthew Pappas
Daciana Papura
Michel Paques
Agnes Paquet
Nicole Paquet
Francois Paquet-Durand
Cherie Paquette
Dominic Paquin-Proulx
Michael Para
Luis Parada
Adriana Parada Silveira
Suzanne Paradis
Elodie Parain
Rajendra Prasad Parajuli
Galina Paramei
David Paramelle
Jesús Paramio
Augusto Paranhos
Gláucia Paranhos-Baccalà
John Parant
Parveen Parasar
Paula Parás-Bravo
Antonios Paraschakis
Abhinav Parashar
Despoina Parasyri
Luca Parca
Mark Parcells
Piero Parchi
Panos Pardalos
Melissa Pardi
Matteo Pardini
Alejandro Pardo
Alfredo José Pardo Cabello
Zachary Pardos
Machelle Pardue
Mary-Lou Pardue
Paul Pare
Peter Pare
Daniel Paredes
Raul Paredes
Roger Paredes
Vanessa Paredes
Rene Pareja
Helios Pareja-Galeano
Bharat Parekh
Swati Parekh
Carole Parent
Eric Parent
Leon-Etienne Parent
Christian Paret
Helena Parfenova
Groesbeck Parham
Kourosh Parham
Ishwar Parhar
Keshab Parhi
Ashish Parida
Swarup Parida
Mihir Parikh
Neehar Parikh
Sunil Parikh
Urvi Parikh
Jean Parinaud
Paolo Parini
Debora Paris
Joel Paris
Mathilde Paris
Sebastian Paris
Anne R. Pariser
Dave Parish
Gustavo Parisi
Loukia Parisiadou
Marc-André Parisien
Nicolas Parisot

Bong-Wook Park
Byung Bae Park
Chan Kee Park
Changwon Park
Chankyu Park
Choul Yong Park
Daewon Park
Do Hyun Park
Dongjin Park
Duck Hwan Park
Elyse Park
Eun Hye Park
Eun-Cheol Park
Hae-Jeong Park
Haeme Park
Hana Park
Hee Chul Park
Hee-Soo Park
Heiyoung Park
Hun-Kuk Park
Hyojin Park
Hyun Park
Hyunjin Park
Hyun-Seok Park
Hyunsook Park
Il-Kwon Park
In-Hyun Park
Jae-Hyeong Park
Jae-Il Park
Jeehye Park
Jerry Park
Ji Park
Ji Sook Park
Jin Park
Jin Kyun Park
Jin-Woo Park
Jong Hoon Park
Jongseok Park
Joo-Cheol Park
Joong Yull Park
Joonghoon Park
Joong-Ki Park
Joon-Young Park
Joshua Park
Jun Yong Park
Jung-Eun Park
Junyoung Park
Juyong Park
Ki Ho Park
Kwon-Moo Park
Kyong Soo Park
Kyoung Un Park
Kyoung-Joo Jenny Park
Kyung (Kevin) Park
KyungBae Park
Kyung-Tae Park
Lesley Park
Margriet Park
Mee Sook Park
Mi-Jung Park
Moo Kyun Park
Myung Park
Myung Hee Park
Paul Park
S.K. Park
Sang Park
Sang Gyu Park
Sanghyun Park
Sang-Kyu Park
Seong Yong Park
SeungWon Park
Si Hong Park
Solip Park
Song-Young Park
Soo Park
Soo Young Park
Sung Park
Sung Bae Park
Sungha Park
Sung-Hong Park
Sungsu Park
Sungwoo Park
Susanna Park
Thomas Park
Woong-June Park
Yehyun Park
Yeon-Joon Park
Yong-Cheol Park
YongKeun Park
Yong-Lak Park
Yong-Moon Park
Yoonjung Park
Young Joo Park
Youngja Park
Youngjin Park
Young-Jun Park
Albert Parker
Britt Parker
Charles Parker
Claire Parker
Craig Parker
Dane Parker

Daniel Parker
Darren Parker
Denham Parker
Glendon Parker
Gordon Parker
Helen Parker
Ian Parker
J. Alex Parker
Jason Parker
Joel Parker
Joseph Parker
Lisa Parker
M. Rockwell Parker
Marianna Parker
Marti Parker
Martyn Parker
Matthew Parker
Maura Parker
Megan Parker
Michael Parker
Wayne Parker
William Parker
Jan Parker-Thornburg
Laura Parkes
Julian Parkhill
Michael Parkins
Brian Parkinson
Jennifer Parkinson
John Parkinson
Joy Parkinson
Kathryn Parkinson
John Parks
Michael Parks
Nathan Parks
Robin Parks
Sean Parks
Susan Parks
Onur Parlak
Aldo Parlato
Natalie Parletta
Eric Parmentier
Priya Parmer
Christopher Parmeter
Maria Parmley
Veena Parnaik
Grant Parnell
J.A. Parnell
Scott Parnell
Lucilla Parnetti
Mohamad Parnianpour
Martin Parniske
Sittiporn Parnmen
Guido Parodi
Maurizio Parola
Philippe Parola
Daniela Parolaro
Marco Parolini
Maria Paola Paronetto
Matteo Parotto
Ann Parr
Diana C Parra
Mario Parra
Socorro Parra
Mauro Parra-Cordero
Cristina Parrado-Fernandez
Emilio Parrado-Hernández
C. Alejandro Parraga
Jorge Parra-Ruiz
Carlos Parras
Miguel Parra-Saavedra
Steven Parratt
Chiara Parravicini
Ricardo Parreira
Lucas Parreiras-e-Silva
Paola Parrella
Viviana Parreño
Abby Parrill
Inmaculada Parrilla
Christina Parrinello
Toshima Parris
Colin Parrish
Christopher Parry
Selina Parry
Sharon Parry
Adrian Parry-Jones
Amber Parry-Strong
Soroush Parsa
Laura Parsley
Simon Parson
Walther Parson
Chris Parsons
Daniel Parsons
Glenn Parsons
Harriet Parsons
John Geoffrey Morgan Parsons
Michael Parsons
Kaushik Parthasarathi
Kotzekidou Parthena
Santiago Partida-Sanchez
Debra Partington
Timo Partonen
Charlyn Partridge

Lynda Partridge
Sally Partridge
Valerie Partridge
Amit Parulekar
Avi Parush
Michelle Parvatiyar
Nikhat Parveen
Thomas Parzefall
Michal Parzuchowski
Sirichai Pasadhika
Anastasia Paschalidou
Eleftherios Paschalis
V.Z. Paschenko
Maurizio Paschero
David Pasco
Peter Pascoe
Philippe Pascua
J. Pascual
Marta Pascual
Mercedes Pascual
David Pascual-Ezama
Alberto Pascual-Garcia
David Pascucci
Manijeh Pasdar
Matthew Pase
Daniela Pasero
Marco Pasi
Giulio Pasinetti
Antonello Pasini
Erica Pasini
Jantsje Pasma
Edoardo Pasolli
Giuseppe Pasqualetti
Massimo Pasqualetti
Claudio Pasquali
Giancarlo Pasquali
Lorenzo Pasquali
Matias Pasquali
Colasuonno Pasqualina
Antonella Pasqualone
Emanuele Pasqualotto
Alain Pasquet
Jean-Max Pasquet
Eddy Pasquier
Jérémy Pasquier
Maria Passafaro
Sabina Passamonti
Carlos Passarelli
Giuseppe Passarino
Don Passey
Daniela Passilongo
Nadine Paßlack
Martin Passmore
Giselle Passos
Pedro Passos
Franck Pasta
Linda Pasta
Irena Pastar
Robert Pastel
Lucio Pastena
Bruce Paster
Jeroen Pasterkamp
Alexander Pasternak
Amy Pasternak
Anna Pasternak
Björn Pasternak
Ofer Pasternak
Zohar Pasternak
Manoj Pastey
Rodrigo Pasti
Fernando Pastor
Antonio Pastore
Concetta Pastorelli
Luca Pastorelli
Luigi Pastormerlo
Maria Pastor-Valero
Chiara Pastrello
Matthew Paszek
Vikas Patade
Todd Pataky
Solenn Patalano
Swati Patankar
Manuel Patarroyo
Harland Patch
Joy Pate
Sandeep Patei
Amrish Patel
Ankita Patel
Ashok Patel
Asish Patel
Basant Patel
Bhavesh Patel
Dhavalkumar D. Patel
Dipika Patel
Girijesh Patel
Hiten Patel
J.K. Patel
Jignesh Patel
Ketan Patel
Mahesh Patel
Mahomed Patel
Mitesh Patel

Mrinali Patel
Niketa Patel
Nimesh Patel
Raj Kishore Patel
Rakesh Patel
Rashmi Patel
Ravi Patel
Rekha Patel
Riyaz Patel
Samir Patel
Sandip Patel
Sanjay Patel
Sheila Patel
Sravan Kumar Patel
Vyomesh Patel
Yashomati Patel
Edoardo Patelli
Andrew Paterson
Brett Paterson
Pauline Paterson
Arvind Pathak
Ashutosh Pathak
Chandramani Pathak
Devendra Pathak
Harsh Pathak
Al-Sakib Khan Pathan
Ishani Pathmanathan
Lawrence Patihis
B. Patil
Kiran Patil
Naeem Patil
Satish Patil
Sumeet Patil
Vijay Patil
Jairo Patino
Reynaldo Patino
Mrinal Patnaik
Carrie Patnode
Sanjay Patole
James Paton
Joanne Paton
Nicholas Paton
Abani Patra
Amlan Patra
Partha Patra
Amee Patrawalla
Om Patri
Riccardo Patriarca
Patricia Patrician
Christopher Patrick
Jego Patrick
Kennerly Patrick
Kristin Patrick
Steve Patrick
Paola Patrignani
Glenn Patriquin
Michelle Patriquin
Cesare Patrone
Eleni Patrozou
Antonia Patruno
Philippos Patsalis
Daniel Patschan
Oliver Patschan
Theofania-Sotiria Patsiou
Lucy Patston
Vallandramam Pattabhiraman
Kovit Pattanapanyasat
Sittporn Pattaradilokrat
Cristian Pattaro
Sivakumar Pattathil
Gabriela Patten
Michael Patten
Monica Patten
Adam Patterson
Andrew Patterson
Brandon J. Patterson
David Patterson
James Patterson
Ned Patterson
Sue Patterson
William Patterson
Laszlo Patthy
Giuseppe Patti
Mary Elizabeth Patti
Chris Pattillo
Natalie Pattison
Bramhadev Pattnaik
Stephanie Patton
Thomas Patton
Frank Pattyn
Kelly Patyk
Edward Patz
Sebastian Patzelt
Maximilian Patzig
Yannick Pauchet
Khum Paudayal
Babu Ram Paudel
Priyamvada Paudyal
Ramesh Paudyal
Antonio Paul
Arindam Paul
Birthe Paul

David Paul
Dennis Paul
Jacob Paul
Mandira Paul
Michael Paul
Paramita Paul
Sandeep Paul
Soumen Paul
Subhankar Paul
Débora Paula
Fabiola Paula-Lopes
Dagenais Paule
Juraj Paule
Cristina Paules
Loïc Paulevé
Heiko Paulheim
Adèle Paul-Hus
Jonathan Pauli
Natasha Pauli
Roxane Paulin
John Pauling
Ivan Paulino-Lima
Gianni Paulis
Timothy Paulitz
Angelique Paulk
Nicole Paulk
Robert Paull
Markus Paulmichl
Ramasamy Paulmurugan
Barbara Pauloski
Peter Pauls
Jonas Paulsen
Alison Paulson
Joseph Paulson
Natalia Paulucci
Martin Paulus
Bernadette Pauly
Nicolas Pauly
Fabienne Paumet
Francisco Paumgartten
Christian Paumi
Gheorghe Paun
Tatjana Paunesku
Katarina Paunovic
Ralf Paus
Juli Pausas
Hubert Pausch
Ricardo Pautassi
Emmanuel Pauthe
Alex Pauvolid-Corrêa
Fredrick Pavalko
Stefano Pavan
Ana Pavasovic
Gregory Pavela
Giacomo Pavesi
Giulio Pavesi
Louisa Pavey
Roman Pavic
Zoran Pavicevic
Nicole Pavio
Juan Pablo Pavissich
Graham Pavitt
Tea Pavkov-Keller
David Pavlacky
George Pavlakis
Martha Pavlakis
Mihaela Pavlicev
Peter Pavlidakey
Nena Pavlidi
Ioannis Pavlidis
Nicholas Pavlidis
Paul Pavlidis
Pavlos Pavlidis
Mojca Pavlin
Patricia Pavlinac
Luigi Michele Pavone
Kantikumar Pawar
Rajendra Pawar
Agnieszka Pawelek
John Pawelek
Janusz Paweska
M. Pawlak
Michal Pawlak
Ludmila Pawlikowska
Charlotte Pawlowski
Jan Pawlowski
Michal Pawlowski
Stephen Pawson
Alexandra Paxton
Eben Paxton
Jessica Paxton
Robert Paxton
Ivan Paya
Mehrdad Payandeh
Doris Payer
Dominique Payet-Bornet
Beth Payne
Brendan Payne
Christopher Payne
Daniel Payne
Deborah Payne
Elspeth Payne

Jessica Payne
Keith Payne
Kyle Payne
Jessica Paynter
Sarah Paynter
Antoine Payot
A. Payton
Cristina Paz
Rony Paz
Ziv Paz
Antonio Paz González
Adam Pazda
Katarzyna Pazdzior-Czapula
Gilberto Paz-Filho
Valerio Pazienza
Lev Pazin
Gregory Pazour
Federico G. Pazzona
Federico Pea
Karl Peace
Neal Peachey
Mary Peacock
Taylor Peak
Rod Peakall
Jonathan Peake
Mark Peakman
Warren Pear
Anna Pearce
Douglas Pearce
Elizabeth Pearce
Grant Pearce
Hannah Pearce
J. Pearce
Stephen Pearce
Michael Pearen
Monica Pearl
Eric Pearlman
John Pearn
Catherine Pears
Amy Pearson
Christopher Pearson
David Pearson
Erin Pearson
Frances Pearson
Gareth Pearson
James Pearson
Jennifer Pearson
Melanie Pearson
Mike Pearson
Ryan Pearson
Talima Pearson
William Pearson
Yanthe Pearson
Claire Peart
Jason Peart
Courtney Peasant
James Pease
Thomas Peat
Alexis Peaucelle
Marc Peaucelle
Jean Peccoud
Clint Pecenka
Jean-Claude Pech
Roger Pech
Jennifer Pechal
Tibor Pechan
Jan Pechenik
Kate Pechenkina
Marta Pecina
Kyung Peck
Michael Peck
Robert Peck
Octavia Peck Palmer
Michelle Peckham
Paulette Peckol
Lorenzo Pecoraro
Jacques Pecreaux
Vidyullatha Peddireddy
Margie Peden
Alma Pedersen
Anette Fischer Pedersen
Dorthe Pedersen
Eric Pedersen
Martin Pedersen
Mette Pedersen
Mikael Pedersen
Steen Pedersen
Bart Pederson
Federica Pedica
Charles Pedlar
Joao Pedra
Alisa Pedrana
José Pedraza-Chaverri
Corrado Pedrazzani
Thierry Pedrazzini
J. Pedrera
Romina Pedreschi
Alessandro Pedretti
Nicolas Pedrini
Gianni Pedrizzetti
Nuria Pedrol
Thierry Pedron

Jorge Pedrosa
Andrea Pedrosa-Harand
Abraham Pedroza-Torres
Christie Peebles
Donald Peebles
Ray Peebles
R. Peebles Jr.
Nancye Peel
Peter Peeling
Rosanna Peeling
Mark Peeples
Nasheeta Peer
Ben Peeters
Eveline Peeters
Geeske Peeters
Sarah Peeters
Mandy Peffers
Vicente Peg
Elise Pegg
Corinna Pehrs
De-Sheng Pei
Honglei Pei
Ming Pei
Shuwen Pei
Xin-Wu Pei
Willie Peijnenburg
V. Peinado
Anna Peired
Pubudu Peiris
T. Harshani Peiris
Jose Luis Peiró
Wiebke Peitsch
Henry Peixoto
Raquel Peixoto
Tanja Pejovic
A. Pejovic-Milic
Stanislav Pekar
Kerem Pekkan
Vanja Pekovic-Vaughan
Joel Pekow
Giovanna Pela
Carmen Pelaez
Girolamo Pelaia
Camille Pelat
R.A. Pelc
Pawel Pelczar
Istvan Pelczer
Shahaf Peleg
Yoav Peleg
Mireia Pelegrin
Simone Peletto
Gert Jan Pelgrim
Pier Guiseppe Pelicci
Aylin Pelin Cil
Antoine Pelissolo
D. Pelisson
Lisa Pell
Maria Teresa Pellecchia
Anthony Pellegrini
Barbara Pellegrini
Christine Pellegrini
Lawrence Pellegrini
Manuel J. Pellegrini
Maria Antonietta Pellegrino
Sara Pellegrino
Roseli Pellens
Chelsea Pelletier
Guy Pelletier
Jerry Pelletier
Julien Pelletier
Tara Anne Pelletier
Vivien Pellis
Cláudia Pellizzon
Livio Pellizzoni
Jennifer Pellowski
Joanna Peloquin Melia
Kevin Pelphrey
Rick Peltier
Gretel Pelto
Stephen Pelton
Juha Peltonen
Gianfranco Peluso
Ilaria Peluso
John Peluso
Kirsten Pelz-Stelinski
Keith Pembleton
Lucy Pembrey
Alex Pemov
Javier Pena
Fernando Peña
José Peña
Jose Penades
Pablo Peña-García
Francisco Peñagaricano
Pablo Penaloza
Miguel Penalva
Marta Penas-Prado
Brian Pence
Valerie Pence
Silvana Penco
Mario Pende
Ann Marie Pendergast

Julie Pendergast
Sarah Pendergrass
María Victoria Pendón-Ruiz de Mier
Leslie R. Pendrill
Ram Pendyala
Eva Penelo
Vlada Peneva
Ai Peng
Aimin Peng
Bi-Wen Peng
Bo Peng
Chao Peng
Chen Peng
Cheng-Yuan Peng
Daxin Peng
Deliang Peng
Fang Peng
Fei Peng
Giia-Sheun Peng
Guang Peng
Guanghan Peng
Guangyong Peng
Han Peng
Hao Peng
Hsien-Yu Peng
Huiru Peng
Jiajie Peng
Jian Peng
Jiaxi Peng
Junhua Peng
Junmin Peng
Kuan-Po Peng
Liang Peng
Lu-Ying Peng
Min-Sheng Peng
Qinmu Peng
Shushi Peng
Tao-Chun Peng
Ting Peng
Wei Peng
Wen-Huang Peng
Xinxia Peng
Xuan-Xian Peng
Y. Peng
Yibing Peng
Yichuan Peng
Yifan Peng
Yi-Jen Peng
Ying Peng
Yong Peng
Yu Peng
Yulan Peng
Zhaohua Peng
Zhaoxia Peng
Zhikang Peng
Zongju Peng
Kenneth Pengel
Vittorio Pengo
Virginia Penhune
Jonathan Penm
Alex Penn
Claudia Penna
Fabio Penna
Mario Penna
Angela Pennacchio
Iris-Katherina Penner
Johannes Penner
Giuseppa Pennetta
Trevor Penning
Charles Penninger
Steven Pennings
Charlotte Pennington
Katie Pennington
Cristian Pennisi
Marzio Pennisi
Kristina Penniston
Erin Penno
Joanne Pennock
Robert Pennock
Keith Pennypacker
Sri Ram Pentakota
Deepak Pental
Mikko Pentinsaari
M. Pentrák
Kirsi Penttinen
Srinivasa Penumutchu
Salvatore Pepe
Jean-Louis Pepin
Yanina Pepino
Marc Pépino
Jenny Peplies
Beata Peplonska
Carolyn Pepper
Gillian Pepper
Michael Pepper
Irene Pepperberg
Claire Peppiatt-Wildman
Kathryn Pepple
Jason Peragallo
Pandelis Perakakis
Celia Perales
Jose Cesar Perales

Ariane Peralta
Carmen Peralta
Jose Peralta
Juan Manuel Peralta-Sanchez
Daniela Perani
William Peranteau
Ravindra Peravali
Mark Perazella
Gloria Perazzoli
Matjaz Perc
Christine Percheski
Gaetano Perchiazzi
Christopher Percival
Riccardo Percudani
Alan Percy
Richard Percy
Dionysios Perdikis
Helène Pere
M. Perea
Silvia Perea
Felipe Perecin
Agnieszka Perec-Matysiak
Javier Pereda
Carlos Peredo
Carla Perego
Alexandre Pereira
Andy Pereira
Carina Pereira
David Pereira
Eliseu Pereira
Francisco Gilberto Fernandes Pereira
Fred Pereira
Joana Pereira
João Pereira
Jose Pereira
Lara Pereira
Lenore Pereira
Luciano J. Pereira
Luiz Pereira
Luiz Filipe Pereira
Pedro Pereira
Rinaldo Pereira
Ruth Pereira
Sandrine Pereira
Tatiani Pereira-Cenci
Marco Pereira-Sampaio
Pablo Perel
Josep Perello
Mario Perello
Asya Pereltsvaig
Dominic Pérennou
Jason Perepelkin
Anoma Perera
Marlon Perera
Ranjan Perera
Marco Peres
Raphael Peres
Nuno Peres Almeida
Sabine Pereyre
Alexander Perez
Christian Perez
Dennis Perez
Jesus Perez
Juan Perez
Juliana Perez
Manolo Perez
Marco Perez
Marta Perez
Paulino Perez
R. Perez
Ruben Perez
S. Perez
Aritz Pérez
Francisco Pérez
Teodosio Perez Amaral
Ricardo Perez Cuevas
Ignacio Perez de Castro
Ignacio Pérez de Castro
Verónica Perez de la Cruz
Luis Pérez De Sevilla Mueller
José Napoleón Pérez Farinós
Miguel Pérez Fontán
Juan Pérez García
Alex Perez Girbes
Gerardo Pérez Ponce de León
Francisco Jose Perez Reche
María José Pérez-Alvarez
Miguel Pérez-Amador
Miguel Angel Perez-Angon
R. Pérez-Badia
Daisy Perez-Brito
David Perez-Callejo
Juan Pérez-Claros
Daniel Perez-Cremades
Sofia Perez-del-Pulgar
Koraly Pérez-Edgar
Miguel Perez-Enciso
N. Pérez-Hernández
Jesús Pérez-Losada
C.C. Perez-Marin
Leonor Perez-Martinez
Alejandro Perez-Matus

Valentín Pérez-Mellado
Manuel Perez-Molina
Dafne Pérez-Montarelo
Javier Perez-Moreno
Mirna Perez-Moreno
Antoni Perez-Navarro
Eduardo Pérez-Palma
José Manuel Pérez-Pérez
María-Jesús Pérez-Pérez
José Pérez-Pomares
Tomas Perez-Porcuna
Yasset Perez-Riverol
Ana Perez-Ruiz
Angel Pérez-Ruzafa
Jaume Pérez-Sánchez
Ricardo Pérez-Tomás
Victoriano Pérez-Vázquez
Miguel Perez-Viloria
Francisco Perez-Vizcaino
Francisco Perfectti
Charles Perfetti
Renzo Perfetti
Muthu Periasamy
Luca Perico
Eva Pericolini
Jamie Perin
Laura Perin
Fernando Perini
Albert Peris
Palsamy Periyasamy
Oliver Perkin
Douglas Perkins
Jessica Perkins
Matthew Perkins
Rebecca Perkins
Stephen Perkins
Theodore Perkins
Timothy Perkins
Vlado Perkovic
Gabor Perlaki
Susan Perlen
Fotis Perlikos
Rita Perlingeiro
Dan Perlman
Susan Perlman
Eran Perlson
Cyril Pernet
Guey Chuen Perng
Jeffrey Pernica
Mathieu Pernice
Gilles Pernod
Tony Pernthaner
Ana Carolina Pero
Alexandre Perochon
Franck Peron
Guillaume Péron
Sammy Perone
Jean-Pierre Peros
Silvia Perotto
Olga Perovic
Nicola Perra
Anastassis Perrakis
Jean-Pierre Perreault
Leigh Perreault
Alain Perret
Carlo Perricone
Daniel Perrien
Charles Perrier
Nancy Perrier
Byron Perrin
Jeanne Perrin
Laurent Perrin
Nicolas Perrin
Susan Perrine
Laurent Perrinet
Thomas Perring
Agnes Perrin-Guyomard
Sebastio Perrini
Brian Perrino
Hervé Perron
Serafina Perrone
Nora Perrone-Bizzozero
Fabrizio Perroni
Linda Perrotti
Gianluca Perrucci
Adam Perry
Ben Perry
Christopher Perry
George Perry
Henry Perry
Howard Perry
Linda Perry
Nicholas Perry
Robert Perry
Thomas Perry
Geeta Persad
Luca Persani
Luca Persano
Pedro Persechini
Pedro M. Persechini
Gianluca Perseghin
Juliana Perseguini

Anton Persikov
Aaron Persinger
Michael Persinger
Susan Persky
Derek Peršoh
Alexander Persoskie
Cecilia Persson
Egon Persson
Karina Persson
Katarina Persson
Lo Persson
Tomas Persson
Loris Perticarini
Francesco Perticone
Inna Pertsovskaya
Piero Perucca
Giulio Perugi
Matthew Perugini
Deepak Perumal
Ekambaram Perumal
Omathanu Perumal
Ramssamy Perumal
Ramar Perumal Samy
Licia Peruzzi
Luca Peruzzotti-Jametti
Farzana Perwad
Guy Peryer
Giovanni Pes
Todd Pesavento
Angelo Peschiaroli
Bernice Pescosolido
Melanie Pescud
Mario Pesendorfer
Hitesh Peshavariya
Michael Peshkin
Francesca Pesola
Graziano Pesole
Anu-Katriina Pesonen
Juliano Pessan
Gustavo Pessin
Augusto Pessina
Silvina Pessino
Bruno Pessoa
Luiz Pessoa
David Pestana
Mario Pestarino
Franco Pestilli
William Pestle
Ekaterine Pestvenidze
Marko Pesu
Liisa Petaja
Ferenc Petak
Theodora Petanidou
Metin Petek
Adrian M. Peter
Andreas Peter
Claudio Peter
Daniel Peter
Eibich Peter
Jonathan Peter
Karlheinz Peter
Marcus Peter
Trevor Peter
Angel Peterchev
Robert Peterka
Renata Peterkova
Amber Peterman
Thomas Peterman
B. Peters
Bjoern Peters
Brian Peters
Christian Peters
David Peters
Dorien Peters
Frank Peters
Isabella Peters
Jeffrey Peters
Jessica Peters
Joerg Peters
Joseph Peters
Madelon Peters
Megan Peters
Michael Peters
Nathan Peters
Ole Peters
Oliver Peters
Remco Peters
Ryan Peters
Wibke Peters
Abdul Petersen
Alexander Petersen
Bent Petersen
Bjørn Petersen
Brenden Petersen
C.B. Petersen
Chris Petersen
Christine Petersen
Elijah J. Petersen
Eskild Petersen
Inge Petersen
Johanna Petersen
Kitt Petersen

Kyle Petersen
Matt Petersen
Rasmus Petersen
Stephen Petersen
Svend Petersen-Mahrt
Catherine Peterson
Charles Peterson
Christian Peterson
Courtney Peterson
Daniel Peterson
David Peterson
Ellena Peterson
Gregory Peterson
Hikaru Hanawa Peterson
James Peterson
Jen Peterson
K.M. Peterson
M. Nils Peterson
Paul Peterson
Sean Peterson
Shelley Peterson
Stephen Peterson
Thomas Peterson
Townsend Peterson
William Peterson
Arthur Peterson Jr.
Kevin Pethe
Rohan Pethyagoda
Janos Peti-Peterdi
Christine Petit
Gael Petitjean
James Petitte
Eleni Petkari
Nikolai Petkau
Georgi Petkov
Petko Petkov
Stoyan Petkov
Carmen Lucia Petkowicz
Tunde Peto
Frank Petrak
Melissa Petrakis
Panagiotis Petrantonakis
Václav Petráš
Jeremy Petravicz
Benjamin Petre
Antonello Petrella
Fausto Petrelli
Oleksi Petrenko
Valery Petrenko
Ana Maria Roxana Petrescu
William Petri Jr.
Brian Petrich
K. Petrides
Dennis Petrie
Vilma Petrikaite
Ilona Petrikovics
Marco Petrillo
Antoniu Petris
Andrea Petróczi
Andy Petroianu
Walter Matthew Petroll
John Petros
Nicola Petrosillo
Megan Petrov
Anna Petrova
Mila Petrova
Constantinos Petrovas
Andraž Petrovcic
Isidora Petrovic
Natasa Petrovic
Zoran Petrovic
Goran Petrovski
Antonio Petrucci
Orlando Petrucci
Aras Petrulis
Adam Petrusek
Clive Petry
Rolfe Petschek
Elizabeth Petsios
Con Petsoglou
Tristan Pett
Salvatore Petta
Elijah Petter
Michaela Petter
Alexander Petter-Puchner
Gunn Pettersen
Susan Petterson
Carloalberto Petti
Melinda Pettigrew
Matteo Pettinari
April Pettit
Stephen Pettitt
Harri Pettitt-Wade
Julien Pettré
Fiorenzo Peverali
Joshua Pevnick
Penny Pexman
Payam Peymani
Adrien Peyrache
Marisa Peyre
Jean-Francois Peyron
Catherine Peyrot des Gachons

Leo Pezzementi
Ilaria Pezzini
Marcus Pezzolesi
Gianni Pezzoli
Luciano Pezzullo
Francesca Pezzuto
Daniela Pfabigan
Alexander Pfaff
Donald Pfaff
Michael Pfaller
Jesse Pfammatter
Boris Pfander
Joachim Pfannschmidt
T. Joshua Pfefer
Lawrence Pfeffer
Ulrich Pfeffer
Carmem Pfeifer
Roman Pfeifer
Yvonne Pfeifer
Andreas Pfeiffer
Michael Pfeiffer
Ronald Pfeiffer
Pascal Pfiffner
Kurt Pfister
Riccardo Pfister
Roman Pfister
Hans Pflueger
Stephen Pflugfelder
Elizabeth Pfoh
Carmen Pfortmueller
Fabian Pfrengle
Chetan Phadke
Suhas Phadnis
Ben Phalan
David Phalen
Phuong-Thu T. Pham
Tuan Pham
Viet-Thanh Pham
Gilles Phan
Gregg Phares
Anastasia Pharris
Binita Phartiyal
Carmen Pheiffer
Paul Phelan
Benjamin Phelps
Toby Phesse
Suzanne Phibbs
Colin Phifer
John Philbeck
Michael Philben
Ingrid Philibert
Benjamin Philip
Pierre Philip
Manfred Philipp
Marielle Philippens
B. Philips
Nolan Philips
Lou Philipson
Veit Phillip
William Phillip
Andrea Phillipou
Brian Phillippy
Andrew Phillips
Anna Phillips
Anna C. Phillips
Barbara Phillips
Clive Phillips
David Phillips
Donald Phillips
Jacqueline Phillips
James Phillips
Jeffrey Phillips
John Phillips
Julie Phillips
Justin Phillips
Kevin Phillips
L. Alison Phillips
Margaret Phillips
Mark Phillips
P. Jonathon Phillips
Phoebe Phillips
R. Stephen Phillips
Richard Phillips
Richard E Phillips
Robert Phillips
Ruth Phillips
Scott Phillips
Stuart Phillips
Susan Phillips
Timothy Phillips
Zachary Phillips
Penelope Phillips-Howard
Andy Philp
Carl Philpott
Caroline Philpott
Stuart Phinn
Wanda Phipatanakul
Richard Phipps
Warren Phipps
Edward Phlips
Mai Pho
Florence Phocas

Jack Phu
Lee Cheng Phua
Suparat Phuanukoonnon
Alisa Phulukdaree
Quang Phung
Son Lam Phung
Leonidas Phylactou
Lorenzo Pia
Giorgio Piacentini
Alberto Piaggesi
Paolo Piaggi
Antoinette Piaggio
Vitoria Piai
Jean-Philippe Pialasse
Vincent Pialoux
Stefano Piana
Maria Grazia Piancin
Stefano Pianta
Steven Piantadosi
Zhongyun Piao
Jean-Christophe Piard
Julia Piaskowski
John Piatt
Adriano Piattelli
Bryan Piazza
Gary Piazza
Ornella Piazza
Roxane Piazza
Luigi Piazzi
Manuela Piazzi
Ana Picado
Brigitte Picard
Nicolas Picard
Mathieu Picardeau
Ernesto Picardi
Gianluca Picariello
Jean Charles Picaud
Serge Picaud
Antonio Picazo-Mozo
Pier Paolo Piccaluga
Daniel Picchietti
Jonathan Piccini
Camila Piccinin
Giuseppe Piccione
Alessandra Piccirillo
Claudia Piccoli
Giorgina Piccoli
Brian D. Piccolo
Alessandro Picelli
Pietro Picerno
Muriel Pichavant
Martin Pichler
Andreas Pichlmair
Aurélien Pichon
Chantal Pichon
Fiona Pichon
Sabrina Pichon
Claude Pichonnaz
Kathy Pichora-Fuller
Iva Pichová
Jean-François Picimbon
Andrea Picin
Neora Pick
Benjamin Pickard
Amy Pickering
Gisèle Pickering
John Pickering
Martin Pickering
Paula Pickering
Justin Pickett
Will Pickett
Anja Pickhard
Fernando Pico
Joanna Picot
Stéphane Picot
Sandrine Picq
Anabela Picton
Helen Picton
Stefano Pidello
Myroslav Pidkuyko
Trevor Piearce
Karin Pieber
Pedro Piedra
Jesus Piedrafita
Jorge Piedrahita
Lorenzo Piemonti
Martin Pienkowski
Robert Pieper
Ursula Pieper
Hans-Peter Piepho
Riccardo Pierantoni
Denis Pierard
Brad Pierce
Brian Pierce
G. N. Pierce
Lamar Pierce
Jonathan Pierce-Shimomura
Michele Piercey-Normore
Julius Piercy
Luca Pierelli
Mario Pierik
Carlo Piermarocchi

Sabata Pierno
Marco Pieroni
Elizabeth Pierson
Jennifer Pierson
Leland Pierson III
Mariusz Pierzchala
Marlien Pieters
Roel Pieters
Tiziana Pietrangelo
Rosemeire Pietro
Ricardo Pietrobon
Michael Pietrock
Susanna Pietropaolo
Michael Pietrusewsky
Mariusz Pietruszka
Maciej Pietrzak
Lukasz Pietrzyk
Uwe Pietrzyk
Marcin Pietrzykowski
Laura Piffer
Simone Pifferi
Antonio Piga
Matteo Piga
Gwenael Piganeau
Jon Piganelli
Gabriel Pigeon
Marie Pigeyre
Kathleen Pigg
Kurt Piggott
Claudio Pignata
Patrizia Pignatti
Domenico Pignone
Lasse Pihlstrøm
Youry Pii
Pim Pijnappel
Tom Pike
Suzanne Pilaar Birch
Eleftherios Pilalis
Andrea Pilastro
Conrad Pilditch
Henriette Pilegaard
Kirsten Pilegaard
Joseph Pilewski
Diogo André Pilger
Erik Pilgrim
Adrian Piliponsky
Pamela Pilkington
Anilkumar Pillai
Dinesh Pillai
Jagan Pillai
Prakash Pillai
Radhakrishna Pillai
Shiv Pillai
Smitha Pillai
Vikram Pillai
Ajay Pillarisetti
Venu Pillarisetty
Viness Pillay
Evangéline Pillebout
Michiel Pillet
Elena Pilli
Darrell Pilling
Luke Pilling
David Pilliod
Alexis Pillsbury
Praveen Pilly
Marc Pilon
Marinus Pilon
Karsten Pilones
Violetta Pilorz
Andrea Pilotto
Sara Pilotto
Lara Pilutti
Sergei Pilyugin
Luisa Pilz
David Pim
Daniel Pimenta
Fabiana Pimenta
Luiz Pimenta
David Pimentel
Laura Pimentel
Mark Pimentel
Tatiana Pimentel
Stuart Pimm
François Pimont
Christopher Pin
Chueh Pin Ju
Benjamin Piña
Miguel Pina e Cunha
Mariona Pinart
Maria Dolores Pinazo-Duran
Díaz-Jaimes Píndaro
Ross Pinder
Dominika Pindus
Pascal Pineau
Nicolas Pineault
Federico Pineda
Fernando Pineda
Mercé Pineda
Miguel Pineda
Ruben Pineda-Lopez
Nicolás Pineda-Trujillo

Bárbara Piñeiro
Fulvio Pinelli
Heather Pines
Pablo Pineyro
Yuan Ping
Alessandro Pingitore
Leora Pinhas
Ana Pinheiro
Bruno do Valle Pinheiro
Céline Pinheiro
Eloan Pinheiro
Hudson Pinheiro
Joaquim Pinheiro
Rejane Pinheiro
Armando Pinho
Mariana Pinho
Suani Pinho
Vanessa Pinho
Simon Pini
Ryan Pink
Ronit Pinkas-Kramarski
Michael Pinkawa
Zsolt Pinke
Katja Pinker
JoAnn Pinkerton
Kent Pinkerton
Porntip Pinlaor
Somchai Pinlaor
Graziano Pinna
Gavin Pinniger
Joan Pino
Marco Pino
Sara Pinosio
Maria Pino-Yanes
Frank Pintar
Lionel Pintard
Camelia-M Pintea
Emmanuel Pinteaux
Daniela Pinter
Sándor Pintér
Lauren Pinter-Brown
Marcello Pinti
Amit Pinto
Daniel Pinto
Francisco Pinto
João Pinto
Jose Pinto
Lancelot Pinto
Luciana Pinto
Luísa Pinto
Marta Pinto
Miguel Pinto
Míriam Pinto
Rafael Pinto
Ricardo Pinto
Serge Pinto
Rosa Pintó
Perpétua Pinto-do-Ó
Filipa Pinto-Ribeiro
Sara-Joan Pinto-Sietsma
Jillian Pintye
Nina Pintzinger
Rianne Pinxten
Ilka Pinz
David Pinzon
Natalia Pinzon
Martha Pinzón Daza
Marco Piola
Daniele Piomelli
Nicolas Pionnier
Katarzyna Piórkowska
Niels Piot
Olivier Piot
Jeff Piotrowski
Tatjana Piotrowski
Cyril Piou
Rory J. Piper
C. Pipili
Daniel Pique
Raquel Piqué
Laura Piqueras
Stefano Piraino
Antonio Piralla
Elena Pirani
Fabrizio Piras
Federica Piras
Joachim Pircher
Karolina Pircs
Leila Pirdel
Camilla Pires
Carmen Pires
José C.M. Pires
Liliana Pires
Ricardo Pires
Ricardo A. Pires
Sara Pires
Thiago Pires
Vasil Pirgozliev
Jouko Pirhonen
Fraser Pirie
Steven Pirie-Shepherd
Primoz Pirih

Angela Pirillo
Edibe Pirincci
Christian Pirk
Markus Pirklbauer
Sergej Pirkmajer
Jean-Paul Pirnay
Rosario Piro
Carlos Pirola
Enrico Pirotta
Julien Pirrello
Antonio Pisabarro
Yusuf Pisan
Didier Pisani
Laura Pisani
Katarzyna Pisanski
Renato Pisanti
Benoît Pisanu
Liudmila Pisarchyk
Tobias Pischon
Pietro Pisciotta
Salvatore Piscuoglio
Joe Pisegna
Joseph Pisegna
Pramod Pisharady
Michael Pishvaian
Jakub Piskorski
Alberto Pisoni
Emidio Pistilli
Francesca Pistoia
Vito Pistoia
Gina Cecilia Pistol
Massimo Pistolesi
David W. Piston
Wojciech Pisula
Teerat Pitakrat
Benjamin Pitcher
Brandelyn Pitcher
Mark Pitcher
Patrick Pithua
Alexandra Pitman
Robert Pitman
Dario Pitocco
Bertram Pitt
Bruce Pitt
Geoffrey Pitt
Jason Pitt
Mark Pitt
Dipti Pitta
Anna Pittaluga
Stefania Pittaluga
Sharon Pitteri
Jarmila Pittermann
Mauro Pittiruti
Alexandros Pittis
H. Pittman
Quentin Pittman
Letizia Pitto
R. Pitts
Susanne Pitz
Erik Pitzer
Francesco Piva
Nela Pivac
Rosario Pivonello
Lukasz Piwek
Fiona Pixley
Sarah Pixley
Fabio Pizza
Diego Pizzagalli
Roberto Pizzala
Milos Pjanic
Jesús Pla
Patrick Pla
Dimitris Placantonakis
Nicolas Place
Sandra Plachta-Danielzik
Chris Plack
Timothy Plageman
Léon Plaghki
Celine Plainvert
Eric Plaisance
Laetitia Plaisance
J. Plaizier
Oleguer Plana-Ripoll
Frances Plane
Vicente Planelles
Francisco Planes
Paul Planet
Barbara Plank
Michael Plank
Benjamin Planque
Giles Plant
Richard Plant
Tim Plant
Elena Plante
Anna Plantinga
Laura Plantinga
Myriam Plantinga
Nienke L. Plantinga
Jeremiah Plass-Johnson
Angel R. Plastino
Jonathan Platkiewicz
Jeffrey Platt

Jodyn Platt
Manu Platt
Robert Platt
Brandon Plattner
Adrian Platts
James Platts-Mills
Thomas Platts-Mills
Maria Platzeck
Ivan Platzek
Davor Plavec
Jessica Plavicki
Enrique Playán
Grazyna Plaza
Daniel Plaza-Bonilla
Agnieszka Plazek
Melanie Plazy
Katherine Pleasants
R. A. Pleasants
David Pleasure
Mario Plebani
Milda Pleckaityte
Kristen Pleil
Jürgen Pleiss
Richard Plemper
Paul Plener
Stephanie Plenty
Michael Plenzler
Nancy Pleshko
Charles Plessy
Steven Pletcher
Daniel Pletinckx
Leos Pleva
Thorsten Plewan
Triantafyllos Pliakas
Maksim Plikus
Sergey Plis
George Plitas
Maciej Plocharski
Martin Plöderl
Annemie Ploeger
Randy Ploetz
Markus Plomann
Peter Plomgaard
Robert Plomin
Stephanie Plon
Alexander Ploner
Przemyslaw Plonka
Ori Plonsky
Stefan K. Plontke
Diego Ploper
Victoria Ploplis
Charles Plopper
Balbina Plotkin
Lilian Plotkin
Marya Plotkin
Ronald Plotnikoff
Sergey Plotnikov
Brian Plouffe
Jeff Plowman
Raina Plowright
Geneviève Plu-Bureau
Jessica Plucain
Alessandro Pluchino
Nicola Pluchino
Mateusz Plucinski
Graham Pluck
Jonathan Plucker
Pawel Pludowski
Mark Plumbley
Jackie Plumbridge
Andrew Plunk
Gerd Pluschke
Tomáš Pluskal
John Pluske
Janice Pluth
Eftychios Pnevmatikakis
Dominic Poccia
Radhika Pochampally
Thomas Pochapsky
Roland Pochet
Oleh Pochynyuk
Nicholas Pocock
Klaus Podar
Anthony Podberscek
Andreas Podbielski
Kenneth Podell
Manuel Podestà
Michela Podestà
Osvaldo Podhajcer
Radka Podlipná
Vedran Podobnik
Viktor Podolskiy
Jerrod Poe
Jan-Hendrik Poehls
Daniel Poehnert
Tamie Poepping
Wolfgang Poeppl
A. Poff
Paolo Poggio
Joe Pogliano
Marko Poglitsch
Gennady Pogorelko

Claudia Pogoreutz
Maksym Pogorielov
Catherine Poh
Chit-Laa Poh
Tuang Yeow Poh
Raimo Pohjanvirta
Carolina Pohl
Christian Pohl
Ehmke Pohl
Martin Pohl
Sandra Pohl
George Poinar
Laurent Poirel
Steve Poirier
Yves Poirier
S. Poitras
Isabelle Poizot-Martin
Martina Pokorná
Bostjan Pokorny
Jennifer Pokorny
Mieczyslaw Pokorski
Katja Pokrovskaja Tamm
Edith Poku
Olga Pol
Norbert Polacek
Christopher Polage
Rani Polak
Lillian Polanco
Kinga Polanska
Jaroslaw Polanski
Paula Polastri
Andrew Polaszek
Hüseyin Polat
Onur Polat
Özge Polat
Bart Polder
Don Poldermans
Ludmila Polechonska
Lubos Polerecky
Jerry Polesel
Caroline Polet
Chiara Poletto
Steven Polevoi
Zita Polgár
Linnea Polgreen
Alessandro Poli
Andrea Poli
Giuseppe Poli
Carlos Poli De Figueiredo
Eugenia Poliakov
Anne Poliard
Benjamin Policicchio
Alberto Policriti
David Polidori
Maria-Cristina Polidori
Igor Polikarpov
Renato Polimanti
Kishore Polireddy
Luisa Politano
Luis Politi
Pierluigi Politi
Ioannis Politikos
Dimitris Politis
Panagiotis Politis
Cristina Polito
Michael Polito
Zoe Polizopoulou
Karen Polizzi
Mark Polizzotto
Anne Poljak
Zvonimir Poljak
Brent Polk
D. Brent Polk
Deborah Polk
Jessica Polka
Adam Polkinghorne
Gerard Poll
Craig Pollack
Gerald Pollack
Harold Pollack
Harvey Pollack
Jonathan Pollack
Daniella Pollak
Yehuda Pollak
Marina Pollán
Harvey Pollard
Joshua Pollard
Katie Pollard
Rachel Pollard
Stefania Pollastro
David Poller
Ingrid Pollet
Thomas Pollet
Spencer Polley
Alex Pollitt
Antonella Pollo
Jennifer Pollock
Michael Pollock
Erqi Pollom
Riley Pollom
Bart Pollux
P. Polly

Daniel Pollyea
Olli Polo
Karol Polom
Soraia Poloni
Tamar Polonsky
Riccardo Polosa
Jeffrey Polovina
Samuel Poloyac
Sarit Polsky
Annikka Polster
Tobias Polte
Elisabetta Poluzzi
Eva Polverino
Lina Polvi Sjöberg
Maksym Polyakov
Constantin Polychronakos
Georgios Polychronidis
Nikolaos Polyzos
David Polzin
Lars Pomara
Constança Pomba
Marco Pombi
Carrie Pomeroy
V. Pomeroy
Régis Pomès
Vitor Pomin
Celine Pompeia
Michel Pompeu
Paulo Pompeu
Maurizio Pompili
James Pomposelli
Kyle Pomraning
Liza Pon
Gabriel Ponce de Leon
Adrian Ponce-Alvarez
Julia Poncela-Casasnovas
Jean Christophe Poncer
Aurore Ponchon
Willem Pondaag
Satyanarayana Pondugula
Jan Ponert
Jean-Francois Ponge
Lauren Ponisio
Rakesh Ponnala
V.K. Chaithanya Ponnaluri
K. Ponnambalam
Sreenivasan Ponnambalam
Moorthy Ponnusamy
Ivo Ponocny
Maite Pons
Roser Pons
Peter Ponsaerts
Cyriel Ponsioen
Anne-Louise Ponsonby
Siriluck Ponsuksili
Roberto Pontarolo
Pierre Pontarotti
Alicia Ponte Sucre
Eleonora Ponterio
Jerome Ponthier
Claudia Pontillo
Herman Pontzer
Gilles Ponzio
Marina Ponzio
Riccardo Ponzone
Eva-Maria Pool
John Pool
Alastair Poole
C.J. Poole
Daniele Poole
David Poole
Karen Poole
Robert Poole
Toni L. Poole
Simon Pooley
Liona Poon
Selvamuthu Poongulali
Anil Pooran
Jalal Poorolajal
Ate Poorthuis
Wouter Poortinga
Toney Poovelikunnel
Kittiyod Poovorawan
Yong Poovorawan
Shabnam Pooya
Ioan Pop
Aurel Popa-Wagner
Lizzy Pope
Phillip Pope
Welkin Pope
Mihail Popescu
David Popham
Piotr Popik
Daniel Popkin
Patrizia Popoli
Victor Popoola
Tzvetan Popov
Zoran Popovic
Kyle Popovich
Oleksandr Popovych
David Popp
Jürgen Popp

Brigitte Poppenberger
Arthur Popper
Holger Poppert
Simon Poppinga
Linda Popplewell
Harish Poptani
Christopher Porada
Camillo Porcaro
Davide Porcellato
Steven Porcelli
Raphaël Porcher
David Porciani
Giuseppina Porciello
Daniel Poremski
Luciana Porfirio
André Pornon
Peerapong Pornwongthong
Gustavo Porpino
Carolina Porras
Laura Porretti
Esteban Porrini
Carlo Porro
Janos Porszasz
Alberto Porta
Eduard Porta-Pardo
Stuart Portbury
Emma Portch
Robert Porte
Annabel Porté
Erja Portegijs
Anayda Portela
Margareth Portela
Anna Porter
Christopher Porter
Emily Porter
George Porter
James Porter
Jason Porter
Joseph Porter
Marc Porter
Megan L. Porter
Michelle Porter
Nada Porter
Ryan Porter
Warren Porter
Weston Porter
William Porter
Angel Porteros
Stéphanie Portet
Cosima Porteus
Damien Portevin
Christopher Portier
Javier Portilla
Carmen Portillo
José R. Portillo
Ezio Portis
Michael Portman
Allison Portnoy
David Portnoy
Sigal Portnoy
Alla Portnychenko
Felipe Porto
Igor Portoghese
José Portoles
Ana Porto-Pazos
Agustina Portu
Kevin J. Portune
Anna Porwit
Ana Porzecanski
Elena Porzio
Andrea Porzionato
Klaas Pos
Inmaculada Posadas
Anna Posadino
James Posakony
Charlotte Poschenrieder
Marshall Posner
Mason Posner
Nico Posnien
Helmut Pospiech
Andre Possani
Lourival Possani
Richard Possemato
Bernard Possidente
Gerald Post
Mark Post
Rory Post
Cosimo Posth
Michael Posthumus
Catherine Postic
Guillaume Postic
Anthony Postle
Arnold Postlethwaite
Albert Postma
Alex Postma
Eric Postma
Johannes Postma
Joseph Postman
Svetlana Postnova
Lynne-Marie Postovit
Marek Postula
Ronald Postuma

Olga Posukh
Caroline Pot
Zuzana Potacova
Tonia Poteat
Jan Potempa
Luciano Potena
Arnaud Poterszman
Raffaello Potestio
Charalabos Pothoulakis
Bhavana Pothuri
Francesco Poti
Jennifer Poti
Rahul Potluri
Eric Potma
Neha Potnis
Brian Potoski
Mark Potosnak
Hans Pottel
Barry Potter
Daniel Potter
Kathleen Potter
Kevin Potter
Lee Potter
Lesley Potter
Mary Potter
S. Steven Potter
Sally Potter
W. Potter
Matthew Potthoff
Jaime Potti
Christophe Pottier
Nicolas Pottier
Donald Potts
Geoffrey Potts
Matthew Potts
Ryan Potts
Richard Potvin
Patrice Poubelle
Resham Poudel
Pierre Pouget
Mahmoud Pouladi
Bernard Poulain
Nikos Poulakakis
Serge Poulet
Dimitra Pouli
Elie Poulin
Lionel Poulin
Sebastien Pouliot
Thomas Poulos
Mads Poulsen
Nicole Poulsen
Simon Poulson
John Poulton
George Poultsides
Michael Pound
Nicholas Pound
Pandora Pound
Atharva Poundarik
Lucie Poupel
Maria Poupin
Mohsen Pourahmadi
Christine Pourcel
Michael Pourfar
Farshad Pourmalek
Evangelos Pournaras
Spyros Pournaras
Philippe Pourquier
Olivier Pourret
Kambiz Pourrezaei
Mohammad Pourshafie
Gunilla Pousette Lundgren
Susan Poutanen
Koen Pouwels
Simon D. Pouwels
Eva Poveda
Katja Poveda
Megan Povelones
Johan Povlsen
Desmond Powe
Amy Powell
Anna Powell
Colin Powell
Daniel Powell
David Powell
Don Powell
Douglas Powell
Frank Powell
Jessica Powell
Joann Powell
Kevin Powell
Madison Powell
Marta K. Powell
Mickie Lynn Powell
Penny Powell
Tara Powell
Tiffany Powell
Warren Powell
Tiffany Powell-Wiley
Anne Marie Power
Chris Power
Christopher Power
David Power

Eileen F. Power
Jennifer Power
Michael Power
Michelle Power
Thomas Power
Evan Powers
Randal Powers
Robert Powers
Thomas Powers
Simon Powis
Sue Pownall
Mathew Poy
D.R. Poyner
Mary Poynten
Matthew Poynter
Necdet Poyraz
David Poza
Nikita Pozdeyev
Alex Pozhitkov
Gabor Pozsgai
Antonio Pozzi
Nishi Prabdial-Sing
Chandra Prabhakar
Poornima Prabhakaran
Satria Arief Prabowo
Sophie Prache
Brenda Pracheil
Fiorella Prada
Ana E. Pradas del Real
Pierre-François Pradat
Rolf Prade
Appukuttan Pradeep
B. E. Pradeep
Wagle Pradeep
P. Pradeepkumar
Elizabeth Pradel
Jean Philippe Pradere
Abani Pradhan
Arun Pradhan
Jalandhar Pradhan
Prajal Pradhan
Pranil Pradhan
Kearkiat Praditpornsilpa
Julia G. Prado
Maria Prado
Pavel Prado
Sara Prado
Soizic Prado
Vitor Prado
Heriberto Prado-Garcia
Alexandra Prados-Torres
Manuel Praga
Sean Prager
Alexa Pragman
Samir Kumar Praharaj
Arun Prakash
Hridayesh Prakash
Siddharth Prakash
Y.S. Prakash
Megha Prakash Bangalore
Niki Prakoura
Jan O. Pralits
Vincent Praloran
Pairot Pramual
Vinca Prana
Fabio Pranovi
Anamika Prasad
Anantha Prasad
Asuri Prasad
Kameshwar Prasad
Konasale Prasad
Narayan Prasad
Rajendra Prasad
Sahdeo Prasad
Sandip Prasad
Vikas Prasad
Vinayaka Prasad
Vinod Prasad
Vishal Prasad
Prateek Prasanna
Ondrej Prášil
Antje Prasse
Aleix Prat
Jordi Prat
Salome Prat
Lorenza Pratali
Aditya Pratap
Bhim Pratap
Morgan Pratchett
Timothy Prather
Francesca Prati
Christine Pratilas
Emanuela Prato-Previde
Anne-Catherine Prats
Clara Prats
Harris Pratsinis
Guy Pratt
Kathleen Pratt
Philip Pratt
Rex Pratt
Wayne Pratt
Guillem Pratx

Devarsetty Praveen
Shelly Praveen
Sarva Mangala Praveena
Dirk Prawitt
Josef Prchal
Jochen Prehn
Kristin Prehn
Gerd Prehna
Alexander Prehn-Kristensen
Christian Preisinger
Evan Preisser
Karina Preiss-Landl
Klaus Preissner
Drazen Prelec
Christina Prell
Tino Prell
Martina Prelog
Roman Prem
Thyagaseely Premaraj
Glenn Preminger
Francesc Prenafeta
Garreth Prendergast
Luke Prendergast
Mark Prendergast
Lorenzo Prendini
John Prenter
Heather Prentice
Howard Prentice
Marc Prentki
Daniel Preotiuc-Pietro
Fabio Presaghi
John F. Prescott
Steven Prescott
Sharon Presnell
Eva Pressman
Peter Pressman
Catherine Preston
Daniel Preston
Nancy Preston
Samuel Preston
Federico Preti
Ana Preto
Carel Pretorius
Luca Pretti
Paolo Pretto
Claudia Preuschhof
Christoph Preuss
Nora Preuss
Ulrich Preuss
A. Preutthipan
D. Prevedello
Peter Prevelige
Janet Prevey
Michael Previs
Stephen Previs
Martine Prévost
Bernard Prévosto
D. Rebecca Prevots
Oliver Preyer
Luigi Preziosi
John Priatel
Alison Price
Andrea Price
Catherine Price
Christopher Price
Gregory Price
Jon Price
Julie Price
Lawrence Price
Matthew Price
Max Price
Michael Price
Nathan Price
Rebecca Price
S. Price
Stephen Price
T. Douglas Price
Theodore Price
J. Roxanne Prichard
David Pride
Brendan Prideaux
Peter Pridmore
Tony Pridmore
Dominique Prié
Teresa Priego
Feliciano Priego-Capote
Olaf Prieske
James Priest
Naomi Priest
Anne Prieto
Claudia Prieto
Jaime Prieto
Miguel Prieto
Minolfa Prieto
Victor Prieto
Cesar Prieto de Frias
Angeles Prieto-Fernandez
David Prieto-Merino
Xavier Prieur
Christiane Prifert
Kostas Priftis
Claire Prigent-Combaret

Sean Prigge
Iva Prikrylová
Michelle Primeau
Stefany Primeaux
Ana Ligia Primo
Fernando Primo
John Primrose
M. Prin
Meghan Prin
A. Matthew Prina
Alice Prince
Lawrence Prince
Lea Prince
Martin Prince
Stephanie Prince
Mary Princip
Giovanni Principato
Mariabeatrice Principi
Nicola Principi
Pamela Principi
Anne Pringle
Jamie Pringle
Cindy Prins
Jan-Bas Prins
Maarten Prins
Robert Prins
Erik Prinsen
Sosja Prinsen
Linda Prinsloo
Andreas Prinzing
Adriano Priola
Anders Prior
Ian Prior
Kirsten Prior
Lynda D. Prior
Catarina Prista
Antonia Pritchard
Catrin Pritchard
D. Pritchard
Kirkwood Pritchard
Victoria Pritchard
Timothy Pritts
Sivan Priya
Aashish Priye
Janice Probst
Anne-Katrin Pröbstel
Rainer Probstmeier
Claudio Procaccini
Micah Prochaska
Vaclav Prochazka
Giuseppe Procopio
Darby Proctor
Michael Proctor
Richard Proctor
Robert Proctor
Steven Proctor
William Procunier
Flavia Prodam
Roger Prodon
Chrisostomos Prodromou
Catherine Proenza
Willem Proesmans
Antonios Proestakis
Antonio Profico
Thomas Proft
Stefano Profumo
Jaime Prohens
Alan Proia
Joe Proietto
Laszlo Prokai
Victor Prokhorenko
Peter Proksch
David Prole
Jeanine Prompers
Vasilis Promponas
Supannee Promthet
Paul Pronyk
Leanne Proops
David Propper
Ewgenij Proschak
Laura Prosdocimi
Katarina Prosenc
Mikhail Proskurnin
Chiara Prosperetti
Ennio Prosperi
Mattia Prosperi
Luca Prosperini
Melinda Protani
Bartosz Protas
Elizabeth Protas
Athanassios Protopapas
Kostantinos Protopapas
Natacha Protopopoff
Alexandra Protopopova
John Protzko
David Proud
Amanda Proudfoot
Christos Proukakis
Mariano Provencio
Pietro Provenzale
Paolo Provenzano
Alice Mado Proverbio

Mauro Provinciali
Robert Provine
Federica Provini
Sharen Provoost
Alexander Provost
Sylvain Provot
Natalie Prow
John Prowle
Tracy Prowse
Emil Proynov
Mario Prsa
James Pru
Miroslav Prucha
Carlos Prudencio
Miguel Prudêncio
Marion Prudent
Wendy Prudhomme O'Meara
Igor Prudovsky
Birgit Pruess
Stephen Pruett
Timothy Pruett
Jill Pruetz
Menno Pruijm
Raimon Pruim
A.A. Pruitt
Julien Prunier
Steven Prus
Przemyslaw Prusinkiewicz
Aaron Prussin
Calman Prussin
Annette Prüss-Ustün
Yifat Prut
Priit Pruunsild
Melanie Pruvost
Christopher Pryce
Josephine Pryce
Rob Pryce
Wojtek Przepiorka
Ingo Przesdzing
Andrew Przybylski
Piotr Przybysz
Eryk Przysucha
Lilia Psalta
Alkis Psaltis
Androniki Psifidi
Radek Ptacek
Petr Ptácek
Marcin Ptaszek
Christoph Ptatscheck
Christy Pu
Jeffrey Pu
Juan Pu
Jun Pu
Shenghong Pu
Ye Pu
Yi Pu
Yonglin Pu
Steve Publicover
Annibale Puca
Angela Pucci
Giacomo Pucci
Nancy Puccinelli
Marzia Puccioni-Sohler
Michel Puceat
Adam Puche
Carlo Pucillo
Joanna Pucilowska
James Puckett
Shiva Pudasaini
Haridas Pudavar
Paolo Emilio Puddu
Jardena Puder
Alexander I. Pudovkin
Raghavendra Sumanth Pudupakam
Sébastien Puechmaille
Anne Puel
Jean-Luc Puel
Carlos Puente
Chloe Puett
Vanessa Puetz
Miles Pufall
Eve Puffer
Hélder Puga
Irina Pugach
Oksana Pugach
Elena Pugacheva
Ganesan Pugalenthi
Subbiah Pugazhenthi
Carla Pugh
Thomas Pugh
Anna Maria Puglia
Andrea Pugliese
Mariagabriella Pugliese
Edoardo Puglisi
Fabio Puglisi
Ching-Hon Pui
Montse Puigdelloses
Andreu Puigdollers
Amaya Puig-Kröger
Lorri Puil
Nicolas Puillandre
Alin Puinean

Iroh Tam Pui-Ying
Miguel Angel Pujana
Claude Pujol
Flor Pujol
Jean-Louis Pujol
Jose Martin Pujolar
Budhan Pukazhenthi
Ammi Pulagam
Lakshmi Pulakat
Elinor Pulcini
Bali Pulendran
Julie Pulerwitz
Cedric Puleston
Francisco Pulido
Jose Pulido
David Pulido-Velazquez
Vinesh Kumar Puliyappadamba
Jacob Puliyel
Livia Puljak
Matthew Pullen
Nicholas Pullen
Wim Pullen
Craig Pulling
Helen Pullisaar
Mohammad Pulok
Alfredo Pulvirenti
Dietmar Pum
Denise Pumain
René Pumain
Sher Pun
Sachin Pundhir
Anna Punga
Nalini Puniamoorthy
Kumaradevan Punithakumar
Anil Puniya
Rajesh Punn
Tracy Punshon
Michelino Puopolo
Fulvio Pupilli
Elisabetta Pupillo
André Pupo
Naveen Puppala
Francesca Puppo
Swati Puranik
Jennifer Purcell
Jessica Purcell
Auriol Purdie
Paul Purdom
Deirdre Purfield
Aaron Puri
Ishwar Puri
Prem Puri
Sunil Puria
Ruma Purkait
Samuel Purkis
Mark Purnell
Sarah Purnell
Kristen Purrington
Zachary Pursell
Jessica Purswani
Richard Purves
Katarzyna Purzycka
Benjamin Purzycki
Snigdhasmrithi Pusalavidyasagar
Antonio Pusceddu
Andreas Püschel
Gerhard Püschel
Birgit Puschner
Smruti Pushalkar
M. Pushie
Martin Puskarjov
László Puskás
Krzysztof Puszynski
Nirupama Putcha
Shuby Puthussery
Vesa Putkinen
David Putnam
David Puts
Radhika Puttagunta
Amar Puttanna
Christian Puttlitz
Santhoshkumar Puttur
Brigitte Pützer
Eric Puzenat
Rami Puzis
Alessandro Puzziello
Timothy Pychyl
Saiprasad Pydi
David Pye
Alyssa Pyke
Aryn Pyke
Eva-Theresa Pyl
David Pyle
Lacey Pyle
Laura Pyle
Peter Pyle
Liina Pylkkanen
Magdalini Pylli
Alexander Pym
Petr Pysek
Kristin Pytynia
Okko Pyykkö

Motaz Qadan
Firdausi Qadri
Saber Qanbari
Khaled Qanud
Atif Qasim
Muhammad Qasim
Waseem Qasim
Amal Qattan
A. Qayyum
Dongchen Qi
Donglai Qi
Dunwu Qi
Haitao Qi
Hong Qi
Hongbo Qi
Hongsheng Qi
Ji Qi
Jinsheng Qi
Ruifeng Qi
Ruomei Qi
Shaohai Qi
Wenyuan Qi
Xiang Qi
Xiaoyang Qi
Xin Qi
Xinshuai Qi
Xue-Bin Qi
Yang Qi
Yin Qi
Yun Qi
Zhi Qi
Chao-Nan Qian
Cheng Qian
Feifei Qian
Haifeng Qian
Haohua Qian
Hong Qian
Jiang Qian
Jianjun Qian
Jiayi Qian
Jingjing Qian
Jiwei Qian
Jun Qian
Linmao Qian
Min Qian
Shanshan Qian
Wei Qian
Wenxue Qian
Xi Qian
Xiaohua Qian
Xu Qian
Yu Qian
Yuhua Qian
Yuntao Qian
Zhaohui Qian
Zichen Qian
Ya-Wei Qiang
Aijun Qiao
Aike Qiao
Fangbin Qiao
Ge-Xia Qiao
Jie Qiao
Li-Ya Qiao
Tie Qiao
Xiu-Chen Qiao
Yingli Qiao
Udi Qimron
Bo Qin
Bolin Qin
Daoming Qin
Feng Qin
Fujun Qin
Genji Qin
Guozheng Qin
Hongwei Qin
Jian Qin
Jun Qin
Lei Qin
Li Qin
Qiwei Qin
Shuhao Qin
Wen Qin
Xiang Qin
Yi-Xian Qin
Yuan Qin
Zhixuan Qin
Yong Qing
Zhang Qingxue
Dong Qiongzhu
Bao-Li Qiu
Daowen Qiu
Deqiang Qiu
Dong Qiu
Fan Qiu
H. Qiu
Hong Qiu
Hongyu Qiu
Hua-Ji Qiu
Jian-Ding Qiu
Jian-Wen Qiu
Jia-Xuan Qiu
Junzhi Qiu

Lijuan Qiu
Shuang Qiu
Sujun Qiu
Tian Qiu
Wei Qiao Qiu
Weigang Qiu
Weiliang Qiu
Wen-Yuan Qiu
Wusi Qiu
Xiangguo Qiu
Xiangyun Qiu
Xing Qiu
Xinying Qiu
Xiu Qiu
Yi Qiu
Yingwei Qiu
Yong Qiu
Zhaoxiong Qiu
Zhiying Qiu
Zhongmin Qiu
Chuanqiang Qu
Cunmin Qu
Feng Qu
Guiqin Qu
Jia Qu
Mingjing Qu
Rongda Qu
Shaojian Qu
Shenchun Qu
Weiguang Qu
Xian Qu
Xiaobo Qu
Xingda Qu
Xinhua Qu
Xinshun Qu
Yuan Qu
Zhilin Qu
Zhuohua Qu
Lindi Quackenbush
Mohammed Quader
Christian Quadri
Paulo Quadri
Sadiqa Quadri
Edward Quadros
Isabel Quadros
Susan Quaggin
Sylvia Quaggiotti
Vincenzo Quagliariello
Stella Quah
Im Quah-Smith
Emilio Quaia
Joseph Qualls
Cheng Quan
Chunshan Quan
Lei Quan
Lijun Quan
Melvyn Quan
Taihao Quan
Vanessa Quan
Zhe-Xue Quan
Emmanuel Quansah
Xing Quantai
Luciano Quaranta
Nicola Quaranta
L. Quarles
Alessandro Quartiroli
Andrea Quattrini
Mattia Quattrocelli
John Quattrochi
Walter Quattrociocchi
Cassandra Quave
Paul Quax
Alison Quayle
Qiudeng Que
Erhard Quebe-Fehling
Carrie Queenan
Felisbina Queiroga
Ana Queiros
Antonio Queiros
Nuno Queiroz
Frederick Quelle
Annaïk Quémard
Siobhan Quenby
Jean-Pierre Quenot
Tiago Quental
Josep Quer
Gaëlle Quéré
Uwe Querfeld
Pascal Querner
Amparo Querol
Luis Querol
Giuseppe Querques
Alberto Quesada
Antonio Quesada
Carlos Alberto Quesada
Ivan Quesada
Carlos Quesada-Gómez
Christophe Quesnel
Francois Quesque
Sylvain Quessy
Laurence Questienne
Mario Quevedo

Sandra Quezada
Martha Quezado
Nicola Quick
Virginia Quick
J.G. Quicke
Eamonn Quigley
Pearl Quijada
Maryka Quik
Anita Quiles
Fabienne Quiles
Marie-Laure Quilici
Elizabeth Quilliam
Jessica Quimby
Bo Quin
Thelma Quince
John Quindry
Jacklyn Quinlan
Cheryl Quinn
Elizabeth Quinn
Frederick Quinn
Graham Quinn
John Quinn
Kimberly Quinn
Kylie Quinn
Lauretta Quinn
Leonie Quinn
Niall Quinn
Rhonda Quinn
Thomas Quinn
Luis Quinone
Miguel E. Quinones
Miguel Quinones-Mateu
Eva Quinque
Clay Quint
Jennifer Quint
Giuseppe Quintaliani
Eduard Quintana
Francisco Quintana
Yasmin Quintana
Miguel Quintanilla
Andrea Quintero
Stacey Quintero-Wolfe
Cristiano Quintini
Richard Quinton
Joe Quirk
Lesliam Quiros-Alcala
Amanda Quisenberry
Lucia Rita Quitadamo
Robert Quivey
Jawwad Qureshi
Rahat Qureshi
Nir Qvit
Song-Gyu Ra
Eric Raabe
Thomas Raabe
Ulrike Raap
Morten Raastad
Morgan Raath
Susan Raatz
Bas Raaymakers
Victoria Rabago
Christopher Rábago
Jomar Rabajante
Heike Rabe
Njaratiana Rabeariosa
Jacob Raber
Walaa Rabie
Joseph Rabinowitz
Tom Rabinowitz
Raquel Rabionet
Felicia A. Rabito
Didier Raboisson
Catherine Rabouille
Wolfgang Rabsch
Ralf Rabus
Gwénaël Rabut
Brent Race
Lyne Racette
P. Sivaramakrishna Rachakonda
George Rachiotis
Howard Rachlin
Beth Rachlis
Shimon Rachmilevitch
Jacob Rachmilewitz
Richard Rachubinski
Anna Raciborska
Vitomir Racic
Karen Racicot
Rachel Racicot
Daniela Raciti
Gregory Raciti
Michael Racke
Catherine Racowsky
Lorraine Racusen
Dorota Raczynska
Balazs Rada
Zslot Radak
Alexander Radbruch
Viktoriia Radchuk
Nathan Radcliffe
Rolfe Radcliffe
Nicole Radde

Jeffrey Rade
Canella Radea
Rebecca Rademeyer
Aleksandra Radenovic
Christoph Rader
Daniel Rader
Janet Rader
Marcel Radermacher
Alan Radford
Ammu Kutty Radhakrishnan
Prakash Radhakrishnan
Ravi Radhakrishnan
Sabarinathan Radhakrishnan
Zoran Radic
Filippo Radicchi
Jerald Radich
Anita Radini
Kathy Radke
Rafael Radkowski
Elahe Radmaneshfar
Olof Radmark
Jelena M. Radojicic
Nina Radosevic-Robin
Miloš Radovanovic
Vesela Radovic
Tamás Radovits
Thomas Radtke
Roxana Radu
Andreea Radulescu
Marius Radulescu
Katarina Radulovic
François Radvanyi
Osman Radwan
Przemyslaw Radwanski
Robert Radwin
Jessica Radzio
Alasdair Rae
Lori Raetzman
Vladimir Raevsky
Anu Raevuori
Alex Rafacho
Ota Rafaela
Eshkol Rafaeli
Haloom Rafehi
Ali Rafei
Giuseppe Raffa
Tommaso Raffaello
Robert Raffai
Manuela Raffatellu
Glen Raffel
Pauline Rafferty
Thomas Rafferty
Ute Raffetseder
Milena Raffi
Johannes Raffler
Mahmoud Rafieian-Kopaei
Mohammad Rafienia
Shahin Rafii
Khadija Rafiq
Mohammed Rafiquzzaman
Ismael Rafols
James Raftery
Laurel Raftery
Rosanne Raftery
Vasilios Raftopoulos
Juan Raga
Mona Ragab
Chikako Ragan
Christina Ragan
Maxime Rageot
Guillaume Ragetly
A. Raggi
Alberto Raggi
Daniela Raggio
Kanwal Raghav
Madhavan Raghavan
Malini Raghavan
Rahul Raghavan
Sridharan Raghavan
Pongali Raghavendra
U. Raghavendra
S. Raghu
Anu Raghunathan
Trivellore Raghunathan
Vijay Krishna Raghunathan
Nandula Raghuram
Kavarthapu Raghuveer
Vikram Raghuwanshi
Ann Ragin
Lembit Rago
Sergio Ragonese
Michael Ragosta
Michael Ragozzino
Erik Ragsdale
Minvydas Ragulskis
Padmashri Ragunathan
Sandeep Raha
Michal Rahat
Eyal Rahav
Nuh Rahbari
Frederic Rahbari-Oskoui
Javad Rahebi

Chantal Raherison
Abdolrasol Rahimi
Hamid Reza Rahimi
Nader Rahimi
Andaleeb Rahman
Fazlur Rahman
Irfan Rahman
Mahmudur Rahman
Mohammad Rahman
Mohammad Saifur Rahman
Muhammad Aziz Rahman
Ruman Rahman
Sajjad Rahman
Suhaila Rahman
Zia Rahman
Abdel Rahmani
Ramin Rahmani
Elham Rahme
Arman Rahmim
Homer Rahnejat
Mati Rahu
Iyad Rahwan
Niraj Rai
Padmalatha Rai
Priyamvada Rai
Tatemitsu Rai
Vivek Rai
David Raible
Florian Raible
James Raich
Rosa Raich
Manfred Raida
Arwa Raies
Julia Raifman
Adalbert Raimann
Kaitlin Raimi
Lavinia Raimondi
Peter Raimondi
Pascal Rainard
Richard Rainbow
Adrian Raine
Tim Raine
Charlis Raineki
Alberto Rainer
Peter Rainer
Innocenzo Rainero
Petrie M. Rainey
George Rainger
Ana Rainho
Thomas Rainwater
Soroush Rais-Bahrami
Louisa Raisbek
Masoud Rais-Rohani
Craig J. Raiton
Anil Raj
Nitin Raj
Srikumar Raja
Yusuf Rajabally
Mehdi Rajabi
Anjali Rajadhyaksha
Raman Rajagopal
Sudarshan Rajagopal
Bhaskaran Rajagopaplan
A. Rajakumar
Satish Rajamani
Prashant Rajan
Ramesh Rajan
Sankaranarayani Rajangam
Raoni Rajão
Indika Rajapakse
Senaka Rajapakse
Thilini Rajapakse
Murugesan Rajaram
Satwik Rajaram
Namakkal-Soorappan Rajasekaran
Parthiban Rajasekaran
R. Rajasekaran
S. Rajasekaran
Arumugam Rajavelu
Yogendra Rajawat
D. Rajdl
Joseph Rajendran
K.V. Rajendran
Karthika Rajendran
Priya Rajendran
S. Rajesh
Mukaila Raji
Swaraj Rajkhowa
Andreja Rajkovic
K. Rajkumar
Ravi Rajkumar
Edwin Rajotte
Sandeep Kumar Rajput
C.S.K. Raju
Kalaivani Raju
Agnieszka Rak
Janusz Rak
Stefan Rakete
Gábor Rákhely
Ehsan Rakhshani
David Rakison
T.J. Rakitan

N. Rakkaya
Mark Rakobowchuk
P. Elizabeth Rakoczy
Tebogo Rakola
Don Rakow
Jurij Rakun
Jean-Philippe Ral
Daniel Raleigh
Glenn Rall
Charalampos Rallis
Stephen Ralph
Mark E. Ralston
Sanjay Ram
Nagaraja Reddy Rama Reddy
Anup Ramachandran
Geetha Ramachandran
Girish Ramachandran
Ilangovan Ramachandran
Prakash Ramachandran
Raja Ramachandran
Ranjani Ramachandran
Ravishankar Ramachandran
Shyam Ramachandran
Srinivasan Ramachandran
Surya Ramachandran
Vinayagam Ramachandran
Naify Ramada
Swetha Ramadesikan
Bram Ramaekers
Johannes Ramaekers
Sreeram Ramagopalan
Jip Ramakers
Chandran Ramakrishna
Suresh Ramakrishna
Girija Ramakrishnan
Lakshmy Ramakrishnan
Naren Ramakrishnan
Ramesh Ramakrishnan
Siddharth Ramakrishnan
Ana Ramalhinho
João Ramalho-Santos
J. Ramalingam
Latha Ramalingam
Satish Ramalingam
Sivaprakash Ramalingam
Suresh Ramalingam
Murali Ramamoorthi
Mahesh Ramamoorthy
Prabhu Ramamoorthy
Sammanda Ramamoorthy
Baranidharan Raman
Chander Raman
Rajiva Raman
Venkata Ramana
Athimalaipet Ramanan
Nagasundara Ramanan
Narendrakumar Ramanan
Rajeshwari Ramanan
Arvind Ramanathan
Murali Ramanathan
Rangasamy Ramanathan
R. Geetha Ramani
Sasirekha Ramani
Georgia Ramantani
Manojkumar RamanUnniNair
Jayachandra Ramapuram
Thiruvarangan Ramaraj
Ranjith Ramasamy
Aravindh Babu Ramasamy Parthiban
José Ramasco
Narayanan Ramasubbu
Aparna Ramasubramanian
Girish Ramaswamy
Megha Ramaswamy
Rohit Ramaswamy
Vijay Ramaswamy
T. Ramayah
Serge Rambal
Alessandro Rambaldi
Rajeev Ramchandran
Nirala Ramchiary
Sahienshadebie Ramdas
Sara Ramella
Jessica Ramella-Roman
Rajkumar Ramesar
Aramandla Ramesh
Arati Ramesh
Ganesan Ramesh
Kirti Ramesh
Samiraj Ramesh
Mika Rämet
Andrew Ramey
Steven Ramey
Fahimeh Ramezani Tehrani
Analiza Ramirez
Dario C. Ramirez
Gerardo Ramirez
Gilbert Ramirez
Hugo Ramirez
Ivan Ramirez
Joel Ramirez
Jose Ramirez

Juan David Ramirez
Mario Ramirez
Marizen Ramirez
Pablo Ramirez
Rafael Ramirez
Raul Ramirez
Rolando (J.J.) Ramirez
Sara Ramirez
María Soledad Ramírez
Noelia Ramírez
Santiago Ramirez-Barahona
Nadiezhda Ramirez-Cabral
Antonio Ramirez-Calvo
Hugo Ramirez-Saad
Jose Ramirez-Valiente
Sofia Ramiro
Rebeca Ramis
Ignacio Ramis Conde
Jacqueline Ramke
Doraiswami Ramkrishna
Norlisah Ramli
Steven Ramm
Indar Ramnarine
Vundli Ramokolo
Eva Ramon
Guy Ramon
Jan Ramon
Santiago Ramón y Cajal
Roberta Ramonda
Aline Ramond-Roquin
Alberto Javier Ramos
Alessandro Ramos
Alexandre Ramos
E.M. Ramos
Fernando Ramos
José Ramos
Jose M. Ramos
Mariana Ramos
Sonia Ramos
Mikael Ramos-Casals
Maria Letícia Ramos-Jorge
Erivan Ramos-Junior
Angel Ramos-Ligonio
Tania Ramos-Moreno
Sebastian Ramos-Onsins
Karam Ramotar
Simone Rampelli
Ermanno Rampinini
Giordano Rampioni
M. Rampling
Luca Rampoldi
Vanu Ramprasath
Satish Ramraj
Satish Kumar Ramraj
Andreja Ramšak
James Ramsay
Josh Ramsay
Rona Ramsay
A. Ramsdell
Ian Ramsey
John Ramsey
Jon Ramsey
Kathryn Ramsey
Laura Ramsey
Vijayalakshmi Ramshankar
Pramod Ramteke
Franck Ramus
Chongzhao Ran
Sophia Ran
Yong Ran
Aadia Rana
Jamal Rana
Kunjan Rana
Neha Rana
P. Rana
Sarosh Rana
Giancarlo Ranalli
Shabbar Ranapurwala
Krista Ranby
Terry Rand
Cameron Randall
Jason Randall
Matthew Randall
Jennifer Randerath
Winfried Randerath
Haseeb Randhawa
Adriane Randolph
Gregory Randolph
Julien Randon-Furling
Lisa Ranford-Cartwright
Parvathi Ranganathan
Sri Ranganathan
Sudarshan Ranganathan
Sampathkumar Rangasamy
Ryan Range
Elizabeth Rangel
Juliana Rangel
Mauricio Rangel-Gomez
Erik Ranheim
Anette Ranhoff
Mary Esther Rani
P. Rani

Girolamo Ranieri
Aashish Ranjan
Akash Ranjan
Ganguly Ranjan
Priya Ranjan
Nalini Ranjit
Sarbin Ranjitkar
Roger Rank
Catharine Rankin
Kenneth Rankin
Brigitte Ranque
Stephane Ranque
Erik Ranschaert
Christopher Ransom
Jason Ransom
Tami Ransom
Merja Rantakokko
K. Rantanen
Barbara Rantner
Kalliopi Rantsiou
Marco Ranucci
Mariagrazia Ranzini
Ajaykumar Rao
Anirudh Rao
Aparna Rao
Arvind Rao
Basuthkar Rao
Bindumadhava Hanumantha Rao
Chinthalapally Rao
Christopher Rao
D. Rao
Desirazu Rao
Ganesh Rao
Geeta Rao
Guillaume Rao
Jianyu Rao
Jingping Rao
K. Rao
K.S. Rao
K.S. Jagannatha Rao
Krishna Rao
Martin Rao
Mrinalini Rao
Mukund Rao
P. S. Shantanu Rao
Panduranga Rao
Pulivarthi Rao
Raj Rao
Ram Rao
Raveendra K. Rao
Rema Rao
Sambasiva Rao
Sridhar Rao
Timsi Rao
Veena Rao
Venigalla Rao
Vivek Rao
Weixiong Rao
Xiancai Rao
Xiangjun Rao
Xiang-Jun Rao
Zhiguo Rao
Afshin Raouf
Anna Rapacz-Leonard
Marta Rapado-Castro
Mark Rapaport
Dan Raper
David Raper
Jayne Raper
Brian Raphael
Christopher Raphael
Martin Raphael
William Raphael
Bruno Rapidel
David Rapoport
Micha Rapoport
Alexander Rapp
Edward Rapp
Francesca Rappa
Jay Rappaport
Wouter-Jan Rappel
Chad Rappleye
Giovanna Rappocciolo
Rino Rappuoli
Kiran Rasal
Karen Rascati
Madeline Rasche
Volker Rasche
Wolfgang Rascher
Ericka Rascon
Serena Rasconi
Nicolas Rascovan
Jason Rasgon
Carla Rash
Awais Rasheed
Muneera Rasheed
M.K. Rasheeda
Armin Rashidi
Mahnaz Rashidi
Mohammad Mehdi Rashidi
Aaron Rashotte
Gordana Rasic

Girish Rasineni
Mathias Rask-Andersen
Charlotte Rasmussen
Dennis Rasmussen
Finn Rasmussen
Heather Rasmussen
Katie Rasmussen
Morten Rasmussen
Susan Rasmussen
Theodore Rasmussen
Eva Rasmussen-Barr
Laura Rasmussen-Torvik
Randall Rasmusson
Aida Hanum Rasool
Tienush Rassaf
Francois Rassendren
Minoo Rassoulzadegan
Jens Rassweiler
Phillipe Rast
Gurdeep Rastogi
Giulia Rastrelli
Anna Ratajska
Rajiv R. Ratan
Laurene Ratcliffe
Norman Ratcliffe
Katja Rateitschak
Dominik Rath
Joseph Rath
N. Rath
Nigam Rath
Subha Rath
Timo Rath
Bruce Rathgeber
Anandharajan Rathinasabapathy
Rahul Rathod
Sujit Rathod
Marie-Helene Ratinaud
Felix Ratjen
Aivaras Ratkevicius
Colin Ratledge
John Ratliff
Miriam Ratliff
Milind Ratnaparkhe
Rinki Ratnapriya
Sujeevan Ratnasingham
David Ratner
Jonah Ratsimbazafy
Woraphat Ratta-apha
Frank Rattay
Niels Rattenborg
Antonia Ratti
Claudio Ratti
Ryan Ratts
Paul Ratz
Franz Ratzinger
Domenico Rau
Kristofer Rau
Angela Raucci
Birgit Rauchbauer
Markus Rauchenzauner
Marjatta Raudaskoski
Florian Raudies
Antonio Raudino
Terje Raudsepp
Patrick Raue
Thomas Rauen
Amy Rauer
Michael Rauh
Arvi Rauk
Jean Loup Rault
Navin Rauniyar
Michael Raupach
Jason Rausch
Josef Rauschecker
Alexander Rauscher
Emily Rauschert
Estrella Rausell
Karsten Rauss
Silke Rautenschlein
Pierre-Emmanuel Rautou
Saaeha Rauz
Amit Raval
Mihir Raval
Maryam Ravan
Lara Ravanetti
Paula Ravasco
Susanna Ravassa
Vineesh Raveendran
Jacques Ravel
Andrea Ravelli
Anita Ravelli
Flavia Ravelli
Erika Raven
John Raven
Alison Ravenscraft
Neil Ravenscroft
Yazhini Ravi
Magalie Ravier
Andrea Ravignani
Gudasalamani Ravikanth
Sowmya Ravikumar
Rahul Ravilla

M.H. Ravindranath
Vijayalakshmi Ravindranath
Raffaella Ravinetto
Giuseppe Raviola
Kundapura Ravishankar
Alfredo Ravizza
Sabine Ravnskov
Salman Rawaf
Lal Rawal
Nidhi Rawat
Siddhartha Rawat
Caroline Rawdon
Ann Rawkins
Nic Rawlence
William Rawlinson
Alan Rawls
Araz Rawshani
Eric Rawson
Patrick Rawstorne
Manjusha Rawtiya
Anandasankar Ray
Ann Ray
Debjit Ray
Deepak Ray
Patricio Ray
Pierre Ray
Prabir Ray
Pradipta Ray
Pritha Ray
Russell Ray
Stuart Ray
Sumit Ray
Supriyo Ray
Swayamjit Ray
Ena Ray Banerjee
Kallol Ray Chaudhuri
Helen Raybould
Pradip Raychaudhruri
Siba Raychaudhuri
Abhik Ray-Chaudhury
Abhik Ray-Chaundhury
Emily Rayfield
Nakul Raykar
Mark Rayment
Jason Raymond
Josette Raymond
Caroline Raynal
Helene Raynal
Hugh Rayner
Julian Rayner
Simon Rayner
Yevgeniy Raynes
Martha Raynolds
Annette Raynor
Peter Raynor
Avraham Raz
Itamar Raz
Sarah Raz
Haider Raza
Shahzad Raza
Shan-e-Ahmed Raza
Wameq Raza
Sylvain Razafimandimbison
Onja Razafindratsima
Babak Razani
Shahnaz Razavi
Husna Razee
Mohsen Razeghi
Ahmed Razek
Orly Razgour
Ehud Razin
Sergey Razin
Raymund Razonable
Olga Razorenova
Edoardo Razzetti
Maria Razzoli
Irene Rea
Andrew Read
Emily Read
Jenny Read
Jordan Read
Laurie Read
Scott Read
Tyffen Read
John Reager
Eva Real
Lara Reale
Ana Rebane
Amanda Rebar
Trudy Rebbeck
Kevin Rebe
Vito Rebecca
Mario Rebecchi
Mayvis Rebeira
Peter Rebeiro
Ana Rebelo
Irene Rebelo
Eleanora Reber
Stefan Reber
Robert Rebhun
Terri Rebmann
Matejka Rebolj

Eria Rebollar
Alberto Rebonato
Julien Reboud
David Reboutier
Sylvie Rebuffat
Kilic Recai
Fabio Recchia
Maria Cristina Recchioni
Dino Rech
Wolfgang Recheis
Lawrence Recht
Les Recio
Luciana Reck Remonti
Ana Recober
Sherief Reda
Richard Redak
Sylvio Redanz
Lavanya Reddivari
Hudson Reddon
Arubala Reddy
Cherkupallly Reddy
Elizabeth Reddy
Harikishore Reddy
Janardan K. Reddy
Madhu Reddy
Marpadga Reddy
Narsa Reddy
Pavankumar N.G. Reddy
Ramalinga Reddy
Sanjay Reddy
Sekhar Reddy
Sravana Reddy
Sudarshan Reddy
Sushma Reddy
Umesh Reddy
Vasudevi Reddy
Vishwanatha Reddy
R. Redfield
Thomas Redick
Christoph Redies
Raymond Redline
Joy Redman
Leanne Redman
T. Michael Redmond
Tony Redmond
Lara Redolfi De Zan
Todd Redpath
Simon Redwood
Bethany Reeb-Sutherland
Brian Reed
Charlotte Reed
Damon Reed
J. Reed
Jason Reed
John Reed
Kent Reed
May Reed
Michael Reed
Phil Reed
Randall Reed
Robert Reed
Robyn Reed
Sarah Reed
Brian Reed Silliman
Glenn Reeder
Brian Reedy
Jill Reedy
F. Reen
Brad Rees
Eliot Rees
Megan A. Rees
Adam Reese
Benjamin Reese
Peter Reese
Torsten Reese
Koen Reesink
Robert Reeve
Sarah Reeve
Adam Reeves
Barnaby Reeves
Mari Reeves
Matthew Reeves
Neil Reeves
R. Keith Reeves
Rachel Reeves
Roger Reeves
Scott Reeves
Thomas Reeves
W. Brian Reeves
Myriam Reffay
Roberto Refinetti
Tarik Regad
Daniel Regan
Elizabeth Regan
Cheryl Regehr
Ivonne Regel
Mor Regev
Francesca Reggiani
Thomas Register
Binod Regmi
Jacques Regnard
T. Regnier

A. Rego
Arménio Rego
Eduardo Rego
Hesper Rego
Giuseppe Regolisti
Adrian Regos
Ruben Regterschot
Noemí Reguart
Wail Rehan
Marit Rehavi
Sebastian Rehberg
Barbara Rehermann
David Rehkopf
Thomas Rehle
Jurgen Rehm
Markus Rehm
Ateequr Rehman
Atteeq Rehman
Junaid Rehman
Muhammad Ishaq Asif Rehmani
Holger Rehmann
Lars Rehmann
Johan H. C. Reiber
Thomas Reiberger
Adam Reich
Marlis Reich
Juergen Reichardt
Janine Reiche
Jennifer Reichek
Carmela Reichel
Ronald Reichel
Amy Reichelt
Andreas Reichenbach
Michael Reichenheim
David Reichert
Johanna Reichert
Sophie Reichert
Melvin Reichman
Florian Reichmann
Jonathan Reichner
Brian Reichow
Natalia Reich-Stiebert
Adam Reid
Alice Reid
Cary Reid
Chris Reid
Glen Reid
Graham Reid
Ian Reid
Kate Reid
Liz Reid
Mary Reid
Michael Reid
Tim Reid
Yvonne Reid
Matthew Reidenbach
Jochen Reif
Lennart Reifels
Jason Reifler
Olav Reikeras
James Reilly
Kathleen Reilly
Peter Reilly
Sean Reilly
Robert Reiman
Hendrik Reimann
Christina Reimer
James Reimer
Raylene Reimer
Inonge Reimert
Joshua Rein
Robert Rein
Theo Rein
Richard Reina
Raul Reina Vaillo
Peter Reinach
Ester Reina-Romo
Silke Reinartz
Sophia Reindl
Annette Reineke
Andreas Reiner
Angelika Reiner
Gerald Reiner
Peter Reiner
Thomas Reinert
John Reinfelder
Matthias Reinhard
Robert Reinhard
Christoph Reinhardt
Jan Reinhardt
Keith Reinhardt
Richard Reinhardt
Walter Reinhardt
Vladimir Reinharz
Claudia Reinheimer
Finn Reinholt
Ulrich Reininghaus
Milan Reinis
Hans Reinke
Lester Reinke
Stacey Reinke
Dan Reinking

Andrew Reinmann
Luis Reino
Rose Reins
Sebastian Reinstadler
Douglas Reintgen
Celso Reis
Flavio Reis
Harry Reis
Joice Reis
Ricardo Reis
Rosana Reis
Tânia Reis
Markus Reischl
Carolina Reisenman
Jakob Reiser
Jochen Reiser
Dominic Reisig
Alexander Reisinger
Kerstin Reisinger
Nina Reislev
Benjamin Reisman
David Reisman
Andreas Reisner
Martin Reiss
Daniel Reißmann
Frank Reister
Austin Reiter
Lawrence Reiter
Russel Reiter
Theresa Reiter
Marc Reitman
Eric Reits
Laurie Reitsema
Adam Reitzel
Katarzyna Rejniak
Jose del Carmen Rejon-Orantes
Romdhane Rekaya
Melinda Rekdahl
Islem Rekik
Mohamed Rela
Rubén Rellán Álvarez
Mary Relling
Ryan Rellingson
Alan Remaley
Anthony Remaud
Han Remaut
Xavier Remesar
Fernando Remião
Gilbert Remillard
David Remington
Michelle Remme
Jonathan Remo
Daniel Remondini
Aude Remot
David Rempel
Polychronis Rempoulakis
James Remsen
Aline Remus
Andrea Remuzzi
Carolyn Ren
Chuanli Ren
Dacheng Ren
Decheng Ren
Dongtao Ren
Fei Ren
Feng Ren
Gang Ren
Jian Ren
Jianfeng Ren
Jiangong Ren
Jiaoyan Ren
Jie Ren
Jun Ren
Lei Ren
Ming-Xun Ren
Monica Ren
Pei-Gen Ren
Pengyu Ren
Ren Ren
Shancheng Ren
Shang Ren
Shiyan Ren
Shuxin Ren
Tianheng Ren
Xiobao Ren
Yan-Fang Ren
Yi Ren
Yijin Ren
Yin Ren
Yingxue Ren
Yizhi Ren
Yuhong Ren
Zhuoming Ren
Zong-Xin Ren
Bernhard Renard
Delphine Renard
Jean-Marc Renaud
Yves Renaudineau
Marie-Ange Renault
Fanni Rencz
Alan Rendall
Luke Rendell

Marc Rendell
Elizabeth Rendina
Michael Rendl
Erika Rendon
Willem Renema
Katy Renfro
Lindsay Renfro
Aravind Rengan
Aravind Kumar Rengan
Deivendran Rengaraj
Zed Rengel
Gianluigi Reni
Alessandra Renieri
Frank Renkewitz
Ortwin Renn
Thomas Renne
Scott Renneckar
Jennifer Rennels
Ellen Renner
Ian Renner
Karl-Heinz Renner
Lars Renner
Lorna Renner
Regina-Maria Renner
Robert Rennert
Phil Reno
Benjamin Renoust
Benjamin Renquist
Jenna Renqvist
Susan Rensburg
M. Rensel
Ronald Rensink
Erik Renstrom
Cyrill Rentsch
Stacey Rentschler
Julie Renwick
Elizabeth Repasky
Yohann Repesse
Claudia Repetto
Rodolfo Repetto
Maria Repolles
Grega Repovs
Jesus Requena
Haluk Resat
Bernhard Resch
Michael Resch
Leslie Rescorla
Angela Resende
Renan Resende
Rodrigo Resende
Yakir Reshef
Salvador Resino
Andreas Reske
Lynn Resler
Daniel Resnick
David Resnik
Joshua Rest
Diego Restrepo
Antonietta Restuccia
Joseph Restuccia
Marc Restuccia
Lilia Retegui
Oliver Rettig
Mallik Rettiganti
Ruben Retuerto
Jan Reubens
Min Reuchamps
Bradley Reuhs
Philipp Reuken
Daniel Reuman
Sigrun Reumann
Emma Reungoat
Victor Reus
Thorsten Reusch
Stefan Reuscher
Cezane Reuter
Nina Reuter
Peter Reuter
Sebastian Reuter
Boris Reva
Kelly Reveles
Jefferson Revell
Peter Revell
Massimo Reverberi
Frederique Reverchon
E.J. Reverri
Tamas Revesz
Sergei Revskoy
Boris Rewald
Eric Rexstad
Antonio Rey
Daniel Rey
Federico Rey
Olivier Rey
Pedro Rey-Biel
Henry Reyer
Enrique Reyes
Jinnethe Reyes
Julian Reyes
Luis Reyes
Victor Reyes
Rodrigo Reyes Lamothe

Hugo Reyes-Centeno
Elsa Reyes-Reyes
Paul Reyfman
Gabriel Reygondeau
Louise Reynard
Anna Reyners
Jean-Marc Reynes
Albert Reynolds
C. Lockwood Reynolds
Clare Reynolds
Harmony Reynolds
Joshua Reynolds
Julie Reynolds
Nick Reynolds
Olivia Reynolds
Richard Reynolds
Simone Reynolds
Stacey Reynolds
Susan Reynolds
Tim Reynolds
Roxana Reynoso
Gilberto Reynoso-Meza
Ana Rey-Rico
Maryam Reza
Fariba Rezaee
Ramin Rezaee
Negar Rezaei
Yousef Rezaei
Mostafa Rezaei-Tavirani
Zeljko Rezek
Flavia Rezende
Paulo Rezende
David Reznick
Sandra Reznik
Koon Ho Rha
Sarah Rhea
Jinnie Rhee
Soo Rhee
Joshua Rhein
Maikel Rheinstadter
James Rheinwald
Mijke Rhemtulla
Turk Rhen
Nick Rhind
Gyu-Jin Rho
Mina Rho
Seong-Hwan Rho
Paul Rhoades
Daniel Rhoads
Jon Marc Rhoads
Dale Rhoda
Cody Rhoden
Adelaide Rhodes
Daniela Rhodes
Jonathan Rhodes
Bethany Rhoten
Andrew Rhyne
Moussa Albert Riachy
Dario Riascos-Bernal
Andri Riau
Musarrat Riaz
Cecilia Ribalaygua
Juan Ribas
Laia Ribas
Vinicius Ribas
Margarida Ribau Teixeira
Amanda Ribeiro
Ana Ribeiro
Ana Isabel Ribeiro
Ana Paula Ribeiro
Antonio Ribeiro
António Ribeiro
Bergmann Ribeiro
Claudia Ribeiro
Claudio Tadeu Daniel Ribeiro
Danilo Ribeiro
Dimas Ribeiro
Filomena Ribeiro
Haroldo Ribeiro
José Luís Pais Ribeiro
Laura Ribeiro
M.G. Ribeiro
Márcia Ribeiro
Ricardo Ribeiro
Ruy Ribeiro
Servio Ribeiro
Bruna Ribeiro de Andrade Ramos
Anderson Ribeiro-Carvalho
Salma Ribeiz
Christophe Ribelayga
Angeles Ribera
Simone Ribero
Werner Ribitsch
Daniel Ricard
Jean-Damien Ricard
Mark Ricard
Ana Ricardo
Carlos André Ornelas Ricart
Ezio Ricca
Mark Ricca
Carlo Riccardi
Fulvio Ricceri

Matteo Ricchi
Alessandro Ricci
Alex Ricci
Claudia Ricci
Davide Ricci
Irene Ricci
Zaccaria Ricci
Carmela Ricciardelli
Emiliano Ricciardi
Enzo Ricciardi
Robert Ricciardi
Fabrio Ricciardolo
Andrea Riccio
Jean Ricco
C. Rice
Danielle Rice
David Rice
Dennis Rice
Heather Rice
Ian Rice
Kelly Rice
Laura Rice
Lyndi Rice
Margaret Rice
Robert Rice
Scott Rice
Simon Rice
Terri Rice
Todd Rice
Alisa Rich
Alison Rich
Jeremy Rich
Megan Rich
Thomas Rich
Aline Richard
Bruce Richard
Denis Richard
Florence Richard
Francois Richard
Guy-Franck Richard
Hillary Richard
Isabelle Richard
John Richard
Vincent Richard
Florence Richard-Forget
Adam Richards
David Richards
Jim Richards
JoAnne Richards
Katrina Richards
Mark Richards
Martin Richards
Rickelle Richards
S. Richards
Stephen Richards
Todd Richards
William Richards
Astrid Richardsen
Rebecca Richards-Kortum
David Richardson
Gerry Richardson
Jason Richardson
Laurie Richardson
Leif Richardson
Mark Richardson
Philip Richardson
Rodney Richardson
Seth Richards-Shubik
Amanda Richdale
Philippe Richebe
Jennifer Richer
Martin Richer
Peter Richerson
John Richey
Katie Richgels
Jerome Richie
Lauren Richmond
Rebecca Richmond
Juergen Richt
Chesney Richter
Claus-Peter Richter
Franziska Richter
Hans Richter
Jessika Luth Richter
Joel Richter
Juliane Richter
Katharina Richter
Klaus Richter
Lars Richter
Ronny Richter
Sandra Richter
Sara Richter
Tobias Richter
Joan Richtsmeier
Alexander Rickard
Caroline Rickards
Thomas Ricketts
Daniel Ricklin
C.W. Rico
Leonardo Ricotti
Hugh Riddell
Lynn Riddiford

Bruce Riddle
Nicole Riddle
Ryan Riddle
Sharon Riddler
Aline Rideau Batista Novais
Benjamin Ridenhour
Alexandra Rideout
Jennifer Rider
Lisa Rider
Angela Ridgel
Jennifer Ridgeway
Lisa Ridnour
Erminia Ridolo
Samuel Ridout
Alvaro Ridruejo
Ezequiel Ridruejo
Annelies Rie
Tobias Riede
Bettina Riedel
Thomas Riedel
Walter Riedell
Florian Rieder
Hans Rieder
Michael Rieder
Christopher Riedl
Christine Riedy
Timo Rieg
Heiko Rieger
Sandra Rieger
Kimberly Rieger-Christ
Christina Riehl
Michael Riehle
Michelle Riehle
Tapani Riekki
Leonardo Riella
Angelika Riemer
Bart Rienties
Maria Riera
Jonas Ries
Kristian Riesbeck
John Rieser
Ana Riesgo Gil
Thomas Riess
Scott Riester
Pia Riestra
Jens Rietdorf
Shirley Rietdyk
Arne Rietsch
Ivo Rieu
Jason Rife
Riaan Rifkin
F. Rigamonti
Daniela Rigano
Solange Rigaud
Thierry Rigaud
Alan Rigby
Edward Rigdon
Blake Riggs
Elisha Riggs
Nicoletta Righini
Cynthia Riginos
Arianna Rigon
Jason Rihel
Robert Gabriel Coumine Riis
Monique Rijnkels
Yasuko Rikihisa
Ben Riley
Brien Riley
Christopher Riley
David Riley
Elise Riley
James Riley
Kasandra Riley
Malcolm Riley
Robert Riley
Roger Riley
Kirsi Rilla
A. Rillig
Gerald Rimbach
Rebecca Rimbach
Yves Rimet
Joseph Rimland
Guus Rimmelzwaan
Espen Rimstad
Barbara Rinaldi
Carlos Rinaldi
Serena Rinaldo
Ursula Rinas
Paolo Rinaudo
Dmitry Rinberg
Alexander Rind
Fabio Rindi
Laura Rindi
Claire Rinehart
Timothy Rinehart
Johannes Ring
Dagmar Ringe
Donald Ringe
Richard Ringel
Marc Ringelhan
Tamar Ringel-Kulka
Adrian Ringelstein

Berndt Ringelstein
E. Bernd Ringelstein
Steffen Ringgaard
Steffen Ringhof
Jennifer Ringrose
Robert Ringseis
Ingo Ringshausen
John Rinker
Gordon Rintoul
John Rinzel
Natalia Riobo-Del Galdo
Jorge Rioja
Matteo Riondato
Francisco José Rios
David Rios-Covián
Julien Riou
Ugo Ripamonti
Jonathan Ripp
Steven Ripp
Bengt Rippe
Catarina Rippe
Jürgen Ripperger
M.R. Rippo
Francisco Riquelme
Shannon Risacher
David Risco
Patrizia Rise
Kathryn Risher
William Risher
Patricia Risica
Evan Risko
Kimberly Risma
Theo Rispens
Saara Rissanen
G. Ristagno
Giuseppe Ristagno
Sashko Ristov
Michael Ristow
Viviana Ritacco
Jason Ritchie
Kerry Ritchie
Rebecca Ritchie
Scott Ritchie
Stuart Ritchie
William Ritchie
Tiarney Ritchwood
Nicolas Riteau
Konstantinos Ritis
Erik Ritman
Koert Ritmeijer
Ilana Ritov
Markus Ritter
Laure Rittie
Beate Ritz
Christiane Ritz
Roy Ritzmann
Giuseppe Riva
Casto Rivadulla
David Rival
Florent Rivals
Jillian Rivard
Valeria Rivarola
Carmen Rivas
Jesus Rivas
Monica Rivas Casado
Jean-Pierre Riveline
Debra Rivera
Francisco Rivera
Windell Rivera
Maite Rivera Gorrin
Rafael Rivera-Bustamante
Mónica Rivera-Díaz
Susana Rivera-Mancía
Jesus Rivera-Nieves
Fredy Rivera-Páez
Crisalejandra Rivera-Perez
Francisco Rivero
José Luis Rivero
Adolfo Rivero-Müller
Jacob Riveron
Adam Rivers
Charles Rivers
James Rivers
Emily Rivest
Guillaume Riviere
Jim Riviere
Eleonor Rivindelcampo
Laura Rivino
Carlo Rivolta
Konstantinos Rizas
Raed Rizkallah
Dimitrios Rizos
Sakina Rizvi
Syed Rizvi
Muhammad Rizwan
Kristina Rizzardi
Giuliano Rizzardini
Marta Rizzi
Sandra Rizzi
Caterina Rizzo
Giovanni Rizzo
Giuseppe Rizzo

Manfredi Rizzo
Michael Rizzo
Piervincenzo Rizzo
Gabrielle Rizzuto
Simon Ro
Young Ro
Sergio Roa
Alexandra Roach
Michael Roach
Neil Roach
Vanessa Roach
Filip Rob
Kedir Roba
Bernard Robaire
Juan Robalino
Shannon Robalino
Kelly Roballo
Joana Robalo
John Robb
David Robbe
Suelee Robbe-Austerman
Charles Robbins
Cheryl Robbins
Matthew Robbins
Megan Robbins
Robert Robbins
Gwen Robbins Schug
Bruno Robbs
Michael Robek
Stefanie Robel
Carlos Robello
Elisha Roberson
Erik Roberson
Paula Roberson
Christelle Robert
Eric Robert
Matthieu Robert
Cazzato Roberto
Jessica Roberto
Amity Roberts
Bayard Roberts
Blaine Roberts
Chris Roberts
Claire Roberts
Craig Roberts
Dale Roberts
Derek Roberts
Eric Roberts
Evan Roberts
Gareth Roberts
James M. Roberts
Jason Roberts
Katherine Roberts
Llion Roberts
Lynette Roberts
Marilyn Roberts
Melissa Roberts
Michael Roberts
Philipp Roberts
Rachel Roberts
Robert Roberts
Sam Roberts
Seán Roberts
Stephen Roberts
Tamalee Roberts
Andrew Robertson
Charles Robertson
Claudia Robertson
David Robertson
Deborah Robertson
Eleanor Robertson
Gregory Robertson
Henry Robertson
James Robertson
John Robertson
Kevin Robertson
Lindsay Robertson
Lucy Robertson
Nicola Robertson
Peter Robertson
Robin Robertson
Roy Robertson
Sam Robertson
Tony Robertson
Will Robertson
Stephane Roberty
Michael Robeson
Scott Robeson
Pamela Robey
Alain Robichon
Jacques Robidoux
Julie Robillard
Arif Hasan Khan Robin
Charles Robin
Christophe Robin
Joel Robin
Paul Robin
S. Robin
Eric Robinet
William Robins
Roy Robins-Browne
Anne Robinson

Chris Robinson
Christopher Robinson
Clifford Robinson
Colin Robinson
Cory Robinson
Courtney Robinson
Craig Robinson
Elva Robinson
Emma Robinson
Eric Robinson
Harriet Robinson
Heath Robinson
Hugh Robinson
J. Paul Robinson
Jacob T. Robinson
Jennifer Robinson
Joann Robinson
John Robinson
Lauren Robinson
Meghan Robinson
Melissa Robinson
Michael Robinson
Paul Robinson
Richard Robinson
Robert Robinson
Scott R. Robinson
Stacie Robinson
Victoria Robinson
Nicolas Robinson-Garcia
Kessica Robinson-Papp
Christopher Robison
Matthew Robison
Gerardo Robledo
Patricia Robledo
Juan Robledo-Arnuncio
Juan Jose Robledo-Arnuncio
Pedro Robles
Rafael Robles
Martha Robles-Flores
Alexander Robling
Julia Robotham
Holly Robson
Simon Robson
T. Matthew Robson
John Robst
Dylan Roby
Justin Roby
Katherine Roby
G. Roca
Josep Roca
Maite Roca
Pere Roca-Cusachs
Bianca Rocca
Maria Rocca
Michele Rocca
Aldo Roccaro
Marcella Rocchetti
Camilla Rocchi
Giancarlo Roccuzzo
Marie Roch
A. Rocha
Carla Rocha
Edson Rocha
Eduardo Rocha
Jorge Rocha
Luis Rocha
Maria Sheila Rocha
Paulo Rocha
Ricardo Rocha
Axayácatl Rocha-Olivares
Mary-Aude Rochat
Philipe Rochat
Etel Rocha-Vieira
Ann M. Roche
Daniel Roche
Enrique Roche
Erin Roche
Nicolas Roche
R.C. Roche
Jonathan Rocheleau
Emma Rochelle-Newall
Margarida Rocheta
Jacques Rochette
Charles Rock
Christopher Rock
Jason Rock
Melanie Rock
David Rocke
Emma Rocke
Ian Rockett
Joacim Rocklöv
Beth Rockmill
Maxine Rockoff
Stanley Rockson
Brigitte Rockstroh
Cara Rockwell
Cheryl Rockwell
Patricia Rockwell
Robert Rockwell
Edward Rockwood
Barry Rockx
Claudia Roda

Avital Rodal
Gil Rodas
Helen Rodd
Matthew Rodda
Simone Rodda
Louise Roddam
Thomas Rodebaugh
David Rodeberg
Ulrich Rodeck
Franz Rödel
Mark-Oliver Rödel
Luigi Rodella
Christian Rödelsperger
Richard Roden
Bas Rodenburg
Sumayah Rodenburg-Vandenbussche
Scott Rodeo
Heinrich Roder
Marion Röder
Christoph Roderburg
James A. Rodger
Matthew Rodger
Kathleen Rodgers
Ku'ulei Rodgers
Rachel Rodgers
Victor Rodgers
Zachary Rodgers
Thomas Rodhouse
Stefan Rödiger
Ana Rodiles Guerrero
Steliana Rodino
Dmitry Rodionov
Channarong Rodkhum
Karin Rodland
Riccardo Rodolfo-Metalpa
Phillippe Rodon
Guillermo Rodrigo
Luis Rodrigo
Maria Rodrigo
Sébastien Rodrigue
Alice Rodrigues
Camila Rodrigues
Clara Rodrigues
Fernando Rodrigues
Jean-Marie Rodrigues
Jorge Rodrigues
Juliana Rodrigues
Luis Rodrigues
Maria Elizabeth Rodrigues
Pedro Rodrigues
Rosana Rodrigues
Vitor Rodrigues
Alexander Rodriguez
Amy Rodriguez
Christine Rodriguez
Clara Rodriguez
Cristina Rodriguez
Edgardo Rodriguez
Edward K. Rodriguez
Elisa Rodriguez
Elke Rodriguez
Eunice Rodriguez
Fausto Rodriguez
Fernando Rodriguez
Juan Rodriguez
Juan Carlos Rodriguez
Monica Rodriguez
Natalia Castano Rodriguez
Pedro Rodriguez
Ronald M. Rodriguez
Teresa Rodriguez
Tristan Rodriguez
Airam Rodríguez
Amaia Rodríguez
Ana Rodríguez
Esteban Rodríguez
Ricardo Rodríguez
Jose Alberto Rodriguez Castillo
Ricardo Rodríguez de la Vega
Miguel Rodriguez Garcia
Irene Rodriguez Hernandez
Aroa Rodriguez Iglesias
Heriberto Rodriguez Martinez
Cesar Rodriguez Sanchez
Salvador Rodríguez Zaragoza
Jose Rodriguez-Alvarez
Elisabeth Rodriguez-Bies
Carmen Rodriguez-Blazquez
Enrique Rodriguez-Boulan
Teresa Rodriguez-Calvo
Ignacio Rodriguez-Crespo
Carlos E. Rodriguez-Diaz
Susana Rodríguez-Echeverría
Sara Rodríguez-Enríquez
Juan Rodriguez-Flores
Guadalupe Rodríguez-González
Beatriz Rodriguez-Grande
Nieves Rodríguez-Henche
Bernardo Rodriguez-Iturbe
Ismael Rodriguez-Lara
Juan Rodriguez-Lecompte
Mario Rodríguez-Lopez

Alfonso J. Rodriguez-Morales
Victor Rodríguez-Moreno
Luis J. Rodríguez-Muñiz
Antonio Rodríguez-Núnez
Alexander Rodriguez-Palacios
Francisco Rodriguez-Panadero
Manuel Rodríguez-Perálvarez
P. Rodriguez-Pombo
Federico Rodriguez-Porcel
Martin Rodriguez-Porcel
Diego Rodriguez-Puyol
Tatiana Rodriguez-Reyna
Javier Rodriguez-Rodriguez
Noe Rodriguez-Rodriguez
Fernando Rodríguez-Rodríguez
Alexandro Rodriguez-Rojas
José Domingo Rodríguez-Teijeiro
Juan Rodriguez-Testal
Aída Verónica Rodríguez-Tovar
Alma Rodríguez-Troncoso
Mayela Rodríguez-Violante
Roger Rodriguez-Vivas
Reitze Rodseth
Anna Roe
Brian Roe
Jung-Hye Roe
Kevin Roe
Simon Roe
Oluf Røe
Urte Roeber
Anthony J. Roecker
Ingo Roeder
Mareike Roeder
Ben Roediger
Rainer Roehe
Jobst Roehmel
Eric J. Roeland
Bart Roelands
Mathieu Roelants
Ryan Roemmich
Till Roenneberg
Troy Roepke
Michael Roerecke
Luiz Roesch
Neal Roese
Martin Roessle
Blake Roessler
Philip Roessler
Annelieke Roest
Hendrik Jan Roest
Meta Roestenberg
Francois Roets
Antonella Roetto
Herbert Roeyers
Henrique Rofatto
Ofer Rog
Emily Rogalski
Slavko Rogan
Jana Rogasch
Elizabeth Rogawski
Claire Rogel-Gaillard
Boris Rogelj
Joeri Rogelj
Lars Rogenmoser
Jerome Roger
Thierry Roger
Amino Rogerio
Alan Rogers
Alice Rogers
Barbara Rogers
Buck Rogers
Chris Rogers
George Rogers
Helen L. Rogers
Jeffrey Rogers
Justin Rogers
Lynette Rogers
Maximillian Rogers
Morwenna Rogers
Peter Rogers
Raymond Rogers
Thomas Rogers
Tracey Rogers
Laura Rogers-Bennett
Peter Rogerson
Stephen Rogerson
Robert Roghair
Mehrdad Roghani
Didier Rognan
Ute Rogner
Giulio Rognini
Øivind Rognmo
Moshe Rogosnitzsky
Isabelle Rogowski
Ewelina Rogozinska
Jee Hoon Roh
Jinsook Roh
Jung Roh
Martin Rohacek
Lisa Rohan
Maryam Rohani
Anand Rohatgi

Mohammad Rohban
Kerstin Rohde
Manfred Rohde
Paul Rohde
Gudrun Rohe
Aarti Rohira
Nadin Rohland
Katrin Rohlf
Fabian Rohner
Bärbel Rohrer
Doug Rohrer
Thomas Rohrlack
Oliver Röhrle
Sabine Rohrmann
Remo Rohs
Damaris Rohsenow
Forest Rohwer
Jens Rohwer
Sievert Rohwer
Giulio Sergio Roi
Idan Roifman
Jordi Roig
Marc Roig
Anna Roik
Emmanuel Roilides
Timo Roine
Lassi Roininen
Evgeny Roitberg
David Roiz
Laia Rojano Doñate
Patricio Rojas
Jose Rojas-Caraballo
Jesus Rojo
José Rojo
Leonel E. Rojo
Slawa Rokicki
Darin Rokyta
Damian Roland
Sara Rolandsson
Muradian Roldan
Andreas Rolf
Megan Rolf
Jens Rolff
Alessandro Rolfo
Christian Rolfo
Martin Rolfs
Susanne Rolinski
Shawn Roll
Jess Rollason
Marsha Rolle
Richard Roller
Hardy Rolletschek
Christine Rollier
Judith Rollinger
Asya Rolls
Gary Roloff
William Rom
Tania Romacho
Alessandra Romagnoli
Renato Romagnoli
Stefano Romagnoli
Giovanni Romagnoni
Simon Romaine
Jesus Romalde
Michael Romalis
Brian Roman
Jesse Roman
Michael Roman
Shaun Roman
Rosa Roman Cuesta
Marc Romana
Gianfranco Romanazzi
Alessandra Romanelli
Svetlana Romanenko
Razvan Romanescu
Andrea Romani
Chiara Romani
Roberto Romani
Michael Romann
Andrea Romano
Daniele Romano
Diego Romano
Giovanna Romano
Marco Romano
Mario Romano
Marta Romano
Maurizio Romano
Megan Romano
Simona Romano
Thiago Romano
Marcel Romanos
Victor Romanov
Mateusz Romanowski
Luisa Romao
Jan-Erik Romar
Maria Romay-Barja
Puck Rombach
Cesar Valmor Rombaldi
Simona Rombo
Yoann Rombouts
Jörg Romeis
Stefania Romeo

Eva María Romera
Fabio Romerio
Alejandro Romero
Alvaro Romero
Francisco Romero
Guillermo Romero
Hernan Romero
Ignacio Romero
Laura Romero
Teresa Romero
Yair Romero
Jon Romero-Aguirregomezcorta
Juanita Romero-Diaz
Manuel Romero-Gómez
Ángel Romero-Martínez
Marina Romero-Ramos
Consuelo Romero-Sanchez
Lindsey Romick-Rosendale
Jonathan Romiguier
John Romley
Paulus Rommer
Joost Rommers
Benedetto Romoli
Annette Rompel
MaryAnn Romski
Isobel Ronai
Vittoria Roncalli
Maria Roncero
Andrea Ronchi
Carlos Ronchi
Cristina Ronchi
Chiara Ronchini
Paolo Roncon
Jorge Ronderos
Zeev Ronen
Jun Rong
Shisong Rong
Yi Rong
Luisa Ronga
Rongchun Rongchun
Pornpimol Rongnoparut
Christopher Rongo
Anthony Rongvaux
Antti Ronkainen
Justiina Ronkainen
Tina Rönn
Henrik Ronnberg
Mohammad Roohani
Farzin Roohvand
Nicola Rooney
Rebecca Rooney
Peter Roopnarine
Christian Roos
Christopher Roos
Kenny Roose
Mikeal Roose
Allen Root
Karen Root
Martin Root
Lindsey Root Luna
Cally Roper
David Roper
Katrina Roper
Marcus Roper
Randall Roper
Stephen Roper
Santiago Ropero
Delphine Ropers
Athina Ropodi
Matheus Roque
Anne-Marie Roque-Afonso
Maria Cristina Roque-Barreira
Damian Roqueiro
Antoine Roquilly
Rasmus Rørth
Joaquim Ros
Margarita Ros
Robert Ros
Vera Ros
Adriane Rosa
Marcello Rosa
Maria Rosa
Patricia Rosa
Rui Rosa
Susana C. Rosa
Alfonso Rosa Garcia
José Cesar Rosa Neto
Consolación Rosado
Elizabeth Rosado Balmayor
Carlos Rosales
Jesusa Rosales
María Rosales Statkus
Luís Miguel Rosalino
Gus Rosania
Mario Rosanova
F. Rosario
Gracy Rosario
Ivan Rosas
Paola Rosas
Mauricio Rosas Ballina
Rogelio Rosas-Valdez
Antonio Rosato

Zeev Rosberger
Hamilton Roschel
Paul Roschger
Bernd Roschitzki
Federica Roscioni
Sabine Roscoat
Federico Rosconi
Chad Rose
Charles Rose
David Rose
Debra Rose
Devin Rose
Greg Rose
Jessica Rose
Joan Rose
Kathryn Rose
N. Rose
Noah Rose
Noel Rose
Peter Rose
Shyanika Rose
Stephen Rose
Timothy Rose
Mary Rosedale
Anna Rosell
Leonilde Roselli
Esther Roselló-Lletí
Martin Rosema
Stefanie Rosema
Ira Roseman
Denis Rosemberg
Mario Rosemblatt
Alexander Rosemurgy
Danya Rosen
Jennifer Rosen
Kirill Rosen
Michael Rosen
Paul Rosen
Steven Rosen
Sydney Rosen
Vicki Rosen
Jean Rosenbaum
Alexander Rosenberg
Avi Rosenberg
Matthew Rosenberg
Michael Rosenberg
Molly Rosenberg
Nora Rosenberg
William Rosenberger
Adam Rosenblatt
Paul Rosenblit
Mosi Rosenboim
Jonas Rosendahl
Mikkel Rosendahl
Søren Rosendahl
Jordan Rosenfeld
Michael Rosenfeld
Mark Rosenfield
Rebeca Rosengaus
Patrice Rosengrave
Anders Rosengren
Jessica Rosenholm
Jeremy Rosenkranz
Peter Rosenkranz
Richard Rosenkranz
Signe Rosenlund
Joshua Rosenow
Ilan Rosenshine
Robert Rosenson
Jacob Rosenstein
Tatiana Rosenstock
Tom Rosenström
Benjamin Rosenthal
David Rosenthal
Lawrence Rosenthal
Meagen Rosenthal
Philip Rosenthal
Rita Rosenthal
Sandra Rosenthal
Victor Rosenthal
Steven Rosenzweig
Connie Roser-Renouf
Florencia Rosetti
Roger Ros-Freixedes
Mikhail Roshal
Susanna Rosi
Patrizia Rosignoli
Zuzanna Rosin
Marcel Rosinger
Isabelle Rosinski-Chupin
Roman Rosipal
Laszlo Rosivall
Beverly Roskos
Robert Roskoski
Johanna Rosman
Yossi Rosman
Karli Rosner
Philip Rosoff
John Rosowski
A. Ross
Alonzo Ross
Ann Ross

B. Ross
Bernhard Ross
Beth Ross
Colin Ross
Duncan Ross
James Ross
Jennifer Ross
Levi Ross
Michael Ross
Mike Ross
Nancy Ross
Owen Ross
Ryan Ross
Stephen Ross
Ted Ross
Jan Rossaint
Mateus Rossato
Amy Ross-Davis
Ramon Rossello-Mora
Ramón Rossello-Mora
John Rossen
Lauren Rossen
Andrea Rossetti
Andreacarlo Rossetti
Giulio Rossetti
Manuel Rossetti
Alyssia Rossetto
Cyprian Rossetto
Anna Rita Rossi
Antonio Rossi
Daniele Rossi
Esther Rossi
Ethan Rossi
Federico Rossi
Francesca Rossi
Gian Rossi
Giulio Rossi
Livia Rossi
Luca Rossi
Marcelo Rossi
Marco Rossi
Marvin A. Rossi
Maximo Rossi
René Rossi
Sergio Rossi
Simona Rossi
Sonja Rossi
Valentina Rossi
Ombeline Rossier
Julien Rossignol
Marcos Rossi-Izquierdo
Paolo Rossini
D.G. Rossiter
Michael Rossmann
Moritz Rossner
Steffen Rossner
C. Rosso
Lorenzo Rosso
Manuela Rossol
Rebecca C. Rossom
Theresa Rossouw
Klaus Rostgaard
Vittorio Rosti
Siri Rostoft
Robert Rostomily
Michael Rosu-Myles
Martin Rosvall
Matteo Rota
Neal Rote
Susan Rotenberg
Bradley J. Roth
Elliot Roth
Eric Roth
Holger Roth
Jeffrey Roth
Joachim Roth
Melissa Roth
Michael Roth
Olivia Roth
Patrick Roth
Tania Roth
Tobias Roth
Zvi Roth
Ulrike Rothe
Martina Rothenbühler
Gernot Rother
Kristina Rother
Kathrin Rothermich
Till Rothig
Kai Rothkamm
Holger Rothkegel
Paul Rothmore
Joseph Rothnagel
Bruce Rothschild
David Rothschild
Lily Rothschild
Daniela Rotin
Roberto Rotini
Oren Rotman
Daniele Rotolo
Dino Rotondo
Veerle Rots

David Rotsch
Anand Rotte
Shlomo Rottem
Jerome Rotter
Victoria Rotter Sopasakis
Stephen Rottgers
Wouter Rottier
Paola Rottoli
Giulia Rotundo
Jean-Max Rouanet
David Roubik
Eric Roubos
Philippe Rouch
Aymeric Rouchaud
Xavier Roucou
François Rouet
Rodolphe Rougerie
Guillermo Rougier
Gholamreza Rouhi
Yves Rouillé
Dimitrios Roukos
Jean-Francois Roulet
Lucien Roulet
Anne-Lyse Roulin Ducrest
Carli Roulston
Frans Roumen
Sharon Rounds
Paul Roundy
Christina Roup
Nuria Roura-Pascual
Barry Rouse
George Roush
Richard Roush
Kathrin Rousk
Konstantinos Rouskas
Guy Rousseau
Karine Rousseau
Ronald Rousseau
Sophie Rousseaux
Nathalie Roussel
Jonathan Roussey
Matthieau Roustit
Matthieu Roustit
Michael Rout
Micheal Rout
Namita Rout
Andrew Routh
Jonathan Routh
Juha Rouvinen
Jean-Christophe Roux
Julien Roux
Michel Roux
Pierre-François Roux
Saartjie Roux
Pascale Roux-Lombard
François Rouyer
Anne Rovelet-Lecrux
Alessio Rovere
Francesco Rovero
Giandomenico Roviello
Meritxell Rovira
Jeff Row
Andrew Rowan
Sarah Rowan
Yorke Rowan
David Rowat
Ashley Rowden
Christopher Rowe
Edwin Rowe
Glenn Rowe
Richard Rowe
Susan Rowe
Tawandra Rowell-Cunsolo
Pedram Rowhani
David Rowland
Mark Rowland
Alex Rowlands
Christopher Rowlands
Roger Rowlett
Anne Rowley
Christopher Rowley
James Rowley
Fábio Roxo
Abhrajeet Roy
Ambrish Roy
Arpita Roy
Avijit Roy
Badal Roy
Bimal Roy
Indrajit Roy
Jane Roy
Joy Roy
Laurence Roy
Monique Roy
Nilotpal Roy
Partha Roy
Pierre-Marie Roy
Rajendra Roy
Sabita Roy
Sankanika Roy
Shouraseni Roy
Snehashis Roy

Somnath Roy
Sushmita Roy
Syamal Roy
Rimjhim Roy Choudhury
Donald Royall
Mark Roycik
Astrid Roy-Engel
Stephen Royle
Jose Luis Royo
Johannes Royset
Sanya Roysommuti
Sabine Roza
Ignacio Rozada
Vianney Rozand
Dmitri Rozanov
Malgorzata Rózanowska
David Rozansky
Emmanuel Roze
Jean-Christophe Roze
Scott Rozelle
Jos Rozema
Daniel Rozen
Patrick Rozenberg
Shai Rozenberg
Danaë Rozendaal
Enrique Rozengurt
Paul Rozin
Jan Rozman
Sharon Rozovsky
Adam Rozumalski
Henry Rozycki
Laurentiu Rozylowicz
Bowen Ruan
Daniel Ruan
Haowen Ruan
Lifang Ruan
Songlin Ruan
Zhenhua Ruan
Sara Ruane
Matthew Ruark
Patricia Ruas-Madiedo
Martial Ruat
Abdur Rub
Paola Rubbioni
Andrea Rubboli
Leonid Rubchinsky
Julian Rubel
Mollie Ruben
Rui Ruben
Jonas Rubenson
Daniel Rubenstein
Eric Rubenstein
Leonard Rubenstein
Peter Rubenstein
Nicolas Rubido
Brian Rubin
Bruce Rubin
Clinton Rubin
Eitan Rubin
Janet Rubin
Jeffrey Rubin
Joshua Rubin
Lewis Rubin
Marcie S. Rubin
Sebastien Rubin
Shimshon Rubin
Alessandro Rubinacci
Clara Rubincam
Dvora Rubinger
Mauro Rubini
Mikail Rubinov
Clifford Rubinstein
Jack Rubinstein
Belén Rubio
Luis Rubio
Luis Ángel Rubio
María Rubio
Xavier Rubio-Campillo
Paula Rubio-Fernandez
Patrizia Rubiolo
P.P. Rubis
Diego Rubolini
Nikolai Rubtsov
Antoni Rucinski
Robert Rucker
Gerta Rücker
Julia Rucklidge
Ireneusz Ruczynski
Jana Ruda
Donna Rudd
Robert Rudd
Nancy Ruddle
Richard Ruddy
Lawrence Rudel
Douglas Ruden
Sasha Rudenstine
Neil Ruderman
Knut Rudi
R. Daniel Rudic
Assaf Rudich
Seitz Rüdiger
Stephen Rudin

Mats Rudling
Laurie Rudman
David Rudnick
Michael Rudnicki
Kara Rudolph
A.K.M. Ashiqul Haqze Rudra
Sairam Rudrabhatla
Jerry Rudy
Meghan Ruebel
Oliver Ruebel
M. Rosario Rueda
Laura Milena Rueda Delgado
Manuel Ruedi
Maria Adele Rueger
Simon Ruegg
Urs Ruegg
Paul Ruegger
Julie Ruel-Bergeron
Eric Ruelland
Antonio Ruellas
Jennifer Ruesink
L. Bruno Ruest
Maja Ruetten
Klaus Ruetzler
Kurt Ruetzler
Alessandra Rufa
Katharina Rufener
Ronald Ruff
Jay Ruffell
Giulio Ruffini
Heinz Ruffner
Ana Rufino
María Rugeles
Rosaria M. Ruggeri
Michael Ruggieri, Sr.
Paolo Ruggiero
Dumitrita Rugina
Zachary Ruhe
Constance Ruhl
Heiko Rühl
Ralph Rühl
Martina Ruhmland
Isabelle Ruhnke
Junpeng Rui
Rong Rui
Tao Rui
Xianliang Rui
Xue Rui
Ynte Ruigrok
L.M. Ruilope
Matthijs Ruiter
C.A. Ruiz
Carlos Ruiz
Federico Ruiz
Ignacio Ruiz
John Ruiz
Manuel Ruiz
Milagros Ruiz
Natividad Ruiz
Oscar Ruiz
Sofía Ruiz de Gauna
Maria Jose Ruiz Martos
Jesus Ruiz-Cabello
Ana Ruiz-Casado
Mónica Ruiz-Casares
Javier Ruiz-Castillo
Francisco Ruiz-Dueñas
Jose Francisco Ruiz-Fons
Mariano Ruiz-Gayo
Mario Ruiz-Gonzalez
José Ruiz-Herrera
Ezequiel Ruiz-Mateos
Eliel Ruiz-May
Kepa Ruiz-Mirazo
Martin Ruiz-Ortiz
Victor L Ruiz-Perez
Magdalena Ruiz-Rodriguez
Cristina Ruiz-Romero
Marcos Ruiz-Soler
Victor Ruiz-Velasco
Stephen Rulisa
Nikolai Rulkov
Eric Rullman
Stuart Rulten
Kendra Rumbaugh
Martin Rumbo
Alice Rumbold
Darren Rumbold
Tillmann Rumenapf
Vivian Rumjanek
Andreas Rummel
Cornelia Rumpel
Tatjana Rundek
Jørgen Rungby
Kurt Runge
George Runion
Erik Runkle
James Runkle
Raymond Runyan
Justin Runyon
Anthony Ruocco
Arnold Ruoho

Margherita Ruoppolo
Jani Ruotsalainen
Thusitha Rupasinghe
Stefan Rupf
Maja Rupnik
Jan Rupp
Steffen Rupp
Etienne Ruppé
Werner Ruppitsch
Hans-Jürgen Rupprecht
Guillermo Rus
Horea Rus
Nancy Rusch
Laura Rusche
Massimiliano Ruscica
John Ruscio
Michael G. Ruscio
Roberto Rusconi
Stefano Rusconi
Rasa Ruseckaite
Alex Rushforth
Craig Rusin
Brian Russ
Aaron Russell
Anthony Russell
Armistead Russell
Avery Russell
Brenda Russell
Charles Russell
Colin Russell
David Russell
Fiona Russell
Fraser Russell
Gareth Russell
George Russell
Ginny Russell
Jacob Russell
James Russell
Jane Russell
John Russell
Margaret Russell
Mark Russell
Nerissa Russell
Rodney Russell
Steven Russell
Tanya Russell
Kasi Russell-Lodrigue
Maurizio Russello
Marion Russier
Eugenia Russinova
Alessandra Russo
Annapina Russo
Antonio Russo
Giandomenico Russo
Giorgio Ivan Russo
Giulia Russo
Ilaria Russo
Luca Russo
Maria Russo
Matteo Russo
Natalie Russo
Sarah Russo
Tommaso Russo
Vincenzo Russotto
Michael Russwurm
Tige Rustad
Ingo Rustenbeck
Sachin Rustgi
Alina Rusu
Matt Rutar
Abraham Rutchick
Christian Rüter
Matthias Ruth
Klaus Rüther
B. R. Rutherford
Elaine Rutherford
Helena Rutherford
Julian Rutherford
Mark Rutherford
Sarah Rutherford
Zoe Rutherford
Kay Rutherfurd
Eric Ruthruff
Justin Ruths
Sharon Ruthstein
Heloisa Rutigliano
Bastiaan Rutjens
I. Rutkai
Jessica Rutkoski
Seward Rutkove
Arkady Rutkovskiy
Lainie Rutkow
Tom Rutkowski
Adam Rutland
Paul Rutland
Daniel Rutledge
Frank Rutsch
Geert Rutten
Loes Rutten-Jacobs
Lukas Rüttiger
Johan Ruud
Olli Ruuskanen

Timo Ruusuvirta
Clandio Ruviaro
Jean-Marie Ruysschaert
Bente Ruyter
Daniel Ružek
Alexey Ruzov
Maurizio Ruzzi
Srikanth Ryali
Aimee Ryan
Alan Ryan
Bridget Ryan
Colleen Ryan
Cormac Ryan
John Ryan
Martin Ryan
Michael Ryan
Robert Ryan
Terence Ryan
Thomas Ryan
Fanny Rybak
Jürgen Rybak
Leonard Rybak
Emma Rybalka
Ed Rybicki
Evgeny Rybin
Krystyna Rybka
Grigori Rychkov
Ivan Rychlik
Malgorzata Rychlowska
Jan Rychtar
Kelli Ryckman
Artur Rydosz
Chris Ryerson
Rafal Rygula
Ryan Rykaczewski
Jamie Rylance
Chris Rylander
Bartosz Rylski
Amanda Rymal
Tasmin Rymer
Agnieszka Rynda-Apple
Evelyn Rynkiewicz
Angela Rynne Vidal
Hyung Don Ryoo
Hyun-Mo Ryoo
Na Kyung Ryoo
Eduard Ryschich
Marc Ryser
Mina Ryten
Seppo Rytkönen
Jong Hoon Ryu
Keun Ho Ryu
Sangryeol Ryu
Sung Ho Ryu
Dmitry Ryvkin
Patricia Rzezak
Witold Rzyman
Gopala Krishnan S.
Radha S.
Arni S.R. Srinivasa Rao
Ramesh S.V.
Gaurisankar Sa
João Carlos Sa
Bechara Saab
Gloria Saab-Rincón
Farid Saad
Sara Saad
Sonia Saad
Ann Saada
Assieh Saadatpour
Debora Saade
Elie Saade
Michael Saag
Hannes Saal
Tiina Saanijoki
Elizabeth Saarel
Olli Saarela
Seppo Saarelainen
Tarja Saaresranta
Teijo Saari
Mart Saarma
Urmas Saarma
Norah Saarman
Annika Saarto
Aldo Saavedra
Carlos Saavedra
Emma Saavedra
Juan Saavedra
Keene Saavedra
Miguel Saavedra
Vincent Saba
Farnaz Sabahi
Mark Sabaj
Daniel Saban
Charumathi Sabanayagam
Nasser Sabar
Marion Sabart
Akmal Sabarudin
Anna Sabat
Artur Sabat
Pablo Sabat
Joan Sabaté

Gerardo Sabater-Grande
Ana Sabates
Rodolphe Sabatier
Marina Sabatini
Denise Sabatino
Laura Sabatino
Maurizio Sabatti
Ahmed Sabbah
Steffanie Sabbaj
Massimo Sabbatini
Koen Sabbe
Bastian Sabel
Poune Saberi
Claude Sabeta
Sanjeeve Sabharwal
Severine Sabia
Eduard Sabido
Olivia Sabik
Caroline Sabin
Caetano Sabino
Joao Sabino
Pamela Sabioni
Gaurav Sablok
Roy Sabo
Tara Sabo-Attwood
Ali Sabri
Nirmeen Sabry
Juan Sabuco
Mert Sabuncu
Ahmet Sacan
Paola Sacchi
Roberto Sacchi
Veronica Sacchi
James Sacco
Patrick Saccone
Pawel Sachadyn
Monika Sachadyn-Król
Mariusz Sacharczuk
Bindiya Sachdev
Monika Sachdev
K.S. Sachdeva
Sonya Sachdeva
Lucia Maria Sacheli
Chetana Sachidanandan
George Sachs
Martin Sachs
Michael Sachs
Martin Sack
Ulrich Sack
Dan Sackett
Ben Sacks
Emma Sacks
Frank Sacks
Subash Sad
Tetsushi Sadakata
Prabodh Sadana
Manish Sadarangani
Sivalal Sadasivan
Masoumeh Sadeghi
Soroush Sadeghi
Hesham Sadek
Samy Sadek
Gökhan Sadi
Ruxana Sadikot
Kashif Sadiq
Amena Sadiya
Seyed Sadjadi
Said Sadki
Patrick Sadoghi
Laura Sadori
Eran Sadot
Yoel Sadovsky
Joanna Sadowska
Izabela Sadowska-Bartosz
Alireza Sadr
Karim Sadr
Sunitha Sadula
Kourosh Saeb Parsy
Eva Saedder
A. Saeed
Anwaar Saeed
Farhan Saeed
Hamid Saeed
Mahdi Saeed
Maythem Saeed
Mohsan Saeed
Rafat Saeed
Sahar Saeed
Claude Saegerman
Chihiro Saegusa
Hiroshi Saeki
Xavier Saelens
Lorena Saelices
Pablo Saenz-Agudelo
Andrea Sáenz-Arroyo
Bernt-Erik Sæther
Stig Arve Sæther
Marina Saetta
Claudia Saez
Fabrice Saez
Sara Saez Atienzar
Marco Safadi

Naser Safaie
Ilgar Safak
Mohammad Safari
Ali Reza Safarpour
Basmah Safdar
Stephen Safe
Joshua Safer
Saveez Saffarian
Mona Saffarzadeh
Robert Safirstein
Susan Safley
Olga Safronova
Audrey Saftlas
Manish Sagar
Andrew Sage
Mehrnoosh Saghizadeh
Ronit Sagi-Eisenberg
Ramesh Sagili
M. Saglam
Murat Saglam
Giuseppe Saglio
Evangelista Sagnelli
Konstantinos Sagonas
Pratha Sah
Robert Sah
Amartya Saha
Chabita Saha
Chandan Saha
Shubhayu Saha
Sibu Saha
Sushanta Saha
Ziad Sahab
Goutam Sahana
Dushyant Sahani
Sharonit Sahar-Helft
Pipsa Saharinen
Anagh Sahasrabuddhe
Gaurav Sahay
Manisha Sahay
Peeyush Sahay
Ratnesh Sahay
Satya Sahay
Seema Sahay
Baljinder Sahdra
Srujana Sahebjada
Orhan Sahin
Ozgur Koray Sahingoz
Sinem Sahingur
Steffen Sahl
Sven Sahle
Veronica Sahlén
Olle Sahler
Cecilia Sahlgren
Laura Sahm
Sanjeev Sahni
Daisy Sahoo
Lingaraj Sahoo
Nirakar Sahoo
Rupam Sahoo
Sambit Sahoo
Sanjaya Sahoo
Satyaprakash Sahoo
Daljit Singh Sahota
Adolfo Saiardi
Nelson Saibo
Catherine Said
Hamid Said
Maria Said
Mas Ayu Said
Salah Said
Massoud Saidijam
Abdul Saied
Nahel Saied
Mauro Saieg
Romesh Saigal
Hiroto Saigo
Masayuki Saijo
Yasuaki Saijo
Shizuya Saika
Yoshikatu Saiki
Hemanta Saikia
Verena Sailer
Corinne Sailleau
Steve Sain
Arun Saini
Deepak Saini
Hemraj Saini
Manisha Saini
Sharanjot Saini
Vikram Saini
Amanda Sainsbury
David Saint
Dave Saint-Amour
Johanne Saint-Charles
Magali Saint-Geniez
Cristina Sainz-Borgo
Yoshifumi Saisho
Adnan Saithna
Atsushi Saito
F. Saito
Hiroaki Saito
K. Saito

Kanako Saito
Laurel Saito
Mariko Saito
Mayuko Saito
Priscila Saito
Ryuta Saito
Satoru Saito
Shigeki Saito
Shigeyiki Saito
Tais Saito
Takehiko Saito
Takeo Saito
Takeshi Saito
Tami Saito
Tomohito Saito
Yoshiki Saito
Kazuyoshi Saitoh
Kenji Saitoh
Noriko Saitoh
Yumiko Saito-Nakano
Antonino Saitta
Eduardo Saiz
Inigo Saiz-Fernandez
Arnaud Saj
Stefano Zauli Sajani
Sima Sajedinejad
Hideo Saji
Imran Sajid
Umadevi Sajjan
Mamta Sajwan-Khatri
Kazuhiko Sakaguchi
Koichi Sakaguchi
Lynn Sakai
M. Sakai
Masaru Sakai
Ryuichi Sakai
Takayoshi Sakai
Toshiyuki Sakai
Yuichi Sakairi
Hiroyuki Sakakibara
John Sakaluk
Ryotaro Sakamori
Ayako Sakamoto
Kenji Sakamoto
Kensuke Sakamoto
Maiko Sakamoto
Masahiro Sakamoto
Masaki Sakamoto
Masaya Sakamoto
Wataru Sakamoto
Yuichi Sakamoto
Seiichiro Sakao
Kazuko Sakata
Masayo Sakata
Naoaki Sakata
Hiroshi Sakaue
Grigorios Sakellariou
Khashayar Sakhaee
Prashant Sakharkar
Amirhossein Sakhteman
Kazuo Sakka
Samir Sakka
Giorgos Sakkas
Lazaro I. Sakkas
George Sakoulas
Priyanka Saksena
Seema Saksena
Sumeet Saksena
Amanda Saksida
Takashi Saku
Atsushi Sakuma
Tetsushi Sakuma
Anavaj Sakuntabhai
Takaya Sakura
Yasuhito Sakuraba
Tsukasa Sakurada
Tsutomu Sakurada
Yoichi Sakurada
Hidetoshi Sakurai
Hiroyuki Sakurai
M. Sakurai
Samuel Sakyi
M.M. Sala
Concepcion Salaberria
David Salac
Srinivas Saladi
Francoise Salager-Meyer
Mohammed Saji Salahudeen
Muhammad Salam
Rehana Salam
Guy Salama
Joseph Salama
Pariya Salami
Simpa Salami
Nicolas Salamin
Irene Salamon
Justin Salamon
Daniel Salamone
Asaf Salamov
Antonio Salar
Eva Salas
Leonardo Salas

Jordi Salas-Salvadó
Marcel Salathé
Aleix Sala-Vila
Gloria Salazar
Mariano Salazar
Valerie Salazar
J. Michael Salbaum
Mauricio Salcedo
Ian Saldanha
Sandra Saldivia
Raquel Sá-Leão
Paul Saleeb
Moin Saleem
Sarah Saleem
Marwah Saleh
Omar Saleh
Leili Salehi
Ali Salehzadeh-Yazdi
Miroslav Salek
Mohamed Salem
N.Y. Salem
Elma Salentijn
Michael Salerno
Nadia Salerno
Verônica P. Salerno
Rosangela Salerno-Goncalves
Anne Sales
Celia Sales
Antonio Salgado
Cassandra Salgado
Roberto Salgado
Marcello Salgado Filho
Wilmara Salgado-Pabon
Padmini Salgame
R.K. Salgotra
Elie Saliba
Muneeb Salie
Samina Salim
Bharathi Salimath
Irma Salimovic-Besic
Elena Salina
Armando Salinas
Gustavo Salinas
Irene Salinas
Patricia Salinas
Amy Salisbury
Chris Salisbury
Glenn Salkeld
Dina Salkovic
Hesham Sallam
Mohd Zaki Salleh
Salam Salloum-Asfar
Stephen Salloway
Jorge Salluh
Maria Anice Sallum
Rodney Salm
Dahlia Salman
Mehrdad Salmasi
Marko Salmi
Mikko Salminen
Adam Salmon
Yann Salmon
Robert Salmond
Vsevolod Salnikov
Konstantin Salnikow
Perttu Salo
Tuula Salo
Johanna Salomon
Alberto Salomone
Federico Salomone
Brendan Saloner
Ettore Salsano
Humam Saltaji
Frédéric Saltel
Anita Saltmarche
Stephen Salton
Fréderik Saltré
Wendy Saltzman
Ashok Saluja
Liliana Salvador
Raquel Salvador
Gianluca Salvagno
Ioanna Salvarina
Domenico Salvatore
Francesco Salvatore
Mary Salvatore
Renato Salvatteci
Valentina Salvestrini
Mario Salvi
Silvio Salvi
Leonardo Salviati
M. Constantine Samaan
Deborah Samac
Rodney Samaco
Yaser Samadi
Nadia Sam-Agudu
Taraz Samandari
Gregory Samanez-Larkin
Carole Samango-Sprouse
Afshin Samani
Mrinal Samanta
Palash Samanta

Saheli Samanta
Supriti Samantaray
Nezar Samarah
Amali Samarasinghe
Amanda Samarawickrama
Eric Samarut
Lobelia Samavati
Srinivas Samavedam
Kumar Sambamurti
Murali Sambasivan
Fabio Sambataro
Hiroaki Samejima
Diana Samek
Benjamin Samelson-Jones
Jonathan Samet
Jameal Samhouri
Arun Samidurai
Marcin Samiec
Konstantinos Samitas
Stefan Sammito
Helen Sammons
R. Sammons
R. Douglas Sammons
Melita Samoilys
Sara Samoni
Aline Sampaio
Cesar Sampaio
Paula Sampaio
Bhaven Sampat
Hemal Sampat
Karuna Sampath
Kesava Kovanur Sampath
Venkatesh Sampath
Sruthi Sampathkumar
Jelmer Samplonius
Geoff Sampson
Matthew Sampson
Marsha Samson
Rebekah Samsonraj
Ferenc Samu
Chandramathi Samudi Raju
Buck Samuel
Didier Samuel
Douglas Samuel
Gabrielle Samuel
Preethy Samuel
William Samuel
Aaron Samuels
Fiona Samuels
Gary Samuels
Joshua Samuels
Noah Samuels
James Samuelson
Kristin W. Samuelson
Anne-Maj Samuelsson
Mehmet Samur
Michael Samways
Michael San Francisco
Alejandro San Juan
Rody San Martín
Phillip San Miguel
Diego San Millan Ruiz
Thibault Sana
Adnen Sanaa
Sabri Sanabani
Daniel Sanabria
Hiromi Sanada
Marcos Sanches
Aranzazu Sanchez
Benjamin Sanchez
Bruno Sanchez
Christopher Sanchez
David Sanchez
Diana Sanchez
Elena Sanchez
Eugenia Sanchez
Hector Sanchez
Isabel Maria Sanchez
Karen Sanchez
Luis Sanchez
Mairena Sanchez
Manuel Sanchez
Mar Sanchez
Marie-Pierre Sanchez
Susan Sanchez
Yolanda Sanchez
Sergio Sànchez
Marta Sánchez de la Torre
Alvaro Sanchez Ferro
Carmen Sánchez Gonzalez
Francisco Sánchez Vázquez
Antonio Sanchez-Amat
Victor Javier Sanchez-Arevalo
Francisco Sanchez-Bayo
Carlos Felix Sanchez-Ferrer
Miguel Sanchez-Garcia
M. Victoria Sánchez-Gómez
Myriam Sánchez-Gómez
Jorge Sánchez-Guerrero
Fermin Sanchez-Guijo
Ernesto Sanchez-Herrero
Virginia Sánchez-Marcos

Adrián Sánchez-Montalvá
Juan S. Sánchez-Oliver
Santiago Sánchez-Pagés
Arturo Sánchez-Paz
Ricardo Sanchez-Prieto
Fernando Sanchez-Santed
José Sánchez-Tomero
Carmen Sanchez-Torres
Patricia Sanchez-Velazquez
Santiago Sanchez-Vicente
Marcelo Sánchez-Villagra
Jose Sanchez-Zapata
Pau Sancho-Bru
Lena Sanci
Michael Sand
Luisa Sandalio
Edwin Sandanaraj
Manoharie Sandanayaka
Frida Sandberg
Kelly Sandberg
Nathan Sandbo
Brody Sandel
Michael Sandel
Beata P. Sander
Dirk Sander
Josemir Sander
Kerstin Sander
P. Martin Sander
Brett Sandercock
G.R.H. Sandercock
Barbara Sanders
Christian Sanders
Eduard Sanders
James Sanders
Kenton Sanders
Phil Sanders
Robert Sanders
Rogier Sanders
Tanja Sanders
Charlotte Sandersen
Ashley Sanders-Jackson
Kiran Sandhu
Paul Sandifer
Martin Sandig
Kari Sand-Jecklin
Ben Sandkam
Robin Sandkühler
Irwin Sandler
Netanya Sandler
Wendy Sandler
Johanna Sandling
Jonas Sandlund
Curt Sandman
Suzanne Sandmeyer
David Sando
Peter Sandoee
Leonidas Sandoval
Luis Sandoval
Ionel Sandovici
Jean-Christophe Sandoz
Olivier Sandra
Marco Sandri
Brian Sandroff
Emily Sands
Scott Sands
Ulf Sandström
Daniel Sandweiss
Monica Sañé Schepisi
Eric Sanford
Rachel Sanford
Robert Sanford
Huiyan Sang
Nianli Sang
Shengmin Sang
Yongming Sang
Gareth Sanger
Florencia Sangermano
Morgan Sangeux
Haleh Sangi
Federica Sangiuolo
Kazunori Sango
Bruno Sangro
George Sangster
Anawin Sanguankeo
Karen Sanguinet
N. Sangwan
Rajender Singh Sangwan
Davison Sangweme
Mario Sanhueza
O. Saniel
Gangadhar Sanjayan
Nobuo Sanjo
Luis San-Jose
Alejandra Sanjuan Pla
Adam Sanjurjo
M. Jeeva Sankar
Pamela Sankar
Renu Sankar
Uma Sankar
Saikolappan Sankaralingam
R. Sankaranarayanan
Sumathi Sankaran-Walters

Ramasubbu Sankararamakrishnan
Valerie Sankatsing
Raimon Sanmarti
Isabel Sanmartin
Juan C. SanMiguel
Mattia Sanna
Serena Sanna
Michel Sanner
Hiroko Sano
Motoaki Sano
Teruo Sano
Yukie Sano
Gerard Sanromà
Giovanni Sansoè
Stephanie Sansom
Clementina Sansone
Andrea Sant
Vinerte Santa
Anthony Santago
Carlo Santaguida
Alfredo Santalla
Abel Santamaria
Alberto Santamaria
Juanjo Santamaria
Cesar Santa-Maria
Joan Santamaría
Paolo Santambrogio
Sharlene Santana
Nalini Santanam
Adam Santanasto
Javier Santander
Elisa Santandrea
Giovani Santangelo
Philip Santangelo
Enrica Santarcangelo
Michael Santare
Andrea Santarelli
Regina Santella
John Santelli
Marco Santello
Eva Santermans
Miklós Sántha
K.G. Santhya
Paolo Santi
Karina Santiago
Marie Laure Santiago-Raber
Marina Santic
Mark Santillan
C. Santín
Luca Santini
Valeria Santini
George Santis
Debora Santo
Marc Santolini
B.G. Santoni
Daniele Santoni
Giorgio Santoni
Manuel Santonja
Filippo Santorelli
Alessandra Santoro
Domenico Santoro
Marco Santoro
Mario Santoro
Nicola Santoro
Rosaria Santoro
Adalberto Santos
Angela Santos
Cecilia Santos
Cláudia Santos
Flavia Santos
Fran Santos
Francisco de Assis Ribeiro dos Santos
Guido Santos
José Santos
Jose Maria Santos
Josefina Santos
Lucio Santos
Luísa Santos
Maria M. M. Santos
Mauro Santos
Mudjekeewis Santos
Renato Santos
Robson Santos
Sérgio Henrique Sousa Santos
Sílvio Santos
Sónia A. O. Santos
Lara Lívia Santos da Silva
Ailiana Santosa
Jordan Santos-Concejero
Maria Santos-Galindo
Carlos Santos-Gallego
Alejandro Santos-Lozano
Carlos Santos-Ocaña
Filipe Santos-Silva
Sampa Santra
Annalisa Santucci
Carla Sanvicente-Vieira
A. Sanyal
Mrinmoy Sanyal
Suparna Sanyal
Jesus Sanz
Joaquin Sanz Remon

Simona Marianna Sanzani
C. Sanz-Lazaro
Alfredo Sanz-Medel
Victoria Sanz-Moreno
Roberto Sanz-Requena
Dam Sao Mai
Carlos São-José
Sean Sapcariu
Elizabeth Sapey
Przemyslaw (Mike) Sapieha
Paul Sapienza
Anna Sapino
Matthew Sapio
Gonzalo Sapisochin
M. Saponari
Anna Sapone
Rebecca Sappington
Thomas Sappington
Sameer Saproo
Anil Sapru
Mahesh Saqcena
Uzma Saqib
Maurizio Sarà
Santosh Saraf
Bruno Saragiotto
Andre Saraiva
Margarida Saraiva
Roberto Saraiva
Anni Saralahti
Jari Saramaki
Indrani Saran
Debalin Sarangi
Babak Sarani
Stephanie Sarantopoulos
Antti Saraste
Jaakko Saraste
Gautam Sarath
Rathindra Sarathy
Vanessa Sarathy
Thanos Saratzis
Ganapathy Saravanan
Jordy Saravia
Patricia Saravia-Otten
Gennaro Sardella
Beata Sarecka-Hujar
David Sargan
James Sargent
Ernest Sargsyan
Bruno Sargueil
Ibrahim Sari
Selahattin Saribay
Zenia Saridaki
Mehmet Sarikaya
Emine Ulku Saritas
K. Saritha
Ilker Sariyer
Temel Sariyilidiz
Balázs Sarkadi
Teppo Särkämö
Banwarilal Sarkar
Chinmoy Sarkar
Dibyendu Sarkar
Dipak Sarkar
Fazlul Sarkar
Jayant Sarkar
Joy Sarkar
Neelakshi Sarkar
Sailendra Sarkar
Santosh Kumar Sarkar
Saumendra Sarkar
Shubhashish Sarkar
Siddharth Sarkar
Somwrita Sarkar
Sudeep Sarkar
Uttam Sarkar
Jann Sarkaria
Pallab Sarker
Rim Sabrina Jahan Sarker
Shah-Jalal Sarker
Marianna Sarkissyan
Peter Sarlin
Birinchi Sarma
Devojit Kumar Sarma
Nayan Sarma
Ramendra Sarma
Loredana Sarmati
Gabriella Sarmay
A. Sarmento
Hugo Sarmento
R. Sarmugam
Sanjeev Sarmukaddam
Eli Sarnat
Stefania Sarno
Clea Sarnquist
Marco Saroglia
Jasmine Saros
Mirkka Sarparanta
Demba Sarr
Michael Sarras
Oriane Sarrasin
Jean Philippe Sarrette
Maria-Rosa Sarrias

Alejandro Sarrion-Perdigones
Jerome Sarris
Salvador Sarró
Elena Sarropoulou
Marcello Sartarelli
Massimo Sartelli
Vijay Sarthy
Laura Sartiani
Claudio Sartini
R. Balfour Sartor
R.B. Sartor
Andrea Sartore Bianchi
Daniela Sartorelli
Stephane Sartoretto
Alexandrina Sartori
Luisa Sartori
Maria Teresa Sartori
Alexander Sartorius
Tina Sartorius
Leonie Sarubbo
Pernille Sarup
Junji Saruwatari
John Sarwark
Masakiyo Sasahara
Shinichiro Sasahara
Taeko Sasai-Sakuma
Hideyuki Sasaki
K. Sasaki
Ken-ichiro Sasaki
Tamaki Sasaki
Tetsuhiko Sasaki
Tsuyoshi Sasaki
Tomohiro Sasanami
Delphine Sasanguie
Shizuka Sasazuki
M. Sasikala
Smitha Sasindran
Christopher Saski
Prakash K. Sasmal
Muhammad Bayu Sasongko
Teguh Haryo Sasongko
Andrea Sass
Claudia Sassenrath
Gretchen F. Sassenrath
Jennifer Sasser
Lauren M. Sassoubre
Amen Sassy
Inka Sastalla
Jaume Sastre-Garriga
Masataka Sata
Honoo Satake
Vijaya Satchidanandam
Sadhana Sathaye
Santosh Sathe
D. Sather
Noah Sather
William Sather
Brijesh Sathian
Gnanasekar Sathishkumar
Muthukrishnan Sathiyabama
Thozhukat Sathyapalan
Jitendra Satija
Jonathan Satin
Leslie Satin
Claudia Satizabal
Saulius Satkauskas
Lisa Satlin
Ilhan Satman
Ana Sato
Ana Paula Sato
Atsushi Sato
Brian Sato
Chloe Sato
Douglas Sato
Hidetoshi Sato
Hiroshi Sato
Hirotoshi Sato
Joao Sato
Kei Sato
Keisaku Sato
Koji Sato
Maria Sato
Masa Sato
Masamitsu Sato
Misako Sato
Shuichi Sato
Shunichi Sato
Takaaki Sato
Takami Sato
Takuichi Sato
Takuya Sato
Tetsuya Sato
Wataru Sato
Yasuto Sato
Yutaka Sato
Ayano Satoh
Takashi Satoh
Anjali Satoskar
Yorifumi Satou
Zlatko Satovic
Jorgina Satrustegui
Jetsumon Sattabongkot

Sampurna Sattar
Terre Satterfield
Vishnupriya Satti
Frank Sattler
Martin Sattler
Michael Sattler
Pratik Satya
Kapaettu Satyamoorthy
Srinath Satyanarayana
Veena Satyanarayana
Ebenezer Satyaraj
Catherine Satzke
Lawrence Saubermann
Christophe Sauboin
Sarah Sauchelli
Christian Sauder
Ahmed Saudi
Sascha Sauer
Robert Sauerwein
Ola Saugstad
Simon Saule
Jean-Sébastien Saulnier-Blache
Bryan Saunders
Charles Saunders
Christobel Saunders
Erika Saunders
Jake Saunders
Jeff Saunders
John Saunders
Kate Saunders
Megan Saunders
Milda Saunders
Norman Saunders
Pamela Saunders
Ryan A. Saunders
Thomas Saunders
Herbert Sauro
Khara Sauro
Olivier Saut
Daniel Sauter
Carola Sauter-Louis
Anne Sauvaget
Fabien Sauvet
Nicolas Sauvion
Nathalie Sauvonnet
Christina Savage
Lisa Savage
Patrick Savage
Paul Savage
Shazly Savahl
Dennis Savaiano
Rashmin Savani
Marco Savarese
Philippe Savarin
Ricardo Savaris
John Savaryn
Cosmin Saveanu
Rodolfo Savica
Barry Saville
Marion Saville
Paul Saville
Jonathan Savin
Lara Savini
Raushan Savirov
David Savitz
Jean-Michel Savoie
Alena Savonenko
George Savva
Patrick Sawa
Zimi Sawacha
Atsushi Sawada
Kenjiro Sawada
Norie Sawada
Reiko Sawada
Yu Sawada
Yvonne Sawall
Devendra Sawant
Samir Sawant
Atomu Sawatari
Simon Sawhney
Nadia Sawicka-Gutaj
Gregory Sawicki
Lino Sawicki
Hemant Sawnani
Elinor Sawyer
Kayle Sawyer
Kelly Sawyer
Michael Sawyer
Roger Sawyer
Anita Saxena
Anjana Saxena
K. Saxena
Payal Saxena
Ruchi Saxena
S. Saxena
Tamsin Saxton
Jong Say
Ilyas Sayar
Sharon Saydah
Mohamed Sayed-Ahmed
Abu Sayeed
Aejaz Sayeed

John Sayer
Leyla Sayigh
Nihat Sayin
Megan Saylor
Zoe Saynor
Jessica Sayyad
Awis Qurni Sazilli
Hossain Sazzad
Orfeo Sbaizero
Catalina Sbert
Silviu Sbiera
Valerio Sbordoni
Dragos Sburlan
Andrew Scaboo
Paolo Scacciatella
Glenis Scadding
K. Scaglione
Pier Paolo Scaglioni
Carolina Scagnolari
Simona Scaini
Antonio Scala
Stefania Scala
Franco Scaldaferri
Stefano Scalercio
I.C. Scaletsky
Antonio Scalfari
Salvatore Scali
Joshua Scallan
Aylwyn Scally
Giancarla Scalone
Fabien Scalzo
M. M. Scandiani
Massimo Scandura
Colin Scanes
Charles Scanga
David Scanlan
Pauline Scanlan
Alessandro Scano
Morris Scantlebury
Bruno Scanu
Giovanni Scapagnini
Sandra Scapin
Luca Scapoli
Guido Scarabelli
Alessio Scarafoni
Silvia Scarano
Giseppe Scarascia-Mugnozza
Giuseppe Scarcella
Raquel Scarel-Caminaga
Joy Scaria
Isobel Scarisbrick
Federica Scarpina
Samuel Scarpino
Stefania Scarsoglio
Mario Scartozzi
Derek Scasta
Pietro Scaturro
Urs Schaad
Marco Schaaf
Anne Schaafsma
Sara Schaafsma
Benoist Schaal
Heiner Schaal
Mona Schaalan
Beatriz Schaan
Marcel Schaap
Benedikt Schaarschmidt
Jan Schaart
W. Schaasberg
Jenna Schabacker
Anja Schablon
Siobhan Schabrun
Ronald Schachar
Adena Schachner
Alexander Schachtrupp
Kyle Schachtschneider
Eva Schaden
J. Schaecter
Adam Schaefer
Brian Schaefer
Jake Schaefer
Jan Schaefer
Jerremy Schaefer
Karl-Herbert Schaefer
Michael Schaefer
Sabine Schaefer
Steve Schaefer
Tobias Schaefer
Ute Schaefer-Graf
Urs Schaefer-Rolffs
Joanna Schaenman
Christa Schafellner
Marissa Schafer
Michal Schafer
Zach Schafer
Hendrik Schäfer
Simon Schäfer
Thorsten Schäfer
Valentin Schäfer
Britta Schaffelke
David Schaffer
Kirsten Schaffer

Robert Schaffer
Stephen Schaffer
Christina Schäffer
Donald Schaffner
Susan Schaffnit
Hans-Georg Schaible
Helmut Schaider
Coby Schal
Kevin Schalinske
Inga Schalinski
Enrico Schalk
Joost Schalkwijk
Marc Schallenberg
Jörg Schaller
Nils Schallner
Renata Schama
Andrea Schamberger
Cynthia Schandl
Moses Schanfield
Jeffrey Schank
Marie-Claire Schanne-Klein
Alessandra Schanoski
Joost Schanstra
Moritz Schanz
William Schapaugh
Arnaldo Schapire
Ulrike Schara
Christopher Schardl
Christopher Scharer
Birgit Scharf
Christian Scharf
Frederick Scharf
Inon Scharf
Raphael Scharfmann
Walter Ernesto Schargel
Mathias Scharinger
Michael Scharl
Mathias Scharmann
Andrea Scharnhorst
Robert Scharpf
Joern Scharsack
Manfred Schartl
Heather Schatten
Heide Schatten
Jörn Schattenberg
Mirta Schattner
George Schatz
Jonathan Schatz
Annette Schaub
Michael Schaub
Wilmar Schaufeli
Katharina Schaufler
Gabriele Schaumann
Mia Schaumberg
Frieder Schaumburg
Robert Schaut
Valerie Schawaroch
Nils Schebb
Karl Schebesta
Adrienne Scheck
Martin Schecklmann
Manfred Schedlowski
Celena Scheede-Bergdahl
Todd Scheetz
Jacob Scheff
Sonja Scheffer
Carmen Scheibenbogen
Christoph Scheidegger
Blanca Scheijen
A. Scheim
Chelsea Schein
Samuel Scheiner
Stefan Scheiner
Steve Scheiner
Meir Scheinfeld
Dustin Scheinost
Katharina Scheiter
E. Glenn Schellenberg
Herbert Schellhorn
Jeffrey Schelling
Mirle Schemionek
Benjamin Schempf
Leonardo Schena
Gerhard Schenk
Manfred Schenk
Simon Schenk
Katerina Schenke
Silvia Schenone
Lynne Schepartz
Dmitry Schepaschenko
Filip Scheperjans
Paul Schepers
Victor Schepkin
Hagen Scherb
George Scherer
Marcia J. Scherer
Philipp Scherer
Rafal Scherer
Reinhold Scherer
Stephen Scherer
Suzanne Scherf
Ellen Scherl

Gerit Schernthaner
Douglas Scherr
Benoit Scherrer
Clemens Scherzer
Stefanie Scheu
Deborah Scheuer
Alexander Scheuerlein
Taneisha Scheuermann
Marina Scheumann
Christian Scheurig
Carina Schey
Anna Scheyett
Silvia Schiaffino
Lawrence Schiamberg
Augustin Schiariti
Giuditta Fiorella Schiavano
Stefania Schiavone
Rudolf Schicho
Udo Schickhoff
Nathalie Schicktanz
Edgar Schiebel
Matthew Schiefer
John Schieffelin
Günter Schiepek
Andreas Schiermeyer
Adam Schiffenbauer
Joshua Schiffer
Mark Schiffman
Julia Schiffner-Rohe
Adam Schikora
Tamara Schikowski
Nathan Schilaty
Stefan Schild
Steven E. Schild
Jonathan Schildcrout
Christina Schilder
Matthijs Schilder
Stefan Schildknecht
Oliver Schilke
Leonhard Schillbach
Stefan Schillberg
Frank Schiller
Martin Schiller
Birgit Schilling
Jonathan Schilling
Juan Schilling
Marcel Schilling
Oliver Schilling
Thomas Schilling
Pablo Schilman
Maarten Schim van der Loeff
Lisa Schimmenti
Victor Schinazi
Christian Schindelhauer
Rainer Schindl
Abigail Schindler
Simon Schindler
Susanne Schindler
Bernhard Schink
Alfred Schinkel
Alison Schinkel-Ivy
Alexandru Schiopu
Helgi Schioth
Ernestina Schipani
Jan Schipper
Matthew Schipper
Axel Schippers
Walter Schippinger
Chiara Schiraldi
Jan Schirawski
Peter Schirmbacher
Clemens Schirmer
Lyn-Rouven Schirra
Michael Schirrmann
Johannes Schittny
Michael Schivo
Nikolaos Schizas
Uffe Schjoedt
Natalia Schlabritz-Lutsevich
David Schlaepfer
Axel Schlagenhauf
Patricia Schlagenhauf
Oliver Schlager
Stefan Schlager
Michael Schlander
Luregn Schlapbach
Michael Schläppi
Angelika Schlarb
Peter Schlattmann
Uwe Schlattner
Robert Schlauch
Gottfried Schlaug
Nicolas Schlecht
Winfried Schlee
Patrick Schlegel
Paul Schlegel
Peter Schlegel
Sandra Schlegl
Yosef Schlein
Dorit Schleinitz
Christian Schlenstedt
Mark Schlesinger

William Schlesinger
Avner Schlessinger
Katalin Schlett
Johanna Schleutker
Georg Schlieper
Fernando Schlindwein
Barbara Schlingmann
Axel Schlitt
Mathias Schloegl
Florian Schlotter
Wolff Schlotz
Ursula Schlötzer-Schrehardt
Annette Schlueter
Klaus-Dieter Schlueter
Neil Schluger
Angela Schlumbaum
Yolanda Schlumpf
Günther Schlunck
Andreas Schlüter
C. Schmaderer
Alvin Schmaier
Rachel Schmale
Ralf Schmälzle
Ben Schmand
Michael Schmeisser
Eric Schmelz
Marty Schmer
Klaus Schmetterer
Leopold Schmetterer
Amy Schmid
Axel Schmid
Daniela Schmid
Florian Schmid
Holger Schmid
Jochen Schmid
Pirmin Schmid
Silvan Schmid
Stefan Schmid
Simon Schmidbauer
Robert Schmidhammer
Alexander Schmidt
Andre Schmidt
Angelika Schmidt
Axel Schmidt
Brian Schmidt
Burkhard Schmidt
Carl Schmidt
Christoph Schmidt
Daniel Schmidt
Eric Schmidt
Frank Schmidt
Gregory Schmidt
Gudula Schmidt
Hannes Schmidt
Heath Schmidt
Helena Schmidt
Herbert Schmidt
Isabell Schmidt
J. Michael Schmidt
Kerstin Schmidt
Krzysztof Schmidt
Laura Schmidt
M. Schmidt
Maria Schmidt
Michael Schmidt
Michael G. Schmidt
Nathan Schmidt
Patrick Schmidt
Paul Schmidt
Peter Schmidt
Rebecca Schmidt
Ruben Schmidt
Sabine Schmidt
Susanne Schmidt
Thomas Schmidt
Travis Schmidt
Warren Schmidt
Katharina Schmidt-Bleek
Ursula Schmidt-Erfurth
Heike Schmidt-Posthaus
Carsten Schmidt-Weber
Virginia Schmied
Daniela Schmieder
Michelle D. Schmiegelow
Mike Schmierbach
Cyril Schmit
Claus Peter Schmitt
Nathalie Schmitt
Thomas Schmitt
Marcus Schmitt-Egenolf
Thomas Schmittgen
Gerold Schmitt-Ulms
Andreas Schmitz
Anke Schmitz
Christoph Schmitz
Florian Schmitz
Gerd Schmitz
Ingo Schmitz
Josef Schmitz
Kathryn Schmitz
Norbert Schmitz
R. Schmitz

Thomas Schmitz
Ulf Schmitz
Bernd Schmitz-Dräger
Stephan Schmitz-Esser
Tanja Schmitz-Hübsch
Carsten Schmitz-Peiffer
Thomas Schmitz-Rixen
Michael Schmuker
Sheila Schmutz
Erich Schmutzhard
James Schnable
Tine Schnack
Jonathan Schneck
Daniel Schneditz
Alessandro Schneebeli
Gerald Schneeweiss
Ralf Schneggenburger
Antoine Schneider
Bruce Schneider
David Schneider
E. Marion Schneider
Erich Schneider
Frank M. Schneider
Frederic Schneider
Harald Schneider
Hartmut Schneider
Juliane Schneider
Kevin Schneider
Laura Schneider
Margaret Schneider
Maria Cristina Schneider
Markus Schneider
Michael Schneider
Peter Schneider
Robert Schneider
William Schneider
Jürgen Schneider-Schaulies
R. Schneier
Bettina Schnell
David Schnell
Nalani Schnell
Rick Schnellmann
Karin Schnetz
Astrid Schnetzer
Francisco Schneuer
Roman Schniepp
Leonhard Schnittger
Gavin Schnitzler
Paul Schnitzler
Martina Schnölzer
Tatiana Schnur
Thorsten Schnurbusch
B. Schnuriger
Theresia Schnurr
Silvia Schnyder
Stefan Schob
Christian Schöb
Joseph Schober
Patrick Schober
Otto Schoch
Michael Schocke
Dale Schoeller
Ludger Schoels
Marina Schoemaker
Minouk Schoemaker
Victor Schoenbach
Michael Schoenbaum
Felix Schoenbrodt
Lukas Schoenenberger
Bradley Schoenfeld
Paul Schoenhagen
Verena Schoepf
Randal Schoepp
Kim Schoessow
Anna Schoettle
R.A. Schoevers
Verena Schoewel
Annelot Schoffelen
Christopher Schofield
Gail Schofield
Paul Schofield
Robert Schofield
Steven Schofield
Timothy Schofield
Dirkjan Schokker
Sebastian Schölch
James Scholey
Sebastian Scholl
Michael Scholle
Steffen Scholpp
Denise Scholtens
Herman Scholthof
Clarke Scholtz
Alexander Scholz
Carsten Scholz
Chris Scholz
Christian F. P. Scholz
Glen Scholz
Henrike Scholz
Holger Scholz
Jaqueline Scholz
Stefan Scholz

Judith Schomaker
Lutz Schomburg
Georg Schomerus
Karin Schon
Daniele Schön
Bernd Schöne
Stefan Schöneich
Dustin Schones
Sabine Schönfeld
Gabriele Schönian
Uwe Schöning
Kristian Schønning
Peter Schönswetter
Robert Schooley
C. Mary Schooling
Catherine Schooling
Jon Schoonmaker
Kirsten E. Schoonover
Sebastian Schoppmann
John Schorge
Nicholas Schork
Sebastian Schornack
Michael Schotsaert
Eline Schotsmans
Anne-Marie Schott
Johanna Schott
Ulf Schott
Ulli Schotten
Elizabeth Schotter
Jennifer Schrack
Susanna Schraen
Ulrich Schraermeyer
Mark Schram
Miranda Schram
Maarten Schrama
Christoph Schramm
Tina Ken Schramm
Augusto Schrank
Ingrid Schraufstatter
Isabelle Schrauwen
Georg Schreckenbach
Barbara Schreiber
Darren Schreiber
Jacob Schreiber
Martin Schreiber
Stefanie Schreiber
Sabrina Schreiner
Thomas Schreiner
Michael Schreinlechner
Harald Schrem
Stephan Schreml
Michiel Schreuder
David Schriemer
Liesbeth Schrijvers
Lynn Schriml
Andreas Schröder
Sara Schröder
Ulrich Schröder
Wolfgang Schröder
Volker Schroeckh
Declan Schroeder
Frank Schroeder
Gregory Schroeder
Lee Schroeder
Mary Schroeder
Michael Schroeder
Susan Schroeder
Sven Schroeder
Thies Schroeder
Harry Schroeder Jr.
F. Schroeper
Kara Schroepfer
H.J. Schroers
Maya Schroevers
Henning Schroll
Martine Schroyen
Jacob Schrum
Amie Schry
Annie Schtscherbyna
Peter Schu
Andras Schubert
Brian Schubert
Jonathan Schubert
Philipp Schubert
Tilman Schubert
Florian Schuchardt
Rudi Schuech
Silke Schuele
Dirk Schueler
Mark Schuenke
Michael Schuepbach
Tobias Schuerholz
Andreas Schuering
Gordon Schuett
Ursel Schuette
Philipp Schuetz
Roman Schuetz
Pamela Schuetze
Scott Schuetze
Norbert Schuff
Kevin Schug
Stephan Schug

Klaus Schughart
Amy Schuh
Anna Schukat
David Schuldberg
Joseph Schuldenrein
Andreas Schuldt
Chrisitine Schuler
Rainer Schuler
Lavinia Schuler-Faccini
A.A. Schuller
Hildegard Schuller
Kathy Schuller
Gerald Schulman
Bruce Schulte
Fiona Schulte
Lisa Schulte
Mieke Schulte
Peter Schulte
Wilhelm Schulte-Mattler
Michael Schulte-Mecklenbeck
Holger Schultheis
Cristian Schulthess
Cesar Schultz
Eric Schultz
H. D. Schultz
Julia Schultz
Kirk Schultz
Krisann Schultz
Sandy Schultz
Susan Schultz
Stacey Schultz-Cherry
Anna Schultze
Joachim Schultze
Stefan Schultze-Mosgau
Zachary Schultzhaus
Benjamin Schulz
Georg Schulz
Geralyn Schulz
Holger Schulz
Peter Schulz
Richard Schulz
Rudiger Schulz
Sabine Schulz
Stefan Schulz
Almut Schulze
Kerry Schulze
Lars Schulze
Julian Schulze zur Wiesch
Karl Schulze-Hagen
Sven Schulzke
Ellen Schulz-Kornas
Bruce Schumacher
Eric Schumacher
Fredrick Schumacher
Julia Schumacher
Petra Schumacher
Robert Schumacher
Tracy Schumacher
Nathan Schumaker
Meredith C. Schuman
Ronnie Schumann
John Schuna
Heribert Schunkert
Celia Schunter
Jorg Schupbach
Gertraud Schüpbach-Regula
Jonas Schupp
Detlef Schuppan
Peter Schur
Nick Schurch
Roger Schürch
Claudia Schurmann
Annette Schürmann
Michael Schurr
Alexander Schuster
Christina Schuster
Jennifer Schuster
Susanne Schuster
Roger Schutgens
Russell Schutt
Fabian Schütt
Aletta Schutte
Brian Schutte
Dennis Schutter
Jörg Schüttrumpf
Michael Schutz
Alexander Schütz
Noemi Schuurman
Mara Schvarzstein
Christoph Schwab
Clarissa Schwab
Helmut Schwab
Jan Schwab
Stefan Schwab
Susanne Schwab
Yannick Schwab
John Schwabe
Justus Schwabedal
Martin Schwacha
Lori Schwacke
Margo Schwadron
Wilhelm Schwaeble

Thomas Schwaha
Markus Schwaiger
Beat Schwaller
Josèli Schwambach
Hermann Schwameder
Tanja Schwander
Lisa Schwanz
Philipp Schwartenbeck
Ann Schwartz
Bradley Schwartz
Dean Schwartz
Egbert Schwartz
Elisabeth Schwartz
Faina Schwartz
Gregory Schwartz
Hansen Schwartz
Herbert Schwartz
John Schwartz
Kevin Schwartz
Mark Schwartz
Nancy Schwartz
Peter Schwartz
Rachel Schwartz
Sheree Schwartz
Thomas Schwartz
Tonia Schwartz
Kevin Schwartzman
Christoph Schwarz
Edward Schwarz
Erich Schwarz
Karlheinz Schwarz
M.A. Schwarz
Michael Schwarz
Peter Schwarz
Sebastian Schwarz
Trude Schwarzacher
Anke Schwarzenberger
Evelin Schwarzer
Katerina Schwarzerová
D. Samuel Schwarzkopf
Daniel Schwarzkopf
Larissa Schwarzkopf
Thomas Schwarzl
Torsten Schwede
Edzard Schwedhelm
Jake Schweigert
Nicolas Schweighofer
Stefan Schweinberger
Andrew Schweitzer
Ronen Schweitzer
Wolf Schweitzer
Reinhard Schweitzer-Stenner
Herbert Schweizer
Jürg Schweizer
Matthias Schweizer
Roland Schweizer
Ryan Schweller
Michael Schwemm
Falk Schwendicke
Beate Schwer
Kathleen Schwerdtner Máñez
Christian Schwerk
Kathryn Schwertfeger
Jim Schwiegerling
Andreas Schwiertz
Benno Schwikowski
Dylan Schwilk
Raoul Schwing
Malte Schwinger
Susanne Schwinning
Amee Schwitters
Dominik Schwudke
Jürgen Schymeinsky
Joost Schymkowitz
Virginia Sciacca
Salvatore Sciacchitano
Sebastiano Sciarretta
Cristina Scielzo
Paola Scifo
Giuseppe Scionti
Anthony Sclafani
R. Scofield
Steve Scofield
Virginia Scofield
Caterina Scoglio
Sabino Scolletta
Chiara Scopa
Alison Scope
Kézia Scopel
Giancarlo Scoppettuolo
Alessandro Scorpecci
Diana Scorpio
Marco Scortichini
Antonella Scorziello
Morena Scotece
Katia Scotlandi
Alan Scott
Andrew Scott
Barry Scott
Benjamin Scott
Caitlin Scott
Daniel Scott

Daryl Scott
David Scott
Evan Scott
Fiona Scott
Graham Scott
Hayley Scott
Ian Scott
Isabel Scott
Jamie Scott
Jeffrey Scott
John Scott
Karen Scott
Linda Scott
Melanie Scott
Naomi Scott
Patrick Scott
Paul Andrew Scott
Peter Scott
Rachel Scott
Ryan Scott
Stephanie Scott
Thomas Scott
Whitney Scott
Claudia Scotti
Lorenza Scotti
Marco Scotti
Nicholas Scott-Samuel
Efstathia Scoulica
Anna Ivana Scovassi
Kristin Scrabis-Fletcher
Marianna Scrima
Chiara Scrocco
Domenico Scrutinio
Gianluca Scuderi
Anton Sculean
John Scullion
Eileen Scully
Erin Scully
Matthias Sczesny-Kaiser
Ali Sdiri
Stephen Seah
Abhik Seal
Andrew Seal
Marc Seal
Anna Seale
Holly Seale
Lucia Seale
Michael Seaman
Jeremy Seamans
Thomas Seamans
Giorgio Seano
Grant Searchfield
Christopher Searcy
William Searcy
Charles Searles
Dorothy Sears
M. Sebastian
Sarah Sebastian
Thomas Sebastian
Federico Sebastiani
M. Sebastiani
Yuri Sebastião
Lafont Sebastien
Mireille Sebbag
Sylvain Sebert
Ismail Sebina
Adriano Sebollela
Robert Sebra
Maamar Sebri
Antonio Secchi
Silvia Secchi
Paola Secchiero
David Secco
A. Sechman
Segundo Seclen
Christopher Secombes
Agnese Secondo
William Secor
Lukasz Seczyk
Riad Seddik
Jennifer Seddon
Yolande Seddon
Lisa Sedger
Mamdouh Sedhom
Ali Reza Sedighi
Constantine Sedikides
Ruth Sedlak
Claudia Sedlinsky
Steven P. Sedlis
Chee Gee See
Violaine See
Michael Seear
J.E. Seeb
Frank Seebacher
Hanno Seebens
Brian Seed
Patrick Seed
Teresa Seefeldt
Christian Seegelke
Manuel Seeger
Katrin Seeher
Corey Seehus

David Seekell
Janet Seeley
Ego Seeman
Ralf Seemann
Stefan Seemann
Torsten Seemann
Barry Seemungal
Teet Seene
Arivudainambi Seenichamy
Navindra Seeram
Christine Seers
Leopoldo Segal
Mark Segal
Osnat Segal
Joaquim Segales
Richard Segall
Deborah Segaloff
James Segars
Gernot Segelbacher
Daniel Segenreich
Charles Seger
Suzanne Segerstrom
Mohamed Seghier
Chiara Seghieri
Helmut Segner
Chris Segrin
Cheryle Seguin
Diego Segura
Remedios Segura
Isabel Segura-Bedmar
Jansen Seheult
Deepmala Sehgal
Pravin Sehgal
Sunish Sehgal
Archana Sehrawat
Brad Seibel
Verena Seibold
Bernhard Seiboth
Nabil Seidah
Amber Seidel
George Seidel
Gerald Seidel
Katja Seider
Amanda Seidl
Michael Seidl
Rachael Seidler
Christine Seidman
Ernest Seidman
Issaka Seidu
Mark Seielstad
Edith Seier
Sayed Seif el-Din
Merav Seifan
David Seifer
Erin Seifert
Ludovic Seifert
Michael Seifert
Ulrike Seifert
Thomas Seifert-Held
Vanadin Seifert-Klauss
Ali Seifi
B. Seifi
Arnaud Seigneurin
Koji Seike
Gabriela Seiler
Sarah Seiler
Michael Seimetz
Nick Seiter
Dallas Seitz
Peter Seizer
Marcel Seiz-Rosenhagen
Ervin Sejdic
Miho Sekai
N. Sekercioglu
Bülent Sekerel
Konjeti Sekhar
Sreeja Sekhar
Rajandeep Sekhon
George Seki
Hikaru Seki
Shuhji Seki
Miho Sekiguchi
Y. Sekine
Tetsuji Sekiryu
Yoichi Sekizawa
Alejandra Sel
David Sela
Michael Sela
Gloria Selabe
Melissa Selb
William Selbie
Edward Selby
Heather Selby
Mareike Selcho
Thomas Selden
Oksana Seldimirova
Lucy Seldin
Yvonne Selecki
Salih Selek
Majlinda Selenica
Jana Selent
Matt Self

Neil Seligman
Fatih Selimefendigil
Silvia Selinski
Heloisa Selistre de Araujo
Vitaly Selivanov
Galina Selivanova
Petra Selke
Kimberly Selkoe
Ana Maria Sell
Henrike Sell
Scott Sell
Adnane Sellam
Jeremie Sellam
Jacobo Sellares
Roberta Sellaro
Dyan Sellayah
Holly Sellers
Frederic Sellet
Damien Sellier
Pierre Sellier
Lorenz Sellin
Michael Sellix
Bret Sellman
Johann Sellner
Massimo Selva
Ravi Selvaganapathy
P.R. Selvakannan
Govindan Selvam
Angamuthu Selvapandiyan
Karuppaiyah Selvendiran
Joel Selway
Kassa Semagn
Kazue Semba
Jonathan Sembrano
Carlo Semenza
Viktoriya Semeshenko
Wael Semida
Gün Semin
David Seminowicz
Abdelhabib Semlali
Oliver Semmes
John Semmler
Nasser Semmo
José Sempere
John Semple
Malcolm Semple
Emily Sempou
Jeremy Semrau
Á.F. Semsei
Serafima Semyenova
Aditya Sen
Aritro Sen
Baha Sen
Kunal Sen
Parimal Sen
Ranjan Sen
Sanchayita Sen
Sevket Sen
Shaunak Sen
Sourav Sen
Subrata Sen
Tuhinadri Sen
Tarun Sen Gupta
Giovanni Sena
Marcelo Sena
David Senaeve
Shantibhusan Senapati
Adriano Senatore
Assunta Senatore
Sascha Senck
Magdalena Senderecka
Ramon Sendra
Alp Sener
Nevzat Sener
P. Seners
Julien Seneschal
Pierre Senesse
Erin Seney
Takeshi Senga
Manju Sengar
Bram Sengers
Toru Sengoku
Sarah Sengstake
Durba Sengupta
Mahuya Sengupta
Saikat Sengupta
Shiladitya Sengupta
Srikumar Sengupta
Satoru Senju
Stephen Senn
Irene Senna
Ladislav Šenolt
Laura Senovilla
Florian Sense
Muthappa Senthil-Kumar
Loïc Sentilhes
Serif Senturk
Cheong Hoon Seo
Jung Kwan Seo
Jung-Hee Seo
Keun Seok Seo
Kyoung Yul Seo

Min Seo
Sang Heui Seo
Sang Won Seo
Sang-Uk Seo
Sangwon Seo
Seok Kyo Seo
Yeon Seok Seo
Youngwoo Seo
Javier Seoane
Patricia Seoane-Collazo
Geun Hee Seol
Moon-Woo Seong
Frances Separovic
Romina Sepe
Zahra Sepehri
Allan Seppanen
Mikko Seppänen
Ralf Seppelt
Alison September
Seth Septer
Edwards Septimus
Endang Septiningsih
Jorge Sepulcre
Francisco V. Sepulveda
Osvaldo Jhonatan Sepulveda Villet
Juan Sepulveda-Arias
Andrea Sequeira
Francesco Sera
Renato Seracchioli
Katherine Serafine
Agnese Serafini
Nick Serão
Adriane Serapião
Lisa Serbin
Laura Serbus
Chanuki Seresinhe
Albrecht Serfling
Inna Serganova
Kjell Sergeant
Nicolas Sergeant
Stylianos Serghiou
Consolato Sergi
Pier Nicola Sergi
Vladimir Sergiev
Andrea Serino
Graham Serjeant
Natalie Serkova
Mireille Serlie
Walter Sermeus
Evelyne Sernagor
Joao Serodio
Karin Seron
Christine Seroogy
Ignacio Seropian
Alexander Serov
Ary Serpa Neto
James Serpell
Louise Serpell
Engin Serpersu
Fabio Serpiello
Alessandro Serra
Andreas Serra
Assumpta Serra
Consol Serra
Manuel Serra
Roberto Serra
Sara Serra
Lluís Serra-Majem
Adelfa Serrano
Antonia Serrano
Antonio Serrano
Cesar Serrano
David Serrano
Emmanuel Serrano
Irene Serrano
Jose Serrano
María Serrano
Oscar Serrano
Ricardo Serrano
Manuel Serrano Ríos
Antonio Serrano-Mislata
Ignacio Serrano-Pedraza
Juan Serrano-Rodríguez
José A. Serrano-Sanchez
Aurelio Serrao
Mariano Serrao
José Eduardo Serrão
Florenci Serras
David Serre
Marc Serre
Arnaud Serres
Deborah Serrien
Patrick Serruys
Gregor Sersa
Henry Sershen
Elisavet Serti
Sabrina Servanty
Chris Servheen
Sandra Servia-Rodríguez
John Service
Alain Servin
Thomas Serwold

Jun Sese
Parthasarathy Seshacharyulu
Mukund Seshadri
Polani Seshagiri
Venkatraman Seshan
Janakiram Seshu
Steven E. Sesnie
Guido Sessa
Paola Sessa
Julian Sessions
October Sessions
Francesca Seta
Vijayasaradhi Setaluri
Mamoudou Sétamou
Tesfaye Setegn
Ajay Seth
Pankaj Seth
Ratanesh Seth
William Sethares
Anirudh Sethi
Manveen Sethi
Sunil Sethi
Vaheshta Sethna
Kandan Sethumadhavan
Wendy Setiawan
Sunita Setlur
Hiroaki Setoguchi
Yin Setoh
Mitsutoshi Setou
Luca Settanni
Michele Settanni
Claudio Sette
Tirumala Bharani Kumar Settypalli
Torsten Seuberlich
Verena Seufert
Thomas Seufferlein
Laurent Seugnet
Terri Seuntjens
Alexandra Séverac Cauquil
Stefano Severi
John Severson
Eva Sevick-Muraca
Catherine Sevier
Jean Sevigny
Noemí Sevilla
Ricardo Sevilla-Escoboza
Matthew Sewell
Thomas Sewell
William Sewell
Vishanthie Sewpaul
Caroline Sewry
William Sexauer
Amber Sexton
Eithne Sexton
Pat Sexton
Tracy Sexton
Tsukasa Seya
Karl Seydel
Mohamed Ali Seyed
Negar Seyed
Mousavi Seyed Mohsen
Hans-Martin Seyfert
Ali Seyfoddin
Nicholas Seyfried
Thomas Seyfried
Nevra Seyhan
John Seykora
Thorsten M. Seyler
Gregory Seymann
Alexander Seymour
Berhane Seyoum
Leen Seys
Sven Seys
Ugur Sezerman
Spyros Sfenthourakis
Thomas Sferra
Petros Sfikakis
Chiarella Sforza
Susanna Sforzini
Cecilia Sgadari
A. Sgambato
Abdelghani Sghir
Fabio Sgolastra
Fabrizio Sgolastra
Jacopo Sgrignani
Aihua Sha
Li-Qing Sha
Qiuying Sha
Mona Shaaban
Farzin Shabani
Doron Shabat
John Shacka
Nick Shackel
Julia Shackelford
Nancy Shackell
David Shackleford
Mujeeb Shad
Luciana Machion Shaddox
Starley Shade
Babak Shadgan
Kamal Shadi
Shahzad Shaefi

Massiullah Shafaq-Zadah
Orie Shafer
Robert Shafer
Zachary Shaffe
Christopher Shaffrey
Asrul Shafie
Touran Shafiei
Muhammad Shafique
Sharoni Shafir
Bridget Shafit-Zagardo
Mohsen Shafizadeh
Birju Shah
Devendra Shah
Dhara Shah
Dhaval Shah
Dilip Shah
Gulzar Shah
Ira Shah
Ketan Shah
Maitri Shah
Monica Shah
Neeral Shah
Nigam Shah
Nirmish Shah
Pankaj Shah
Payal Shah
Prakeshkumar Shah
Prediman Shah
Prithvi Shah
Pushkar Shah
Raj Shah
Ravi Shah
Rucha Shah
Sameer Shah
Shagun Bhatia Shah
Swati Shah
Syed Shah
Tayyab Shah
Viral Shah
Yatrik Shah
Shai Shaham
Uma Shahani
Ron Shahar
Yuval Shahar
Syahrul Sazliyana Shaharir
Mohammad-Ali Shahbazi
Farzana Shaheen
Rubina Shaheen
Tharwat Shaheen
Zachary Shaheen
Armita Shahesmaeili
Mehdi H. Shahi
Mohamed Shahin
Renu Shahrawat
Farhad Shahsavar
Rahil Shahzad
Sheila Shaibu
Atik Shaikh
Sunita Shailajan
Kenneth Shain
Kathy Shair
Abdallah Shaito
Helena Shaked
Anisa Shaker
Jane Shakespeare-Finch
Mehdi Shakibaei
Elham Shakibazadeh
Vikram Shakkottai
Holly Shakya
Ali S. Shalash
Edith Shalev
Idit Shalev
Kavita Shalia
Joel Shalowitz
Yuk Sham
Narges Shamabadi
Majid Shaman
Donat Shamba
Ahmar Shamim
Reuben Shamir
Elana Shamji
Delva Shamley
Rany Shamloul
Rawand Shamoon
Shahaboddin Shamshirband
Uzma Shamsi
Mohammad Shamsudduha
Simukai Shamu
Baoci Shan
Chunlei Shan
Hongli Shan
Jing Shan
Jinjun Shan
Lei Shan
Letian Shan
Ling Shan
Tao Shan
Catherine Shanahan
Daniel Shanahan
Asifkhan Shanavas
Sid Shanbhag
Fiona Shand

Kate Shane
Phil Shane
R. Andrew Shanely
Ce Shang
Hui-Fang Shang
Jing Shang
Lei Shang
Shi Shang
Songhao Shang
Zengfu Shang
Zhouping Shangguan
Aparna Shankar
Premlata Shankar
Sunita Shankar
Sreejith Shankar Pooppanal
Sunita Shankaran
Manjunatha Shankarappa
Kartik Shanker
David Shanks
G. Dennis Shanks
Leslie Shanks
Orin Shanks
Robert Shanks
Erin Shanle
Virupaksha Shanmugam Harave
K. Shanmuganandan
Dhanansayan Shanmuganayagam
Santhanam Shanmughapriya
Jonathan Shannahan
Graeme Shannon
Thomas Shannon
Hinton Shanta
Meek Shantel
Lisa M. Shantz
Changwei Shao
Dan Shao
Di Shao
Guofan Shao
Hongbo Shao
Hui Shao
Jianlin Shao
Jian-Zhong Shao
Jin-Yu Shao
Junfei Shao
Lijian Shao
Mingfu Shao
Qing Shao
Qingsong Shao
Yaming Shao
Ying Shao
Yu-Yun Shao
Zengwu Shao
Zeshu Shao
Zheren Shao
Ron Shaoul
Benjamin Shapero
Michal Shapira
Art Shapiro
Bruce Shapiro
Eugene David Shapiro
Frederic Shapiro
Joseph Shapiro
Julie Shapiro
Lee Shapiro
Paul Shapiro
Shane Shapiro
Shashwat Sharad
Anjali Sharathkumar
Melissa Sharer
Behnam Sharif
Jafar Sharif
Mohsen Sharifi
Nima Sharifi
Somayeh Sharifi
Vandad Sharifi
Michael Sharkey
Thomas Sharkey
Abhay Sharma
Aman Sharma
Amit Sharma
Amitabh Sharma
Anayata Sharma
Anuj Sharma
Arati Sharma
Arundhati Sharma
Atul Sharma
Bimala Sharma
Cynthia Sharma
Deepak Sharma
Divya Sharma
G. Taru Sharma
Gaurav Sharma
Geetanjali Sharma
Gyan Sharma
Hari Sharma
Joe Sharma
K. Sharma
Koustubh Sharma
Krishna Sharma
Kulbhushan Sharma
Laxmi Kant Sharma
Madhuri Sharma

Manoj Sharma
Manu Sharma
Manuj Sharma
Megha Sharma
Mona Sharma
Monisha Sharma
Mridula Sharma
Mukut Sharma
Namrata Sharma
Naveen Kumar Sharma
Paresh Sharma
Pawan Sharma
Pranay Sharma
Pushpa Sharma
R.R. Sharma
Rajeev Sharma
Rajendra Sharma
Rajiv Sharma
Rakesh Sharma
Rakesh Kumar Sharma
Ram Sharma
Sadarshana Sharma
Sandeep Sharma
Sangeeta Sharma
Sherven Sharma
Shivani Sharma
Shruti Sharma
Shyam Sharma
SK Sharma
Sonika Sharma
Sunny Sharma
Surendra Sharma
Suresh Sharma
Swarkar Sharma
Umender Sharma
Varun Kumar Sharma
Vijay Sharma
Vishal Sharma
Vishwas Sharma
Vivek Sharma
James Sharman
Steve Sharman
Gonen Sharon
Claire Sharp
Debbie Sharp
Frank Sharp
Julia Sharp
Louise Sharpe
Paul Sharpe
Richard Sharpe
Christopher Sharpley
Brenton Sharratt
Nicola Sharratt
Michael Shashaty
L. S. Shashidhara
Andrey Shatrov
Ivan Shatsky
Robin Shattock
Milena Shattuck
Allen Shaughnessy
Kevin Shaughnessy
Donna Shaver
John Shaver
Alex Shaw
Andrey Shaw
Christopher Shaw
David Shaw
Evelyn Shaw
Gary Shaw
J. Shaw
Jacqui Shaw
Jared Shaw
John Shaw
Jonathan Shaw
Jonathan G. Shaw
Kristi Shaw
Lindsey Shaw
Pang-Chui Shaw
Paul Shaw
Rachel Shaw
Robin Shaw
Simon Shaw
Stephanie Shaw
Timothy Shaw
Ramzi Shawahna
Mohamed Shawarby
Jill Shawe
Anna Shawyer
Jay Shayevitz
Renat Shaykhiev
Dzmitry Shcharbin
Mikhail Shchepetilnikov
Halyna Shcherbata
Bing She
Jin-Xiong She
Jill Shea
John Shea
Patrick Shea
Neil Shear
Heather Sheardown
Darlene Shearer
Jane Shearer

Emily Shearier
Bryony Sheaves
David Shechter
Michael Sheehan
Penelope Sheehan
Rory Sheehan
Christy Sheehy
Shyr-Ming Sheen-Chen
Debdoot Sheet
Carrie Sheets
Lavinia Sheets
Sandra J. Shefelbine
Richard Shefferson
Cory Sheffield
Orit Shefi
Amy Sheflin
Kareem Shehab
Mohamed Shehab
Syed Shehnaz
Amarda Shehu
Sabir Shehzad
Nader Sheibani
Javaid Sheikh
Kazim Sheikh
Shehzad Sheikh
Sonia Sheikh
M. Sheikholeslami
Douglas Sheil
Lynette Shek
Paul Shekelle
M. S. Shekhar
G. Shekhawat
Avat Shekoofa
Kevin Shelburne
Kent Shelby
Eric Shelden
Kim Shelden
Fran Sheldon
I. Sheldon
Pavica Sheldon
Miriam Shelef
Scarlet Shell
Mack Shelley
Todd Shelly
Matan Shelomi
Anthony Shelton
Christian Shelton
Clough Shelton
Delia Shelton
Noula Shembade
H. Shemesh
Einat Shemesh Mayer
Jonathan Shemmell
A-dong Shen
Bing Shen
Changxian Shen
Changyu Shen
Chia-Ning Shen
Chia-Rui Shen
Chiayi Shen
Dehua Shen
Dejun Shen
Guohua Shen
Hao Shen
Hong Shen
Hsin-Hui Shen
Huangxuan Shen
Huawei Shen
Hui Shen
Huicong Shen
Jana Shen
Jiabin Shen
Jianhua Shen
Jianlin Shen
Jianzhong Shen
Jinbo Shen
Jincheng Shen
Jingshi Shen
Jun Shen
Kang-Ning Shen
Le Shen
Li Shen
Liming Shen
Lin Shen
Linlin Shen
Liquan Shen
Megan Shen
Miaogen Shen
Peiyan Shen
Qiang Shen
Qirong Shen
Shichen Shen
Shihua Shen
Wei Shen
Weifeng Shen
Xian Shen
Xiaolin Shen
Xiaoteng Shen
Xiaoyan Shen
Xilin Shen
Xinlian Shen
Xu Shen

Y. Shen
Yang Shen
Yanting Shen
Ying Shen
Yinzhong Shen
Yiping Shen
Zhigang Shen
Jayant Shenai
Edmond Shenassa
Philip Shenefelt
Changsheng Sheng
Chang-Sheng Sheng
Lianxi Sheng
Yang Sheng
Zhifeng Sheng
Zizhang Sheng
Shirish Shenolikar
Belle Damodara Shenoy
Niraj Shenoy
Shweta D. Shenoy
Elizabeth Shephard
Bryan Shepherd
Greene Shepherd
Jason Shepherd
John Shepherd
Megan Shepherd
Anna Sheppard
Carmen Sheppard
Charles Sheppard
Chris Sheppard
Dean Sheppard
Haynes Sheppard
Lianne Sheppard
Paula Sheppard
James Shepperd
Gene Sher
Brian Sheridan
Margaret Sheridan
Megan Sheridan
Khaled A. Sherif
Kevin Sherin
Lori Sherlock
Mark Sherlock
Craig Sherman
Elizabeth Sherman
John Sherman
Larry Sherman
Phillip Sherman
Suzanne Sherman
Sue Sherman-Broyles
Kathleen Sherman-Morris
Stanton Keith Shernan
Lorraine Sherr
Laura Sherrard
Barbara Sherry
David Sherry
Edward Sherwood
Graham Sherwood
James Sherwood
Joanna Sherwood
Nancy Sherwood
Namratha Sheshadri
Asha Shetty
Shishir Shetty
Sreerama Shetty
Vivekananda Shetty
Chau-Chyun Sheu
Meei-Ling Sheu
Shwu-Jiuan Sheu
Tzong-Jen Sheu
Maxim Shevtsov
Chwen-Yang Shew
Hemant Shewade
Lucy Shewell
Frank Shewmaker
Denis Sheynikhovich
Ainong Shi
Baochen Shi
Bing Shi
Chang Shi
Cheng-Min Shi
Chung-Sheng Shi
Cynthia Shi
Guo-Liang Shi
Haifei Shi
Hang Shi
Huai-Long Shi
Huidong Shi
J. Shi
Jianxin Shi
Jie Shi
Jingping Shi
Jinjun Shi
Jishu Shi
Jun Shi
Junfeng Shi
Kun Shi
Lanbo Shi
Lei Shi
Lianxuan Shi
Meiqing Shi
Min Shi

Nan Shi
Nianqing Shi
Qian Shi
Qiong Shi
Riyi Shi
Ruiqing Shi
Run Zhang Shi
Shaoping Shi
Shengbo Shi
Suhua Shi
Weibin Shi
Wenyin Shi
Xianming Shi
Xiaoguang Shi
Xiaohong Shi
Xiaoli Shi
Xiaoyang Shi
Xinguo Shi
Yanmei Shi
Yanping Shi
Yawei Shi
Yijun Shi
Yixin Shi
Yongyong Shi
Yufei Shi
Yulan Shi
Yuyan Shi
Yuying Shi
Zhanghong Shi
Zhendan Shi
Zheng Shi
Zhi Shi
Zhou Shi
Zhuanghua Shi
Zhumei Shi
Shine-Gwo Shiah
Rita Shiang
Stephen Shiao
Toshikazu Shiba
Yugo Shibagaki
Rei Shibata
Shigeru Shibata
Toshio Shibata
Mineko Shibayama
Jamil Shibli
Caroline Shiboski
Makoto Shibutani
Rahul Shidhaye
J. Shiea
Chi-Chang Shieh
Kun-Ruey Shieh
Sheau-Yann Shieh
Aaron Shield
Alexandra Shields
Anthony Shields
Cleveland Shields
Patricia Shields
Richard Shields
Ryan Shields
Shannon Shields
Timothy Shields
Alan Shiels
Meredith Shiels
Melashu Shiferaw
Adrian Shifren
Takashi Shigematsu
Jun Shigemura
Shuichi Shigeno
Ohno Shigeru
Naoki Shigi
Masayoshi Shigyo
Arthur Chun-Chieh Shih
H. T. Shih
Hung-Jen Shih
Jean Shih
Ludy Shih
Ming-Chieh Shih
Ming-der Shih
Patrick Shih
Shin-Ru Shih
Shu-Fang Shih
Lamya Shihabuddin
Akihiro Shiina
Masaaki Shiina
Teiichiro Shiino
Jonathan Shik
Toshiharu Shikanai
Shuichi Shikano
Takahito Shikano
Ariella Shikanov
Kenichi Shikata
Ronald Shikiya
Nicole Shilkofski
Andrey Shilnikov
Joseph Shiloach
Yosef Shiloh
Eun Bo Shim
Seung-Cheol Shim
Sung Han Shim
Won Joon Shim
Won-Bo Shim
Yosio Shimabukuro

Hideaki Shimada
Hiroaki Shimada
Hiroyuki Shimada
Kazunori Shimada
Masakazu Shimada
Masayuki Shimada
Satoshi Shimada
Tomohiro Shimada
Tadashi Shimamoto
Hitoshi Shimano
Hitoshi Shimasaki
Ichiro Shimatani
Ron Shimelmitz
Hiroshi Shimizu
Ippei Shimizu
Jun Shimizu
Makoto Shimizu
Maria Heloisa Shimizu
Tatsuya Shimizu
Tomoharu Shimizu
Toru Shimizu
Toshihiko Shimizu
Yuko Shimizu-Motohashi
Shigeto Shimmura
Hiroshi Shimoda
Kazuya Shimoda
Shingo Shimoda
Yasuyuki Shimohigashi
Yoshihiro Shimoji
Tsuyoshi Shimomura
Ayako Shimono
Joshua Shimony
Takeshi Shimosato
Koichi Shimotori
Mary Shimoyama
Derya Shimshek
Hanako Shimura
Tsutomu Shimura
Byung-Cheul Shin
Chan Young Shin
Chong Shin
Dmitriy Shin
Dong Jin Shin
Dong Wook Shin
Eui-Cheol Shin
Eunsam Shin
H. Y. Shin
Hai-Rim Shin
Ho-Joon Shin
Hye-Won Shin
Hyun-Dong Shin
Hyung-Ik Shin
Hyun-Jin Shin
Jae-Won Shin
Jong-Yeon Shin
Jung Eun Shin
Kyung-Hoon Shin
Min Shin
Min-Hye Shin
Shyi-Jang Shin
Sung Jae Shin
Taehoon Shin
Teo Jeon Shin
Yong-Uk Shin
Young Kee Shin
Nicolas Shinada
Arti Shinde
Vishal Shinde
Matt Shinderman
Kenneth Shindler
Hideo Shindou
Prashant Shingate
Alexander Shinkov
Shlomo Shinnar
Cristiane Shinobu Mesquita
Mari Shinohara
Masakazu Shinohara
Russell Shinohara
Yasuaki Shinohara
Yoshiaki Shinohara
Haruo Shintaku
Yasushi Shintani
Takashi Shinzato
Tatsuo Shioda
Masakazu Shiota
Seiji Shiota
Kate Shipman
Pat Shipman
Anna Shipov
Nathan Shippee
Yuichiro Shirai
Atsushi Shiraishi
Yoshishiro Shiraiwa
Kyoko Shirakabe
Makoto Shiraki
Kenta Shirasawa
Koumei Shirasuna
Katsuhiro Shiratake
Mohd Shiratuddin
Sajjad Shirazi
Shayan Shirazian
Soraya Shirazi-Beechey

Abbas Shirinifard
Ronit Shiri-Sverdlov
Mark Shirley
Matthew Shirley
Yoshitsugu Shiro
Toshihiko Shiroishi
Nikolay Shirokikh
Eric Shiroma
C. Shirong
Yuichiro Shirota
Neelam Shirsat
Ben Shirt-Ediss
Michael Shirts
Andrey Shiryaev
Tetsuro Shishido
Kohei Shitara
Egor Shitikov
Sergey Shityakov
Ivy Shiue
Sruti Shiva
Chaitanya Shivade
Sisinthy Shivaji
Muthugounder Shivakumar
Amol Shivange
Binoy Shivanna
Velizar Shivarov
Kodthalu Shivashankara
Kalyanam Shivkumar
Ziiv Shkedy
Michael Shlipak
Maya Shmulevitz
Steve Shnyder
Zahra-Sadat Shobbar
Yehuda Shoenfeld
Kooresh Shoghi
Mizutaka Shogo
Menachem Shoham
Shahla Shojaei
Mohammad Shojafar
Ikuo Shoji
Mahsa Shokouhi
Shadi Shokralla
Babak Shokrani
Wuraola Shokunbi
Oluwafolahan Sholeye
Lynette Sholl
Sarah Shomstein
Addmore Shonhai
Suzanne Shontz
Douglas Shook
Natalie Shook
Steven Shoptaw
Soulmaz Shorakae
Anna Shore
Jennifer Shore
Kirsty Short
Sarah Short
James Shorter
John Shorter
David Shortle
Erin Shortlidge
Linda Shortliffe
Julie Shortridge
Varda Shoshan-Barmatz
Joseph Shostell
Huixia Shou
Scott Showalter
Timothy Showalter
Louise Showe
Carolin Showers
Michael Shoykhet
Elena Shpak
Nahum Shpigel
O.M. Prakash Shrama
Govinda Shrestha
Mani Shrestha
Milan Shrestha
Neha Shrestha
Manju Shri
Shailesh Shrikhande
Oren Shriki
Gireesh Shrimali
Mark Shrimpton
Shubham Shrivastava
Monal Shroff
Rachna Shroff
Martha Shrubsole
Louena Shtrepi
Jiang Shu
Jwu-Ching Shu
Longfei Shu
Shiyu Shu
Wenmiao Shu
Xiaokang Shu
Yongqian Shu
Xin Shuai
Ashfaq Shuaib
Nicholas Shubin
Brian Shuch
Kathryn Shuford
Holly Shugart
Yumin Shui

David Shuker
Alok Shukla
Animesh Shukla
Devesh Shukla
Dhananjay Shukla
Dinesh Shukla
Diwakar Shukla
Girja Shukla
Rakesh Shukla
Ravi Shukla
Sanjeev Shukla
Sourabh Shukla
Sudhanshu Shukla
Surendra Shukla
Peter Shull
Dorothy Shulman
Lee Shulman
Anderson Shum
Howard Shuman
Daniel Shumer
Martha Shumway
Shiow Ching Shun
Michael Shurin
S. Shushruth
Stephen Shuster
Dave Shutler
Timothy Shutt
Lori Shutter
Shade Shutters
Kristin Shutts
David Shveiky
Yu Shyr
Guangwei Si
Jianhua Si
Shuyi Si
Si Si
Tian-Mei Si
Elaine Sia
Spyridon Siakavellas
Ashfaq Sial
Cephas Sialubanje
Popluechai Siam
Miroslawa Siatecka
Baha Sibai
Euphemia L. Sibanda
Mncengeli Sibanda
Shannon Sibbald
Maria Sibilia
Nicholas Sibinga
Chris Sibley
Eric Sibley
Kathryn Sibley
Gustavo Sibona
Olivier Sibony
Delphine Sicard
Nadine Sicard
Rosa Sicari
Wen Sicheng
Richard Sicher
Frank Sicheri
Jason Sicklick
Cosmin Sicora
Nancy Sicotte
Elisa Sicuri
Anna Siczek
Katia Sidali
Rafat Siddiq
Kamran Siddiqi
Najma Siddiqi
Noman Siddiqi
Hifzur Siddique
Shahid Siddique
Imtiaz Siddiqui
M Rizwan Siddiqui
M. Siddiqui
Mustafa Siddiqui
Nazlee Siddiqui
Saad Siddiqui
Peter Sidebotham
John Sides
Venkataramana Sidhaye
Manbinder Sidhu
Pritam Sidhu
Samuel Sidi
Christos Sidiropoulos
Kiki Sidiropoulou
D. Sidjanin
Brian Sidlauskas
Christian Sidor
Olga Sidorova
Natalia Sidorovskaia
Ellen Sidransky
Sebastian Siebelmann
Anna Sieben
Alrun Siebenkäs
Kevin Siebenlist
Christian Siebenwirth
Fritz Sieber
Jessica Sieber
W. Karl Sieber
Christine Sieberg
Peer-Olaf Siebers

Stefan Siebert
Gary Sieck
Anna Maria Siega-Riz
David Siegel
Jason Siegel
Jeffrey Siegel
Martin Siegel
Paul Siegel
Deborah Siegele
Noam Siegelman
Bob Siegerink
Ingo Siegert
Kellee Siegfried
Claire-Anne Siegrist
Dan Siehl
Jakob Sieker
Daria Siekhaus
Evan Siemann
Salvatore Siena
Marcin Sienczyk
Emilio Siendones
Waldemar Sienkiewicz
Adam Siepielski
Florence Siepmann
Ulrich Siering
Frederick Sierles
Jeroen Siero
Jorge Sierra
Constantinos Siettos
Christina Siettou
Harri Sievanen
William Sievert
Roland Siezen
Volker Siffrin
Daniel Sifrim
Marco Sifringer
Jose Sifuentes-Osornio
Ian Sigal
David Sigalet
Dominique Sigaudo-Roussel
Erin M. Sigel
Robert Siggins
Sanne Sigh
Michael Sigler
Geraldina Signa
Tania Signal
Johannes Signer
Salvatore Signorelli
Santiago Signorelli
Rosario Signorello
Giuseppe Signoriello
Marcelo Signorini
Ólafía Sigurjónsdóttir
Smita Sihag
Pasi Sihvonen
Juha Siikamaki
Harri Siitari
Eric Sijbrands
Paul Sijens
Alice Sijts
Siddhartha Sikdar
Robert Sikes
Mile Šikic
Predrag Sikiric
Keith Siklenka
Aleksandra Sikora
Marianna Sikorska
Claudia Sikorski
Gaurav Sikri
Sverker Sikström
Parames Sil
Sheetal Silal
Philippe Silar
Lawrence Silbart
Ariel Silber
Jonathan J. Silberg
Dafne Magali Silberman
Rebecca Silbermann
Janet Silbernagel
Claudia Silberstein
Mark Silby
José Siles
Robert Siliciano
Giuliano Siligardi
Alba Silipo
Joan Silk
John Silke
Steffe Silke
Fernando Siller-López
Jolanta Siller-Matula
Christopher Silliman
Ian Sillitoe
Roy Sillitoe
Erin Sills
Miles Silman
Nigel Silman
Laura Silo-Suh
Adelino Silva
Adriana Silva
Alexandre Silva
Ana Silva
Ana Luisa Silva

Ana Sílvia C. Silva
Anabela Silva
Analiza Silva
André E. Silva
Andressa Silva
Carmen Silva
Catarina Silva
Claudia Silva
Cristiane Silva
Daniel Silva
Denise Silva
Emmanuel Silva
Erick Silva
Etel Silva
Fernando Silva
Francisco Silva
J. M. Silva
Joana Silva
João Silva
Jonathan Silva
Jonathas Silva
Kleber Silva
Lara Silva
Manuela Silva
Matthew Silva
Paula Silva
Pedro Silva
Ricardo Silva
Roberto Silva
Rodrigo Silva
Rosane Silva
Samuel Silva
Tatiane Silva
Vania Silva
Noelia Silva del Rio
Helio Tedesco Silva Jr.
Nelson Silva Jr.
Thiago Silva Paiva
Miguel Silva Vieira
Nicholas Silvaggi
Inacio Silva-Neto
Alessandro Silvani
Juha Silvanto
Cristina Silvar
Luz Silva-Torres
Mario Silva-Vergara
Fernando Silveira
Landulfo Silveira
Luis Silveira
Mariana Silveira
Thaís Silveira
Brian Silver
David Silver
Jonathan Silver
Lynn Silver
Robert Silver
Roxane Silver
Samuel Silver
Carlos Silvera Batista
N. Silverberg
M. Caterina Silveri
Karina Silverio
Anne Silverman
Jerald Silverman
Neal Silverman
Rachel A. Silverman
William Silverman
Ruth Silversmith
Douglas Silverstein
Peter Silverstein
Roy Silverstein
Jean-Sebastien Silvestre
Jean-Sébastien Silvestre
Francesca Silvestri
Rosalia Silvestri
Simone Silvestri
Franco Silvestris
Nicola Silvestris
Paul Silvia
Cheolho Sim
Julius Sim
Kelvin Sim
Leslie Sim
Sheina Sim
Valerie Sim
Anca Sima
Miguel Angel Simancas Pallares
J. Marc Simard
Martin Simard
Rosineide Simas
Leickness Simbayi
Dmitri Simberg
Sara Simblett
Charles Simenstad
Luis E. Simental-Mendia
Rune Simeonsson
Domagoj Simic
Filippo Simini
Laura Siminoff
Paul Simion
Dan Simionescu
Warren Simison

Peter Simkin
James Simkins
Douglas Simkiss
Myrtill Simko
Andreas Simm
Thomas Simmen
Karen Simmer
Mark Simmerman
Andrew Simmonds
Bethany Simmonds
Michael Simmonds
Benno Simmons
David Simmons
Graham Simmons
Mark Simmons
Vani Simmons
Victoria Simms
Davina Simoes
Elisabetrh Simoes
Fernando Simoes
Taynana Simões
Zilá Simões
Paulo Simões-Lopes
Anne Simon
Anne-Laure Simon
Christine Simon
Dan J. Simon
Daniel Simon
Marcelo Simon
Nicholas Simon
Raphael Simon
Scott Simon
Steffen Simon
Carlos Simón
Patricia Simon-Assman
Mauro Simonato
Noa Simon-Delso
Jonathan Simone
Lisa Simone
Martin Simoneau
Emilie Simoneau - Buessinger
Michael Simone-Finstrom
Ilaria Simonelli
Jane Simoni
Clémence Simonin
Anne Simonis
Frank Simonis
Andrean Simons
Cas Simons
Claudia Simons
Guus Simons
Johan Simons
Rachel Simons
Robert Simons
Javier Simón-Sánchez
Anne Simonsen
Ulf Simonsen
Uri Simonsohn
Thomas Simonson
Kristina Simonyan
Jose-Enrique Simo-Ten
Stefan Simovic
Colin Simpfendorfer
Alexander Simpson
Angela Simpson
Daniel Simpson
David Simpson
Elizabeth Simpson
Evan Simpson
Garth Simpson
Gavin Simpson
Ian Simpson
Julie Simpson
Kimberley Simpson
Kit Simpson
Nigel Simpson
Pippa Simpson
Scott Simpson
Shannon Simpson
Steven Simpson
Tracy Simpson
Emily Sims
Natalie Sims
Sunder Sims-Lucas
Bertha Simwaka
Emanuele Sinagra
Gale Sinatra
Andrew Sinclair
Bradley Sinclair
David Sinclair
Hannah Sinclair
Karin Sinclair
Lucas Sinclair
Robert Sinden
Sardar Sindhu
Shireen Sindi
Mikkel Sinding
Luca Sineo
Pawan Singal
Vasanth Singan
Barbara Singer
Dinah Singer

Joel Singer
Michael Singer
Mirko Singer
Steven Singer
A. Singh
Aadesh Singh
Abhishek Singh
Aditya Singh
Akhilesh Singh
Amareshwar Singh
Amaya Singh
Amit Singh
Amrita Singh
Anil Singh
Anirudh Singh
Anshuman Singh
Anup Singh
Ashok Singh
Baljinder Singh
Bhagat Singh
Bhupinder Pal Singh
Brahmanand Singh
Brajendra Singh
Dharmendra Singh
Dheer Singh
Dinesh Singh
Gopal Singh
Guramrit Singh
Gurmeet Singh
Gurmit Singh
Harminder Singh
Harpreet Singh
Himansha Singh
Inderpaul Singh
Ishwar Singh
Jay Shankar Singh
Jeff Singh
K.P. Singh
Karuna Singh
Kashmir Singh
Kavita Singh
Kiran Singh
Krishna Singh
Lakhan Singh
Lalit Singh
Lokesh K. Singh
Madhu Singh
Mahavir Singh
Mandeep Singh
Meera Singh
N.K. Singh
Nadia Singh
Nagendra Singh
Nandita Singh
Navneet Singh
Neeloo Singh
Nevil Singh
Om Singh
Palwinder Singh
Pomila Singh
R. Singh
Raj Kumar Singh
Rajan Singh
Rajesh Singh
Ramandeep Singh
Ramesh Singh
Ravi Singh
Reetu Singh
Ritu Singh
Sanjay Singh
Sanjeev Singh
Saranjit Singh
Sarman Singh
Satya P. Singh
Satyendra Singh
Shailza Singh
Shane Singh
Shashi Singh
Shiva Singh
Soudamani Singh
Sukhwinder Singh
Sunita Singh
Surender Singh
Surinder Singh
Surjit Singh
Tarkeshwar Singh
Vijai Singh
Vijay Singh
Vikas Singh
Vinayak Singh
Vineet Singh
Vir Singh
Yadvir Singh
Yogen Singh
Yogendra Singh
Amit Singhal
Aneesh Singhal
Pravin Singhal
Andrew Singleton
Henrik Singmann
Amit Sinha
Animesh Sinha

Ashish Sinha
Debasish Sinha
Gaurav Sinha
Palash Sinha
Rohit Sinha
Subarna Sinha
Sunil Sinha
Surajit Sinha
Uttam K. Sinha
Suprakas Sinha Ray
Abhijit Sinha Roy
Pritam Sinha Roy
Renato Sinico
Robert Sinkin
Dong Hyun Sinn
Daniel Sinnecker
Debora Sinner
Scott Sinnett
Frederic Sinniger
Herman Sintim
Tasnim Sinuff
Ut Na Sio
Wai Ting Siok
C. Sionean
George Siontis
Konstantinos Siontis
Matthias Sipiczki
Kalle Sipila
Petra Sipila
Detmer Sipkema
Ferenc Sipos
Peter Sipos
André Siqueira
Franciele Siqueira
Herbert Siqueira
Walters Siqueira
Johan Siqveland
Rossana Sirabella
Linda Siracusa
Jean-Claude Sirard
John Sirard
Marc-André Sirard
Larry Siref
Amna Sirelkhatim
Sema Sirin
Charalampos Siristatidis
Chukiat Sirivichayakul
Sunee Sirivichayakul
Niroshan Siriwaradena
Davud Sirjani
Martin Sirois
Patricia Sirois
Tarja Sironen
Luigi Sironi
Howard Sirotkin
Valerie Siroux
Daniel Sirtes
Cesare Sirtori
Gaetano Siscaro
Marco Sisignano
Dan Siskind
Zuzana Šišková
Susan Sisson
Andone Sistiaga
Ioannis Sitaras
Cassian Sitaru
Freddy Sitas
Karim Si-Tayeb
Folke Sitbon
Fortune Sithole
Colleen Sitlani
Stephanie Sitnick
Nadia Sitoe
Nádia Sitoe
Tilahun Sitote
Vinoth Sittaramane
Holly Sitters
Metin Sitti
Gilman Siu
Parco Siu
Timothy Siu
J. Siuda
Hari Sivakumar
M. Sivakumar
Rathinam Sivakumar
Yakov Sivan
Priya Sivaramakrishnan
William Sivitz
Victor Sivozhelezov
Justyna Siwy
Christophe Six
Payam Siyari
Stephane Sizonenko
Anders Sjalander
Matthias Sjerps
Virginie Sjoelund
Anna Sjörs
Agnes Sjöstrand
Christopher Sjöwall
Tea Skaaby
Eric Skaar
Todd Skaar

Debra Skafar
R. Skagarwal
Melissa Skala
Helen Skaletsky
Simon Skalicky
Natasa Skalko-Basnet
Alexios–Leandros Skaltsounis
Marion Skalweit
Spyridon Skandalis
Panagiotis Skandamis
Finn Skårderud
Serguei Skatchkov
Sheila Skeaff
Chris Skedgel
Richard Skeffington
Deanne Skelly
Peter Skelsey
James Skelton
Barbara Skerlavaj
Cristina Skert
David Skibbe
Karolina Skibicka
Randal Skidgel
Greg Skilleter
Anne Skinner
Daniel Skinner
Eila Skinner
Elizabeth Skinner
Jared Skinner
Tina Skinner
Lisa Skinner (Louth)
James Skipworth
Rolv Skjarven
Larry Sklar
Mihaela Skobe
Cezary Skobowiat
Steve Skoda
Erika Skoe
Nicole Skoetz
Krissa Skogen
Inger Marie Skogseid
Laila Skogstad
Nadine Skoluda
Karl Skorecki
Tomasz Skorski
Jacek Skorupski
Anna Skórzewska
Leif Skot
Jan Skotheim
Arthur Skoultchi
Vasileios Skouras
Rachid Skouta
Pernille Skovby
Knut Skovereng
Henrik Skovgaard
Gry Skovsted
Agnieszka Skowron
Marcin Skowron
Thomas Skripuletz
Isaac Skromne
Marko Skrtic
Jason Skues
Verena Skuk
Pavel Skums
Evgenii Skurikhin
Thomas Skurk
M. Skutsch
Pavel Skutschas
Vasilisa Skvortsova
Jerod Skyberg
Isabel Skypala
Andreas Skyschally
Mark Slabaugh
Catherine Slack
F.J. Slack
Marion Slack
Mary Slack
Susan Slade
Tim Slade
Frances Sladek
Susan Slager
Betty Slagle
Nada Slakeski
Laura Slane
Robert Slany
Jan Šlapeta
Hannah Slater
Mel Slater
Barton Slatko
Susan Slatyer
James Slaughter
Virginia Slaughter
Nikolai Slavov
Jean Slawinski
John Sled
William Sledge
Margaret Sleeper
Victoria Sleight
Zdenka Slejkovec
Vladlen Slepak
Michael L. Slepian
Halina Slesak

Paul Slesinger
Marit Sletmoen
Robert Slevc
Dennis Slice
Ilja Sligte
Mahmoud Slim
Rima Slim
Nadia Slimani
Craig Slingluff
Sarah A. Sliwa
Chantel Sloan
Derek Sloan
Frank Sloan
Luke Sloan
Richard Sloan
Victoria Sloan
Semyon Slobounov
Katie Slocombe
Alexander Slocum
Ivan Sloma
Steven Sloman
Lauren Slone
Michelle Slone
Lizeth Sloot
Arjen Slooter
Michel Slotman
Hans-Christian Slotved
Tina Slusher
David Slusky
Tamara Sluss
Ronald Sluyter
Laura Sly
William Sly
Laura Smale
Emily Smalheiser
David Small
Henry Small
Ian Small
Michael Small
Pamela Small
Randall Small
Rhonda Small
John Smalley
Richard Smalling
Younes Smani
Jonathan Smart
Leonard Smart
Maarten van Smeden
Fred Smedes
Annique Smeding
Damian Smedley
Jeremy Smedley
Bard Smedsrod
Timothy Smeeding
Pierre Smeesters
Jeroen Smeets
Berit Smestad Paulsen
Richard Smeyne
Jerusa Smid
William E. Smiddy
Nynke Smidt
Timo Smieszek
James Smiley
Jeff Smiley
Richard Smiley
Tierra Smiley Evans
Jennifer Smilowitz
Despina Smirlis
Oleg Smirnov
Alexandra Smirnova
Jesper Smit
Joost Smit
Lidwien Smit
Roelof Smit
Suzanne Smit
Suchi Smita
A. Peyton Smith
Aaron Smith
Abigail Smith
Adam Smith
Amber Smith
Amy Smith
Andra Smith
Andrew Smith
Andy Smith
Ashleigh Smith
Barry Smith
Ben Smith
Brett Smith
C. Smith
Carine Smith
Christiana Smith
Clyde Smith
Colin Smith
Courtland Smith
Craig Smith
Darci Smith
Dave Smith
David Smith
Donelson F Smith
Douglas Smith
Edvard Smith

Edward C. Smith
Edwin Smith
Eliot Smith
Eric Smith
Eric P. Smith
Fang Smith
George Smith
Gordon Smith
Gregory Smith
Jacqueline Smith
James Smith
Jeffrey Smith
Jennifer Smith
Jim Smith
Joe Smith
Joshua Smith
Justin Smith
Kathlyn Smith
Kenneth Smith
Kenny Smith
Kimberley Smith
Kirk Smith
Kyle Smith
Lee Smith
Lewis Smith
Lloyd Smith
M. Smith
Mark Smith
Martyn Smith
Matthew Smith
Michael Smith
Micholas Smith
Monica Smith
Noelle Smith
Pamela Smith
Patrick Smith
Peter Smith
Quentin Smith
R. Theodore Smith
Rebecca Smith
Richard Smith
Robert Smith
Rusty Smith
Sakima Smith
Sara Smith
Seth Smith
Shamus Smith
Shelley Smith
Sinead Smith
Stephanie Smith
Stephen Smith
Stephen M. Smith
Steve Smith
Steven Smith
Stuart Smith
Susan Smith
Terrence Smith
Thomas Smith
Toby Smith
Vicki Smith
Vincent Smith
Webb Smith
Yoland Smith
Zachary Smith
Zachary M. Smith
Laurel Smith Doerr
Claudia Smith Kelly
Rachel Smith-Bolton
Noah Smith-Drelich
Peter Smithers
Thomas Smithgall
Vania Smith-Oka
Alexa Smith-Osborne
Lauren Smith-Ramesh
Erica Smithwick
Alexander Smits
Anke Smits
Hermelijn Smits
Niels Smits
Sander Smits
John Smol
Albert Smolenski
Ryszard Smolenski
Patrick Smolinski
Bogdan Smolka
Georgiy Smolyakov
Abdelaziz Smouni
Alvin Smucker
Byran Smucker
Jason Smucny
Yvo Smulders
Nikolaos Smyrnis
Pavlo Smyrnov
Andrew Smyth
Davida Smyth
Susan Smyth
Erik Snapp
Michelle Sneck
Marcus Snell
Terry Snell
Brian Snelling
Sarah Snelling

Warren Snelling
Emilie Snell-Rood
Ban Sng
Nathan Sniadecki
Ashley Snider
Julia Snider
Natasha Snider
Allan Sniderman
Antoine Snijders
Soren Snitker
Joel Snodgrass
Christophe Snoeck
Féline Snoeck
Hager Snoussi
Allison Snow
Andrew Snow
Dean Snow
Jonathan Snow
Jessica Snowden
Charles Snowdon
Abraham Snyder
Bruce Snyder
Christopher Snyder
Hannah R. Snyder
Kenneth Snyder
Nathaniel Snyder
Wing Chee So
Louise Soanes
Aline Soares
Ana Soares
Dinesh Soares
Irene Soares
Marcelo Soares
Nelson Soares
Rodrigo Soares
Sandra Soares
Vasco Soares
Ruann Janser Soares de Castro
Rafael Soares Lindoso
Ricardo Soares Magalhaes
Britaldo Soares-Filho
Luisa Soares-Miranda
Armin Soave
Gungor Sobaci
Patricia Sobecky
Ken Sobel
Noam Sobel
Roy Soberman
Jorge Soberon
Mario Soberón
Patrick Sobetzko
Iradj Sobhani
S. Sobhani
Magdalena Sobieszczyk
Jason Soble
Alexander Sobolev
Stanislav Sobolevsky
Eric Sobolewski
Piotr Sobolewski
Jonathan Soboloff
Radoslaw Sobota
Gabriela Sobral
Rita Sobral
Raúl Sobrero
Lucia Sobrin
Francisco Sobrino
Luis Cristovão Sobrino Porto
Gen Sobue
Mike Socha
Eugenia Socias
Sanjeev Sockalingam
R. Sockett
Merav Socolovsky
R. Socorro
Silvia Socorro
Yao Sodahlon
Andrea Soddu
Daniel Soderberg
Cecilia Söderberg-Naucler
David Soderlund
Maria Söderlund-Venermo
Per Sodersten
Komal Sodhi
Nita Sodhi-Berry
Andrea Sodi
Regis Sodoyer
Johannes Soeding
Oliver Soehnlein
Kazutaka Soejima
Mathias Soergaard
Brian Soetikno
Yael Sofer
Michael Sofroniew
Tadashi Sofue
Jean-Jacques Soghomonian
Cristina Soguero-Ruiz
Debra Soh
Kwang-Sup Soh
Leen-Kiat Soh
Muhammad Sohaib
Muhammad Sohail Zafar
Zahra Sohani

Ratna Sohanpal
Christian Sohlenkamp
Chang Hwan Sohn
Seonghyang Sohn
Won Joon Sohn
Thomas Pave Sohnesen
Ediz Sohoglu
Sayed Sartaj Sohrab
Amir Sohrabi
Sara Sohr-Preston
Uri Soiberman
Eeva Soininen
Nancy Soja
Aleksandra Sojic
Samuel Sojinu
Dorothy Sojka
Jaap Sok
Robert Sokol
Arseny Sokolov
Sasha Sokolov
Milena Sokolowska
Malgorzata Sokolowska-Wojdylo
Katie Sokolowski
Marla Sokolowski
Evgeni Sokurenko
Carles Sola
Isabel Sola
Laura Sola
Zakaria Solaiman
Cuneyt Solak
Francisco Solano
David Solà-Oriol
Alessandra Solari
Fiorella Solari
Jan Helge Solbakk
Leah Solberg Woods
Petr Solc
Montse Sole
Floréal Solé
Majid Soleimani-damaneh
Cristina Solé-Padullés
Louis-George Soler
Xavier Soler
Zachary Soler
Javier Solera
Fernando Soler-Toscano
Gaye Soley
Mariano Soley-Guardia
Michele Solfrizzo
Halvor Solheim
Paolo Solidoro
Lisa Solieri
Elsayed Soliman
Hatem Soliman
Karam Soliman
Sameh Soliman
Michele Solimena
Kiran K Solingapuram Sai
Marc Solioz
Ivonne Solis-Trapala
David Soll
Jürgen Soll
Dieter Söll
Salvatore Sollima
Rahel Sollmann
Marco Solmi
Andrea Solnes Miltenburg
Jay Solnick
Razafimahefa Solofoniaina
Olga Solomina
Daniel Solomon
George Marty Solomon
Jeffrey Solomon
Jonathan Solomon
Joshua Solomon
Justin Solomon
Mark Solomon
Sorin Solomon
Thomas Solomon
Zahava Solomon
Tessa Solomon-Lane
Alicia Solorzano
Caius Solovan
Ivan Solovic
Andrew Solow
John Solow
Trygve Solstad
Laura Solt
Ali Reza Soltanian
Joseph Soltis
Alec Solway
Sudipta Som
Biju Soman
Arun Somani
Agila Somasundaram
Kumaravel Somasundaram
Kunlaya Somboonwiwat
George Somero
Annemie Somers
Erkki Somersalo
Shawn Somerset
Nami Someya

Tamás Somfai
Daniele Sommaggio
Christine Sommer
Denise Sommer
Gunhild Sommer
Natascha Sommer
Nicolas Sommer
Rolf Sommer
Simone Sommer
Ulrich Sommer
Werner Sommer
Olaf Sommerburg
Mark Sommerfeld
Jessica Sommerville
Beatriz Somoza
Gustavo Somoza
Hokyoung Son
Seung-Woo Son
Humira Sonah
Maria de Fatima Sonati
Avinash Sonawane
Kailas Sonawane
Fernando Soncini
Rakesh Sondekoppam
Hideko Sone
Teruo Sone
Toshimasa Sone
Manish Soneja
Samir Soneji
Sharon Sonenblum
Abraham Sonenshein
Charlotte Soneson
Beng Kah Song
Bo Song
Brian Song
Byeongwoon Song
Changcheng Song
Chang-Seon Song
Chang-Xu Song
Chunhua Song
Dong Song
Fengming Song
Gaoyuan Song
Guili Song
GuoHua Song
Guoli Song
Haengseok Song
Hai-Tao Song
Hee Kwon Song
Ho-Chun Song
Houhui Song
Huan Song
Hyohak Song
Hyun Seok Song
Jae Kwang Song
Je Seon Song
Jianmin Song
Jianxun Song
Jiasheng Song
Jie Song
Jikui Song
Jim Song
Jingyuan Song
Jinlin Song
Jiuzhou Song
Juan Song
Jun Song
Ki Jun Song
Kwan-Jeong Song
Lei Song
Li-Peng Song
Lixin Song
Long-Sheng Song
Min Song
Mina Song
Ming Song
Mingyang Song
Moshi Song
Rhayun Song
Se Jin Song
Shaojuan Song
Shujie Song
Shumei Song
Si Yeol Song
Song-Quan Song
Soon Song
Tao Song
Tian Song
Wei Song
Weihua Song
Wen-Jie Song
Wenxia Song
Woo Keun Song
Xiao-Yan Song
Xuemei Song
Yajun Song
Yan Song
Yanzhi Song
Yao-Bin Song
Yougui Song
Yufang Song
Yun Seon Song

Yun-Mi Song
Zhaobin Song
Zhaoliang Song
Zhen Song
Zhenyuan Song
Zhiwei Song
Marco Songini
Gautam Soni
Sanjeev Soni
M. A. Sonibare
Stephen Sonis
Milan Sonka
Geoffrey Sonn
Joshua Sonnen
Anton Sonnenberg
Caroline Sonnenberg
Tim Sonntag
Kenzo Sonoda
Masahiro Sonoo
Doron Sonsino
Chia Soo
Po-Chi Soo
Akshay Sood
Gaurav Sood
Rashmi Sood
Suman Sood
E. Soodi
Sunhapas Soodvilai
Mehdi Sookhak
Chun Siong Soon
Villu Soon
Boonchoy Soontornworajit
John Soper
Alex Sopilniak
Mohan Sopori
Alberto Sorace
Atilla Soran
Gianni Soraru
Alma Sörberg
Ingvil K. Sørbye
Sveinung Sørbye
Regina Sordi
Isabella Soreca
Kjetil Søreide
Larsen Soren Thor
Robert Soreng
Glorian Sorensen
Jens Laurids Sørensen
Karen Sørensen
Line Sørensen
Michael Sørensen
Uffe Sørensen
Olav Sorenson
Hermona Soreq
Marie Soressi
Christian Sorg
Joseph Sorg
Rüdiger Sorg
Robert Sorge
Antonio Sorgente
Edgar Soria-Gomez
Maria Eugenia Soriano
Miguel Soriano
Alberto Soriano-Maldonado
Audrey Soric
Maurizio Sorice
Emanuele Soricelli
Victoria Sork
Maria Pia Sormani
Liliana Soroceanu
Stuart Soroka
Andrey Sorokin
Shahryar Sorooshian
Patrik Sörqvist
Michael Sorrell
Trevor Sorrells
Brian Sorrentino
Dario Sorrentino
Mohamed Sorror
Carlos Oscar Sorzano
Aaron Sosa
Modesto Sosa
Victoria Sosa
Bolivar Sosa Madrid
Sergio Sosa-Estani
Louis Soslowsky
Gabriel Sosne
Philippe Sosner
Tobin Sosnick
Teiji Sota
Ana Sotelo
Julio Sotelo
Elpidoforos Soteriades
Cibele Sotero-Caio
Demetris Soteropoulos
Ioanis Sotiroppoulos
George Sotitiou
Ilka Sötje
Axel Soto
Ignacio Soto
Jose Luis Soto
Lilian Soto

Luis Soto
Patricia Soto
Yudira Soto
Jose Soto Shoender
Benito Soto-Blanco
Alejandro Soto-Gutierrez
David Soto-Pantoja
Ricardo Soto-Rifo
Daniela Sotres-Alvarez
Lina Soualmia
Sébastien Soubeyrand
Pavel Soucek
Céline Souchay
Isabelle Souchon
Nadejda A. Soudzilovskaia
Jiahen Soueid
Assem Soueidan
Nafaa Souissi
Alexander Soukas
Bernardo Souki
Tomas Soukup
Nicholas Soulakis
Pauline Soulas-Sprauel
Eric Soule
Anaelle Soulebeau
Kyriakos Souliotis
Carl Soulsbury
Panos Soultanas
Vassili Soumelis
Pierre Jean Souquet
Sandrine Souquière
Carole Sourbier
Harald Sourij
Victor Sourjik
Ana Sousa
Daniela Sousa
Diana Sousa
Fani Sousa
Filipa Sousa
Magda Sousa
Maria Sousa
Maria João Sousa
Nuno Sousa
Paulo Sousa
Sofia Sousa
Tais Sousa
Carlos José Sousa Passos
Marta Sousa Silva
Renata Sousa-Lima
Rita Sousa-Nunes
Michael Soussan
Laurent Soustelle
Andrew South
Kieron South
Mikle South
Brandon Southall
Theresa Southard
Michelle Southard-Smith
Brian Southern
Melissa Southey
Jennifer Southgate
Bridget Southwell
André Souto
David Souto
Fabricio Souto
Olga Soutourina
Michele Souyri
Alexandre Navarro Alves Souza
Cleverson Souza
Danielle Souza
Givago Souza
Renan Souza
Rhonda F. Souza
Silvia Souza
Tharsis Souza
Carlos Souza Jr.
Jayme Souza-Neto
Tamás Sovány
Inga Soveri
Simona Soverini
Mikhail Sovershaev
Marianne Sowa
Wojciech Sowa
Paul Sowden
Richard Sowell
James Sowers
Pawel Sowinski
Joseph Sowka
David Soybel
Philippe Soyer
Yesim Soyer
Soner Soylu
Efe Soyman
Floor Spaans
Roberta Spaccapelo
Erica Spackman
Teresa Spadea
Oliver Spadiut
Christine Spadola
Andrea Spaeth
Jason Spaeth
Johannes Spaethe

Sean T. Spagnoli
Matteo Spagnolo
Paolo Spagnolo
Heiko Spallek
Gianfranco Spalletta
Giampiero Spalluto
Claudia Spampinato
Paul Span
Rainer Spang
Robert Spang
Jo Spangaro
Michael Spannagl
Anthony Spano
Giuseppe Spano
Michiel Spape
Herbert Spapen
Joseph Sparano
Tim Sparer
Debbie Sparkes
Adam Sparks
Fraser Sparks
Jackson Sparks
Jeffrey Sparks
Matthew Sparks
Francesca Sparla
Janet Sparrow
Dennis Sparta
Nicole Spartano
Sabrina Spatari
Peter Spath
Michelle Spaulding
Sarah Spaulding
C. Wendy Spearman
Silvia Speca
Stuart Spechler
Hanno Specht
Karsten Specht
Oliver Speck
Anne Speckens
Alan C. Spector
Andrew Spector
Arthur Spector
Logan Spector
Reinhart Speeckaert
Doug Speed
Corey Speers
Ben Speers-Roesch
Branka Spehar
David Speicher
William Speier
Douglas Speirs
Phyllis Speiser
Guenter Speit
Ilene Speizer
H. Spelbrink
Frank Speleman
Camilla Speller
Tim Spelman
Andrew Spence
Caroline Spence
Ian Spence
Marsha Spence
C. Spencer
David Spencer
Kate Spencer
Lisa Spencer
Sarah Spencer
Thomas Spencer
Megan Spencer-Smith
Lindsay Spender
Jessica Spengler
Felipe Sperandio
Irene Sperandio
Alessandra Sperduti
Jonathan Spergel
Billy Sperlich
Dominik Sperlich
Or Sperling
Silke Sperling
Ann Sperry
Jason Sperry
Paola Spessotto
Karina Speziale
James Spicer
Robert Spicer
Timothy Spicer
Corinne Spickett
David Spiegel
Martin Spiegel
Kai Spiegelhalder
Bruce Spiegelman
Edda Spiekerkoetter
Tobias Spielman
Wolfgang Spielmeyer
Lucas Spierer
Donald Spiers
Claudia Spies
Laure Spieser
Alexander Spiess
Andrej-Nikolai Spiess
Ewa Spiesz
Peter Spieth

Ewa Spiez
Enrico Spiga
Anne Spijker
Sabine Spijker
Annemieke Spijkerman
Brad Spiller
Celsa Spina
Dominik Spinczyk
Jennifer Spindel
Sherry Spinelli
Davide Spinello
Jennifer Spinler
Sarah Spinler
Rubens Spin-Neto
Michael Spiotto
Bruno Spire
Carlo Spirlì
Andrea Spitaleri
Pietro Spitali
Grazia Spitoni
Jan Spitsbergen
B. Spittau
Alicia Spittle
Andreas Spittler
Mark Spitz
Martin Spitzer
Marla Spivak
Marcia Spoelder
Eberhard Spoerl
Ana Maria Spohr
Robert Spooner
Jennifer Spoor
Victor Spoormaker
Michael Sporn
Peter Sporns
Silvia Spoto
Glen Spraggon
Jennifer Sprague
Stuart Sprague
Brian Spratt
Heidi Spratt
David Spray
Armand Sprecher
Simon Sprecher
Ole Sprengeler
Andreas Sprenger
Cynthia Sprenger
Jose Spricigo
Mathieu Spriet
David Spring
Michele Spring
Stefan Spring
Matthew Springer
Sandra Springer
Georg Sprinzl
Poli Mara Spritzer
Daniel Sprockett
Henri Spronk
Emma Sprooten
John Sproule
Daisy Sproviero
William Sproviero
Karen Spruyt
Elizabeth Spry
Anne Spurkland
Greg Spyreas
Ioakim Spyridopoulos
Cristiane Squarize
Andrea Squartini
Nicola Squillace
Fabio Squina
Christopher Squire
Iain Squire
Allison Squires
Alessandro Squizzato
Andre Sradnick
S. Sreelatha
Chandrashekhar Sreeramareddy
Sargur Srideshikan
Jayalakshmi Sridhar
Saranya Sridhar
T.S. Sridhar
Vijayalakshmi Sridharan
Aparna Srikantam
Preethi Srikanthan
Anon Srikiatkhachorn
Kornsorn Srikulnath
Shabarinath Srikumar
Govindarajan Srimathveeravalli
Mangala Srinivas
Miduturu Srinivas
R. Srinivas
S. Srinivas
Babji Srinivasan
Gokul Raj Srinivasan
Mahesh Srinivasan
Manoj Srinivasan
Mythily Srinivasan
Prakash Srinivasan
Rajagopalan Srinivasan
Rajagopalbabu Srinivasan
Ramaprasad Srinivasan

Ramprakash Srinivasan
Shanthi Srinivasan
Subashini Srinivasan
Varun Srinivasan
Hao Srinivasasetty
Rebecca Sripada
Hutcha Sriplung
Nattachai Srisawat
Shiranee Sriskandan
Hathaitip Sritanaudomchai
A. Srivastava
Ajay Srivastava
Amit Srivastava
Anuj Srivastava
Aradhana Srivastava
Arunima Srivastava
Bhupendra Srivastava
Disha Srivastava
Kamna Srivastava
Kshitij Srivastava
Nisheeth Srivastava
Nishi Srivastava
Om Srivastava
Pankaj Srivastava
Pranay Srivastava
Prashant Srivastava
Pratap Srivastava
Preeti Srivastava
Priyanka Srivastava
Rohit Srivastava
Sanjay Srivastava
Saurabh Srivastava
Sudhakar Srivastava
Tulisdas G. Srivastava
Arjun Srivathsa
Eri Srivatsan
Franz St John
James St John
Colleen St. Clair
Samuel St. Clair
Melissa St. Hilaire
Franz St. John
Justin St. John
Maie St. John
Martin St. Maurice
Claudia Staab-Weijnitz
J. Staal
E.V. Stabb
Judith Stabel
Sarah Stabenfeldt
Daniel Stabler
Gary Stacey
Natasha Stacey
Rhodri Stacey
Richard Brandon Stacey
William Stacey
Shawn Stachel
Peter Stachon
John Stachowicz
Austin Stack
Sharon Stack
Steven Stack
Robert Stackman
Samantha Staddon
Daniel Stadlbauer
Vanessa Stadlbauer
Krisztian Stadler
Marc Stadler
Zsofia Stadler
Andrew Stadnyk
Peter Staeheli
Bart Staels
An Staes
Anne Cathrine Staff
Nathan Staff
James Stafford
Lorenzo Stafford
Randall Stafford
Thorsten Stafforst
Alex Stagnaro-Green
Rita Stagni
Dave Stahl
Frank Stahl
Karl Stahl
Peter Stahl
Karl Ståhl
Karen Stahlheber
Amanda Staiano
Henry Staines
Richard Staines
Joseph Stains
Douglas Stairs
Kate Stalin
Guenter Stalla
Christopher Stallings
Sericea Stallings-Smith
David Stallknecht
Cecilia Stalsby Lundborg
Jonny St-Amand
Zania Stamataki
K. Stamatelopoulos
Nikiforos Stamatiadis

Dimitrios Stamatialis
Iva Stamatova
Hana Štambergová
Vuk Stambolic
W. Daniel Stamer
James Stamey
Lola Stamm
David Stammers
Michael Stamos
Dimitrios Stamovlasis
Dimitrios Stampoulis
G.J.J.M. Stams
Gheorghe Stan
Radu Stan
Daniela Stan Raicu
Matthew Stanbrook
Michael Stanchina
Holly Standing
Marie Standl
David Stanek
Craig Stanford
Kim Stanford
Espen Stang
Claudia Stange
Biljana Stangeland
Paula Stangeland
Catherine Stanger
Gabriele Stangl
Phillip Staniczenko
Juraj Stanik
Lee Stanish
Branislav Stankovic
Goran Stankovic
Milan S. Stankovic
Slaviša Stankovic
Theodore Stankowich
Dara Stanley
Dragana Stanley
E. Stanley
Edouard Stanley
Jeffrey Stanley
Jenni Stanley
Jennifer Stanley
Joanna Stanley
Kenneth Stanley
Margaret Stanley
Pamela Stanley
George Stanley Jr.
Nicola Stanley-Wall
Mike Stannett
Pasquale Stano
Maja Stanojevic
Sanja Stanojevic
Olivera Stanojlovic
Stephen Stansfeld
Brian Stansfield
Philip Stansly
Giorgio Stanta
Christina Stantis
Cynthia Stanton
Mary Ellen Stanton
Patric Stanton
Richard Stanton
Robert Stanton
Tasha Stanton
Peter Stanwell
Christina Malmose Stapelfeldt
Ann Stapleton
Jack Stapleton
Kristina Star
John Starbuck
Katrin Starcke
Zenon Starcuk
George Stark
Jeremy Stark
Ken Stark
Michael Stark
Renee Stark
Wendelin Stark
Jeffrey Starke
Robert Starke
Brooke Starkoff
Melissa Starling
Binil Starly
René St-Arnaud
Joseph Starobin
John Starr
Richard Starr
Timothy Starr
Carla Startin
C. Stasi
Georgios Stathopoulos
Athanasia Stathopoulou
Hakan Statin
Meir Statman
Christine Stauber
Tobias Stauber
Roland Staud
Dawid Staudacher
Heidi Staudacher
John Staudenmayer
Glenn Stauffer

Natalie Stauffer
S. Staunton
Doekele Stavenga
Stavros Stavrakis
Andreas Stavropoulos
Charitini Stavropoulou
Philipp Stawowy
Leslie Stayner
Lindsay Stead
Lucy Stead
Michael Stear
Deborah Stearns-Kurosawa
Simon Stebbings
Charles Stebbins
Patrizia Steca
Carla Stecco
Todd Steck
G. Christopher Stecker
Sophie Steculorum
Jonathon Stecyk
Robert Stedtfeld
Jennifer Steeb
Douglas Steeber
Clemens Steegborn
Kim Steegen
Thomas Steeger
John Steel
Karen Steel
Kylie Steel
Laura Steel
Charles Steele
Derek Steele
Douglas Steele
Jennifer Steele
Katherine Steele
Kelly Steele
Ric Steele
Vaughn Steele
Toddi Steelman
Andrew Steen
Christel Steen
R. Grant Steen
Richard Steen
Virginia Steen
Marco Steenbergen
Maria Steenhof
Leonie Steenis
Ivan Steenstra
Eefje Steenvoorden
Robin Steenweg
Norbert Stefan
Irene Stefanaki
Paola Stefanelli
Renae Stefanetti
Alessandro Stefani
Giovanni Stefani
Stefania Stefani
Anthony Stefanidis
Giulio Stefanini
Michael Stefanone
Spiro Stefanou
Detelin Stefanov
Lydia Stefanova
Vedran Stefanovic
Darko Stefanovski
Justyna Stefanowicz-Hajduk
Claudia Stefanutti
Annika Steffen
Imke Steffen
Daniel Steffens
Michal Steffl
Denes Steffler
Linda Steg
Johannes Stegbauer
Elizabeth Stegemoller
Alexander Stegh
Matthias Steglich
Johannes Stegmaier
Georgina Stegmayer
David Stegner
Elke Stehfest
Jörg Stehle
Bertine Stehouwer
Juan Steibel
Hans Steiger
Katrina Steiling
Viktor Steimle
Ariel Stein
Aryeh Stein
Daniel Stein
Eric Stein
H.H. Stein
Jeff Stein
Mark Stein
Michael Ashley Stein
Phyllis Stein
Richard Stein
Timo Stein
Ulrike Stein
J.H. Steinbach
Joseph Steinbach
William Steinbach

Martin Steinbauer
Katherine Steinbeck
Benjamin Steinberg
Bettie Steinberg
E.L. Steinberg
Fabian Steinberg
Gregory Steinberg
Sergey Steinberg
Yosef Steinberger
Michael Steinborn
Holger Steinbrenner
Daniel Steinemann
Alexandre Steiner
Florian Steiner
Peter Steiner
Ruth Steiner
S. Steiner
Sebastian Steinfartz
Joanna Steinglass
Helen Steingroever
Laura Steinhardt
Yael Steinhart
Stuart Steinhauer
Hans-Christoph Steinhausen
Nanette Steinle
Ortrud Steinlein
Alan Steinman
Lawrence Steinman
Eike Steinmann
Andreas Steinmayr
Hanspeter Steinmetz
Oliver Steinmetz
Robert Steinmetz
Jürgen Steinmeyer
Silje Steinsbekk
Paschalis Steiropoulos
Joanne Stekler
Michael Stekoll
Lukasz Stelinski
Giulia Stella
Lorenzo Stella
Pieter Stella
Giovanni Stellato
Michael Stellefson
Jeanne Stellman
J.P. Stellmann
Konstantinos Stellos
Marta Stelmach-Mardas
Klaus Stelter
Lars Stemmann
Olle Stendahl
Steen Stender
Lars C. Stene
Bo Stenerlöw
Andreas Stengel
Dagmar Stengel
Drake Stenger
Steffen Stenger
Mark Stenglein
Iain Stenhouse
Neil Stenhouse
Deborah Stenkamp
Marcus Stensmyr
Jørgen Stenvik
Werner Stenzel
Martin Stepan
Ondrej Stepanek
Ruben Stepanyan
Holger Stephan
Kurt Stephan
Paula Stephan
François Stéphan
Hua Stephane
Olof Stephansson
Dimity Maree Stephen
Julia Stephen
Francis Stephens
Jaclyn Stephens
Jacqueline Stephens
Patrick Stephens
Robin Stephens
Scott Stephens
Alisa Stephens-Shieldsa
Susan Stepney
Mary Ann Stepp
Elisabeth Sterck
Vicky Stergiopoulos
Aspasia Sterioti
Julie Sterling
Anat Stern
Elsbeth Stern
Judy Stern
Marc Stern
Paula Stern
Dagmar Sternad
Claus Sternberg
Jared Sterneckert
R. Sternglanz
Richard Sterns
Gaetana Sterrantino
Silke Stertz
Torsten Sterzenbach

Michael Sterzik
Julia Steuber
Ralf Steuer
Bas Steunenberg
Lotte Steuten
Annelies Stevaert
Lara Stevanato
Dejan Stevanovic
Tariq Stevart
Sharon Stevelink
Daniel Steven
Alexander Stevens
Charles Stevens
Gretchen Stevens
H. Stevens
Hanna Stevens
Jens Stevens
Kim Stevens
Matthew Stevens
Nathaniel Stevens
Tim Stevens
Vanessa Stevens
Warren Stevens
Jennifer Stevens Aubrey
Stanley M. Stevens Jr.
Andrew Stevenson
Grace Stevenson
Mark Stevenson
Mary Stevenson
Robert Stevenson
Michael Steward
Trevor Steward
Alexander Stewart
Alexandre Stewart
Andrew Stewart
Bryan Stewart
Cameron Stewart
Caroline Stewart
Christopher Stewart
Clinton Stewart
Dan Stewart
Donald Stewart
Donna Stewart
Douglas Stewart
George Stewart
Grace Stewart
Jane Stewart
Jennifer Stewart
Jeremy Stewart
Jill Stewart
Joanna Stewart
Laura Stewart
Mark Stewart
Matthew Stewart
Meredith Stewart
Orion Stewart
Phil Stewart
Philip Stewart
Ralph Stewart
Robert Stewart
Russell J. Stewart
Ryan Stewart
Sheila Stewart
Sherry Stewart
Simon Stewart
Tom Stewart
C. Neal Stewart Jr.
Arnaud Steyaert
Nelia Steyn
Herwig Stibor
M Stiborova
Abigail Stickford
Michael Stickland
Peter Stief
Bruno Stieger
Stefan Stieger
Lars Helge Stien
Ger Stienen
Marc Stift
Arwen Stikvoort
Bangyan Stiles
Brad Stiles
David Stillwell
James Stinear
Timothy Stinear
Francesco Stingo
Sandra Stinnett
John Stins
Monique Stins
Alin Stirban
Tom Stiris
Wendy A. Stirk
Michael Stirratt
Karyn Stitzenberg
Kelly Stiver
Anthony Stobbe
Maciej Stobiecki
Matteo Stocchero
Andrea Stocco
Ann-Kathrin Stock
Eileen Stock
Emmelie Stock

Jennifer Stock
Konrad Stock
Patricia Stock
Laura Stockdale
Reinhold Stockenhuber
Kurt Stocker
Martin Stocker
Karen Stockin
Brigitta Stockinger
Rachel Stockley
Jacqueline Stöckli
Anders Stockmarr
Thomas Stockner
Susan Stocks
Melissa Stockwell
Timothy Stockwell
David Stocum
Barry Stoddard
Frederick Stoddard
Kathleen Stoddart
Barbara Stoecker
Mark Stoeckle
Esther Stoeckli
Andrew Stoehr
Nicole Stoesser
Gijsbert Stoet
John Stoffel
Michael Stoffel
Thomas Stoffregen
Sabine Stöhr
Milan Stoiljkovic
Jure Stojan
Dejan Stojanovic
Diana Stojanovski
Chris Stojanowski
Jane Stojkov
Kathryn Stok
Andrew Stoker
Alexia Stokes
Ian Stokes
Karen Stokes
Tim Stokes
Runar Stokke
Aaron Stoler
Elly Stolk
Gautier Stoll
Kathrin Stoll
Moritz Stolla
Michael Stoller
David Stoltenberg
Regina Stoltenburg
Rebecca Stoltzfus
John Stolz
Henning Stolze
Elijah Stommel
Martin Stommel
Brandee Stone
Clement Stone
David Stone
James Stone
Jeffrey Stone
Neil Stone
Nelson Stone
Trevor Stone
Nicola Stonehouse
Mark Stoneking
Bradley Stoner
Cynthia Stonnington
Ruedi Stoop
Mark Stopfer
Christian Stoppe
Justin Storbeck
Kai-Florian Storch
Gilles Storelli
Helen Storey
Erik Storholm
Jonathan Storkey
Walter Storkus
Curt Storlazzi
Gary Stormo
Robert Storms
Mariano Stornaiuolo
Jorge Storniolo
Kate Storrs
Brad Story
Chandra Story
Michael Story
Christina Stothard
Paul Stothard
Clifford Stott
Philip Stott
Shannon Stott
Jens Stougaard
Daniel Stout
Dietrich Stout
Jake Stout
Jason Stout
Julie Stout
Jose Stoute
Christophe Stove
Cordula Stover
John Stover

Ann Stowe
James Stowe
Julia Stowe
Dan Stowell
Michael Stowell
Tanya Stoyanova
Eric D. Strachan
Giovanni Stracquadanio
Lucia Strader
Julia Strahl
Jennifer Strahle
Jana Strahler
Dietmar Straile
Tracey Straker
Diana Stralberg
Daniel Stram
Sebastiano Stramaglia
Bjørn Heine Strand
Douglas Strand
Michael Strand
Susanne Strand
Tor Strand
Sara Strandberg
Timo Strandberg
Ismo Strandén
Björn Strander
Manuela Straneo
Eric Sträng
Bryan Strange
Johan Stranne
Michael Strano
Janette Strasburger
Roger Strasser
Beverly Strassmann
Piergiorgio Strata
Gordon Strathdee
Andreas Straub
Timothy Straub
Volko Straub
Eberhard Straube
Sebastian Straube
Robert Straubinger
Ulrich Strauch
Sharon Straus
Claudia Strauss
Ewa Strauss
J. Rudi Strauss
Johannes Strauss
Phyllis Strauss
Roland Strauss
Stephen Stray
Birgit Streamer
Jan-Kolja Strecker
Robert Strecker
Charles Streckfus
Richard Street
Elizabeth Streicher
Jeffrey Streicher
Daniel Streicker
David Streiner
Yulia Strekalova
Jens Strelau
Petr Strelkov
Gillian Stresman
Paul Stretesky
Pasquale Striano
Brent Strickland
Dudley Strickland
Matthew Strickland
J. Rudi Strickler
James Strickler
Dan Strickman
Christopher Striemer
Gustav Strijkers
Kristof Strijkers
Arunas Strike
Rob Striker
Catherine Striley
Pontus Strimling
Samantha Strindberg
Jeffrey Stringer
Kathleen Stringer
Silvia Stringhini
Rebecca Stringwell
Caterina Strisciuglio
Franc Strle
Anja Strobel
Henry Strobel
Oliver Strobel
Enrico Strocchi
Todd Strochlic
Nathalie Stroeymeyt
Ruslan Strogantsev
David Strogatz
Albrecht Stroh
Kingman Strohl
Erhard Strohm
Jeff Strohm
Nina Strohminger
Sara Strom
Stephen Strom
Staffan Stromblad

Birgit Strommenger
Astrid Stronen
Daniel Strongin
Karien Stronks
Donna Stroup
Marc Strous
Roy Strowd
Thomas Strowitzki
Mathias Strowski
Claudio Struchiner
M.F. Struck
Eva Strucken
Victoria Struckmann
Tobias Struffert
Richard Strugnell
Erik Štrumbelj
Daniel Strunk
Julia Strupp
Paul Strutton
Sofie Struyf
Artur Struzik
Sarah Strycharz-Glaven
Rafal Stryjek
Michael Stryker
Adam Strzelczyk
Robert Strzodka
Amy Stuart
Andrew Stuart
Barbara Stuart
Patrick Stuart
Sam Stuart
Rachel Stubbington
Francesca Stubbins
Ben Stubbs
Brendon Stubbs
Kent Jason Stuber
Timo Stübig
Lucie Stuchlíková
Heiko Stuckas
Deborah Studer
Joseph Studer
David Studholme
James Studnicki
Eva E. Stüeken
Snorre Stuen
Wolfgang Stuerzl
Bruno Stuhlmüller
Therese Stukel
Debra Stulberg
Jorg Stulke
Richard Stumpf
Jason Stumpff
Ottokar Stundner
Robert Stupar
Mihai-Sorin Stupariu
Liborio Stuppia
Enrico Sturaro
Melissa Sturge-Apple
John A. Sturgeon
Erich Sturgis
James Sturgis
Mark Sturme
Edward Sturrock
Hugh Sturrock
Marc Sturrock
Franck Sturtz
Bridget Stutchbury
Maik Stüttgen
Jack Stutts
Rebecca Stutz
Nico Stuurman
Olaf Stüve
Besiki Stvilia
Craig Styan
Mark Styczynski
Triantafyllos Stylianopoulos
Alison Styring
Amy Styring
A.T. Su
Anselm Ting Su
B. Su
Bin Su
Bo-Han Su
Chen-Ming Su
Chi-Ting Su
Chun-Li Su
Diansan Su
Emily Chia-Yu Su
Fengtao Su
Huabo Su
Huanxing Su
Jen-Liang Su
Jianguo Su
Jianzhong Su
Kang-Cheng Su
Kang-Yi Su
Lei Su
Lianghu Su
Li-Chen Su
Longfei Su
Lydia Su
Ming-Wei Su

Qi Su
Riqi Su
Shaoyong Su
Shihbin Su
Songkun Su
Sui-Lung Su
Ting-Shi Su
Tom Su
Tung-Hung Su
Vincent Yi-Fong Su
W. R. Su
Wei-Wen Su
Wenqing Su
Winnie Su
Xiaosi Su
Yewang Su
Yi-Hsien Su
Yin Su
Yirong Su
Yong Su
Yu-Chung Su
Zheng Su
Zhengchang Su
Zhengquan Su
Zheng-Yuan Su
I. Wayan Suardana
Henry Suarez
Pablo Suarez
Scott Suarez
Sebastian Suarez
Juan Suárez
Maria Suarez Diez
Brian Suarez Mantilla
Abel Suárez-Fueyo
Marcela Suarez-Rubio
Eva Suarthama
Jose Suazo
Amanda Subalusky
Angela Subauste
Ramnath Subbaraman
Shaila Subbarao
Vignesh Subbian
Sergei Subbotin
Lovro Šubelj
Yousif Subhi
Claudia Subic-Wrana
Damien Subit
T. Subkhankul
Marion Subklewe
Palani Subramani
Bala Subramaniam
Dharmalingam Subramaniam
Mythily Subramaniam
Renuka Subramaniam
Ahalya Subramanian
Ramaswamy Subramanian
Senthil Subramanian
Shivashankar Subramanian
Subbaya Subramanian
Vijay Subramanian
Bhadriraju Subramanyam
Meena Subramanyam
S.H. Subramony
Jose Such
Malini Suchak
Boris Suchan
Robert Suchland
Nancy Suchman
Jordan Suchow
Philippe Sucosky
Goki Suda
Takashi Suda
Mateus Sudano
Vidya Sudarshan
Mysore Sudarshana
Sonja Sudarski
Palakolanu Sudhakar Reddy
M. Sudhakara Reddy
Babu Sudhamalla
G. Sudhandiran
Hideki Sudo
Ryo Sudo
Shih-Che Sue
Viranuj Sueblinvong
D.J. Sueldo
Claudia Suemoto
Sze-Chuan Suen
Yusuke Suenaga
Franziska Suess
Felix Suessenbach
Kenneth Sufka
Kazuyuki Sugahara
Alan Sugar
Joel Sugar
Akira Sugawara
Koji Sugawara
Norio Sugawara
Sho Sugawara
Sebastian Suggate
David Suggett
Michael Sughrue
Genichi Sugihara

Tohru Sugihara
Kazuhito Sugimori
Cassidy Sugimoto
Jonathan Sugimoto
Ken Sugimoto
Shinya Sugimoto
Toshiro Sugimoto
Haruhiko Sugimura
Yoshihisa Sugimura
Keishi Sugino
Norihiro Sugino
Kenichiro Sugitani
Hisatoshi Sugiura
Wataru Sugiura
Hitoshi Sugiyama
Munetaka Sugiyama
Takashi Sugiyama
Meryam Sugulle
Byung-Chang Suh
Chang Suk Suh
Sang Jun Suh
Won Suh
Wonhee Suh
Young-Ah Suh
Andreas Suhrbier
Karsten Suhre
Dawen Sui
Feng Sui
Jie Sui
Jing Sui
Wei Sui
Zhenghong Sui
Wawan Sujarwo
N. Sujatha
Gintare Sujetoviene
Ki Tae Suk
Theresa Sukal-Moulton
Galina Sukhova
Béla Suki
Jana Suklan
Naeti Suksomboon
Gita Sukthankar
Raman Sukumar
Pramod Sukumaran
Siddharth Sukumaran
Woo Jun Sul
Mohamad Sulaiman
Ken Sulak
Petr Sulc
Todd Sulchek
Preeti Sule
Justin Sulik
Danielle Sulikowski
Sharain Suliman
Giorgia Sulis
Amanda Sullivan
Brian K. Sullivan
Corwin Sullivan
Craig Sullivan
Cris Sullivan
Daniel Sullivan
David Sullivan
Debra Sullivan
Jon Sullivan
Kimberly Sullivan
Maryrose Sullivan
Michael Sullivan
Robert Sullivan
Sheena Sullivan
Slawomir Sulowicz
Ronan Sulpice
Soriano Sulpicio
Shagufta Sultan
Arshiya Sultana
Papia Sultana
James Sulzer
Dalal Suman
Devi Suman
Saulius Sumanas
Izabela Sumara
Mark Sumarah
Surahyo Sumarsono
Irene Sumbele
Daigo Sumi
Shoichiro Sumi
Yoshio Sumida
Tomiki Sumiyoshi
Ross Summer
Carolyn Summerbell
Artur Summerfield
Cliff Summers
Kyle Summers
Paul Summers
Roger Summons
Anne Sumner
Christine Sumner
Dale Sumner
Emma Sumner
Susan Sumner
John Sumpter
Deepa Sumukadas
Baocun Sun

Bing Sun
Bo Sun
Changquan Sun
Chao Sun
Cheuk-Kwan Sun
Chiao-Yin Sun
Chia-Wei Sun
Chuanzheng Sun
Chunyan Sun
Daniel Sun
Dianjianyi Sun
Dong Sun
Dongxiao Sun
Fang Sun
Fei Sun
Feida Sun
Feng-Hua Sun
Guangyu Sun
Gui-Quan Sun
H. J. Sun
Haibo Sun
Haixiang Sun
Hong Sun
Hong-Qiang Sun
Hongyan Sun
Hongying Sun
Hua Sun
Ji Sun
Jian Sun
Jianguo Sun
Jianlei Sun
Jianwei Sun
Jing Sun
Jingbo Sun
Jingru Sun
Jinhao Sun
Jinyue Sun
Jun Sun
Junfeng Sun
Junying Sun
Kai Sun
Keer Sun
Kehui Sun
Lei Min Sun
Lena Sun
Li Sun
Liang Sun
Liangliang Sun
Lifu Sun
Lihua Sun
Lijun Sun
Lin Sun
Li-Tao Sun
Li-Wei Sun
Lixin Sun
Lun-Quan Sun
Ming Sun
Peng Sun
Peng Gang Sun
Phillip Sun
Qin-Ye Sun
Qun Sun
Ruopeng Sun
Ruoyu Sun
Sean Sun
Sheng Sun
Shiwen Sun
Shujuan Sun
Songsong Sun
Stella Sun
Tianhu Sun
Wei Sun
Weili Sun
Weining Sun
Wenchao Sun
Wenxiang Sun
Wenyue Sun
X. Sun
Xiangdong Sun
Xiangqing Sun
Xiangyang Sun
Xiaodong Sun
Xiaofang Sun
Xiaofei Sun
Xiao-Feng Sun
Xiao-Hong Sun
Xiaojuan Sun
Xiaojun Sun
Xiaoming Sun
Xiaoqian Sun
Xiaoshuai Sun
Xin Sun
Xinghuai Sun
Xuejin Sun
Xue-Zhong Sun
Yang Sun
Yanni Sun
Ya-Xun Sun
Ye Sun
Ye-Huan Sun
Yeqing Sun
Yi Sun

Ying Sun
Yinghao Sun
Yingpu Sun
Yingxian Sun
Yonghua Sun
Yougang Sun
Yu Sun
Yuanyuan Sun
Yuchun Sun
Yuxia Sun
Yuyang Sun
Yvonne Sun
Zeyu Sun
Zhanli Sun
Zhaogui Sun
Zhenxu Sun
Zhi Sun
Zhifu Sun
Zhi-Jun Sun
Zhiyi Sun
Zhonghua Sun
Zilin Sun
Zuoming Sun
Kartik Sunagar
Hajime Sunagozaka
Isaac Sundar
Chandru Sundaram
Krishnaswamy Sundararajan
Balaji Sundararaman
T. Sundararaman
Alamelu Sundaresan
Gobalakrishnan Sundaresan
N. Ravi Sundaresan
Velusamy Sundaresan
Mackenzie Sunday
Mary Sunday
John Sundberg
Rolf Sundberg
T. Sundberg
Johanne Sundby
Roger Sunde
Ulrik Sundekilde
Caroline Sunderland
Pia Sundgren
Henrik Sundh
George W. Sundin
Martha Sund-Levander
Christopher Sundling
Karin Sundström
Asli Suner
Selim Suner
Cynthia Sung
Junne-Ming Sung
Shih-Hsien Sung
Tzu-Ching Sung
Young Chul Sung
Kim Sunggil
Thankam Sunil
Nana Sunn
Nishanth Sunny
Francesco Sunseri
Zacharias Suntres
Sonja Suntrup
Xun Suo
Zhenhe Suo
Tarja Suominen
Matej Supej
Fran Supek
Heidi Super
Gordana Supic
Vijay Suppiah
Vishnu Suppiramaniam
P. Suprasanna
P. Surai
G.K Suraishkumar
Werasak Surareungchai
Paola Surcinelli
Andrzej Surdacki
Eugene Surdutovich
Camille Sureau
Sripathi Sureban
Xisca Sureda
K.C. Surendra
Sinnathamby Surendran
Amritha Suresh
Aneesha Suresh
Kumar Suresh
Sahana Suresh
A. Suresh Babu
Guarav Suri
Jasjit Suri
Joan Suris
Eva Surmacz
C.B. Surman
D. Surmeier
Todd Surovell
Eric Surrey
Kadek Agus Surya Dila
A. Suryawanshi
Alfonso Susanna
John Suschak III
Ashleigh Sushames

Petri Susi
Jamie Suski
Kristina Suson
Vladislav Susoy
Rossana Sussarellu
Leah Susser
Roderich Süssmuth
Robert Suszynski
Angela Sutan
Takashi Sutani
Vijay Sutariya
Siobhan Sutcliffe
Daniel Suter
Glenn Suter
Laura Suter-Dick
Vishal Suthar
Sandra Suther
Chris Sutherland
Clare Sutherland
Colin Sutherland
Heidi Sutherland
Leslie Sutherland
Marcia Sutherland
Matthew T. Sutherland
Scott Sutherland
Iain Suthers
Glenn Sutter
Anne-Laure Sutter-Dallay
Hedwig Sutterlüty-Fall
Fayyaz Sutterwala
Thitima Suttichet
Catherine Suttle
Jeffrey Suttle
Anthea Sutton
Brad Sutton
Jeannette Sutton
Paul Sutton
R. Sutton
Rosemary Sutton
Scott Sutton
Steven Sutton
Troy Sutton
Jolien Suurmond
Petri Suurronen
Kamol Suwannakarn
I. Suwastika
Wannapa Suwonakerd
Mikita Suyama
Marie Suzan-Monti
Jelena Suzic Lazic
Shinya Suzu
Akihiro Suzuki
Atsushi Suzuki
H. Suzuki
Haruo Suzuki
Hideaki Suzuki
Hidekazu Suzuki
Hiroshi Suzuki
Katsuya Suzuki
Kohta Suzuki
Kota Suzuki
Kuninori Suzuki
Makuto Suzuki
Masatoshi Suzuki
Michio Suzuki
Miwa Suzuki
Nobuhiro Suzuki
Rie Suzuki
Rumiko Suzuki
Satoru Suzuki
Taichi Suzuki
Takashi Suzuki
Takeshi Suzuki
Tohru Suzuki
Toru Suzuki
Yôiti Suzuki
Yoshihiko Suzuki
Yuichiro Justin Suzuki
Yuko Suzuki
Yuriko Suzuki
Yusuke Suzuki
Yasuo Suzuoki
Richard Svanbäck
Eva Svanborg
Mia Svantesson
Staffan Svard
John Svaren
Alena Svatkova
Jörundur Svavarsson
Alan Sved
Annika Svedholm-Häkkinen
Josefin Sveen
Erik Svendsen
Per Svenningsen
Sine Svenningsen
Tor Svensjö
Ola Svenson
Birte Svensson
Aaron Sverdlov
Dmitri Svergun
Eva Sverremark-Ekström
Maria Carolina Costa Melo Svidnicki

Terje Svingen
Dmitri Sviridov
Petr Svoboda
John Swaddle
Doug Swain
J. Swain
Lija Swain
Peter Swain
Neil Swainston
Daniel Swale
Sunil Swami
S. Joshua Swamidass
Gokul Swaminathan
Meenupriya Swaminathan
Priya Swaminathan
Siddharth Swaminathan
Subramanyam Swaminathan
Mallikarjuna Swamy
Mummedy Swamy
Shivalingappa Swamynathan
Andrew Swan
William Swaney
Todd Swannack
Brook Swanson
David Swanson
Geoffrey Swanson
Kenneth Swanson
Patrick Swanson
Randel Swanson
Michelle Swanson-Mungerson
Eileithyia Swanton
Seshadri Swapna
Helen Swarbrick
Walter Swardfager
Peter Swart
Benjamin Swarts
Daan Swarts
Tim Swartz
Ghanshyam Swarup
Kathleen Sweadner
Stephen Swearer
Joann Sweasy
Karen Sweazea
Jonathan Sweedler
Elizabeth Sweeney
Jon Sweeney
Kate Sweeny
Matthew Sweet
Michael Sweet
Annika Sweetland
Andrew Sweetmen
David Sweetser
Fadi Sweiss
Dallas Swendeman
Cees Swenne
Jennifer Swenson
Neal Swerdlow
Russell Swerdlow
Elfie Swerts
Tom Swetnam
Susan Swetter
Luc Swevers
Alexander Swidsinski
Pawel Swietach
Benjamin Swift
Michael M. Swift
David Swigon
Yonat Swimmer
William Swindell
Bryan Swingle
Jean Swings
Dorine Swinkels
Johannes Swinnen
David Swinney
Stephen Swioklo
Marek Switonski
Neil Switz
Steven Swoap
Kathryn Swoboda
W. Edward Swords
Ming Hui Sy
Keertiman Syal
Kirtimaan Syal
Adam Sybilski
Khajamohiddin Syed
Moin Syed
Yasir Syed
Zainulabeuddin Syed
Sharifah Faridah Syed Omar
Catherine Sykes
Jan Sykora
Martin Sykora
Peter Sykora
Zuzana Sykorova
Francesca Sylos-Labini
Abigail Sylva
Chad Sylvester
Marie-Pierre Sylvestre
Michel Sylvestre
Stefan Symeonides
Craig Symes
Laurel Symes

Martyn Symmons
Zvi Symon
William Symondson
Andrew Symons
Celia Symons
Marc Symons
Wing-Kin Syn
Grazyna Sypniewska
Vana Sypsa
George Syrogiannopoulos
Vladimir Sytnyk
Jari Syväranta
Laszlo Szabados
Agnes Szabo
Attila Szabo
Ildiko Szabo
Les Szabo
Milan Szabo
Judit Szabó
Piroska Szabó
Annamaria Szabolcs
A.N. Szabo-Reed
Krzysztof Szade
Adam Szafran
Szymon Szafranski
Bertram Szagun
Bozena Szal
Gabriella Szalai
Ewa Szalowska
Lukasz Szarpak
Maria Szczepanska
Agnieszka Szczepek
Izabela Szczerbal
Karen Sze
Marc Sze
Sing-Hoi Sze
Wei Ping Sze
Gábor Szederkényi
Andrea Szekely
Michael Szell
Éva Szentirmai
Nicholas Szerlip
Andrew Szeto
Cheuk-Chun Szeto
Grace Szeto
Nathaniel Szewczyk
Zsolt Sziklavari
Damian Szklarczyk
Grazyna Szklarz
Maciej Szkulmowski
Joanna Szmydynger-Chodobska
Cassandra Szoeke
Francis Szoka
Zsuzsa Szondy
Agnieszka Szopa
Moriah Szpara
R. Sztajzel
S.C. Szu
Przemyslaw Szufel
Pawel Szulc
Irena Szumiel
Sabine Szunerits
Zsuzsa Szvetelszky
Gregor Szycik
Joanna Szyda
Maciej Szydlowski
Emilia Szymanska
Boleslaw Szymanski
Filip Szymanski
P. Szymanski
Agnieszka Szypowska
Mieczyslaw Szyszkowicz
Leen M. 't Hart
Ellen 't Hoen
Hang Ta
Jan-Willem Taanman
Niels Taatgen
Walter Tabachnickk
Valentina Tabanelli
Yasuharu Tabara
Takako Tabata
Yasuhiko Tabata
Daniel Taber
Javier Tabima
Ali Tabish
Eduardo Taboada
Alberto Taboada-Crispi
Juan Taboas
Whitney Tabor
Barbara Taborsky
Michael Taborsky
Gregg Tabot
Shams Tabrez
Maryam Tabrizian
Akiko Tabuchi
Fumiaki Tabuchi
Sadaharu Tabuchi
Takahiro Tabuchi
Ozden Tacal
Carlo Tacchetti
Andrea Tacchino
Ken Tachibana

Tetsuya Tachibana
Hidenori Tachida
Yuuya Tachiki
Yuji Tachimori
Ilias Tachtsidis
Jason Tack
Frank Tacke
Paul Tacon
Angela Taddei
Maria Taddei
Zerihun Tadele
Bernard Hope Taderera
Birkneh Tilahun Tadesse
Satyashankara Tadinada
Rafael Tadmor
Rafik Tadros
Kim Tae Hee
Miriam Taegtmeyer
Mohsen Tafaghodi
Carolina Tafalla
Marco Tafani
Steven Taffet
Fabrizio Taffoni
Angela Taft
Benjamin Taft
Diana Taft
Marcus Taft
Ali Tafti
Agostino Tafuri
Mary Anne Tafuri
Andrew Tager
Gaurav Kumar Taggar
Michael Taggart
Mansour Taghavi Azar Sharabiani
Alberto Tagliafico
Lidia Tagliafierro
Jared Taglialatela
Maurizio Taglialatela
Enzo Tagliazucchi
Nevio Taglieri
Denis Tagu
Tetsushi Taguchi
Y-h. Taguchi
Yuzuru Taguchi
Christina (Naomi) Tague
Rachida Tahar
Behdad Tahayori
Tahereh Taheri
Wendy Taheri
Fariza Tahi
Abd Tahrani
Changfeng Tai
Chi-Ming Tai
Ta-Wei Tai
Behnam Taidi
Alain Taieb
Vijay Tailor
You-Lin Tain
Men-Wong Taing
Emanuela Taioli
Sami Taipale
Hanna Taipaleenmaeki
Campbell Tait
Douglas Tait
Rhim Taiyoun
Shahragim Tajbakhsh
Shahin Tajeri
Maral Tajerian
Gabriel Tajeu
Kiyoshi Tajima
Karoly Takacs
Endre Takács
Ayato Takada
Masahiko Takada
Silvia Takada
Yoshikazu Takada
Giichi Takaesu
Atsushi Takagi
Hideki Takagi
Hiroshi Takagi
Kentaro Takagi
Yuichiro Takagi
Teruhiko Takahara
Akinori Takahashi
Chris Takahashi
Hidetoshi Takahashi
Katsuhide Takahashi
Kazutaka Takahashi
Kei Takahashi
Kohske Takahashi
Koichiro Takahashi
Kyoko Takahashi
Masafumi Takahashi
Mizuki Takahashi
Naoki Takahashi
Nobunori Takahashi
Nobuyuki Takahashi
Shigeo Takahashi
Shigeru Takahashi
Yohsuke Takahashi
Yoshinori Takahashi
Yuji Takahashi

Yusuke Takahashi
Akinobu Takaki
Tomohiro Takaki
Amol Takalkar
Daisuke Takamatsu
Ken Takamatsu
Toshihiro Takamatsu
Shinzo Takamori
Iseki Takamoto
Takeki Takamura
Eriko Takano
Takeshi Takarada
Hajime Takase
Tomoyuki Takase
Shogo Takashiba
Eizo Takashima
Emi Takashita
Atsushi Takata
Fuyuko Takata
Yumie Takata
Yoshiharu Takayama
Hideki Takayasu
Misako Takayasu
K. Takayma
Hirohide Takebayashi
Kiyoshi Takeda
M. Takeda
Makio Takeda
Shunichi Takeda
Yoshitsugu Takeda
Kaori Takehara-Nishiuchi
Yoshiteru Takekita
Hiromu Takematsu
Kazuhiro Takemoto
Minoru Takemoto
Akihiro Takemura
Genzo Takemura
Keizo Takenaga
Shigeo Takenaka
Nikawa Takeshi
Kyosuke Takeshita
R.G. Taketani
Teruko Taketo
Shuji Taketomi
Dan Takeuchi
Hideaki Takeuchi
Ichiro Takeuchi
Osamu Takeuchi
Shoji Takeuchi
Takeshi Takeuchi
Tohru Takeuchi
Tsutomu Takeuchi
Yasuo Takeuchi
Aya Takeyama
Naoshi Takeyama
Faten Taki
Masumi Taki
Gaku Takimoto
Yumi Takiyama
Takumi Takizawa
Keiyo Takubo
Tomoyuki Takura
Richard Takx
Reshef Tal
Adel Talaat
Hossam Sanyelbhaa Talaat
Sahra Talamo
Vicente Talanquer
Antoine Talarmin
Ardesheer Talati
Douglas Talbert
Benoit Talbot
Guylaine Talbot
Robert Talbot
Fahmida Taleb
Tanaji Talele
Pedro Talhinhas
Iannis Talianidis
Leda Talib
Rajeev Taliyan
Stefan Talke
Ben Tall
Victor A. Tallada
Chantal Tallaksen
Miikka Tallavaara
Costellia Talley
Gregory Tallman
Melissa Tallman
Catherine Tallon-Baudry
Matthew Talluto
Regine Talon
Delia Talos
Marina Taloyan
Jaya Talreja
Asoke Talukder
Sangeeta Talwar
Clarence Tam
Connie Tam
Enrico Tam
Lai-Shan Tam
Nicoladie Tam
Ali-Mohammad Tamaddon

Paula Tamagnini
Elena Tamagno
Luca Tamagnone
Ichiro Tamaki
Masanori Tamaki
Satoshi Tamaki
Phanourios Tamamis
Hirokazu Tamamura
Rakesh Tamang
Masanori Tamaoki
Jordi Tamarit
Yutaka Tamaru
Juan Antonio Tamayo-Ramos
Robyn Tamboli
Vittoradolfo Tambone
James Tambong
Leandro Tambosi
Kanittha Tambunlertchai
Jerome Tamburini
Jérôme Tamburini
Sylvie Tambutté
James Tamerius
Ashish Tamhane
Marco Tamietto
Eleonora Tamilia
Faleh Tamimi
Lynda Tamine-Lechani
Ernst Tamm
Toomas Tammaru
Owen Tamplin
Gindo Tampubolon
Kazuhiro Tamura
Kosuke Tamura
Kouichi Tamura
Masahito Tamura
Masato Tamura
Naoto Tamura
Tomohiko Tamura
Yasuaki Tamura
Yuichi Tamura
Yuki Tamura
Andrew Tan
Anjiang Tan
Bee Kang Tan
Beiping Tan
Bruce Tan
Cheemeng Tan
Cher Heng Tan
Chin Wee Tan
Christopher Tan
Chrystalle Tan
Darrell Tan
David Tan
Dun Y. Tan
Gene Tan
Heng Wee Tan
Hong Tan
Hong Yien Tan
Huachun Tan
Hui Shan Tan
Iain Tan
Jiangning Tan
Jie Tan
Jiwei Tan
Joanne Tan
Judy Tan
Katherine Tan
Ken Tan
Lee Tan
Lubin Tan
Nelly Tan
Nguan Soon Tan
Poh Tan
Rui Zhen Tan
Tao Tan
Tina Tan
Tony Tan
Vina Tan
W.L. Tan
Wanlong Tan
Xiaochao Tan
Xu Tan
Xun Tan
Xungang Tan
Yi Tan
Yuliang Tan
Yulong Tan
Yunhao Tan
Zhi Tan
Zhiliang Tan
Zhongming Tan
Katsuyuki Tanabe
Akane Tanaka
Aparecida Tanaka
Eiichi Tanaka
Fumiaki Tanaka
Hirokazu Tanaka
Hiromitsu Tanaka
Kazuhiro Tanaka
Kohichi Tanaka
Kunihico Tanaka
Minoru Tanaka

Nobue Tanaka
Rica Tanaka
Ryouichi Tanaka
Satoshi Tanaka
Shinji Tanaka
Shuhei Tanaka
Takuji Tanaka
Tetsuya Tanaka
Tomoharu Tanaka
Toshiharu Tanaka
Toshio Tanaka
Tsuyoshi Tanaka
Yoshio Tanaka
Yuko Tanaka
Keiko Tanaka-Yamamoto
Vivek Tanavde
Salunya Tancharoen
Tara Tancred
David Tandberg
Edson Tandoc
Nitin Tandon
Shashank Tandon
Vibha Tandon
Bhupesh Taneja
Kentaro Tanemura
Naoko Tanese
Alessandra Tanesini
Aihua Tang
Alice Tang
Baopeng Tang
Beisha Tang
Bo Tang
Buzhou Tang
Chaorong Tang
Chengchun Tang
Cheng-Hao Tang
Chengkai Tang
Chengwei Tang
Chongren Tang
Daniel Tang
Daoquan Tang
David Tang
Faqing Tang
Feng-Yao Tang
Haibao Tang
Hailin Tang
Hong Tang
Huilin Tang
Jen-Ruey Tang
Jiabin Tang
Jie Tang
Jihua Tang
Jijun Tang
Jing Tang
Jin-Hai Tang
Jinsong Tang
Jiping Tang
Jun Tang
Jun-Ming Tang
Kevin Tang
Kwan Ho Tang
Lei Tang
Li Tang
Lingli Tang
Lingqi Tang
M. Tang
Mei San Tang
Ming Tang
Moon-Shong Tang
Peifu Tang
Qiongyao Tang
Qiyi Tang
Qizhu Tang
Rong Tang
Ruimin Tang
Sen-Lin Tang
Shanjiang Tang
Shao-Jun Tang
Shaoxun Tang
Shixing Tang
Simon Tang
Tie-Shan Tang
Tingting Tang
W. H. Wilson Tang
Wanxin Tang
Wei Tang
Wei-Hua Tang
Wei-Jen Tang
Weiming Tang
Weiqing Tang
Wenwu Tang
Xian Dong Tang
Xiangyu Tang
Xiaolin Tang
Xingchun Tang
Yang Tang
Yaohui Tang
Yinghua Tang
Yingying Tang
Yu Tang
Yu Ping Tang
Yuanyuan Tang

Yuanzhi Tang
Yun-Zhao Tang
Zhaoxin Tang
Zhi Tang
Zhonglin Tang
Balamurugan Tangiisuran
Frédéric Tangy
Jun Tani
Kohzo Taniguchi
Koji Taniguchi
Leandro Taniguchi
Manabu Taniguchi
Toshiyasu Taniguchi
Yu Taniguchi
Hiroshi Tanimoto
Keiji Tanimoto
Maged A. Tanios
Yoshihiro Taniyama
Hatice Tankisi
Akhenaten Tankwanchi
Angelo Tanna
Edmund Tanner
Jared Tanner
Julian Tanner
Kandice Tanner
Nichole Tanner
Rachel Tanner
Susan Tanner
Dionne Tanneta
Eric Tannier
Lisa Tannock
Jason Tanny
Marian Tanofsky-Kraff
A. Tanoglu
Masaru Tanokura
Nongnuj Tanphaichitr
Bekir Tanriover
Mathew Tantama
Azza Tantawy
Dean Tantillo
Kelan Tantisira
Michele Tanturli
Bahattin Tanyolac
A. I. Tao
Dayun Tao
Ge Tao
Haiying Tao
Han Tao
Hongbing Tao
Jeremiah Tao
Jianping Tao
Jun Tao
Li Tao
Ling Tao
Mi-Hua Tao
Pan Tao
Sha Tao
Wenbing Tao
Wenjing Tao
Yanmei Tao
Mohammed Taouis
Julio Tapia
Milagritos Tapia
Soile Tapio
Stephanie Taplin
János Tapolcai
Marta Tapparo
Elliot Tapper
D. Tappin
Hannah Tappis
Adriana Tapus
Hanan A. Taqi
Roberta Tarallo
Lucia Taramasso
Margaret Tarampi
Giovanni Tarantino
Marco Tarantola
Eustachio Tarasco
Francisco Jose Tarazona Santabalbina
John Tarbell
Adi Tarca
Isabelle Tardieux
Eric Tardif
Catherine Tardin
Lorenzo Tardon
Florence Tardy
Marianne Tare
Douglas Taren
David Tareste
E.M. Targarona
Kimara Targoff
Ahmad Tarhini
Amare Tariku
Sergey Tarima
Edith Tarimo
M. Umer Tariq
Ina Tarkka
Ahti H.A. Tarkkanen
Geraint Tarling
Susan Tarlo
Mike Tarlov
Pavel Tarlykov

Kustiariyah Tarman
Ioannis Tarnanas
Der-Cherng Tarng
Attila Tarnok
Adam Tarnoki
Mark Tarnopolsky
Alexander Tarnutzer
Jakob Tarp
Lee Tarpley
David Tarpy
Ana Tarquis
Michael Tarr
Philip Tarr
Phillip Tarr
Ilaria Tarricone
Arnaud Tarroux
Aurelien Tartar
Ermira Tartari
Hidenori Taru
Neslihan Tas
Tolga Tasdizen
Alexandre Tashima
Yasutaka Tashiro
Donald Tashkin
Ljubica Tasic
Robert Tasker
Erdogan Taskesen
Anchalee Tassanakajon
Laura Tassi
Bruno Tassin
Chrysoula Tassou
Matthew Tata
Andrey Tatarenkov
Tatiana Tatarinova
Kelly Tatchell
Chemen Tate
Eleanor Tate
Eric Tate
Shunsuke Tatebe
Ryosuke Tateishi
Nicholas Tatonetti
Hirosuke Tatsumi
Shinichi Tatsumi
Ichiro Tatsuno
Keita Tatsuno
Masami Tatsuno
Glenn Tattersall
Martin Tattersall
Pierre Tattevin
Utpal Tatu
Suren Tatulian
Mary Taub
Stefan Taube
Jeffery Taubenberger
Alfred Tauber
Eran Tauber
Marco Taubert
Valentin Taucher
Marilena Tauro
Chris Tausanovitch
Mohammad Tauseef
Mark Tausig
Daniel Taussky
Juergen Tautz
Mohammad Tavakkoli
E.M. Tavakol
Mitra Tavakoli
S. Tavakolpour
Andre Tavares
Joana Tavares
Joao Manuel R.S. Tavares
Kaio Tavares
Tiago Taveira-Gomes
Sara Tavella
E.J. Tavender
Andrea Taverna
Constanza Taverna
Simona Taverna
Marco Taviani
John Tavis
Mahmoud Tavousi
Akinobu Tawada
Ahmed M. Tawfik
Dan Tawfik
Mohamed Tawfik
Bouchra Tawk
Rima Tawk
Christof Taxis
Hock Tay
K. Tay
Richard Tay
Sun Tee Tay
W.T. Tay
Fumihiko Taya
Ichiro Tayasu
Nancy Tayles
Amanda Taylor
Andrea Taylor
Angela Taylor
Anne Taylor
Anthony Taylor
Barbara Taylor

Benton Taylor
Bruce Taylor
Caroline Taylor
Dawn Taylor
Gavin Taylor
Graham Taylor
Hayden Taylor
James Taylor
Janet Taylor
Jared Taylor
Jeremy Taylor
John Taylor
Jordan Taylor
Julia Taylor
Kimberley Taylor
Kira Taylor
Lauren Taylor
Lee Taylor
Linnéa Taylor
Margot Taylor
Mark Taylor
Mark S. Taylor
Melanie Taylor
Michael Taylor
Moray Taylor
Nicholas Taylor
Nigel Taylor
Paul Taylor
Peter Taylor
Rebecca Taylor
Renea Taylor
Robert Taylor
Rod Taylor
Roger Taylor
Roy Taylor
Scott Taylor
Steve Taylor
Tonya Taylor
Tory Taylor
Tyl Taylor
W. Robert Taylor
James Taylor VI
Thomas Taylor-Clark
Sian Taylor-Philips
Martin Taylor-Rowan
Leanne Taylor-Smith
Jean-Marc Taymans
Saad Tayyab
Amin Tayyebi
Reema Tayyem
Cagdas Tazearslan
Abdellatif Tazi
Asmaa Tazi
Andre Tchernof
Veronika Tchesnokova
Nicolas Tchitchek
Braden Te Ao
Lindsey te Brake
Lisa Te Morenga
Jan te Nijenhuis
Arjan te Pas
Hein te Riele
Gregory Teague
Victoria Team
Chloe Teasdale
Matthew Teasdale
Thomas Teasdale
Sarah Teatero
Maria Teresa Tebano
Christoph Tebbe
Angsana Techatassanasoontorn
Karl Technau
Andrea Tedeschi
Gabriele Tedeschi
Luis Tedeschi
Rosamaria Tedeschi
Dario Tedesco
Lisa Tedesco Triccas
Louise Teel
Lawrence Teen
Teemu Teeri
Matthew Teeter
Nathan Tefft
Jonas Tegenfeldt
Mije Teglas
Andrea Teglio
George Tegos
Chee-Keng Teh
Cheng Teh
Cindy Shuan Ju Teh
Lay Kek Teh
Lee-Suan Teh
Oksana Tehlivets
Jamshid Tehrani
Hajime Tei
Kristine Teichman
Vladimir Teif
Markus Teige
Jeffrey Teigler
Kaare Teilum
Yee Yang Teing
Andrzej Teisseyre

Justin Teissie
Justin Teissié
Natacha Teissier
Jerry Teitel
Tiina Teivaanmäki
Ana Teixeira
Andre Teixeira
Bernardo Teixeira
Fernanda Teixeira
Fernando Teixeira
Lúcia Teixeira
Marcus Teixeira
Marta Teixeira
Paul Teixeira
Robson Teixeira
Santuza Teixeira
Fatima Teixeira-Clerc
Armando Teixeira-Pinto
Alberto Teixido
Cristina Teixido
Julian Tejada
Aaron Tejani
Jaime Tejedor
Mysore Tejesvi
Shai Tejman-Yarden
Bimo Tejo
Agnes Telbisz
Dilek Telci
Aneta Teleglow
Aurelio Teleman
Rory Telemeco
Marilyn Telen
Flavia Teles
Fabrice Teletchea
Evgenij Telezhenko
Joseph Telfair
Evelyn Telfer
Janice Telfer
Scott Telfer
Richard Telford
Sam Telford III
Ross Tellam
Erich Telleria
Guillermo Tellez
Maria Tellez-Plaza
Charles Tellier
Antonio Tello-Montoliu
Ariel Telpaz
Arndt Telschow
Luke R. Tembrock
Philip Temby
Ryan Temel
Kevin Temeyer
Andries Temme
Marleen Temmerman
Fernando Tempera
Daniela Tempesta
Joshua Temple
Louise Temple
Pamela Templer
Matthew Templeton
Steven Templeton
Thomas Templeton
Christian Templin
Jean-Jacques Temprado
Arina ten Cate-Hoek
Pius ten Hacken
Bas ten Harkel
Peter ten Klooster
Huib ten Napel
Kirsten ten Tusscher
Olivier Tenaillon
Klaus Tenbrock
Matthias Tenbusch
Michaela Tencerova
Elena Tenconi
Scott Tenenbaum
Tobias Tenenbaum
Lida Teneva
Alexander Teney
Andrea Teng
Ba-Bie Teng
Ching-Hao Teng
Christopher Teng
Hao-Wei Teng
Nianjun Teng
Santani Teng
Weiping Teng
Xiangbin Teng
Raimund Tenhaken
Mirja Tenhunen
Bud Tennant
Ingrid Tennant
Jonathan Tennant
Howard Tennen
Claudio Tennie
Carol Tenopir
Nikolaos Tentolouris
Adrian Teo
Soo Kng Teo
Wei-Peng Teo
Yik-Ying Teo

Mihaela Teodorescu
Jose Teodoro
Uygar Teomete
Luc Teot
Henrique Teotónio
Jetze Tepe
Alan Tepley
Max Teplitski
Beverly Tepper
James Tepper
Sigal Tepper
Cajo ter Braak
Feiko ter Kuile
Akihiko Terada
Yasuhiko Terada
N. Terai
Masanori Terajima
Chikashi Terao
Junji Terao
Hiroki Teraoka
Takeshi Terashima
Hiroyuki Terawaki
Sylvia Terbeck
D. Terentes
Dimitrios Terentes-Printzios
Andrew Terentis
Dmitry Terentyev
Larisa Tereshchenko
Stephanie Terezakis
Scott Terhune
Andrew Terker
Berend Terluin
Nicolás Terrados
Niccolo Terrando
Marco Terraneo
Nora Terrasini
Luis Terrazas
Kim Terrell
Levi Carina Terribile
Philippe Terrier
Paul Terry
Pramod Terse
Jose Teruel
Maria Teruel
Stefan Terzer
Valeria Terzi
Vladimir Tesar
Robert Tesh
Vernon Tesh
Vera Tesic
Jennifer Teske
Gianpaolo Tessari
Jeffery Tessem
May-Britt Tessem
Benjamin Tessler
Claudio Tessone
Alison Testa
Claudia Testa
Maria Testa
David Tester
Pat Tester
Traci Testerman
Catherine Tétard-Jones
Jens Tetens
Diana Teti
Ian Tetlow
Sotiri Tetradis
Sotirios Tetradis
Guillaume Tetreau
Marie-Pier Tetreault
Osamu Tetsu
Tetsuhiro Tetsuhiro Tanaka
Ciro Tetta
Gianluca Tettamanti
Marco Tettamanti
Mauro Tettamanti
Hervé Tettelin
Kay Tetzlaff
Katrin Teubner
Andreas Teufel
Martin Teufel
Wim Teughels
Alexander Teumer
Charlotte Teunissen
Marcel Teunissen
Miguel Teus
Yvonne Teuschl
Rita Tewari
Vishal Tewari
Marta Texeira
Siok-Keen Tey
Sophie Tezenas du Montcel
Sara Tezza
Tom Thacher
Anil Thachil
Christine Thacker
Tyler Thacker
Rosemary Thackeray
Larissa Thackray
Varykina Thackray
Joshua Thaden
Duy Thai

Nhi Thai
Izabella Thais da Silva
Bijin Thajudeen
Juilee Thakar
Maulik Thaker
Mahesh Thakkar
Dhiren Thakker
Basant Thakur
Jitendra Thakur
Krishna Thakur
Meghna Thakur
Mukesh Thakur
Neeta Thakur
Shalabh Thakur
Vijay Kumar Thakur
Serge Thal
Lindsay Thalheim
Richard Thallman
George Thalmann
Clement Tham
Ivan Tham
Johan Tham
Waihong Tham
Mae Thamer
Markus Thamm
Vipa Thanachartwet
Thangavel Thanaraj
David Thanassi
Wendy Thanassi
Rajarajan Thandavarayan
Vu Hong Thang
Balamugesh Thangakunam
Shankar Thangamani
Annadurai Thangaraj
Shakila Thangaratinam
Lakshmipriya Thangavel
Raman Thangavelu
Le Thanh Hoa
K.R. Thankappan
Dharendra Thapa
Rajesh Thapa
Udya Thapa
Roopa Thapar
Binu Tharakan
Pierre-Louis Tharaux
Natalie Thatcher
Shawn Thatcher
Kednapa Thavorn
Jayesh Thawani
Wesley Thayer
Zaneta Thayer
Elitza Theel
Brett Theeler
Elizabeth Theil
Kim Theilgaard-Mønch
Hla-Hla Thein
Daniel Theisen
Michael Theisen
Arianne Theiss
Antonia Thelen
Marcus Thelen
Simon Thelen
Eric Thelin
B.K. Thelma
Goncalo Themudo
Thenappan Thenappan
David Theobald
Martin Theobald
Achilleas Theocharis
Yannis Theocharis
Dan Theodorescu
Georgios Theodoridis
Spyros Theodoridis
Anastasia Theodoridou
Marina Theodorou
Elvar Theodorsson
Argyrios Theofilopoulos
Theoharis Theoharides
Ulrich Theopold
Jakob Theorell
Jenny Theorell-Haglöw
Christine Theoret
Dominique Thepot
Gerhard Theron
Grant Theron
Guillaume Theroux-Rancourt
Francois Therrien
Jörn Theuerkauf
Sebastiaan Theuns
Igor Theurl
Markus Theurl
Catherine Thevenot
Thierry Thevenot
Etienne A. Thévenot
Thanigasalam Thevi
Karin Thevissen
Sascha Thewes
Mia Thi
Jeneni Thiagavel
Francois Thiaucourt
Yann Thibaudier
Helene Thibault

Patrick Thibodeau
David Thickett
Aye Thida
Jean-Baptiste Thiebot
Brian Thiede
Denis Thieffry
Christiane Thiel
Gerald Thiel
Marco Thiel
Martin Thiel
Teresa Thiel
Vera Thiel
Christoph Thiele
Dennis Thiele
M. Thiele
Sven Thiele
Frederick Thielen
Jordy Thielen
E. Thieler
Samuel Thielman
Marcel Thielmann
Joachim Thiem
Tara Thiemann
Thomas Thieme
Christoph Thiemermann
Jorge Thierer
Alain Thierry
Bernard Thierry
Guillaume Thierry
Matthew Thiese
Hans-Jürgen Thiesen
Carrie Thiessen
Ingo Thievessen
Bart Thijs
Vincent Thijs
Victor Thijssen
Michelle Thill
A.W. Thille
Marco Thines
Chloe Thio
Ravivarman Thiruchselvam
Nivethida Thirugnanasambandam
Parthasarathy Thirumala
Parthasarathy D. Thirumala
Soumya Narayani Thirumoorthy
Muthusamy Thiruppathi
Patrice This
Charles Thivolet
Ramkumar Thiyagarajan
Pradeep Thiyyagura
Ingunn Tho
Stephane Thobois
Aaron Thode
Charles Thoen
Kerry Thoirs
Baby Tholanikunnel
Franck Thollot
Dominik Thom
Jeanette Thom
Vladimiros Thoma
Götz Thomalla
Ajith Thomas
Alan Thomas
Andrew Thomas
Balshaw T. Thomas
Beena Thomas
Bernadette Thomas
Billy R. Thomas
Binu Thomas
Bobby Thomas
Bolaji B. Thomas
Bourguignon Thomas
Christoforos Thomas
D. Thomas
David Thomas
Douglas Thomas
Elizabeth Thomas
Emmanuel Thomas
Florence Thomas
Frank R. Thomas
Gerry Thomas
Göen Thomas
Gregg Thomas
Ian Thomas
Isabelle Thomas
James Thomas
Jonathan Thomas
Kelley Thomas
Laura Thomas
Linda Thomas
Matt Thomas
Murray Thomas
Neil Thomas
Nicky Thomas
Peter Thomas
Philipp Thomas
Rebekah Thomas
Rhiannon Thomas
Roger Thomas
Ryan Thomas
Shirley Thomas
Stephanie Thomas

Stephen Thomas
Sufi Thomas
Susan Thomas
T.J. Thomas
Tabitha Thomas
Teh Thomas
Ulrich Thomas
Linda Thomashow
Jeffrey Thomason
Wade Thomason
Robert Thomas-Reilly
Mads Thomassen
Magny Thomassen
Sidinei Thomaz
Rebecca S. Thombre
Beatriz Thome
Margot Thome
Ulrich Thome
Quentin Thommen
Costas Thomopoulos
Aiko Thompson
Amanda Thompson
Angela Thompson
Arthur Thompson
Belinda Thompson
Christopher Thompson
Deanne Thompson
Donna Thompson
Engelberta Thompson
Helen Thompson
Jennifer Thompson
Jeremy Thompson
Jessica Thompson
Jonathan Thompson
Kim Thompson
Lisa Thompson
M.A. Thompson
Mark Thompson
Mary Lou Thompson
Michael Thompson
Mya Thompson
Peter Thompson
Peter A. Thompson
Philip Thompson
R. Houston Thompson
Robert Thompson
Sandra Thompson
Scott Thompson
Stewart Thompson
William Thompson
Julie Thoms
Ann Thomsen
Hauke Thomsen
Isaac Thomsen
Jörn Thomsen
Philip Thomsen
Angus Thomson
Cynthia Thomson
David Thomson
Michael Thomson
Neil Thomson
Sarah Thomson
Scott Thomson
Michael Thon
Frank Thonfeld
Bernard Yu-Hor Thong
Charat Thongprayoon
Jean-Louis Thonnard
Peter Thor
James Thorburn
Stéphane Thore
Lisa Thorell
Bernard Thorens
Gabriel Thorens
Marianne Thoresen
Wallace Thoreson
Gary H. Thorgaard
Eric Thorin
Henrik Thorlacius
Andrew Thorley
Jonas Thorlund
Jonas Thormar
Peter Thorn
Paul Thornalley
Loralei Thornburg
Robert Thornburg
Anne Thorndike
James Thorne
Peter Thorne
Robert Thorne
Sally Thorne
Lars-Eric Thornell
Daniel Thornhill
Graham Thornicroft
Brett Thornton
Claire Thornton
Daniel Thornton
Janet Thornton
Justin Thornton
Patrick Thornton
Ruth Thornton
Timothy Thornton

Margaret Thoroughgood
Steven Thorp
Peter Thorpe
Stephen Thorpe
Susannah Thorpe
Dominic Thorrington
David Thorsley
Christopher Thorstenson
Jagdeshwar Reddy Thota
Dinesh Thotala
Olivier Thoumine
Todd Thrash
Timothy Thrasher
Ronald Thresher
Aaron Thrift
Christopher Thron
James Throne
Edwin Thrower
Mahender Thudi
Peter Thule
Luiz Thuler
Martin Thullner
Ryan Thum
Philip Thuma
S. Thumbi
Julia Thumfart
Michele Thums
Per Thunberg
Evelina Thunell
Kimhan Thung
Pasutha Thunyakitpisal
David Thura
Andrew Thurber
Katherine Thurber
Kristofer Thurecht
Franka Thurm
Steven Thurman
Petra Thürmann
Philipp Thurner
David Thurnham
Ruth Thurstan
Wilfreda Thurston
Ben Thuy
David Thwaites
Guy Thwaites
Julie Thwing
Dominic Thyagarajan
Srinivasan Thyagarajan
Thorsten Thye
Gregory Thyssen
Jacob Thyssen
Lianping Ti
Hanna Tiainen
Marjaana Tiainen
Bo Tian
Chang Fu Tian
Chaoguang Tian
Daxin Tian
Dechao Tian
Geng Tian
Jie Tian
Jing Tian
Jinhui Tian
Jun-Ce Tian
Junzhang Tian
Kegong Tian
Kun Tian
Li Tian
Ling Tian
LinLin Tian
Lixia Tian
Mei Tian
Miaoying Tian
Na Tian
Renmao Tian
Subo Tian
Tianhai Tian
Wei Tian
Weidong Tian
Xiaohong Tian
Xiao-Jun Tian
Xiao-Li Tian
Yangguang Tian
Yantan Tian
Yaping Tian
Ye Tian
Yin Tian
Yuan Tian
Yumin Tian
Yunhong Tian
Guido Tiana
Wang Tianhou
Mao-Meng Tiao
Daniele Tibullo
Vladimir Tichelaar
Andrea Ticinesi
Nicola Ticozzi
Jennifer Tidey
Rosana Tidon
Emmanuele Tidoni
Lu Tie
Anne Tiedemann

Ralph Tiedemann
Markus Tiedge
Meng Tie-Gang
Henning Tiemeier
Bea Tiemens
David Tien
Ming Tien
Adam Tierney
Mary Tierney
Marja Tiirola
Jurgen Tijms
Peter Tijssen
Denis Tikhonov
Ruben Tikidji-Hamburyan
Madeleine M.A. Tilanus-Linthorst
Tamara Tilburgs
Vallo Tilgar
Niko Tiliopoulos
Benedikt Till
Eloise Till
Kevin Till
Douglas Tilley
Leann Tilley
Richard Tilley
Wayne Tilley
Sergei V. Tillib
Therese Tillin
Heather Tillman
F.P. Tillmann
Antonio Tilocca
Sam Tilsen
Jenna Tilt
Bogdan Timar
Narhari Timilshina
Collin Timm
Joerg Timm
Kalinka Timmer
Hans Timmerman
Wilhelmina Timmermans
Leo Timmers
Lucas Timmins
Brian Timmons
Tonis Timmusk
Vladimir Timoshevskiy
Patricia Timper
Sebastian Tims
Jean-Francois Timsit
Youri Timsit
David Timson
Hakan Timur
Willard Tinago
Camilla Tincati
David Tindall
Paulina Tindana
Mbaye Tine
Chien-Kun Ting
Daniel Ting
David Ting
Kang Ting
Lena Ting
David Tingay
Agneta Tinnfalt
Peter Tino
Fausto Tinti
Nathan Tintle
Eve Tiollier
Ramreddy Tippana
Lynette Tippett
Craig Tipple
Trent Tipple
Kyle Tipton
Judit Tirado Muñoz
Valeria Tiranti
Carlos Tirapelli
Claudio Tiribelli
Luca Tirinato
Roberto Tirindelli
Virginia Tirino
Venkataswarup Tiriveedhi
David Tirschwell
Yordanos Tiruneh
Karsten Tischer
Shelley Tischkau
Anna Tischler
Dirk Tischler
Ellen Tisdale
James Tisdale
Peter Tiselius
Anders Tisell
Marcello Tiseo
Samuel Tisherman
Renaud Tissier
Pierre Tissieres
Kehmia Titanji
Vincent Titanji
Alan Titchenal
Vladimir Titorenko
Derek Tittensor
Maarten Titulaer
Angela Tiura
Abhinav Tiwari
Ashutosh Tiwari

Fung Yee Tiwari
Kavindra Tiwari
M. Tiwari
Neeraj Tiwari
Purushottam Tiwari
Siddharth Tiwari
Swasti Tiwari
Vaibhav Tiwari
Vijay Tiwari
Aude Tixier
Marie-Stephane Tixier
Michèle Tixier-Boichard
Ian Tizard
Francesco Tiziano
Marco Tizzano
Kjersti Tjensvoll
Arnt Tjønna
Natalia Tkach
Alexander Tkatchenko
Esteban Tlelo-Cuautle
Ing. Pavel Tlustoš
Michael Tlusty
Chiho To
Kelvin To
Masako To
Sabrina To
Franklin Toapanta
Toru Tobe
Kathleen Tober
Aaron Tobian
Daniel Tobiansky
Desmond Tobin
Irene Tobler
Mathias W. Tobler
Carlo Tocchetti
Giuliano Tocci
Jiraporn Tocharus
Douglas Tocher
Mamoru Tochigi
Chitoku Toda
Shigenobu Toda
Catherine Todd
David Todd
James Todd
Jim Todd
Michael Todd
Nevins Todd
Rebecca Todd
David Todem
Anne Todgham
Sokol Todi
Michail Todorov
Svetoslav Todorov
Vladimir Todorov
Margarita Todorova
Jan Toelen
Rene Toes
Alexander Toet
Anna Toffan
Claire Toffano-Nioche
Elena Toffol
Giuseppe Toffoli
Monica Toffoli-Kadri
Jason Toft
Soren Toft
Roberto Togneri
Alessandro Tognetti
Massimiliano Tognolini
Gianluca Tognon
Tatsuru Togo
Junya Toguchida
Toyin Togun
Alicia Toh
Cheng-Hock Toh
Yasushi Toh
Bojan Toholj
Virpi Töhönen
Gabriele Toietta
Diana Toivola
Raine Toivonen
Yuji Toiyama
Hakan Toka
Erik Tokar
Daniel Toker
Anna-Maria Tokes
Serhat Tokgoz
Seiichi Toki
Toshihiro Tokiwa
Aytekin Tokmak
Savvas Tokmakidis
Gaku Tokuda
Junichi Tokuda
Makoto Tokuda
Kumpei Tokuyama
Shogo Tokuyama
Stefano Toldo
Magdalena Tolea
Paloma Toledo
Rafael Toledo
Gabriela Toledo-Ortiz
Conny Tolf
Virginie Tolle

Ann Tollefson
Sue Tolleson-Rinehart
Keith Tolley
Krystal Tolley
Daniel Tollin
Ralph Tollrian
Sergei Tolmachev
Marcelo Tolmasky
Vicki Tolmay
Emanuela Tolosano
Ariel Toloza
Ashita Tolwani
Sarah Tom
Veronica Tom
Catalin Toma
Claudia Toma
Marieta Toma
Mike Toman
Ram Sewak Tomar
Scott Tomar
Hummel Tomas
José Manuel Tomás
Josefa Tomás
Francisco A. Tomás-Barberán
Giovanni Tomasello
Milica Tomasevic
Matteo Tomasi
Thomas Tomasi
Barbara Tomasino
Adam Tomašových
Jordi Tomas-Roig
Francesco Tomassini
Analia Tomat
Yasutake Tomata
Diana Tomback
Etelka Tombácz
Jeffery Tomberlin
Huseyin Tombuloglu
Michal Tomczyk
Sara Tomczyk
Margarida Tomé
Vajdana Tomic
Kentaro Tomii
Andrew Tomita
Tadakimi Tomita
Shinichiro Tomitaka
Susumu Tomiya
Hirofumi Tomiyama
Kazuhito Tomizawa
Minoru Tomizawa
Oren Tomkins
Robert Tomko Jr.
Dana Tomlinson
Simon Tomlinson
Tucker Tomlinson
Luca Tommasi
Steven Tommasini
Mark Tommerdahl
Keizo Tomonaga
Ichiba Tomoyuki
Emile Tompa
Piotr Tompalski
Charlotte Tompkins
Phillip Tomporowski
T.T. Tompuri
Anton Tomsic
Jiang Ton
Massimo Tonacchera
Sarah Tonack
Camilla Tondel
Claudio Tondo
Renata Tonelli
Lucio Tonello
Mehmet Toner
Michela Tonetti
Aaron Tong
Carl Tong
Daoqin Tong
Henry Tong
Jingou Tong
Louis Tong
Philip Tong
Qingchun Tong
Shuping Tong
Tiejun Tong
Van Tong
Wei Tong
Xiaoyong Tong
Xinming Tong
Xiuhong Tong
Xuetao Tong
Yiping Tong
Yongqing Tong
Yu Tong
Yufeng Tong
Enrico Tongiorgi
Sissades Tongsima
Pierluigi Toniutto
Anke Tönjes
Henri Tonnang
Giulio Tononi
Chun Lai Too

Christopher Toomajian
Zahra Toossi
Reuben Tooze
Evangelos Topakas
József Topál
Gulacti Topcu
Nursen Topcuoglu
Jolanta Topczewska
Natalia Toporikova
Stephanie M. Topp
Jorma Toppari
Stefano Toppo
Magdy Torab
Fatemeh Torabi Asr
Pablo Toral
Homero Toral-Cruz
Gary Toranzos
Elena Torban
Atilla Tordai
Daniele Torella
Luiza Torelli
Helena Torezan Silingardi
Christian Torgersen
Paul Torgerson
Senoe Torgerson
Vågen Tor-Gunnar
Angelica Torices
Toshihiko Torigoe
Claudia Torino
Maria Torino
Åsa Torinsson Naluai
Dante Torio
Carol Toris
Kinya Toriyama
Oivind Torkildsen
Jared Torkington
José Tormos
Montserrat Torne
Nuria Torner
Jorge Toro
Beverly Torok-Storb
Hans Torp
Andrea Torrão
Juan Torras
Ignasi Torre
Asunción Torregrosa
Jordi Torrelles
Montserrat Torremorell
Francisco Torrens
Marc Torrent
Angelo Torrente
Maria Torrente
Yvan Torrente
Adriana Torres
Antoni Torres
Arturo Torres
Cesar Torres
David Torres
Fernando Torres
Iraci Torres
Jiram Torres
Joana Torres
Jordi Torres
Julio Torres
Larissa Torres
Letícia Torres
Rita Torres
Tatiana Torres
Thiago Torres
Ulysses Torres
Vicente Torres
Verônica Torres da Costa e Silva
Daniel Torres Lagares
Mario Torres-Acosta
Ignacio Torres-Aleman
Clara Torres-Barceló
Julian Torres-Dowdall
Enelio Torres-Garcia
Diego Torres-Russotto
Jesus Torres-Vazquez
Francesca Torriani
Sandra Torriani
Pernille Torring
Niels Tørring
Lluís Oviedo Torró
Antonio Torroni
Donald Torry
Antonio Torsello
Adriano Tort
Jose Tort
Marta Tortajada
Massimo Tortarolo
Mauro Torti
Suzy Torti
Valeria Torti
Domingo Tortonese
Alfonso Tortorella
Vetle Torvik
Franco Toscani
Matteo Toscani
Marguerite Toscano
Vincenzo Toscano

Nicola Toschi
Moreno Toselli
Nobuyuki Toshikuni
Luiz Tosi
Sabrina Tosi
Simone Tosi
Andrea Tosti
Tibor Tot
Adel Toth
Arnold Toth
Attila Toth
Damon Toth
Ian Toth
Judit Toth
Kalman Toth
Michael Toth
Szilvia Toth
Tunde Toth
Balázs Tóth
Kinga Tóth
Emily Toth Martin
Béla Tóthmérész
Agnes Toth-Petroczy
J.E.E. Totte
Patricia Totten
Sarah Totton
Julie Toubiana
Chirine Toufaily
Mahmoud Toulany
Marlin Touma
Themis Toumanidou
Fateh Toumi
Fatouma Toure
David Tourigny
Jean-Nicolas Tournier
Jérôme Tourret
Warren Tourtellotte
Jason Tourville
Yannick Tousignant-Laflamme
Dimitris Tousoulis
Emmanuel Toussaint
Leonide Toussaint
Olga Toussova
Pierre-Louis Toutain
Robert Toutkoushian
Hazem Toutounji
Daan J. Touw
William Tov
Andres Tovar
Juan Tovar
Sulay A. Tovar
Gordana Tovilovic
Chris Towlson
Natavudh Townamchai
Jonathan Towner
Lindsay Townes
Ellen Townes-Anderson
Helen Townley
Aloen Townsend
Ellen Townsend
George Townsend
Marilyn Townsend
Nathan Townsend
Simon Townsend
Brian Toy
Sophie Toya
Ashley Toye
Francine Toye
Hidenori Toyoda
Takashi Toyofuku
Satoshi Toyokawa
Wataru Toyokawa
Shinya Toyokuni
M. Toyoshima
Kevin Tozer
Jozsef Tozser
Alberto Tozzi
Vincent Traag
Nathaniel Traaseth
Maret Traber
Michele Trabucchi
Edouard Trabulsi
Daniah Trabzuni
Joshua Trachtenberg
Howard Trachtman
Zdenek Trachtulec
Chris Tracy
Derek Tracy
Kathleen Tracy
Melissa Tracy
Sarah Tragesser
Margot Tragin
Claudia Traidl-Hoffmann
Frances Trail
Heather Traino
Anne Trainor
Brian Trainor
Paula Traktman
Dmitry Traktuev
J.G. Tralhao
S. Trambacz-Oleszak
Clinton Trammel

Scott Trammell
Bich Tran
Bonnie Tran
Dat Tran
Duong Tran
Nhan Tran
Pamela Tran
Quang-Kim Tran
Thach Tran
Tuan Tran
Guy Tran Van Nhieu
Timothy Tranbarger
Jean-François Trani
James Traniello
Karin Tran-Lundmark
Lucy Tran-Nguyen
François Tranquart
Michael Tranter
Michael Tranulis
Isidore Tiandiogo Traore
Kuan Traoré
Bruce Trapnell
Leonardo Trasande
Philip Trathan
Mary Traub
Michael Traub
Richard Traub
Michael Traurig
Walther Traut
Jeanette Trauth
R. Alberto Travagli
Bruno Travassos
Mark Travassos
Ana Traven
Brenna Traver
Brittany Travers
Carlos Travieso
Sachia Jo Traving
Alexander Travis
Fred Travis
Ilias Travlos
Vincent Traynelis
Kirsten Traynor
Laurel Traynowicz
Martin Trbusek
Armen Trchounian
Michael Treadway
Charlene Treanor
Darren Treanor
Jonel Trebicka
Piotr Trebicki
Enricomaria Trecarichi
Edward Tredget
Nicole Trefault
Nathan Treff
Giorgio Treglia
Indi Trehan
Gareth Treharne
Sandra Trehub
Emmanuel Treiner
Roi Treister
Sebastian Trejo
Oswaldo Trelles
Francois Tremblay
Marie-Eve Tremblay
Michel Tremblay
Yves Tremblay
Eric Treml
Vaclav Treml
Stephanie Trend
Michael Trenell
Robert Trengove
Verena Trenkel
Edmondo Trentin
Dominic Trepel
Marco Trerotola
Achim Tresch
Martin Tresguerres
Laszlo Tretter
Natalia Tretyakova
François Treussart
Analia Trevani
Ben Trevaskis
Stacey Trevathan-Tackett
Silvia Trevelin
Victor Trevino
Rafael Trevisan
Sara Trevisan
Paolo Trevisi
Jill Trewhella
Karli Treyvaud
Viviana Trezza
Omar Triana-Chávez
Kathy Triantafilou
Cristos Triantos
Stefano Triberti
Jeffrey Triblehorn
Andrea Tricco
Antonia Trichopoulou
Hervé Tricoire
Steven Triezenberg
N. Trifi-Farah

Elena Trifiletti
Dragana Trifunovic
Robert Trigiano
Cesar Trigueros
Mirko Trilling
Maria Angeles Trillo
Jeffrey Trimarchi
Matteo Trimarchi
Mark Trimmer
Katherine Trinajstic
Javier Triñanes
Alexandre Trindade
Tito Trindade
Cong Trinh
Quoc-Dien Trinh
Thu Le Trinh
Vickery Trinkaus-Randall
Laura Trinkle-Mulcahy
Ludovic Trinquart
Olimpia Trio
Wanida Tripanichkul
Anubhav Tripathi
Anusri Tripathi
Deeksha Tripathi
Dinesh Tripathi
Kaushlendra Tripathi
Kumar Parijat Tripathi
Manish Tripathi
Ravi Tripathi
Shashank Tripathi
Timir Tripathi
Vandana Tripathi
Vinay Tripathi
Vishal Tripathi
Chittu Tripathy
Jaya Tripathy
Shreejoy Tripathy
Simon Triphan
N. Triplett
Amber Tripodi
Gail Tripp
Vivian Tristão
Stephen Tristram
Luigi Tritapepe
Jennifer Trittmann
Tiziana Triulzi
Stefania Triunfo
Drenka Trivanovic
Alpa Trivedi
Chinmay Trivedi
Chintan Trivedi
Ranak Trivedi
Jan Trnka
Chris Trobacher
Mats Troedsson
Emily Troemel
Justin Trogdon
Richard Troiano
Jörg Trojan
Sara Trojan
Luigi Trojano
Maria Trojano
Maria Trojanowska
Kim Trollope
Julian Trollor
Domenico Trombetta
Isabelle Tromme
Stella Trompet
Laure Tron
Xoana Troncoso
Volker Tronnier
Nicholas Troop
Carl Tropper
Nadine Troquete
Marius Troseid
Yuri Trotsenko
Darren Trott
Jocelyn Trottier
Patrick Trotzke
Ioannis Trougakos
Jacqueline Trouillas
Lucy Troup
Constantine Troupes
Amalia Trousson
Leendert Trouw
Antonio Trovato
Fabio Trovato
Guglielmo Trovato
Jone Trovik
Kelly Trowbridge
Karen Troy
Todd Troyer
Gilles Truan
Emiliano Trucchi
Jean-Francois Truchon
Lisbeth Truelstrup Hansen
Germain Trugnan
Allison Truhlar
Andrea Truini
Bill Truitt
Andrea Trujillo
Carlos Trujillo

Pavel Trunecka
Dothang Truong
Hong-Ha Truong
Lisa Truong
Trong-Kha Truong
Jesse Trushenski
George Truskey
Anita Truttmann
Piotr Tryjanowski
Mateusz Trylinski
Volodymyr Tryndyak
Gosia Trynka
Monika Trzcinska
Krzysztof Trzcinski
Anna Trzeciecka
Olga Tsachouridou
Konstantinos Tsagarakis
Nikos Tsagias
Chang-Hsiung Tsai
Chang-Youh Tsai
Chia-Fen Tsai
Chia-Liang Tsai
Chih-Fong Tsai
Ching-Hui Tsai
Ching-Piao Tsai
Chun-Hao Tsai
Henghsiu Tsai
James Tsai
Jer Chia Tsai
Kate Tsai
Keng-Chang Tsai
Kenneth Tsai
Kun-Hsien Tsai
Kun-Ling Tsai
Li-Yu Tsai
Meng-Hsun Tsai
Meng-Kun Tsai
Ming-Chao Tsai
Ming-Horng Tsai
Pei-Chien Tsai
Pei-Jane Tsai
Pi-En Tsai
Ray Tsai
Rong-Kung Tsai
Scott Tsai
Siu Tsai
Tsen-Fang Tsai
Tsuen-Chiuan Tsai
W. Tsai
Wei-Lun Tsai
Wen-Chan Tsai
Wen-Chieh Tsai
Yien Tsai
Ying-Chieh Tsai
Yu-Huan Tsai
Panagiotis Tsakanikas
Ephraim Tsalik
Miriam Tsalyuk
Anna Tsang
Ling Ming Tsang
Michael Tsang
Stephen Tsang
Suk Ying Tsang
Tim Tsang
Wang Tsang-En
Anna Tsantili-Kakoulidou
Betty Tsao
Doris Tsao
Fen-Ming Tsao
Ming-Sound Tsao
Tsu-Yu Tsao
Anastasios Tsaousis
Aristidis Tsatsakis
Jau-Yih Tsauo
Timothy Tschaplinski
Diogo Tschoeke
Carsten Tschoepe
Alexander Tschoner
Gary Tse
Herman Tse
Hubert Tse
John Tse
Lap Ah Tse
Shu Tse
Yuk-Ching Tse-Dinh
Theodore Tsekeris
Lawrence Tsen
Chi-Hong Tseng
Chih-Wei Tseng
Ching-Li Tseng
Chin-Hsiao Tseng
Deng-Yu Tseng
Elaine Tseng
Henry Tseng
Kuei Tseng
Kuo-Chih Tseng
Li-Chun Tseng
Shun-Fu Tseng
Tai-Chung Tseng
Victoria Tseng
Yung-Che Tseng
Yu-Yao Tseng

Zhijie Jack Tseng
Vera Tsenkova
Victor Tsetlin
Amalia Tsiami
George Tsiamis
Chryssa Tsiara
Konstantinos Tsilidis
Karl Tsim
Maria Tsimidou
Apostolos Tsiouris
Nikolaos Tsirikos Karapanos
Dimitrios Tsitsigiannis
Serafeim Tsitsilonis
Georgios Tsivgoulis
Guy T'Sjoen
Lai Sze Tso
Emmanuel Tsochatzis
Pavlina Tsoka
Apostolos Tsolakis
Renée Tsolis
Andrew Tsotinis
Hsiao-Hui (Sophie) Tsou
Ming-Hsiang Tsou
Georgios Tsoulfas
Candy Tsourounis
Tatiana Tsoutsman
Konstantin Tsoyi
Debby Tsuang
Hiroto Tsuboi
Hiroyuki Tsubomi
Kazuo Tsubota
Hideo Tsubouchi
Tsutomu Tsuchida
Takuya Tsuchihashi
Atsunori Tsuchiya
Ken Tsuchiya
Masahiro Tsuchiya
Masao Tsuchiya
Naoyuki Tsuchiya
Makoto Tsuda
Soichiro Tsuda
Yoshiaki Tsuda
Tsuyoshi Tsuduki
Amy Tsui
Martin Tsui
Po-Hsiang Tsui
Taishi Tsuji
Takemasa Tsuji
Tetsuro Tsujimoto
Kayoko Tsujino
Takahiro Tsukahara
Hidekazu Tsukamoto
Hiroki Tsukamoto
Kazuhisa Tsukamoto
Hikaru Tsukazaki
Takuji Tsukiyama
Kazunori Tsukuda
Masayuki Tsuneki
Allan Tsung
James Tsung
Mikhail Tsurkan
Kanji Tsuru
T. Tsuruda
Junya Tsurukiri
Shin-ichi Tsuruta
Shogo Tsuruta
Yuya Tsurutani
Kazuhiko Tsuruya
Takumi Tsutaya
Neil Tsutsui
Kamen Tsvetanov
Milena Tsvetkova
Z. Tsvuura
Andrey Tsyganov
Olga Tsyusko
Benjamin Tu
C.F. Tu
Dongsheng Tu
Huakang Tu
Jianhua Tu
Jumin Tu
Qichao Tu
Samson Tu
Tsang-Wei Tu
Wei Tu
Xiaolin Tu
Nguyen Tuan
Nguyen Minh Tuan
Apichai Tuanyok
Christopher Tubbs
Roberto Tuberosa
Sandy Tubeuf
Franca Maria Tuccillo
Tiziano Tuccinardi
Mihran Tuceryan
Lorena Tuchscherr de Hauschopp
Kellie Tuck
Abigail Tucker
Anthony Tucker
Catherine Tucker
Jared Tucker
Katherine Tucker

Kristal Tucker
Kylie Tucker
Mark Tucker
Richard Tucker
Robin Tucker
Lisa Tucker-Kellogg
Robert Tuckey
Midori Tuda
Sue Tuddenham
Rubin Tuder
Griselda Tudo
Eva Tudurí
Bettina Tudzynski
Paul Tudzynsky
Ivan Tuf
Fatih Tufan
Vincenzo Tufarelli
Alda Tufro
Volkan Tugcu
Sharof Tugizov
Benedetta Tugnoli
Remco Tuinier
Mick Tuite
Anil Man Tuladhar
Zsolt Tulassay
Hardeep Singh Tuli
Sara Tulipani
Mats Tullberg
Markus Tuller
Jonathan Tullis
Madalina Tuluc
Kamil Tuluce
Hayrettin Tumani
Lyvonne Tume
Dmitry Tumin
James Tumlin
Katherine Tumlinson
Santa Tumminia
Innocent Tumwebaze
Nazarius Mbona Tumwesigye
James Tumwine
Rosa Tundis
Marta Tunesi
Bui Thanh Tung
Chih-Wei Tung
Chun-Wei Tung
Jenny Tung
Kenneth Tung
Wen-Wen Tung
Dirk Tunger
Su Tung-Hung
James Tunnell
María Tuñón
Huruma Tuntufye
Helena Tuomainen
Elaine Tuomanen
Jaakko Tuomilehto
Francesca Tuorto
Delphine Tuot
Rossella Tupler
Simon Tuplin
Domenico Tupone
Andrea Tura
Olga Tura
Nefize Turan
Ferit Turanli
Paola Turano
Tamás Turányi
Chiara Turati
Massimo Turatto
Guillaume Turc
Catrinel Turcanu
Gianluca Turcatel
Andreia Turchetto Zolet
Chiara Turchi
Giovanni Turchini
Marco Turco
Ronald Turco
Katia Turcot
Gustavo Turecki
Jay Tureen
Ali Turhan
Zsolt Turi
Luca Turin
Francesco Turino
Adrián Turjanski
Boris Turk
Katherine Turk
Metin Türkay
Kemal Turker
Eric Turkheimer
Thomas Turkington
M. Turkyilmazoglu
Mustafa Turkyilmazoglu
Eva Turley
Matt Turley
Katarzyna Turnau
Deborah Turnbull
Jeremy Turnbull
Anne Turner
Anthony Turner
Bradley Turner

Brianna Turner
Claire Turner
Helen Turner
Ian Turner
Jill Turner
Jonathan Turner
Joseph Turner
Justin Turner
Katherine Turner
Kevin O. Turner
Kieran Turner
Lorinda Turner
Michael Turner
Nancy Turner
Neil Turner
Nigel Turner
Paul Turner
Renée Turner
Richard Turner
Robert Turner
Russell Turner
Shane Turner
Susan Turner
Trudy R. Turner
Tychele Turner
Dan Turner-Evans
Gabrielle Turner-McGrievy
Gordon Turner-Walker
Xavier Turon
Noreen Tuross
Konstantin Turoverov
Bernd Turowski
Pablo Turrero
Stefania Turrina
Francesco Turrini
Francesca Turroni
A. Tursi
Stephen Turton
Serap Türüt-Asik
Carolyn Turvey
Víctor Manuel Tuset
Thomas Tütken
Mike Tuttle
Antonino Tuttolomondo
Emin Ediz Tutuncu
Erdem Tüzün
Anders Tveita
Michael Twa
Marc Twagirumukiza
Simon Tweddell
Jean Twenge
Hannock Tweya
Gilad Twig
Stephen Twigg
Sally Twining
Craig Twist
Martin Twiste
Neetu Tyagi
Nikhil Tyagi
Rahul Tyagi
Suresh Tyagi
Wricha Tyagi
Joshua Tybur
Erika Tyburski
Alexandra Tyers
Lars-Oliver Tykocinski
Ronald Tykoski
Robert Tykot
Anna Tyler
Damian Tyler
Kenneth Tyler
Ludmila Tyler
Rich Tyler
Peter Tymms
Angela Tyner
Jeffrey Tyner
Andrew Tyre
David Lorne Tyrrell
Pippa Tyrrell
Rick Tyrrell
Niklas Tysklind
Reny Tyson
Reidar Tyssen
Eric Tytell
Yulia Tyurina
Taina Tyystjärvi
Orian Tzadik
Georgios Tzanakakis
Nikolaos Tzanakis
Evangelos Tzanatos
Ioannis Tzanetakis
Socrates Tzartos
Athanasia Tzelepi
C. Tzeng
Dong-Sheng Tzeng
Jung-Ying Tzeng
Tzong-Der Tzeng
Wen-Jer Tzeng
Dimitrios Tziakas
Athina Tzinia
Konstantinos Tziomalos
Athanasios Tzioufas

Nikolaos Tzortzakis
Argyris Tzouvelekis
