# Supplementary material for: PLOS ONE 2016 Reviewer and Editorial Board Thank You
Source: PLoS One. 2017 Mar 20;12(3):e0174259. doi: 10.1371/journal.pone.0174259 (PMC5358840; doi:10.1371/journal.pone.0174259)
Supplement: S5 Reviewer List — (PDF) [file pone.0174259.s006.PDF]

PLOS | ONE

PLOS ONE would like to thank all those who reviewed on behalf of the journal in 2016:

Carles Ubeda
Paloma Ubeda
Ergun Uc
Olga Ucar
Yurdanur Ucar
Annachiara Uccellatore
Nurcan Üçeyler
Rosalie Uchanski
Hiroo Uchida
Shunya Uchida
Shusaku Uchida
Takafumi Uchida
Thomas Uchida
Kimiko Uchii
Haruto Uchino
Ken Uchino
Shigehiko Uchino
Shigeo Uchino
Kazuhiko Uchiyama
Munehiro Uda
Nobuyuki Udagawa
Joshua Udall
Makarla Udayakumar
Aftab Uddin
Shahadat Uddin
Zakir Uddin
Belinda Udeh
Klas Udekwu
Dan Uden
Julia Udesky
Heiichiro Udono
Jayaram Udupa
Kaviraja Udupa
Sripada Udupa
Khalil Udwan
Kazuhiro Ueda
Keiji Ueda
Masahiro Ueda
Masashi Ueda
Mitsuyoshi Ueda
Natsuo Ueda
Peter Ueda
Seiji Ueda
Shinichiro Ueda
Gen Uehara
Kazumasa Uehara
Yuki Uehara
Carlos Ueira
Christopher Uejio
Atsuko Ueki
Masao Ueki
S. Ueki-shige
Thor Uelend
Hiroji Uemura
Mamoru Uemura
Shinji Ueno
Takashi Ueno
Yoshiyuki Ueno
Yoshihiro Uesawa
Hirotsugu Ueshima
Massaro Ueti
Akihito Uezato
Rafael Ufret-Vincenty
Sebastian Ugbaje
Caroline Uggla
Carlos Ugrinowitsch
Arnaud Uguen
Florian Uhle
Anne-Catrin Uhlemann
Per Uhlen
Holm Uhlig
Stefan Uhlig
Jiri Uhlik
Mirka Uhlirova
Eric Luis Uhlmann
Sebastian Uhlmann
Kathryn Uhrich
R. Glen Uhrig
Pavel Uhrin
Rosalie Uht
Jouni Uitto
Bernadett Ujhelyi
Tetsuro Ujihara
Takanori Uka
Anindita Ukil
Dede Ukueberuwa
Kandasamy Ulaganathan
Ifeoma Ulasi
Sebastian Ulbert
Karin Ulbrich

Susanne Ulbrich
Niels Uldbjerg
Thomas Uldrick
Glen Ulett
Tim Ulinski
Paola Ulivi
Ahammed Ullah
Ghanim Ullah
Hemayet Ullah
Insha Ullah
Mohammad Ullah
Erik Ullian
Amanda J. Ullman
Buddy Ullman
Diane Ullman
Ekkehard Ullner
Alfredo Ulloa-Aguirre
Rolando Ulloa-Gutierrez
Oliver Ullrich
Stephen Ullrich
Susanne Ullrich
Markus Ullsperger
Barbara Ulmasov
Angela Ulrich
Frey Ulrich
Magda Ulrich
Scott Ulrich
Werner Ulrich
Yvonne Ulrich-Lai
Engin Ulukaya
Sinan Uluyol
Stine Ulven
Alexandra Ulyanova
Soo-Jung Um
Divya Uma
Shanmugasundaram Uma
Samuil Umansky
Viktor Umansky
Shahid Umar
Umar Umar Ali
Brian Umberger
Kate Umbers
Claudia Umbreit
M. Umbrello
Ifeanyi Ume
Hiroyuki Umegaki
Hisanori Umehara
T. Umehara
Myco Umemura
Lars Umlauf
Ramesh Ummanni
Daniel Umpierre
M. Renée Umstattd Meyer
Hamiyet Unal
Robert Unckles
Charles Underwood
Fiona Underwood
Jonathan Underwood
Tony Underwood
Jaime Undurraga
Lyle Ungar
Christoph Ungemach
Elizabeth Unger
Erica Unger
Jennifer Unger
Martin Ungerer
Rodolfo Ungerfeld
Tamas Ungi
Peter Unmack
Elizabeth Unni
Kenji Unno
Tatsuya Unno
Jon Unosson
Klaus Unsicker
Nash Unsworth
Costin Untaroiu
Josef Unterrainer
Eva Untersmayr
Joseph Unthank
Sathya Unudurthi
Frederick Unverzagt
Susanne Unverzagt
Pramod Upadhyay
Santosh Upadhyay
Swapna Upadhyay
John Updegraff
Stephan Uphoff
Priya Uppuluri
Hidetoshi Urakawa
Fumihiko Urano
Takeshi Urano
Iván Uray
Karen Uray
Kevin Urayama
Constantin Urban
Josef Urban
Joseph Urban
Jacek Urbanek
Michael Urbaniak
Rosa Urbanos Garrido
Ewa Urbanska
Rolf Urbanus

Jenny Urbina
Nicolas Urbina-Cardona
Carlo Urbinati
Antonio Urda
Philip Uren
Aykut Üren
Tamsyn Uren Webster
Pablo Ureña-Torres
Jaime Uria-Diez
Iker Uriarte
J. Uriarte
Flavio Uribe
Phillip Uribe
Armando Uribe-Rivera
Nastassia Urien
Elodie Urlacher
Samuel Urmy
Gerald Urquhart
Olaia Urrutia
Nikhil Urs
Francesco Ursini
Gianluca Ursini
Stefan Ursu
Raquel Urtasun
Elizabeth M. Urteaga
Pawel Uruski
Clara Urzí
Ulises Urzua
P. Usai
Masahide Usami
Karen Usdin
Jakob Usemann
Jane Usher
Juliet Usher-Smith
James Usherwood
Takahiro Ushida
Takashi Ushimaru
Vytautas Usonis
David Ussery
James Ussher
Julnar Usta
Suayib Üstün
Kentaro Usuda
Vera Usuelli
Effua Usuf
Chie Usui
Kengo Usui
Mykhaylo Usyk
Victor Uteshev Gaard
Olalekan Uthman
Achint Utreja
Pilar Utrilla
Katarina Uttervall
Lesley Uttley
Sagar Utturkar
Kristina M. Utzschneider
Geoffrey Uy
Metin Uyanik
Shin-ichi Uye
Korkut Uygun
Baris Uz
Antonio Uzal
Francisco Uzal
Katsuhiro Uzawa
Svetlana Uzbekova
Gunes Uzer
Benjamin Uzochukwu
Jude Uzonna
Vinod V.
A. Vaandrager
Suvi Vaara
Felipe Vaca Paniagua
Alessandra Vacari
Michele Vacca
Monica Vaccari
Olga Vaccaro
Matteo Vacchi
Christel Vaché
Tushar Vachharajani
Dominic Vachon
Pierre Vachon
Federica Vacondio
Timothy Vaden
Pieter Vader
Rajanikanth Vadigepalli
Francesco Vadini
Kumar Vadlapudi
Viral Vadwai
Henrik Vægter
Krist Vaesen
Federico Vaggi
Riccardo Vago
Nasim Vahabi
Shahabeddin Vahdat
Nasser Vahdati Mashhadian
Antti Vaheri
Wilfried Vahjen
Marie Vahter
Stefania Vai
Andrea Vaiana
Anil Vaidya
Avinash Vaidya

Dhananjay Vaidya
Gaurav Vaidya
Jatin Vaidya
Varun Vaidya
Vidita Vaidya
Ravi Vaidyanathan
Subramanian Vaidyanathan
E.A. Vail
David Vaillancourt
René Vaillancourt
Andrew Vaillant
Daniel Vaiman
William Vainchenker
Anssi Vainikka
Martti Vainio
Seppo Vainio
Mariz Vainzof
G. Vaiopoulos
Parag Vaishampayan
Chetana Vaishnavi
Anne-Charlotte Vaissière
Pradeebane Vaittinada Ayar
Sandor Vajda
Claire Vajdic
Peter Vajkoczy
Majid Vakilynejad
Efstratios Vakirlis
Adi Vaknin-Dembinsky
D. Vakula
Samuel Valable
Giuseppe Valacchi
Maria Pia Valacco
Saba Valadkhan
Jarmo Valaja
Sebastian Valanko
Muriel Valantin-Morison
Alan Valaperti
Rubén Valbuena
Nikola Valchev
Adriana Valcu
Pablo Valderrabano
Augusto Valderrama-Aguirre
Ángel Valdés
Jesús Valdés
Juan Valdés
André Valdez
Gregorio Valdez
Rupa Valdez
Luis Alonso Valdez Aguilar
Vasilis Valdramidis
Nuno Vale
Giampiero Valè
Marion Valeix
Eduardo Valencia-Cantero
Peter Valent
Kim Valenta
Patrícia Valentão
André Valente
Carina Valente
Christine Valente
Rubia Valente
Thomas Valente
Luca Valenti
Alexandra Valentin
Helene Valentin
Benjamin Valentine
Katherine Valentine
David Valentiner
Alessio Valentini
Alice Valentini
Elia Valentini
Maria Valentino
Verônica Valentinuzzi
Marie Valenza
C. Fernando Valenzuela
Rodrigo Valenzuela
David Valenzuela-Galván
Elvira Valera
Fabiana Valera
Linda Valeri
Massimiliano Valeriani
Cristina Valero
M. Adela Valero
Myriam Valero
Francisco Valero-Cuevas
Fernando Valiente
Claire Valiente Moro
Clarissa Valim
Anna-Liisa Välimaa
Vesa Välimäki
Liora Valinsky
Mindaugas Valius
Suresh Valiyaveettil
Alireza Valizadeh
Priit Väljamäe
Sofie Valk
Sándor Valkai
Vyara Valkanova
Francesc Vallderiola
Denise Valle
Mario Valle
Luis Valledor

Alberto Vallejo
Edgar Vallejo
Jody Vallejo
Mario Vallejo
Maite Vallejo
Daniel Vallero
Astrid Valles
Soraya Valles
Antonino Vallesi
Patrick Vallet
Maria Beatrice Valli
Heather Vallier
Marta Vallino
Giorgio Vallortigara
Josep Valls-Sole
Ravi Valluru
Nuria Vallverdú Coll
Nakul Valsangkar
Matteo Valsecchi
Paola Valsecchi
Sergio Valsecchi
Valeria Valsecchi
Deltcho Valtchanov
Z. Valuckiene
Anna Valujskikh
Ángela Valverde
Aránzazu Valverde
Rodrigo Valverde
Cinzia Valzania
Xenia Vamvakoussi
Jan van Aardt
Heleen Van Acker
Ronny van Aerle
Robbie van Aert
Alexander van Akkooi
Jason Van Allen
Mike Van Amburgh
Ari Van Assche
Thea van Asselt
Marcel van Assen
Nienke van Atteveldt
Anneloes Van Baar
Margriet van Baar
Peter van Baarlen
Sunshine Van Bael
Françoise Van Bambeke
Femke van Beek
Hans van Beek
Robert van Beers
Ilja Van Beest
Frank van Bel
Nicole Van Bergen
M. van Berkel
Yvette van Beurden
Elisabeth Van Beveren
Philip van Beynen
Wim Van Biesen
Jonathan Van Blerkom
Marka van Blitterswijk
Thomas Van Boeckel
Leonieke C. van Boekel
Job van Boven
Cornelis van Breemen
Johanna van Breugel
Ariena van Bruggen
Jaap van Buul
Olivier Van Caenegem
Steven Van Cruchten
Jeroen van Cutsem
Ulrike Van Daele
Loes Van Dam
Rob van Dam
Martijn van de Bunt
Tim van de Hoef
Mart van de Laar
Roel van de Laar
Thijs van de Laar
Pierre-Francois Van de Moortele
Fredrik Van de Steen
Dominique Van de Velde
Thomas Van de Velde
Vincent van de Ven
David van de Vijver
Fons Van de Vijver
Hans van de Vis
Dedmer Van de Waal
Gerlinde Van de Walle
Steven Van de Walle
Peter Van De Weijer
Koen van de Wetering
Janneke van de Wijgert
Jan Van de Zande
Tim Van Deelen
Johannes van Delden
Thierry Van den Abbeele
Guido Van den Ackerveken
Eline van den Akker
Erica van den Akker
Elske Van den Akker-van Marle
Marius van den Beek
Patrícia van den Bemt
Bert Van Den Berg

Ronald van den Berg
Thomas van den Berg
Timo van den Berg
Hubert van den Bergh
Carissa van den Berk-Clark
Peter Van den Besselaar
Twan van den Beucken
Erika van den Bogaart
Wilma van den Boogaard
Tobi Van den Bossche
Henry van den Brand
Marianne van den Bree
Rob H. S. van den Brink
Gesa van den Broek
Sofie Van Den Broucke
Peter J. van den Elsen
Leigh van den Heuvel
Tim van den Heuvel
Agnes van den Hoogen
Bernadette van den Hoogen
Wolbert van den Hoorn
Wilbert van den Hout
Daniel van den Hove
Jan Van den Stock
Daniel Van Denderen
Gert Van der Auwera
Alex van der Bliek
Elisabeth van der Elst
Arie van der Ende
Markus van der Giet
Brandon Van Der Heide
Erik van der Heijden
Marcel van der Heijden
Julie Van der Hoop
Björn van der Hoort
Charlie van der Horst
Geertje van der Horst
Irene van der Horst-Bruinsma
Mathieu van der Jagt
Mart van der Kam
Ida J. van der Klei
Esther van der Knaap
Casper van der Kooi
Katinka van der Kooij
Peter van der Kraan
Hugo van der Kuy
Antoinette van der Kuyl
Johannes van der Kwast
Martin van der Laan
Marike van der Leeden
Peter Van Der Ley
Jeannie Van der Linde
C. van der Linden
Dimitri van der Linden
Mark van der Linden
Sander van der Linden
Silvère van der Maarel
Marieke van der Maaten-Theunissen
Nathalie van der Mee-Marquet
Felix Van der Meer
Marcel T.J. van der Meer
P. F. van der Meer
Peter van der Meer
Paola van der Meijden
Margaretha Van der Merwe
Nicolaas van der Merwe
Jan Van der Meulen
Ans Van der Ploeg
Barbara Van Der Pol
Sueli Van Der Sand
Ellen van der Schoot
Yvonne van der Schouw
F. Josef van der Staay
Nele Van Der Steen
Nathan van der stoep
Catherine Van Der Straeten
Freddy van der Veen
Eric van der Veer
Jorien van der Velde
Adrianus van der Velden
Alike van der Velden
Peter G. van der Velden
Sanne van der Ven
Albert van der Vliet
Marjolein van der Waal
S. van der Waal
Judith Van Der Waerden
Philip Van der Wees
A. J. van der Wekken
Tjip van der Werf
Donne van der Westhuizen
Julie van der Zee
Wietske Van der Zwaag
B. C. van der Zwaard
Allen Van Deynze
Anne D. Van Diepeningen
David van Dijk
Hans van Dijk
J. Van Dijk
Jacqueline van Dijk
Jitse van Dijk
Koene Van Dijk

Kor-jent van Dijk
Marie van Dijk
Jan Maarten van Dijl
Craig van Dolleweerd
Josanne van Dongen
Thijs van Dongen
Karine Van Doninck
Coby van Dooremalen
Bryce Van Doren
Bieke Van Dorst
Joram van Driel
Cornelis van Drunen
Karel van Duijvenboden
D. van Duin
Eelco van Duinkerken
Bram Van Dun
Luca van Duren
Delfien Van Dyck
Linda van Dyk
Gerhild van Echten-Deckert
Herman J. van Eck
Nees Jan van Eck
Laura Van Eerd
Casper van Eijck
Maureen van Eijnatten
Evelien Van Ekert
Wouter van Elmpt
Peter van Endert
Nick van Es
Peter van Esse
Eddie van Etten
Hanneke van Ewijk
Armorel van Eyk
Eric van Ganse
Geert-Jan van Geffen
Celia Van Gelder
Russell Van Gelder
Teun van Gelder
Martin J. C. van Gemert
Dik van Gent
Robert-Jan van Geuns
Frédérique Van Gijsegem
Jo Van Ginderachter
Bram van Ginneken
Nadja van Ginneken
Stefaan Van Gool
Harry van Goor
Johannes van Goudoever
Johan van Griensven
Roy van Grunsven
William van Grunsven
Bethany Van Guelpen
Peter Van Haastert
Guido Van Hal
Wim van Harten
Wim Van Hecke
Waander van Heerde
Alastair Van Heerden
Henriette van Heerden
Loek van Heerebeek
Tristan van Heijst
Mieke Van Hemelrijck
Martijn van Hemert
Saskia van Hemert
Jo Van Herwegen
Charlotte Van Herzeele
Rob van Hest
Marieke van Heugten
Albert Jan van Hoek
Monique van Hoek
Sofie Van Holle
S. Van Hooft
Jorien van Hoorn
Lee Van Horn
Rene van Hout
Noemi Van Hul
Eric van Hullebusch
Marc van Iersel
Marinus Van IJzendoorn
Luc Van Kaer
Irene van Kamp
Michiels van Kessenich Laurens
Ellen van Kleef
Esther van Kleef
Joshua van Kleef
Roel van Klink
G. Cornelis van Kooten
Joost van Kordelaar
Silvester van Koten
Erik van Kuijk
Kristel Van Laethem
Ron van Lammeren
Laurianne Van Landeghem
Jacques van Lankveld
Coretta van Leer-Buter
Anieke van Leeuwen
Florian Van Leeuwen
Frank van Leeuwen
Karen M. van Leeuwen
Sonja van Leeuwen
Thed van Leeuwen
Theo van Leeuwen

Thomas Van Leeuwen
Travis Van Leeuwen
Geneviève van Liere
Johannes van Lieshout
Anita van Loenhoud
Andy Van Loocke
Gunther van Loon
Rob C. van Lummel
Carine Van malderen
Wouter van Marken Lichtenbelt
Harm van Marwijk
David Van Mater
Belle L. van Meer
Marieke van Meggelen
J. P. Richard van Merkesteyn
Tim Van Mieghem
Hanneke van Mier
Saskia van Mil
Margo van Mol
Dirk van Moorselaar
Kim Van Naarden Braun
Peter Van Ness
Ed van Niel
Martine van Nierop
Erik van Nieukerken
Cornelis Van Noorden
Arie van Noordwijk
Meine Van Noordwijk
Joy Van Nostrand
Rene van Oerle
Kees Van Oers
Pim Van Ooij
Joep van Oorschot
Ralph van Oort
Filip Van Opstal
Thijs van Osch
Yvette van Osch
Hans Van Ososterwyck
Leonard van Overbeek
Martin van Overveld
Frank Van Overwalle
Jaap van Pelt
Stan van Pelt
Wilfrid van Pelt
Sven Van Poucke
Joffrey van Prehn
Robb Van Putte
Ingrid Van Putten
Jos van Putten
Michel van Putten
Daniel van Raalte
Jeremy Van Raamsdonk
Anthony van Raan
Terry Van Raay
Carien van Reekum
Bas van Rhijn
Annelies Van Rie
Ronald Van Rij
Piet van Rijn
Leonie van Rijt
Dee Van Riper
Willeke van Roon-Mom
Diane van Rooy
Michele Van Rooyen
Marion van Rossum
Dimitri Van Ryckeghem
Dirkjan van Schaardenburg
Erin van Schaik
Daan Van Schalkwijk
Hein van Schie
Joseph Van Sickels
Ard van Sighem
Esther van Sluijs
Nienke van Staaveren
John Van Stan II
Gregory Van Stavern
Maarten Van Steenberge
H. W. van Steenbergen
Barbara Van Straaten
Arco Van Strien
Tatjana Van Strien
Bruno van Swinderen
Thomas van 't Erve
Jonathan van Tam
Edwin van Teijlingen
Celine Van Themsche
Herman van Tilbeurgh
Wijnand van Tilburg
Ronald van Toorn
Joyce van Tunen
Janet Van Uem
Peter van Veelen
Mirjam van Veen
Pieter Van Vlierberghe
Sandra van Vliet
Marieke van Vugt
Aren van Waarde
Robert van Waardenburg
Carter Van Waes
David Van Wagoner
Michiel van Weeghel
Rene van Weeren

Eduard van Wijk
Erwin van Wijk
Femke van Wijk
Guido van Wingen
Arie Jan van Winkelhoff
Laura Van Winkle
Hannes van Wyk
Nico van Zandwijk
Arthur van Zanten
Marta van Zanten
Catherine van Zelst
Wieske van Zoest
Arjan van Zuilen
André A.J. Van Zundert
Gert van Zyl
Jiri Vana
Markus Vanackeren
Nicola Vanacore
Maddi Vanaja
Abi Vanak
Olivier Vanakker
Christine VanBeek
Davy Vancampfort
David Vance
Jason Vance
Vicki Vance
Marie Vancová
Ivana Vancurova
Mark VanDam
Anne-Mieke Vandamme
Katleen Vandamme
Pierre Vandamme
Timon Vandamme
Bolormaa Vandanmagsar
Venu Gopal Vandavasi
Jonathan Vande Geest
Ina Vandebroek
Ilse Vandecandelaere
Grégoire Vandecasteele
Laurence Vandel
Corneel Vandelanotte
Jozef Vanden Broeck
Peter Vandenabeele
Laura Vandenberg
David Vandenbergh
Wim Vandenberghe
Roosmarijn Vandenbroucke
Tineke Vandenbroucke
Christina Vandenbroucke-Grauls
Freija Vandendriessche
Élodie Vandenhaute
Philippe Vandenkoornhuyse
Yvan Vandenplas
Patrick Vandeputte
Craig Vander Kooi
Robert Vander Stichele
Rachel Vanderlaan
Johan VanderMeer
John Vandermeer
Ashley Vandermorris
Rodney Vanderploeg
Rebecca Vanderpool
Alain Vanderpoorten
Nele Vandersickel
Deborah Vanderveen
Anthony Vandervoort
A.J. Vanderzanden
Pierre Vanderzwalmen
Stefanie Vandevijvere
Pamela VandeVord
Andre Vandierendonck
Alain Vandormael
Ruurd M. Vanelburg
Odelu Vanga
Siri Vangen
Lynn Vanhaecke
Petr Vanhara
Dimitri Vanhecke
Raymond Vanholder
Paul Vanhoutte
Janaki Vani
Nongnuch Vanittanakom
Hugo Vankelecom
Joann Vankessel
Radomira Vankova
Greg Vanlerberghe
Tim Vanmierlo
Richard Vann
Elizabeth Vanner
Jennifer Vannest
Joel Vanneste
Steffen Vanneste
Yvonne Vanneste
Ester Vanni
Edouard Vannier
Jean Vannier
Michael Vannier
Thuva Vanniyasingam
Alessandro Vannucchi
Maria Giuliana Vannucchi
Erik Vansebille
Caroline VanSickle

Ralph Vanstreels
Anna van't Hoog
Stephen Vantassel
Linda Vantil
Vincent Vantrepotte
Mirjam Vantricht
Eline Vanuytrecht
Dolors Vaqué
Eva Vaquero
Vinay Varadan
Kulandaiappan Varadaraj
Kartik Varadarajan
Navin Varadarajan
Sudhahar Varadarajan
Jasmina Varagic
Pietro Varaldo
Soory Varambally
Hacer Dogan Varan
Cristina Varas-Lorenzo
Chiara Varazzani
Szabolcs Várbíró
Konstantinos Vardakas
Trupti Vardam
Vivek Vardhan
Carlos Varea
Cristian Varela
Joao Varela
Mariana Varela
Ruben Varela
Pablo Varela-Centelles
Isabel Varela-Nieto
Armando Varela-Ramirez
Andrea Varella
Marco Varella
Jaroslava Varella Valentova
Cristina Varese
Luigi Varesio
Gabor Varga
Lilian Varga
Peter Varga
Sandra Varga
Zoltan Varga
Zsuzsanna Varga
Ashley Vargas
Hernan Vargas
Gilberto Vargas-Alarcón
Diego Vargas-Inchaustegui
Rosa Vargas-Poussou
María I. Vargas-Rojas
Neil Vargesson
Jishy Varghese
Kucku Varghese
Leonard Varghese
John A. Vargo
Ebrahim Variava
Brian Varisco
Markku Varjosalo
Tracey Varker
Ajit Varki
Anupam Varma
Ashok Varma
Dileep Varma
Disha Varma
Sameer Varma
Vivek Varma
Mandira Varma-Basil
Arul Mozhy Varman
Aniko Varnai
Dickson Varner
Kurt Varner
Onur Varol
Serena Varotto
Antonio Varriale
Lilian Varricchio
Arvind Varsani
Raunak Varshney
Frantisek Vasa
I. A. Vasalos
Ashwin Vasan
Senthil Vasan
Sonia Vasconcelos
Tiago Vasconcelos
Marmar Vaseghi
Slava Vasenev
Paul L. Vasey
Arti Vashist
Sandeep Vashist
Himanshu Vashistha
Michael Vasil
Efi Vasileiou
Catalin Vasilescu
Dragos Vasilescu
V. G. Vasilikos
Nikolaos Vasiloglou
Pavlos Vasilopoulos
Shravan Vasishth
Michael Vasko
Bruno Vaslin
Maite Vaslin
Karen Vasquez
Anna Vassall
Massimo Vassalli

Neville Vassallo
Paolo Vassallo
R. Vassallo
Francesca Vassanelli
R. Vassena
Nikon Vassilakos
Dimitrios Vassilopoulos
Michele Vasso
Evangelos Vassos
Gerardo Vasta
Peter Vasterman
Ramesh Vasudeva
Dileep Vasudevan
Erin Vasudevan
Shobha Vasudevan
Olena Vatamaniuk
Mohammad Vatanparast
Deniz Vatansever
Eric Vatikiotis-Bateson
Alessandro Vatrella
Pankaj Vats
Vatsalya Vatsalya
Matteo Vatta
Lars Vatten
Hartmut Vatter
Thomas Vatter
Gaetano Vaudo
David Vaudry
Adam Vaughan
Catherine Vaughan
Gilberto Vaughan
Kerrie Vaughan
Roxanne Vaughan
Ted Vaughan
Charlotte Vaughn
Leigh Vaughn
Thomas Vaughn
Sophie Vaulont
Marie-Noelle Vaultier
Dominique Vautier
David Vauzour
Divya Teja Vavilala
George Vavougios
Vasileios Vavourakis
Reaz Vawda
Effy Vayena
Amaury Vaysse
Frederic Vaysse
Fatima Vaz
Leandro Vaz
Marco Vaz
Emilie Vazeille
Jayesh Vazirani
Alberto Vazquez
Guillermo Vazquez
José Antonio Vazquez
Miguel Vazquez
Felisa Vazquez-Abad
Carlos Arturo Vázquez-Chacón
Javier Vazquez-Corral
Francisco Gabriel Vázquez-Cuevas
Rafael Vazquez-Duhalt
Felipe Vazquez-Flota
José Luis Vázquez-Ibar
Hector Vazquez-Leal
Roberto Vazquez-Padron
Andres Vazquez-Torres
Peter Vdacný
Jaimie Veale
Elizabeth Veasey
Ashley Veatch
Sarah Veatch
Christian Veauthier
Ronald Veazey
Chiara Vecchi
Carmine Vecchione
Raffaele Vecchione
Cecilia Vecoli
Laszlo Vecsei
Rajani Ved
Srinivasan Vedantham
Rajesh Vedanthan
Oscar Vedder
Anni Vedeler
Maria Cristina Vedovati
Fernand Vedrenne
Michael Veeman
Brendan Veeneman
Gerry Veenstra
Jan Veenstra
Ilya Veer
Arumugam Veera Ravi
Yaligara Veeranagouda
Sudhakar Veeranki
Harini Veeraraghavan
Janne Veerbeek
J. Lennert Veerman
Ainhoa Vega
Almudena Vega
Cristina Silveira Vega
Marco Vega López
Elisabetta Vegeto

Federico Vegetti
Fabrizio Veglia
Vic Veguilla
Pat Vehrs
Ana Veiga
Ana Salome Veiga
Patrick Veiga
Almudena Veiga-Lopez
Carla Veigas
Courtney Veilleux
Ralf Veit
Paul Veith
Daniela Vejrazkova
Ian Vela
Sitaram Velaga
Frederic Velard
Roser Velarde
Eladio Velasco
Santiago Velazco
Carlos Velazquez
Miguel Velazquez
Aldrik Velders
Jet Veldhuijzen van Zanten
Maria Veldhuizen
Stephanie Veleasquez
Emir Veledar
Louiza Velentzis
Juan Carlos Velez
María Vélez
Valerya Velezheva
Phillipp Velicky
David Veliz
Emiliya Velizarova
Tony Velkov
Alfred Vella
Stefano Vella
Sukumar Vellakkal
Priyathama Vellanki
Sandra Velleman
Licio Velloso
Artur Veloso
Kiran Kumar Velpula
Vijayakumar Velu
P. Velusamy
Kalyana Veluvolu
Geeta Vemuganti
Sai Vikram Vemula
Emilie Venables
Patrick Venail
Mikko Venäläinen
Miguel Vences
Sofia Venceslau
Anders L. Vendelboe
Vincenzo Venditti
Venkata Pulla Rao Vendra
Koen Venema
Monica Venere
Tiziana Venesio
David Venet
Fabienne Venet
Christos Venetis
Fotini Venetsanou
Robert Venette
Viktoria Venglovecz
Avner Vengosh
Robert Venick
Domenica Veniero
Sreedhar Venkannagari
Arun Venkataraman
Gayatri Venkataraman
Narayanan Venkatesan
Ajaybabu Venkatesan Pobbati
Bala Venkatesh
K. Venkatesh
Swaminathan Venkatesh
Muthusubramanian Venkateshwaran
Venkat Venkateswaran
Chamcha Venkateswarlu
Gopinath Venkatraman
Subbu Venkatraman
Vishwanath Venketaraman
Jason Venkiteswaran
Balu Alagar Venmathi Maran
Venugopal Reddy Venna
Michel Vennetier
Conchita Vens
Bruno Ventelou
Andre Venter
Estelle Venter
Henrietta Venter
Stephanus Venter
Nicholas Ventham
Maximo Vento
Gary Ventolini
Iván Ventoso
Alicia Ventresca Miller
Alexander Ventura
Ana Ventura
Francesc Ventura
Marco Ventura
Nicole Ventura
Walter Ventura

Javier Ventura-Juarez
Massimo Venturelli
Vayalam Venugopalan
Aldo Venuti
Charles Venuto
Jaime Vera
Lucio Vera-Cabrera
Rosario Vera-Estrella
Jennifer Verani
Michelle Verant
John Veranth
Jorge Vera-Otarola
Arturo Vera-Ponce De León
Paulo Verardi
Enrique Vera-Remartinez
Dineke Verbeek
Marcel Verbeek
Mark Verbraak
Johan Verbraecken
Stefaan Verbruggen
Niels Verburg
Peter Verburg
Dave Verbyla
Lavern Vercaigne
Alessandro Vercelli
Bruce Verchere
Luciana Vercoza Viana
Nuria Verdaguer
Zoraida Verde
Elisabetta Verderio Edwards
Claude Verdier
Kristien Verdonck
Jose Verdu
Iris Vered
Marilena Vered
Danny Vereecke
Balazs Veres
Daniel Veres
Gabor Veres
Laura Verga
Laura Vergani
Leoncio Vergara
Margarita Vergara
Natalia Vergara
Marta Vergara-Martínez
Giuseppe Vergaro
Pierre Verger
Joe Verghese
Flavia Verginelli
Gilles Vergnaud
Alain Vergnenegre
Pascale Vergne-Salle
Nathalie Vergnolle
Remi Vergnon
Elisabeta Vergu
Cristobal Vergudo
Joost Verhaagen
Pauline D.H.M. Verhaegen
A.P. Verhagen
Céline Verheggen
Kenneth Verheggen
Bram Verheijen
Steven Verhelst
Kristen Verhey
G. Verheyden
H. Verheyden
Linda Verhoef
Geert Verhoeven
Koen Verhoeven
Chris Verhofstede
Paulo Verissimo
António Veríssimo
Georges Verjans
Sergio Verjovski-Almeida
Erwin Verkade
Annemarie Verkerk
Kurt Verkest
Gennady Verkhivker
Alexey Verkhovtsev
Maykel Verkuyten
Jorrit-Jan Verlaan
Giovanna Verlato
Geert Verleden
Stijn Verleden
Hugo Verli
Christophe Verlinde
Wim Verlinden
Maite Verloigne
Amrisha Verma
Ashutosh Verma
Chaman Verma
Mayank Verma
Nitin Verma
R.K. Verma
Raj Verma
Rajkumar Verma
Rama Verma
Ruchi Verma
Subhash Verma
Sunil Verma
Vikash Verma
Katrien Vermeire

Lance Vermeire
Yannick Vermeiren
Ivar Vermeulen
David Vermijlen
Dolinsky Vern
Pietro Vernazza
Cristiano Vernesi
Gianluca Vernillo
Robin W.M. Vernooij
Marc Verny
Eve Veromann
Nicola Veronese
Silvio Veronese
Fabio Veronesi
Giovanni Veronesi
Giulia Veronesi
Brixner Veronika
Pierfrancesco Veroux
Franck Verrecchia
François Verrey
Bernard Verrier
Francesco Versace
Admar Verschoor
Eric Verschooten
Vytas Verselis
Paul Verslues
Lilly Verso
Karin Verspoor
Henri Versteeg
Hans Verstraelen
Kevin Verstrepen
Tim Verstynen
Timothy Verstynen
Jukka-Pekka Verta
Ilse Verveer
Suzanne Verver
M.G. Vervloet
Eleni Verykouki
Roberto Verzicco
Omar Vesga
Michael Vesia
Paul Vespa
Umberto Vespasiani-Gentilucci
Jan Vesper
Stephen Vesper
Christian Vestergaard
Mette Vesterhus
Ville Vesterinen
Peter Veth
Davide Vetrano
Marina Vetrova
Céline Vetter
Stefan Vetter
Adrien Vetterli
Cristina Vettori
Kristen Veum
Gregory Vey
Bernard Veyret
Nicolas Veziris
Julien Vezoli
Paola Vezza
Loris Vezzali
Giuseppe Vezzoli
Esther Via
Michael Via
François Vialard
Aurélie Vialette-Guiraud
Denis Vialou
Duarte Viana
Mafalda Viana
Maria Teresa Viana
Michelangelo Vianello
Monique Vianey-Liaud
Monica Vianna
Alexander Viardot
Andreas Viardot
Arnaud Viarouge
Diego Viasus
John Viator
Rajeev Vibhakar
Stefania Vicari
Carmelo Vicario
Antonio Vicent
Guillermo Vicent
Cláudia Vicente
Isabel Vicente
Joana Vicente
João Vicente
Joaquin Vicente
Miguel Vicente
Miguel Vicente-Manzanares
Renato Vicentini
Elisa Vicenzi
Carlos Vicient
Peter Vickerman
Andrew Vickers
James Vickers
Joan Vickers
Kasey Vickers
T. Winston Vickers
Brian Vickery
Karen Vickery

Michele Vicovaro
T. Victoni
Benjamin Victor
Janine Victor
Jennifer Victor
Sarah Victor
David Victorson
Celine Vidaillac
Benedicto Vidal
Juan R. Vidal
Lital Vidal
Pierre-Paul Vidal
Ruben Vidal
Georgios Vidalakis
Emmanuelle Vidal-Petiot
Manuel Vidal-Sanz
Jose Vidal-Taboada
Paula Videira
Romeu Videira
John Videler
Eric Vidoni
Meghan Vidt
Sunil Vidya
Sharmili Vidyadaran
Stephanie Vie
Susana Viegas
Peter Viehoff
Ferdinand Vieider
Alexandre Vieira
Armando Vieira
Bruna Vieira
Cristina Vieira
Elaine Vieira
Fernando Vieira
Joana Vieira
Jorge Vieira
Marcus Vieira
Paulo Vieira
Rodolfo Vieira
Ana Catarina Vieira de Castro
Madalena Vieira-Pinto
Victoria Vieira-Potter
Marcos Viejo
Abel Viejo-Borbolla
Tania Viel
Philippe Vielh
Veronika Vielsmeier
Solveig Vieluf
Neal Viemeister
Cyril Vienne
Kari Vienola
Jeff Vierstra
Bruno Viertel
Dumitru Vieru
Michael Vieth
Adalberto Vieyra
Matheus Viezzer Bianchi
Adarsh Vig
Alessandro Viganò
Paola Viganò
Cheryl Vigen
Davide Vigetti
Andrea Viglino
Edward Vigmond
Rita Vignani
Federica Vigna-Taglianti
Nathalie Vigneron
Ramachandran Vignesh
Aglaia Vignoli
Rafael Vignoli
Irene Vignon-Clementel
Linda Vignozzi
Isabelle Vigon
Enrique Viguera
Catherine Viguie
Effie Viguiliouk
Kaido Viht
Tapani Viitala
Shruti Vij
Mathilakath Vijayan
Murali Vijayan
Vipin Vijayan
Sivakumar Vijayaraghavalu
Krishna Vijayaraghavan
Ponnuswamy Vijayaraghavan
Kiruthiga Vijayaraman
Shyan Vijayasekaran
Dhanasekaran Vijaykrishna
S. Vijaykumar
Matam Vijay-Kumar
Daniel Vijlbrief
Steven Vik
Prashant Vikram
Bjørn Egil Vikse
Kristina Viktorsson
Jordi Vila
Jose Vila
Peter Vila
Nuria Vilaboa
Carolina Vila-Cha
Nuria Viladrich
Alberto Vilagrosa

Gemma Vilagut
Laura Vilander
Guillermo Vilanova
Manuel Vilanova
Ester Vilaprinyo
Enric Vilar
Marçal Vilar
Raquel Vilar-López
David Vilchez
Juan J. Vilchez
Andreas Vilcinskas
Jose Vilela-Martin
Felip Vilella
Anna Vilgelm
Oddur Vilhelmsson
Alessandro Villa
Federica Villa
Francesco Villa
Gianluca Villa
Kira Villa
Paola Villa
Giselle Villa Flor Brunoro
Pedro A. Villablanca
Luis Villada
Jose Villalba
Alice Villalobos
Fernando Villalta
Frederick Villamena
Luc Villandré
Daniela Villani
Gianfrancesco Maria Villani
Andrea Villanti
Augusta Villanueva
Elmer Villanueva
Roger Villanueva
Iria Villar
Livia Melo Villar
Luisa Villar
Manuel Villaran
Marc-André Villard
Tracy Villareal
Carlos Villarreal
Francisco Villarreal
Maria Villarroel
Laura Villa-Torres
Saúl Villa-Treviño
Andrés Villaveces
Alejandro Villaverde
Valentin Villaverde
Nathalie Villa-Vialaneix
Camila P. Villavicencio
Yves Ville
Frederic Villebrun
Saul Villeda
Luis Eduardo Martinez Villegas
Daniel Villela
Josep Villena
Julio Villena
Sara Villén-Pérez
Pablo Villoslada
Sebastien Villotte
Andreas Villunger
Andres Vina
Catarina Vinagre
Lydia Vinals-Castonguay
Fabrizio Vinante
José-María Vinardell
Jordi Vinas
Manjula Vinayak
Delphine Vincent
Grace Vincent
Jean-Louis Vincent
Jeffrey R. Vincent
Maxence Vincent
Peter Vincent
Royce Vincent
Stephen Vincent
Bruno Vincenzi
Francesca Vinchi
Antonio Vinciguerra
Manlio Vinciguerra
Fabien Vinckier
Brigitte Vinçon-Leite
Aron Vincze
Éva Vincze
Veronika Vincze
Tabitha Viner
Jonathan Vinet
Kerri Viney
J.J.M. Vingerhoets
Martin Vingron
Ramachandran Vinitha
Cor Vink
Gerko Vink
Jacqueline Vink
C.H. Vinkers
Peter Vinkler
Sarah Vinnicombe
K.K. Vinod
Neomi Vin-Raviv
David Vinson
Jakob Vinther

Manuela Viola
Nerissa Viola-Villegas
Dirix Violette
Benoit Viollet
Julien Vionnet
Lorenzo Viora
Caroline Vipond
Sacha Viquerat
Pooja Vir
Jitka Virag
Attila Virág
Meta Virant-Doberlet
Irma Virant-Klun
Jugsharan Singh Virdi
Parminder Virk
Guillermo Virkel
Renu Virmani
David Virshup
Jouko Virtanen
Marianna Virtanen
Juan Viruel
Christiaan Vis
Daniel Vis
Pablo Visconti
Jeane Visentainer
Vicktoria Vishnevskia-Dai
Bannikuppe Sannanaik Vishwanath
Rohini Vishwanathan
Triinu Visnapuu
Shelina Visram
Christian Visscher
Henk Visscher
Bart Visser
Brendan Visser
Jenny Visser
Lydia Visser
Maretha Visser
Theo Visser
Frank Visseren
Giulio Vistoli
Hema Viswambharan
Pavithra Viswanath
Ganesh Viswanathan
Shiv Kumar Viswanathan
V. Viswanathan
Vijay Viswanathan
Alessandro Vitale
Augusto Vitale
Luca Vitale
Mario Vitale
Nicolas Vitale
Antonio Vitarelli
Martha Vitaterna
Libor Vitek
Michael Vitevitch
Stanislav Vitha
Meththika Vithanage
Carlo Viti
Ben Vitiello
Mariateresa Vitiello
Alena Vitova
Olivier Vitrac
Agnes Vitry
Ragini Vittal
Dario Vitturi
Patma Vityakon
Maria Vivanco
Fabio Vivarelli
Maria Viveiros
Miguel Viveiros
Perumal Vivekanandan
Juan Vivero-Escoto
Cristofol Vives-Bauza
Jay Vivian
Régis Vivien
Barbara Vizmanos-Lamotte
Denise Vizziano Cantonnet
Jiri Vlach
Konstantinos Vlachonasios
Marilena Vlachou - Konstantinidou
Viktorie Vlachova
Johan W.S. Vlaeyen
Petia Vlahovska
Just Vlak
Marija Vlaski
Anastasia Vlasova
Anestis Vlysidis
Ashley Vo
Nam Vo
Silja Vocks
Anthony Vodacek
Irena Vodenska
Ulrich Voderholzer
Yoram Vodovotz
Stefan Voegele
David Voegeli
David Voehringer
Florian Voelk
Moritz Voelker-Albert
Mirko Voelkers
Kerstin Voelz
Vincent Voelz

Natalie Voets
Adam Vogel
Britta Vogel
Christina Vogel
Gernot Vogel
Hans Vogel
Heiko Vogel
Jörg Vogel
Joseph Vogel
Lotte Vogel
Rachel Vogel
Tilley Vogel
Tobias Vogel
Mike Vogelbaum
Rufin Vogels
Bert Vogelstein
Ioannis Vogiatzis
Amy Vogler
Sabine Vogler
Andreas Vogt
Guillaume Vogt
Lars Vogt
Marjorie Vogt
Rolf Vogt
Stefanie Vogt
Emily Vogtmann
Cosmin Voican
Annette Voigt
Kerstin Voigt
Kristina Voigt
Mark Voigt
Michael Voigt
Robin Voigt
Stacy Voils
Cécile Voisset
Renate Voit
Steven Vokes
Florence Volaire
A.G. Volaklis
Robert Volcic
Filip Volckaert
John Volckens
Petr Volf
Jean-Nicolas Volff
David Volgas
Bela Volgyi
Ladislav Volicer
Gunnar Völkel
John Volkman
E. Volkmann
Tyson Volkmann
Vadim Volkov
Luba Volkova
Patricia Volkow
Niels Vollaard
Vaughan Voller
L. Vøllestad
Linda Vollmer
Sebastian Vollmer
Regina Vollmeyer
Fritz Vollrath
Maria Vollsæter
Elena Volodina
Ilya Volodyaev
Alexandra Voloshina
Elena Voloshina
Stefano Volpato
Elisabetta Volpe
Fernando Volpe
Olga Volpert
Iride Volpi
Laura Volpicelli-Daley
Rildo Volpini
Beth Volpov
C.A. Volta
Umberto Volta
Igor Volzhanin
Frederick Vom Saal
Nicolas von Alvensleben
Louisa von Baumgarten
Helene von Bibra
Clemens von Birgelen
Daniel von Bornstaed
Ysander von Boxberg
Christian von Buchwald
Noreen von Cramon-Taubadel
Peter von Dassow
Yasmin von Dassow
Bernadette Von Dawans
Andrea von Delft
Lisa Von Diemen
Arnold von Eckardstein
Eric von Elert
Alexander von Gise
Anne von Gottberg
Achaz von Hardenberg
Jost von Hardenberg
Maximilian von Heesen
Claes von Hofsten
Betsy Von Holle
Isabella von Holstein
Roland von Känel

Arvind Von Keudell
Max von Kleist
Andreas von Knethen
Maren von Köckritz-Blickwede
Kathrein von Kopylow
Michael Von Korff
Alex von Kriegsheim
Rüdiger von Kries
Marie von Lilienfeld-Toal
Erika von Mutius
Kristin von Ranson
Catherine von Reyn
Erik von Rosenvinge
Chris von Rueden
Clemens von Schacky
Malcolm von Schantz
Antje Von Suchodolotz
Martin von Websky
Eric von Wettberg
Penny von Wettstein-Knowles
Thomas von Zglinicki
Constantin von zur Muhlen
Linda Vona-Davis
Thomas Vondriska
Wanwipa Vongsangnak
Jennifer Vonk
Harald Vonkeman
Kimberly Vonnahme
Andrew Voorhees
Eric Voorn
Trudy Voortman
Mariana Voos
Gary Vora
Kranti Vora
Mehul Vora
Neil M. Vora
Christoph Vorburger
H. Martin Vordermeier
Charles Vorhees
Panagiotis Vorkas
Misha Vorobyev
Denis Voronin
Angelika Voronova
Thomas Vorup-Jensen
Dierk Vorwerk
Margreet Vos
Melissa Vos
Robin Vos
Miroslav Vosatka
Kenneth Voss
Martin Voss
Patrice Voss
S. Voss
Till Voss
Ulrikke Voss
Simone Vossel
Mark Vosvick
Mark Votruba
Michael Vouche
Patrick Vourc'h
Jovanka Voyich-Kane
Nicola Voyle
Bradley Voytek
A. Vozikis
Semir Vranic
Sudhanshu Vrati
Rachel Vreeman
Sarah Vreugde
Thom Vreven
Alice Vrielink
Chris Vriend
Gert Vriend
Jerry Vriend
Dennis Vriens
Joris Vriens
Sylvain Vrignon-Brenas
Bernard Vrijens
Christiaan Vrints
Sophie Vriz
Joyce Vromen
Susanne Vrtala
Olga Vsevolozhskaya
Giang Vu
Luka Vucemilo
Slobodan Vucetic
Domagoj Vucic
Steve Vucic
Aleksandra Vuckovic
Aline Vuckovic
Ivan Vuckovic
Sonja Vuckovic
Louis Vuga
Anthony Vugler
Eric Vugrin
Anne Vuillemin
Lazar Vujanovic
Nikola Vujanovic
Maja Vujic
Stela Vujosevic
Vladana Vukojevic
Vuk Vukovic
Vesna Vuksanovic

Rik Vullings
Eero Vuoksimaa
Ville Vuollo
Nguyen Lam Vuong
Ilkka Vuori
Anna-Leena Vuorinen
Claudia Vuotto
Dervis Vural
Laszlo Vutskits
Saleha Banu Vuyyuri
Ajai Vyas
Ami Vyas
Jatin Vyas
Sheela Vyas
Tomáš Vyhnánek
Jan Vymazal
Tony Vyn
Yuliya Vystavna
A.N. Vzorov
Cees Waalwijk
Peter Wabel
Dagmar Waberski
Colette Wabnitz
Sven Wach
Patrick Wachholz
Juddy Wachira
Matt Wachowiak
Michael Wachs
Mitchell Wachtel
Dagmar Wachten
Rolf Wachter
Thomas Wachtler
Margarethe Wacker
Werner Wackernagel
Bartlomiej Waclaw
Agnieszka Waclawik
Hideo Wada
Hiroshi Wada
Hisashi Wada
Jun Wada
Keiji Wada
Makoto Wada
Morimasa Wada
Naohisa Wada
Taira Wada
Takashi Wada
Takayuki Wada
Takehiko Wada
Thaddeus Wadas
Dwight Waddell
Simon Waddell
John Waddington
Jonathan Waddington
Susan Waddy
Alex Wade
Charles Wade
Djibril Wade
Mark Wade
Timothy Wade
Goran Wadell
Raj Wadgaonkar
Sunil Wadhwa
Bassem Wadie
Phillip Wadl
Lyn Wadley
Jonathan Wadsworth
Lucia Wadt
Christian Waeber
Lynn Waelde
Jamie Waese
Hazem Wafa
Francis Wafula
Yukiko Wagatsuma
Alexandre Wagemakers
Stuart Wagenius
Anton Wagenmakers
Jessica Wagenseil
Elizabeth Wager
Nolan Wages
Adrian Wagg
James Waggitt
Darrel Waggoner
Jesse Waggoner
Kshitij Wagh
Alpana Waghmare
Mahendra Wagle
Abram Wagner
Alan Wagner
Amy Wagner
Ana Wagner
Anjuli Wagner
Anne Wagner
Bradley Wagner
Brandie Wagner
Carol Wagner
Caroline Wagner
Carsten Wagner
David Wagner
Eric Wagner
Henry Wagner
John Wagner
Ludwig Wagner

Martin Wagner
Norman Wagner
Philipp Wagner
Robert Wagner
Ryan Wagner
Sarah Wagner
Sasha Wagner
Ulrich Wagner
William Wagner
David Wagner Jr.
Kaira Wagoner
Kristina Wagstrom
Konrad Wagstyl
Fazal Wahab
Rizwan Wahab
Grace Wahba
M. Wahb-Allah
Siegfried Wahl
Banrida Wahlang
Ayo Wahlberg
Magnus Wahlberg
Mats Wahlgren
Thomas Wahli
Walter Wahli
Victoria Wahl-Jensen
Lars-Olof Wahlund
Christine Wai
Clifford Wai
Sun Wai
Sushrut Waikar
Rebekah Waikel
Zev Wainberg
J. Waines
Charlotte Wainwright
Derek Wainwright
Mark Wainwright
Peter Wais
Dan Waisman
D. Wait
Marcelo Wajchenberg
Shadi Wajih Hasan
Jose Roberto Wajman
Ichiro Wakabayashi
Koich Wakabayashi
Taku Wakahara
Joe Wakano
Minako Wakasugi
Masaru Wakatsuki
Cameron Wake
Marvalee Wake
Imam Waked
Juliet Wakefield
Thomas Wakefield
Joanna Wakefield-Scurr
Ajay Wakhloo
Hironori Waki
Tewodros Wakie
Jun-ichi Wakita
Joseph Wakshlag
Monika Waksmundzka-Hajnos
Paulina Wakula
Harald Walach
Sibongile Walaza
Christopher Walcek
Cristy Walcher-Chevillet
Ori Wald
Peter Walde
Marcel D. Waldinger
Michael Waldman
Herman Waldmann
Thomas Waldrop
Philippe Waldteufel
Annemiek Walenkamp
Marie-Laure Walet-Balieu
Alicia Walf
Jibran Wali
Gagandeep Kaur Walia
Rasna Walia
Seth Walk
Alexander Walker
Ameae Walker
Amy Walker
Ann Walker
Anthony Walker
Ashley Walker
David Walker
Dilys Walker
Edward Walker
Ellen Walker
Faith Walker
Francis Walker
Glenn Walker
Grant Walker
Harrison Walker
Henry Walker
James Walker
Judy Walker
Kara Walker
Kerry Walker
Louise Walker
Mark Walker
Mary Walker

Naomi Walker
Nykia Walker
P. Walker
Rachel Walker
Robert Walker
Ryan Walker
Stephen Walker
Susan Walker
Valerie Walker
Vernon Walker
Virginia Walker
William Walker
Allan Walkey
Gernot Walko
Benjamin Wall
Brian Wall
Carrie Wall
Crystal Wall
Kristin Wall
Marlene Wall
Meaghan Wall
Peter Walla
Aaron Wallace
Bruce Wallace
Darren Wallace
David Wallace
Davin Wallace
Douglas Wallace
Gordon Wallace
I. R. Wallace
Jacqueline Wallace
Marita Wallace
Mark Wallace
Ryan Wallace
Vesna Wallace
Daniel Wallach
Jan Wallander
Solveig Wållberg-Jonsson
Mikkel Wallentin
Donald Waller
Ed Waller
Kimberly Waller
Brenda Walles
Shannon M. Wallet
Keith Walley
Elisabeth Wallhäusser-Franke
Jacco Wallinga
Märta Wallinius
Guy Wallis
Robert Wallis
Russell Wallis
Thomas Wallis
Björn Wallner
Fabrice Wallois
Anne Walls
Theodore Walls
Oliver Wallscheid
Owen Wally
A. Damien Walmsley
Julia Walochnik
Scott Walper
Adam Walsh
Allyn Walsh
Brian Walsh
Christopher Walsh
Conor Walsh
Eamonn Walsh
Elaine Walsh
Erin Walsh
Fiona Walsh
Garry Walsh
Ian Walsh
Kieran Walsh
Kyle Walsh
Martin Walsh
Paul Walsh
Simon Walsh
Stephen R Walsh
Tom Walsh
Zachary Walsh
Judd Walson
David Walt
Christi Walter
Fruzsina Walter
John Walter
Jonathan Walter
Michael Walter
Nicholas Walter
Steffen Walter
Uwe Walter
W. David Walter
Adam Walters
Arthur Walters
Dafydd Walters
Edgar Walters
Haydn Walters
Keith Walters
Michael Walters
Theresa Walters
Thomas Walters
Zoe Walters
Matthias Waltert

Bruno Walther
G. Walther
Sebastian Walther
Marina Walther-Antonio
Chad Walton
Gemma Walton
Noah Walton
William Walton
Wendy Walwyn
Christian Walzer
Jennifer Wambach
Joyce Wamoyi
Peter Wampler
Meredith Wampler-Kuhn
Chao Wan
Hong Wan
Jiafu Wan
Jian Wan
Jian-Bo Wan
Jian-Min Wan
Julian Wan
Lei Wan
Murphy Lam-Yim Wan
Wen Wan
Xia Wan
Xiu-Feng Wan
Xuehua Wan
Ying Wan
Yinsheng Wan
Yung-Liang Wan
Wan Nurazreena Wan Hassan
Wan Yusoff Wan Sulaiman
Johannes Wancata
Benedict Wand
Joshua Wand
Gilles Wandeler
Almir Wanderley
Francisco Wandosell
Aide Wang
Aiqin Wang
Alice Wang
Anran Wang
Baohua Wang
Baozhan Wang
Bao-Zhong Wang
Beixi Wang
Benquan Wang
Bin Wang
Bing Wang
Bingbing Wang
Bingbo Wang
Bo Wang
Boyuan Wang
C.W. Wang
Cailin Wang
Caixia Wang
Caiyun Wang
Can Wang
Chang-Fang Wang
Changqian Wang
Chao Wang
Chaofeng Wang
Chao-Yung Wang
Chen Wang
Chenghui Wang
Chengshu Wang
Chenguang Wang
Chen-Zhu Wang
Chi Wang
Chih-Chi Wang
Chih-Hung Wang
Chih-Jen Wang
Ching-Wei Wang
Ching-Yi Wang
Chong Wang
Chong-Wen Wang
Chongyun Wang
Christina Wang
Chrong-Reen Wang
Chuan Wang
Chuanxin Wang
Chun Wang
Chun-Chao Wang
Chunfeng Wang
Chunming Wang
Chunyan Wang
Chunyu Wang
Clay Wang
Con Yi Wang
Cong-Zhi Wang
Cuicui Wang
D. Wang
Dan-Li Wang
Danny Wang
Daowen Wang
Daryi Wang
David Wang
David Z. W. Wang
Dayong Wang
Da-Zhi Wang
Debby Wang
De-Hua Wang

Demin Wang
Deshou Wang
Dong Wang
Dongfang Wang
DongXiao Wang
Dong-Xin Wang
Duochun Wang
Edith Wang
Eunice Wang
Fahui Wang
Faliang Wang
Fan Wang
Fang Wang
Feilong Wang
Feng Wang
Fu Wang
Fudi Wang
Fun-In Wang
Fusheng Wang
Fuyi Wang
Gang-Jin Wang
Gangping Wang
Genxu Wang
Grace Wang
Greg Wang
Guan Wang
Guangce Wang
Guangshun Wang
Guangyu Wang
Guang-Zhong Wang
Guanyu Wang
Guijing Wang
Guirong Wang
Guixue Wang
Guochang Wang
Guojun Wang
Guoxing Wang
Guoye Wang
GuoZhen Wang
Haichao Wang
Haichen Wang
Hailing Wang
Haiping Wang
Haiyan Wang
Han-Ching Wang
Han-I Wang
Hao Wang
He Wang
Heming Wang
Hesheng Wang
Hong Wang
Hongbing Wang
Hongbo Wang
Hongjun Wang
Hongli Wang
Hongmei Wang
Hongning Wang
Hongran Wang
Hong-Sheng Wang
Hongwei Wang
Hongxia Wang
Hongyang Wang
Horng-Dar Wang
Hua Wang
Huafeng Wang
Huaimin Wang
Huaiyuan Wang
Hui Wang
Hui-Cong Wang
Huifang Wang
Huijuan Wang
Hui-Min Wang
Hui-Yu Wang
Huizhi Wang
Hung-Jung Wang
J.W. Wang
Jann-Tay Wang
Jaw-Yuan Wang
Jean Wang
Jeff T.H. Wang
Jen-Ren Wang
Jessica Wang
Ji Wang
Jia Wang
Jia-Bo Wang
Jiadong Wang
Jialiang Wang
Jian Wang
Jianchuan Wang
Jiangfeng Wang
Jianglin Wang
Jianguo Wang
Jianhua Wang
Jiankang Wang
Jianke Wang
JianLi Wang
Jianmin Wang
Jianming Wang
Jiano Wang
Jianwei Wang
Jianwen Wang
Jianxin Wang

Jiaojian Wang
Jiaqi Wang
Jiayi Wang
Jia-Yi Wang
Jiejing Wang
Jieqiong Wang
Ji-Guang Wang
Jih-Terng Wang
Jihua Wang
Jin Wang
Jinfeng Wang
Jin-Feng Wang
Jing Wang
Jing-Feng Wang
Jing-Houng Wang
Jingkuan Wang
Jingping Wang
Jingyun Wang
Jinhui Wang
Jinjun Wang
Jin-Jun Wang
Jin-Town Wang
Jinyan Wang
Jinyang Wang
Jinzhou Wang
Jiqiu Wang
Jiquan Wang
Jiu Yao Wang
Jiu-Feng Wang
Jiun-Ling Wang
John Wang
Joshua Wang
Juan Wang
Judong Wang
Jue Wang
Juexiao Wang
Julia Wang
Jun Wang
Junbai Wang
Jung-Der Wang
Junhui Wang
Junjie Wang
K. Wang
Kai Wang
Kan Wang
Kanix Wang
Ke Wang
Kehua Wang
Kejian Wang
Ketong Wang
Kevin Wang
Kimberley Wang
Kun Wang
Lai-Shuan Wang
Lanqing Wang
Lei Wang
Leon Wang
Lezhi Wang
Li Wang
Liang Wang
Liang-Jen Wang
Liansheng Wang
Liaoyuan Wang
Libing Wang
Lifeng Wang
Liguo Wang
Lihui Wang
Lijun Wang
Limin Wang
LiMing Wang
Lin Wang
Lina Wang
Ling Wang
Lingxiang Wang
Ling-Zhi Wang
Lin-Jie Wang
Liping Wang
Li-San Wang
Lisha Wang
Lizhong Wang
Lu Wang
Lubin Wang
Lubing Wang
Luqiao Wang
Maggie Haitian Wang
Man-Qun Wang
Man-Tzu Wang
Marilene Wang
Meng Wang
Menghua Wang
Miao Wang
Michael Wang
Min Wang
Ming-Shan Wang
Mingyi Wang
Minxian Wang
Mo Wang
Na Wang
Nan Wang
Nian Wang
Ning Wang
Ningli Wang

Ningning Wang
Ningtao Wang
P. Jeremy Wang
Pa-Chun Wang
Panwen Wang
Pei Wang
Peigang Wang
Pei-Hui Wang
Pei-Ning Wang
Peng Wang
Ping Wang
PoJen Wang
Pu Wang
Qi Wang
Qian Wang
Qian-Fei Wang
Qiang Wang
Qiaochun Wang
Qiaomei Wang
Qi-En Wang
Qike Wang
Qin Wang
Qing Wang
Qingfeng Wang
Qinghong Wang
Qingwen Wang
Qingyun Wang
Qiong Wang
Qiongxin Wang
Qiuhong Wang
Qiwei Wang
Quan Wang
Qun Wang
Ran Wang
Ranran Wang
Renhou Wang
Renxiao Wang
Robert Wang
Rong Wang
Rowan Wang
Rufeng Wang
Rui Wang
Ruijia Wang
Ruikang Wang
Ruisi Wang
Ruo Yu Wang
Sa Wang
Seok Mui Wang
Sha Wang
Shanfeng Wang
Shaocheng Wang
Shaoli Wang
Shaolin Wang
Shaoying Wang
Sheng Wang
Shengzhang Wang
Shen-Nien Wang
Shen-Yung Wang
Shibo Wang
Shichen Wang
Shih-Kai Wang
Shih-Tien Wang
Shih-Wei Wang
Shiqi Wang
Shixuan Wang
Shouyi Wang
Shu Wang
Shuang Wang
Shuangyin Wang
Shu-Hua Wang
Shuihua Wang
Shukui Wang
Shuo Wang
Shutao Wang
Shuxia Wang
Shuying Wang
Sibao Wang
Songjie Wang
Steven S.S. Wang
Su Wang
Suge Wang
Su-hua Wang
Su-Jing Wang
Su-xia Wang
Tai Wang
Taia Wang
Tao Wang
Tian Wang
Tian-fang Wang
Ting Wang
Ting-Fang Wang
Tingting Wang
Tong Wang
Tongguang Wang
Tong-Hong Wang
Tuanlao Wang
Tzu-Hua Wang
Tzung-Dau Wang
Tzu-Wei Wang
W. Wang
Wan Wang
Wangsheng Wang

Wei Wang
Weijie Wang
Wei-Lien Wang
Wei-Zhong Wang
Wen Wang
Wen-Der Wang
Wendi Wang
Wen-Hui Wang
Wenjie Wang
Wen-Jun Wang
Wenru Wang
William Shi-Yuan Wang
Wuli Wang
XianBing Wang
Xiang Wang
Xiangdong Wang
Xianling Wang
Xianqin Wang
Xianwei Wang
Xianwen Wang
Xiao Wang
Xiaodan Wang
Xiaodong Wang
Xiaofang Wang
Xiaofeng Wang
Xiaogang Wang
Xiaohong Wang
Xiaojing Wang
Xiaok Wang
Xiaoli Wang
Xiaonan Wang
Xiaoping Wang
Xiao-Qi Wang
Xiaowei Wang
Xiaoyan Wang
Xiaozhong Wang
Xifeng Wang
Xihua Wang
Ximing Wang
Xin Wang
Xingfen Wang
Xinglong Wang
Xingyuan Wang
Xingzheng Wang
Xinjiang Wang
Xinlei (Sherry) Wang
Xinping Wang
Xinyu Wang
Xiue Wang
Xiu-Jie Wang
Xiuli Wang
Xiuqing Wang
Xiurong Wang
Xiyang Wang
Xiyin Wang
Xu Wang
Xuan Wang
Xuannian Wang
Xuchu Wang
Xudong Wang
Xuede Wang
Xuefeng Wang
Xuejun Wang
Xuemei Wang
Xuewen Wang
Xuwen Wang
Xu-Wen Wang
Yafei Wang
Yajing Wang
Yan Wang
Yan-Chun Wang
Yan-Dong Wang
Yang Wang
YangGang Wang
Yang-Kao Wang
Yanhui Wang
Yanjun Wang
Yanru Wang
Yanshan Wang
Yaqun Wang
Ya-Wen Wang
Ya-Yun Wang
Yejun Wang
Yen-Feng Wang
Yen-Po Wang
Yi Wang
Yi Eva Wang
Yibin Wang
Yichen Wang
Yi-Dong Wang
Yigang Wang
Yi-Hong Wang
Yihua Wang
Yijie Wang
Yijun Wang
Yi-Lei Wang
Yin Wang
Ying Wang
Yingchun Wang
Yinshan Wang
Yipeng Wang
Yi-Ping Wang

Yiran Wang
Yitang Wang
Yiwei Wang
Yiwen Wang
Yizhen Wang
Yizhong Wang
Yong Wang
YongBo Wang
Yongchao Wang
Yongguang Wang
Yonghua Wang
Yongjun Wang
Yonglin Wang
Yongqin Wang
Yongsheng Wang
Yongxiang Wang
Youping Wang
Youwei Wang
Yuan Wang
Yuanchao Wang
Yuandi Wang
Yuanguo Wang
Yuanhong Wang
Yuan-Hung Wang
Yuanpeng Wang
Yuanzhi Wang
Yu-Chao Wang
Yucheng Wang
Yue Wang
Yuesong Wang
Yuexia Wang
Yufeng Wang
Yuguang Wang
Yuhong Wang
Yujie Wang
Yu-Jin Wang
Yulan Wang
Yumin Wang
Yun Wang
Yunfei Wang
Yunlong Wang
Yun-Ming Wang
Yun-Xing Wang
Yuqi Wang
Z. Wang
Zan Wang
Zefeng Wang
Zehua Wang
Zemin Wang
Zhang Wang
Zhanwei Wang
Zhao Wang
Zhaohui Wang
Zhao-Wen Wang
Zhen Wang
Zhen-Bo Wang
Zheng Wang
Zhenghe Wang
Zhengyi Wang
Zhenjia Wang
Zhenlin Wang
Zhen-Ying Wang
Zhigong Wang
Zhiguo Wang
Zhiheng Wang
Zhi-Hua Wang
Zhiquan Wang
Zhiqun Wang
Zhirui Wang
Zhishan Wang
Zhiyong Wang
Zhiyu Wang
Zhizhi Wang
Zhong Wang
Zhongfang Wang
Zhonghua Wang
Zhongqiu Wang
Zhongxiao Wang
Zhongyuan Wang
Zhou Wang
Zhoufei Wang
Zixin Wang
Zunyao Wang
Lawrence Wangh
Shabir H. Wani
Peter Waniek
Dierk Wanke
Erich Wanker
Goya Wannamethee
Barry Wanner
Kevin Wanner
Julia Wanschitz
Carolien Wansleeben
Sylvia Wanzala
Torsten Wappler
Eiji Warabi
Jonathan Warawa
Clea Warburton
David Warburton
Simon Warby
E. Warchalowska-Sliwa
Carol Ward

Daniel Ward
David Ward
Donald Ward
Emily Ward
Evan Ward
Julia Ward
L. Monique Ward
Leigh Ward
Marie-Louise Ward
Martha Ward
Michael Ward
Paul Ward
Peter Ward
Philip Ward
Robert Ward
Claire Wardak
Grant Wardell-Johnson
Klaas Wardenaar
Joanna Wardlaw
Tim Wardlaw
Kenneth Wardle
Chris Ware
James Ware
Jerry Ware
John Ware
Mark Ware
Norma Ware
John Wares
Barney Warf
Benjamin Warf
Jeffrey Waring
Molly Waring
Timothy Waring
Christina Warinner
Gulam Waris
Vivek Warkad
Sarah Warkentin
Laura Warman
Soren Warming
Saman Warnakulsuriya
Klaus Warnatz
Lisa Warnecke
Tobias Warnecke
Barbara Warner
Chris Warner
Erica Warner
Kenneth Warner
Natasha Warner
Sophie Warner
Timothy Warner
Andrea Warner-Czyz
Clemens Warnke
David Warnock
Tandy Warnow
Jan Warntjes
Alan Warren
Jeremy Warren
Katherine Warren
Meghan Warren
Mitchell Warren
Nicole Warren
Robert Warren
Sean Warren
Sharon Warren
Travis Warren
Zachary Warren
Kevin Warrian
Gary Warrick
Amy H. Warriner
Junie Warrington
Nicole Warrington
Erin Warshaw
Arjumand Warsy
Karolina Wartolowska
Anthony Waruru
James Waschek
Katherine Waselkov
Brian Washburn
Jason Washburn
Michael Washburn
Laraine Washer
Kazuo Washida
Naoki Washida
Robert Washington-Allen
Jason Wasiak
Agata Wasik
Jaroslaw Wasilewski
Job Wasonga
Mark Wass
Doug Wassenaar
L.I. Wassenaar
Trudy Wassenaar
Christoph Wasser
Clive Wasserfall
Max Wasserman
Molla Wassie
Anton Wasson
Katherine Wasson
Kate Wassum
Ayako Watabe
Kounosuke Watabe
Tadashi Watabe

Tetsuro Watabe
Atsushi Watanabe
Chiho Watanabe
Hideaki Watanabe
Hideto Watanabe
Hiroshi Watanabe
Junji Watanabe
Katsutoshi Watanabe
Masahiko Watanabe
Michiko Watanabe
Noriya Watanabe
Takayuki Watanabe
Tomohiro Watanabe
Toshiaki Watanabe
Toshihiro Watanabe
Yohei Watanabe
Yoichi Watanabe
Yosuke Watanabe
Yuichiro Watanabe
Yuuki Watanabe
Anna Waterhouse
Clare Waterman
Chris Waters
Michael Waters
Patrick Waters
Valerie Waters
W Waters
Patrick Waterson
John Waterton
Malcom Watford
D. Wathes
David Watkins
John Watkins
Laura Watkins
Richard Watkins
Dawn Watkins-Chow
James Watling
Jennifer Watling Neal
Gary Watmough
Shaun Watmough
Simon Watmough
Leiv Otto Watne
Stanley Watowich
Andrew Watson
Anna Watson
Bruce Watson
Charles Watson
Craig Watson
David Watson
Dennis Watson
Erica Watson
Malcolm Watson
Rachel Watson
Reginald Watson
Silvana Watson
Sue-Ann Watson
John Watt
Matthew Watt
Peter Watt
Richard Watt
Rory Watt
Torquil Watt
Alice Wattam
Elizabeth Wattenberg
Kenneth Watterson
Anneleen Watteyn
Pierre Wattiau
Gaetan Wattieaux
Rémi Wattier
Mike P. Wattjes
Jeffrey Watts
Jennifer Watts
Joel Watts
Stephanie Watts
Henrik Watz
Bianca Waud
Norman Waugh
Michaël Waumans
Nadeeka Wawegama
Adam Wax
Stephen G. Waxman
Astri Wayadande
Christian Waydhas
Mary Miu Yee Waye
Owen Waygood
Heidi Wayment
Jennifer Wayne
Craig Wayson
Ashley Wazana
Kerri Wazny
William Wcislo
Robert Weathers
Connie Weaver
Keith Weaver
Marcia Weaver
Mark Weaver
Nancy Weaver
Bryn Webb
Christian Webb
Christine Webb
Clinton Webb
Ian Webb

Jacqueline Webb
Ken Webb
Louise Webb
Nathan Webb
Roger Webb
Tonya Webb
Ann Webber
Gail Webber
Kelly Webber
Michael Webber
Andreas Weber
Bernd Weber
Bernhard Weber
Daniel Weber
Daniela Weber
Don Weber
Elin Weber
Frank Weber
Franz Weber
Georg F. Weber
Gerhard Weber
Ingmar Weber
Jennifer Weber
Jerzy Weber
Kerstin Weber
Lynn Weber
Mary Weber
Oliver Weber
Thomas Weber
Wolfgang Weber
Wolfgang Weber-Fahr
Sina Webering
Bonnie Webster
Christopher Webster
Nicholas Webster
A. Wechalekar
Mihir Dilip Wechalekar
Larry Wechsler
Stephen Wechsler
Frank Wechsung
Floyd Weckerly
Jan Weckstrom
Lucy Wedderburn
Lisa Wedding
Gero Wedemann
Aaron Weed
Brendan Weekes
Donald Weeks
Emma Weeks
Candice-Michelle Weems
Eranthie Weerapana
Scott Weese
Alfred Wegener
Ulrich Weger
Andrea Weghofer
Jerzy Wegiel
Joost Wegman
Michael Wegmann
Lars Wegner
Mathias Wegner
Michael Wegner
Grzegorz Wegrzyn
Martin Wegrzyn
Christian Wehenkel
Randy Wehling
Stefan Wehner
Alexander Wehr
John Wehr
Bernhard Wehrle-Haller
Jon Wehrlin
Ingo Wehrtmann
Chen Wei
Chih-Lin Wei
Chi-Ju Wei
Chu Wei
Chuanyu Wei
Cunxu Wei
Dan-Dan Wei
Daoyan Wei
Dongqing Wei
Dongtao Wei
Fang-Fei Wei
Fuwen Wei
Ge Wei
Gongyuan Wei
Guanghong Wei
Guanghui Wei
Haiyang Wei
Hong-Jiang Wei
Hongkui Wei
Hongping Wei
Hui Wei
James Cheng-Chung Wei
Jianhong Wei
Jianwei Wei
Jing Wei
Junni Wei
Kaifa Wei
Kang Wei
Lai Wei
Leyi Wei
Lijian Wei

Minxin Wei
Na Wei
Po-Li Wei
Qingqing Wei
Ri-Bao Wei
Sheng Wei
Shu-Chen Wei
Wei Wei
Wenbin Wei
Wenhui Wei
Wenqiang Wei
Xiaolin Wei
Xiaorong Wei
Xierong Wei
Yinan Wei
Yintao Wei
Yiyong Wei
Yongxiang Wei
Yu-Chung Wei
Yufeng Wei
Zhang Wei
Zhi Wei
Thomas Weide
Ornella Weideli
Thomas Weidemann
Michael Weiden
Christopher Weidenmaier
Karel Weidinger
David Weidman
Thomas Weig
Markus Weigand
Ronald Weigel
Steffen Weigend
Cora Weigert
Wm. Guy Weigold
Martin Weigt
Tom Weihmann
Cornelis Weijer
Mariska Weijerman
Dolf Weijers
Bert Weijters
Patrick Weik
Clifford Weil
E. Weil
Michael Weil
Danielle Weiler
Hope Weiler
Thomas Weimbs
Annelie Weinberg
Clarice Weinberg
David Weinberg
Ellen Weinberg
Seth Weinberg
Adam Weinberger
Irene C. Weinberger
Miles Weinberger
Agnes Weiner
Allon Weiner
Eran Weiner
Howard Weiner
Ina Weiner
Lev Weiner
Carolyn Weiniger
David Weinkove
Sheila Weinmann
Shantel Weinsheimer
Alejandro Weinstein
David Weinstein
Karen Weinstein
Robert Weinstein
Ada Weinstock
Michael Weinstock
Alva Weir
B.S. Weir
Gordon Weir
Matthew Weir
Luca Weis
Nina Weis
Stephen Weis
Andrea Weise
Cornelia Weise
Louis Weise
Scott Weisenberg
L. Weiser
Mark Weiser
Thomas Weiser
Heide Weishaar
Jochen Weishaupt
Kurt Weising
Daniela Weiskopf
Noah Weisleder
Kara Weisman
Alison Weiss
Bjoern Weiss
Brian Weiss
Dana Weiss
Daniel Weiss
David Weiss
Ehud Weiss
Johanna Weiss
Louis Weiss
Marie Weiss

Michael Weiss
Robert Weiss
Siegfried Weiss
Herbert Weissenböck
Wolfgang Weisser
Robert Weissert
Alison Weisskopf
Allan Weissman
Bernard Weissman
Daniel Weissman
Hanna Weiss-Schneeweiss
Nathan Weisz
Charles Weitz
Erica Weitz
Joshua Weitz
Nathaen Weitzel
Megan Weivoda
Alon Weizer
Lior Weizman
Christian Wejse
Eliud Wekesa
A. Welbourn
Pauline Welby
Carrie Welch
Dan Welch
Danny Welch
Ian Welch
Kenneth Welch
Berhe Weldearegawi
Christopher Weldon
Paul Weldon
Stephanie Weldon
Richard Weleber
Scott Welford
Aalim Weljie
Erik Welk
Martin Welk
Dennis Welker
Aron Weller
Donald Weller
Jennifer Weller
Joel Weller
Deneen Wellik
Bradley D. Welling
Paul Welling
Melanie Wellington
Barry Wellman
Charles Wellman
Athol Wells
Christine Wells
Claire Wells
Deborah Wells
Dominic Wells
E. Christian Wells
Hannah Wells
J. Wells
James Wells
Jerry Wells
Jim Wells
Kentwood Wells
Konstans Wells
Liz Wells
Scott Wells
Scott M. Wells
Sue Wells
Tim Wells
Tony Wells
Wendy Wells
Bryan Welm
Christoph Welsch
Ralf Welsch
Jane Welsh
Robert Welsh
Kathleen Welsh-Bohmer
Wade Welshons
Stephen E. Welty
Bohai Wen
Chun-Yi Wen
Colin Wen
Danyi Wen
Fengtong Wen
Frank Wen
Fuqiang Wen
Guoguang Wen
Haitao Wen
Han Wen
Hao Wen
Hongkai Wen
Hongwei Wen
Jie Wen
Jin-Kun Wen
Ke Wen
Li Wen
Quan Wen
Rong Wen
Shi Wu Wen
Shijun Wen
Sicheng Wen
Sijin Wen
Su-Ying Wen
Tzai-Hung Wen
Weijun Wen

Xi Wen
Xiang Wen
Xiaopeng Wen
Xiaotong Wen
Yingqiang Wen
Yiping Wen
Adam Wende
Wolfgang Wende
Chris Wendel
Hans-Guido Wendel
Ewa Wender-Ozegowska
Franz Wendler
Amanda Wendt
Michael Wendt
Mark Wener
Andrew Weng
Ching-Feng Weng
Chiu-Hsing Weng
Jing-Ru Weng
Juyang Weng
Kuo-Feng Weng
Shijun Weng
Xisheng Weng
Yaguang Weng
Zhen Weng
Ziqing Weng
Tobias Wengenmayer
Jonathan Wenger
Karl Wenger
Roland Wenger
Dorothea Weniger
Godehard Weniger
Xu Wenming
Karl Wennberg
Ann Wennerberg
Krister Wennerberg
E. Christina M. Wennerström
Gregor Wenning
Johan Wens
Knut-Arne Wensaas
Theodore Wensel
Annemarie Wensing
Felix Wensveen
Michaela Wenzel
Philip Wenzel
Sally Wenzel
Tom Wenzel
Uwe Wenzel
Wolfgang Wenzel
Frank Wenzhöfer
Hang-Yeon Weon
Lars Werdelin
Kennedy Were
Elizabeth Weretilnyk
Wopke Werf
Krystal Werfel
Janet Werker
Dirk Werling
Guilherme Werneck
Miriam Werneck
Ursula Werneke
Adrian Werner
Benjamin Werner
Earl Werner
Erika Werner
Felix-Martib Werner
Hauke Werner
Jeffery Werner
Kimberly Werner
Perla Werner
William Werner
Mathias Wernet
Kerstin Wernike
Sarah Werning
David Werring
David Wert
Alexander Werth
Victoria Werth
Margaret Werts
Philip Wertz
Eduardo Weruaga
Oliver Werz
Johannes Werzowa
David Wesche
Jørgen Wesche
Louise Weschler
Daniel Wescott
Wim Wesemael
Józefa Wesierska-Gadek
Cedric Wesley
Johnna Wesley
Robert Wesolowski
Patrick Wessa
Jennifer Wessel
Niels Wessel
Ralf Wessel
Stefan Wessel
Frank Wesselingh
Ignaz Wessler
Barbara Wessner
D. Wesson
Dan Wesson

Brady West
Bruce West
Charles West
J. B. West
Jevin West
John West
Robert West
Frances Westall
Lars Westberg
Jules Westbrook
Erik Westein
Hans-Jürgen Wester
Vincent Wester
Nelli Westercamp
Bjorge Westereng
Ryan Westergaard
Paul Westerhoff
J. Westerink
Ulrika Westerlind
Erica Westerman
Dirk Westermann
Stefan Westermann
Pål Westermark
Patrick Western
Theresia Westers
Klaas Westerterp
Margriet Westerterp-Plantenga
Margaret Westfall
Gerald Westheimer
Katarina Westling
Per-Olof Westlund
Karin Westlund High
Peter Westman
Cara Westmark
Kathryn Weston
Katrina Weston-Green
Kenneth Westover
Andreas Westphal
Johanna Westra
Ligia Westrich
Randal Westrick
Elizabeth Westrupp
Clara Westwell-Roper
Heather Westwood
James Westwood
Bradley Wetherbee
Mark Wetherell
James Wetmur
Jorn Wetterslev
Michael Wetz
Daniela Wetzel
Ronald Wetzel
Vera Wewer
Mark Wewers
Thomas Wex
Anthony Wexler
Steven Wexner
Tina Wey
Ingo Weyand
Nathan Weyand
Patrick Weydt
Jacqueline Weyer
Steven Weyers
Martin Weygandt
Karsten Weylandt
Laura Weyrich
Steve Whalan
Christopher Whalen
Diana J. Whalen
Alex Whaley
Paul Whaley
Heather Whalley
Lawrence Whalley
Dong Hee Whang
Edward Whang
Shuo Whang
Amy Wharton
Julian Wharton
Stephen Wharton
Adrian Whatmore
Rebecca Whear
Adam Wheatley
Lewis Wheaton
James Whedon
Sarah Wheelan
David Wheeler
Heather Wheeler
Michael Wheeler
Raymond Wheeler
Robert Wheeler
Sarah Wheeler
Tisha Wheeler
Travis Wheeler
Ward Wheeler
Thomas Whelan III
Patrick Whelley
Laura Wherry
Peter Whigham
Ian Whishaw
Desley Whisson
Keith Whitaker
Michael Whitaker

Jill Whitall
Aaron White
Angela White
Billy White
Corey White
Craig White
Crow White
Deborah White
Easton White
Elizabeth White
Emily White
Fletcher White
Gilbert White
Heath White
J. White
Jacob White
K. Andrew White
Kevin R. White
Kris White
Kristin White
Lauren White
Leigh White
Martin White
Michael White
Michelle White
Nancy Marie White
Neil White
Patricia White
Paul White
Peter White
Phillip White
Sarah White
Shane White
Stephanie White
Steve White
Tom White
Tonya White
Amy Whitehead
Clare Whitehead
John Whitehead
Ross Whitehead
Shawn Whitehead
Timothy Whitehead
Abigail Whitehouse
Jamie Whitehouse
Christopher Whitehurst
Michael Whitehurst
William Whiteley
Andrew Whiten
Travis White-Schwoch
Lauren Whiteside
Katrine Whiteson
Thomas Whitfield
Thomas Whitford
Veronica Whitford
Martin Whitham
Steven Whitham
Scott Whiting
Matthew Whitley
Janis Whitlock
Jonathan Whitlock
Donna Whitlon
William Whitman
Tristan Whitmarsh
Ken Whitney
Simon Whitney
Susanna Whitney
Tim Whitsett
Danielle Whittaker
Robert Whittaker
Sarah Whittle
Alexis Whitton
Chris Whitton
J. Lindsay Whitton
John Whitton
Lisa Whop
Steve Whyard
Stephen Whybrow
Elisabeth Whyte
Marlene Wiart
Gudrun Wibbelt
Sebastian Wiberg
Constantinos Kurt Wibmer
Ove Wiborg
Michael Wibral
Mark Wicclair
Nicole Wicha
Thomas Wichard
Szymon Wichary
Marieke Wichers
Anette Wichman
Julian Wichmann
Rolf Wichmann
Søren Wichmann
Oliver Wicht
Macdonald Wick
Susann Wicke
Robert Wickesberg
Norman Wickett
Abeni Wickham
Samuel Wickline
Larysa Wickman

Paul Wicks
Eric Wickstrom
J. Widder
Julian D. Widder
Jos Widdershoven
Jonathan Widdicombe
Robert Widdop
Anders Widell
Ralf Widenhorn
Michael Widlansky
Giovanni Widmer
Matthew Wiebe
Sandra Wiebe
Tonya Wiechel
Wolf Wiedemeyer
Susanne Wiedmer
Ryan Wiegand
Thorsten Wiegand
Armin Wiegering
J. Simon Wiegert
Geert Wiegertjes
Brian Wiegmann
Lutz Wiehlmann
Barbara Wieland
Ilse Wieland
Jannelien Wieland
Kai Wieland
Martijn Wieling
Mark Wielpütz
Philipp Wiemann
David Wiemer
Elizabeth Wiemers
Martin Wiemers
Lyle Wiemerslage
Frank Wien
John Wiencke
Martin Wiener
Klaus Wienhard
Brian Wienhold
Stefanie Wienkoop
Matthew Wiens
Nicolette Wierdsma
Diane Wiernasz
John Wiernikowski
Corinde Wiers
Reinout Wiers
Rodney Wiersma
Adam Wierzbicki
Andrzej Wierzbicki
Piotr Wierzbicki
Bob Wiese
Claudia Wiese
Holger Wiese
L. Wiese
Simon Wiesel
Dennis Wiesenborn
David Wiesenthal
Martin Wiesmann
Glen Wiesner
Ulrike Wiesspeiner
Roland Wiest
Ellen Wiewel
Thimo Wiewelhove
Maciej Wiewióra
Brian Wigdahl
George Wiggans
Øystein Wiggen
Giulia Wiggers
Geraint Wiggins
Jim Wight
Johanna Wigman
Helge Wiig
Emma Wiik
Arief Wijaya
Sobanawartiny Wijeakumar
Buddhika Wijerathne
Berry Wijers
Danushka Wijesundara
Alienke Wijmenga-Monsuur
Ellen Wijsman
Anne Wijtzes
Jan Wikgren
John Wikswo
Aaron Wilber
David Wilbur
Damien Wilburn
Matthew Wilce
Andrea Wilcks
C. Wilcox
Kevin Wilcox
Laurie Wilcox
Walter Wilczynski
Christian Wild
David Wild
Klemens Wild
Margaret Wild
Andrew Wilde
Parke Wilde
Willem Wildering
Einar Wilder-Smith
Dane Wildner
Gerhild Wildner

Christine Wildsoet
Craig Wilen
Louise Wiles
Siouxsie Wiles
James Wiley
Joshua Wiley
Kerrie Wiley
Steven Wiley
Traci Wilgus
Brian Wilhelm
Clare Wilhelm
Dagmar Wilhelm
Dirk Wilhelm
Helmut Wilhelm
Oliver Wilhelm
Steven Wilhelm
D. Ray Wilhite
Mateusz Wilinski
Anna Wilk
Mieszko Wilk
Piotr Wilk
Claus Wilke
Jan Wilke
Jason Wilken
Heinrike Wilkens
Elissa Wilker
Curtis Wilkerson
Lesley Wilkes
Martin Wilkes
Rima Wilkes
Iain Wilkie
John Wilkin
Hendrik Wilking
John Wilkins
Michael Wilkins
Anna Wilkinson
Brian Wilkinson
Christopher Wilkinson
David Wilkinson
Ian Wilkinson
Lynne Wilkinson
Mark Wilkinson
Patrick Wilkinson
Royce Wilkinson
Ivy Wilkinson-Ryan
Manuel Will
Matthew Will
Andy Willaert
Kurt Willamson
Victoria Willard
Endre Willassen
Dieter Willbold
Rebecca J. Willcocks
Carrie Willcox
Donald Willcox
Holger Wille
Florian Willecke
Stefanie Willekens
Anne Willems
Annemarie Willems
Lucas Willems
Rob Willems
Ina Willemsen
Barry Willer
Robb Willer
Henning Willers
Eske Willerslev
Cali Willet
Christopher Willett
Robert Willey
Basem M. William
Watson William
William N. William Jr.
Alan Williams
Allison Williams
Andrew Williams
Anna Williams
Anne Williams
Arthur Williams
B. A. Williams
Brett Williams
Candace Williams
Carey Williams
David Williams
Desmond Williams
Diana Williams
Dionna Williams
Dominic Williams
Elizabeth Williams
Frances Williams
Gabrielle Williams
Gary Williams
Hannah Williams
Helen Williams
Ivor Williams
Jake Williams
James Williams
Joah Williams
Joanne Williams
Joshua Williams
June Williams
Kelly Williams

Lisa Williams
Louise Williams
Lynda Williams
Mark Williams
Monique Williams
Paige Williams
Phoebe Williams
Rebecca Williams
Redford Williams
Richard Williams
S. Williams
Scott Williams
Simon Williams
Sophie Williams
Steven Williams
Thomas Williams
Tom Williams
Trevor Williams
Veronika Williams
Wendy Williams
Martin M. Williams II
Craig Williamson
Diane Williamson
Esther Williamson
Grant Williamson
Nancy Williamson
Peter Williamson
C.K. Willie
Nienke Willigenburg
Benjamin Willing
Marian Willinger
Tim Willinger
Arve Willingham
Field Willingham
John Willinsky
Craig Willis
Mary S. Willis
Megan Willis
Scooter Willis
Marian Willner
Paul Willner
Thomas Willnow
Laetitia Willocquet
Darryn Willoughby
Nik Willoughby
Thomas Wills
Zachary Wills
John Willson
Sascha Willuweit
Bettina Wilm
Ivan Wilmot
Annick Wilmotte
Kate Wilmut
Michael Wilschanski
A. Wilson
Adel Wilson
Alan Wilson
Anne Wilson
Carole Wilson
Charles Wilson
Christopher Wilson
Darrell Wilson
David Wilson
Dawn Wilson
Derek Wilson
Donald Wilson
Eric Wilson
Fernando Wilson
Gerald Wilson
Greg Wilson
Heather Wilson
James Wilson
Jefferson Wilson
Jennifer Wilson
Joyce Wilson
Kenady Wilson
Lewis Wilson
M. J. Wilson
Mark Wilson
Matthew Wilson
Melinda Wilson
Patrick Wilson
Paul Wilson
R. Wilson
Rick Wilson
Shaun Wilson
Stephen Wilson
Stuart Wilson
Sylia Wilson
Thad Wilson
Thomas Wilson
Christine Wilson-Mendenhall
Emily Wilson-Smith
Andreas Wilting
Jörg Wilting
Steve Wilton
Bodo Wilts
Jeffrey Wilusz
Kandatege Wimalasena
William Wimley
Ernst Wimmer
Isabella Wimmer

Valentin Wimmer
Klaus Wimmers
Wipas Wimonsate
Gina M. Wimp
Aung Win
Elizabeth Wina
Cecilia Winata
Jan-Olof Winberg
Julia Winchester
Flavia Winck
Thomas Winckler
Stephan Windecker
Virginia Winder
Daniel Windhorst
Hannah Windley
Frank Windmeijer
Paul Windschitl
Amanda Windsor
John Windsor
Michael Wine
Amy Winecoff
Tony Winefield
Katarzyna Winek
Andrew Winer
Daniel Winetsky
Rena Wing
Quinton Winger
Jason Winget
John Wingfield
Aliza Wingo
Stacey Winham
Yvonne Winhofer
John Winkelman
Holger Winkels
Kevin Winker
Anderson Winkler
Cheryl Winkler
Christian Winkler
Robert Winkler
Jeffrey Winkles
Kirsty Winkley
Michael Winklhofer
Bryan Winn
Mary Winn
Rainer Winnenburg
Andrew Winokur
Peter Winskill
Dean Winslow
Craig Winstanlay
John Winston
Anne-Luise Winter
Evelyn Winter
Felix Winter
Lilli Winter
Lukas Winter
Stephan Winter
Stuart Winter
Patrick Wintrode
Wipawee Winuthayanon
Renaud Winzenrieth
Ursula Winzer-Serhan
Anuwat Wiratsudakul
Karin Wirdefeldt
Isaac Wirgin
Christian Wirkner
Herman Wirshing
Jonathan. Wirsich
Norman Wirsik
Aaron Wirsing
Carlheinz Wirsing Von Koenig
Dagmar Wirth
Francesca Wirth
Janine Wirth
Michael Wirth
Michelle Wirthensohn
Linda Wirthwein
David Wirtshafter
Andrea Wirtz
Markus Wirz
M. Wischnewski
Jonathan Wisco
David Wise
Paul Wise
Steven Wise
Toby Wise
Jennifer Wisecaver
Richard Wiseman
Roger Wiseman
Virginia Wiseman
Gerald Wisenberg
Kari Wisinski
Sabine Wislet
Marie Wislez
Michael Wisniewski
Hilmar Wisplinghoff
Bernd Wissinger
Birte Wistinghausen
Henryk Witas
Carol Witczak
Henryk Witek
Maria Witek
Kim With

Joan Wither
Phil Withers
Jeffrey Withey
John Withey
Steven Witkin
Veronique Witko-Sarsat
Benoit Witkowski
Janusz Witowski
Jens Witsch
Maarten Witsenburg
Benjamin Witt
Christopher Witt
Karsten Witt
Erika Wittchen
Daniel R. Witte
Klaudia Witte
Matthias Witte
Russell Witte
Wolfgang Witte
Christian Wittekind
Jurrian Witteman
George Wittemyer
Luke Wittenburg
Paula Witt-Enderby
Gary Wittert
Briana Witteveen
Ilka Wittig
Alfred Wittinghofer
Helmut Wittkowski
Christiane Wittmann
Isabel Witzel
Maren Witzig
Viroj Wiwanitkit
Mary Wlodek
Gary Wnek
Wendy Wobeser
Andreas Wodarz
Eric Woehler
Christoph Woelfl
Gert Woerheide
Christoph Woernle
Malte Woestmann
Guinevere Wogan
Ellen Wohl
Thorsten Wohland
Teddy John Wohlbold
Peter Wohlfahrt
Nicholas Wohlgemuth
Roland Wohlgemuth
Stephanie Wohlgemuth
Peter Wohlsein
Daniel Wohlwend
Markus Wöhr
Jochen Wöhrle
Wendy Woith
Jerzy Wojciechowski
Martin Wojciechowski
Slawomir Wójcik
Katarzyna Wojczulanis-Jakubas
Christina Wojewoda
Marcin Wojewodzic
Talya Wolak
Eric Wolanski
Susane Wolbank
Gertjan Wolbink
Gregor Wolbring
Tobias Wolbring
Jeff Wolchok
Daniel Wolcott
C. Wolf
Dennis Wolf
Dieter Wolf
Jennifer Wolf
Jennyfer Wolf
Jürgen Wolf
Katarina Wolf
Leslie Wolf
Marina Wolf
Myles Wolf
Nicole Wolf
Ronald Wolf
Tiffany Wolf
Timo Wolf
Bernd Wolfarth
Adam Wolfberg
Alan Wolfe
Benjamin Wolfe
Charles Wolfe
Heather Wolfe
Joanna Wolfe
John Wolfe
Kenneth Wolfe
Thomas Wolfers
Daniel Wolff
Daynna Wolff
Ewan Wolff
Petra Wolffs
James Wolffsohn
Christopher Wolfgang
Astrid Wolf-Magele
Mariana Wolfner
Karen Wolford

Christina Wolfson
Willem Wolkers
Bencie Woll
Gena Wollenberg
G. Wollensak
Tim Wollesen
Lonnie Wollmuth
Andreas Wollstein
Gadi Wollstein
Marc Wolman
Bettina Wölnerhanssen
Katarzyna Wolny-Koladka
Herman Wolosker
Frank Wolschendorf
Steven Woltering
Maike Wolters
Paul Wolters
Knud Woltjen
Ines Wolz
Kyu Chang Won
Kyung Won
Misun Won
Young-Joo Won
Betsy Wonderly
Dessie Salilew Wondim
Adrian Wong
Albert Wong
Angel Wong
Arnold Wong
Boon-Seng Wong
Carlos Wong
Charles Wong
Chee Wai Wong
Chi-Huey Wong
Chongkim Wong
Chris Wong
Conroy Wong
Cynthia Wong
Elaine Wong
Evan Wong
Flora Wong
Francis Wong
George Wong
Grace Wong
Hoi Leong Xavier Wong
Hoo Keat Wong
Irene O.L. Wong
Jack Wong
Jason Wong
Jeremy Wong
Jessica Wong
Joshua Wong
Kah Keng Wong
Kam-Bo Wong
Karen Wong
Kelvin Wong
Ken Koon Wong
Kingsley Wong
Kwong Fai Wong
Kwoon Wong
Lee-Jun Wong
Li Ping Wong
Limy Wong
Madeline Wong
Man-Sau Wong
Mitchell Wong
Oscar Wong
Pooi Wong
Raymond C. B. Wong
Samuel Wong
Soon Boon Justin Wong
Tak Pan Wong
Teck Yee Wong
Ten Tsao Wong
Ting Hway Wong
Tzu-Tsung Wong
Vickie Wong
Wei Wen Wong
Wendy S.W. Wong
William Wong
William W. L. Wong
Wilson Wong
Won Fen Wong
Yetta Wong
Surasakdi Wongratanacheewin
Chang-Hoon Woo
Connie Woo
Jean Woo
Jessica Woo
John Woo
Jongchan Woo
Shih Lung Woo
Tom Woo
Youngkeun Woo
Aaron Wood
Andy R. Wood
Brennon Wood
Brian Wood
Chelsea Wood
Clare Wood
Eric Wood
Fiona Wood

Ian Wood
James Wood
Janet Wood
John Wood
Kevin Wood
Kris Wood
Lara Wood
Lekki Wood
Matthew Wood
Naomi Wood
Petra Wood
Rachel Wood
Scott Wood
Susan Wood
Susanna Wood
Tana Wood
Terri Wood
William Woodall
Craig Woodard
Geoffrey Woodard
S. Woodard
Ryan James Wood-Bradley
Michelle Woodbury
Ben Woodcock
Skye Woodcock
Michael Woodford
James Woodgett
James Woodhall
Zoe Woodhead
Francis Woodhouse
Jason Woodhouse
Jim Woodhouse
Mark Woodin
Owen Woodman
Lorna Woodrow
Andrew Woods
Heather Woods
Jason Woods
Rachael Woods
Rebecca Woods
Sarah Woodson
Alistair Woodward
Maria Woodward
Craig Woodworth
Orison Woolcott
Sarah E. Woolf-King
Kevin Woollard
Thomas Woolley
Kyle Woosnam
Joshua Wooten
Daniel Wooton
Franz Worek
Tilla Worgall
Lorraine Work
Thierry Work
Biruh Workeneh
Joanna Workman
Abebaw Worku
Micah Worley
Dag Wormanns
Floyd Wormley Jr.
Michael Worobey
Viktoria Woronik
Jonathan Worrall
Austen Worth
Randall Worth
Darrell Worthy
Bruce Worton
Leah Worton
Han Wosten
Bernd Wöstmann
Joyce Woudenberg
Jan Wouters
Maartje Wouters
Merridee Wouters
Olivier Wouters
Paul Wouters
Brian Wowk
Greta Wozniak
Karen Wozniak
Krystyna Wozniak
Michal Wozniak
Grzegorz Wozniakowski
Susan Wray
Anna Wredenberg
Alexander Wree
Johanna Wren
Jonathan Wren
Ian Wrench
Carsten Wrenger
Amber Wright
Casey Wright
Chris Wright
Craig Wright
Davene Wright
David Wright
Emma Wright
Gavin Wright
Genevieve Wright
J. Timothy Wright
Jeremy Wright
Jessica Wright

Justin Wright
Kenneth Wright
Matthew Wright
Melecia Wright
Mia Wright
Michael Wright
Pamela Wright
Patricia Wright
Rachel Wright
Robin Wright
Thomas Wright
Timothy J. Wright
William Wright
Kenneth Wright Jr.
Julie Wright Nunes
Katarzyna Wrobel
Marta Wróblewska
Lydia Wroblewski
Nina Wronkowitz
Kazimierz Wrzeszczynski
Ai-Ping Wu
Alan Wu
Anhua Wu
Baojun Wu
Baolin Wu
Bei Wu
Ben-Hong Wu
Bin Wu
Bing Wu
Bingfang Wu
Bingyi Wu
Boming Wu
Boyang Wu
Can Wu
Changqing Wu
Changshan Wu
Chao Wu
Chaodong Wu
Chen Wu
Chen-Chi Wu
Chenggang Wu
Cheng-Kun Wu
Chengtie Wu
Chen-Hsuan Wu
Chen-Yi Wu
Chia-Chao Wu
Chia-Ching Wu
Chia-Lin Wu
Christina Wu
Chuanfeng Wu
Chun-Ying Wu
Congming Wu
David Wu
De-Cheng Wu
Ding-Kwo Wu
Dong Chuan Wu
Dong-Dong Wu
Erxi Wu
Fan Wu
Fang-Tzy Wu
Fred Wu
Fred M. Wu
Gang Wu
Gao-Lin Wu
Gary Wu
Ge Wu
Gregory Wu
Guan-Chung Wu
Guang Wu
Guangxi Wu
Guanming Wu
Guodong Wu
Guojin Wu
Guojun Wu
Guoqiu Wu
Haiyan Wu
Haiyong Wu
Hanwen Wu
Hao Wu
Hau-tieng Wu
Hong Wu
Hongxian Wu
Hongzhuan Wu
Hsiang-En Wu
Hsiu Wu
Hua Wu
Huapeng Wu
Hui Wu
Hung-Tsung Wu
J.J. Wu
Jasmine Wu
Jaw-Ching Wu
Jeng-Yih Wu
Jen-Leih Wu
Jiahe Wu
Jianhui Wu
Jianmin Wu
Jianming Wu
Jianqiang Wu
Jianshuang Wu
Jianyu Wu
Jiawen Wu

Jing Wu
Jinhu Wu
Jinjun Wu
Jinshui Wu
Jiong Wu
Jiunn-Tzong Wu
John Wu
Joy Wu
Jun Wu
Junfang Wu
Juxun Wu
Kai Wu
Keliu Wu
Kendra Wu
Keng-Liang Wu
Kevin Wu
Kongming Wu
Kuenyuh Wu
Kuo-Sheng Wu
Lang Wu
Lei Wu
Li Wu
Li-Chen Wu
Li-Ling Wu
Liu Wu
Liusan Wu
Liyong Wu
Long Fei Wu
Mai-Szu Wu
Mian-Hua Wu
Min Wu
Ming Wu
Mingfu Wu
Minghua Wu
Ming-Ju Wu
Nan Wu
Pei-Chen Wu
Pei-Hsun Wu
Peiwen Wu
Ping Wu
Pingsheng Wu
Qiang-Sheng Wu
Qiaofeng Wu
Qi-Jun Wu
Qingbo Wu
Qingfa Wu
Qingping Wu
Renyi Wu
Rong-Tsun Wu
Ruifang Wu
Sean Wu
Semon Wu
Shan Wu
Shandong Wu
Shangong Wu
Sheng Wu
Sherry Wu
Shinn-Chih Wu
Shiyong Wu
Shu Wu
Shuang Wu
Shuangchan Wu
Shuicai Wu
Shyi-Kuen Wu
Siqi Wu
Song Wu
Szu-Yuan Wu
Ta Yeong Wu
Tao Wu
Te Wu
Teresa Wu
Tianfu Wu
Ting-Fang Wu
Tingfeng Wu
Tsung-Ju Wu
Tsu-Yin Wu
Victor Chien-Chia Wu
Wanqing Wu
Wei Wu
Weihua Wu
Wei-Sheng Wu
Wen-Bin Wu
Wen-Chau Wu
Wen-Jeng Wu
Wenzhuo Wu
Xiang Wu
Xiaofeng Wu
Xiaogang Wu
Xiaohang Wu
Xiaohong Wu
Xiaohua Wu
Xiao-Lin Wu
Xiaomin Wu
Xinkai Wu
Xinwei Wu
Xiong-Zhi Wu
Xiujuan Wu
Xiuyun Wu
Xueling Wu
Xufeng Wu
Xugan Wu
Y. Wu

Yan Wu
Yanyuan Wu
Yen-Wen Wu
Yih-Ru Wu
Yin Wu
Ying Wu
Yong Wu
Yong-Ping Wu
Yongqi Wu
Yongxia Wu
Youyou Wu
Yuanxin Wu
Yue Wu
Yun-Hsuan Wu
Yu-Wei Wu
Yuxia Wu
Zeyan Wu
Zhaohui Wu
Zhenfang Wu
Zheng Wu
Zhengrong Wu
Zhengwang Wu
Zhenlong Wu
Zhichao Wu
Zhihao Wu
Zhijian Wu
Zhiqiang Wu
Zhong-Dao Wu
Jasper Wubs
Patrick Wuchter
Max Wuehr
Ullrich Wuellner
Jens Wuerfel
Barbara Wueringer
Wahyu Wulaningsih
Brian Wulff
Heike Wulff
Elsio Wunder Jr.
Richard Wunderink
F. Thomas Wunderlich
Zeba Wunderlich
Hanno Würbel
Kenneth Wurdack
Hada Wuriyanghan
Florian Wurm
Yannick Wurm
Olivier Wurtz
Rolf Würtz
Danielle Wurzel
Maria Wurzinger
Judy Wu-Smart
Hilde Wustenberghs
Daniel Wüstner
Vanaporn Wuthiekanun
Marcel Wuthrich
Amber Wutich
Wim Wuyts
Tana Wuyun
Gail Wyatt
Michael Wyatt
Richard Wyatt
Robert Wyatt
Sean Wyatt
Maciej Wybraniec
Michael Wybrow
C. Andrew Wyenandt
Peter Wyer
Malgorzata Wygrecka
Kristine Wylie
Stephen Wylie
Elvin Wyly
Brian Wymbs
Niels Wynant
Laure Wynants
Jeanette Wyneken
Grace Wyngaard
Clive Wynne
Jut Wynne
Lonce Wyse
Barbara Wyslouzil
Marcin Wysoczynski
Grzegorz Wystrychowski
Stavra Xanthakos
Petros Xanthopoulos
Raquel Xavier
Sandhya Xavier
Bobbi Xayarath
Michalis Xenos
Christian Xerri
Bowei Xi
Chuanwu Xi
Jie Xi
Jinxiang Xi
Lei Xi
Liqiang Xi
Peng Xi
Xiaodong Xi
Yaguang Xi
Yi-Bo Xi
Yutao Xi
Chao-Ming Xia
Haiyong Xia

Jian-Chuan Xia
Jianjun Xia
Junfeng Xia
Junhong Xia
Kuaifei Xia
Lijun Xia
Lizhong Xia
Ming Xia
Mingrui Xia
Qingyou Xia
Rui Xia
Shibin Xia
Siyu Xia
Tian Xia
Ting Xia
Xianchun Xia
Xiaofeng Xia
Xiao-Jian Xia
Xiao-Ping Xia
Xin Xia
Yong Xia
Yongqiu Xia
Yongxiang Xia
Yuchen Xia
Yun Xia
Zhengjun Xia
Zhengyuan Xia
Dehui Xiang
Fengning Xiang
Heng Xiang
Huiyun Xiang
Jianjun Xiang
Qiuyun Xiang
Sen Xiang
Wei Xiang
Xu Xiang
Yang Xiang
Yu Xiang
Yun Xiang
Yu-Tao Xiang
Bin Xiao
Binggang Xiao
Fei Xiao
Guanghua Xiao
Guanping Xiao
Guoying Xiao
Guozhi Xiao
Han Xiao
Hang Xiao
Hua Xiao
Hui Xiao
Jiajie Xiao
Jian Xiao
Jianru Xiao
Jie Xiao
Jin Xiao
Jing Xiao
Jingfa Xiao
Junfeng Xiao
Junjie Xiao
Lanbo Xiao
Lehui Xiao
Li Xiao
Mingming Xiao
Qian Xiao
Shijun Xiao
Shui-Yuan Xiao
Shunyuan Xiao
Shuo Xiao
Shuqi Xiao
Sumei Xiao
Tian Xiao
Weidong Xiao
Xiangshu Xiao
Xuan Xiao
Yan Xiao
Yandong Xiao
Yanni Xiao
Yian Xiao
Yihong Xiao
Yong Xiao
Yu-Xi Xiao
Zhong-Dang Xiao
Feng Xiaobo
Jiing Xiaoqing
Bing Xie
Bowen Xie
Conghua Xie
Dan Xie
Guosen Xie
Guosheng Xie
Hongbo Xie
Huaqing Xie
Huirong Xie
Huisheng Xie
Jianfei Xie
Jianping Xie
Jingwei Xie
Junhui Xie
Keqin Xie
Lu Xie
Minqiang Xie

Peng Xie
Ping Xie
Qi Xie
Qingmei Xie
Shao-Dong Xie
Shao-Hua Xie
Sheng Xie
Shuguang Xie
Shulian Xie
Tao Xie
Wei-Dong Xie
Wei-Ping Xie
Wen-Jie Xie
Xiangqun Xie
Xian-Jin Xie
Xiaohua Xie
Xiaoming Xie
Xueqian Xie
Xue-Qian Xie
Yang Xie
Yanqi Xie
Yichun Xie
Yihong Xie
Ying Xie
Yuanyuan Xie
Yuxiang Xie
Yuying Xie
Zheng Xie
Zhenping Xie
Zhi-Xun Xie
Yin Xiling
Maria Xilouri
Xu Ximing
Gao Xin
Gongming Xin
Haiping Xin
Ruolei Xin
Tao Xin
Zhong-Cheng Xin
Bengang Xing
Changhong Xing
Chao Xing
Chuanhua Xing
Dongqi Xing
Guanglin Xing
Haoyang Xing
Jingjing Xing
Keyi Xing
Lianping Xing
Qi Xing
Yongzhong Xing
Zhou Xing
Bo Xiong
Chengjie Xiong
Fei Xiong
Glen Xiong
Guangyan Xiong
Guosheng Xiong
Hongyan Xiong
Huangui Xiong
Huihua Xiong
Jianyin Xiong
Jun Xiong
Lize Xiong
May P. Xiong
Peng Xiong
Shigang Xiong
Shunbin Xiong
Shuping Xiong
Tou Cheu Xiong
Wei Xiong
Weijun Xiong
Xiaoxing Xiong
Xinyu Xiong
Yan Xiong
Ye Xiong
Yulan Xiong
Zhilong Xiu
Bin Xu
Bing Xu
Binghe Xu
Bingliang Xu
Binjie Xu
Changyan Xu
Chaoqun Xu
Chengfu Xu
Chengqi Xu
Chenwu Xu
Chonghai Xu
Da Xu
Dan Xu
Daochun Xu
Dawei Xu
Dazhong Xu
Degang Xu
Dingli Xu
Dongrong Xu
Dongsheng Xu
Fei Xu
Feng Xu
Fengfeng Xu
Fengguo Xu

Guan Xu
Guang-Yin Xu
Guangyu Xu
Guilian Xu
Guobin Xu
Hai-Chuan Xu
Haigen Xu
Haineng Xu
Hao Xu
Henglong Xu
Hengyi Xu
Heping Xu
Hongbin Xu
Hongxia Xu
Hua-Guo Xu
Huajun Xu
Huanbin Xu
Huimin Xu
Jia Xu
Jian Xu
Jianbo Xu
Jianchu Xu
Jianfeng Xu
Jianguo Xu
Jian-Hong Xu
Jianjiang Xu
Jianping Xu
Jian-Rong Xu
Jianzhong Xu
Jia-Ping Xu
Jiliang Xu
Jin Xu
Jinchong Xu
Jing Xu
Jingliang Xu
Jingyue Xu
Jiru Xu
Juan Xu
Jun Xu
Junqian Xu
Junzeng Xu
Ke Xu
Kehui Xu
Lei Xu
Leyan Xu
Li Xu
Lin Xu
Ling Xu
Liping Xu
Meifeng Xu
Min Xu
Minggang Xu
Nianjun Xu
Nong Xu
Peng Xu
Pengfei Xu
Ping Xu
Pingyi Xu
Qi Xu
Qiang Xu
Qianli Xu
Qingwen Xu
Quanqing Xu
Ren Xu
Rifu Xu
Rong Xu
Rugen Xu
Ruliang Xu
Sen Xu
Shengfeng Xu
Shiqing Xu
Shi-Wen Xu
Shufang Xu
Shun Xu
Shunbin Xu
Shutong Xu
Sihua Xu
Tianjun Xu
Ting Xu
Ting-Yan Xu
W. Xu
Wei Xu
Weifeng Xu
Weigang Xu
Wenbo Xu
Wenfang Xu
Wenhua Xu
Wenyuan Xu
Wenzhong Xu
Wu Xu
Xiangyang Xu
Xiaofeng Xu
Xiaohui Xu
Xiao-Hui Xu
Xiao-Ke Xu
Xiaomeng Xu
Xiaoyong Xu
Xin Xu
Xing Xu
Xingliang Xu
Xingshun Xu
Xin-Jian Xu

Xiufeng Xu
Xiulong Xu
Xuehu Xu
Xuehua Xu
Xuenong Xu
Xue-Wei Xu
Yan Xu
Yang Xu
Yanming Xu
Yaozhan Xu
Yi Xu
Yichi Xu
Ying-Chun Xu
Yong Xu
Youhua Xu
Yue Xu
Yuming Xu
Yun Xu
Yunan Xu
Yungang Xu
Z. Xu
Zhangrong Xu
Zhao-Shi Xu
Zheng Xu
Zhi Xu
Zhice Xu
Zhiqiang Xu
Zhonglin Xu
Qi Xuan
Tran Dang Xuan
Liu Xuanming
Bin Xue
Chaoyang Xue
Chunyi Xue
Dawei Xue
Huijie Xue
Jian Xue
Jinzhuang Xue
Junli Xue
Liang Xue
Lixiang Xue
Niantao Xue
Pengfei Xue
Rui-De Xue
Ruyi Xue
Sheng-Guo Xue
Xian Xue
Xiang Xue
Xiaofeng Xue
Yiqun Xue
Yu Xue
Zhong Xue
Xia Xueshan
Dimitris Xygalatas
Evaghelos Xynos
Evangelos Xynos
Beery Yaakov
Rami Yaari
Daisuke Yabe
Alexey Yablokov
Rabi Yacoub
Talene Yacoubian
Ajay Kumar Yadav
Chandra Yadav
Gitanjali Yadav
Jagjit Yadav
Naresh Yadav
Neelu Yadav
Prashant Yadav
Rajesh Yadav
Ravi Yadav
Roopali Yadav
Sanjay Yadav
Savita Yadav
Sudesh Yadav
Sumit Yadav
Sunita Yadav
Vishal Yadav
Pramod Yadava
Sagar Yadavali
Tejabhiram Yadavalli
Azadeh Yadollahi
Rona Yaeger
Maziar Yaesoubi
Faysal Yafi
Jill Yager
Shadi Yaghi
Mohsen Yaghoubi
Tetsuya Yagi
Yukako Yagi
Julia Yaglom
Eray Yagmur
Kazuhide Yahata
Timothy Yahr
Mahmoud Yaish
Mamiko Yajima
Michail Yakimov
Sergiy Yakovenko
Binnaz Yalcin
Ozlem Yalcin
Gary Hin-Fai Yam
Akira Yamada

Atsushi Yamada
Hanano Yamada
Hisakata Yamada
Kaori Yamada
Kaoru Yamada
Kenji Yamada
Kiyofumi Yamada
Mamoru Yamada
Mitsuhiro Yamada
Sohsuke Yamada
T. Yamada
Yoshiji Yamada
Yoshitsugu Yamada
Yosuke Yamada
Hiroshi Yamagami
Akira Yamaguchi
Atsushi Yamaguchi
Hiroyuki Yamaguchi
Takeshi Yamaguchi
Tsuyoshi Yamaguchi
Yoshifumi Yamaguchi
Hirosuke Yamaji
Ryoichi Yamaji
Takeo Yamakawa
Hidenaga Yamamori
Elichiro Yamamoto
Kazuhiko Yamamoto
Kazuhiro Yamamoto
Kazuki Yamamoto
Ken Yamamoto
Kohji Yamamoto
Koichi Yamamoto
Masakazu Yamamoto
Masato Yamamoto
Masayuki Yamamoto
Naohide Yamamoto
Nobuhiko Yamamoto
Norio Yamamoto
Ryohei Yamamoto
Satoshi Yamamoto
Shinya Yamamoto
Shuichiro Yamamoto
Tae Yamamoto
Tsunehisa Yamamoto
Yasuhiko Yamamoto
Yoshiharu Yamamoto
Yuji Yamamoto
Jesus Yamamoto-Furusho
Aya Yamamura
Soichiro Yamamura
Kazushige Yamana
Teresa Yamana
Hiroki Yamanaka
Hisashi Yamanaka
Keiichi Yamanaka
Naoki Yamanaka
Toshiro Yamanaka
Hirotomo Yamanashi
Daisuke Yamane
Seiichi Yamano
Keitaro Yamanouchi
Kunihiro Yamaoka
Hideyuki Yamashiro
Kenji Yamashiro
Atsushi Yamashita
Haruyuki Yamashita
Renata Yamashita
Takehiro Yamashita
Tomohisa Yamashita
Tomoya Yamashita
Toru Yamashita
Yoshihisa Yamashita
Koji Yamatsu
Tomoki Yamatsuji
Akira Yamauchi
Hiroshi Yamauchi
Osamu Yamauchi
Yusuke Yamauchi
Hiroki Yamaue
Hideyuki Yamawaki
Shigeto Yamawaki
Hidekatsu Yamazaki
Kazuto Yamazaki
Masahito Yamazaki
Soh Yamazaki
Tomio Yamazaki
Sophie Yammine
Ebenezer Yamoah
Wataru Yamori
Yukio Yamori
Lev Yampolsky
A. Yamunadevi
Aixin Yan
Bin Yan
Dandan Yan
Elsie Yan
Gang Yan
Guijun Yan
Guoliang Yan
Hai Yan
Hangyi Yan
Hao Yan

Hong Yan
Huimin Yan
Jian Yan
Jianhua Yan
Jiazhi Yan
Jie Yan
Jingwen Yan
Jinpei Yan
Jinyuan Yan
Jiusheng Yan
Jizhou Yan
Jun Yan
Koon-Kiu Yan
Lily Yan
Long Yan
Meilying Yan
Qi Yan
Qingyun Yan
Shaoze Yan
Shian-Jang Yan
ShiDu Yan
Shuangchun Yan
Tianyi Yan
Ting Yan
Wenjun Yan
Xiaoyong Yan
Xiao-Yong Yan
Xin Yan
Xingbin Yan
Xuedong Yan
Xuefeng Yan
Ying Yan
Yonghong Yan
Yongmin Yan
Yuanqing Yan
Yueming Yan
Yue-Ming Yan
Yun Yan
Yunjun Yan
Yu-Ting Yan
Zengguang Yan
Zhe Yan
Zhenguang Yan
Zhennan Yan
Masamitsu Yanada
Yasuo Yanagi
Hiromi Yanagisawa
Kunio Yanagisawa
Robert Yanagisawa
Motoko Yanagita
Ruth Yanai
Brian Yandell
Timothy Yandle
Alberto Yáñez
Laura Yañez Espinosa
Carlos Yañez-Arenas
Maria Yanez-Mo
Aiguo Yang
Aijun Yang
Albert Yang
Angela Yang
Annan Yang
Bao Yang
Baofeng Yang
Baoxue Yang
Bin Yang
Bing Yang Yang
Bo Yang
C. Yang
Chang-Hao Yang
Changjun Yang
Changtong Yang
Chao Yang
Chaoxing Yang
Chao-Yie Yang
Chaoyong Yang
Charles Yang
Chen-Chang Yang
Chengfeng Yang
Chengwu Yang
Chengzhong Yang
Chih-Jen Yang
Chih-Tsung Yang
Chih-Yu Yang
Chinhua Yang
Chin-Ying Yang
Chiou-Ying Yang
Chi-Rei Yang
Chuanwei Yang
Chung-Lin Yang
Chunping Yang
Chunwu Yang
Cih-Wei Yang
Cui Yang
Da Yang
Daiwen Yang
Dapeng Yang
Da-Qing Yang
David Yang
Delong Yang
Dongya Y. Yang
Dongzi Yang

Eddy Yang
Ence Yang
Fanmuyi Yang
Fei Yang
Feng Yang
Fengtang Yang
Feng-Yi Yang
Gaihe Yang
George Yang
Guangcan Yang
Guangxiao Yang
Guang-Yu Yang
Guo-Yuan Yang
Guozheng Yang
Haesik Yang
Haifeng Yang
Haining Yang
Haizhao Yang
Hanchun Yang
Hong Yang
Hongtian Yang
Horng-Jyh Yang
Hsin-Chou Yang
Hua Yang
Huadong Yang
Huan Yang
Huangtian Yang
Hui Yang
Huijie Yang
Huilin Yang
Hung-Chih Yang
Hushan Yang
Ivana Yang
James Yang
Jean Yang
Jenny J. Yang
Jer-Yen Yang
Jiachen Yang
Jiajia Yang
Jialiang Yang
Jian Yang
Jianbo Yang
Jianha Yang
Jianhua Yang
Jianyi Yang
Jichun Yang
Jie Yang
Jingchun Yang
Jingjing Yang
Jinpu Yang
Jiongjiong Yang
Jun Yang
Junning Yang
Jun-Yi Yang
Junyuan Yang
Justin Cheng-Ta Yang
Jyh-Chin Yang
K. Yang
Kai Yang
Kai-Chien Yang
Kailin Yang
Kaiping Yang
Kedi Yang
Ke-hu Yang
Kuen Cheh Yang
Kun Yang
Kuo-Liang Yang
Lee-Wei Yang
Li Yang
Liang Yang
Lifei Yang
Lifen Yang
Lijian Yang
Lijun Yang
Li-Jun Yang
Lili Yang
Lin Yang
Ling Yang
Lingling Yang
Liping Yang
Li-Tan Yang
Litao Yang
Liu Yang
Lixia Yang
Li-Ye Yang
Mary Yang
Meng Yang
Meng-Han Yang
Michele Yang
Mijia Yang
Min Yang
Mu Yang
Ning Yang
Nong Yang
Peizeng Yang
Peng Yang
Phillip Yang
Ping Yang
Ping-Chang Yang
Qian Yang Yang
Qiang Yang
Qifeng Yang

Qin Yang
Qing-Cheng Yang
Qinglin Yang
Qingwu Yang
Qingyong Yang
Raymond Yang
Rong-Sen Yang
Ruey-Bing Yang
Seung Yang
Seungmi Yang
Shang-Hsun Yang
Shao-Nian Yang
Sheng Yang
Shengjie Yang
Shengping Yang
Sheng-Shun Yang
Sheng-Xiang Yang
Shi Yang
Shiyou Yang
Shouhui Yang
Shu Yang
Shu-Hua Yang
Shuman Yang
Shunkun Yang
Shwu-Huey Yang
Sihyung Yang
Siyoung Yang
Sizhong Yang
Songguang Yang
Stephen Yang
Su Yang
Tae-Jin Yang
Tai-Hua Yang
Tao Yang
Tewu Yang
Tianyun Yang
Tie-Lin Yang
Ting-Bao Yang
Tse-Yen Yang
Tsuey-Ching Yang
Tsung-Lin Yang
Wan Yang
Wancai Yang
Wankou Yang
Wei Yang
Wei Yuan Yang
Weidong Yang
Weihua Yang
Wei-Lei Yang
Wen-Bin Yang
Wencai Yang
Wen-Chin Yang
WenLong Yang
Wentao Yang
Wen-Yi Yang
X. Frank Yang
Xianfeng Yang
Xiao Yang
Xiaobao Yang
Xiaodong Yang
Xiaofan Yang
Xiaohe Yang
Xiaoju Yang
Xiaokui Yang
Xiaoli Yang
Xiao-Ming Yang
Xiaoyan Yang
Xin Yang
Xin-Chun Yang
Xingbo Yang
Xing-Ke Yang
Xinmai Yang
Xiyan Yang
Xudong Yang
Xue Yang
Xueming Yang
Xuerui Yang
Xuexia Yang
Xueyun Yang
Ya Yang
Yajun Yang
Yan Yang
Yang Yang
Yangfan Yang
Yanqi Yang
Yanyan Yang
Ye Yang
Yi-Hsin Yang
Yimin Yang
Ying Yang
Ying-Ying Yang
Yinmo Yang
Yong Yang
Yu Yang
Yubin Yang
Yuchen Yang
Yun Yang
Yun-Fa Yang
Yungjen Yang
Yun-Gui Yang
Yushe Yang
Yuting Yang

Yu-Xiao Yang
Zaili Yang
Zengjin Yang
Zhang Yang
Zhanlong Yang
Zhaohai Yang
Zhen Yang
Zhenglin Yang
Zhi Yang
Zhi Min Yang
Zhijun Yang
Zhi-Jun Yang
Zhi-Ling Yang
Zhong-Nan Yang
Zhongxia Yang
Zhongzhou Yang
Ziyin Yang
Zu-Jun Yang
Emilio Yanguez
Elizabeth Yanik
Yael Yaniv
Thomas Yankee
Gustavo Yannarelli
Joseph Yanni Gerges
Junko Yano
Ryoichi Yano
Shozo Yano
Yoshihiko Yano
Yuichiro Yano
Chen Yanover
Marcelo Yanovsky
Bing Yao
Bo Yao
Chaoqun Yao
Chengcan Yao
Gaiqi Yao
Hai Yao
Hong Yao
Hongwei Yao
Hui Yao
Jia Yao
Jianxiu Yao
Jinfu Yao
Jing Yao
Jun Yao
Kang Yao
Li Yao
Lin Yao
Linong Yao
Lixia Yao
Quan-Hong Yao
Shuqiao Yao
Shuyu Yao
Song Yao
Wei Yao
Wenjun Yao
Xiaoxi Yao
Xincheng Yao
Yanhua Yao
Yao Yao
Ye Yao
Yong-Ming Yao
Yuan Yao
Yuncong Yao
Zemin Yao
Hong Kai Yap
Lee Fah Yap
Yit-Sheung Yap
Manisha Yapa
Christos Yapijakis
Hakan Yarali
Kevin Yarasheski
Oded Yarden
Masaru Yarime
Nagendra Sastry Yarla
Mohammad Yarmohammadian
James Yarmolinsky
Kelly Yarnell
Noam Yarom
Ala Yaromina
Marianna Yaron
Viktor Yarotskyy
Felix Yarovinsky
Kielan Yarrow
Joanne Yarwood
Stephanie Yarwood
Erika Yashiro
Masakazu Yashiro
J.K. Yasin
Akihiro Yasoda
Yasunobu Yasoshima
Ben Yaspelkis
Haleh Yasrebi
Taha Yasseri
Annalee Yassi
Mohamed Yassin
Takanori Yasu
Hideo Yasuda
Hideto Yasuda
Hiroyuki Yasuda
Satoshi Yasuda
Shinsuke Yasuda

Takashi Yasuda
Tomohiro Yasuda
Takao Yasuhara
Shin Yasui
Teruhito Yasui
Keiko Yasumatsu
Shinobu Yasuo
Akihito Yasuoka
Hidekata Yasuoka
Akiba Yasutada
Paul Yaswen
Junichi Yatabe
Marília Yatabe
Christian Yates
Deborah Yates
Matthew Yates
Patsy Yates
Thomas Yates
Andriy Yatsenko
Jeffrey Yau
Stephen Yau
Joseph Yavitt
Duran Yavuz
Metin Yavuz
Sahzene Yavuz
Utku Yavuz
Makoto Yawata
Alfred Yawson
Josef Yayan
Ilhan Yaylim
Takuya Yazawa
Mohammad Yazdani
Jinoos Yazdany
S. Yazici
Chuyang Ye
Ding-Wei Ye
Dong-Qing Ye
Feng Ye
Gong-Yin Ye
Guoyou Ye
Haihui Ye
Hang Ye
Hilda Ye
Hong Ye
Hui Ye
Huijing Ye
Jiangfeng Ye
Jianqiang Ye
Li Ye
Lin Ye
Ling Ye
Meixia Ye
Min Ye
Ming Ye
Naihao Ye
Ning Ye
Rongzhong Ye
Siying Ye
Sunyue Ye
Tiantian Ye
Wenwu Ye
Wuwei Ye
Xiao Qi Ye
Xingguo Ye
Xingqian Ye
Xuehua Ye
Yihong Ye
Ying-Hui Ye
Yuxiang Ye
Yuzhen Ye
Zhiqiang Ye
Maurice Yeadon
Mark Yeager
Chen-Hsiang Yeang
Samuel Yearman
Alison Yeates
Karen Yeates
Sara Yeatman
Karin Yeatts
Kojo Yeboah-Antwi
Gonzalo Yebra
Narayana Yeddula
Albert Yee
Amy S. Yee
Andrew Yee
Brendon Yee
Douglas Yee
Jennifer Yee
Jerry Yee
Jie Yin Yee
Jiing-Kuan Yee
Marianne Yee
Nelson Yee
Behzad Yeganeh
Herman Yeger
Michel Yegles
Chau-Ting Yeh
Chih-Jung Yeh
Chih-Ko Yeh
Chi-Tai Yeh
Diana Yeh
Elizabeth Yeh

Fang-Cheng Yeh
Gloria Yeh
Hsiang-Yuan Yeh
James Yeh
Kai-Wun Yeh
Kuo-Chen Yeh
Ming-Lun Yeh
Shauh-Der Yeh
Ting-Ting Yeh
Yi-Chun Yeh
Rachel Yehuda
Adoke Yeka
Ajay Yekkirala
Syam Kumar Yelamanchi
José Yélamos
Samantha Yeligar
Alexei Yeliseev
Sailu Yellaboina
Chandra Yelleswarapu
Chen-Tung Yen
Chihfeng Yen
David Yen
David H.T. Yen
Hung-Rong Yen
Irene Yen
Nai-Shing Yen
Paul Yen
Shen-Horn Yen
Shirley Yen
Tim Yen
Tzu-Chen Yen
Yi-Hao Yen
Midori Yenari
Gorsev Yener
Yener Yeni
Jenna Yentes
Chew Chieng Yeo
In-Sung Yeo
Ronald Yeo
Seung-Gu Yeo
Tsin Yeo
Carl Yeoman
Yoon Keng Yeong
Levon Yepiskoposyan
Venkata Yeramilli
Can Yerebakan
Alfred Yergey
Martine Yerle
Laxmi Yeruva
Marc Yeste Velasco
Chun-Yan Yeung
David Yeung
Man Lung Yeung
Simon Yeung
Susanna Yeung
Victoria Yeung Wai Lan
Pierre Yger
Bin Yi
Gi-Hwan Yi
Huiyu Yi
Jing Yi
Jingang Yi
Juneho Yi
Keke Yi
Li Yi
Min Yi
Nengjun Yi
Shuhua Yi
Tao Yi
Yu-Jun Yi
Zhengping Yi
Zhenzhen Yi
An Yihua
Anthony Yii
Adem Yildirim
Eda Yildirim
Abdullah Yildiz
Burak Yilmaz
Hakki Yilmaz
Huseyin Yilmaz
Levent Yilmaz
Sun Young Yim
Changchang Yin
De-Tao Yin
Donghua Yin
Dongmin Yin
Dwight Yin
Erwei Yin
Gavin Yin
Guowei Yin
Haitao Yin
Hengfu Yin
Hua-Bin Yin
J. Yin
Jia Yin
Jiaoyang Yin
Jie Yin
Jiehui Yin
Jieyun Yin
Jin Yin
Jing Dong Yin
Jinlong Yin

Ji-Ye Yin
Jun Yin
Lei Yin
Liya Yin
Meng Yin
Ming Yin
Qingqin Yin
Rui Yin
Ruichuan Yin
Ruohe Yin
Sha Yin
Shen Yin
Shi-An Yin
Viravuth Yin
Wang Yin
Wen-Yao Yin
Xiaoxia Yin
Xinyou Yin
Xue-Ren Yin
Yanbin Yin
Yulong Yin
Zuowei Yin
Louis-Marie Yindom
Chaoran Ying
Gui-Shuang Ying
Hao Ying
Leslie Ying
Michael Ying
Shihui Ying
Tianlei Ying
Tiejin Ying
Wen Ying
Liu Ying Bin
Ai Kia Yip
Connie Yip
Cyril Yip
Peter Yip
Kesetebirhan Yirdaw
Sami Yli-Piipari
Victor Yman
Arne Yndestad
Nigel Yoccoz
Larissa Yocom
Martha Yocupicio-Monroy
Jonathan Yoder
Mervin Yoder
Michael Yodzis
Masafumi Yohda
Shoji Yokobori
Aki Yokohama
Hideki Yokoi
Taichi Yokokawa
Kyoko Yokomori
Tomoya Yokota
Toshifumi Yokota
Hitoshi Yokoyama
Kazuaki Yokoyama
Shinji Yokoyama
Yoko Yokoyama
Sadiye Yolcu
Robert Yolken
Elad Yom-Tov
Arfi Yonathan
Kimio Yonesaka
Mitsutoshi Yoneyama
Hideo Yonezawa
Jeongsik Yong
Jose Yong
Kelvin Yong
Min Hooi Yong
Wei-Peng Yong
Gerald Yonga
Qin Yonghua
Han Sang Yoo
Jung-Yoon Yoo
Ki-Oug Yoo
Tae-Hyun Yoo
Yun Joo Yoo
A-Rum Yoon
Do-Young Yoon
Hyung-Jin Yoon
John Yoon
Joo Chun Yoon
June-Sun Yoon
Karina Yoon
Keejung Yoon
Kyung Chul Yoon
Sang Min Yoon
Seung Kew Yoon
Seung-Yong Yoon
Sungwon Yoon
Woong Yoon
Yeonyee Yoon
Mark Yorek
Jeremy Yorgason
Takashi Yorifuji
Ian York
Eric Yorkston
Hiderou Yoshida
Hiroaki Yoshida
Hiroshi Yoshida
Kentaro Yoshida

Kunito Yoshida
Masahito Yoshida
Masaki Yoshida
Naoko Yoshida
Nobuya Yoshida
Sanichiro Yoshida
Satoshi Yoshida
T. Yoshida
Tadashi Yoshida
Yoshio Yoshida
Yuichi Yoshida
Yutaka Yoshida
Hiroki Yoshihara
Hiroyuki Yoshihara
Sasaki Yoshihiro
Akiomi Yoshihisa
Yuichi Yoshii
Eisho Yoshikawa
Hiroto Yoshikawa
Kenichi Yoshikawa
Takahiro Yoshikawa
Takeshi Yoshikawa
Kayo Yoshimatsu
Kazuto Yoshimi
Keitaro Yoshimoto
Takayuki Yoshimoto
Kenjiro Yoshimura
Natsue Yoshimura
Reiji Yoshimura
Shige Yoshimura
T. Yoshimura
Takesumi Yoshimura
Yoshihiro Yoshimura
Timothy Yoshino
Jun Yoshioka
Keiji Yoshioka
Yosuke Yoshioka
Yasuhide Yoshitake
Kohei Yoshiyama
Yasumasa Yoshiyama
Tomokazu Yoshizaki
Christian Yost
Christopher Yost
Ihor Yosypiv
Marcel Yotebieng
Raquel Yotti
Chun You
Dahui You
Danzhen You
Emily (Chuanmei) You
Feng You
Jingjing You
Jinsheng You
Lidan You
M. You
San-Lin You
Sylvaine You
Tongjian You
Weiwei You
Zhiqiang You
Ada Youk
Hyun Youk
Adel Youkhana
Mike Youle
Bonnie Youmans
Jeehee Youn
Seock-Won Youn
Mehwish Younas
Naji Younes
Alexandra Young
Andrew Young
Antony Young
Benjamin Young
Danielle Young
Heather Young
Hillary Young
Iain Young
Jared Young
Jason Young
Jette Young
Karen C. Young
Ki Lee Young
Kristin Young
Kymberly Young
Marian Young
Martin Young
Mary Young
Melissa Young
Michael Young
Nathan Young
Neil Young
Pampee Young
Paul Young
Rachel Young
Robert Young
Sera Young
Simon Young
Won-Bin Young
Yuan-Nan Young
Nicholas Youngblut
Jane Younger
Jarred Younger

Robert Youngquist
Shawn Youngstedt
Malik Yousef
Amy Yousefi
Maryam Yousefi
Reza Yousefi-Nooraie
Nasser Yousif
George Youssef
Noha Youssef
Seema Yousuf
Ceylan Yozgatligil
Lidia Yshii
Anders Ytterberg
Bin Yu
Bing Yu
Cai-Guo Yu
Chack-Yung Yu
Chang Yu
Changhe Yu
Chen-Ping Yu
Chin Sheng Yu
Chong-Jen Yu
Chuanhe Yu
Danxia Yu
Dao-Yi Yu
Deyue Yu
Dianke Yu
Douglas Yu
Elizabeth Yu
Evan Yu
Fang-Fang Yu
Fangyuan Yu
Feiqiao Yu
Fei-Yuan Yu
Feng Yu
Fengyan Yu
Guanghui Yu
Guanzhen Yu
Guihai Yu
Guizhen Yu
Guojun Yu
Guozhong Yu
Haitao Yu
Hao Yu
Hengyong Yu
Hong Yu
Honglin Yu
Hongwei Yu
Hsien-Chung Yu
Hsin-Hui Yu
Hui Yu
Hung-Hsiang Yu
Jack Yu
James Yu
Jen-Shiang Yu
Jeremy Yu
Jia Yu
Jialin Yu
Jian Yu
Jin Yu
Jindan Yu
Jing Yu
Jingquan Yu
Jinze Yu
Jiyang Yu
Junping Yu
Kai Yu
Kailiang Yu
Ke-Da Yu
Li Yu
Lianchun Yu
Linda Yu
Lingyun Yu
Long Yu
Lu Yu
Luis Yu
Ly-Mee Yu
Maoqun Yu
Miao Yu
Min Yu
Mingjian Yu
Ming-Jiun Yu
Minli Yu
Nanfang Yu
Ning Yu
Pengtao Yu
Pengzhi Yu
Qiang Yu
Qigui Yu
Run Yu
Rwei-Ling Yu
S.W. Yu
Seongjin Yu
Seong-Jin Yu
Shan Ping Yu
Shaoyong Yu
Shen Yu
Shengyuan Yu
Shiyuan Yu
Shudong Yu
Simin Yu
Su Jong Yu

Teng Yu
Tianwei Yu
Tong-Kui Yu
Wei Yu
Weijie Yu
Weikuan Yu
Wen-Chung Yu
Xianghui Yu
Xiaohua Yu
Xiaojia Yu
Xiaojun Yu
Xiaoping Yu
Xiaozhong Yu
Xinhua Yu
Xinxiao Yu
Xiuping Yu
Xue Yu
Yan Yu
Yao Yu
Yong Yu
Yude Yu
Yuhe Yu
Yunsong Yu
Zhen Yu
Zhenwei Yu
Zhiqiang Yu
Zhisheng Yu
Zhitong Yu
Zhongtang Yu
Ziniu Yu
Zitong Yu
Chun Yuan
Ganjun Yuan
Guoyong Yuan
Hua Yuan
Hui Yuan
Huiping Yuan
Ji Yuan
Jian Yuan
Jiazheng Yuan
Jing Yuan
Jingsong Yuan
Junhua Yuan
Junying Yuan
Kai Yuan
Kebin Yuan
Li Yuan
Lijuan Yuan
Lin Yuan
Meijing Yuan
Meng Yuan
Nanci Yuan
Pu-Qing Yuan
Quan Yuan
Rong Yuan
Shi-Min Yuan
Shuangrong Yuan
Ting Yuan
Weihong Yuan
Xianglin Yuan
Xu Yuan
Xue Yuan
Xueli Yuan
Yan Yuan
Yonggui Yuan
Youlu Yuan
Yuan Yuan
Ze-Chun Yuan
Zengqiang Yuan
Zhenfeng Yuan
Zhilin Yuan
Zhi-Yong Yuan
Xie Yuansheng
Kuaybe Yucebilgili Kurtoglu
Yeni Yucel
Bing Yue
Changwu Yue
Feng Yue
Hong Yue
Hua Yue
Jia-Xing Yue
Jun Yue
Peng Yue
Wai-Mun Yue
Weihua Yue
Wyatt Yue
Xiaodong Yue
Yun Yue
Kit-San Yuen
Lilly Yuen
Nancy Yuen
Peter Yuen
Shiu Yuen
Vadim Yuferov
Sung Yuh
Shao Yu-Hsuan Joni
Kunio Yui
Jae-Min Yuk
Michi Yukawa
Ayfer Yukselen
Cai-Hong Yun
Eun Ju Yun

Jong Yun
Jong Won Yun
Katherine Yun
Low Yun
Ui Jeong Yun
Ye Yun
Jose Yunes
José Yunes
Chee Fu Yung
Mingo Yung
W.K. Alfred Yung
Peter Yunker
Theodore Yuo
Natalia Yurlova
Andrew Yurochko
Anastasia Yurtseva
Anna Yusa
Paul A. Yushkevich
Stuart Yuspa
Jason Yustein
H. Yusuf
Mohd Yusuf
Nabiha Yusuf
Salim Yusuf
Katherine Yutzey
Ikuko Yuyama
Alexander Yuzhakov
Monika Zaba
Javier Zabalza
Brian Zabel
Darya Zabelina
Dan Zabetakis
Katarzyna Zabielska-Koczywas
Vitalii Zablotskii
Rouhollah Zaboli
Olga Zaborina
Gaetano Zaccara
Francesco Zaccardi
Mauro Zaccarelli
Edoardo Zaccaria
Serena Zacchigna
Asterios Zacharakis
Ulrich Zachariae
Ioannis Zacharioudakis
Kai Zacharowski
L.R. Zacharski
Martin Zack
Matthew Zack
Eldad Zacksenhaus
Cindy Zadikoff
Ruth Zadoks
Erno Zádor
Antonio Zadra
Tino Zaehle
Romi Zaeske
Afia Zafar
Zafar Zafari
Mai Zafer
Mirko Zaffagnini
Kareem Zaghloul
Mohamed Saad Zaghloul
Giovanni Zagli
Miriam Zago
Osvaldo Zagordi
Maxim Zagoskin
Noel Zagre
Ana-Maria Zagrean
Marta Zagrebelsky
Jean-Ralph Zahar
Greg Zaharchuk
Zohreh Zahedi
Asgar Zaheer
Maliha Zahid
Alan Zahler
Sandrine Zahn
Caroline Zahn-Waxler
Stephen Zahorian
Pavel Zahorik
Ivan Zahradník
Dalia Zahran
Thomas Zahrt
Joseph Zaia
A. Zaid
Dahlia Zaidel
Ronen Zaidel-Bar
Alexander Zaika
Alexey Zaikin
Mukti Zainuddin
Dietmar Zaiss
Alexey Zaitsev
Kei Zaitsu
Roman Zajac
Nadia Zakaria
Hovakim Zakaryan
Dan Zakay
Emily Zakem
Alexey Zakharov
Hasan Zaki
Kristen L. Zakian
Harold Zakon
Tanya Zakrison
Dariusz Zakrzewicz

Magdalena Zakrzewska
Julia Zakrzewski
Patrycja Zalas-Wiecek
Bernard Zalc
John Zalcberg
Maciej Zalewski
Charles Zaloudek
Christoff Zalpour
Gil Zalsman
Martin Zaltz Austwick
Myron Zalucki
Yoshito Zamami
Junaid Zaman
Luis Zaman
Raiyan Zaman
Rita Zamarchi
Dmitriy Zamarin
Filippo Zambelli
Tomaso Zambelli
Gerard Zambetti
Paolo Zambonelli
Fausto Zamboni
Paolo Zamboni
Gabriel Zambrano Rey
Aleksandra Zambrowicz
Eli Zamir
Rosario Zamora
Francisco Javier Zamora-Camacho
Gorka Zamora-Lopez
Rose Zamoyska
Fernando Zampieri
Stefania Zampieri
Massimiliano Zampini
Maria Giulia Zampino
Rosa Zampino
Natasha Zamudio
Antonio Zamuner
Linsen Zan
Yunlong Zan
Margarete Zanardo Gomes
Antonio Zanatta
Dan Zandberg
Eric Zander
Esther Zander
Thorsten Zander
Francesca Zanderigo
Keivan Zandi
Fabio Zanella Farneda
Regis Zanette
Orazio Zanetti
Chuanli Zang
Chunpeng Zang
Mengyan Zang
Qun Zang
Xiujuan Zang
Yingan Zang
Jochen Zange
Ulrich Zanger
Thomas Zangle
Massimiliano Zaniboni
Elisa Zanier
Laura Zanin
Mark Zanin
Barbara Zanini
Damien Zanker
Johannes Zanker
Amy Zanne
Markella Zanni
Gian Franco Zannoni
Clément Zanolli
Giuseppe Zanotti
Ilaria Zanotti
Stefano Zanotti
P. Zanotti-Fragonara
Alfeu Zanotto-Filho
Pat Zanzonico
Ewa Zaobidna
Agustín Zapata
Lauren Zapata
Ma José Zapata
Antonio Zapparata
Laura Zapparoli
Martina Zappaterra
Giacomo Zara
Oscar Zaragoza
Angel Zarain-Herzberg
Gokmen Zararsiz
Pascale Zaraté
Apostolos Zaravinos
Peter Zarb
Sam Zarbakhsh
Marco Zarbin
Ralf Zarbock
Doron Zarchy
Maria Zarcone
Rafael Zardoya
Mohsen Zare
Parisa Zare
Richard Zare
Gernot Zarfel
Svetislav Zaric
Jose Zariffa

Neven Zarkovic
Daniela Zarnescu
Paul Zarogoulidis
Kostas Zarpas
Ainhoa Zarraga
Raffaele Zarrilli
Ali Zarrinpar
Tal Z. Zarsky
Marc Zaruba
Natalia Zarzeczna
Jan Zarzycki
Michael Zasloff
Kurt Zatloukal
Mayana Zatz
Roberto Zatz
Michael Zaugg
Giorgio Zauli
Egija Zaura
Lisa Zaval
Alejandro Zavala-Hurtado
Ricardo Zavala-Yoe
Anna Zavodni
Joanna Zawacka-Pankau
Rainer Zawatzky
Hossam Zawbaa
Antoine Zazzo
Andrea Zbinden
Magali Zbinden
Zachery Zbinden
Anselm Zdebik
Zenon Zdunczyk
Sherali Zeadally
Qendrim Zebeli
Simon Zebelo
Abir Zebian
Leslie Zebrowitz
Marta Zebrowska
Manuela Zebunke
Christoph Zechner
Ellen Zechner
Ulrich Zechner
Aglaia Zedlitz
Marcel Zeelenberg
Rene Zeelenberg
Anat Zeelim-Hovav
Luiz Zeferino
David Zegers
Clement Zeh
Hebert Zeh
Gianguglielmo Zehender
Christoph Zehendner
Grigor Zehirov
Daniel Zehnder
James Zehnder
James L. Zehnder
E. Paul Zehr
Asad Zeidan
Youssef Zeidan
Adam Zeilinger
Katrin Zeilinger
Susanne Zeilinger
Jill Zeilstra-Ryalls
Wadih Zein
Robert Zeiser
Axel Zeitler
Jennifer Zeitlin
Pamela Zeitlin
Julia Zeitlinger
Christina Zeitz
Amir Zeki
Adrian Zelazny
Philip Zelazo
Noam Zelcer
Darryl Zeldin
John Zeldis
Andrew Zele
Darla Zelenitsky
S. Zelenitsky
John Zelenski
Moriel Zelikowsky
Igor Zelko
Ethan Zell
Kathy Zeller
Sebastian Zellmer
J.P. Zellweger
Jonathan Zelner
Sven Zels
Cedric Zeltz
Edith Zemanick
Olivier Zemb
Sergio Zeme
Erika Zemkova
Adam Zemla
John Zempel
Janos Zempleni
Graham Zemunik
Kazem Zendehdel
Benjamin Zendejas
Benjamin Zendel
Lauren Zenewicz
An Zeng
Bai-Yun Zeng

Bo Zeng
De-Hui Zeng
Guangming Zeng
Gucheng Zeng
Hao Zeng
Jianbin Zeng
Li Zeng
Lili Zeng
Lingfang Zeng
Ling-Li Zeng
Lixian Zeng
Qiang Zeng
Quan Zeng
Shaohua Zeng
Songjun Zeng
Tao Zeng
Wei Zeng
Wenzhi Zeng
Xiangpei Zeng
Xiangxiang Zeng
Xianying Zeng
Xiaofeng Zeng
Yanru Zeng
Yi Arial Zeng
Yong Zeng
Yukai Zeng
Zhen Zeng
Joseph Zeni
Olga Zeni
Marcos Zenobi
Alexandre Zénon
Thomas Zentall
Juergen Zentek
Rudolf Zentel
Karen Zentgraf
Marcel Zentner
Jacopo Zenzeri
Cristina Silvia Zepeda-Cisneros
Gadi Zerach
Paolo Zerbinati
Gianpaolo Zerbini
Daniel Zerbino
William Zerges
Paula Zermoglio
Magdalena Zernicka-Goetz
Melinda Zeron Mullins
Georgios Zervakis
Eleftherios Zervas
S. Zervoudaki
Gül Zerze
John Zettel
Henrik Zetterberg
Madeleine Zetterberg
Holger Zetzsche
Kirsten Zeuner
Dirk Zeuss
Jose Zevallos
Juan Zevallos
Maximilian Zeyda
Annette Zeyner
Líbia Zé-Zé
Helen Zgurskaya
Olena Zhabenko
Bing Zhai
Jun-Wen Zhai
Li Zhai
Lu Zhai
Peiyong Zhai
Qiwei Zhai
Rihong Zhai
Bin Zhan
Faxian Zhan
Le Zhan
Meng Zhan
Shuai Zhan
Wenbin Zhan
Xiangjiang Zhan
Xianyuan Zhan
Yuanbo Zhan
Zhi-Hui Zhan
Ai- Hua Zhang
Ai-Bing Zhang
Aijun Zhang
Aimin Zhang
A-Mei Zhang
Amy Zhang
An Zhang
An-Sheng Zhang
Baohong Zhang
Baolong Zhang
Bei Zhang
Ben-Gang Zhang
Bin Zhang
Bing Zhang
Bo Zhang
Boyang Zhang
Caiqiao Zhang
Can Zhang
Changqing Zhang
Changwen Zhang
Chang-Yi Zhang
Chao Zhang

Chen Zhang
Cheng Zhang
Chengcheng Zhang
Chengcui Zhang
Chengjun Zhang
Chengzhi Zhang
Chen-Yu Zhang
Chi Zhang
Chu-Long Zhang
Chun Zhang
Chunni Zhang
Chunxi Zhang
Chunxia Zhang
Chunyan Zhang
Chunyu Zhang
Claire Shuiqing Zhang
Dabao Zhang
Dabing Zhang
Dadong Zhang
Dan Zhang
Daopei Zhang
Dawei Zhang
Dayong Zhang
Degan Zhang
Deng-Feng Zhang
Desheng Zhang
Dingxiao Zhang
Dong Zhang
Dongqing Zhang
Dongsheng Zhang
Emma Jingfei Zhang
Faming Zhang
Fan Zhang
Fang Zhang
Feng Zhang
Fengju Zhang
Fuzhong Zhang
Gary Zhang
Ge Zhang
Gengxin Zhang
Gong Zhang
Guanglan Zhang
Guang-Xian Zhang
Guangxiang Zhang
Guangyan Zhang
Guangzhao Zhang
Guimin Zhang
Guiquan Zhang
Guo-Chang Zhang
Guodong Zhang
Guofu Zhang
Guojun Zhang
Guoliang Zhang
Guoqiang Zhang
Guo-Qiang Zhang
Guoquan Zhang
Guo-Zhong Zhang
Haicheng Zhang
Haifeng Zhang
Haitao Zhang
Haiyang Zhang
Haiyu Zhang
Han Zhang
Hanwang Zhang
Hao Zhang
Helong Zhang
Hengwei Zhang
Henry Zhang
Heping Zhang
Hong Zhang
Hongbo Zhang
Hongquan Zhang
Hongsheng Zhang
Hongyan Zhang
Hongying Zhang
Hua Zhang
Huabing Zhang
Huan Zhang
Huaye Zhang
Hui Zhang
Huidan Zhang
Huiming Zhang
J. Zhang
Jennifer Zhang
Jenny Zhang
Jiabao Zhang
Jiacai Zhang
Jiachao Zhang
Jiahua Zhang
Jiaming Zhang
Jian Zhang
Jiang Zhang
Jiangwen Zhang
Jianlei Zhang
Jian-Ping Zhang
Jianqi Zhang
Jianqiang Zhang
Jianyi Zhang
Jianying Zhang
Jianzhen Zhang
Jiaquan Zhang
Jiaxing Zhang

Ji-Chun Zhang
Jie Zhang
Ji-Fang Zhang
Jiiaju Zhang
Jiming Zhang
Jin Zhang
Jinfa Zhang
Jinfen Zhang
Jinfeng Zhang
Jing Zhang
JingJing Zhang
Jingjuan Zhang
Jing-Ren Zhang
Jinjin Zhang
Jinling Zhang
Jinlong Zhang
Jinpeng Zhang
Jinping Zhang
Jinqiang Zhang
Jinsheng Zhang
Jinsong Zhang
Jiwen Zhang
Joe Zhang
Juan Zhang
Jue Zhang
Jun Zhang
Junbin Zhang
JunJie Zhang
Junling Zhang
Junyun Zhang
Kai Zhang
Kang Zhang
Kangling Zhang
Ke Zhang
Kerang Zhang
Kewei Zhang
Kezhong Zhang
Kui Zhang
Kunyan Zhang
Lei Zhang
Leiliang Zhang
Li Zhang
Lianfeng Zhang
Liang Zhang
Liangsheng Zhang
Libiao Zhang
Lifan Zhang
Lijuan Zhang
Lin Zhang
Linglin Zhang
Lingling Zhang
Linjie Zhang
Liping Zhang
Liqin Zhang
Liren Zhang
Lisheng Zhang
Liyi Zhang
Liying Zhang
Liyun Zhang
Long Jiang Zhang
Long-Wa Zhang
Lu Zhang
Lujia Zhang
Luyan Zhang
Mei Zhang
Melvyn Zhang
Mengliang Zhang
Mengxi Zhang
Miao Zhang
Miaomiao Zhang
Min Zhang
Ming Zhang
Mingli Zhang
Ming-Li Zhang
Mingyong Zhang
Mingzhen Zhang
Ming-Zhi Zhang
Min-Ling Zhang
Mu Zhang
Nan Zhang
Ning Zhang
Peipei Zhang
Peng Zhang
Pengfei Zhang
Ping Zhang
Qi Zhang
Qian Zhang
Qian-Ming Zhang
Qianru Zhang
Qibo Zhang
Qijun Zhang
Qin Zhang
Qing Zhang
Qingpeng Zhang
Qingrun Zhang
Qing-Shuo Zhang
Qingyuan Zhang
Qiuwen Zhang
Qiwei Zhang
Qi-Ya Zhang
Quan Zhang
Quanqi Zhang

Quanwei Zhang
Quinjiao Zhang
Ren Zhang
Renyi Zhang
Ri-jun Zhang
Rong Zhang
Rongqing Zhang
Rongxin Zhang
Rui Zhang
Runxuan Zhang
Ruxu Zhang
Sarah Zhang
Sarah X. Zhang
Shanshan Zhang
Shaojie Zhang
Shaowu Zhang
Sheng Zhang
Shenghong Zhang
Shengping Zhang
Shengxiang Zhang
Shi-Bao Zhang
Shi-Hong Zhang
Shijian Zhang
Shi-Ming Zhang
Shixiu Zhang
Shjie Zhang
Shuanglin Zhang
Shuhua Zhang
Shujie Zhang
Shuning Zhang
Shuping Zhang
Shu-Yang Zhang
Sichen Zhang
Simo Zhang
Song Zhang
Songying Zhang
Sufang Zhang
Tao Zhang
Teng Zhang
Tianyu Zhang
Tiemei Zhang
Tongli Zhang
Tongwu Zhang
Tracy Zhang
W. Zhang
W.F. Zhang
Wangfeng Zhang
Wanqi Zhang
Wanyu Zhang
Wei Zhang
Wei-De Zhang
Weidong Zhang
Weihua Zhang
Weijun Zhang
Wen Zhang
Wenbo Zhang
Wenhai Zhang
Wenhong Zhang
Wenjing Zhang
Wenjun Zhang
Wenli Zhang
Wenlong Zhang
Wenlu Zhang
Wenyan Zhang
Wen-Yi Zhang
Xi Zhang
Xian Zhang
Xianchun Zhang
Xiang-Dong Zhang
Xiangqi Zhang
Xiao Zhang
Xiaobo Zhang
Xiaodong Zhang
Xiaoge Zhang
Xiaoguang Zhang
Xiaohong Zhang
Xiaohua Zhang
Xiaoju Zhang
Xiaojun Zhang
Xiaoke Zhang
Xiao-Kun Zhang
Xiaoling Zhang
Xiaoming Zhang
Xiaoqun Zhang
Xiaotian Zhang
Xiaoting Zhang
Xiaowei Zhang
Xifeng Zhang
Xilong Zhang
Xin Zhang
Xinchang Zhang
Xingang Zhang
Xingyi Zhang
Xinhuan Zhang
Xinsheng Zhang
Xinyu Zhang
Xiong Zhang
Xiquan Zhang
Xiufeng Zhang
Xiulan Zhang
Xiurong Zhang
Xue Zhang

Xuehong Zhang
Xueli Zhang
Xuelin Zhang
Xuewu Zhang
Xuexin Zhang
Xueying Zhang
Xue-Ying Zhang
Xu-Xiang Zhang
Yajun Zhang
Yan Zhang
Ya-Nan Zhang
Yangjian Zhang
Yangsong Zhang
Yani Zhang
Yanjin Zhang
Yanjun Zhang
Yanqiong Zhang
Yansheng Zhang
Yaoping Zhang
Yi Zhang
Yi-Cheng Zhang
Yi-Fan Zhang
Yifeng Zhang
Yili Zhang
Yimin Zhang
Yi-Min Zhang
Ying Zhang
Yinghr Zhang
Yingtao Zhang
Yingze Zhang
Yiquan Zhang
Yiran Zhang
Yixuan Zhang
Yiyi Zhang
Yizhai Zhang
Yi-Zheng Zhang
Yong Zhang
Yong-An Zhang
Yong-Biao Zhang
Yongbin Zhang
Yongdeng Zhang
Yonggang Zhang
Yong-Guo Zhang
Yonghe Zhang
Yong-Jie Zhang
Yongkang Zhang
Yong-Mei Zhang
Yongrong Zhang
Yong-Yuan Zhang
Yongzhong Zhang
You Zhang
Yu Zhang
Yuan Zhang
Yubin Zhang
Yue-Miao Zhang
Yufeng Zhang
Yuhang Zhang
Yujing Zhang
Yumiao Zhang
Yun Zhang
Yunlin Zhang
Yunqi Zhang
Yun-Wu Zhang
Yuwei Zhang
Yuxia (Lisa) Zhang
Yuxiang Zhang
Zejun Zhang
Zhancheng Zhang
Zhang-Jin Zhang
Zhanyuan Zhang
Zhaojun Zhang
Zhaoqi Zhang
Zhaowei Zhang
Zhe Zhang
Zhen Zhang
Zheng Zhang
Zhengbin Zhang
Zhengdong Zhang
Zhengsheng Zhang
Zhenguo Zhang
Zhengzhi Zhang
Zhenhai Zhang
Zhen-Lin Zhang
Zhihao Zhang
Zhihong Zhang
Zhijun Zhang
Zhiming Zhang
Zhiqiao Zhang
Zhisheng Zhang
Zhong Yi Zhang
Zhongheng Zhang
Zhongnan Zhang
Zhuhong Zhang
Zi Zhang
Zichuan Zhang
Ziming Zhang
Ziping Zhang
Zizhen Zhang
Zuoyi Zhang
Zuxin Zhang
Aqun Zhao
Bin Zhao

Bingqiang Zhao
Bingyu Zhao
Bingzi Zhao
Chang-Sheng Zhao
Chao Zhao
Chen Zhao
Chenchen Zhao
Dayong Zhao
De Zhao
Dengji Zhao
Di Zhao
Dong Zhao
Dongxin Zhao
Fangqing Zhao
Feng-Qin Zhao
Fuping Zhao
Gang Zhao
Genming Zhao
Guangyu Zhao
Guifang Zhao
Haibo Zhao
Haiqing Zhao
Haitao Zhao
Han Zhao
Helen Zhao
Hong Zhao
Hongwei Zhao
Hongyu Zhao
Hua Zhao
Hui Zhao
Huimin Zhao
Huixian Zhao
Jia-Guo Zhao
Jiajun Zhao
Jiangchao Zhao
Jianmin Zhao
Jiaying Zhao
Jichang Zhao
Jichao Zhao
Jie Zhao
Jin Zhao
Jincun Zhao
Jing Zhao
Jinling Zhao
Jiwei Zhao
Jun Zhao
Jun-Hua Zhao
Juzi Zhao
Kaijun Zhao
Ke Zhao
Kexin Zhao
Kun Zhao
Kuo Zhao
Lan Zhao
Lili Zhao
Lingxia Zhao
Linlu Zhao
Liqin Zhao
Lixia Zhao
Lue Ping Zhao
Maosheng Zhao
Min Zhao
Ming Zhao
Mingbo Zhao
Mingchang Zhao
Ming-Gao Zhao
Mingwei Zhao
Mingzhu Zhao
Ni Zhao
Peng Zhao
Ping Zhao
Qi Zhao
Qijun Zhao
Qing Zhao
Qinglan Zhao
Qingshun Zhao
Qingyuan Zhao
Qinjian Zhao
Qiong Zhao
Rongmin Zhao
Rui Zhao
Shanshan Zhao
Shu Zhao
Sumei Zhao
Tao Zhao
Tiejun Zhao
Ting C. Zhao
Tingning Zhao
Tong Zhao
Tongyan Zhao
Weian Zhao
Weiqiang Zhao
Wenwu Zhao
Wenxiu Zhao
Xiang Yu Zhao
Xiangna Zhao
Xiaohang Zhao
Xiaojing Zhao
Xiaoli Zhao
Xiaoshan Zhao
Xincheng Zhao
Xing Zhao

Xingming Zhao
Xingquan Zhao
Xinqing Zhao
YaE Zhao
Yan Zhao
Yanan Zhao
Yanglu Zhao
Yanping Zhao
Yichuan Zhao
Yidan Zhao
Yingxin Zhao
Ying-Yong Zhao
Yong Zhao
Yongbing Zhao
Yongliang Zhao
Yue Zhao
Yuechao Zhao
Yuejen Zhao
Yunfeng Zhao
Yunsong Zhao
YuPei Zhao
Yutong Zhao
Zhaoyang Zhao
Zheng Zhao
Zhi-Dan Zhao
Zhi-Gang Zhao
Zhiguang Zhao
Zhihui Zhao
Zhi-Jun Zhao
Zhili Zhao
ZhiWei Zhao
Zhizhen Zhao
Zhuohui Zhao
Zibo Zhao
Zongbao Zhao
Alex Zhavoronkov
Irina Zhdanova
Shandian Zhe
Hristina Zhekova
Jie Zhen
Shi Zhen
Yu Zhen
Zonglei Zhen
Bangyou Zheng
Bing Zheng
Bingsong Zheng
Bowen Zheng
Chang Zheng
Chenfei Zheng
Chengchao Zheng
Chunfu Zheng
Chun-Hou Zheng
Dandan Zheng
Du-Ping Zheng
E. Zheng
Enhao Zheng
Haixue Zheng
Heping Zheng
Hongkun Zheng
Huaixin Zheng
Huakun Zheng
Jian Zheng
Jie Zheng
Jing Zheng
Jufeng Zheng
Lei Zheng
Lemin Zheng
Liang Zheng
Liangrong Zheng
Lianming Zheng
Lianyuan Zheng
Ming Zheng
Mingqiang Zheng
Muzi Zheng
Peng-Sheng Zheng
Qi Zheng
Qing Zheng
Qingshan Zheng
Qinsi Zheng
Qiping Zheng
Qiusheng Zheng
Rongqin Zheng
Ruijin Zheng
Shijun Zheng
Shunzhen Zheng
Shusong Zheng
Tiantian Zheng
Wang Zheng
Wei Zheng
Weifan Zheng
Xi Zheng
Xianhu Zheng
Xiaofei Zheng
Xiaolong Zheng
Xiaoping Zheng
Xiaotian Zheng
Xinde Zheng
Xiufen Zheng
Xu Zheng
Xuexing Zheng
Yanting Zheng
Yaowu Zheng

Yi Zheng
Ying Zheng
Yingye Zheng
Yong-Hui Zheng
Yong-Ping Zheng
Yongqiang Zheng
Yong-Qiu Zheng
Yong-Tang Zheng
Yuejiu Zheng
Yuesheng Zheng
Yulong Zheng
Yun-Wen Zheng
Zeyu Zheng
Zhe Zheng
Zheng Zheng
Zhihai Zheng
He Zhengquan
Zhenning Zhenning Cao
Qi Zhi
Cao Zhijun
Anatoly Zhitkovich
Alexander Zholos
Baoliang Zhong
Bineng Zhong
Caiming Zhong
Chunjiu Zhong
Daibin Zhong
Guohua Zhong
Hua Zhong
Jian Zhong
Jiang Zhong
Jian-Hong Zhong
Jianhui Zhong
Jidan Zhong
Mei Zhong
Min Zhong
Nanbert Zhong
Ping Zhong
Qing Zhong
Rui Zhong
Shan Zhong
Shaobin Zhong
Shuping Zhong
Wei Zhong
Wei-De Zhong
Yi Zhong
Zheng Zhong
Zhenhui Zhong
Boris Zhorov
Aizhi Zhou
Beiyun Zhou
Bin Zhou
Bing-Rong Zhou
Bo Zhou
Caicun Zhou
Cindy Zhou
Dan Zhou
Di Zhou
Dongming Zhou
Enhua Zhou
En-Min Zhou
Fei Zhou
Feng Zhou
Gaofeng Zhou
Gordon G. D. Zhou
Guangsheng Zhou
Guangwei Zhou
Guohua Zhou
Guohui Zhou
H. Zhou
Hai-Chao Zhou
Haiyan Zhou
Hang Zhou
Haoyan Zhou
Heling Zhou
Huakun Zhou
Hui Zhou
Huiping Zhou
Jiahua Zhou
Jianjiang Zhou
Jianli Zhou
Jie Zhou
Jiliang Zhou
Jing Zhou
Jing-Jiang Zhou
Jingsong Zhou
Jinyuan Zhou
Jiyong Zhou
Jizhong Zhou
Jun Zhou
Junhong Zhou
Kai-Qing Zhou
Lan Zhou
Lei Zhou
Li Zhou
Liang Zhou
Libin Zhou
Lifang Zhou
Lijun Zhou
Ling Zhou
Liqun Zhou
Lufang Zhou

Luping Zhou
Man Zhou
Mi Zhou
Minchuan Zhou
Mingqi Zhou
Peng Zhou
Penghui Zhou
Ping Zhou
Pingyu Zhou
Qiang Zhou
Qing Zhou
Qixin Zhou
Renlai Zhou
Ruimin Zhou
Shang-Ming Zhou
Shiliang Zhou
Shuanhu Zhou
Shuntai Zhou
Sichang Zhou
Siru Zhou
Tao Zhou
Tianhua Zhou
Tieli Zhou
Tong Zhou
Wang Zhou
Wei Zhou
Weibing Zhou
Weiping Zhou
Wenchao Zhou
Wenya Zhou
X. Zhou
X. M. Zhou
Xiang Zhou
Xiangmei Zhou
Xiao Zhou
Xiaobo Zhou
Xiaochun Zhou
Xiaofeng Zhou
Xiao-Hong Zhou
Xiaojie Zhou
Xiaolai Zhou
Xiaolu Zhou
Xiaoying Zhou
Xin Zhou
Xingtao Zhou
Xuanwei Zhou
Xueyuan Zhou
Xuhui Zhou
Yan Zhou
Yang Zhou
Yibiao Zhou
Yihua Zhou
Yi-Hua Zhou
Yingbi Zhou
Yong Zhou
Yongsheng Zhou
Yu Zhou
Yuan Zhou
Yufeng Zhou
Yuling Zhou
Yun Zhou
Yuyu Zhou
Zhekun Zhou
Zhemin Zhou
Zhigang Zhou
Zhi-Gang Zhou
Zhongliang Zhou
Zhong-Shi Zhou
Zhou Zhou
Zhuan Zhou
Banghao Zhu
Baoge Zhu
Benzhong Zhu
Bi Zhu
Bingmei Zhu
Bo Zhu
Changfu Zhu
Changlian Zhu
Chengzhou Zhu
Dajiang Zhu
Dongqiang Zhu
Fan Zhu
Feng Zhu
Feng-Xiang Zhu
Gengping Zhu
Guangyu Zhu
Guanshan Zhu
Haiyan Zhu
Hao Zhu
Haojie Zhu
Heming Zhu
Hong Zhu
Hu Zhu
Hua Zhu
Huaiqiu Zhu
Huilian Zhu
James Zhu
Jane Zhu
Jianhua Zhu
Jieqing Zhu
Jingde Zhu
Jinsong Zhu

Jun Zhu
Junyong Zhu
Kai Zhu
Kun Zhu
Kun Yan Zhu
Liang Zhu
Lifeng Zhu
Lili Zhu
Lin Zhu
Longfu Zhu
Min Zhu
Mu Zhu
Ning Zhu
Pengfei Zhu
Ping Zhu
Qian Zhu
Qian-Hao Zhu
Sen Zhu
Shengwei Zhu
Shimin Zhu
Shuijin Zhu
Tianqing Zhu
Tingyu Zhu
Tongyu Zhu
Wanze Zhu
Wei-Li Zhu
Wei-Ming Zhu
Weiyun Zhu
Wenbo Zhu
Wenhan Zhu
Wentao Zhu
Wenzhen Zhu
Wuqiang Zhu
Xiang-Yang Zhu
Xiangyu Zhu
Xiaodong Zhu
Xiaofeng Zhu
Xiao-Feng Zhu
Xiaolei Zhu
Xiaoping Zhu
Xiaoyuan Zhu
Xinbo Zhu
Xin-Guang Zhu
Xueling Zhu
Xuli Zhu
Yajuan Zhu
Yan Zhu
Yezi Zhu
Yihong Zhu
Yimin Zhu
Ying Zhu
Yinghui Zhu
Yitan Zhu
Yong Zhu
Yongchuan Zhu
Yongzhang Zhu
Yueming Zhu
Yue-Yong Zhu
Yun Zhu
Yunhua Zhu
Yuwen Zhu
Zaichun Zhu
Zezhang Zhu
Zhen Zhu
Zhendong Zhu
Zhengfei Zhu
Zheng-Jiang Zhu
Zhigang Zhu
Zhongjie Zhu
Zhongyu Zhu
Guoqiang Zhuang
Jie Zhuang
Jing Zhuang
Ling Zhuang
Xun Zhuang
Yong Zhuang
Yongbin Zhuang
Zhen Zhuang
Zhihao Zhuang
Ronghua Zhuge
Yang Zhugen
Vita Zhukova
Chuanjun Zhuo
Jiachen Zhuo
Ran Zhuo
Yan Zhuo
Keyan Zhu-Salzman
Xiaolin Zi
Zhike Zi
Panayiotis Ziakas
Muhammad Zia-Ul-Haq
Kazem Zibara
Ran Zichel
Suzanna Zick
Nicolas Ziebarth
Noel Ziebarth
John Ziebuhr
Andrew Zieffler
Joseph Ziegelbauer
Anette-Gabriele Ziegler
Ekhard Ziegler
Martin Ziegler

Melanie Ziegler
Thomas Ziegler
Thomas R. Ziegler
Urszula Zielenkiewicz
Acacio Zielinski
Jacek Zielonka
Ewa Ziemann
Christian Ziener
Krzysztof Zieniewicz
Clemens Zierhofer
Janine Ziermann
Kristina Zierold
James Zieske
Liliya Eugenevna Ziganshina
Michael Zigmond
Anna Zignego
Matteo Zignol
Michal Zigo
Paola Zigrino
Elise Ziipkin
Wilma Zijlema
Andries Zijlstra
Eduard Zijlstra
David Zilberman
Sam Zilioli
Mindaugas Žilius
Marietta Zille
Julie Zilles
Davide Zilli
Peter Zilm
Danila Zimenkov
Gregory Zimet
Francesca Zimetti
Mirko Zimic
Andrea Zimmer
Danna Zimmer
Elizabeth Zimmer
Geraldine Zimmer
Martin Zimmer
Michael Zimmer
Peter Zimmer
Philipp Zimmer
Sebastian Zimmer
Stefan Zimmer
Stefan Zimmerli
Ludovic Zimmerlin
Andrew Zimmerman
Cathy Zimmerman
Emily Zimmerman
Jerry Zimmerman
Molly Zimmerman
Naupaka Zimmerman
Albert Zimmermann
Eckart Zimmermann
Fridolin Zimmermann
Hanna Zimmermann
Henning Zimmermann
Jonas Zimmermann
S.C. Zimmermann
Ari Zimran
Valery Zinchenko
Adriana Zingone
Brian Zink
Janneke Zinkstok
Steven Zinn
Christoph Zinner
Natalia Zinovyeva
Gaurav Zinta
Annetta Zintl
Luca Zinzula
Justyna Ziolkowska
Hubert Ziólkowski
Mark Ziolo
Anna Ziomkiewicz
Djemel Ziou
Peter Zioupos
Ouliana Ziouzenkova
Alexander Zipprich
Vadim Zipunnikov
Marc Zirnsak
Panagiotis Zis
Giovanni Zito
Julie Zito
Karen Zito
Federica Zito Marino
Emanuel Zitt
Armin Zittermann
Elad Ziv
Noam Ziv
Robert Zivadinov
Georg Zizka
Tzvetan Zlatanov Zlatanov
Indre Zliobaite
Sharon Zlochiver
Urszula Zlotek
Stanley Zlotkin
Jaroslaw Zmijewski
Mohammed Znari
Samira Zoa Assoumou
Giovanna Zoccoli
Pierluigi Zoccolotti
Margot Zoeller

Inka Zoernig
Yohathan Zohar
Sarah Zohdy
Irene Zohn
Tsimtsiou Zoi
Jerome Zoidakis
Georg Zoidl
Christos Zois
James Zois
Niklas Zojer
Jerzy Zoladz
Janeta Zoldan
Massoud Zolgharni
Amin Zollanvari
Heinz Zoller
Bengt Zöller
Christoph Zollikofer
Patrick Zollner
Frank Zöllner
Fabiana Zollo
Vasiliy Zolotarev
Sergei Zolotukhin
Serfozo Zoltan
Rebecca Zoltoski
Marielle Zondervan-Zwijnenburg
Mark Zonfrillo
Geng Zong
Shixiang Zong
Xiaopeng Zong
Zhiyong Zong
Samantha Zongaro
Harshal Zope
Regine Zopf
Claudio Zoppi
Giacomo Zoppini
Chiara Zoppiroli
Hrvoje Zorc
Pinar Zorlutuna
Angeles Zorreguieta
Pedro Zorrilla
Silvia Zorrilla
Antonio Zorzano
Changfu Zou
Changliang Zou
Chang-Lin Zou
Cheng Zou
Chunbin Zou
Haidong Zou
Huasong Zou
Jian Zou
Jun Zou
Lan Zou
Lanfang Zou
Ling Zou
Ming-Hui Zou
Qingjian Zou
Qingze Zou
Quan Zou
Shu-Ming Zou
Wei Zou
Xi Zou
Xiangyang Zou
Xuenong Zou
Xukai Zou
Yajie Zou
Yunzeng Zou
Zhen Zou
Zhengting Zou
Zhenhua Zou
Zhi Zou
Amina Zoubeidi
Rita Zrenner
Zsuzsanna Zsengellér
Agustin Zsögön
Eniko Zsoldos
Rebeka Zsoldos
Jian Zu
Zhongliang Zu
Philine zu Ermgassen
Roman Zubarev
Peter Zuber
Ana Zubiaga
B. Zubik-Kowal
Irati Zubizarreta
Chloe Zubrieta
Celia Zubrinich
Gesualdo Zucco
Guido Zuccon
Marco Zucconi
Gianna Zuccotti
Andreas Zucker
Nancy Zucker
Brian Zuckerbraun
Ethan Zuckerman
Alejandro Zucol
Richard Zuerner
Antonio Zuffianò
Micah Zuhl
Laurian Zuidmeer-Jongejan
Stephanie Zuilkowski
Violetta Zujovic
Helen Zulch

Aldo Zullini
Martin Zumarraga
Sharon K. Zumbrunn
Thomas Zumbrunn
Johanna Zumer
L. Zungu
Matthias Zunhammer
Jorge Zuniga
Sonia Zuñiga
Joseph Zunt
Maria Victoria Zunzunegui
Chuantao Zuo
Jian Zuo
Jian-Ping Zuo
Kejun Zuo
Li Zuo
Nianming Zuo
Rujuan Zuo
Wangmeng Zuo
Yi Zuo
Yongchun Zuo
Zhiyi Zuo
Manja Zupan
Nicole Zurcher
Amer Zureikat
Dejan Zurovac
Mateusz Zurowski
Martin Zuschin
Mary Zutter
Coert Zuurbier
Elena Zuykova
Ana Zuzuarregui
Elena Zvereva
Elika Zvesk
Renata Zvyagilskaya
Marcel Zwahlen
Sabine Zwakenberg
Alix Zwane
Mark Zwart
Joost Zwartenkot
Leo Zwarts
Christiane Zweier
Werner Zwerschke
Janusz Zwiazek
Robert Zwitser
Mark Zwolinski
Andrew Zydney
Wojciech Zygner
Mark Zylka
Sa'ed Zyoud
Nicholas Zyromski
Dorota Zysko
Sharon Zytynska
